# Gold-catalyzed stereoselective cycloisomerization of allenoic acids for two types of common natural γ-butyrolactones

**DOI:** 10.1038/s41467-018-03894-6

**Published:** 2018-04-25

**Authors:** Jing Zhou, Chunling Fu, Shengming Ma

**Affiliations:** Laboratory of Molecular Recognition and Synthesis, Department of Chemistry, Zhejiang University, 310027 Hangzhou, Zhejiang People’s Republic of China

## Abstract

γ-(*E*)-Vinylic and γ-alkylic γ-butyrolactones are two different types of lactones existing extensively in animals and plants and many of them show interesting biological activities. Nature makes alkylic γ-butyrolactones by many different enzymatic lactonization processes. Scientists have been mimicking the natural strategy by developing new catalysts. However, direct and efficient access to γ-(*E*)-vinylic γ-butyrolactones is still extremely limited. Here, we wish to present our modular allene approach, which provides an efficient asymmetric approach to (*E*)-vinylic γ-butyrolactones from allenoic acids by identifying a new gold complex as the catalyst. Based on this cycloisomerization strategy, the first syntheses of racemic xestospongiene and xestospongienes E, F, G, and H have been realized and the absolute configurations of the chiral centers in xestospongienes E and F have been revised. In addition, by applying a C–O bond cleavage-free hydrogenation, the syntheses of naturally occurring γ-alkylic γ-lactones, (*R*)-4-tetradecalactone, (*S*)-4-tetradecalactone, (*R*)-γ-palmitolactone, and (*R*)-4-decalactone, have also been achieved.

## Abstract (web summary)

Gamma-butyrolactones are widespread in Nature, however direct catalytic methods to access them are limited. Here, the authors report a gold-catalyzed cycloisomerization of allenoic acids to acces γ-butyrolactones and apply it to the asymmetric synthesis of xestospongienes E, F, G, and H and other naturally occurring lactones.

## Introduction

**Fig. 1:**
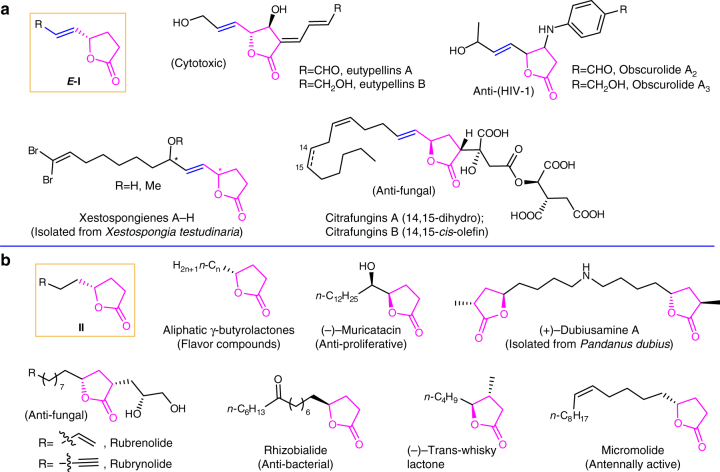
Representative examples of γ-(*E*)-vinylic and γ-alkylic-γ-butyrolactones. **a** Common structure unit and representative examples of γ-(*E*)-vinylic γ-butyrolactones (*E-***I**); **b** Common structure unit and representative examples of γ-alkylic γ-butyrolactones (**II**)

Natural products are a big treasure for human beings, which exhibit rich academic and industrial potentials due to their structural diversity and biological activities. As we know, γ-butyrolactones with common structures of γ-(*E*)-vinylic and alkylic γ-butyrolactones, *E-***I**^1–4^ and **II**^5–10^, exist extensively in nature and many of them have been identified with interesting biological potentials, such as anti-HIV^1^, anti-fungal^2,9^, cytotoxic^3^, anti-bacterial^7^, anti-proliferative^8^ activities, etc., featuring applications in pharmacy (Fig. 1). Some of these lactones, especially for aliphatic γ-butyrolactones, are also common flavor source in plants and food, which involved in several metabolic pathways^11^.

**Fig. 2:**
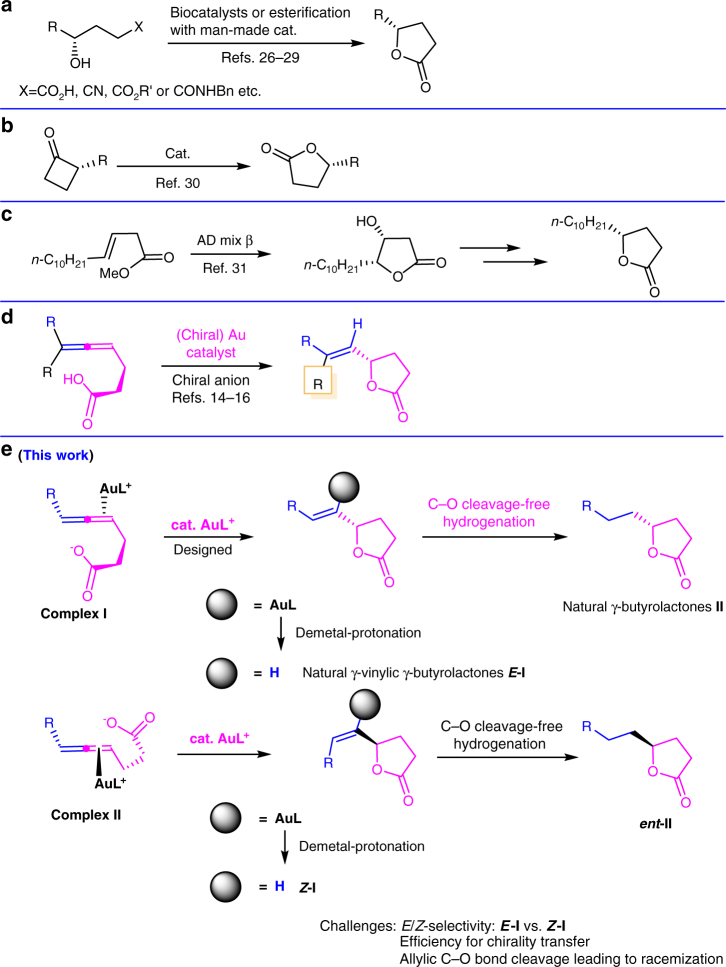
Known approaches and the designed general protocol to γ-butyrolactones *E*-**I** and **II**. **a** Nature’s enzymatic approaches; **b** Baeyer–Villiger oxidation; **c** Alkenoate dihydroxylation; **d** Allene enantioselective approach described in refs. ^14–16^. **e** Au-catalyzed chirality transfer-based asymmetric cyclization of allenoic acids (this work)

So far, very few highly selective asymmetric synthesis of *E-***I**-type γ-butyrolactones with a *trans* 1,3-disubstituted C=C bond has been reported^12,13^. The approaches for the synthesis of unique γ-(2,2-disubstituted or 1-iodo) vinylic γ-butyrolactones^14–18^ are not applicable for the synthesis of the natural γ-butyrolactones due to the substrate limitation. It is well reasoned that in the Au-catalyzed enantioseletcive approach the control of *E*/*Z* selectivity and enantioselectivity are most likely the challenge when 6-mono-substituted allenoic acids were used (Fig. 2d)^14–16^. We envisioned a Au-catalyzed cycloisomerization^19–25^ of optically active 4,5-alkadienoic acids, readily available from terminal alkynes and aldehydes, for the direct access to various natural and non-natural enantioenriched *E-***I**-type common γ-butyrolactones, which could further be easily hydrogenated to provide naturally occurring γ-alkylic γ-butyrolactones **II** (Fig. 2e). The latter was usually prepared via the lactonization (Fig. 2a)^26–29^, Baeyer-Villiger oxidation (Fig. 2b)^30^, and dihydroxylation-lactonization-elimination (Fig. 2c), etc^31–34^. The challenge for strategy in Fig. 2e is the efficiency of axial-to-central chirality transfer^35–43^ and the control of *Z*/*E* selectivity (*E*-**I** vs. *Z*-**I**, which after hydrogenation would afford the enantiomers of lactones **II**, respectively, thus, leading to a much lower ee) during the cyclic anti-nucleometalation and the efficiency of the proto demetalation process to deliver the required 1,3-disubstituted *E*-C=C bond finishing the catalytic cycle. Overall it is highly desirable to identify a suitable ligand **L** for a much more stable complex **I** over complex **II**.

Herein, we present the highly stereoselective cycloisomerization of optically active 5-monosubstituted 4,5-alkadienoic acids affording various non-natural and natural enantioenriched γ-(*E*)-alkenyl-γ-butyrolactones by using AuCl(LB-Phos) as the catalyst.

## Results

### Synthesis of AuCl(LB-Phos)

**Fig. 3:**
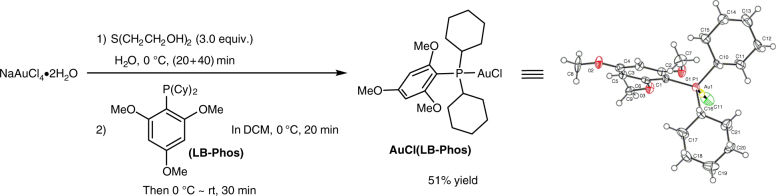
Complex AuCl(LB-Phos). Synthesis and ORTEP representation of AuCl(LB-Phos)

At the beginning, we treated (*R*_a_)-4,5-tridecadienoic acid (*R*_a_)-**5a** (for its synthesis from aldehyde and terminal alkyne, see: Supplementary Tables 1 and 2) as the model substrate. After screening of some commonly used gold catalysts such as AuCl, AuCl(IPr), Au_2_Cl_2_(dppm), Au_2_Cl_2_(dppm) combined with AgOTs was identified as the first generation catalyst to afford the desired γ-1*(E)*-*alkenyl* (*S*)-γ-butyrolactone (*S*,*E*)-**6a** in a quantitative yield with a *E*/*Z* selectivity of 93:7 and 96% ee in CHCl_3_ at room temperature for 3 h (Table 1, entry 1). For the purpose of improving the *E/Z* selectivity, we tried to identify a more stereoselective catalyst. Based on our recent development of monophosphine ligands^44^, some of the gold complexes of these ligands have been prepared. AuCl(LB-Phos), the structure of which was determined by X-ray single crystal diffraction study (Fig. 3)^45^, is one of them.

### Optimization of reaction conditions

With this rather sterically bulky AuCl(LB-Phos), gladly, an *E*/*Z* selectivity of 97:3 with 100% yield and 97% ee was observed, indicating that the new catalyst was able to control both the C=C stereoselectivity and ensure the efficiency of chirality transfer (Table 1, entry 2). Inspired by this result, we explored the effect of solvents: CH_2_Cl_2_ gave a similar *E*/*Z* and ee (Table 1, entry 3), while 1,2-DCE led to a rather poor *E*/*Z* selectivity of 91:9 (Table 1, entry 4); CH_3_NO_2_ showed a better *E*-selectivity of 98:2, but a much lower ee of 90% (Table 1, entry 6); other solvents such as toluene, dioxane, and CH_3_CN failed to yield any better results even with a prolonged time of 12 h (Table 1, entries 5, 7–8). In the absence of AgOTs, the expected product **6a** was obtained in only 8% yield with 92% recovery of (*R*_a_)-**5a** after 24 h (Table 1, entry 9), and the lactonization couldn’t take place by just using AgOTs (Table 1, entry 10), indicating the significance of the gold-catalysis. Examining the effect of different salts showed the importance of the counter anion: AgPF_6_ resulted in the same *E/Z*-selectivity but with a lower ee of 92% (Table 1, entry 11), while other common silver salts such as AgOTf, AgSbF_6_, and AgOAc, etc. all caused a dropped *E*/*Z* selectivity ranging from 82:18 to 93:7 (Table 1, entries 12–16). Thus, we defined the standard reaction conditions as follows: 5 mol% Au(LB-Phos)Cl/AgOTs in CHCl_3_ at 25 °C for 3 h (Table 1, entry 2).

**Table 1:** Optimization of the reaction conditions for AuCl(LB-Phos)-catalyzed stereoselective cyclization of 4,5-allenoic acid (*R*_a_)-**5a**


Entry	[Ag]	solvent	*t* (h)	6a^a^		ee^b^ of (*S*,*E*)-6a (%)
				Yield (%)	(*S*,*E*)*/*(*R*,*Z*)	
1^c,d^	AgOTs	CHCl_3_	4	100	93:7	96
2^d^	AgOTs	CHCl_3_	3	100	97:3	97
3	AgOTs	CH_2_Cl_2_	3	100	96:4	95
4	AgOTs	1,2-DCE^e^	3	98	91:9	96
5	AgOTs	toluene	12	100	94:6	95
6	AgOTs	CH_3_NO_2_	3	99	98:2	90
7	AgOTs	dioxane	12	100	94:6	96
8	AgOTs	CH_3_CN	12	100	97:3	95
9^f^	–	CHCl_3_	24	8	–	–
10^g^	AgOTs	CHCl_3_	24	0	–	–
11	AgPF_6_	CHCl_3_	3	100	97:3	92
12	AgOMs	CHCl_3_	12	99	93:7	97
13	AgOTf	CHCl_3_	2	100	92:8	–
14	AgSbF_6_	CHCl_3_	2	96	91:9	–
15	AgOAc	CHCl_3_	8.5	100	82:18	–
16	AgBF_4_	CHCl_3_	2	99	92:8	–

AgOTs (0.01 mmol), AuCl(LB-Phos) (0.01 mmol), and solvent (2 mL) were stirred at room temperature for 15 min under nitrogen atmosphere; then 0.2 mmol (*R*_a_)-**5a** and solvent (1 mL) were added

^a^ Determined by ^1^H NMR of the crude product using 1,3,5-trimethylbenzene as internal standard

^b^ Determined by chiral high-performance liquid chromatography (HPLC) analysis

^c^ 2.5 mol% Au_2_Cl_2_(dppm) was used instead of AuCl(LB-Phos)

^d^ The reaction was conducted at 25 °C

^e^ 1,2-DCE: 1,2-dichloroethane

^f^ 92% recovery of (*R*_a_)-**5a**

^g^ The reaction was conducted in the absence of AuCl(LB-Phos); 100% recovery of (*R*_a_)-**5a**

### Substrate scope

With the optimized reaction conditions in hand, differently substituted 4,5-allenoic acids (*R*_a_)-**5** were treated with AuCl(LB-Phos) to afford γ-1*(E)*-*alkenyl* (*S*)-γ-butyrolactones in high yields (93–98%) with an excellent axial-to-center chirality transfer and *E*/*Z*-selectivity (up to >99:1 *E*/*Z*) (Table 2): R could be primary alkyl: *n*-heptyl (**6a**), *n*-butyl (**6b**), *n*-undecyl (**6c**), and phenylethyl (**6g**), or branched alkyl: *i*-Pr (**6d**), and Cy (**6e**). The reaction of benzyl-substituted (*R*_a_)-**5f** should be conducted at −40 °C for 6 h to keep the enantioselectivity due to the observed racemization at 25 °C (**6f**) (compare entry 6 with entry 7 in Table 2). Functional groups including benzyl group, C=C, and C≡C bonds were also tolerated (**6f**, **6g**, **6h**, and **6i**).

**Table 2:** Highly stereoselective synthesis of (*S*, *E*)-**6**


Entry	(*R*_a_)-5		(*S*,*E*)-6		
	R	ee (%)	Yield^a^ (%)	(*S,E*)*/*(*R*,*Z*)^b^	ee^c^ (%)
1	*n*-C_7_H_15_ ((*R*_a_)-**5a**)	96	96 (**6a**)	98:2 (97:3)	96
2	*n*-C_4_H_9_ ((*R*_a_)-**5b**)	97	93 (**6b**)	97:3 (96:4)	96
3^d^	*n*-C_11_H_23_ ((*R*_a_)-**5c**)	97	98 (**6c**)	98:2 (97:3)	96
4	*i*-Pr ((*R*_a_)-**5d**)	98	94 (**6d**)	99:1^e^	98
5	Cy ((*R*_a_)-**5e**)	97	96 (**6e**)	99:1^e^	96
6	Bn ((*R*_a_)-**5f**)	96	90 (**6f**)	95:5 (95:5)	85
7^f^	Bn ((*R*_a_)-**5f**)	96	95 (**6f**)	98:2 (97:3)	97
8	BnCH_2_ ((*R*_a_)-**5g**)	96	97 (**6g**)	97:3 (95:5)	97
9^g^	CH_2_=CH(CH_2_)_8_ ((*R*_a_)-**5h**)	96	94 (**6h**)	>99:1 (97:3)	96
10	TBSC≡C(CH_2_)_6_ ((*R*_a_)-**5i**)	97	96 (**6i**)	97:3 (97:3)	97

AgOTs (0.05 mmol), AuCl(LB-Phos) (0.05 mmol), and CHCl_3_ (5 mL) were stirred at room temperature for 15 min under nitrogen atmosphere; then 1.0 mmol (*R*_a_)-**5** and CHCl_3_ (5 mL) were added at 25 °C

^a^ Isolated yield

^b^ Determined by ^1^H NMR of the isolated product; the data in parentheses were determined by ^1^H NMR of crude product using 1,3,5-trimethylbenzene as internal standard

^c^ Determined by chiral HPLC analysis of isolated (*S*, *E*)-**6**

^d^ Reaction time was 2.5 h

^e^ Not able to be determined in the crude product; determined by quantitative ^13^C NMR of the isolated product

^f^ The reaction was conducted at −40 °C for 6 h

^g^ The reaction time was 2 h

**Fig. 4:**
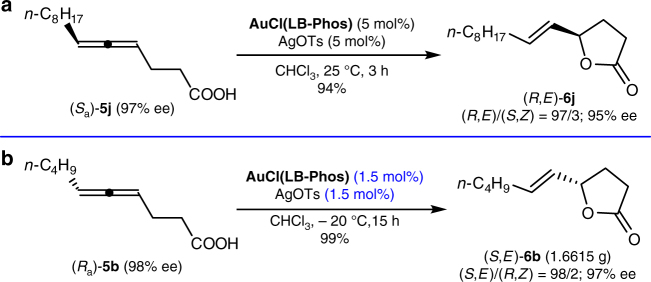
Extended scope of Au-catalyzed cycloisomerization reaction. **a** Synthesis of the enantiomer (*R*,*E*)-butyrolactone **6j**; **b** Gram scale synthesis for γ-butyrolactone (*S*, *E*)-**6b**

As expected, the enantiomer (*R*,*E*)-butyrolactone (**6j**) could also be prepared from the enantiomer of the starting allene, (*S*_a_)-4,5-dienoic acid (*S*_a_)-**5j**, in a high yield and an excellent *E*/*Z*-selectivity (Fig. 4a). After some further optimization (see Supplementary Table 3), a 10 mmol scale reaction with just 1.5 mol% each of AuCl(LB-Phos) and AgOTs in CHCl_3_ at −20 °C for 15 h was realized, delivering an excellent yield of chiral lactone (*S*,*E*)-**6b** with 97% ee and 98:2 *E*/*Z* selectivity (Fig. 4b).

### The effect of different gold catalysts

The results of different gold catalysts combined with AgOTs in CHCl_3_ are listed in Table 3, which showed that AuCl(LB-Phos) was indeed the best catalyst (Table 3, entry 6).

**Table 3:** The effect of different gold catalyst on the stereoselective cyclization of 4,5-allenoic acid (*S*_a_)-**5m**



Entry	[Au]	6m		ee of (*S*,*E*)-6m (%)^b^
		Yield^a^	(*R*,*E*)/(*S*,*Z*)^a^	
1^c^	AuCl	11	–	–
2	AuCl(IPr)	100	95:5	96
3^d^	Au_2_Cl_2_(dppm)	99	93:7	99
4	AuCl(*t*-Bu_3_P)	98	96.5:3.5	97
5	AuCl(Ph_3_P)	98	94:6	99
6	AuCl(LB-Phos)	100	97:3	99
7^e^	AuCl(Zheda-Phos)	99	96:4	99
8^f^	AuCl(Gorlos-Phos)	93	94:6	92

AgOTs (0.01 mmol), [Au] (0.01 mmol), and CHCl_3_ (2 mL) were stirred at room temperature for 15 min under nitrogen atmosphere; then 0.2 mmol of (*S*_a_)-**5m** and CHCl_3_ (1 mL) were added; the reaction mixture was then continuously stirred at 25 °C for 4 h

^a^ Determined by ^1^H NMR of crude product using 1,3,5-trimethylbenzene as internal standard

^b^ Determined by chiral high-performance liquid chromatography (HPLC) analysis

^c^ The reaction was conducted in the absence of AgOTs; 88% of (*S*_a_)-**5m** was recovered

^d^ 2.5 mol% Au_2_Cl_2_(dppm) was used

^e^ Reaction time was 3.75 h

^f^ 2% recovery of (*S*_a_)-**5m**

### Synthesis of racemic xestospongiene

**Fig. 5:**
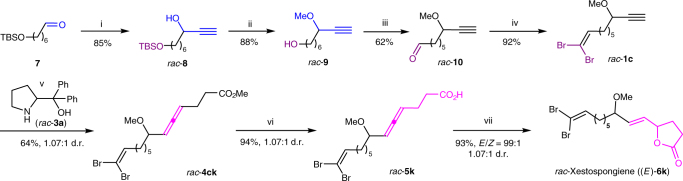
First total synthesis of *rac*-xestospongiene. Reagents and conditions: i. ethynyl-magnesium bromide (1.7 equiv.), THF, rt, 12.5 h; ii. NaH (1.0 equiv.), THF, 0 °C, 0.5 h, then rt, 0.5 h; then MeI (1.2 equiv.), 0 °C, 2 min, then rt, 5 h; then HCl (aq., 3.0 M), 0 °C, then rt, 1.5 h; iii. Fe(NO_3_)_3_∙9H_2_O (8 mol%), TEMPO (8 mol%), NaCl (8 mol%), O_2_ balloon, DCM, 25 °C, 12.5 h; iv. CBr_4_ (1.5 equiv.), PPh_3_ (3.0 equiv.), DCM, 0 °C, 0.5 h, then 0 °C ~ rt, 1 h; v. *rac*-**3a** (1.5 equiv.), methyl 4-oxobutanoate **2k** (1.5 equiv.), CuBr_2_ (20 mol%), dioxane, 130 °C, 17 h; vi. LiOH∙H_2_O (1.5 equiv.), EtOH/H_2_O = 1:1, 90 °C, 1.5 h; vii. AuCl(LB-Phos) (10 mol%), AgOTs (10 mol%), CHCl_3_, -30 °C, 24 h

Such a strategy should deliver a direct entry to the optically active natural γ-butyrolactone with common structure *E*-**I** as shown in Fig. 1a. Xestospongienes are a series of brominated polyunsaturated lipids isolated from the Chinese marine sponge *Xestospongia testudinaria* (shown in Fig. 1a)^4^. No total synthesis has been reported yet. Thus, 7-((*tert*-butyldimethylsilyl)oxy)heptanal **7** underwent 1,2-addition reaction with ethyl magnesium bromide to yield propargylic alcohol **8**. Methylation of **8** via deprotonation with NaH, followed by quenching with MeI, and subsequent removal of the TBS group via acidic hydrolysis yielded the primary alcohol **9** with a terminal C-C triple bond in 88% yield. Fe(III)-catalyzed aerobic oxidation of **9** afforded aldehyde **10** in 62% yield^46^. Wittig olefination of the aldehyde functionality in **10** afforded the terminal alkyne **1c**^47^, which underwent the ATA (allenylation of terminal alkynes) reaction with methyl 4-oxobutanoate **2k** (readily available from γ-butyrolactone in 2 steps^48^) in the presence of diphenylprolinol *rac*-**3a** in a sealed tube at 130 °C^49^ to yield 4,5-allenoate *rac*-**4ck** in 64% yield. Through hydrolysis, allenoic acid *rac*-**5k** was prepared in 96% yield with a d.r. of 1.07:1, which underwent the gold-catalyzed cycloisomerization with 10 mol% catalyst at −30 °C for 24 h (for details, see Supplementary Table 4) to afford *rac*-xestospongiene in an excellent yield and *E*/*Z* ratio of 99:1 (Fig. 5).

### Asymmetric synthesis of xestospongienes E–H

**Fig. 6:**
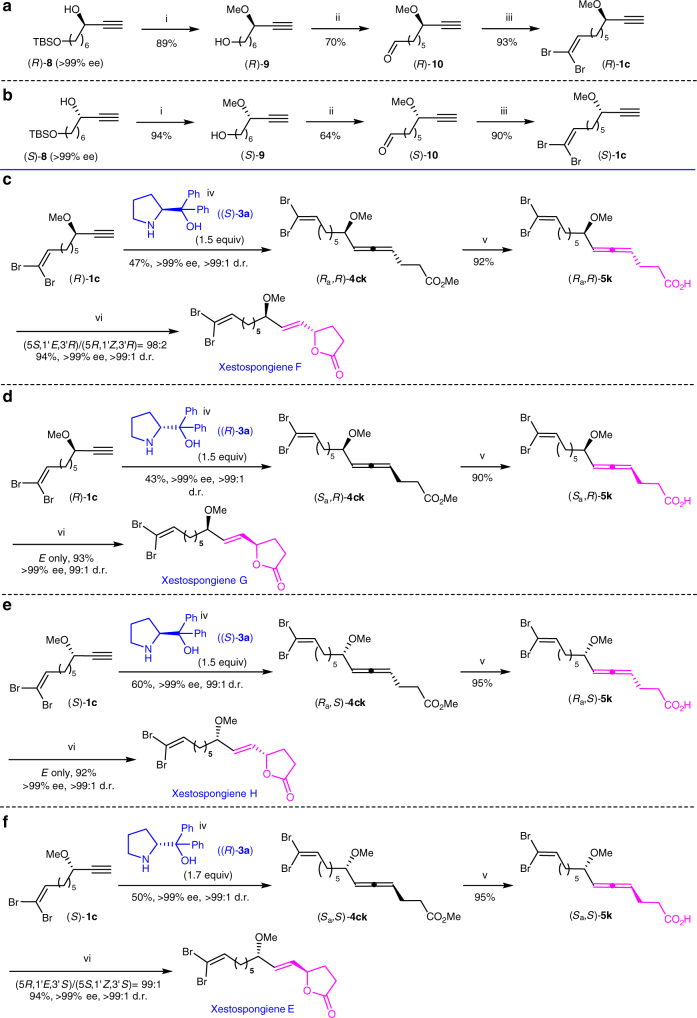
First total synthesis of xestospongienes E–H. **a** Synthesis of (*R*)-**1c**; **b** Synthesis of (*S*)-**1c**; **c** Total synthesis of xestospongiene F; **d** Total synthesis of xestospongiene G; **e** Total synthesis of xestospongiene H; **f** Total synthesis of xestospongiene E. Reagents and conditions: i. NaH (1.0 equiv.), THF, 0 °C then rt; then MeI (1.2 equiv.), 0 °C ~rt; then HCl (aq., 3.0 M), MeOH, rt; ii. Fe(NO_3_)_3_∙9H_2_O (10 mol%), TEMPO (10 mol%), NaCl (10 mol%), O_2_ balloon, DCM, rt; iii. CBr_4_ (1.5 equiv.), PPh_3_ (3.0 equiv.), DCM, 0 °C; iv. methyl 4-oxobutanoate **2k** (1.5–1.7 equiv.), CuBr_2_ (20 mol%), dioxane, 120 °C; v. LiOH∙H_2_O (1.5 equiv.), EtOH/H_2_O = 1:1, 90 °C; vi. AuCl(LB-Phos) (10 mol%), AgOTs (10 mol%), CHCl_3_, −30 °C

It is well known that different stereoisomers of drug molecules may show very distinct biological activities. After methylation, deprotection, aerobic oxidation, and Wittig olefination reaction, (*R*)-**8** and (*S*)-**8** (for their syntheses, see Supplementary Tables 5, 6 and Supplementary Methods^50^) were easily converted to terminal propargylic methyl ethers (*R*)-**1c** and (*S*)-**1c**, respectively (Fig. 6a, b).

The reaction of (*R*)-**1c**, **2k**, and (*S*)-diphenylprolinol (*S*)-**3a** in a ratio of 1:1.5:1.5 afforded (*R*_a_,*R*)-**4ck** as a single stereoisomer in 47% yield with >99% ee and >99:1 d.r. Hydrolysis of (*R*_a_,*R*)-**4ck** was conducted subsequently by its treatment with LiOH∙H_2_O at 90 °C for 1.5 h affording (*R*_a_,*R*)-**5k**, which was cycloisomerized with 10 mol% of AuCl(LB-Phos) at −30 °C to afford (5 *S*,1′*E*,3′*R*)-**6k**, i.e., xestospongiene F (reported as xestospongiene E^4^) in 94% yield with >99% ee and >99:1 d.r (Fig. 6c). Xestospongienes G, H, and (5 *R*,1′*E*,3′*S*)-**6k**, i.e., xestospongiene E (reported as Xestospongiene F^4^) could also be obtained easily with high stereo- and enantioselectivity in a similar way by just replacing amino alcohol (*S*)-**3a** with (*R*)-**3a** or propargylic alcohol (*R*)-**1c** with (*S*)-**1c** (Fig. 6d–f). Subsequently, gram scale synthesis of xestospongiene F was easily realized with a high enantiopurity (99% ee and 98:2 d.r.) (for details, see Supplementary Methods).

### Asymmetric syntheses of naturally occurring γ-alkylic γ-lactones

As stated above, aliphatic γ-butyrolactones are the major aroma components of many industrial fragrances and ingredients in flavors and as food additives^11^, some of which also work as quorum-sensing molecules in vivo^51^. Hydrogenation of the C=C bond in (*E*)-alkenyl γ-butyrolactones *E*-**I** would provide an efficient entry to these naturally occurring γ-alkylic γ-lactones **II** listed in Fig. 1b, provided that the cleavage of the allylic C–O bond causing racemization to the chiral center under the transition metal catalysis may be avoided.

**Fig. 7:**
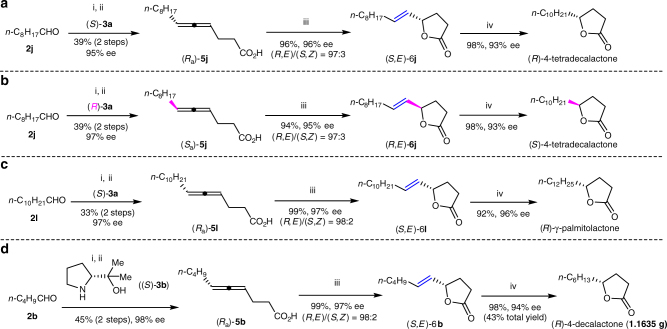
Synthesis of biologically active aliphatic γ-butyrolactones. **a** Synthesis of biologically active (*R*)-4-tetradecalactone; **b** Synthesis of biologically active (*S*)-4-tetradecalactone; **c** Synthesis of biologically active (*R*)-γ-palmitolactone; **d** Synthesis of biologically active (*R*)-4-decalactone. Reagents and conditions: i. ethyl pent-4-ynoate **1b** (1.2 equiv.), **2** (1.5 equiv.), **3** (1.0 equiv.), CuBr_2_ (20 mol%), dioxane, 120 °C; ii. LiOH∙H_2_O (1.5 equiv.), EtOH/H_2_O = 1:1, 90 °C; iii. AuCl(LB-Phos)/AgOTs (1.5–5 mol%), CHCl_3_; iv. Pd/C (2.4–5 mol%), H_2_ (25 atm), EtOAc, rt

It has been established that the two enantiomers of γ-alkylic γ-butyrolactones made some differences in odor quality and odor intensity^11,52,53^. Thus, we tried to synthesize both (*R*) and (*S*)-γ-lactones by utilizing the current strategy. Starting from asymmetric allenylation of readily available ethyl pent-4-ynoate **1b** with nonanal **2j** in the presence of (*S*)-**3a**, followed by hydrolysis, 4,5-allenoic acid (*R*_a_)-**5j** was afforded in 39% yield with 95% ee. Subsequent Au-catalyzed cycloisomerization of (*R*_a_)-**5j** was executed to yield γ-butyrolactone bearing a *trans* C=C bond (*S*,*E*)-**6j** in 96% yield with 96% ee and 97:3 *E*/*Z*. (*R*)-4-tetradecalactone was obtained by hydrogenation of (*S*,*E*)-**6j** in the presence of 5 mol% Pd/C in 98% yield with 93% ee (Fig. 7a)^30,31,54,55^.

The enantiomer (*S*)-4-tetradecalactone was executed by following the same synthetic route just by replacing (*S*)-**3a** with (*R*)-**3a** in the step of EATA (enantioselectivi allenylation of terminal alkynes) reaction, giving the desired product with a similar yield and enantioselectivity (Fig. 7b)^30,55^. Similarly, naturally occurring aromatic (*R*)-γ-palmitolactone was synthesized in 96% ee by just using undecanal **2l** as the starting aldehyde in EATA reaction (Fig. 7c)^56^. (*R*)-4-Decalactone is a sex pheromone of *Osmoderma eremite* released mainly or exclusively by male beetles^57^, which has also proven to be cytotoxic^58,59^, and synthesized with biocatalysts^26,58,59^ or by other strategies^29,53,60^. Gram scale synthesis of (*R*)-4-decalactone was also realized easily in 43% total yield for 4 steps with 94% ee (Fig. 7d).

## Discussion

A facile strategy for general asymmetric synthesis of two types of common γ-butyrolactones from readily available common chemicals-terminal alkynes and aldehydes-has been developed by applying the newly identified catalyst, AuCl(LB-Phos), with the stereoselectivity of up to >99:1 *E*/*Z* and >99% ee. The first total syntheses of xestospongienes E, F, G, and H have been realized with high stereoselectivity. In addition, the C–O bond cleavage-free hydrogenation led to a general access to naturally occurring γ-alkyl γ-butyrolactones, such as (*R*)-4-tetradecalactone, (*S*)-4-tetradecalactone, (*R*)-γ-palmitolactone, and (*R*)-4-decalactone, efficiently with ee of 93–96%. Such a modular solution to two different types of optically active γ-butyrolactones will surely stimulate further interest in the synthetic and bio-potential of these compounds and identifying even better aromas for human life. Further studies on this area are being carried out in our laboratory.

## Methods

### General method for cycloisomerization of alkadienoic acids

To a dry Schlenk tube were added AgOTs (0.0142 g, 0.05 mmol, weighed in glove box, 98%), AuCl(LB-Phos) (0.0299 g, 0.05 mmol), and CHCl_3_ (5 mL) under nitrogen atmosphere sequentially. After stirring for 15 min, (*R*)-trideca-4,5-allenoic acid (*R*_a_)-**5a** (0.2108 g, 1 mmol) and CHCl_3_ (5 mL) were added. After being continuously stirred at 25 °C for 3 h, the reaction was complete as monitored by thin layer chromatography (TLC). Filtration through a short column of silica gel [eluent: Et_2_O (20 mL × 3)] and evaporation afforded a crude mixture of (*S*,*E*)-**6a** and (*R*,*Z*)-**6a** ((*S*,*E*)*/*(*R*,*Z*) = 97:3, as determined by ^1^H NMR analysis). Column chromatography on silica gel afforded (*S*,*E*)-**6a** (0.2017 g, 96%, (*S*,*E*)*/*(*R*,*Z*) = 98:2 as determined by ^1^H NMR analysis) [eluent: petroleum ether (60–90 °C)/ethyl acetate = 15/1 (400 mL) to 10/1 (550 mL)] as an oil with pleasant flavor: 96% ee (HPLC conditions: Chiralcel OJ-H column, *n*-hexane/*i*-PrOH = 200/1, 1.0 mL/min, *λ* = 214 nm, *t*_R_ (major) = 22.73 min, *t*_R_ (minor) = 20.86 min); [*α*]_D_^20^ = + 29.6 (*c* = 1.01, CHCl_3_); ^1^H NMR (300 MHz, CDCl_3_) *δ* 5.81 (dt, *J*_1_ = 15.3 Hz, *J*_2_ = 7.2 Hz, 1H, =CH), 5.49 (dd, *J*_1_ = 15.3 Hz, *J*_2_ = 7.2 Hz, 1H, =CH), 4.90 (q, *J* = 7.2 Hz, 1H, CH), 2.61–2.50 (m, 2H, CH_2_), 2.46–2.31 (m, 1H, one proton from CH_2_), 2.13–1.90 (m, 3H, CH_2+_ one proton from CH_2_), 1.45–1.18 (m, 10H, CH_2_ × 5), 0.88 (t, *J* = 6.6 Hz, 3H, CH_3_); the following signals are discernible for (*R*,*Z*)-**6a**: *δ* 5.72–5.62 (m, 1H, =CH), 5.31–5.21 (m, 1H, CH); ^13^C NMR (75 MHz, CDCl_3_) *δ* 177.0, 135.6, 127.2, 81.1, 32.0, 31.6, 29.0, 28.9, 28.7, 28.64, 28.61, 22.5, 14.0; IR (neat) *ν* (cm^−1^) 2955, 2926, 2855, 1778, 1673, 1459, 1415, 1378, 1327, 1296, 1216, 1177, 1123, 1010; GC-MS (GC condition: injector: 280 °C; column: DB5 column 30 m × 0.25 mm, temperature programming: 60 °C (2 min), 20 °C/min to 280 °C, 280 °C (30 min); detector: 280 °C) (70 ev, EI) *m/z* (%) for (*S*,*E*)-**6a**: *t*_R_ (major) = 5.83 min: 210 (M^+^, 2.31), 111 (100); for (*R*,*Z*)-**6a**: *t*_R_ (minor) = 5.76 min: 210 (M^+^, 0.75), 111 (100). HRMS calcd for C_13_H_22_O_2_ [M^+^]: 210.1620, found: 210.1624.

### Data availability

All data that support the findings of this study are available in the online version of this paper in the accompanying Supplementary Methods (including experimental procedures, compound characterization data).

The X-ray crystallographic coordinates for structure of AuCl(LB-Phos) reported in this study has been deposited at the Cambridge Crystallographic Data Centre (CCDC), under deposition number CCDC 1558142. This data can be obtained free of charge from The Cambridge Crystallographic Data Centre via www.ccdc.cam.ac.uk/data_request/cif.

## Electronic supplementary material

Supplementary Information
