# Supplementary material for: Gold-catalyzed stereoselective cycloisomerization of allenoic acids for two types of common natural γ-butyrolactones
Source: Nat Commun. 2018 Apr 25;9:1654. doi: 10.1038/s41467-018-03894-6 (PMC5916948; doi:10.1038/s41467-018-03894-6)
Supplement: Supplementary file 1 — Supplementary Information [file 41467_2018_3894_MOESM1_ESM.pdf]

Supplementary Information for

Gold-catalyzed Stereoselective Cycloisomerization of Allenoic Acids for Two Types of Common Natural γ-Butyrolactones

Zhou et al.

Supplementary Methods

Materials:

^1H and ^{13}C nuclear magnetic resonance spectra were recorded with an instrument operated at 300 MHz for ^1H NMR spectra and 75 MHz for ^{13}C NMR spectra. $CDCl_3$ was used as solvent in all NMR experiments. Chemical shifts (δ) are given in parts per million (ppm). Infrared spectra were recorded from the films of pure samples on sodium chloride plates on a FT-IR spectrometer. Mass and HRMS spectra were carried out in EI or ESI mode. Flash column chromatography was performed on silica gel H. Dioxane and THF were refluxed over sodium wire using diphenyl ketone as indicator and distilled right before use. $CuBr_2$ was purchased from *J* & *K*. (*S*)-α,α-diphenylprolinol and (*R*)-α,α-diphenylprolinol were purchased from Shanghai Darui Fine Chemicals. Aldehydes were distilled right before use. AgOTs was purchased from Alfa Aesar. CBr_4 used was recrystallized from EtOH, washed with ethyl ether, and dired by vacuum. Other commercially available reagents were purchased and used as received.

Synthesis of enantioenriched 4-allenoic acids.

Enantioselective allenylation of terminal alkynes **1a** with aldehyde **2a** in the presence of amine (*S*)-**3a** (EATA) afforded the desired product (R_a)-**4aa** in 36% yield with 95% ee in a sealed tube at 130 °C for 15 h (Supplementary Table 1, entry 1).[1] With 1.1 equiv. of benzyl pent-4-ynoate **1a**, the yield of (R_a)-**4aa** was improved to 38% albeit with 94% ee (Supplementary Table 1, entry 2). By replacing benzyl pent-4-ynoate **1a** with ethyl pent-4-ynoate **1b**, the yield of 4,5-dienoate (R_a)-**4ba** was improved to 53% but ee dropped to 85% (Supplementary Table 1, entry 3). We then screened the effect of the loading of **1b**, showing that 1.2 equiv. are the best (Supplementary Table 1, entries 4 and 5). The yield for the reaction at 120 °C in the sealed tube with 1.2 equiv. **1b** is poor (Supplementary Table 1, entry 6); the reaction under reflux at 120 °C afforded (R_a)-**4ba** in 46% yield with 96% ee (Supplementary Table 1, entries 7-11). Thus, the optimal reaction conditions were established as follows: 20 mol% $CuBr_2$, alkyne **1b**/aldehyde **2a**/amine (*S*)-**3a** = 1.2/1.5/1 in dioxane

under reflux (Supplementary Table 1, entry 7).

Supplementary Table 1 Synthesis of 4,5-allenoatea (*R*a)-4aa and (*R*a)-4ba [a]

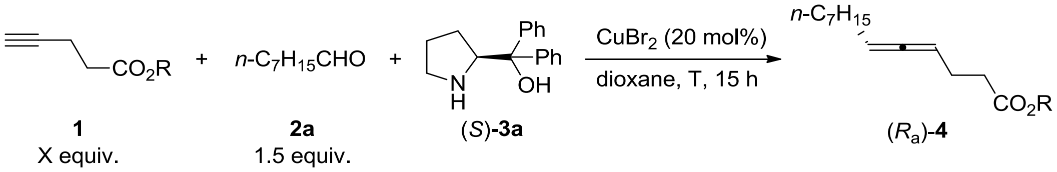

entry	**1**		T/°C	(R_a)-**4**	
	R	X (equiv.)		yield [b] (%)	ee [c] (%)
1	Bn (**1a**)	1.5	130 [d]	36 ((R_a)-**4aa**)	95
2	Bn (**1a**)	1.1	130 [d]	38 ((R_a)-**4aa**)	94
3	Et (**1b**)	1.1	130 [d]	53 ((R_a)-**4ba**)	85
4	Et (**1b**)	1.2	130 [d]	60 ((R_a)-**4ba**)	93
5	Et (**1b**)	1.4	130 [d]	63 ((R_a)-**4ba**)	91
6	Et (**1b**)	1.2	120 [d]	49 ((R_a)-**4ba**)	93
**7**	**Et (1b)**	**1.2**	**120** [e]	**46 ((R_a)-4ba)**	**96**
8	Et (**1b**)	1.1	120 [e]	37 ((R_a)-**4ba**)	95
9	Et (**1b**)	1.3	120 [e]	44 ((R_a)-**4ba**)	94
10	Et (**1b**)	1.4	120 [e]	47 ((R_a)-**4ba**)	96
11	Et (**1b**)	1.5	120 [e]	45 ((R_a)-**4ba**)	93

[a] The reactions were conducted on a 1.0 mmol of (*S*)-**3a** in 3 mL of dioxane with 1.5 equiv. **2a** under nitrogen atmosphere. [b] Isolated yield. [c] Determined by chiral HPLC analysis of isolated product. [d] Reactions were conducted in a sealed tube. [e] Reactions were conducted under reflux under N_2 atmosphere.

Then a series of 4-allenoic acids (R_a)-**5** were prepared by treating the corresponding carboxylates (R_a)-**4** with 1.5 equiv. of $LiOH{\cdot}H_2O$ in EtOH/H_2O under reflux at 90 °C (Supplementary Table 2).

Supplementary Table 2 Synthesis of 4-allenoic acids (R_a)-5 [a]

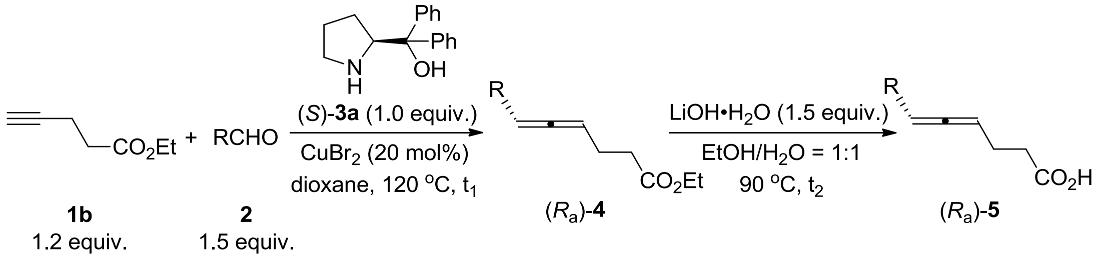

Entry	2	t_1	t_2 (h)	(R_a)-**5**	
	R	(h)		yield [b] (%)	ee [c] (%)
1	*n*-C_7H_{15} (**2a**)	15	8	40 ((R_a)-**5a**)	96
2 [d]	*n*-C_4H_9 (**2b**)	18	8	38 ((R_a)-**5b**)	97
3	*n*-$C_{11}H_{23}$ (**2c**)	17	19	46 ((R_a)-**5c**)	97
4 [e]	*i*-Pr (**2d**)	15.5	12	24 ((R_a)-**5d**)	98
5	Cy (**2e**)	15	18	34 ((R_a)-**5e**)	97
6	Bn (**2f**)	17	12	34 ((R_a)-**5f**)	96
7	$BnCH_2$ (**2g**)	17.5	18	45 ((R_a)-**5g**)	96
8	$CH_2=CH(CH_2)_8$ (**2h**)	19	18	40 ((R_a)-**5h**)	96
9 [f]	$TBSCC(CH_2)_6$ (**2i**)	18	5	42 ((R_a)-**5i**)	97

[a] The reactions were conducted on a 15 mmol scale of (*S*)-**3a** with 1.2 equiv. **1b** and 1.5 equiv. **2** under nitrogen atmosphere. [b] Combined yield of 2 steps. [c] Determined by chiral HPLC analysis of the corresponding carboxylate of (R_a)-**5**. [d] The reaction was conducted on a 25 mmol scale of (*S*)-**3a**. [e] The reaction was conducted on a 20 mmol scale of (*S*)-**3a**. [f] The reaction was conducted on a 7 mmol scale; Hydrolysis under a mild condition: 1.0 equiv. of LiOH·H_2O in THF/MeOH/H_2O = 1:1:1 at room temperature for 5 h.

Optimization of the lactonization reaction for gram scale synthesis

For gram scale synthesis of (*E*)-**6**, further adjustments for the temperature of the lactonization reaction were conducted to improve the *E*/*Z* selectivity: runing the reaction at 0 °C made no difference as compared with tht at 25 °C (Supplementary Table 3, entries 1-2), while the reaction at -20 °C gave a higher selectivity (*E*/*Z* = 98:2) (Supplementary Table 3, entry 3); when the reaction was conducted at -40 °C, a result

of 99:1 *E/Z* selectivity in 44% yield with 56% recovery of acid **5b** was observed (Supplementary Table 3, entry 4). Thus, we defined the reaction temperature as -20 °C.

Supplementary Table 3 Further optimization of the reaction conditions for racemic (*E*)-6b[a]

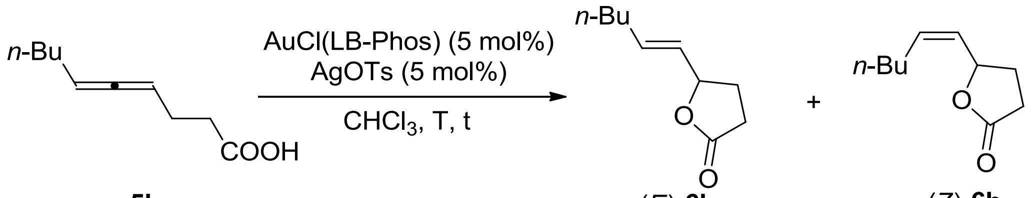

Entry	T (°C)	t (h)	6b NMR yield[b] (%)	6b *E*/*Z*[b]
1	25	3	100	96/4
2	0	12	100	97/3
**3**	**-20**	**12**	**100**	**98/2**
4[c]	- 40	17	44	99/1

[a] AgOTs, AuCl(LB-Phos), and $CHCl_3$ (2 mL) were stirred for 15 min under nitrogen atmosphere; then 0.2 mmol **5b** and $CHCl_3$ (1 mL) were added. [b] Determined by ^{1}H NMR of crude product using 1,3,5-trimethylbenzene as internal standard. [c] 56% recovery of **5b** was detected.

Optimization of the the synthesis of *rac*-xestospongiene

Under the optimized conditions, the Au-catalyzed lactonization reaction of *rac*-**5k** was executed to afford racemic xestospongiene (**6k**) with an *E*/*Z* selectivity of 94:6 (Supplementary Table 4, entry 1). The reaction at -30 °C for 31 h led to an *E*/*Z* selectivity of 97:3 but with 11% *rac*-**5k** being recovered (Supplementary Table 4, entry 2); further lowering the temperature to -40 °C couldn't make a better selectivity while more starting acid *rac*-**5k** was recovered even after 36 h (Supplementary Table 4, entry 3). Then, we tried to increase the loading of the catalysts. When the reaction at -20 °C with 10 mol% catalysts, **6k** was formed in 99% NMR yield with a *E*/*Z*

selectivity of 96:4 after 20 h (Supplementary Table 4, entry 4); Reducing the temperature to -30 ^{o}C with 10 mol% catalysts gave **6k** a higher 97:3 selectivity with no *rac*-**5k** recovered after 24 h (Supplementary Table 4, entry 5). Thus, we defined 10 mol% catalyst at -30 ^{o}C for 24 h as the standard reaction conditions.

Supplementary Table 4 Optimization of the reaction conditions for the cyclization of *rac*-5k [a]

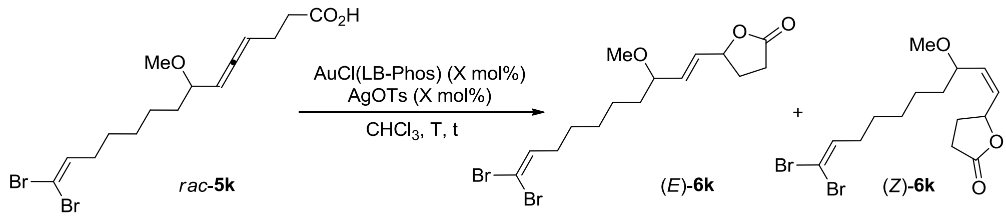

Entry	X mol%	T (^{o}C)	t (h)	6k		*rac*-5k
				NMR yield [b] (%)	*E*/*Z* [b]	recovery [b] (%)
1 [c]	5	22	2.5	100	94:6	0
2	5	-30	31	88	97:3	11
3	5	-40	36	90	97:3	10
4 [d]	10	-20	20	99	96:4	0
**5**	**10**	**-30**	**24**	**97**	**97:3**	**0**

[a] AgOTs, AuCl(LB-Phos), and $CHCl_3$ (2 mL) were stirred for 15 min under nitrogen atmosphere; then 0.2 mmol *rac*-**5k** and $CHCl_3$ (1 mL) were added. [b] Determined by ^{1}H NMR of the crude product using 1,3,5-trimethylbenzene as internal standard. [c] The reaction was conducted on a 1.0 mmol scale of *rac*-**5k** in 10 mL of $CHCl_3$ under N_2 atomosphere. [d] The reaction was conducted on a 0.1 mmol scale of *rac*-**5k** in 1.5 mL of $CHCl_3$ under N_2 atomosphere.

Kinetic resolution of racemic 8: synthesis of optically active propargylic alcohols (*R*)-8 and (*S*)-8

A novozym-435-catalyzed kinetic resolution of 9-((*tert*-butyldimethylsilyl)oxy)non-1-yn-3-ol **8** with vinyl acetate was studied and some typical results are presented in Supplementary Table 5. We ran the reaction under the reported conditions initially:[2] The treatment of racemic propargylic alcohol

8 with 10 *w*% novozym-435-catalyzed in 3 mL of vinyl acetate at 60 ^{o}C for 2.75 h afforded propargylic acetate (*S*)-**11** in 33% NMR yield with 93 % ee and 66% NMR yield of propargylic alcohol (*R*)-**8** (Supplementary Table 5, entry 1); shorten the time to 1.5 h led to a slightly higher ee of 94% for (*S*)-**11** but a lower 21% yield (Supplementary Table 5, entry 2); prolonging the time to the point when acetate (*S*)-**11** and alcohol (*R*)-**8** were approximately equal in yield didn't give a better ee (Supplementary Table 5, entry 3); further extending the time to 19.5 h made the yield of (*S*)-**11** 55% with 81 % ee and 45% of alcohol (*R*)-**8** recovered in 79% ee (Supplementary Table 5, entry 4). Then the loadings of enzyme and vinyl acetate were also screened (Supplementary Table 5, entries 5-7). The ee of (*S*)-**11** was improved to 95% with an increased yield of 37% when temperature went down to 30 ^{o}C (Supplementary Table 5, entry 8). 20% *w* of the catalyst used for the reaction at this temperature for 6 h led to a better result of 41% yield of acetate (*S*)-**11** with 99% ee (Supplementary Table 5, entry 9).

Supplementary Table 5 Novozym-435-catalyzed kinetic resolution of racemic 8 [a].

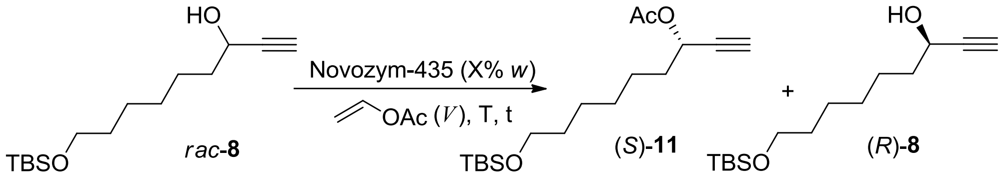

Entry	T (°C)	X (% *w*)[b]	*V* (mL)	t (h)	(*S*)-**11**		(*R*)-**8**	
					yield[c] (%)	ee[d] (%)	yield[c] (%)	ee[e] (%)
1	60	10	3	2.75	33	93	66	-
2	60	10	3	1.5	21	94	77	-
3	60	10	3	11	51	86	49	86
4	60	10	3	19.5	55	81	45	79
5	60	20	3	2.75	41	89	51	-
6	60	20	6	2.75	30	91	64	-
7	60	20	6	1.75	28	93	71	-
8	30	10	3	9	37	95	60	-
**9**	**30**	**20**	**3**	**6**	**41**	**99**[f]	**59**	**-**

[a] Reactions were conducted on a 0.5 mmol scale of *rac*-**8**. [b] % by weight. [c] Determined by ^{1}H NMR of the crude product using 1,3,5-trimethylbenzene as internal standard. [d] Determined by chiral HPLC analysis of isolated acetate (*S*)-**11**. [e] Determined by chiral HPLC analysis of acetate of isolated alcohol (*R*)-**8**. [f] Determined by chiral GC analysis of isolated acetate (*S*)-**11**.

On the basis of these observations, we set out to explore how to get higher ees of (*S*)-**11** and alcohol (*R*)-**8** at the same time. For this purpose, we identified *n*-hexane as the solvent with the addition of different amounts of vinyl acetate for the reaction at 30 °C. Fortunately, when 20 *w*% of enzyme was used with 5.2 equiv. of vinyl acetate in *n*-hexane for 11.5 h, ee of (*R*)-**8** reached 97% in 44% yield along with 93% ee of (*S*)-**11** in 46% yield (Supplementary Table 6, entry 1). Reducing the reaction time to 2.5 h failed to give a better ee of (*S*)-**11** (Supplementary Table 6, entry 2) (compare with the best result showed in Supplementary Table 5); we then prolonged the reaction time to 19 h, affording (*R*)-**8** with 98% ee and 47% yield (Supplementary Table 6, entry 3).

Supplementary Table 6 Further optimization of Novozym-435-catalyzed kinetic resolution of 8 [a]

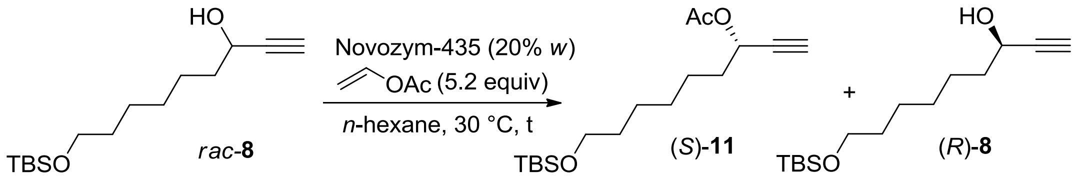

Entry	T (h)	(*S*)-**11**		(*R*)-**8**	
		yield [b] (%)	ee [c] (%)	yield [b] (%)	ee [d] (%)
1	11.5	46	93	44	97
2	2.5	35	97	44	-
**3**	**19**	**50**	**92**	**47**	**98**

[a] Reactions were conducted on a 0.5 mmol scale of *rac*-**8** at 30 °C. [b] Determined by ^{1}H NMR of the crude product using 1,3,5-trimethylbenzene as internal standard. [c] Determined by chiral HPLC analysis of isolated acetate (*S*)-**11.** [d] Determined by chiral HPLC analysis of acetate of isolated alcohol (*R*)-**8.**

Synthesis of enantioenriched 4-allenoic acids

1. Synthesis of (R_a)-benzyl 4,5-tridecadienoate (R_a)-**4aa** (zj-8-078)

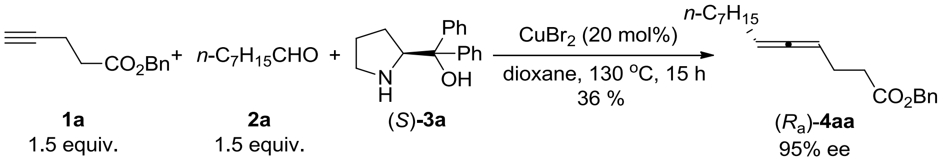

To a flame-dried Schlenk tube with a polytetrafluoroethylene plug were added $CuBr_2$ (0.0462 g, 0.2 mmol, 98%), (*S*)-**3a** (0.2582 g, 1 mmol，98%), **1a** (0.2824 g, 1.5 mmol)/dioxane (2 mL), and octaldehyde **2a** (0.1927 g, 1.5 mmol)/dioxane (1 mL) sequentially under nitrogen atmosphere. The Schlenk tube was then sealed with screwing the polytetrafluoroethylene plug tightly. The reaction was complete after being stirred in an oil bath preheated at 130 °C for 15 h as monitored by TLC. The mixture was filtrated through a short column of silica gel [eluent: Et_2O (10 mL × 3)] and then washed with an aqueous solution of hydrochloric acid (3 M, 10 mL × 3). The organic layer was separated and the aqueous layer was extracted with Et_2O (10 mL).

The combined organic layer was washed with brine and dried over anhydrous Na_2SO_4. After filtration and evaporation, the residue was purified by chromatography [eluent: petroleum ether (60-90 °C)/ diethyl ether = 100/1 (600 mL)] on silica gel to afford (R_a)-**4aa** (0.1089 g, 36%) as a liquid: 95% ee (HPLC conditions: Chiralcel OJ-H column, *n*-hexane/*i*-PrOH = 200/1, 0.8 mL/min, λ = 214 nm, t_R (major) = 9.6 min, t_R (minor) = 8.5 min); $[\alpha]_D^{20}$ = -54.5 (c = 1.01, $CHCl_3$); ^1H NMR (300 MHz, $CDCl_3$) δ 7.45-7.25 (m, 5H, Ar-H), 5.19-5.04 (m, 4H, =CH × 2 + CH_2), 2.53-2.41 (m, 2H, CH_2), 2.37-2.25 (m, 2H, CH_2), 2.00-1.87 (m, 2H, CH_2), 1.43-1.18 (m, 10H, CH_2 × 5), 0.87 (t, J = 6.8 Hz, 3H, CH_3); ^{13}C NMR (75 MHz, $CDCl_3$) δ 203.6, 172.8, 135.9, 128.4, 128.1, 128.0, 92.5, 89.3, 60.0, 33.3, 31.7, 29.03, 28.99, 28.7, 23.7, 22.5, 14.0; IR (neat) ν (cm^{-1}) 3093, 3066, 3034, 2955, 2926, 2855, 1962, 1740, 1498, 1456, 1380, 1353, 1256, 1154, 1002; MS (70 ev, EI) *m/z* (%) 301 (M^+ + 1, 2.36), 300 (M^+, 2.56), 167 (100), 91 (100); HRMS calcd for $C_{20}H_{28}O_2$ [M^+]: 300.2089, found: 300.2090.

2. Synthesis of (R_a)-4,5-tridecadienoic acid (R_a)-**5a** (zj-8-039, 8-040)

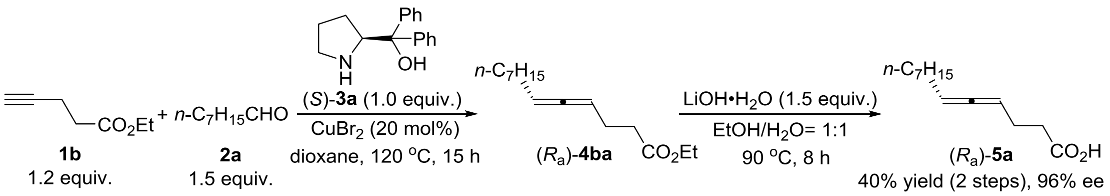

Typical Procedure I: To a dry Schlenk flask were added $CuBr_2$ (0.6768 g, 3 mmol, 99%), (S)-2-(diphenylhydroxymethyl)pyrrolidine (S)-**3a** (3.8707 g, 15 mmol, 98%), ethyl pent-4-ynoate **1b**[3] (2.2715 g, 18 mmol)/dioxane (20 mL), and octaldehyde **2a** (3.5 mL, d = 0.821 g cm^{-3}, 2.8739 g, 22.45 mmol)/dioxane (10 mL) sequentially under nitrogen atmosphere. After continuous stirring for 15 h under reflux at 120 °C, the reaction was complete as monitored by TLC. Then the resulting mixture was cooled down to room temperature. The mixture was diluted with ether (60 mL) and then washed with an aqueous solution of hydrochloric acid (3 M, 10 mL × 3). The organic layer was separated and the aqueous layer was extracted with Et_2O (10 mL). The combined organic layer was washed with brine and dried over anhydrous Na_2SO_4. After filtration and evaporation, the residue was purified by

chromatography [eluent: petroleum ether (60-90 °C)/diethyl ether = 100/1 (500 mL × 3) to 50:1 (300 mL)] on silica gel to afford (R_a)-**4ba** (1.4870 g) as a liquid, which was then used in the next step without further characterization.

Typical Procedure II: To a round-bottom flask were added (R_a)-**4ba** (1.4870 g, 6.25 mmol, prapared above), EtOH/H_2O = 1:1 by volume (pre-mixed by using 31 mL of H_2O and 31 mL of EtOH), and LiOH·H_2O (0.4147 g, 9.375 mmol, 95%) sequentially. After continuous stirring for 8 h under reflux at 90 °C, the reaction was complete as monitored by TLC. Then the mixture was cooled down to room temperature. After evaporation to remove EtOH, the resulting mixture was acidified with an aqueous solution of hydrochloric acid (aq., 3.0 M) until pH = 1 and extracted with Et_2O (20 mL × 3). The combined organic layer was washed with brine and dried over anhydrous Na_2SO_4, filtration, evaporation, and column chromatography on silica gel gave (R_a)-**5a**[4] (1.2547 g, 40%, 2 steps) [eluent: petroleum ether (60-90 °C)/ ethyl acetate = 15/1 (480 mL) to 10:1 (600 mL) to 5:1 (300 mL)] as an oil: 96% ee (determined by the corrsponding esterification product (*R*)-ethyl trideca-4,5-dienoate (R_a)-**4ba**); $[\alpha]_D^{20}$ = -70.1 (c = 1.00, $CHCl_3$); ^{1}H NMR (300 MHz, $CDCl_3$) δ 11.21 (bs, 1H, COOH), 5.27-5.04 (m, 2H, =CH × 2), 2.48 (t, *J* = 7.5 Hz, 2H, CH_2), 2.36-2.23 (m, 2H, CH_2), 2.03-1.90 (m, 2H, CH_2), 1.45-1.18 (m, 10H, CH_2 × 5), 0.88 (t, *J* = 6.8 Hz, 3H, CH_3); ^{13}C NMR (75 MHz, $CDCl_3$) δ 203.7, 179.7, 92.9, 89.2, 33.1, 31.8, 29.1, 28.8, 23.5, 22.6, 14.1; IR (neat) ν (cm^{-1}) 3582-2257 (COOH), 3031, 2957, 2926, 2855, 2662, 1963, 1713, 1436, 1412, 1378, 1335, 1277, 1249, 1211, 1173, 1077; MS (70 ev, EI) *m/z* (%) 210 (M^+, 4.49), 126 (100).

Esterification for determination of the ee value of (R_a)-**5a**: synthesis of (R_a)-ethyl 4,5-tridecadienoate (R_a)-**4ba**[5] (zj-8-043)

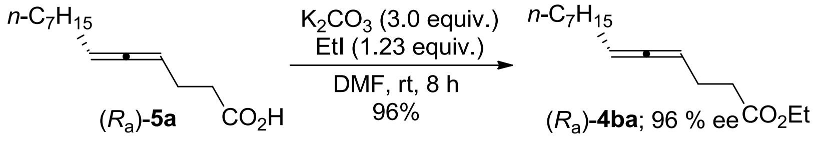

Typical Procedure III: To a Schlenk tube were added (R_a)-**5a** (0.0846 g, 0.4 mmol, prapared above) and DMF (4 mL). Then K_2CO_3 (0.1657 g, 1.2 mmol) and EtI

(0.04 mL, d = 1.94 g cm^{-3}, 0.0768 g, 0.49 mmol, 99%) were added sequentially. After continuous stirring at room temperature for 8 h, the reaction was complete as monitored by TLC. After quenching with water (10 mL) and being stirred for 10 min, the aqueous solution was extracted with Et_2O (15 mL × 3). The combined organic layer was washed with H_2O then brine and dried over anhydrous Na_2SO_4. Filtration, evaporation, and column chromatography on silica gel gave (R_a)-**4ba** (0.0917 g, 96%) [eluent: petroleum ether (60-90 °C)/ ethyl acetate = 50/1 (450 mL)] as a liquid: 96% ee (HPLC conditions: Chiralcel OD-H column, *n*-hexane, 0.5 mL/min, λ = 214 nm, t_R (major) = 15.8 min, t_R (minor) = 15.4 min); $[\alpha]_D^{20}$ = -61.9 (c = 0.99, $CHCl_3$); ^{1}H NMR (300 MHz, $CDCl_3$) δ 5.30-4.95 (m, 2H, =CH × 2), 4.13 (q, *J* = 7.1 Hz, 2H, OCH_2), 2.45-2.34 (m, 2H, CH_2), 2.34-2.23 (m, 2H, CH_2), 2.02-1.88 (m, 2H, CH_2), 1.44-1.18 (m, 13H, CH_2 × 5 + CH_3), 0.88 (t, *J* = 6.6 Hz, 3H, CH_3); ^{13}C NMR (75 MHz, $CDCl_3$) δ 203.6, 173.0, 92.4, 89.4, 60.1, 33.3, 31.7, 29.03, 28.99, 28.8, 23.8, 22.5, 14.1, 13.9; IR (neat) ν (cm^{-1}) 2958, 2926, 2855, 1962, 1739, 1464, 1446, 1421, 1372, 1349, 1300, 1259, 1195, 1158, 1133, 1096, 1077, 1039; MS (70 ev, EI) *m/z* (%) 238 (M^+, 5.49), 80 (100); HRMS calcd for $C_{15}H_{26}O_2$ [M^+]: 238.1933, found: 238.1933.

3. Synthesis of (R_a)-4,5-decadienoic acid (R_a)-**5b** (zj-6-047, 6-053)

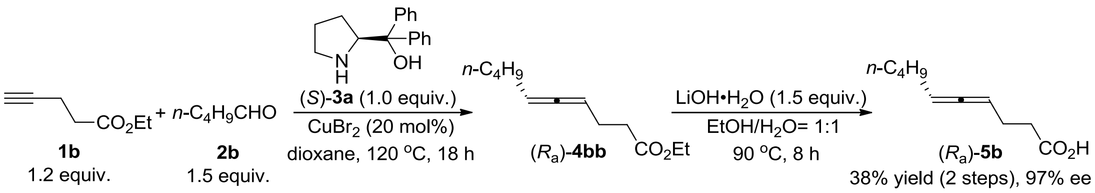

Typical Procedure IV: To a dry Schlenk flask were added $CuBr_2$ (1.1261 g, 5.0 mmol, 99%), (*S*)-**3a** (6.4555 g, 25 mmol, 98%), **1b** (3.7875 g, 30 mmol)/dioxane (30 mL), and valeraldehyde **2b** (4.0 mL, d = 0.81 g cm^{-3}, 3.24 g, 37.67 mmol)/dioxane (15 mL) sequentially under nitrogen atmosphere. After continuous stirring for 18 h under reflux at 120 °C, the reaction was complete as monitored by TLC. Then the resulting mixture was cooled down to room temperature. The mixture was filtrated through a short column of silica gel [eluent: Et_2O (25 mL × 3)], and then washed with an aqueous solution of hydrochloric acid (3 M, 10 mL × 3). The organic layer was

separated and the aqueous layer was extracted with Et_2O (10 mL). The combined organic layer was washed with brine and dried over anhydrous Na_2SO_4. After filtration and evaporation, the residue was purified by chromatography [(eluent: petroleum ether (60-90 ^oC)/diethyl ether = 100/1 (500 mL × 2) to 50/1 (500 mL × 2)] on silica gel to afford (R_a)-**4bb** (2.0071 g) as a liquid, which was then used in the next step without further characterization.

Following **Typical Procedure II**, the reaction of (R_a)-**4bb** (2.0071 g, 10.2 mmol, prepared above), EtOH/H_2O = 1:1 by volume (pre-mixed by using 50 mL of H_2O and 50 mL of EtOH), and LiOH·H_2O (0.6768 g, 15.3 mmol, 95%) at 90 ^oC for 8 h afforded (R_a)-**5b** (1.6152 g, 38%, 2 steps) [(eluent: petroleum ether (60-90 ^oC)/ethyl acetate = 10/1 (550 mL) to 8/1 (540 mL) to 5/1 (600 mL)] as an oil: 97% ee (determined by the corrsponding esterification product (R_a)-**4bb**); $[\alpha]_D^{20}$ = -77.3 (c = 0.995, $CHCl_3$); ^1H NMR (300 MHz, $CDCl_3$) δ 11.42 (bs, 1H, COOH), 5.26-5.08 (m, 2H, =CH × 2), 2.48 (t, J = 7.4 Hz, 2H, CH_2), 2.36-2.23 (m, 2H, CH_2), 2.06-1.90 (m, 2H, CH_2), 1.45-1.24 (m, 4H, CH_2 × 2), 0.90 (t, J = 7.2 Hz, 3H, CH_3); ^{13}C NMR (75 MHz, $CDCl_3$) δ 203.7, 179.9, 92.8, 89.2, 33.1, 31.2, 28.5, 23.5, 22.1, 13.8; IR (neat) ν (cm^{-1}) 3699-2161 (COOH), 3031, 2958, 2928, 2871, 2863, 2668, 1963, 1711, 1436, 1412, 1377, 1333, 1274, 1247, 1211, 1173; MS (70 ev, EI) m/z (%) 169 (M^+ + 1, 2.39), 168 (M^+, 5.55), 126 (100); HRMS calcd for $C_{10}H_{16}O_2$ [M^+]: 168.1150, found: 168.1148.

Esterification for determination of the ee value of (R_a)-**5b**: synthesis of (R_a)-ethyl 4,5-decadienoate (R_a)-**4bb** (zj-6-055)

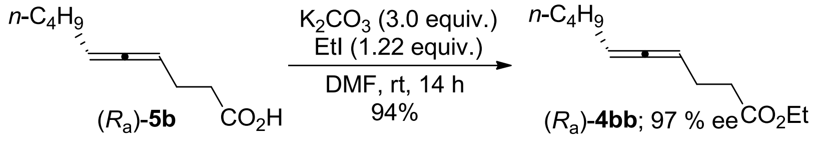

Following **Typical Procedure III**, the reaction of (R_a)-**5b** (0.0839 g, 0.5 mmol)/DMF (5 mL), EtI (0.05 mL, d = 1.94 g cm^{-3}, 0.0951 g, 0.61 mmol, 98%), and K_2CO_3 (0.2072 g, 1.5 mmol) for 14 h afforded (R_a)-**4bb** (0.0924 g, 94%) [eluent: petroleum ether (60-90 ^oC)/ethyl acetate = 100/1 (500 mL) to 50/1 (300 mL)] as a

liquid: 97% ee (HPLC conditions: Chiralcel PC-2 column, *n*-hexane, 0.7 mL/min, λ = 214 nm, t_R (major) = 44.8 min, t_R (minor) = 38.4 min); $[\alpha]_D^{20}$ = -68.0 (c = 0.99, $CHCl_3$); ^{1}H NMR (300 MHz, $CDCl_3$) δ 5.18-5.07 (m, 2H, =CH × 2), 4.13 (q, *J* = 7.1 Hz, 2H, OCH_2), 2.47-2.36 (m, 2H, CH_2), 2.35-2.23 (m, 2H, CH_2), 2.06-1.91 (m, 2H, CH_2), 1.44-1.28 (m, 4H, CH_2 × 2), 1.26 (t, *J* = 7.2 Hz, 3H, CH_3) 0.90 (t, *J* = 7.1 Hz, 3H, CH_3); ^{13}C NMR (75 MHz, $CDCl_3$) δ 203.7, 173.0, 92.3, 89.4, 60.1, 33.4, 31.2, 28.5, 23.8, 22.1, 14.1, 13.7; IR (neat) ν (cm^{-1}) 2959, 2929, 2875, 2859, 1963, 1738, 1466, 1444, 1418, 1372, 1350, 1300, 1250, 1159, 1097, 1052, 1033; MS (70 ev, EI) m/z (%) 197 (M^+ + 1, 10.80), 196 (M^+, 24.87), 81 (100); HRMS calcd for $C_{12}H_{20}O_2$ [M^+]: 196.1463, found: 196.1461.

4. Synthesis of (*R*a)-4,5-heptadecadienoic acid (*R*a)-**5c** (zj-4-028, 4-033)

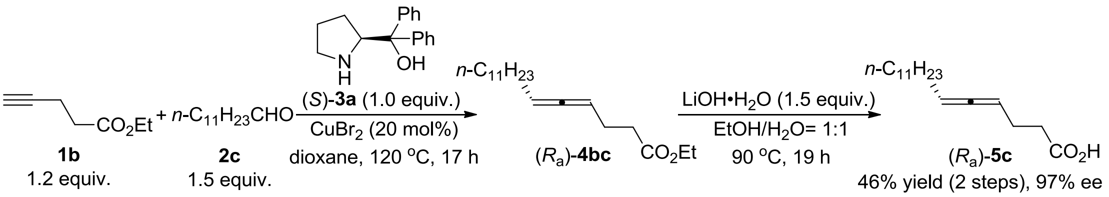

Following **Typical Procedure IV**, the reaction of $CuBr_2$ (0.6767 g, 3.0 mmol, 99%), (*S*)-**3a** (3.8698 g, 15 mmol, 98%), **1b** (2.2795 g, 18 mmol)/dioxane (15 mL), and *n*-dodecanal **2c** (4.98 mL, d = 0.831 g cm^{-3}, 4.1384 g, 22.49 mmol)/dioxane (10 mL) for 17 h afforded product (*R*a)-**4bc** (1.9936 g) [(eluent: petroleum ether (60-90 °C) (500 mL) to petroleum ether (60-90 °C)/diethyl ether = 100/1 (500 mL × 2)] as a liquid, which was then submitted to next step.

Following **Typical Procedure II**, the reaction of product (*R*a)-**4bc** (1.9936 g, 6.78 mmol, prepared above), EtOH/H_2O = 1:1 by volume (pre-mixed by using 33.5 mL of H_2O and 33.5 mL of EtOH), and LiOH·H_2O (0.4460 g, 10.17 mmol, 95%) for 19 h afforded (*R*a)-**5c** (1.8322 g, 46%, 2 steps) [(eluent: petroleum ether (60-90 °C)/ethyl acetate = 20/1 (500 mL) to 10/1 (500 mL × 3)] as an oil: 97% ee (determined by the corrsponding esterification product (*R*a)-**4bc**); $[\alpha]_D^{20}$ = -56.5 (c = 1.00, $CHCl_3$); ^{1}H NMR (300 MHz, $CDCl_3$) δ 11.39 (bs, 1H, COOH), 5.24-5.07 (m, 2H, =CH × 2), 2.48 (t, *J* = 7.2 Hz, 2H, CH_2), 2.36-2.23 (m, 2H, CH_2), 2.04-1.90 (m, 2H, CH_2), 1.45-1.18

(m, 18H, CH_2 × 9), 0.88 (t, J = 6.6 Hz, 3H, CH_3); ^{13}C NMR (75 MHz, $CDCl_3$) δ 203.7, 179.9, 92.8, 89.2, 33.1, 31.9, 29.6, 29.5, 29.4, 29.2, 29.1, 28.8, 23.5, 22.7, 14.1; IR (neat) ν (cm^{-1}) 3657-2311 (COOH), 2955, 2924, 2854, 1963, 1712, 1438, 1278, 1210, 1196, 1132, 1077; MS (70 ev, EI) *m/z* (%) 267 (M^+ + 1, 2.88), 266 (M^+, 5.98), 126 (100); HRMS calcd for $C_{17}H_{30}O_2$ [M^+]: 266.2246, found: 266.2251.

Esterification for determination of the ee value of (R_a)-**5c**: synthesis of (R_a)-ethyl 4,5-heptadecadienoate (R_a)-**4bc** (zj-4-111)

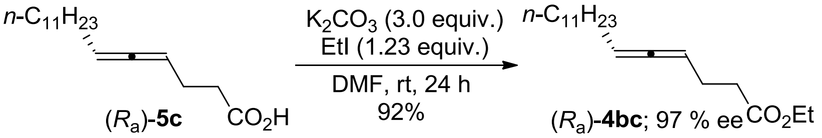

Following **Typical Procedure III**, the reaction of (R_a)-**5c** (0.1326 g, 0.5 mmol)/DMF (5 mL), EtI (0.05 mL, d = 1.94 g cm^{-3}, 0.0960 g, 0.616 mmol, 99%), and K_2CO_3 (0.2079 g, 1.5 mmol) for 24 h afforded (R_a)-**4bc** (0.1355 g, 92%) [eluent: petroleum ether (60-90 ^{o}C)/ethyl acetate = 100/1 (500 mL)] as a liquid: 97% ee (HPLC conditions: Chiralcel OD-H column, *n*-hexane, 0.7 mL/min, λ = 214 nm, t_R (major) = 14.2 min, t_R (minor) = 13.9 min); $[\alpha]_D^{20}$ = -51.0 (c = 1.00, $CHCl_3$); ^{1}H NMR (300 MHz, $CDCl_3$) δ 5.19-5.07 (m, 2H, =CH × 2), 4.13 (q, J = 7.1 Hz, 2H, OCH_2), 2.47-2.37 (m, 2H, CH_2), 2.36-2.23 (m, 2H, CH_2), 2.02-1.89 (m, 2H, CH_2), 1.45-1.15 (m, 21H, CH_3 + CH_2 × 9), 0.88 (t, J = 6.6 Hz, 3H, CH_3); ^{13}C NMR (75 MHz, $CDCl_3$) δ 203.6, 173.1, 92.5, 89.5, 60.2, 33.4, 31.9, 29.6, 29.5, 29.3, 29.1, 28.9, 23.9, 22.6, 14.2, 14.1; IR (neat) ν (cm^{-1}) 2956, 2925, 2854, 1963, 1739, 1466, 1372, 1350, 1300, 1250, 1159, 1097, 1038; MS (70 ev, EI) *m/z* (%) 294 (M^+, 6.39), 80 (100); HRMS calcd for $C_{19}H_{34}O_2$ [M^+]: 294.2559, found: 294.2556.

5. Synthesis of (R_a)-7-methyl-4,5-octadienoic acid (R_a)-**5d** (zj-8-048, 8-051)

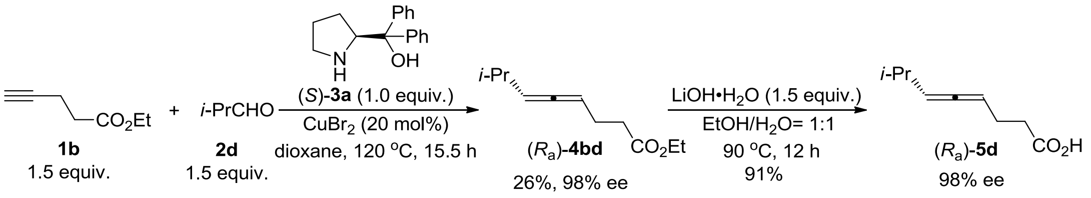

Following **Typical Procedure I**, the reaction of $CuBr_2$ (0.9014 g, 4 mmol, 99%),

(S)-**3a** (5.1672 g, 20 mmol, 98%), **1b** (3.7830 g, 30 mmol)/dioxane (35 mL), and isobutaldehyde **2d** (2.73 mL, d = 0.79 g cm^{-3}, 2.1567 g, 29.95 mmol)/dioxane (15 mL) for 15.5 h afforded product (R_a)-**4bd** (0.9565 g, 26%) [(eluent: petroleum ether (60-90 ^oC)/diethyl ether = 100/1 (500 mL × 3)] as a liquid: 98% ee (HPLC conditions: Chiralcel OD-H column, *n*-hexane, 1.0 mL/min, λ = 214 nm, t_R (major) = 12.9 min, t_R (minor) = 12.2 min); $[\alpha]_D^{20}$ = -66.0 (c = 1.00, $CHCl_3$); ^1H NMR (300 MHz, $CDCl_3$) δ 5.26-5.12 (m, 2H, =CH × 2), 4.13 (q, J = 7.2 Hz, 2H, CH_2), 2.46-2.36 (m, 2H, CH_2), 2.35-2.18 (m, 3H, CH + CH_2), 1.26 (t, J = 7.2 Hz, 3H, CH_3), 0.99 (d, J = 6.9 Hz, 6H, CH_3 × 2); ^{13}C NMR (75 MHz, $CDCl_3$) δ 202.0, 172.9, 99.8, 90.7, 60.1, 33.3, 27.8, 23.8, 22.3, 22.2, 14.1; IR (neat) ν (cm^{-1}) 2961, 2925, 2869, 1961, 1738, 1466, 1447, 1420, 1372, 1350, 1299, 1251, 1227, 1199, 1159, 1097, 1055, 1029; MS (70 ev, EI) m/z (%) 182 (M^+, 27.17), 93 (100); HRMS calcd for $C_{11}H_{18}O_2$ [M^+]: 182.1307, found: 182.1310.

Following **Typical Procedure II**, the reaction of (R_a)-**4bd** (0.8194 g, 4.5 mmol, prepared above), EtOH/H_2O = 1:1 by volume (pre-mixed by using 22.5 mL of H_2O and 22.5 mL of EtOH), and LiOH·H_2O (0.2990 g, 6.75 mmol, 95%) for 12 h afforded (R_a)-**5d** (0.6317 g, 91%) [(eluent: petroleum ether (60-90 ^oC)/ethyl acetate = 15/1 (480 mL) to 10/1 (550 mL) to 5/1 (600 mL)] as an oil: 98% ee (determined by the corrsponding esterification product (R_a)-**4bd**); $[\alpha]_D^{20}$ = -71.8 (c = 1.00, $CHCl_3$); ^1H NMR (300 MHz, $CDCl_3$) δ 10.90 (bs, 1H, COOH), 5.28-5.16 (m, 2H, =CH × 2), 2.48 (t, J = 7.2 Hz, 2H, CH_2), 2.38-2.18 (m, 3H, CH + CH_2), 0.99 (d, J = 6.9 Hz, 6H, CH_3 × 2); ^{13}C NMR (75 MHz, $CDCl_3$) δ 202.1, 179.9, 100.3, 90.5, 33.1, 27.9, 23.5, 22.4, 22.3; IR (neat) ν (cm^{-1}) 3749-2203 (COOH), 3036, 2961, 2923, 2869, 2661, 1961, 1713, 1412, 1382, 1364, 1297, 1251, 1212, 1169, 1107, 1080, 1049, 1018; MS (70 ev, EI) *m/z* (%) 154 (M^+, 14.69), 67 (100); HRMS calcd for $C_9H_{14}O_2$ [M^+]: 154.0994, found: 154.0991.

Esterification for determination of the ee value of (R_a)-**5d**: synthesis of (R_a)-ethyl 7-methyl-4,5-octadienoate (R_a)-**4bd** (zj-8-063)

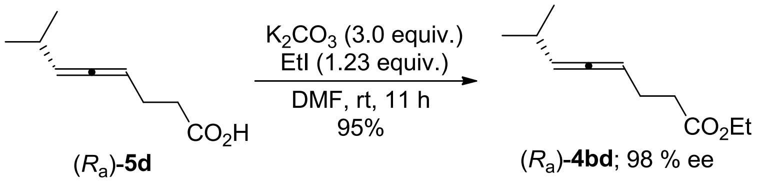

Following **Typical Procedure III**, the reaction of (R_a)-**5d** (0.0773 g, 0.5 mmol)/DMF (5 mL), EtI (0.05 mL, d = 1.94 g cm^{-3}, 0.096 g, 0.616 mmol, 99%), and K_2CO_3 (0.2075 g, 1.5 mmol) for 11 h afforded (R_a)-**4bd** (0.0867 g, 95%) [eluent: petroleum ether (60-90 ^oC)/ethyl acetate = 50/1 (800 mL)] as a liquid: 98% ee (HPLC conditions: Chiralcel OD-H column, *n*-hexane, 1.0 mL/min, λ = 214 nm, t_R (major) = 11.4 min, t_R (minor) = 10.8 min); $[\alpha]_D^{20}$ = -66.4 (c = 1.02, $CHCl_3$); ^1H NMR (300 MHz, $CDCl_3$) δ 5.28-5.11 (m, 2H, =CH × 2), 4.13 (q, *J* = 7.2 Hz, 2H, OCH_2), 2.45-2.37 (m, 2H, CH_2), 2.36-2.18 (m, 3H, CH + CH_2), 1.26 (t, *J* = 7.1 Hz, 3H, CH_3), 0.99 (d, *J* = 6.9 Hz, 6H, CH_3 × 2); ^{13}C NMR (75 MHz, $CDCl_3$) δ 202.1, 173.1, 99.9, 90.7, 60.2, 33.3, 27.8, 23.9, 22.4, 22.3, 14.1.

6. Synthesis of (R_a)-6-cyclohexyl-4,5-hexadienoic acid (R_a)-**5e** (zj-3-175, 3-196)

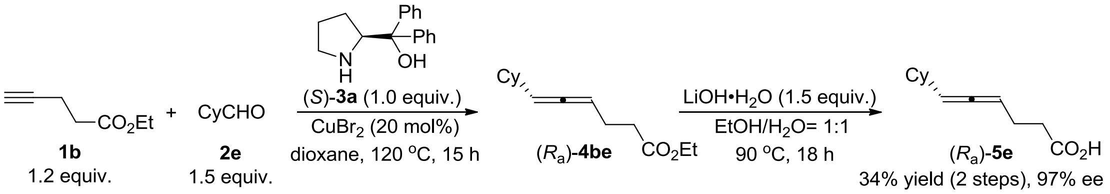

Following **Typical Procedure IV**, the reaction of $CuBr_2$ (0.6757 g, 3 mmol, 99%), (*S*)-**3a** (3.8745 g, 15 mmol， 98%), **1b** (2.2739 g, 18 mmol)/dioxane (17 mL), and cyclohexanaldehyde **2e** (2.72 mL, d = 0.926 g cm^{-3}, 2.5187 g, 22.49 mmol)/dioxane (8 mL) for 15 h afforded product (R_a)-**4be** (1.1104 g) [(eluent: petroleum ether (60-90 ^oC)/diethyl ether = 100/1 (500 mL × 3)] as a liquid, which was then submitted to next step.

Typical Procedure V: To a round-bottom flask were added (R_a)-**4be** (1.1104 g, 5.0 mmol, prepared above), EtOH/H_2O = 1:1 by volume (pre-mixed by using 25 mL of H_2O and 25 mL of EtOH), and LiOH H_2O (0.3321 g, 7.5 mmol, 95%) sequentially. After continuous stirring for 18 h under reflux at 90 ^oC, the reaction was complete as monitored by TLC. Then the mixture was cooled down to room temperature. After

evaporation to remove EtOH, the resulting mixture was extracted with 5 mL of Et_2O. The organic layer was separated and the aqueous layer was acidified with an aqueous solution of hydrochloric acid (aq., 3.0 M) until pH = 1. The aqueous layer was then extracted with Et_2O (20 mL × 3). The combined organic layer was washed with brine and dried over anhydrous Na_2SO_4. Filtration, evaporation, and column chromatography on silica gel [(eluent: petroleum ether (60-90 °C)/ethyl acetate = 8/1 (450 mL) to 5/1 (500 mL × 3)] gave impure (R_a)-**5e** (0.9991) as an oil; then the impure (R_a)-**5e** was purified again on silica gel [(eluent: petroleum ether (30-60 °C)/ethyl acetate = 8/1 (270 mL) to 5/1 (500 mL × 2)] to afford pure (R_a)-**5e** (0.9826 g, 34%, 2 steps) as an oil: 97% ee (determined by the corrsponding esterification product (R_a)-**4be**); $[\alpha]_D^{20}$ = -91.6 (c = 1.005, $CHCl_3$); ^1H NMR (300 MHz, $CDCl_3$) δ 11.18 (bs, 1H, COOH), 5.26-5.11 (m, 2H, =CH × 2), 2.55-2.42 (m, 2H, CH_2), 2.36-2.22 (m, 2H, CH_2), 2.01-1.86 (m, 1H, CH), 1.81-1.55 (m, 5H, one proton of CH_2 + CH_2 × 2), 1.35-0.95 (m, 5H, one proton of CH_2 + CH_2 × 2); ^{13}C NMR (75 MHz, $CDCl_3$) δ 202.4, 180.0, 98.9, 90.1, 37.1, 33.1, 32.9, 26.1, 26.0, 23.5; IR (neat) ν (cm^{-1}) 3624-2266 (COOH), 3032, 2924, 2851, 2659, 1962, 1712, 1448, 1412, 1335, 1288, 1250, 1211, 1175, 1132, 1077; MS (70 ev, EI) *m/z* (%) 194 (M^+, 2.79), 41 (100); HRMS calcd for $C_{12}H_{18}O_2$ [M^+]: 194.1307, found: 194.1305.

Esterification for determination of the ee value of (R_a)-**5e**: synthesis of (R_a)-ethyl 6-cyclohexyl-4,5-hexadienoate (R_a)-**4be**[6] (zj-4-006)

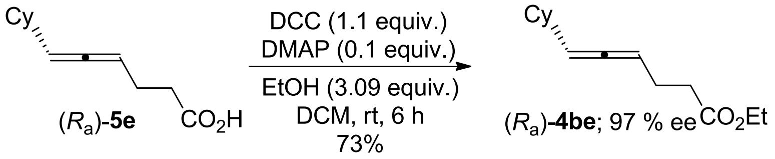

To a reaction tube were added (R_a)-**5e** (0.0978 g, 0.5 mmol, prapared above), EtOH (0.09 mL, d = 0.789 g cm^{-3}, 0.071g, 1.54 mmol), and DCM (5 mL). Then DCC (0.1160 g, 0.55 mmol, 98%) and DMAP (0.0062 g, 0.05 mmol, 98%) were added sequentially. After continuous stirring at room temperature for 6 h, the reaction was complete as monitored by TLC. After quenching with water (10 mL) with stirring for 10 min, the aqueous solution was extracted with Et_2O (10 mL × 3). The combined

organic layer was washed with brine and dried over anhydrous Na_2SO_4. Filtration, evaporation, and column chromatography on silica gel gave (R_a)-**4be** (0.0933 g, impure) [(eluent: petroleum ether (60-90 ^oC)/ethyl acetate = 100/1 (800 mL)] as a liquid. The impure (R_a)-**4be** was diluted with 10 mL of Et_2O and then washed with an aqueous solution of hydrochloric acid (aq., 3.0 M) (10 mL × 3) and brine. After drying over anhydrous Na_2SO_4, filtration, evaporation, and column chromatography on silica gel gave pure (R_a)-**4be** (0.0817 g, 73%) [(eluent: petroleum ether (60-90 ^oC)/ethyl acetate = 100/1 (800 mL)] as a liquid: 97% ee (HPLC conditions: Chiralcel AY column, *n*-hexane, 0.6 mL/min, λ = 214 nm, t_R (major) = 17.32 min), $[\alpha]_D^{20}$ = -81.9 (c = 0.99, $CHCl_3$); ^1H NMR (300 MHz, $CDCl_3$) δ 5.26-5.08 (m, 2H, =CH × 2), 4.13 (q, J = 7.1 Hz, 2H, OCH_2), 2.48-2.36 (m, 2H, CH_2), 2.35-2.22 (m, 2H, CH_2), 2.01-1.84 (m, 1H, CH), 1.81-1.56 (m, 5H, one proton of CH_2 + CH_2 × 2), 1.36-0.95 (m, 8H, CH_3 + one proton of CH_2 + CH_2 × 2); ^{13}C NMR (75 MHz, $CDCl_3$) δ 202.4, 173.1, 98.5, 90.4, 60.2, 37.1, 33.3, 33.0, 32.9, 26.1, 26.0, 23.8, 14.1; IR (neat) ν (cm^{-1}) 2977, 2925, 2851, 1960, 1738, 1448, 1418, 1372, 1348, 1302, 1290, 1250, 1227, 1195, 1158, 1096, 1037; MS (70 ev, EI) *m/z* (%) 222 (M^+, 6.25), 29 (100); HRMS calcd for $C_{14}H_{22}O_2$ [M^+]: 222.1620, found: 222.1618;

7. Synthesis of (R_a)-7-phenyl-4,5-heptadienoic acid (R_a)-**5f** (zj-4-163, 4-169)

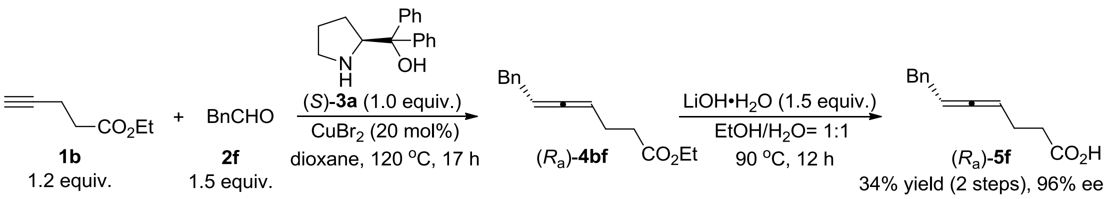

Following **Typical Procedure IV**, the reaction of $CuBr_2$ (0.6763 g, 3.0 mmol, 99%), (S)-**3a** (3.8769 g, 15 mmol, 98%), **1b** (2.2719 g, 18 mmol)/dioxane (15 mL), and phenylacetaldehyde **2f** (2.69 mL, d = 1.025 g cm^{-3}, 2.7021 g, 22.52 mmol, 98%)/dioxane (10 mL) for 17 h afforded (R_a)-**4bf** (1.3907 g) [(eluent: petroleum ether (60-90 ^oC)/diethyl ether = 100/1 (500 mL × 2) to 50/1 (500 mL × 2)] as a liquid, which was then submitted to next step.

Following **Typical Procedure V**, the reaction of (R_a)-**4bf** (1.3907 g, 6.0 mmol,

prepared above), EtOH/H_2O = 1:1 by volume (pre-mixed by using 30 mL of H_2O and 30 mL of EtOH), and $LiOH\cdot H_2O$ (0.4017 g, 9.0 mmol, 95%) for 12 h afforded (R_a)-**5f** (1.0324 g, 34%, 2 steps) [(eluent: petroleum ether (60-90 ^oC)/ethyl acetate = 20/1 (420 mL) to 10/1 (500 mL × 2)] as an oil: 96% ee (determined by the corrsponding esterification product (R_a)-**4bf**); $[\alpha]_D^{20}$ = -79.7 (c = 1.005, $CHCl_3$); ^1H NMR (300 MHz, $CDCl_3$) δ 10.53 (bs, 1H, COOH), 7.33-7.24 (m, 2H, ArH), 7.24-7.11 (m, 3H, ArH), 5.39-5.26 (m, 1H, =CH), 5.25-5.12 (m, 1H, =CH), 3.31 (dd, J_1 = 6.9 Hz, J_2 = 2.7 Hz, 2H, CH_2), 2.46-2.37 (m, 2H, CH_2), 2.34-2.21 (m, 2H, CH_2); ^{13}C NMR (75 MHz, $CDCl_3$) δ 204.2, 179.7, 140.1, 128.4, 128.3, 126.1, 92.2, 89.9, 35.5, 33.0, 23.4; IR (neat) ν (cm^{-1}) 3600-2197 (COOH), 3084, 3060, 3028, 2980, 2915, 2665, 1964, 1712, 1603, 1494, 1453, 1428, 1334, 1279, 1251, 1212, 1173, 1075, 1029; MS (70 ev, EI) *m/z* (%) 202 (M^+, 4.51), 143 (100), 142 (100), 129 (100), 128 (100), 91 (100); HRMS calcd for $C_{13}H_{14}O_2$ [M^+]: 202.0994, found: 202.0997.

Esterification for determination of the ee value of (R_a)-**5f**: synthesis of (R_a)-ethyl 7-phenyl-4,5-heptadienoate (R_a)-**4bf** (zj-4-174)

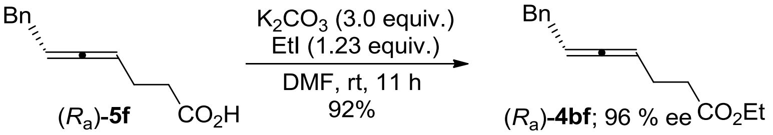

Following **Typical Procedure III**, the reaction of (R_a)-**5f** (0.1017 g, 0.5 mmol)/DMF (5 mL), EtI (0.05 mL, d = 1.94 g cm^{-3}, 0.096 g, 0.616 mmol, 99%), and K_2CO_3 (0.2075 g, 1.5 mmol) for 11 h afforded (R_a)-**4bf** (0.1066 g, 92%) [eluent: petroleum ether (60-90 ^oC)/ethyl acetate = 100/1 (500 mL)] as a liquid: 96% ee (HPLC conditions: Chiralcel AS-H column, *n*-hexane, 1.0 mL/min, λ = 214 nm, t_R (major) = 20.3 min, t_R (minor) = 23.3 min); $[\alpha]_D^{20}$ = -75.3 (c = 1.00, $CHCl_3$); ^1H NMR (300 MHz, $CDCl_3$) δ 7.33-7.24 (m, 2H, ArH), 7.24-7.13 (m, 3H, ArH), 5.35-5.24 (m, 1H, =CH), 5.24-5.13 (m, 1H, =CH), 4.10 (q, J = 7.2 Hz, 2H, OCH_2), 3.31 (dd, J_1 = 7.1 Hz, J_2 = 2.9 Hz, 2H, CH_2), 2.42-2.22 (m, 4H, CH_2 × 2), 1.23 (t, J = 7.2 Hz, 3H, CH_3); ^{13}C NMR (75 MHz, $CDCl_3$) δ 204.2, 172.9, 140.1, 128.3, 128.2, 126.0, 91.7, 90.2, 60.1, 35.5, 33.2, 23.7, 14.1; IR (neat) ν (cm^{-1}) 3084, 3060, 3027, 2980, 2908,

2871, 2848, 1963, 1735, 1603, 1494, 1453, 1420, 1372, 1350, 1301, 1251, 1159, 1096, 1054, 1030; MS (70 ev, EI) *m/z* (%) 230 (M^+, 10.54), 91 (100); HRMS calcd for $C_{15}H_{18}O_2$ [M^+]: 230.1307, found: 230.1311.

8. Synthesis of (*R*a)-8-phenyl-4,5-octadienoic acid (*R*a)-**5g** (zj-4-136, 4-139)

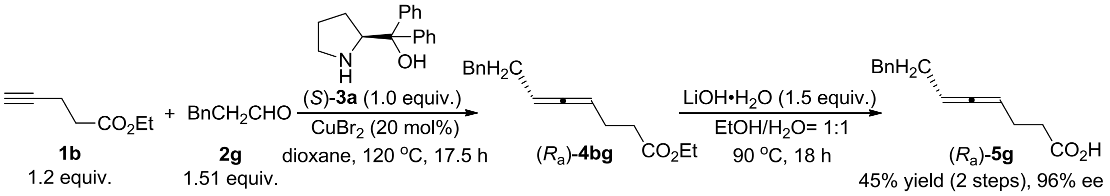

Following **Typical Procedure IV**, the reaction of $CuBr_2$ (0.6764 g, 3.0 mmol, 99%), (*S*)-**3a** (3.8655 g, 15 mmol, 98%), **1b** (2.2671 g, 18 mmol)/dioxane (15 mL), and phenylpropyl aldehyde **2g** (3.0 mL, d = 1.015 g cm^{-3}, 3.045 g, 22.7 mmol)/dioxane (10 mL) for 17.5 h afforded (*R*a)-**4bg** (1.8685 g) [(eluent: petroleum ether (60-90 ^{o}C)/diethyl ether = 100/1 (500 mL × 3) to 50/1 (500 mL × 2)] as a liquid, which was then submitted to next step.

Following **Typical Procedure V**, the reaction of (*R*a)-**4bg** (1.8685 g, 7.66 mmol, prepared above), EtOH/H_2O = 1:1 by volume (pre-mixed by using 30 mL of H_2O and 30 mL of EtOH), and LiOH H_2O (0.5082 g, 11.5 mmol, 95%) for 18 h afforded (*R*a)-**5g** (1.4516 g, 45%, 2 steps) [(eluent: petroleum ether (60-90 ^{o}C)/ethyl acetate = 20/1 (220 mL) to 10/1 (500 mL) to 5/1 (500 mL × 2)] as an oil: 96% ee (determined by the corrsponding esterification product (*R*a)-**4bg**); $[\alpha]_D^{20}$ = -71.8 (c = 1.005, $CHCl_3$); ^{1}H NMR (300 MHz, $CDCl_3$) δ 11.15 (bs, 1H, COOH), 7.32-7.23 (m, 2H, ArH), 7.23-7.13 (m, 3H, ArH), 5.27-5.10 (m, 2H, =CH × 2), 2.76-2.65 (m, 2H, CH_2), 2.42-2.15 (m, 6H, CH_2 × 3); ^{13}C NMR (75 MHz, $CDCl_3$) δ 203.8, 179.7, 141.7, 128.5, 128.2, 125.8, 92.1, 89.8, 35.2, 32.9, 30.4, 23.3; IR (neat) ν (cm^{-1}) 3696-2194 (COOH), 3087, 3062, 3027, 2974, 2920, 2856, 2660, 1963, 1713, 1603, 1496, 1454, 1435, 1411, 1336, 1278, 1250, 1211, 1175, 1133, 1078, 1030; MS (70 ev, EI) *m/z* (%) 216 (M^+, 4.92), 129 (100), 91 (100); HRMS calcd for $C_{14}H_{16}O_2$ [M^+]: 216.1150, found: 216.1154.

Esterification for determination of the ee value of (R_a)-**5g**: synthesis of (R_a)-ethyl 8-phenyl-4,5-octadienoate (R_a)-**4bg** (zj-4-141)

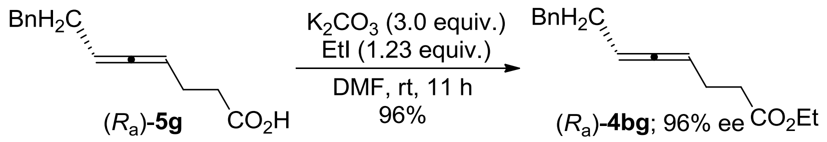

Following **Typical Procedure III**, the reaction of (R_a)-**5g** (0.1081 g, 0.5 mmol)/DMF (5 mL), EtI (0.05 mL, d = 1.94 g cm^{-3}, 0.096 g, 0.616 mmol, 99%), and K_2CO_3 (0.2079 g, 1.5 mmol) for 11 h afforded (R_a)-**4bg** (0.1177 g, 96%) [eluent: petroleum ether (60-90 ^oC)/ethyl acetate = 100/1 (800 mL)] as a liquid: 96% ee (HPLC conditions: Chiralcel AS-H column, *n*-hexane, 0.7 mL/min, λ = 214 nm, t_R (major) = 18.5 min, t_R (minor) = 22.8 min); $[\alpha]_D^{20}$ = -71.3 (c = 1.00, $CHCl_3$); ^{1}H NMR (300 MHz, $CDCl_3$) δ 7.31-7.22 (m, 2H, ArH), 7.22-7.09 (m, 3H, ArH), 5.26-5.09 (m, 2H, =CH × 2), 4.12 (q, *J* = 7.3 Hz, 2H, OCH_2), 2.70 (t, *J* = 7.7 Hz, 2H, CH_2), 2.39-2.18 (m, 6H, CH_2 × 3), 1.25 (t, *J* = 7.2 Hz, 3H, CH_3); ^{13}C NMR (75 MHz, $CDCl_3$) δ 203.8, 173.0, 141.6, 128.4, 128.2, 125.8, 91.7, 90.1, 60.2, 35.2, 33.3, 30.4, 23.7, 14.2; IR (neat) ν (cm^{-1}) 3085, 3062, 3027, 2979, 2924, 2855, 1963, 1737, 1603, 1496, 1454, 1372, 1349, 1300, 1250, 1196, 1158, 1133, 1096, 1077, 1030; MS (70 ev, EI) m/z (%) 244 (M^+, 3.56), 129 (100), 91 (100); HRMS calcd for $C_{16}H_{20}O_2$ [M^+]: 244.1463, found: 244.1470.

9. Synthesis of (R_a)-4,5,15-hexadecatrienoic acid (R_a)-**5h** (zj-5-035, 5-056)

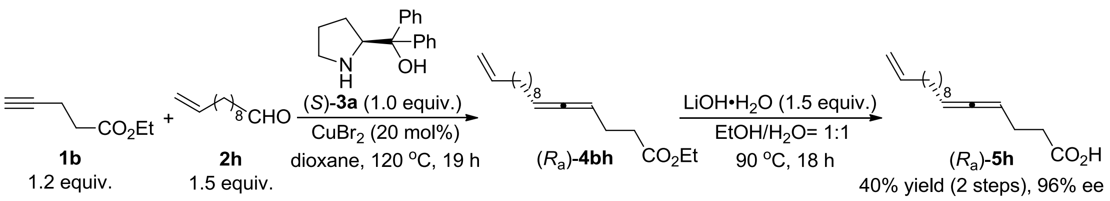

Following **Typical Procedure IV**, the reaction of $CuBr_2$ (0.6760 g, 3.0 mmol, 99%), (*S*)-**3a** (3.8667 g, 15 mmol, 98%), **1b** (2.2702 g, 18 mmol)/dioxane (15 mL), and 10-undecylenealdehyde **2h** (4.6 mL, d = 0.84 g cm^{-3}, 3.7867 g, 22.54 mmol, 98%)/dioxane (10 mL) for 19 h afforded (R_a)-**4bh** (2.6107 g) [(eluent: petroleum ether (60-90 ^oC)/diethyl ether = 100/1 (500 mL × 3) to 50/1 (500 mL)] as a liquid, which

was then submitted to next step.

Following **Typical Procedure V**, the reaction of (R_a)-**4bh** (2.6107 g, 9.4 mmol, prepared above), EtOH/H_2O = 1:1 by volume (pre-mixed by using 30 mL of H_2O and 30 mL of EtOH), and LiOH·H_2O (0.6235 g, 14.1 mmol, 95%) for 18 h afforded (R_a)-**5h**[4] (1.5036 g, 40%, 2 steps) [(eluent: petroleum ether (60-90 ^{o}C)/ethyl acetate = 10/1 (600 mL) to 5/1 (540 mL)] as an oil: 96% ee (determined by the corrsponding esterification product (R_a)-**4bh**); $[\alpha]_D^{20}$ = -60.3 (c = 1.015, $CHCl_3$); ^{1}H NMR (300 MHz, $CDCl_3$) δ 10.56 (bs, 1H, COOH), 5.89-5.70 (m, 1H, =CH), 5.25-5.09 (m, 2H, 2 × =CH), 5.05-4.86 (m, 2H, =CH_2), 2.56-2.42 (m, 2H, CH_2), 2.36-2.23 (m, 2H, CH_2), 2.11-1.90 (m, 4H, 2 × CH_2), 1.44-1.20 (m, 12H, 6 × CH_2); ^{13}C NMR (75 MHz, $CDCl_3$) δ 203.7, 179.8, 139.2, 114.1, 92.8, 89.2, 33.8, 33.1, 29.43, 29.37, 29.09, 29.07, 28.89, 28.81, 23.48; IR (neat) ν (cm^{-1}) 3672-2224 (COOH), 3075, 2977, 2925, 2854, 2664, 1964, 1711, 1640, 1436, 1411, 1337, 1280, 1249, 1211, 1172; MS (70 ev, EI) *m/z* (%) 251 (M^+ + 1, 2.66), 250 (M^+, 3.26), 126 (100), 81 (100).

Esterification for determination of the ee value of (R_a)-**5h**: synthesis of (R_a)-ethyl -4,5,15-hexadecatrienoate (R_a)-**4bh** (zj-5-065)

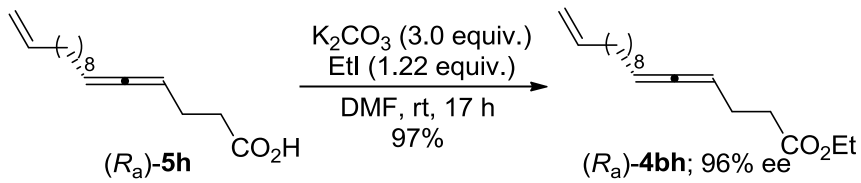

Following **Typical Procedure III**, the reaction of (R_a)-**5h** (0.1020 g, 0.4 mmol)/DMF (4 mL), EtI (0.04 mL, d = 1.94 g cm^{-3}, 0.076 g, 0.49 mmol, 98%), and K_2CO_3 (0.1673 g, 1.2 mmol) for 17 h afforded (R_a)-**4bh** (0.1100 g, 97%) [eluent: petroleum ether (60-90 ^{o}C)/ethyl acetate = 100/1 (700 mL)] as a liquid: 96% ee (HPLC conditions: Chiralcel OZ-H column, *n*-hexane, 0.5 mL/min, λ = 214 nm, t_R (major) = 22.0 min, t_R (minor) = 21.0 min); $[\alpha]_D^{20}$ = -53.9 (c = 0.995, $CHCl_3$); ^{1}H NMR (300 MHz, $CDCl_3$) δ 5.86-5.72 (m, 1H, =CH), 5.18-5.06 (m, 2H, 2 × =CH), 5.04-4.87 (m, 2H, =CH_2), 4.12 (q, J = 7.1 Hz, 2H, OCH_2), 2.45-2.36 (m, 2H, CH_2), 2.34-2.22 (m, 2H, CH_2), 2.09-1.90 (m, 4H, 2 × CH_2), 1.44-1.19 (m, 15H, 6 × CH_2 +

CH_3); ^{13}C NMR (75 MHz, $CDCl_3$) δ 203.6, 172.9, 139.0, 114.0, 92.3, 89.4, 60.1, 33.7, 33.3, 29.32, 29.28, 29.0, 28.8, 28.7, 23.8, 14.1; IR (neat) ν (cm^{-1}) 3076, 2978, 2926, 2854, 1962, 1738, 1640, 1462, 1444, 1371, 1348, 1299, 1249, 1159, 1096, 1037; MS (70 ev, EI) m/z (%) 278 (M^+, 20.44), 279 (M^+ + 1, 6.16), 154 (100); HRMS calcd for $C_{18}H_{30}O_2$ [M^+]: 278.2246, found: 278.2249.

10. Synthesis of (R_a)-14-(*tert*-butyldimethylsilyl)-4,5-tetradecadien-13-ynoic acid (R_a)-**5i** (zj-6-184, 6-186)

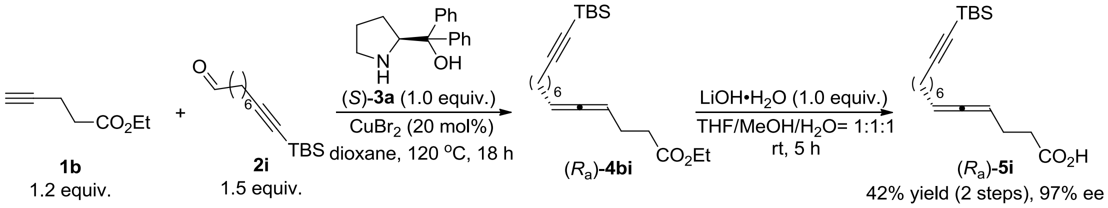

Following **Typical Procedure IV**, the reaction of $CuBr_2$ (0.3155 g, 1.4 mmol, 99%), (*S*)-**3a** (1.8071 g, 7 mmol, 98%), **1b** (1.0575 g, 8.4 mmol)/dioxane (25 mL), and aldehyde **2i**[7] (2.6397 g, 10.5 mmol; TBS: *tert*-butyldimethylsilyl)/dioxane (10 mL) for 18 h afforded (R_a)-**4bi** (1.2865 g) [(eluent: petroleum ether (60-90 °C)/diethyl ether = 100/1 (500 mL × 2) to 50/1 (500 mL) to 30/1 (600 mL)] as a liquid, which was then submitted to next step.

To a round-bottom flask were added (R_a)-**4bi** (1.2865 g, 3.55 mmol, prepared above), THF/MeOH/H_2O = 1:1:1 by volume (pre-mixed by using 6.5 mL of THF, 6.5 mL of MeOH and 6.5 mL of H_2O), and LiOH H_2O (0.1571 g, 3.55 mmol, 95%) sequentially. After continuous stirring for 5 h at room temperature, the reaction was complete as monitored by TLC.[8] Then MeOH and THF were removed via evaporation, the resulting mixture was acidified with an aqueous solution of hydrochloric acid (aq., 1.0 M) until pH = 1. The aqueous layer was then extracted with Et_2O (25 mL × 3). After being washed with brine and dried over anhydrous Na_2SO_4, filtration, evaporation, and column chromatography on silica gel gave (R_a)-**5i** (0.9826 g, 42%, 2 steps) [(eluent: petroleum ether (60-90 °C)/ethyl acetate = 15/1 (480 mL) to 10/1 (550 mL) to 8/1 (540 mL)] as an oil: 97% ee (determined by the corrsponding esterification

product (R_a)-**4bi**); $[\alpha]_D^{20}$ = -48.7 (c = 1.025, $CHCl_3$); ^{1}H NMR (300 MHz, $CDCl_3$) δ 10.92 (bs, 1H, COOH), 5.24-5.03 (m, 2H, =CH × 2), 2.45 (t, J = 7.4 Hz, 2H, CH_2), 2.36-2.24 (m, 2H, CH_2), 2.20 (t, J = 7.1 Hz, 2H, CH_2), 2.01-1.89 (m, 2H, CH_2), 1.57-1.24 (m, 8H, CH_2 × 4), 0.91 (s, 9H, CH_3 × 3), 0.06 (s, 6H, CH_3 × 2); ^{13}C NMR (75 MHz, $CDCl_3$) δ 203.7, 179.8, 108.1, 92.7, 89.3, 82.3, 33.1, 28.9, 28.7, 28.6, 28.5, 26.0, 23.4, 19.7, 16.4, -4.50; IR (neat) ν (cm^{-1}) 3685-2304 (COOH), 2929, 2856, 2660, 2172, 1964, 1711, 1463, 1432, 1413, 1360, 1332, 1251, 1211, 1167, 1009; MS (70 ev, EI) *m/z* (%) 335 (M^+ + 1, 11.48), 334 (M^+, 6.86), 75 (100); HRMS calcd for $C_{20}H_{34}O_2Si$ [M^+]: 334.2328, found: 334.2332.

Esterification for determination of the ee value of (R_a)-**5i**: synthesis of (R_a)-ethyl 14-(*tert*-butyldimethylsilyl)-4,5-tetradecadien-13-ynoate (R_a)-**4bi** (zj-6-191)

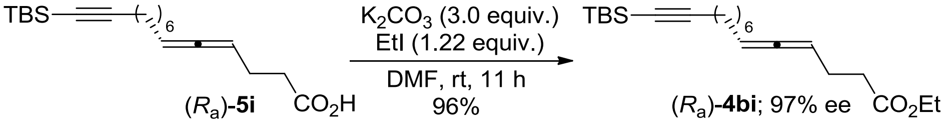

Following **Typical Procedure III**, the reaction of (R)-**5i** (0.1002 g, 0.3 mmol)/DMF (3 mL), EtI (0.03 mL, d = 1.94 g cm^{-3}, 0.057 g, 0.366 mmol, 98%), and K_2CO_3 (0.1242 g, 0.9 mmol) for 11 h afforded (R)-**4bi** (0.1035 g, 96%) [eluent: petroleum ether (60-90 ^{o}C)/ethyl acetate = 100/1 (300 mL) to 50/1 (400 mL)] as a liquid: 97% ee (HPLC conditions: Chiralcel OZ-H column, *n*-hexane, 1.0 mL/min, λ = 214 nm, t_R (major) = 30.5 min, t_R (minor) = 29.1 min); $[\alpha]_D^{20}$ = -45.3 (c = 1.00, $CHCl_3$); ^{1}H NMR (300 MHz, $CDCl_3$) δ 5.19-5.00 (m, 2H, =CH × 2), 4.10 (q, J = 7.2 Hz, 2H, OCH_2), 2.44-2.32 (m, 2H, CH_2), 2.32-2.22 (m, 2H, CH_2), 2.19 (t, J = 6.9 Hz, 2H, CH_2), 2.01-1.86 (m, 2H, CH_2), 1.54-1.26 (m, 8H, CH_2 × 4), 1.22 (t, J = 7.1 Hz, 3H, CH_3), 0.89 (s, 9H, CH_3 × 3), 0.04 (s, 6H, CH_3 × 2); ^{13}C NMR (75 MHz, $CDCl_3$) δ 203.6, 173.0, 108.0, 92.3, 89.5, 60.2, 33.4, 28.9, 28.7, 28.6, 28.5, 26.0, 23.8, 19.7, 16.4, 14.2, -4.5; IR (neat) ν (cm^{-1}) 2931, 2857, 2172, 1963, 1738, 1464, 1370, 1299, 1251, 1159, 1097, 1034; MS (70 ev, EI) m/z (%) 363 (M^+ + 1, 27.35), 362 (M^+, 16.06), 231 (100); HRMS calcd for $C_{22}H_{38}O_2Si$ [M^+]: 362.2641, found: 362.2646.

11. Esterification for determination of the ee value of (S_a)-**5m**: synthesis of (S_a)-ethyl 4,5-dodecadienoate (S_a)-**4bm** (zj-9-055)

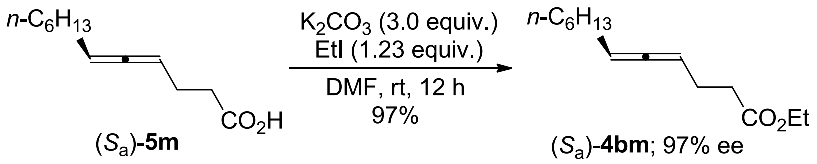

Following **Typical Procedure III**, the reaction of (S_a)-**5m**[4] (0.0982 g, 0.5 mmol)/DMF (5 mL), EtI (0.05 mL, d = 1.94 g cm^{-3}, 0.097 g, 0.62 mmol, 99%), and K_2CO_3 (0.2073 g, 1.5 mmol) for 12 h afforded (S_a)-**4bm** (0.1091 g, 97%) [eluent: petroleum ether (60-90 ^{o}C)/ethyl acetate = 50/1 (450 mL)] as a liquid: 97% ee (HPLC conditions: Chiralcel OD-H column, *n*-hexane, 1.0 mL/min, λ = 214 nm, t_R (major) = 15.2 min, t_R (minor) = 17.1 min); $[\alpha]_D^{20}$ = +63.9 (c = 1.00, $CHCl_3$); ^{1}H NMR (300 MHz, $CDCl_3$) δ 5.23-5.07 (m, 2H, =CH × 2), 4.13 (q, *J* = 7.2 Hz, 2H, OCH_2), 2.46-2.37 (m, 2H, CH_2), 2.36-2.23 (m, 2H, CH_2), 2.06-1.91 (m, 2H, CH_2), 1.44-1.19 (m, 11H, CH_2 × 4 + CH_3), 0.89 (t, *J* = 6.8 Hz, 3H, CH_3); ^{13}C NMR (75 MHz, $CDCl_3$) δ 203.6, 173.0, 92.4, 89.4, 60.1, 33.3, 31.6, 29.0, 28.8, 28.7, 23.8, 22.5, 14.1, 14.0; IR (neat) ν (cm^{-1}) 2957, 2927, 2856, 1963, 1738, 1465, 1446, 1372, 1350, 1300, 1251, 1196, 1180, 1159, 1097, 1076, 1039; MS (70 ev, EI) m/z (%) 224 (M$^+$, 5.38), 80 (100); HRMS calcd for $C_{14}H_{24}O_2$ [M$^+$]: 224.1776, found: 224.1782.

Synthesis of AuCl(LB-Phos)[9] (zj-7-180)

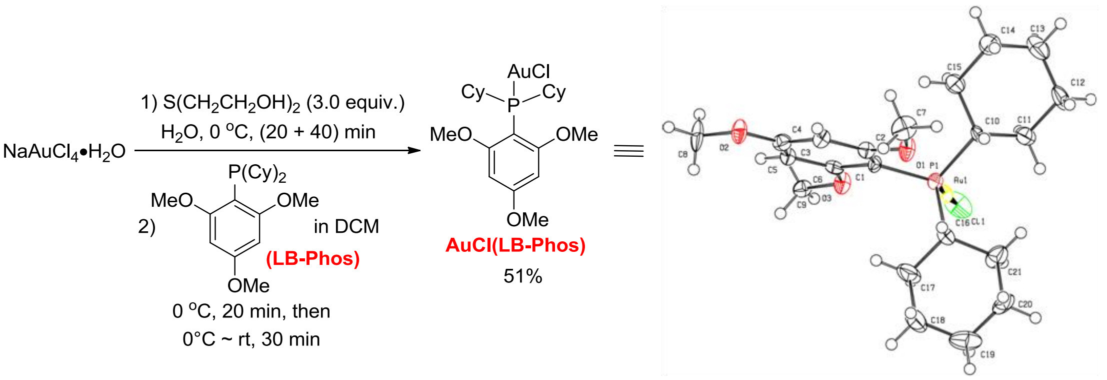

To a three-neck flask were added $NaAuCl_4 \cdot 2H_2O$ (0.5230 g, 1.3 mmol, 99%) and H_2O (20 mL). The solution was cooled to 0 ℃ by an ice bath, and $S(CH_2CH_2OH)_2$ (0.4759 g, 3.9 mmol,) in H_2O (5 mL) was added within 20 min. After the addition was

complete, the reaction mixture was stirred for 40 min at 0 °C. Then a solution of LB-Phos (0.4737 g, 1.3 mmol) in DCM (5 mL) was added dropwise at 0 °C within 20 min. Then the reaction was warmed up to room temperature. After stirring for 30 min, the reaction was complete as monitored by TLC. The reaction mixture was extracted with DCM (20 mL × 4). The combined organic layer was washed with brine and dried over anhydrous Na_2SO_4. After filtration and evaporation, the residue was purified by chromatography [eluent: petroleum ether (60-90 °C)/ethyl acetate = 5/1 (600 mL) to 3/1 (600 mL) to 2/1 (600 mL)] on silica gel to afford AuCl(LB-Phos) (0.3971 g, 51%) as a white solid, which is stable in air and H_2O: m. p. 217.5-218.7 °C (diethyl ether/dichloromethane); ^1H NMR (300 MHz, $CDCl_3$) δ 6.14 (d, 2H, J = 3.0 Hz, ArH), 3.87 (s, 3H, CH_3), 3.84 (s, 6H, CH_3 × 2), 2.53-2.37 (m, 2H, CH_2), 2.12-1.99 (m, 2H, CH_2), 1.88-1.45 (m, 10H, CH_2 × 5), 1.41-1.09 (m, 8H, CH_2 × 4); ^{13}C NMR (75 MHz, $CDCl_3$) δ 164.5, 163.9, 92.7 (d, J = 55.2 Hz), 91.3 (d, J = 4.9 Hz), 55.44, 55.36, 35.8 (d, J = 35.9 Hz), 32.2 (d, J = 5.5 Hz), 29.3, 26.7 (d, J = 13.1 Hz), 26.4 (d, J = 15.8 Hz), 25.6; ^{31}P NMR (121 MHz, $CDCl_3$) δ 31.3; IR (neat) ν (cm^{-1}) 3042, 3002, 2927, 2850, 1597, 1459, 1408, 1334, 1291, 1267, 1228, 1207, 1181, 1161, 1123, 1092, 1029, 1003; MS (70 ev, EI) *m/z* (%) 598 (M^+ + 1, 18.14), 597 (M^+, 12.34), 478 (100); Elemental analysis calcd (%) for $C_{21}H_{33}AuClO_3P$: C, 42.26; H, 5.57; Found: C, 42.18; H, 5.74.

Crystal data for AuCl(LB-Phos): $C_{21}H_{33}AuClO_3P$, M_W = 596.86, orthorhombic, space group P 21 21 21, final R indices [I > 2\s (I)], R_1 = 0.0321, wR_2 = 0.0701, R indices (all data) R_1 = 0.0365, wR_2 = 0.0667, a = 11.6086 (6) Å, b = 13.0711 (8) Å, c = 15.0247 (10) Å, α = 90°, β = 90°, γ = 90°, V = 2279.8 (2) $Å^3$, T = 170 K, Z = 4, reflections collected/unique: 14515/4141 (R_{int} = 0.0544), number of observations [> 2\s (I)] 3869, parameters: 247. CCDC 1558142.

Highly stereoselective synthesis of (*S*,*E*)-6

1. Synthesis of (*S*,*E*)-5-(1-nonenyl)dihydro-2(3*H*)-furanone (*S*,*E*)-**6a** (zj-8-046)

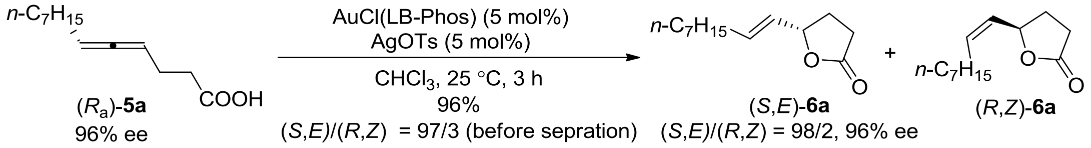

Typical Procedure VI: To a dry Schlenk tube were added AgOTs (0.0142 g, 0.05 mmol, weighed in glove box, 98%), AuCl(LB-Phos) (0.0299 g, 0.05 mmol), and $CHCl_3$ (5 mL) under nitrogen atmosphere sequentially. After stirring for 15 min, (*R*)-trideca-4,5-allenoic acid (R_a)-**5a** and $CHCl_3$ (5 mL) were added. After being continuously stirred at 25 ^{o}C for 3 h, the reaction was complete as monitored by TLC. Filtration through a short column of silica gel [eluent: Et_2O (20 mL × 3)] and evaporation afforded a crude mixture of (*S*,*E*)-**6a** and (*R*,*Z*)-**6a** ((*S*,*E*)/(*R*,*Z*) = 97/3, as determined by ^{1}H NMR analysis). Column chromatography on silica gel afforded (*S*,*E*)-**6a** (0.2017 g, 96%, (*S*,*E*)/(*R*,*Z*) = 98/2 as determined by ^{1}H NMR analysis) [eluent: petroleum ether (60-90 ^{o}C)/ethyl acetate = 15/1 (400 mL) to 10/1 (550 mL)] as an oil with pleasant flavor: 96% ee (HPLC conditions: Chiralcel OJ-H column, *n*-hexane/*i*-PrOH = 200/1, 1.0 mL/min, λ = 214 nm, t_R (major) = 22.73 min, t_R (minor) = 20.86 min); $[\alpha]_D^{20}$ = +29.6 (c = 1.01, $CHCl_3$); ^{1}H NMR (300 MHz, $CDCl_3$) δ 5.81 (dt, J_1 = 15.3 Hz, J_2 = 7.2 Hz, 1H, =CH), 5.49 (dd, J_1 = 15.3 Hz, J_2 = 7.2 Hz, 1H, =CH), 4.90 (q, J = 7.2 Hz, 1H, CH), 2.61-2.50 (m, 2H, CH_2), 2.46-2.31 (m, 1H, one proton from CH_2), 2.13-1.90 (m, 3H, CH_2 + one proton from CH_2), 1.45-1.18 (m, 10H, CH_2 × 5), 0.88 (t, J = 6.6 Hz, 3H, CH_3); the following signals are discernible for (*R*,*Z*)-**6a**: δ 5.72-5.62 (m, 1H, =CH), 5.31-5.21 (m, 1H, CH); ^{13}C NMR (75 MHz, $CDCl_3$) δ 177.0, 135.6, 127.2, 81.1, 32.0, 31.6, 29.0, 28.9, 28.7, 28.64, 28.61, 22.5, 14.0; IR (neat) ν (cm^{-1}) 2955, 2926, 2855, 1778, 1673, 1459, 1415, 1378, 1327, 1296, 1216, 1177, 1123, 1010; GC-MS (GC condition: injector: 280 ^{o}C; column: DB5 column 30 m × 0.25 mm, temperature programming: 60 ^{o}C (2 min), 20 ^{o}C/min to 280 ^{o}C, 280 ^{o}C (30 min); detector: 280 ^{o}C) (70 ev, EI) *m/z* (%) for (*S*,*E*)-**6a**: t_R (major) = 5.83 min: 210 (M^+, 2.31), 111 (100); for (*R*,*Z*)-**6a**: t_R (minor) = 5.76 min: 210 (M^+, 0.75), 111 (100). HRMS calcd for $C_{13}H_{22}O_2$ [M^+]: 210.1620, found: 210.1624.

2. Synthesis of (*S*,*E*)-5-(1-hexenyl)dihydro-2(3*H*)-furanone (*S*,*E*)-**6b** (zj-6-074)

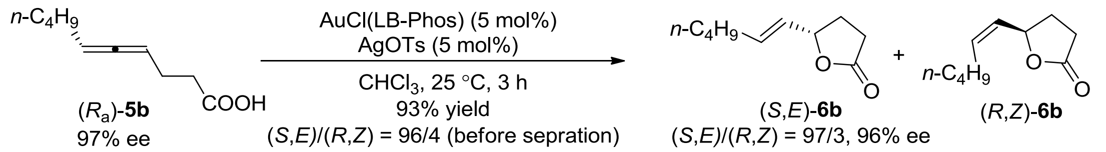

Following **Typical Procedure VI**, the reaction of AgOTs (0.0142 g, 0.05 mmol, 98%), Au(LB-Phos)Cl (0.0299 g, 0.05 mmol), $CHCl_3$ (5 mL), and (*R*$_a$)-**5b** (0.1680 g, 1.0 mmol)/$CHCl_3$ (5 mL) for 3.0 h afforded (*S*,*E*)-**6b**[10] (0.1563 g, 93%, (*S*,*E*)/(*R*,*Z*) = 97/3 determined by ^{1}H NMR) [eluent: petroleum ether (60-90 °C)/ethyl acetate = 10/1 (550 mL) to 8/1 (540 mL)] ((*S*,*E*)/(*R*,*Z*) = 96/4 determined by ^{1}H NMR of crude product) as a liquid with pleasant flavor: 96% ee (HPLC conditions: Chiralcel OJ-H column, *n*-hexane/*i*-PrOH = 200/1, 1.0 mL/min, λ = 214 nm, t_R (major) = 29.0 min, t_R (minor) = 26.3 min); $[\alpha]_D^{20}$ = +36.3 (c = 1.02, $CHCl_3$); ^{1}H NMR (300 MHz, $CDCl_3$) δ 5.81 (dtd, J_1 = 15.3 Hz, J_2 = 7.2 Hz, J_3 = 0.9 Hz, 1H, =CH), 5.50 (ddt, J_1 = 15.5 Hz, J_2 = 7.1 Hz, J_3 = 1.4 Hz, 1H, =CH), 4.90 (q, *J* = 7.1 Hz, 1H, CH), 2.60-2.49 (m, 2H, CH_2), 2.46-2.31 (m, 1H, one proton from CH_2), 2.12-1.90 (m, 3H, CH_2 + one proton from CH_2), 1.45-1.24 (m, 4H, CH_2 × 2), 0.90 (t, *J* = 7.1 Hz, 3H, CH_3); the following signals are discernible for (*R*,*Z*)-**6b**: δ 5.72-5.62 (m, 1H, =CH), 5.31-5.21 (m, 1H, CH); ^{13}C NMR (75 MHz, $CDCl_3$) δ 176.9, 142.0, 124.4, 81.0, 30.4, 28.6, 28.5, 21. 6; IR (neat) ν (cm^{-1}) 2957, 2929, 2872, 2859, 1778, 1673, 1460, 1423, 1379, 1328, 1295, 1217, 1178, 1123, 1054, 1006; GC-MS (GC condition: injector: 280 °C; column: DB5 column 30 m × 0.25 mm, temperature programming: 60 °C (2 min), 20 °C/min to 280 °C, 280 °C (30 min); detector: 280 °C) (70 ev, EI) *m/z* (%) for (*S*,*E*)-**6b**: t_R (major) = 4.47 min: 168 (M^+, 1.61), 111 (100); for (*R*,*Z*)-**6b** t_R (minor) = 4.42 min: 168 (M^+, 1.83), 111 (100).

3. Synthesis of (*S*,*E*)-5-(1-tridecenyl)dihydro-2(3*H*)-furanone (*S*,*E*)-**6c** (zj-4-114)

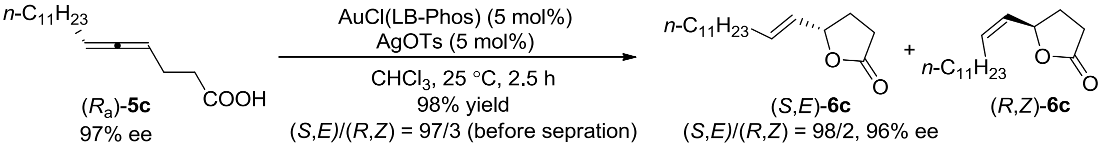

Following **Typical Procedure VI**, the reaction of AgOTs (0.0143 g, 0.05 mmol, 98%), Au(LB-Phos)Cl (0.0300 g, 0.05 mmol), $CHCl_3$ (5 mL), and (*R*$_a$)-**5c** (0.2663 g,

1.0 mmol)/$CHCl_3$ (5 mL) for 2.5 h afforded (*S*,*E*)-**6c** (0.2604 g, 98%, (*S*,*E*)/(*R*,*Z*) = 98/2 determined by ^{1}H NMR) [eluent: petroleum ether (30-60 °C)/ethyl acetate = 20/1 (300 mL) to 10/1 (500 mL)] ((*S*,*E*)/(*R*,*Z*) = 97/3 determined by ^{1}H NMR of crude product) as an oil with pleasant flavor: 96% ee (HPLC conditions: Chiralcel AS-H column, *n*-hexane/*i*-PrOH = 95:5, 1.0 mL/min, λ = 214 nm, t_R (major) = 7.3 min, t_R (minor) = 6.7 min; $[\alpha]_D^{20}$ = +23.0 (*c* = 1.005, $CHCl_3$); ^{1}H NMR (300 MHz, $CDCl_3$) δ 5.81 (dt, J_1 = 15.3 Hz, J_2 = 7.1 Hz, 1H, =CH), 5.49 (dd, J_1 = 15.5 Hz, J_2 = 7.1 Hz, 1H, =CH), 4.89 (q, *J* = 7.2 Hz, 1H, CH), 2.59-2.49 (m, 2H, CH_2), 2.44-2.31 (m, 1H, one proton from CH_2), 2.13-1.89 (m, 3H, one proton from CH_2 + CH_2), 1.46-1.20 (m, 18H, CH_2 × 8), 0.88 (t, *J* = 6.6 Hz, 3H, CH_3); the following signals are discernible for (*R*,*Z*)-**6c**: δ 5.72-5.62 (m, 1H, =CH), 5.30-5.20 (m, 1H, CH); ^{13}C NMR (75 MHz, $CDCl_3$) δ 177.0, 135.6, 127.2, 81.1, 32.0, 31.8, 29.53, 29.51, 29.45, 29.3, 29.2, 29.0, 28.73, 28.66, 28.6, 22.6, 14.0; IR (neat) ν (cm^{-1}) 2925, 2854, 1779, 1672, 1461, 1422, 1378, 1327, 1300, 1216, 1176, 1123, 1010; GC-MS (GC condition: injector: 280 °C; column: DB5 column 30 m × 0.25 mm, temperature programming: 60 °C (2 min), 20 °C/min to 280 °C, 280 °C (30 min); detector: 280 °C) (70 ev, EI) *m/z* (%) for (*S*,*E*)-**6c**: t_R (major) = 4.9 min: 266 (M^+, 1.24), 111 (100); for (*R*,*Z*)-**6c** t_R (minor) = 4.8 min): 266 (M^+, 2.32), 111 (100). HRMS calcd for $C_{17}H_{30}O_2$ [M^+]: 266.2246, found: 266.2250.

4. Synthesis of (*S*,*E*)-5-(3-methyl-1-butenyl)dihydro-2(3*H*)-furanone (*S*,*E*)-**6d** (zj-8-058)

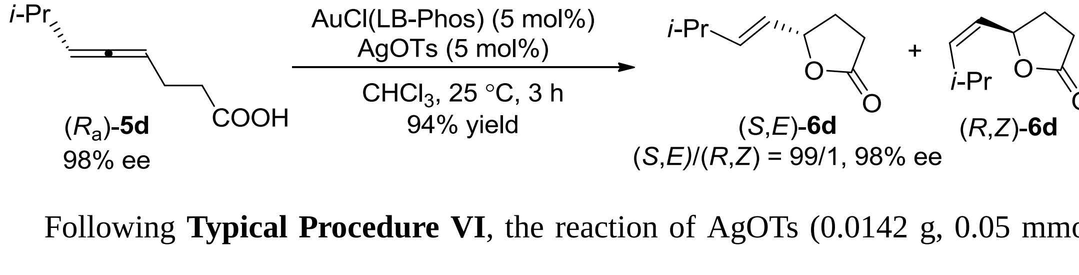

Following **Typical Procedure VI**, the reaction of AgOTs (0.0142 g, 0.05 mmol, 98%), Au(LB-Phos)Cl (0.0300 g, 0.05 mmol), $CHCl_3$ (5 mL), and (R_a)-**5d** (0.1541 g, 1.0 mmol)/$CHCl_3$ (5 mL) for 3.0 h afforded (*S*,*E*)-**6d** (0.1449 g, 94%, (*S*,*E*)/(*R*,*Z*) = 99/1 determined by quantitative ^{13}C NMR analysis) [eluent: petroleum ether (60-90

°C)/ethyl acetate = 15/1 (450 mL) to 10/1 (550 mL)] as a liquid with pleasant flavor: 98% ee (HPLC conditions: Chiralcel AS-H column, *n*-hexane/*i*-PrOH = 98/2, 1.0 mL/min, λ = 214 nm, t_R (major) = 23.6 min, t_R (minor) = 21.3 min); $[\alpha]_D^{20}$ = +37.8 (c = 1.03, $CHCl_3$); ^1H NMR (300 MHz, $CDCl_3$) δ 5.79 (ddd, J_1 = 15.5 Hz, J_2 = 6.5 Hz, J_3 = 0.9 Hz, 1H, =CH), 5.50 (ddd, J_1 = 15.6 Hz, J_2 = 7.2 Hz, J_3 = 1.4 Hz, 1H, =CH), 4.90 (q, *J* = 7.2 Hz, 1H, CH), 2.62-2.50 (m, 2H, CH_2), 2.47-2.25 (m, 2H, CH + one proton from CH_2), 2.07-1.91 (m, 1H, one proton from CH_2), 1.01 (d, *J* = 6.9 Hz, 6H, CH_3 × 2); ^{13}C NMR (75 MHz, $CDCl_3$) δ 176.9, 142.0, 124.4, 81.0, 30.4, 28.6, 28.5, 21.6; IR (neat) ν (cm^{-1}) 2960, 2927, 2870, 1777, 1463, 1421, 1381, 1327, 1296, 1217, 1176, 1132, 1077, 1012; GC-MS (GC condition: injector: 280 °C; column: DB5 column 30 m × 0.25 mm, temperature programming: 60 °C (2 min), 20 °C/min to 280 °C, 280 °C (30 min); detector: 280 °C) (70 ev, EI) *m/z* (%) for (*S*,*E*)-**6d**: t_R (major) = 4.06 min: 154 (M^+, 6.86), 111 (100); for (*R*,*Z*)-**6d** t_R (minor) = 3.95 min: 154 (M^+, 14.95), 111 (100); HRMS calcd for $C_9H_{14}O_2$ [M^+]: 154.0994, found: 154.0996.

5. Synthesis of (*S*,*E*)-5-(2-cyclohexylvinyl)dihydro-2(3*H*)-furanone (*S*,*E*)-**6e** (zj-4-008)

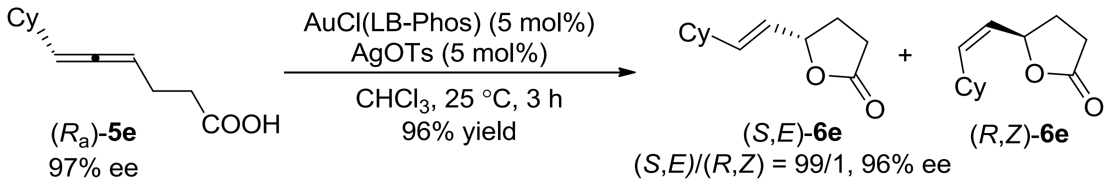

Following **Typical Procedure VI**, the reaction of AgOTs (0.0142 g, 0.05 mmol), Au(LB-Phos)Cl (0.0299 g, 0.05 mmol), $CHCl_3$ (5 mL), and (R_a)-**5e** (0.1938 g, 1.0 mmol)/$CHCl_3$ (5 mL) for 3 h afforded (*S*,*E*)-**6e** (0.1867 g, 96%, (*S*,*E*)/(*R*,*Z*) = 99/1 determined by quantitative ^{13}C NMR analysis) [eluent: petroleum ether (30-60 °C)/ethyl acetate = 8/1 (500 mL)] as an oil with pleasant flavor: 96% ee (HPLC conditions: Chiralcel OJ-H column, *n*-hexane/*i*-PrOH = 95:5, 1.0 mL/min, λ = 214 nm, t_R (major) = 17.1 min, t_R (minor) = 13.2 min; $[\alpha]_D^{20}$ = +29.9 (*c* = 1.005, $CHCl_3$); ^1H NMR (300 MHz, $CDCl_3$) δ 5.75 (ddd, J_1 = 15.3 Hz, J_2 = 6.6 Hz, J_3 = 0.75 Hz, 1H,

=CH), 5.44 (ddd, J_1 = 15.6 Hz, J_2 = 7.2 Hz, J_3 = 1.2 Hz, 1H, =CH), 4.89 (q, J = 7.1 Hz, 1H, CH), 2.60-2.46 (m, 2H, CH_2), 2.46-2.31 (m, 1H, CH), 2.06-1.90 (m, 2H, CH_2), 1.79-1.58 (m, 5H, one proton from CH_2 + CH_2 × 2), 1.36-0.98 (m, 5H, one proton from CH_2 + CH_2 × 2); ^{13}C NMR (75 MHz, $CDCl_3$) δ 176.6, 140.3, 124.7, 80.8, 39.6, 31.89, 31.85, 28.3, 28.2, 25.5, 25.3; IR (neat) ν (cm^{-1}) 2924, 2851, 1776, 1669, 1449, 1421, 1327, 1291, 1216, 1177, 1126, 1010; GC-MS (GC condition: injector: 280 °C; column: DB5 column 30 m × 0.25 mm, temperature programming: 60 °C (2 min), 20 °C/min to 280 °C, 280 °C (30 min); detector: 280 °C) (70 ev, EI) *m/z* (%) for (*S*,*E*)-**6e**: t_R (major) = 5.22 min: 194 (M$^+$, 5.91), 94 (100); for (*R*,*Z*)-**6e** t_R (minor) = 5.08 min): 194 (M$^+$, 11.78), 94 (100). HRMS calcd for $C_{12}H_{18}O_2$ [M$^+$]: 194.1307, found: 194.1307.

6. Synthesis of (*S*,*E*)-5-(3-phenyl-1-propenyl)dihydro-2(3*H*)-furanone (*S*,*E*)-**6f** (zj-4-175)

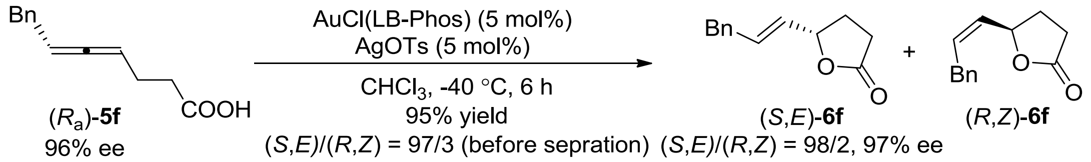

To a dry Schlenk tube were added AgOTs (0.0142 g, 0.05 mmol, weighed in a glove box, 98%), Au(LB-Phos)Cl (0.0299 g, 0.05 mmol), and $CHCl_3$ (5 mL) under nitrogen atmosphere sequentially. After being stirred at room temperature for 15 min, the resulting mixture was cooled down to -40 °C and then (R_a)-**5f** (0.2021 g, 1.0 mmol) and $CHCl_3$ (5 mL) were added. After being continuously stirred at -40 °C for 6 h, the reaction was complete as monitored by TLC. The resulting mixture was warmed up to room temperature. Filtration through a short column of silica gel [eluent: Et_2O (20 mL × 3) at -30°C] and evaporation afforded a crude mixture of (*S*,*E*)-**6f** and (*R*,*Z*)-**6f** ((*S*,*E*)/(*R*,*Z*) = 97/3, as determined by ^{1}H NMR analysis). Column chromatography [eluent: petroleum ether (60-90 °C)/ethyl acetate = 10/1 (330 mL) to 5/1 (450 mL) at -30 °C] on silica gel afforded (*S*,*E*)-**6f** (0.1912 g, 95%, (*S*,*E*)/(*R*,*Z*) = 98/2 determined by ^{1}H NMR) as an oil with pleasant flavor: 97% ee (HPLC conditions: Chiralcel IB

column, *n*-hexane/*i*-PrOH = 98:2, 1.0 mL/min, λ = 214 nm, t_R(major) = 38.9 min, t_R(minor) = 37.6 min; $[\alpha]_D^{20}$ = +34.1 (*c* = 1.005, $CHCl_3$); ^1H NMR (300 MHz, $CDCl_3$) δ 7.31-7.21 (m, 2H, ArH), 7.21-7.09 (m, 3H, ArH), 5.90 (dtd, J_1 = 15.3 Hz, J_2 = 6.8 Hz, J_3 = 1.0 Hz, 1H, =CH), 5.50 (ddt, J_1 = 15.5 Hz, J_2 = 7.1 Hz, J_3 = 1.4 Hz, 1H, =CH), 4.82 (q, *J* = 7.2 Hz, 1H, CH), 3.34 (d, *J* = 6.9 Hz, 2H, CH_2), 2.49-2.36 (m, 2H, CH_2), 2.34-2.18 (m, 1H, one proton from CH_2), 1.95-1.79 (m, 1H, one proton from CH_2); the following signals are discernible for (*R*,*Z*)-**6f**: δ 5.82-5.72 (m, 1H, =CH), 5.34-5.24 (m, 1H, CH); ^{13}C NMR (75 MHz, $CDCl_3$) δ 176.6, 138.8, 133.2, 128.5, 128.14, 128.07, 125.9, 80.2, 37.9, 28.2, 28.1; IR (neat) ν (cm^{-1}) 3084, 3061, 3027, 3001, 2941, 2917, 2840, 1770, 1671, 1602, 1495, 1453, 1422, 1380, 1328, 1290, 1217, 1176, 1121, 1075, 1029, 1008; GC-MS (GC condition: injector: 280 °C; column: DB5 column 30 m × 0.25 mm, temperature programming: 60 °C (2 min), 20 °C/min to 280 °C, 280 °C (30 min); detector: 280 °C) (70 ev, EI) *m/z* (%) for (*S*,*E*)-**6f**: t_R = 4.1 min: 202 (M^+, 19.17), 111 (100); HRMS calcd for $C_{13}H_{14}O_2$ [M^+]: 202.0994, found: 202.0995.

7. Synthesis of (*S*,*E*)-5-(4-phenyl-1-butenyl)dihydro-2(3*H*)-furanone (*S*,*E*)-**6g** (zj-4-146)

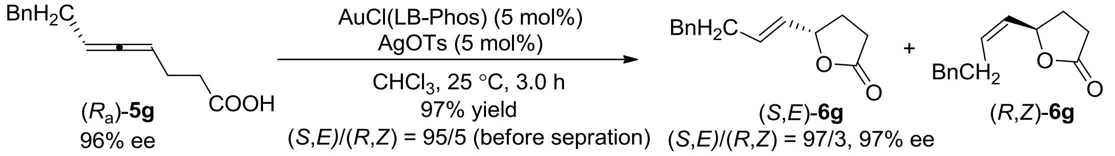

Following **Typical Procedure VI**, the reaction of AgOTs (0.0142 g, 0.05 mmol, 98%), Au(LB-Phos)Cl (0.0298 g, 0.05 mmol), $CHCl_3$ (5 mL), and (R_a)-**5g** (0.2156 g, 1.0 mmol)/$CHCl_3$ (5 mL) for 3.0 h afforded (*S*,*E*)-**6g** (0.2101 g, 97%, (*S*,*E*)/(*R*,*Z*) = 97/3 determined by ^1H NMR) [eluent: petroleum ether (60-90 °C)/ethyl acetate = 10/1 (440 mL) to 8/1 (540 mL)] ((*S*,*E*)/(*R*,*Z*) = 95/5 determined by ^1H NMR of crude product) as an oil with pleasant flavor: 97% ee (HPLC conditions: Chiralcel OD-H column, *n*-hexane/*i*-PrOH = 90:10, 0.7 mL/min, λ = 214 nm, t_R (major) = 27.7 min, t_R (minor) = 29.9 min; $[\alpha]_D^{20}$ = +25.4 (*c* = 1.01, $CHCl_3$); ^1H NMR (300 MHz, $CDCl_3$) δ 7.31-7.22 (m, 2H, ArH), 7.21-7.10 (m, 3H, ArH), 5.81 (dtd, J_1 = 15.3 Hz, J_2 = 6.8 Hz,

J_3 = 1.1 Hz, 1H, =CH), 5.47 (ddt, J_1 = 15.3 Hz, J_2 = 7.2 Hz, J_3 = 1.4 Hz, 1H, =CH), 4.83 (q, J = 7.2 Hz, 1H, CH), 2.74-2.65 (m, 2H, CH_2), 2.51-2.42 (m, 2H, CH_2), 2.42-2.22 (m, 3H, one proton from CH_2 + CH_2), 1.95-1.80 (m, 1H, one proton from CH_2); the following signals are discernible for (*R*,*Z*)-**6g**: δ 5.72-5.62 (m, 1H, =CH), 5.11-5.01 (m, 1H, CH); ^{13}C NMR (75 MHz, $CDCl_3$) δ 176.8, 141.0, 133.9, 128.2, 128.1, 128.0, 125.7, 80.6, 34.9, 33.6, 28.4, 28.3; IR (neat) ν (cm^{-1}) 3081, 3056, 3026, 2998, 2926, 2855, 1774, 1671, 1602, 1496, 1454, 1421, 1379, 1327, 1216, 1176, 1121, 1009; GC-MS (GC condition: injector: 280 ^{o}C; column: DB5 column 30 m × 0.25 mm, temperature programming: 60 ^{o}C (2 min), 20 ^{o}C/min to 280 ^{o}C, 280 ^{o}C (30 min); detector: 280 ^{o}C) (70 ev, EI) *m/z* (%) for (*S*,*E*)-**6g**: t_R = 4.3 min: 216 (M^+, 2.67), 91 (100) ; Elemental analysis calcd (%) for $C_{14}H_{16}O_2$: C, 77.75; H, 7.46; Found: C, 77.60; H, 7.47.

8. Synthesis of (*S*,*E*)-5-(dodeca-1,11-dien-1-yl)dihydro-2(3*H*)-furanone (*S*,*E*)-**6h** (zj-5-083)

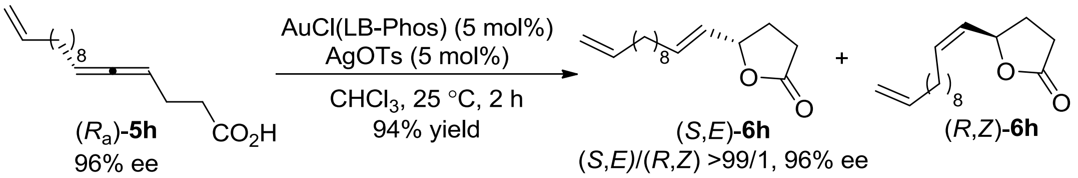

Following **Typical Procedure VI**, the reaction of AgOTs (0.0142 mg, 0.05 mmol, 98%), Au(LB-Phos)Cl (0.0299 mg, 0.05 mmol), $CHCl_3$ (5 mL), and (R_a)-**6h** (0.2504 g, 1.0 mmol)/$CHCl_3$ (5 mL) for 2.0 h afforded (*S*,*E*)-**6h** (0.2356 g, 94%, (*S*,*E*)/(*R*,*Z*) > 99/1 determined by ^{1}H NMR) [eluent: petroleum ether (60-90 ^{o}C)/ethyl acetate = 10/1 (440 mL) to 8/1 (450 mL)] as an oil: 96% ee (SFC (supercritical fluid chromatogram): Chiralcel AD-H column, *n*-hexane/*i*-PrOH = 98/2, 0.7 mL/min, λ = 214 nm, t_R (major) = 12.9 min, t_R (minor) = 12.1 min); $[\alpha]_D^{20}$ = +26.0 (c = 0.99, $CHCl_3$); ^{1}H NMR (300 MHz, $CDCl_3$) δ 5.90-5.69 (m, 2H, =CH), 5.49 (dd, J_1 = 15.3 Hz, J_2 = 7.2 Hz, 1H, =CH), 5.05-4.81 (m, 3H, =CH_2 + CH), 2.57-2.45 (m, 2H, CH_2), 2.43-2.30 (m, 1H, one proton from CH_2), 2.11-1.88 (m, 5H, one proton from CH_2 + CH_2 × 2), 1.45-1.17 (m, 12H, CH_2 × 6); ^{13}C NMR (75 MHz, $CDCl_3$) δ 177.0, 139.1, 135.6, 127.3, 114.1, 81.1,

33.7, 32.0, 29.3, 29.0, 28.81, 28.76, 28.68, 28.65; IR (neat) ν (cm^{-1}) 3075, 2976, 2925, 2854, 1778, 1673, 1640, 1459, 1436, 1422, 1374, 1327, 1300, 1216, 1176, 1123, 1008; GC-MS (GC condition: injector: 280 °C; column: DB5 column 30 m × 0.25 mm, temperature programming: 60 °C (2 min), 20 °C/min to 280 °C, 280 °C (30 min); detector: 280 °C, (70 ev, EI) *m/z* (%) for (*S*,*E*)-**6h**: t_R = 4.60 min: 250 (M^+, 0.42), 41 (100); Elemental analysis calcd (%) for $C_{16}H_{26}O_2$: C, 76.75; H, 10.47; Found: C, 76.73; H, 10.77.

9. Synthesis of (*S*,*E*)-5-(10-(*tert*-butyldimethylsilyl)-1-decen-9-yn-1-yl)dihydro-2(3*H*)-furanone (*S*,*E*)-**6i** (zj-6-188)

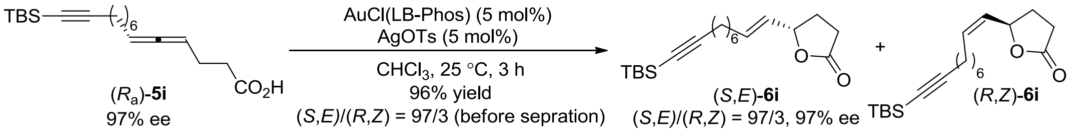

Following **Typical Procedure VI**, the reaction of AgOTs (0.0142 g, 0.05 mmol), Au(LB-Phos)Cl (0.0300 g, 0.05 mmol), $CHCl_3$ (5 mL) and (R_a)-**5i** (0.3330 g, 1.0 mmol)/$CHCl_3$ (5 mL) for 3.0 h afforded (*S*,*E*)-**6i** (0.3206 g, 96%, (*S*,*E*)/(*R*,*Z*) = 97/3 determined by ^{1}H NMR) [eluent: petroleum ether (60-90 °C)/ethyl acetate = 15/1 (480 mL) to 10/1 (850 mL)] ((*S*,*E*)/(*R*,*Z*) = 97/3 determined by ^{1}H NMR of crude product) as an oil: 97% ee (HPLC conditions: Chiralcel OJ-H column, *n*-hexane/*i*-PrOH = 100/1, 0.3 mL/min, λ = 214 nm, t_R (major) = 29.5 min, t_R (minor) = 28.5 min); $[\alpha]_D^{20}$ = +17.6 (c = 1.00, $CHCl_3$); ^{1}H NMR (300 MHz, $CDCl_3$) δ 5.78 (dtd, J_1 = 15.3 Hz, J_2 = 7.2 Hz, J_3 = 0.8 Hz, 1H, =CH), 5.47 (ddt, J_1 = 15.3 Hz, J_2 = 6.9 Hz, J_3 = 1.4 Hz, 1H, =CH), 4.87 (q, *J* = 7.1 Hz, 1H, CH), 2.57-2.47 (m, 2H, CH_2), 2.44-2.28 (m, 1H, one proton from CH_2), 2.20 (t, *J* = 6.9 Hz, 2H, CH_2), 2.10-1.87 (m, 3H, CH_2 + one proton from CH_2), 1.55-1.22 (m, 8H, CH_2 × 4), 0.90 (s, 9H, CH_3 × 3), 0.05 (s, 6H, CH_3 × 2); the following signals are discernible for (*R*,*Z*)-**6i**: δ 5.69-5.60 (m, 1H, =CH), 5.27-5.18 (m, 1H, CH); ^{13}C NMR (75 MHz, $CDCl_3$) δ 176.9, 135.4, 127.4, 107.9, 82.3, 81.0, 31.9, 28.7, 28.6, 28.5, 28.39, 28.36, 28.3, 26.0, 19.6, 16.4, -4.6; IR (neat) ν (cm^{-1}) 2929, 2856, 2172, 1776, 1673, 1461, 1361, 1326, 1249, 1174, 1008; GC-MS (GC condition: injector: 280 °C; column: DB5 column 30 m × 0.25 mm, temperature

programming: 60 °C (2 min), 20 °C/min to 280 °C, 280 °C (30 min); detector: 280 °C) (70 ev, EI) *m/z* (%) for (*S*,*E*)-**6i**: t_R (major) = 5.41 min, *m/z* (%) 277 (M^+ - *t*-Bu, 10.23), 75 (100); for (*R*,*Z*)-**6i**: t_R (minor) = 5.36 min, *m/z* (%) 277 (M^+ - *t*-Bu, 8.13), 75 (100); Elemental analysis calcd (%) for $C_{20}H_{34}O_2Si$: C, 71.80; H, 10.24; Found: C, 71.57; H, 10.18.

10. Synthesis of (*R*,*E*)-5-(1-decenyl)dihydro-2(3*H*)-furanone (*R*,*E*)-**6j** (zj-5-014)

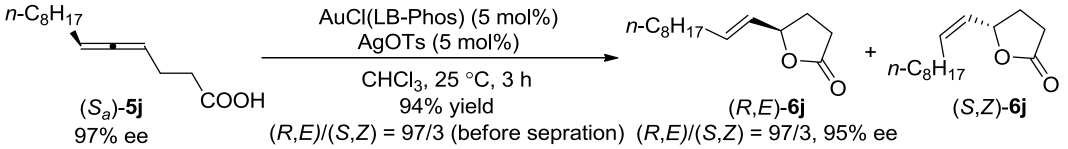

Following **Typical Procedure VI**, the reaction of AgOTs (0.0142 g, 0.05 mmol), AuCl(LB-Phos) (0.0299 g, 0.05 mmol), $CHCl_3$ (5 mL), and (S_a)-**5j** (0.2239 g, 1.0 mmol)/$CHCl_3$ (5 mL) (for synthesis of compound (S_a)-**5j**, see page 68) for 3.0 h afforded (*R*,*E*)-**6j**[11] (0.2094 g, 94%, (*R*,*E*)/(*S*,*Z*) = 97/3 determined by ^{1}H NMR) [eluent: petroleum ether (60-90 °C)/ethyl acetate = 10/1 (220 mL) to 8/1 (450 mL)] ((*R*,*E*)/(*S*,*Z*) = 97/3 determined by ^{1}H NMR of crude product) as an oil with pleasant flavor: 95% ee (HPLC conditions: Chiralcel OJ-H column, *n*-hexane/*i*-PrOH = 100/1, 1.0 mL/min, λ = 214 nm, t_R (major) = 12.9 min, t_R (minor) = 16.2 min); $[\alpha]_D^{20}$ = -27.4 (c = 1.00, $CHCl_3$); (Lit.[10] $[\alpha]_D^{26}$ = -31.2 (c = 2.237, $CHCl_3$)); ^{1}H NMR (300 MHz, $CDCl_3$) δ 5.81 (dtd, J_1 = 15.3 Hz, J_2 = 6.9 Hz, J_3 = 0.8 Hz, 1H, =CH), 5.49 (ddt, J_1 = 15.2 Hz, J_2 = 7.1 Hz, J_3 = 1.5 Hz, 1H, =CH), 4.89 (q, *J* = 7.1 Hz, 1H, CH), 2.58-2.49 (m, 2H, CH_2), 2.45-2.31 (m, 1H, one proton from CH_2), 2.12-1.90 (m, 3H, CH_2 + one proton from CH_2), 1.46-1.18 (m, 12H, CH_2 × 6), 0.88 (t, *J* = 6.9 Hz, 3H, CH_3); the following signals are discernible for (*S*,*Z*)-**6j**: δ 5.72-5.61 (m, 1H, =CH), 5.30-5.20 (m, 1H, CH); ^{13}C NMR (75 MHz, $CDCl_3$) δ 176.9, 135.4, 127.3, 80.9, 31.9, 31.7, 29.2, 29.0, 28.9, 28.7, 28.6, 28.5, 22.5, 13.9; IR (neat) ν (cm^{-1}) 2955, 2926, 2854, 1778, 1673, 1460, 1422, 1378, 1327, 1296, 1216, 1176, 1123, 1011; GC-MS (GC condition: injector: 280 °C; column: DB5 column 30 m × 0.25 mm, temperature programming: 60 °C (2 min), 20 °C/min to 280 °C, 280 °C (30 min); detector: 280 °C) (70 ev, EI) *m/z* (%) for (*R*,*E*)-**6j**: t_R (major) = 4.03 min: 224 (M^+, 0.72), 111 (100); for (*S*,*Z*)-**6j** t_R

(minor) = 3.99 min: 224 (M^+, 1.18), 111 (100).

11. Gram scale synthesis of (*S*,*E*)-**6b** (zj-6-160)

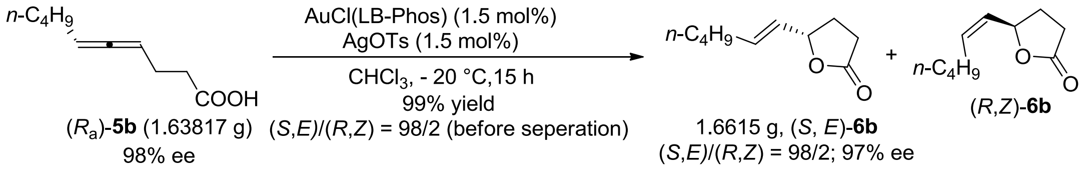

To a dry Schlenk tube were added AgOTs (0.0426 g, 0.15 mmol, weighed in glove box, 98%), Au(LB-Phos)Cl (0.0896 g, 0.15 mmol), and $CHCl_3$ (30 mL) under nitrogen atmosphere sequentially. After stirring at room temperature for 15 min, the reaction was cooled down to -20 °C and then (R_a)-**5b** (1.6817 g, 10.0 mmol) and $CHCl_3$ (10 mL) (for synthesis of compound (R_a)-**5b**, see page 74) were added. After being continuously stirred at –20 °C for 15 h, the reaction was complete as monitored by TLC. The resulting mixture was warmed up to room temperature. Filtration through a short column of silica gel [eluent: Et_2O (25 mL ×3)] and evaporation afforded a crude mixture of (*S*,*E*)-**6b** and (*R*,*Z*)-**6b** ((*S*,*E*)/(*R*,*Z*) = 98/2 determined by ^{1}H NMR analysis). Column chromatography on silica gel [(eluent: petroleum ether (60-90 °C)/ethyl acetate = 15/1 (500 mL) to 10/1 (550 mL)] afforded (*S*,*E*)-**6b** (1.6615 g, 99%, (*S*,*E*)/(*R*,*Z*) = 98/2 determined by ^{1}H NMR) as a liquid with pleasant flavor: 97% ee (HPLC conditions: Chiralcel OJ-H column, *n*-hexane/*i*-PrOH = 200/1, 1.0 mL/min, λ = 214 nm, t_R (major) = 26.4 min, t_R (minor) = 23.7 min); $[\alpha]_D^{20}$ = +37.1 (c = 1.00, $CHCl_3$); ^{1}H NMR (300 MHz, $CDCl_3$) δ 5.81 (dtd, J_1 = 15.3 Hz, J_2 = 6.8 Hz, J_3 = 1.0 Hz, 1H, =CH), 5.49 (ddt, J_1 = 15.3 Hz, J_2 = 7.2 Hz, J_3 = 1.5 Hz, 1H, =CH), 4.90 (q, *J* = 7.2 Hz, 1H, CH), 2.60-2.49 (m, 2H, CH_2), 2.46-2.31 (m, 1H, one proton from CH_2), 2.12-1.90 (m, 3H, CH_2 + one proton from CH_2), 1.46-1.24 (m, 4H, CH_2 × 2), 0.90 (t, *J* = 7.2 Hz, 3H, CH_3); the following signals are discernible for (*R*,*Z*)-**6b**: δ 5.70-5.62 (m, 1H, =CH), 5.31-5.21 (m, 1H, CH); ^{13}C NMR (75 MHz, $CDCl_3$) δ 176.9, 135.4, 127.3, 81.0, 31.6, 30.7, 28.7, 28.5, 21.9, 13.7.

12. Synthesis of (*R*,*E*)-5-(1-octenyl)dihydro-2(3*H*)-furanone (*R*,*E*)-**6m** (zj-9-053)

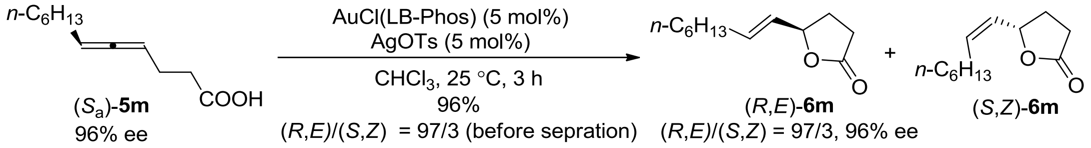

Following **Typical Procedure VI**, the reaction of AgOTs (0.0142 g, 0.05 mmol, 98%), Au(LB-Phos)Cl (0.0300 g, 0.05 mmol), $CHCl_3$ (5 mL), and (*S*$_a$)-**5m** (0.1964 g, 1.0 mmol)/$CHCl_3$ (5 mL) for 3.0 h afforded (*R*,*E*)-**6m** (0.1891 g, 96%, (*R*,*E*)/(*S*,*Z*) = 97/3 determined by ^{1}H NMR) [eluent: petroleum ether (60-90 ^{o}C)/ethyl acetate = 10/1 (550 mL) to 7/1 (480 mL)] ((*R*,*E*)/(*S*,*Z*) = 97/3 determined by ^{1}H NMR of crude product) as a liquid with pleasant flavor: 96% ee (HPLC conditions: Chiralcel OJ-H column, *n*-hexane/*i*-PrOH = 97/3, 1.0 mL/min, λ = 214 nm, t_R (major) = 12.5 min, t_R (minor) = 14.5 min); $[\alpha]_D^{20}$ = -32.6 (c = 0.995, $CHCl_3$); ^{1}H NMR (300 MHz, $CDCl_3$) δ 5.81 (dt, J_1 = 15.3 Hz, J_2 = 7.1 Hz, 1H, =CH), 5.49 (ddt, J_1 = 15.3 Hz, J_2 = 7.2 Hz, J_3 = 1.2 Hz, 1H, =CH), 4.90 (q, *J* = 7.2 Hz, 1H, CH), 2.61-2.48 (m, 2H, CH_2), 2.46-2.32 (m, 1H, one proton from CH_2), 2.15-1.90 (m, 3H, CH_2 + one proton from CH_2), 1.46-1.16 (m, 8H, CH_2 × 4), 0.88 (t, *J* = 6.6 Hz, 3H, CH_3); the following signals are discernible for (*S*,*Z*)-**6m**: δ 5.71-5.61 (m, 1H, =CH), 5.32-5.22 (m, 1H, CH); ^{13}C NMR (75 MHz, $CDCl_3$) δ 176.8, 135.3, 127.2, 80.9, 31.8, 31.3, 28.5, 28.44, 28.41, 22.2, 13.7; IR (neat) ν (cm^{-1}) 2956, 2927, 2856, 1777, 1673, 1459, 1420, 1379, 1327, 1216, 1179, 1132, 1076, 1009; GC-MS (GC condition: injector: 280 ^{o}C; column: DB5 column 30 m × 0.25 mm, temperature programming: 60 ^{o}C (2 min), 20 ^{o}C/min to 280 ^{o}C, 280 ^{o}C (30 min); detector: 280 ^{o}C) (70 ev, EI) *m/z* (%) for (*R*,*E*)-**6m**: t_R (major) = 6.65 min: 196 (M^+, 0.88), 111 (100); for (*S*,*Z*)-**6m**: t_R (minor) = 6.57 min: 196 (M^+, 1.68), 111 (100). HRMS calcd for $C_{12}H_{20}O_2$ [M^+]: 196.1463, found: 196.1466.

Synthesis of naturally occurring xestospongienes

1. Synthesis of racemic xestospongiene

Synthesis of 9-((*tert*-butyldimethylsilyl)oxy)non-1-yn-3-ol[12] *rac*-**8** (zj-7-083)

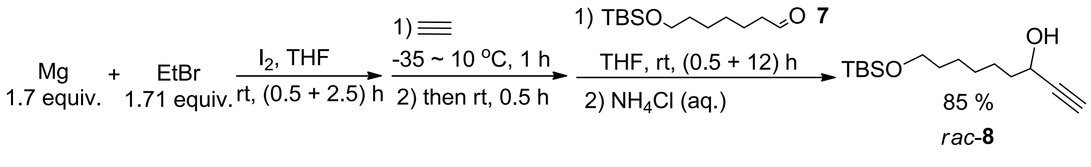

To a flame-dried three-neck flask equipped with a reflux condenser containing Mg turnings (2.6545 g, 110.5 mmol) was added THF (110 mL) under nitrogen atmosphere. After the reaction was initiated with a grain of I_2 and a little amount of EtBr, EtBr [8.3 mL (including the EtBr used above for initiation), d = 1.46 g cm^{-3}, 12.118 g, 111.17 mmol] was then added dropwise within 30 min. After the addition was complete, the resulting mixture was stirred for 2.5 h at room temperature, and then a stream of ethyne was bubbled through the resulting solution at -35 ^{o}C. The ethyne gas was introduced for 30 min at a rate that the temperature was kept under 10 ^{o}C using a dry ice-acetone bath. Then the gas of ethyne was introduced for an extra 30 min before removing the dry ice-acetone bath. The resulting mixture was warmed up to room temperature for 30 min and a solution of 7-((*tert*-butyldimethylsilyl)oxy)heptanal **7** (readily available from 1,7-heptanediol in 2 steps)[13] (15.8817 g, 65 mmol) in THF (25 mL) was added dropwise within 30 min. When the reaction was complete after 12 h as monitored by TLC, a saturated aqueous solution of NH_4Cl (30 mL) was added dropwise at rt. The organic layer was separated and the aqueous layer was extracted with Et_2O (50 mL × 3). The combined organic layer was washed with H_2O and brine and dried over anhydrous Na_2SO_4. After filtration and evaporation, the residue was purified by chromatography [eluent: petroleum ether (60-90 ^{o}C)/ethyl acetate = 100/1 (350 mL) to 50/1 (500 mL) to 15/1 (500 mL × 2)] on silica gel afforded 9-((*tert*-butyldimethylsilyl)oxy)non-1-yn-3-ol *rac*-**8** (14.8471 g, 85%) as a liquid: ^{1}H NMR (300 MHz, $CDCl_3$) δ 4.32 (t, *J* = 6.6 Hz, 1H, CH), 3.57 (t, *J* = 6.6 Hz, 2H, CH_2), 2.68 (s, 1H, OH), 2.42 (d, *J* = 2.1 Hz, 1H, ≡CH), 1.73-1.62 (m, 2H, CH_2), 1.54-1.38 (m, 4H, CH_2 × 2), 1.35-1.26 (m, 4H, CH_2 × 2), 0.86 (s, 9H, CH_3 × 3), 0.01 (s, 6H, CH_3 × 2); ^{13}C NMR (75 MHz, $CDCl_3$) δ 85.1, 72.7, 63.2, 62.0, 37.5, 32.6, 29.0, 25.9, 25.6, 24.9, 18.3, -5.3; IR (neat) ν (cm^{-1}) 3355, 3311, 2930, 2857, 2114, 1472, 1463, 1388, 1361, 1255, 1099, 1006; MS (ESI) *m/z* 271 $[M + H]^+$; HRMS (ESI) calcd. for $C_{15}H_{31}O_2Si^+$ $[M + H]^+$: 271.2088, found: 271.2080.

Synthesis of 7-methoxynon-8-yn-1-ol [14] *rac*-**9** (zj-7-106)

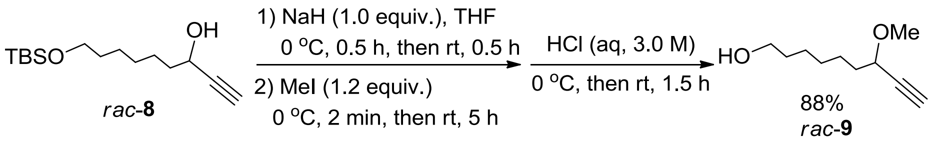

To a dry Schlenk flask were added **8** (2.9494 g, 11 mmol) and THF (33 mL) under nitrogen atmosphere. The solution was cooled to 0 ℃ with an ice bath, and NaH (0.4404 g, 60% dispersion in mineral oil, 11 mmol,) was added carefully. After the addition was complete, the cooling bath was removed and the resulting mixture was stirred for 30 min. Then MeI (0.83 mL, d = 2.28 g cm^{-3}, 1.8735 g, 13.2 mmol, 99%) was added dropwise at 0 ℃ within 2 min. The reaction was warmed up to room temperature again by removing the ice-bath. After stirring for 5 h, the reaction was complete as monitored by TLC and then 3 M HCl (5.5 mL) was added slowly at 0 ℃. The reaction was monitored by TLC again after 1.5 h to make sure that the TBS-group was removed completely. The mixture was quenched with water (11 mL) and then extracted with EtOAc (20 mL × 3). The combined organic layer was washed with brine and dried over anhydrous Na_2SO_4. After filtration and evaporation, the residue was purified by chromatography [eluent: petroleum ether (60-90 ^{o}C)/ethyl acetate = 10/1 (550 mL) to 5/1 (600 mL) to 3/1 (480 mL)] on silica gel to afford 7-methoxynon-8-yn-1-ol *rac*-**9** (1.6238 g, 88%) as a liquid: ^{1}H NMR (300 MHz, $CDCl_3$) δ 3.94 (td, J_1 = 6.5 Hz, J_2 = 2.0 Hz, 1H, CH), 3.63 (t, J = 6.6 Hz, 2H, CH_2), 3.41 (s, 3H, CH_3), 2.60-2.38 (bs, 1H, OH), 2.46 (d, J = 1.8 Hz, 1H, ≡CH), 1.81-1.66 (m, 2H, CH_2), 1.66-1.29 (m, 8H, CH_2 × 4); ^{13}C NMR (75 MHz, $CDCl_3$) δ 82.6, 73.7, 71.0, 62.7, 56.3, 35.3, 32.5, 29.0, 25.5, 25.0; IR (neat) ν (cm^{-1}) 3384, 3299, 2934, 2859, 2822, 2112, 1465, 1336, 1107, 1092, 1058; MS (ESI) *m/z* 171 $[M + H]^+$; HRMS (ESI) calcd. for $C_{10}H_{19}O_2^+$ $[M + H]^+$: 171.1380, found:171.1383.

Synthesis of 7-methoxynon-8-ynal[15] *rac*-**10** (zj-7-110)

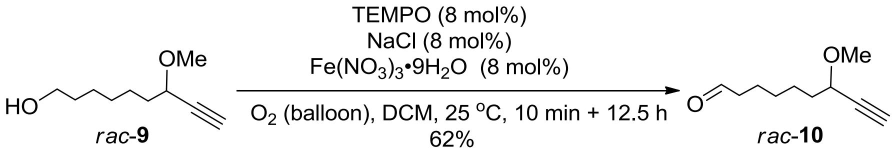

To a three-necked flask were added $Fe(NO_3)_3·9H_2O$ (0.2962 g, 0.72 mmol, 98%),

NaCl (0.0420 g, 0.72 mmol), TEMPO (0.1146 g, 0.72 mmol, 98%), and DCM (75 mL) subsequently. An oxygen balloon was equipped and then a solution of *rac*-**9** (1.5279 g, 9 mmol) in 15 mL of DCM was added dropwise via an addition funnel at 25 °C within 10 min in oxygen atmosphere from the balloon. The resulting mixture was stirred for 12.5 h until the reaction was complete as monitored by TLC. After filtration through a short column of silica gel [eluent: Et_2O (20 mL × 3)] and evaporation, the residue was purified by chromatography [eluent: petroleum ether (60-90 °C)/ethyl acetate = 30/1 (500 mL) to 20/1 (500 mL) to 10/1 (500 mL)] on silica gel to afford 7-methoxynon-8-ynal *rac*-**10** (0.9301 g, 62%) as a liquid: ^{1}H NMR (300 MHz, $CDCl_3$) δ 9.76 (t, J = 1.8 Hz, 1H, CHO), 3.93 (td, J_1 = 6.5 Hz, J_2 = 1.7 Hz, 1H, CH), 3.41 (s, 3H, CH_3), 2.40-2.41 (m, 3H, ≡CH + CH_2), 1.77-1.59 (m, 4H, CH_2 × 2), 1.55-1.43 (m, 2H, CH_2), 1.43-1.31 (m, 2H, CH_2); ^{13}C NMR (75 MHz, $CDCl_3$) δ 201.7, 81.7, 73.3, 70.0, 55.5, 42.9, 34.3, 27.9, 23.9, 21.0; IR (neat) ν (cm^{-1}) 3284, 2986, 2940, 2862, 2823, 2722, 2109, 1724, 1465, 1410, 1391, 1336, 1192, 1156, 1108, 1091; MS (ESI) *m/z* 169 $[M + H]^+$; HRMS (ESI) calcd. for $C_{10}H_{17}O_2^+$ $[M + H]^+$: 169.1223, found: 169.1220.

Synthesis of 1,1-dibromo-8-methoxydec-1-en-9-yne *rac*-**1c**[16] (zj-7-112)

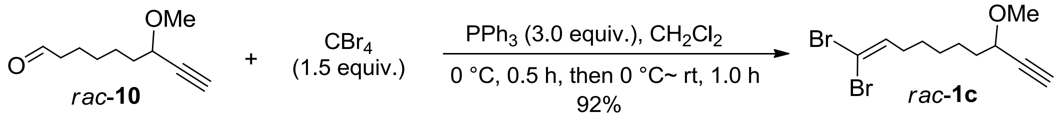

Typical Procedure VII: To a dry Schlenk flask were added aldehyde *rac*-**10** (0.8727 g, 5.2 mmol) and DCM (55 mL, dry) under nitrogen atmosphere. The resulting solution was cooled to 0 ℃ with an ice bath followed by the addition of CBr_4 (2.5884 g, 7.8 mmol). Then a solution of PPh_3 (4.1697 g, 15.6 mmol, 98%) in DCM (20 mL) was added dropwise at 0 ℃ within 0.5 h. The resulting mixture was warmed up to room temperature naturally. After 1 h the reaction was complete as monitored by TLC. Then the resulting mixture was diluted with petroleum ether (75 mL) forming white precipitate. The precipitate was filtrated off and washed with petroleum ether (15 mL × 3). After combining the filtrate and evaporation, the residue

was purified by chromatography [eluent: petroleum ether/ethyl acetate = 100/1 (400 mL) to 50/l (500 mL) to 30/l (200 mL)] on silica gel to afford *rac*-**1c** (1.5451 g, 92%) as a liquid: ^{1}H NMR (300 MHz, $CDCl_3$) δ 6.38 (t, J = 7.2 Hz, 1H, =CH), 3.93 (td, J_1 = 6.5 Hz, J_2 = 2.1 Hz, 1H, CH), 3.41 (s, 3H, CH_3), 2.45 (d, J = 2.1 Hz, 1H, ≡CH), 2.10 (q, J = 7.2 Hz, 2H, CH_2), 1.77-1.66 (m, 2H, CH_2), 1.53-1.27 (m, 6H, CH_2 × 3), ^{13}C NMR (75 MHz, $CDCl_3$) δ 138.6, 88.6, 82.6, 73.8, 70.9, 56.4, 35.3, 32.8, 28.6, 27.6, 24.7; IR (neat) ν (cm^{-1}) 3301, 2985, 2932, 2858, 2822, 2110, 1622, 1463, 1335, 1273, 1261, 1196, 1152, 1109; MS (EI) *m/z* (%) 325 {[M (^{81}Br^{81}Br) - H]$^+$, 0.035}, 323 {[M (^{79}Br^{81}Br) - H]$^+$, 0.069}, 321 {[M (^{79}Br^{79}Br) - H]$^+$, 0.036}, 201 {[M (^{81}Br^{81}Br) - $C_8H_{13}O$]$^+$, 5.37}, 199 {[M (^{79}Br^{81}Br) - $C_8H_{13}O$]$^+$, 12.31}, 197 {[M (^{79}Br^{79}Br) - $C_8H_{13}O$]$^+$, 5.62}, 69 (100); HRMS (EI) calcd for $C_{11}H_{15}{}^{79}Br^{79}BrO$ [M - H]$^+$: 320.9490, found: 320.9499.

Synthesis of methyl 14,14-dibromo-7-methoxy-4,5,13-tetradecatrienoate *rac*-**4ck** (zj-7-114)

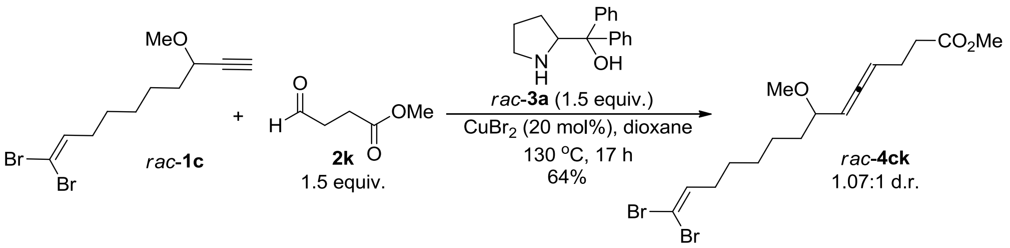

To a flame-dried Schlenk tube with a polytetrafluoroethylene plug were added $CuBr_2$ (0.1803 g, 0.8 mmol, 99%), *rac*-**3a** (1.5497 g, 6.0 mmol, 98%), *rac*-**1c** (1.2943 g, 4.0 mmol)/dioxane (6 mL), and methyl 4-oxobutanoate **2k**[17] (0.6965 g, 6 mmol)/dioxane (6 mL) sequentially under nitrogen atmosphere. The Schlenk tube was then sealed by screwing the polytetrafluoroethylene plug tightly. The reaction was complete after being stirred in an oil bath preheated at 130 °C for 17 h as monitored by TLC. The resulting mixture was cooled down to room temperature, diluted with Et_2O (50 mL), and washed with an aqueous solution of hydrochloric acid (10 mL × 3, 3 M). The organic layer was separated and the aqueous layer was extracted with Et_2O (10 mL). The combined organic layer was washed with brine and dried over anhydrous Na_2SO_4. After filtration and evaporation, the residue was purified by

chromatography [eluent: petroleum ether (60-90 ^{o}C)/ethyl acetate = 50/1 (500 mL) to 30/1 (500 mL) to 15/1 (600 mL)] on silica gel to afford *rac*-**4ck** (1.0789 g, 64%, d.r. = 1.07 : 1 determined by ^{1}H NMR) as a liquid: ^{1}H NMR (300 MHz, $CDCl_3$) δ 6.39 (td, J_1 = 7.2 Hz, J_2 = 2.4 Hz, 1H, =CH), 5.35-5.19 (m, 1H, =CH), 5.06-4.94 (m, 1H, =CH), 3.68 (s, 3H, CH_3), 3.58 (q, J = 6.9 Hz, 1H, CH), [3.32 (s, 1.43H), 3.30 (s, 1.53H), 3H, CH_3], 2.51-2.28 (m, 4H, CH_2 × 2), 2.16-2.02 (m, 2H, CH_2), 1.70-1.18 (m, 8H, CH_2 × 4); IR (neat) ν (cm^{-1}) 2927, 2856, 2819, 1962, 1741, 1462, 1436, 1364, 1254, 1197, 1160, 1087; MS (ESI) *m/z* 449 [M (^{81}Br^{81}Br) + Na]$^{+}$, 447 [M (^{79}Br^{81}Br) + Na]$^{+}$, 445 [M (^{79}Br^{79}Br) + Na]$^{+}$; HRMS (ESI) calcd. for $C_{16}H_{24}{}^{79}Br^{79}BrNaO_3{}^{+}$ [M (^{79}Br^{79}Br) + Na]$^{+}$: 444.9984, found: 444.9966.

Synthesis of 14,14-dibromo-7-methoxy-4,5,13-tetradecatrienoic acid *rac*-**5k** (zj-7-121)

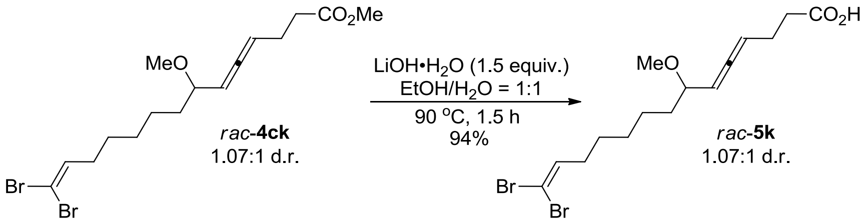

Following **Typical Procedure II**, the reaction of *rac*-**4ck** (0.9959 g, 2.35 mmol), EtOH/H_2O = 1:1 by volume (pre-mixed by using 12 mL of H_2O and 12 mL of EtOH) and LiOH·H_2O (0.1560 g, 3.525 mmol, 95%) for 1.5 h afforded *rac*-**5k** (0.9027 g, 94%, d.r. = 1.07:1 determined by ^{1}H NMR) [eluent: petroleum ether (60-90 ^{o}C)/ethyl acetate = 10/1 (440 mL) to 7/1 (560 mL) to 5/1 (600 mL) to 3/1 (120 mL)] as an oil: ^{1}H NMR (300 MHz, $CDCl_3$) δ 11.23 (bs, 1H, COOH), 6.38 (td, J_1 = 7.3 Hz, J_2 = 1.9 Hz, 1H, =CH), 5.34-5.21 (m, 1H, =CH), 5.07-4.97 (m, 1H, =CH), 3.60 (q, J = 6.7 Hz, 1H, CH), [3.33 (s, 1.45H), 3.31 (s, 1.55H), 3H, CH_3], 2.56-2.44 (m, 2H, CH_2), 2.41-2.28 (m, 2H, CH_2), 2.15-2.03 (m, 2H, CH_2), 1.71-1.23 (m, 8H, CH_2 × 4); IR (neat) ν (cm^{-1}) 3716-2200 (COOH), 3022, 2977, 2931, 2856, 2820, 2651, 1963, 1712, 1435, 1417, 1336, 1277, 1250, 1208, 1157, 1108, 1086; MS (ESI) *m/z* 435 [M (^{81}Br^{81}Br) + Na]$^{+}$, 433 [M (^{79}Br^{81}Br) + Na]$^{+}$, 431 [M (^{79}Br^{79}Br) + Na]$^{+}$; HRMS (ESI) calcd. for $C_{15}H_{22}{}^{79}Br^{79}BrNaO_3{}^{+}$ [M (^{79}Br^{79}Br) + Na]$^{+}$: 430.9828, found:430.9810.

Synthesis of 5-(10’,10’-dibromo-3’-methoxy-1’ (*E*),9’-decadien-1’-yl)dihydro-2 (3*H*)-furanone *rac*-**xestospongiene** (zj-7-158)

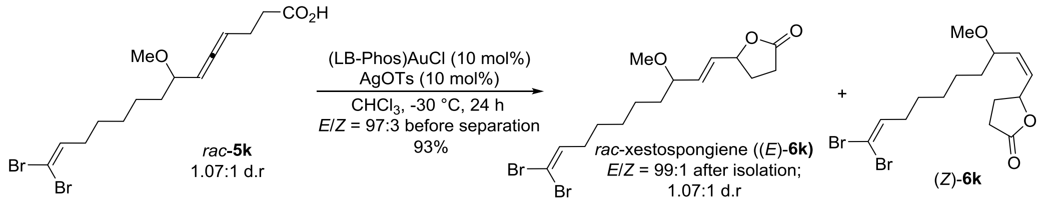

Typical Procedure VIII: To a dry Schlenk tube were added AgOTs (0.0285 g, 0.1 mmol, weighed in glove box, 98%), AuCl(LB-Phos) (0.0597 g, 0.1 mmol), and $CHCl_3$ (5 mL) under nitrogen atmosphere sequentially. After stirring at room temperature for 15 min, the reaction was cooled down to -30 ^oC and then *rac*-**5k** (0.4093 g, 1.0 mmol) and $CHCl_3$ (5 mL) were added. The reaction mixture was then continuously stirred at -30 ^oC for 24 h as monitored by TLC. The reaction was warmed up to room temperature. Filtration through a short column of silica gel [eluent: Et_2O (20 mL × 3)] and evaporation afforded a crude mixture of (*E*)-**6k** and (*Z*)-**6k** (*E*/*Z* = 97:3 determined by ^1H NMR analysis). Column chromatography on silica gel [eluent: petroleum ether (60-90 ^oC)/ethyl acetate = 15/1 (550 mL) to 10/1 (550 mL) to 8/1 (540 mL) to 5/1 (600 mL)] afforded the impure *rac*-xestospongiene (0.3886 g). The impure *rac*-xestospongiene was purified again on silica gel [eluent: petroleum ether (60-90 ^oC)/ethyl acetate/dichloromethane = 150/10/1 (480 mL) to 100/10/1 (550 mL) to 80/10/1 (540 mL × 3)] to give pure *rac*-xestospongiene (0.3792 g, 93%, *E*/*Z* = 99:1 determined by ^1H NMR, d.r. = 1.07:1 determined by quantitative ^{13}C NMR analysis) as a liquid: ^1H NMR (300 MHz, $CDCl_3$) δ 6.39 (t, *J* = 7.4 Hz, 1H, =CH), 5.80-5.57 (m, 2H, =CH × 2), 4.98 (q, *J* = 6.6 Hz, 1H, CH), 3.68-3.50 (m, 1H, CH), 3.26 (s, 3H, CH_3), 2.66-2.37 (m, 3H, CH_2 + one proton of CH_2), 2.16-1.93 (m, 3H, CH_2 + one proton of CH_2), 1.67-1.17 (m, 8H, CH_2 × 4); the following signals are discernible for (*Z*)-**6k**: δ 5.56-5.46 (m, 1H, CH), 5.35-5.26 (m, 1H, CH); IR (neat) ν (cm^{-1}) 2931, 2857, 2821, 1777, 1461, 1422, 1326, 1176, 1113, 1088, 1012; MS (ESI) *m/z* 413 [M ($^{81}Br^{81}Br$) + H]$^+$, 411 (M [$^{79}Br^{81}Br$) + H]$^+$, 409 [M ($^{79}Br^{79}Br$) + H]$^+$; HRMS (ESI) calcd for $C_{15}H_{23}{}^{79}Br^{79}Br\ O_3{}^+$ [M ($^{79}Br^{79}Br$) + H]$^+$: 409.0008, found:

409.0007.

2. Asymmetric synthesis of xestospongienes E-H.

I. Synthesis of optically active propargylic alcohols (*R*)-8 and (*S*)-8.

a. Synthesis of (*R*)-9-((*tert*-butyldimethylsilyl)oxy)non-1-yn-3-ol[3] (*R*)-**8** (zj-7-113)

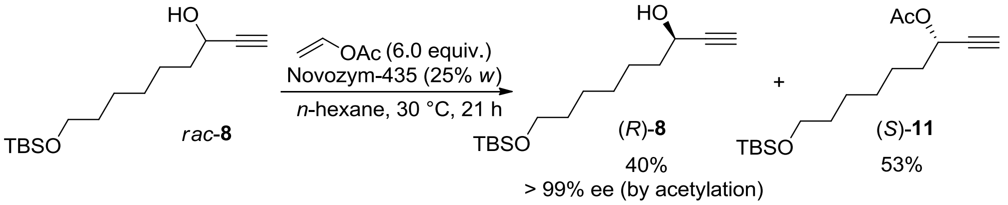

Typical Procedure IX: To a three-necked flask were added *rac*-**8** (10.8189 g, 40 mmol) and *n*-hexane (340 mL). Then vinyl acetate (22.2 mL, d = 0.93 g cm^{-3}, 20.646 g, 240.07 mmol) and Novozym-435 (2.7051 g, 25% *w*) were added sequentially. The reaction mixture was filtrated through a short column of silica gel [eluent: Et_2O (30 mL × 3)] after being stirred in an oil bath preheated at 30 °C for 21 h as monitored by TLC. After evaporation of the filtrate, the residue was purified by chromatography [eluent: petroleum ether (60-90 °C)/ethyl acetate = 100/1 (300 mL) to 50/1 (500 mL) to 30/1 (500 mL) to 15/1 (500 mL) to 10/1 (500 mL × 2)] on silica gel to afford (*S*)-**11** (6.6618 g, 53%) as a liquid and (*R*)-**8** (4.3075 g, 40%) as a liquid.

For (*S*)-**11**: ^{1}H NMR (300 MHz, $CDCl_3$) δ 5.33 (td, J_1 = 6.8 Hz, J_2 = 2.2 Hz, 1H, CH), 3.59 (t, *J* = 6.5 Hz, 2H, CH_2), 2.44 (d, *J* = 2.4 Hz, 1H, ≡CH), 2.08 (s, 3H, CH_3), 1.81-1.71 (m, 2H, CH_2), 1.56-1.29 (m, 8H, CH_2 × 4), 0.88 (s, 9H, CH_3 × 3), 0.04 (s, 6H, CH_3 × 2).

For (*R*)-**8**: > 99% ee (determined by the corrsponding acylation product (*R*)-**11**); $[\alpha]_D^{20}$ = +2.0 (c = 0.95, $CHCl_3$); ^{1}H NMR (300 MHz, $CDCl_3$) δ 4.41-4.30 (m, 1H, CH), 3.58 (t, *J* = 6.5 Hz, 2H, CH_2), 2.44 (d, *J* = 2.1 Hz, 1H, ≡CH), 2.24 (bs, 1H, OH), 1.77-1.63 (m, 2H, CH_2), 1.58-1.24 (m, 8H, CH_2 × 4), 0.87 (s, 9H, CH_3 × 3), 0.03 (s, 6H, CH_3 × 2); ^{13}C NMR (75 MHz, $CDCl_3$) δ 85.0, 72.8, 63.2, 62.2, 37.5, 32.7, 29.0, 25.9, 25.6, 25.0, 18.3, -5.3; IR (neat) ν (cm^{-1}) 3402, 3312, 2931, 2858, 2112, 1471, 1461, 1389, 1361, 1256, 1100, 1006; MS (ESI) *m/z* 293 [M + Na]$^+$, 271 [M + H]$^+$; HRMS (ESI) calcd for $C_{15}H_{30}NaO_2Si^+$ [M + Na]$^+$: 293.1907, found: 293.1910.

Acetylation for determination of the ee value of (*R*)-**8**: synthesis of (*R*)-9-((*tert*-butyldimethylsilyl)oxy)non-1-yn-3-yl acetate (*R*)-**11**[18] (zj-7-117)

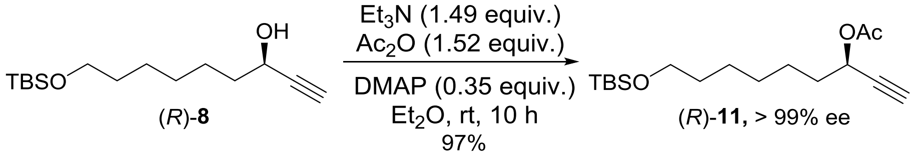

To a dry Schlenk tube were added (*R*)-**8** (0.0802 g, 0.3 mmol, prepared above), Et_2O (3 mL), DMAP (0.0128 g, 0.105 mmol), triethylamine (62 μL, d = 0.73 g cm^{-3}, 0.0453 g, 0.448 mmol), and acetic anhydride (43 μL, d = 1.08 g cm^{-3}, 0.0464 g, 0.455 mmol) sequentially under nitrogen atmosphere. After continuous stirring for 10 h at room temperature, the reaction was complete as monitored by TLC. After evaporation, the residue was purified by chromatography [eluent: petroleum ether (60-90 ^oC)/ethyl acetate = 20/1 (250 mL)] on silica gel to afford (*R*)-**11** (0.0897 g, 97%) as a liquid: >99% ee [GC conditions: CP-Chirasil-DEX CB column (25 m × 0.25 mm × 0.25 μm); carrier: N_2 (10.0 psi); injector: 145 ^oC; detector (FID, H_2): 200 ^oC; oven temperature: 50 ^oC (8 min), 50 ^oC to 150 ^oC (3 ^oC/min, 5 min), 150 ^oC to 180 ^oC ; t_R (major) = 55.9 min]; $[\alpha]_D^{20}$ = +43.9 (c = 0.99, $CHCl_3$); ^1H NMR (300 MHz, $CDCl_3$) δ 5.32 (td, J_1 = 6.7 Hz, J_2 = 2.2 Hz, 1H, CH), 3.58 (t, *J* = 6.5 Hz, 2H, CH_2), 2.44 (d, *J* = 2.4 Hz, 1H, ≡CH), 2.07 (s, 3H, CH_3), 1.80-1.69 (m, 2H, CH_2), 1.55-1.26 (m, 8H, CH_2 × 4), 0.87 (s, 9H, CH_3 × 3), 0.03 (s, 6H, CH_3 × 2); ^{13}C NMR (75 MHz, CDCl3) δ 169.9, 81.2, 73.4, 63.7, 63.1, 34.5, 32.6, 28.8, 25.9, 25.6, 24.8, 20.9, 18.3, -5.3; IR (neat) ν (cm^{-1}) 3312, 2932, 2858, 2123, 1746, 1472, 1463, 1371, 1232, 1098, 1022; MS (ESI) *m/z* 335 $[M + Na]^+$, 313 $[M + H]^+$; HRMS (ESI) calcd for $C_{17}H_{32}NaO_3Si^+$ $[M + Na]^+$: 335.2013, found: 335.2010.

b. Synthesis of (*S*)-9-((*tert*-butyldimethylsilyl)oxy)non-1-yn-3-ol (*S*)-**8** (zj-7-118, zj-7-120, zj-7-140)

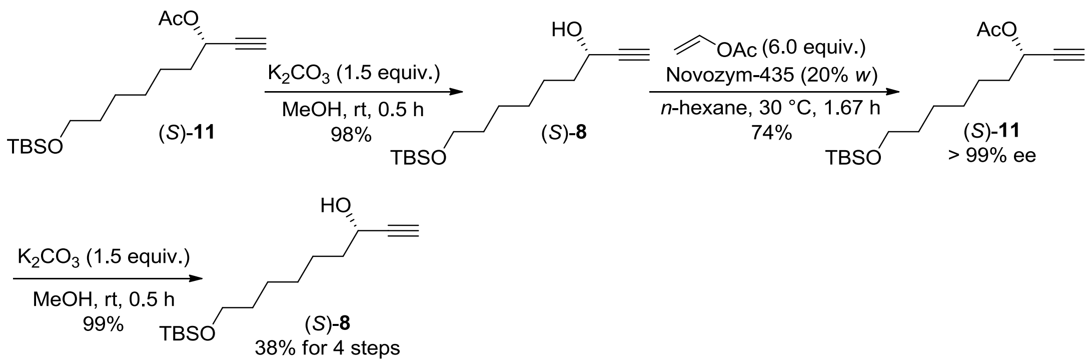

Typical Procedure X: [19] To a round-bottom flask were added (*S*)-**11** (6.6618 g, 21.3 mmol, prepared above, see page 46) and MeOH (22 mL). Then K_2CO_3 (4.4087 g, 31.95 mmol) was added. The reaction was complete as monitored by TLC after being stirred at room temperature for 0.5 h. K_2CO_3 was filtrated off and washed with Et_2O (15 mL ×5). After combining the filtrate and evaporation, the residue was purified by chromatography [eluent: petroleum ether (60-90 ^oC)/ethyl acetate = 100/1 (300 mL) to 50/1 (400 mL) to 30/1 (500 mL) to 10/1 (900 mL)] on silica gel to afford (*R*)-9-((*tert*-butyldimethylsilyl)oxy)non-1-yn-3-ol (*S*)-**8** (5.6511 g, 98%) as a liquid, which was then submitted to next step without further characterization: ^1H NMR (300 MHz, $CDCl_3$) δ 4.38-4.30 (m, 1H, CH), 3.58 (t, *J* = 6.5 Hz, 2H, CH_2), 2.46-2.39 (m, 2H, OH + ≡CH), 1.74-1.64 (m, 2H, CH_2), 1.56-1.27 (m, 8H, CH_2 ×4), 0.87 (s, 9H, CH_3 ×3), 0.02 (s, 6H, CH_3 ×2),

Following **Typical Procedure IX**, the reaction of (*S*)-**8** (5.6305 g, 20.8 mmol, prepared above), Novozym-435 (1.1257 g, 20% *w*), and vinyl acetate (11.55 mL, d = 0.93 g cm^{-3}, 10.7415 g, 124.9 mmol) in *n*-hexane (190 mL) for 1.67 h afforded (*S*)-9-((*tert*-butyldimethylsilyl)oxy)non-1-yn-3-yl acetate (*S*)-**11** (4.8175 g, 74%) [eluent: petroleum ether (60-90 ^oC)/ethyl acetate = 100/1 (500 mL) to 50/1 (500 mL × 2) to 30/1 (300 mL) to 10/1 (550 mL) to 5/1 (300 mL)] as a liquid: > 99% ee [GC conditions: CP-Chirasil-DEX CB column (25 m × 0.25 mm × 0.25 μm); carrier: N_2 (10.0 psi); injector: 145 ^oC; detector (FID, H_2): 200 ^oC; oven temperature: 50 ^oC (8 min), 50 ^oC to 150 ^oC (3 ^oC/min, 5 min), 150 ^oC to 180 ^oC; t_R (major) = 56.3 min]; $[\alpha]_D^{20}$ = -45.5 (c = 0.995, $CHCl_3$); ^1H NMR (300 MHz, $CDCl_3$) δ 5.32 (td, J_1 = 6.6 Hz, J_2 = 1.8 Hz, 1H, CH), 3.58 (t, *J* = 6.5 Hz, 2H, CH_2), 2.44 (d, *J* = 2.1 Hz, 1H, ≡CH), 2.07 (s, 3H, CH_3), 1.80-1.70 (m, 2H, CH_2), 1.56-1.27 (m, 8H, CH_2 × 4), 0.88 (s, 9H,

CH_3 × 3), 0.03 (s, 6H, CH_3 × 2); ^{13}C NMR (75 MHz, $CDCl_3$) δ 169.9, 81.2, 73.4, 63.7, 63.1, 34.4, 32.6, 28.8, 25.9, 25.6, 24.8, 20.9, 18.3, -5.3; IR (neat) ν (cm^{-1}) 3312, 2931, 2858, 2123, 1746, 1472, 1463, 1435, 1372, 1232, 1098, 1022; MS (ESI) *m/z* 335 [M + Na]$^+$, 313 [M + H]$^+$; HRMS (ESI) calcd for $C_{17}H_{32}NaO_3Si^+$ [M + Na]$^+$: 335.2013, found: 335.2014.

Following **Typical Procedure X**, the reaction of (*S*)-**11** (4.7490 g, 15.2 mmol, prepared above), K_2CO_3 (3.1471 g, 22.8 mmol), and MeOH (16 mL) for 0.5 h afforded (*S*)-**8** (4.0801 g, 99%; 38% for 4 steps) [eluent: petroleum ether (60-90 ^{o}C)/ethyl acetate = 50/1 (200 mL) to 30/1 (300 mL) to 15/1 (500 mL) to 10/1 (600 mL)] as a liquid: $[\alpha]_D^{20}$ = -2.2 (c = 1.00, $CHCl_3$); ^1H NMR (300 MHz, $CDCl_3$) δ 4.38-4.30 (m, 1H, CH), 3.58 (t, *J* = 6.5 Hz, 2H, CH_2), 2.46-2.39 (m, 2H, OH + ≡CH), 1.74-1.64 (m, 2H, CH_2), 1.55-1.27 (m, 8H, CH_2 × 4), 0.87 (s, 9H, CH_3 × 3), 0.02 (s, 6H, CH_3 × 2); ^{13}C NMR (75 MHz, $CDCl_3$) δ 85.0, 72.7, 63.2, 62.1, 37.5, 32.6, 29.0, 25.9, 25.6, 24.9, 18.3, -5.3; IR (neat) ν (cm^{-1}) 3398, 3312, 2931, 2858, 2115, 1470, 1463, 1388, 1360, 1255, 1100, 1006; MS (ESI) *m/z* 293 [M + Na]$^+$, 271 [M + H]$^+$; HRMS (ESI) calcd for $C_{15}H_{30}NaO_2Si^+$ [M + Na]$^+$: 293.1907, found: 293.1908.

II. Synthesis of optically active propargylic alcohols (*R*)-1c and (*S*)-1c

a. Synthesis of (*R*)-1,1-dibromo-8-methoxydec-1-en-9-yne (*R*)-**1c**

Synthesis of (*R*)-7-methoxynon-8-yn-1-ol (*R*)-**9** (zj-7-126, 7-129)

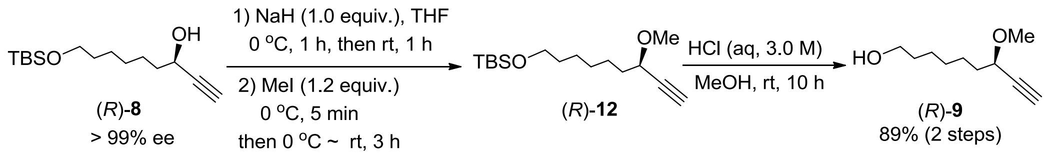

Typical Procedure XI: To a dry Schlenk flask were added (*R*)-**8** (4.1179 g, 15.2 mmol) and THF (45 mL) under nitrogen atmosphere. The resulting solution was cooled to 0 ℃ with an ice bath, and NaH (0.6091 g, 60% dispersion in mineral oil, 15.2 mmol,) was added carefully. After the addition was complete, the resulting mixture was stirred at 0 ℃ for 1 h. Then the cooling bath was removed and the resulting mixture was stirred for another 1 h followed by the addition of MeI (1.15 mL, d = 0.93 g cm^{-3}, 2.5958 g, 18.29 mmol, 99%) dropwise at 0 ℃ within 5 min. The

resulting mixture was warmed up to room temperature naturally. After 3 h with stirring, the reaction was complete as monitored by TLC. The reaction was quenched with water (5.0 mL) at 0 ℃. After separation of the organic layer, the aqueous layer was extracted with Et_2O (10 mL × 3). The combined organic layer was washed with brine and dried over anhydrous Na_2SO_4. After filtration and evaporation, the residue was purified by chromatography [eluent: petroleum ether (60-90 ^oC)/ethyl acetate = 100/1 (500 mL × 2) to 50/1 (500 mL)] on silica gel to afford (*R*)-*tert*-butyl((7-methoxynon-8-yn-1-yl)oxy)dimethylsilane (*R*)-**12** as a liquid, which was then submitted to next step without characterization .

To a round-bottom flask were added (*R*)-**12** (prepared above) and MeOH (60 mL). Then 3.0 M HCl (1.5 mL) was added. The reaction was complete as monitored by TLC after being stirred at room temperature for 10 h. After evaporation, the residue was purified by chromatography [eluent: petroleum ether (60-90 ^oC)/ethyl acetate = 10/1 (550 mL) to 5/1 (600 mL) to 3/1 (900 mL)] on silica gel to afford (*R*)-**9** (2.3027 g, 89%, 2 steps) as a liquid: $[\alpha]_D^{20}$ = +57.8 (c = 0.99, $CHCl_3$); ^1H NMR (300 MHz, $CDCl_3$) δ 3.94 (td, J_1 = 6.5 Hz, J_2 = 2.1 Hz, 1H, CH), 3.59 (t, J = 6.6 Hz, 2H, CH_2), 3.40 (s, 3H, CH_3), 2.80 (bs, 1H, OH), 2.48 (d, J = 2.1 Hz, 1H, ≡CH), 1.78-1.66 (m, 2H, CH_2), 1.61-1.30 (m, 8H, CH_2 × 4); ^{13}C NMR (75 MHz, $CDCl_3$) δ 82.2, 73.7, 70.7, 62.0, 56.0, 35.0, 32.2, 28.8, 25.3, 24.7; IR (neat) ν (cm^{-1}) 3381, 3295, 2986, 2933, 2859, 2824, 2110, 1464, 1336, 1195, 1109, 1091, 1057; MS (ESI) *m/z* 193 [M + Na]$^+$, 171 [M + H]$^+$; HRMS (ESI) calcd for $C_{10}H_{18}NaO_2^+$ [M + Na]$^+$: 193.1199, found: 193.1200.

Synthesis of (*R*)-7-methoxynon-8-ynal (*R*)-**10** (zj-7-135)

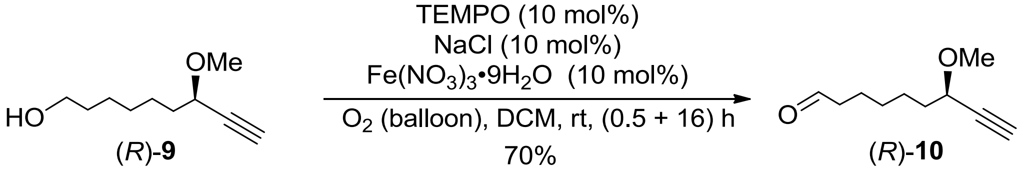

Typical Procedure XII: To a three-necked flask were added $Fe(NO_3)_3·9H_2O$ (0.5307 g, 1.29 mmol, 98%), NaCl (0.0751 g, 1.29 mmol), TEMPO (0.2055 g, 1.29

mmol, 98%), and DCM (100 mL) subsequently. An oxygen balloon was equipped and then a solution of alcohol (*R*)-**9** (2.1921 g, 12.9 mmol) in 30 mL of DCM was added dropwise via an addition funnel at room temperature within 0.5 h in oxygen atmosphere from the balloon. The resulting mixture was stirred for 16 h until the reaction was complete as monitored by TLC. After filtration through a short column of silica gel [eluent: Et_2O (20 mL × 3)] and evaporation, the residue was purified by chromatography [eluent: petroleum ether (60-90 ^oC)/ethyl acetate = 50/1 (200 mL) to 30/1 (500 mL) to 20/1 (500 mL) to 10/1 (440 mL)] on silica gel to afford (*R*)-**10** (1.5245 g, 70%) as a liquid: $[\alpha]_D^{20}$ = +58.8 (c = 1.00, $CHCl_3$); ^1H NMR (300 MHz, $CDCl_3$) δ 9.76 (t, *J* = 1.5 Hz, 1H, CHO), 3.93 (td, J_1 = 6.4 Hz, J_2 = 1.7 Hz, 1H, CH), 3.40 (s, 3H, CH_3), 2.52-2.40 (m, 3H, ≡CH + CH_2), 1.77-1.60 (m, 4H, CH_2 × 2), 1.55-1.30 (m, 4H, CH_2 × 2); ^{13}C NMR (75 MHz, $CDCl_3$) δ 202.0, 81.9, 73.4, 70.2, 55.8, 43.0, 34.5, 28.1, 24.1, 21.2; IR (neat) ν (cm^{-1}) 3280, 2986, 2939, 2862, 2824, 2723, 2109, 1724, 1465, 1409, 1391, 1336, 1193, 1156, 1108, 1091; MS (ESI) *m/z* 191 $[M + Na]^+$; HRMS (ESI) calcd for $C_{10}H_{16}NaO_2^+$ $[M + Na]^+$: 191.1043, found: 191.1045.

Synthesis of (*R*)-1,1-dibromo-8-methoxydec-1-en-9-yne (*R*)-**1c** (zj-7-137)

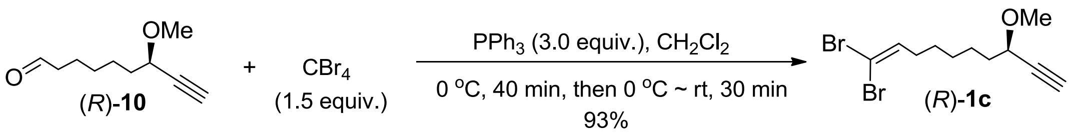

Following **Typical Procedure VII**, the reaction of (*R*)-**10** (1.4345 g, 8.54 mmol)/DCM (90 mL), CBr_4 (4.2532 g, 12.81 mmol), and PPh_3 (6.8541 g, 25.62 mmol, 98%)/DCM (35 mL) for 70 min afforded (*R*)-**1c** (2.5787 g, 93%) [eluent: petroleum ether (60-90 ^oC)/ethyl acetate = 100/1 (500 mL) to 50/l (500 mL) to 30/l (500 mL)] as a liquid: $[\alpha]_D^{20}$ = +29.5 (c = 1.01, $CHCl_3$); ^1H NMR (300 MHz, $CDCl_3$) δ 6.38 (t, *J* = 7.2 Hz, 1H, =CH), 3.94 (td, J_1 = 6.5 Hz, J_2 = 2.1 Hz, 1H, CH), 3.42 (s, 3H, CH_3), 2.45 (d, *J* = 2.1 Hz, 1H, ≡CH), 2.10 (q, *J* = 7.2 Hz, 2H, CH_2), 1.77-1.67 (m, 2H, CH_2), 1.53-1.27 (m, 6H, CH_2 × 3), ^{13}C NMR (75 MHz, $CDCl_3$) δ 138.7, 88.6, 82.6, 73.8, 70.9, 56.5, 35.3, 32.8, 28.6, 27.6, 24.8; IR (neat) ν (cm^{-1}) 3300, 2986, 2929, 2858, 2822, 2108, 1623, 1461, 1335, 1272, 1197, 1151, 1109; MS (EI) *m/z* (%) 325 [M

(^{81}Br^{81}Br) - H)$^+$, 0.10], 323 [M (^{79}Br^{81}Br) - H)$^+$, 0.18], 321 [M (^{79}Br^{79}Br) - H)$^+$, 0.13], 214 {[M (^{81}Br^{81}Br) - $C_7H_{11}O$]$^+$, 17.78}, 212 {[M (^{79}Br^{81}Br) - $C_7H_{11}O$]$^+$, 33.97}, 210 {[M (^{79}Br^{79}Br) - $C_7H_{11}O$]$^+$, 16.83}, 131 (100); HRMS (EI) calcd for $C_{11}H_{15}{}^{79}Br^{79}BrO$ [M - H]$^+$: 320.9490, found: 320.9496.

b. Synthesis of (*S*)-1,1-dibromo-8-methoxydec-1-en-9-yne (*S*)-**1c**

Synthesis of (*S*)-7-methoxynon-8-yn-1-ol (*S*)-**9** (zj-7-141, 7-142)

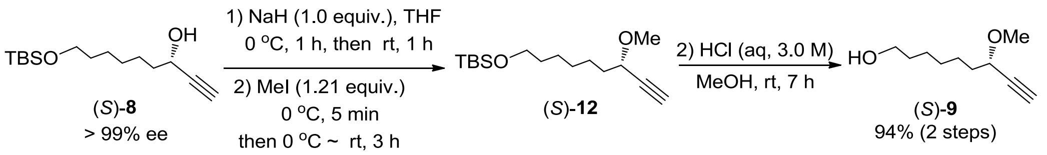

Following **Typical Procedure XI**, the reaction of (*S*)-**8** (4.0285 g, 14.9 mmol), NaH (0.5970 g, 60% dispersion in mineral oil, 14.9 mmol), MeI (1.13 mL, d = 2.28, 2.5506 g, 17.97 mmol, 99%) afforded (*S*)-**12** [eluent: petroleum ether (60-90 ^{o}C)/ethyl acetate = 100/1 (500 mL × 2) to 50/1 (500 mL)] as a liquid, which was then submitted to next step without characterization.

The reaction of (*S*)-**12** (prepared above), 3.0 M HCl (1.5 mL), and MeOH (60 mL) for 7 h afforded (*S*)-**9** (2.3743 g, 94%, 2 steps) [eluent: petroleum ether (60-90 ^{o}C)/ethyl acetate = 10/1 (550 mL) to 6/1 (560 mL) to 5/1 (600 mL) to 3/1 (280 mL)] as a liquid: $[\alpha]_D^{20}$ = -58.1 (c = 0.985, $CHCl_3$); ^{1}H NMR (300 MHz, $CDCl_3$) δ 3.94 (td, J_1 = 6.6 Hz, J_2 = 1.9 Hz, 1H, CH), 3.59 (t, J = 6.0 Hz, 2H, CH_2), 3.41 (s, 3H, CH_3), 2.98 (s, 1H, OH), 2.49 (d, J = 2.1 Hz, 1H, ≡CH), 1.78-1.64 (m, 2H, CH_2), 1.63-1.29 (m, 8H, CH_2 × 4); ^{13}C NMR (75 MHz, $CDCl_3$) δ 82.3, 73.7, 70.8, 62.3, 56.1, 35.1, 32.3, 28.8, 25.4, 24.8; IR (neat) ν (cm^{-1}) 3393, 3300, 2986, 2934, 2860, 2821, 2109, 1464, 1336, 1194, 1092; MS (ESI) *m/z* 191 [M + Na]$^+$; HRMS (ESI) calcd for $C_{10}H_{18}NaO_2^+$ [M + Na]$^+$: 193.1199, found: 193.1197.

Synthesis of (*S*)-7-methoxynon-8-ynal (*S*)-**10** (zj-7-143)

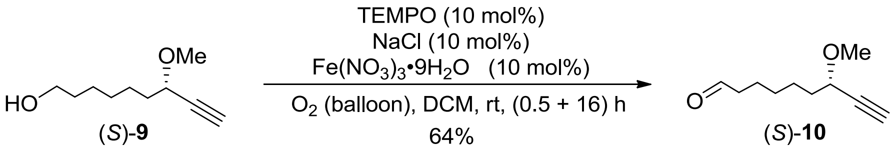

Following **Typical Procedure XII**, the reaction of $Fe(NO_3)_3·9H_2O$ (0.5473 g, 1.33 mmol, 98%), NaCl (0.0771 g, 1.33 mmol), TEMPO (0.2120 g, 1.33 mmol, 98%), DCM (100 mL), and (*S*)-**9** (2.2673 g, 13.3 mmol)/DCM (30 mL) for 16 h afforded (*S*)-**10** (1.4286 g, 64%) [eluent: petroleum ether (60-90 ^oC)/ethyl acetate = 50/1 (300 mL) to 30/1 (530 mL) to 20/1 (500 mL) to 10/1 (330 mL)] as a liquid: $[\alpha]_D^{20}$ = -59.1 (c = 0.98, $CHCl_3$); ^1H NMR (300 MHz, $CDCl_3$) δ 9.76 (t, *J* = 1.8 Hz, 1H, CHO), 3.94 (td, J_1 = 6.5 Hz, J_2 = 2.1 Hz, 1H, CH), 3.40 (s, 3H, CH_3), 2.50-2.41 (m, 3H, ≡CH + CH_2), 1.77-1.59 (m, 4H, CH_2 × 2), 1.55-1.30 (m, 4H, CH_2 × 2); ^{13}C NMR (75 MHz, $CDCl_3$) δ 202.3, 82.2, 73.7, 70.6, 56.1, 43.4, 34.9, 28.5, 24.5, 21.6; IR (neat) ν (cm^{-1}) 3282, 2985, 2939, 2862, 2824, 2722, 2108, 1724, 1464, 1411, 1391, 1336, 1156, 1108, 1091; MS (ESI) *m/z* 191 $[M + Na]^+$; HRMS (ESI) calcd for $C_{10}H_{16}NaO_2^+$ $[M + Na]^+$: 191.1043, found: 191.1041.

Synthesis of (*S*)-1,1-dibromo-8-methoxydec-1-en-9-yne (*S*)-**1c** (zj-7-146)

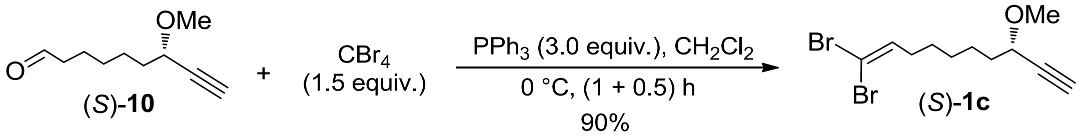

Following **Typical Procedure VII**, the reaction of (*S*)-**10** (1.3286 g, 7.9 mmol)/DCM (90 mL), CBr_4 (3.9307 g, 11.85 mmol), and PPh_3 (6.3361 g, 23.7 mmol, 98%)/DCM (25 mL) afforded (*S*)-**1c** (2.3172 g, 90%) [eluent: petroleum ether (60-90 ^oC)/ethyl acetate = 100/1 (500 mL) to 50/l (500 mL) to 30/l (300 mL)] as a liquid: $[\alpha]_D^{20}$ = -29.2 (c = 0.975, $CHCl_3$); ^1H NMR (300 MHz, $CDCl_3$) δ 6.38 (t, *J* = 7.4 Hz, 1H, =CH), 3.93 (td, J_1 = 6.5 Hz, J_2 = 1.9 Hz, 1H, CH), 3.41 (s, 3H, CH_3), 2.45 (d, *J* = 2.1 Hz, 1H, ≡CH), 2.10 (q, *J* = 7.3 Hz, 2H, CH_2), 1.77-1.66 (m, 2H, CH_2), 1.53-1.28 (m, 6H, CH_2 × 3), ^{13}C NMR (75 MHz, $CDCl_3$) δ 138.6, 88.6, 82.5, 73.8, 70.8, 56.4, 35.2, 32.8, 28.6, 27.6, 24.7; IR (neat) ν (cm^{-1}) 3301, 2986, 2931, 2858, 2822, 2110, 1622, 1463, 1335, 1274, 1196, 1152, 1109; MS (EI) *m/z* (%) 325 {$[M (^{81}Br^{81}Br) - H]^+$, 0.034}, 323 {$[M (^{79}Br^{81}Br) - H]^+$, 0.069}, 321 {$[M (^{79}Br^{79}Br) - H]^+$, 0.03}, 201 {$[M (^{81}Br^{81}Br) - C_8H_{13}O]^+$, 6.70}, 199 {$[M (^{79}Br^{81}Br) - C_8H_{13}O]^+$, 14.91}, 197 {$[M (^{79}Br^{79}Br) - C_8H_{13}O]^+$, 6.62}, 69 (100); HRMS (EI) calcd for $C_{11}H_{15}{}^{79}Br^{79}BrO$ [M -

H]$^+$: 320.9490, found: 320.9475.

III. Stereodivergent synthesis of xestospongienes E~H.

a. Synthesis of (5*S*,1'*E*,3'*R*)-**6k**, i.e., **xestospongiene F** (reported as **xestospongiene E**)

Synthesis of (R_a,*R*)-methyl 14,14-dibromo-7-methoxy-4,5,13-tetradecatrienoate (R_a,*R*)-**4ck** (zj-7-138)

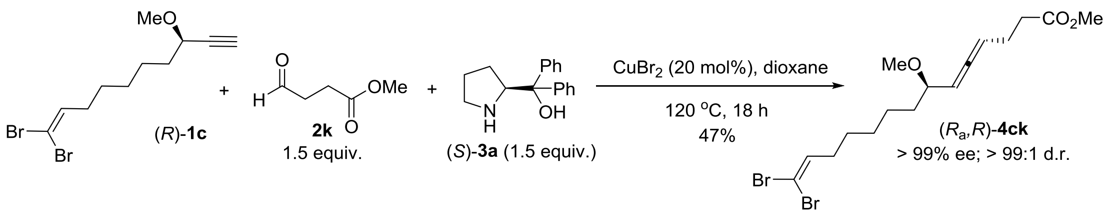

Typical Procedure XIII: To a dry Schlenk flask were added $CuBr_2$ (0.1735 g, 0.77 mmol, 99%), (*S*)-**3a** (1.4943 g, 5.775 mmol), (*R*)-**1c** (1.2481 g, 3.85 mmol)/dioxane (25 mL), and **2k** (0.6702 g, 5.775 mmol)/dioxane (13.5 mL) sequentially under nitrogen atmosphere. After continuous stirring for 18 h under reflux at 120 °C, the reaction was complete as monitored by TLC. Then the resulting mixture was cooled down to room temperature. The mixture was diluted with ether (60 mL) and then washed with an aqueous solution of hydrochloric acid (3 M, 10 mL × 3). The organic layer was separated and the aqueous layer was extracted with Et_2O (10 mL). The combined organic layer was washed with brine and dried over anhydrous Na_2SO_4. After filtration and evaporation, the residue was purified via chromatography [eluent: petroleum ether (60-90 °C)/ethyl acetate = 50/1 (500 mL) to 30/1 (510 mL) to 15/1 (480 mL)] on silica gel to afford (R_a,*R*)-**4ck** (0.7631 g, 47%, > 99:1 d.r. determined by HPLC analysis) as a liquid: > 99% ee (HPLC conditions: Chiralcel IF column, *n*-hexane/*i*-PrOH = 100/1, 1.0 mL/min, λ = 214 nm, t_R (major) = 25.9 min); $[\alpha]_D^{20}$ = -21.0 (c = 0.975, $CHCl_3$); ^1H NMR (300 MHz, $CDCl_3$) δ 6.39 (t, *J* = 7.2 Hz, 1H, =CH), 5.29 (q, *J* = 6.1 Hz, 1H, =CH), 5.05-4.95 (m, 1H, =CH), 3.68 (s, 3H, CH_3), 3.58 (q, *J* = 6.9 Hz, 1H, CH), 3.30 (s, 3H, CH_3), 2.48-2.39 (m, 2H, CH_2), 2.38-2.28 (m, 2H, CH_2), 2.10 (q, *J* = 7.2 Hz, 2H, CH_2), 1.70-1.25 (m, 8H, CH_2 × 4); ^{13}C NMR (75 MHz, $CDCl_3$) δ 204.2, 173.1, 138.6, 93.3, 90.3, 88.5, 80.2, 56.0, 51.5,

35.5, 33.0, 32.8, 28.8, 27.6, 25.1, 23.6; IR (neat) ν (cm^{-1}) 2978, 2931, 2857, 2819, 1963, 1739, 1624, 1436, 1362, 1343, 1253, 1198, 1161, 1087; MS (ESI) *m/z* 449 [M ($^{81}Br^{81}Br$) + Na]$^+$, 447 [M ($^{79}Br^{81}Br$) + Na]$^+$, 445 [M ($^{79}Br^{79}Br$) + Na]$^+$, HRMS (ESI) calcd for $C_{16}H_{24}{}^{79}Br^{79}Br\ NaO_3^+$ [(M ($^{79}Br^{79}Br$) + Na]$^+$: 444.9984, found: 444.9989.

Synthesis of (R_a,*R*)-14,14-dibromo-7-methoxy-4,5,13-tetradecatrienoic acid (R_a,*R*)-**5k** (zj-7-144)

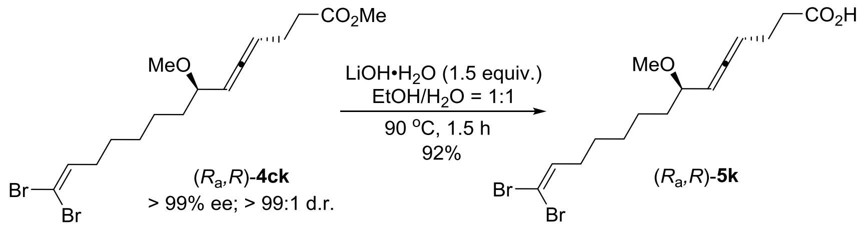

Following **Typical Procedure II**, the reaction of (R_a,*R*)-**4ck** (0.6801 g, 1.6 mmol), EtOH/H_2O = 1:1 by volume (pre-mixed by using 8 mL of H_2O and 8 mL of EtOH), and LiOH·H_2O (0.1063 g, 2.4 mmol) at 90 °C for 1.5 h afforded (R_a,*R*)-**5k** (0.6017 g, 92%) [eluent: petroleum ether (60-90 °C)/ethyl acetate = 10/1 (550 mL) to 8/1 (540 mL) to 5/1 (600 mL)] as an oil: $[\alpha]_D^{20}$ = -20.2 (c = 1.00, $CHCl_3$); ^1H NMR (300 MHz, $CDCl_3$) δ 9.68 (bs, 1H, COOH), 6.39 (t, *J* = 7.4 Hz, 1H, =CH), 5.30 (q, *J* = 6.1 Hz, 1H, =CH), 5.07-4.98 (m, 1H, =CH), 3.60 (q, J_1 = 7.0 Hz, 1H, CH), 3.31 (s, 3H, CH_3), 2.54-2.44 (m, 2H, CH_2), 2.39-2.29 (m, 2H, CH_2), 2.10 (q, *J* = 7.2 Hz, 2H, CH_2), 1.71-1.22 (m, 8H, CH_2 × 4); ^{13}C NMR (75 MHz, $CDCl_3$) δ 204.3, 178.9, 138.8, 93.5, 90.2, 88.6, 80.4, 56.0, 35.5, 33.1, 32.9, 28.9, 27.7, 25.2, 23.4; IR (neat) ν (cm^{-1}) 3752-2188 (COOH), 2978, 2933, 2858, 2822, 2660, 1964, 1713, 1624, 1435, 1336, 1278, 1251, 1208, 1157, 1109, 1086; MS (ESI) *m/z* 435 [M ($^{81}Br^{81}Br$) + Na]$^+$, 433 [M ($^{81}Br^{79}Br$) + Na]$^+$, 431 [M ($^{79}Br^{79}Br$) + Na]$^+$; HRMS (ESI) calcd. for $C_{15}H_{22}{}^{79}Br^{79}BrNaO_3^+$ [M ($^{79}Br^{79}Br$) + Na]$^+$: 430.9828, found: 430.9830.

Synthesis of (5*S*)-(10’,10’-dibromo-3’(*R*)-methoxy-1’(*E*),9’-decadien-1’-yl)dihydro-2(3*H*)-furanone (5*S*,1’*E*,3’*R*)-**6k**, i.e., **xestospongiene F** (reported as **xestospongiene E**) (zj-7-188)

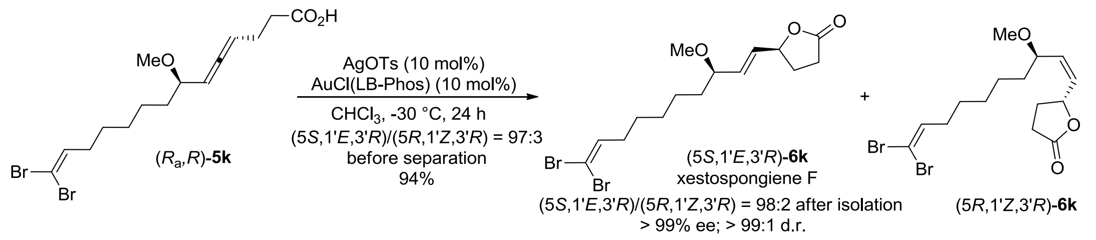

Following **Typical Procedure VIII**, the reaction of AgOTs (0.0285 g, 0.1 mmol, 98%), Au(LB-Phos)Cl (0.0597 g, 0.1 mmol), $CHCl_3$ (5 mL), and (*R*a,*R*)-**5k** (0.4096 g, 1.0 mmol)/$CHCl_3$ (5 mL) at -30 °C for 24 h afforded (5*S*,1'*E*,3'*R*)-**6k**,[20] i.e., xestospongiene F [(0.3844 g, 94%, (5*S*,1'*E*,3'*R*)/(5*R*,1'*Z*,3'*R*) = 98:2 (determined by ^{1}H NMR) and > 99:1 d.r. (determined by SFC analysis)] [eluent: petroleum ether (60-90 °C)/ethyl acetate/dichloromethane = 150/10/1 (480 mL) to 100/10/1 (550 mL) to 80/10/1 (540 mL × 3)] ((5*S*,1'*E*,3'*R*)/(5*R*,1'*Z*,3'*R*) = 97:3 determined by ^{1}H NMR of crude product) as a liquid: > 99% ee (SFC: Chiralcel AD-H column, *n*-hexane/*i*-PrOH = 97/3, 1.3 mL/min, λ = 214 nm, t_R (major) = 15.8 min); $[\alpha]_D^{20}$ = +21.9 (c = 1.01, $CHCl_3$); $[\alpha]_D^{20}$ = +27.3 (c = 0.38, MeOH); (Lit.[20] $[\alpha]_D^{20}$ = -44.3 (c = 0.5, MeOH)); ^{1}H NMR (300 MHz, $CDCl_3$) δ 6.39 (t, *J* = 7.4 Hz, 1H, =CH), 5.82-5.58 (m, 2H, =CH × 2), 4.98 (q, *J* = 6.6 Hz, 1H, CH), 3.58 (q, *J* = 6.2 Hz, 1H, CH), 3.27 (s, 3H, CH_3), 2.64-2.39 (m, 3H, CH_2 + one proton of CH_2), 2.15-1.94 (m, 3H, CH_2 + one proton of CH_2), 1.66-1.20 (m, 8H, CH_2 × 4); the following signals are discernible for (5*R*,1'*Z*,3'*R*)-**6k**: δ 5.56-5.47 (m, 1H, =CH), 5.35-5.25 (m, 1H, CH); ^{13}C NMR (75 MHz, $CDCl_3$) δ 176.6, 138.5, 134.1, 129.4, 88.2, 80.8, 79.7, 56.1, 34.8, 32.6, 28.6, 28.5, 28.2, 27.3, 24.6; IR (neat) ν (cm^{-1}) 2974, 2931, 2857, 2817, 1777, 1460, 1421, 1326, 1296, 1214, 1176, 1112, 1091, 1012; MS (ESI) *m/z* 413 [M (^{81}Br^{81}Br) + H]$^+$, 411 [M (^{79}Br^{81}Br) + H]$^+$, 409 [M (^{79}Br^{79}Br) + H]$^+$.

b. Synthesis of **xestospongiene G**

Synthesis of (*S*a,*R*)-methyl 14,14-dibromo-7-methoxy-4,5,13-tetradecatrienoate (*S*a,*R*)-**4ck** (zj-7-139)

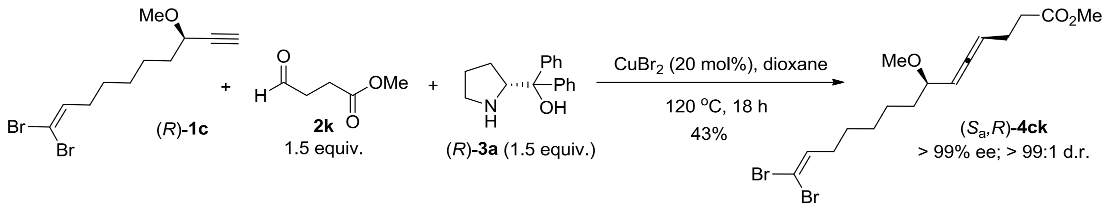

Following **Typical Procedure XIII**, the reaction of $CuBr_2$ (0.1736 g, 0.77 mmol, 99%), (*R*)-**3a** (1.4942 g, 5.775 mmol, 98%), (*R*)-**1c** (1.2469 g, 3.85 mmol)/dioxane (25 mL), and **2k** (0.6702 g, 5.775 mmol)/dioxane (13.5 mL) at 120 ^{o}C for 18 h afforded (S_a,*R*)-**4ck** (0.7035 g, 43%, > 99:1 d.r. by HPLC analysis) [eluent: petroleum ether (60-90 ^{o}C)/ethyl acetate = 50/1 (500 mL) to 30/1 (510 mL) to 15/1 (480 mL)] as a liquid: > 99% ee, > 99:1 d.r. (HPLC conditions: Chiralcel IF column, *n*-hexane/*i*-PrOH = 100/1, 1.0 mL/min, λ = 214 nm, t_R (major) = 36.6 min, t_R (minor) = 25.6 min); $[\alpha]_D^{20}$ = +72.2 (c = 1.025, $CHCl_3$); ^{1}H NMR (300 MHz, $CDCl_3$) δ 6.38 (t, *J* = 7.4 Hz, 1H, =CH), 5.23 (q, *J* = 6.7 Hz, 1H, =CH), 5.04-4.94 (m, 1H, =CH), 3.68 (s, 3H, CH_3), 3.58 (q, *J* = 6.9 Hz, 1H, CH), 3.32 (s, 3H, CH_3), 2.51-2.43 (m, 2H, CH_2), 2.40-2.31 (m, 2H, CH_2), 2.09 (q, *J* = 7.2 Hz, 2H, CH_2), 1.69-1.23 (m, 8H, CH_2 × 4); ^{13}C NMR (75 MHz, $CDCl_3$) δ 204.1, 173.1, 138.7, 93.3, 90.2, 88.5, 80.2, 56.0, 51.5, 35.6, 33.1, 32.8, 28.8, 27.6, 25.0, 23.8; IR (neat) ν (cm^{-1}) 2978, 2931, 2857, 2819, 1963, 1738, 1622, 1463, 1435, 1417, 1362, 1343, 1254, 1227, 1197, 1161, 1087, 1023; MS (ESI) *m/z* 449 [M (^{81}Br^{81}Br) + Na]$^+$, 447 [M (^{79}Br^{81}Br) + Na]$^+$, 445 [M (^{79}Br^{79}Br) + Na]$^+$; HRMS (ESI) calcd for $C_{16}H_{24}{}^{79}Br^{79}BrNaO_3{}^+$ [M (^{79}Br^{79}Br) + Na]$^+$: 444.9984, found: 444.9965.

Synthesis of (S_a,*R*)-14,14-dibromo-7-methoxy-4,5,13-tetradecatrienoic acid (S_a,*R*)-**5k** (zj-7-145)

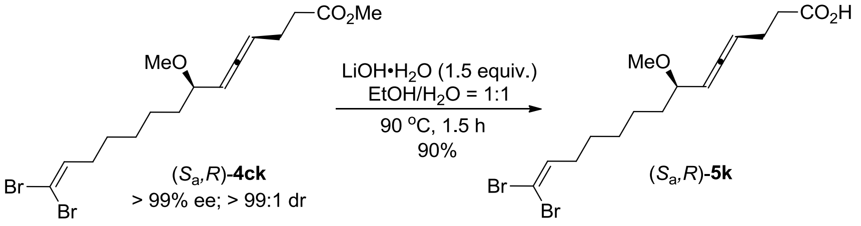

Following **Typical Procedure II**, the reaction of (S_a,*R*)-**4ck** (0.6031 g, 1.42 mmol), EtOH/H_2O = 1:1 by volume (pre-mixed by using 7 mL of H_2O and 7 mL of

EtOH), and LiOH·H_2O (0.0945 g, 2.13 mmol, 95%) at 90 °C for 1.5 h afforded (S_a,R)-**5k** (0.5272 g, 90%) [eluent: petroleum ether (60-90 °C)/ethyl acetate = 10/1 (550 mL) to 8/1 (540 mL) to 5/1 (600 mL)] as an oil: $[\alpha]_D^{20}$ = +72.3 (c = 1.00, $CHCl_3$); ^{1}H NMR (300 MHz, $CDCl_3$) δ 9.72 (bs, 1H, COOH), 6.38 (t, J = 7.1 Hz, 1H, =CH), 5.25 (q, J = 6.0 Hz, 1H, =CH), 5.06-4.97 (m, 1H, =CH), 3.60 (q, J = 6.9 Hz, 1H, CH), 3.33 (s, 3H, CH_3), 2.52 (t, J = 7.1 Hz, 2H, CH_2), 2.41-2.31 (m, 2H, CH_2), 2.09 (q, J = 7.3 Hz, 2H, CH_2), 1.70-1.22 (m, 8H, CH_2 × 4); ^{13}C NMR (75 MHz, $CDCl_3$) δ 204.2, 178.8, 138.7, 93.5, 90.2, 88.5, 80.3, 56.1, 35.6, 33.2, 32.8, 28.8, 27.7, 25.0, 23.6; IR (neat) ν (cm^{-1}) 3618-2182 (COOH), 2978, 2933, 2858, 2820, 2658, 1964, 1712, 1435, 1334, 1250, 1205, 1156, 1109, 1086; MS (ESI) m/z 435 [M (^{81}Br^{81}Br) + Na]$^+$, 433 [M (^{81}Br^{79}Br) + Na]$^+$, 431 [M (^{79}Br^{79}Br) + Na]$^+$; HRMS (ESI) calcd. for $C_{15}H_{22}{}^{79}Br^{79}BrNaO_3^+$ [M(^{79}Br^{79}Br) + Na]$^+$: 430.9828, found: 430.9832.

Synthesis of (5*R*)-(10’,10’-dibromo-3’(*R*)-methoxy-1’(*E*),9’-decadien-1’-yl) dihydro -2(3*H*)-furanone **xestospongiene G** (zj-8-012)

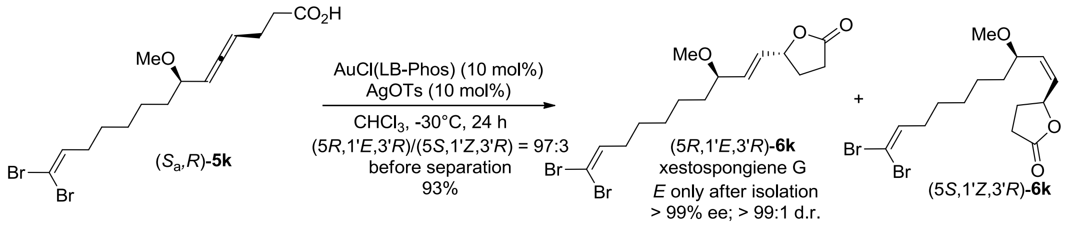

Following **Typical Procedure VIII**, the reaction of AgOTs (0.0285 g, 0.1 mmol, 98%), Au(LB-Phos)Cl (0.0598 g, 0.1 mmol), $CHCl_3$ (5 mL), and (S_a,R)-**5k** (0.4097 g, 1.0 mmol)/$CHCl_3$ (5 mL) at -30 °C for 24 h afforded xestospongiene G[20] (0.3791 g, 93%, *E* only, > 99:1 d.r. determined by SFC analysis) [eluent: petroleum ether (60-90 °C)/ethyl acetate/dichloromethane = 150/10/1 (480 mL) to 70/7/1 (550 mL) to 40/5/1 (540 mL) to 24/3/1 (540 mL)] ((5*R*,1’*E*,3’*R*)/(5*S*,1’*Z*,3’*R*) = 97:3 determined by ^{1}H NMR of crude product) as an oil: > 99% ee (SFC: Chiralcel AD-H column, *n*-hexane/*i*-PrOH = 9/1, 2.0 mL/min, λ = 214 nm, t_R (major) = 16.2 min); $[\alpha]_D^{20}$ = -3.4 (c = 0.98, $CHCl_3$); $[\alpha]_D^{20}$ = -7.1 (c = 0.545, MeOH); (Lit.[20] $[\alpha]_D^{20}$ = -10.5 (c = 0.5, MeOH)); ^{1}H NMR (300 MHz, $CDCl_3$) δ 6.38 (t, J = 7.2 Hz, 1H, =CH), 5.77-5.60 (m, 2H, =CH × 2), 4.98 (q, J = 6.5 Hz, 1H, CH), 3.57 (q, J = 6.3 Hz, 1H, CH), 3.27 (s, 3H,

CH_3), 2.61-2.37 (m, 3H, CH_2 + one proton of CH_2), 2.14-1.94 (m, 3H, CH_2 + one proton of CH_2), 1.64-1.22 (m, 8H, CH_2 × 4); ^{13}C NMR (75 MHz, $CDCl_3$) δ 176.5, 138.5, 133.9, 129.7, 88.2, 80.7, 79.6, 56.1, 34.7, 32.5, 28.5, 28.4, 28.1, 27.3, 24.5; IR (neat) ν (cm^{-1}) 2976, 2932, 2857, 2820, 1778, 1461, 1420, 1325, 1296, 1214, 1177, 1108, 1092, 1012; MS (ESI): *m/z* 413 [M (^{81}Br^{81}Br) + H]$^+$, 411 [M (^{79}Br^{81}Br) + H]$^+$, 409 [M (^{79}Br^{79}Br) + H]$^+$.

c. Synthesis of **xestospongiene H**

Synthesis of (R_a,*S*)-methyl 14,14-dibromo-7-methoxy-4,5,13-tetradecatrienoate (R_a,*S*)-**4ck** (zj-7-148)

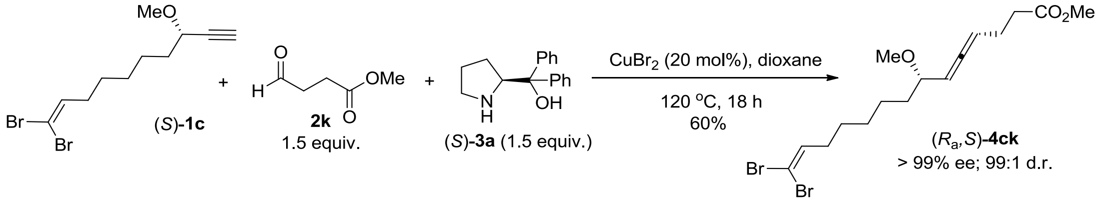

Typical Procedure XIV (three reactions in parallel): To a dry Schlenk flask were added $CuBr_2$ (0.0510 g, 0.226 mmol, 99%), (*S*)-**3a** (0.4380 g, 1.695 mmol, 98%), (*S*)-**1c** (0.3629 g, 1.13 mmol)/dioxane (8.3 mL), and **2k** (0.1970 g, 1.695 mmol)/dioxane (3 mL) sequentially under nitrogen atmosphere; to another dry Schlenk flask were added $CuBr_2$ (0.0508 g, 0.226 mmol, 99%), (*S*)-**3a** (0.4369 g, 1.695 mmol), (*S*)-**1c** (0.3651 g, 1.13 mmol, 98%)/dioxane (8.3 mL), and **2k** (0.1961 g, 1.695 mmol)/dioxane (3 mL) sequentially under nitrogen atmosphere; to the third dry Schlenk flask were added $CuBr_2$ (0.0508 g, 0.226 mmol, 99%), (*S*)-**3a** (0.4368 g, 1.695 mmol, 98%), (*S*)-**1c** (0.3667 g, 1.13 mmol)/dioxane (8.3 mL), and **2k** (0.1971 g, 1.695 mmol)/dioxane (3 mL) sequentially under nitrogen atmosphere; the three reactions were conducted separately. After continuous stirring for 18 h at 120 ^{o}C, the reactions were complete as monitored by TLC. After cooling down to room temperature, the three resulting mixtures were combined and diluted with ether (60 mL), then washed with an aqueous solution of hydrochloric acid (3 M, 10 mL × 3). The organic layer was separated and the aqueous layer was extracted with Et_2O (10 mL). The combined organic layer was washed with brine and dried over anhydrous

Na_2SO_4. After filtration and evaporation, the residue was purified by chromatography [eluent: petroleum ether (60-90 ^oC)/ethyl acetate = 50/1 (500 mL) to 30/1 (500 mL) to 20/1 (500 mL)] on silica gel to afford (R_a,S)-**4ck** (0.8064 g, 60%, 99:1 d.r. determined by ^1H NMR and HPLC analysis) as a liquid: > 99% ee (HPLC conditions: Chiralcel IF column, *n*-hexane/*i*-PrOH = 100/1, 1.0 mL/min, λ = 214 nm, t_R (major) = 25.7 min , t_R (minor) = 37.9 min); $[\alpha]_D^{20}$ = -71.9 (c = 1.025, $CHCl_3$); ^1H NMR (300 MHz, $CDCl_3$) δ 6.38 (t, J = 7.4 Hz, 1H, =CH), 5.24 (q, J = 6.5 Hz, 1H, =CH), 5.04-4.95 (m, 1H, =CH), 3.68 (s, 3H, CH_3), 3.58 (q, J = 6.9 Hz, 1H, CH), 3.32 (s, 3H, CH_3), 2.53-2.42 (m, 2H, CH_2), 2.41-2.30 (m, 2H, CH_2), 2.09 (q, J = 7.2 Hz, 2H, CH_2), 1.69-1.24 (m, 8H, CH_2 × 4); ^{13}C NMR (75 MHz, $CDCl_3$) δ 204.1, 173.1, 138.7, 93.3, 90.2, 88.5, 80.2, 56.0, 51.5, 35.6, 33.1, 32.8, 28.8, 27.6, 25.0, 23.8; IR (neat) ν (cm^{-1}) 2977, 2931, 2857, 2818, 1963, 1740, 1436, 1363, 1250, 1198, 1161, 1109, 1087; MS (ESI) *m/z* 449 [M ($^{81}Br^{81}Br$) + Na]$^+$, 447 [M ($^{79}Br^{81}Br$) + Na]$^+$, 445 [M ($^{79}Br^{79}Br$) + Na]$^+$; HRMS (ESI) calcd for $C_{16}H_{24}{}^{79}Br^{79}Br\ NaO_3^+$ [M ($^{79}Br^{79}Br$) + Na]$^+$: 444.9984, found: 444.9976.

Synthesis of (R_a,S)-14,14-dibromo-7-methoxy-4,5,13-tetradecatrienoic acid (R_a,S)-**5k** (zj-7-151)

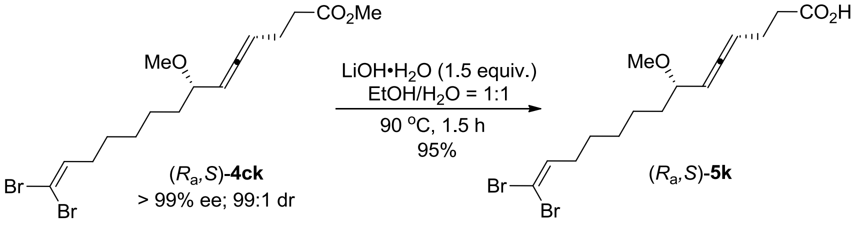

Following **Typical Procedure II**, the reaction of (R_a,S)-**4ck** (0.6664 g, 1.57 mmol), EtOH/H_2O = 1:1 by volume (pre-mixed by using 8 mL of H_2O and 8 mL of EtOH), and LiOH·H_2O (0.1047 g, 2.355 mmol) at 90 ^oC for 1.5 h afforded (R_a,S)-**5k** (0.6102 g, 95%) [eluent: petroleum ether (60-90 ^oC)/ethyl acetate = 10/1 (550 mL) to 8/1 (540 mL) to 5/1 (600 mL)] as a liquid: $[\alpha]_D^{20}$ = -72.2 (c = 1.01, $CHCl_3$); ^1H NMR (300 MHz, $CDCl_3$) δ ^1H NMR (300 MHz, $CDCl_3$) δ 10.73 (bs, 1H, COOH), 6.38 (t, J = 7.4 Hz, 1H, =CH), 5.25 (q, J = 6.2 Hz, 1H, =CH), 5.07-4.97 (m, 1H, =CH), 3.60 (q, J = 6.6 Hz, 1H, CH), 3.33 (s, 3H, CH_3), 2.57-2.46 (m, 2H, CH_2), 2.42-2.30 (m, 2H, CH_2), 2.09 (q, J = 7.2 Hz, 2H, CH_2), 1.70-1.23 (m, 8H, CH_2 × 4); ^{13}C NMR (75 MHz,

$CDCl_3$) δ 204.1, 178.8, 138.7, 93.4, 90.1, 88.5, 80.3, 56.0, 35.5, 33.1, 32.8, 28.7, 27.6, 25.0, 23.5; IR (neat) ν (cm^{-1}) 3693-2185 (COOH), 2976, 2933, 2858, 2820, 2664, 1963, 1712, 1435, 1335, 1251, 1206, 1156, 1108, 1086; MS (ESI) *m/z* 435 [M ($^{81}Br^{81}Br$) + Na]$^+$, 433 [M ($^{79}Br^{81}Br$) + Na]$^+$, 431 [M ($^{79}Br^{79}Br$) + Na]$^+$; HRMS (ESI) calcd. for $C_{15}H_{22}{}^{79}Br^{79}Br\ NaO_3{}^+$ [M ($^{79}Br^{79}Br$) + Na]$^+$: 430.9828, found: 430.9821.

Synthesis of (5*S*)-(10’,10’-dibromo-3’(*S*)-methoxy-1’(*E*),9’-decadien-1’-yl)dihydro-2(3*H*)-furanone **xestospongiene H** (zj-8-013)

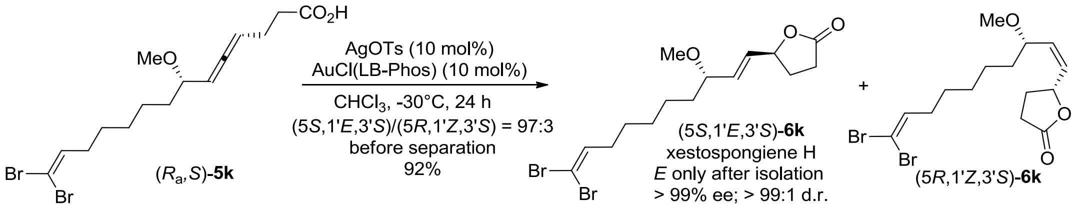

Following **Typical Procedure VIII**, the reaction of AgOTs (0.0285 g, 0.1 mmol, 98%), Au(LB-Phos)Cl (0.0597 g, 0.1 mmol), $CHCl_3$ (5 mL), and (R_a,*S*)-**5k** (0.4101 g, 1.0 mmol)/$CHCl_3$ (5 mL) at -30 ^oC for 24 h afforded xestospongiene H[20] (0.3766 g, 92%, *E* only, > 99:1 d.r. determined by SFC analysis) [eluent: petroleum ether (60-90 ^oC)/ethyl acetate/dichloromethane = 150/10/1 (480 mL) to 70/7/1 (550 mL) to 40/5/1 (540 mL) to 24/3/1 (540 mL)] ((5*S*,1’*E*,3’*S*)/(5*R*,1’*Z*,3’*S*) = 97:3 determined by ^1H NMR of crude product) as an oil: > 99% ee, > 99:1 d.r. (SFC: Chiralcel AD-H column, *n*-hexane/*i*-PrOH = 9/1, 2.0 mL/min, λ = 214 nm, t_R (major) = 9.4 min); $[\alpha]_D^{20}$ = +3.5 (c = 0.995, $CHCl_3$); $[\alpha]_D^{20}$ = +7.0 (c = 0.49, MeOH); (Lit.[20] $[\alpha]_D^{20}$ = +11.0 (c = 0.5, MeOH)); ^1H NMR (300 MHz, $CDCl_3$) δ 6.39 (t, *J* = 7.2 Hz, 1H, =CH), 5.78-5.58 (m, 2H, =CH × 2), 4.98 (q, *J* = 6.8 Hz, 1H, CH), 3.57 (q, *J* = 6.4 Hz, 1H, CH), 3.26 (s, 3H, CH_3), 2.60-2.37 (m, 3H, CH_2 + one proton of CH_2), 2.14-1.93 (m, 3H, CH_2 + one proton of CH_2), 1.66-1.19 (m, 8H, CH_2 × 4); ^{13}C NMR (75 MHz, $CDCl_3$) δ 176.4, 138.4, 133.8, 129.6, 88.1, 80.7, 79.5, 56.0, 34.7, 32.5, 28.5, 28.4, 28.1, 27.3, 24.5; IR (neat) ν (cm^{-1}) 2978, 2932, 2857, 2821, 1778, 1461, 1420, 1326, 1296, 1215, 1177, 1108, 1093, 1012; MS (ESI): *m/z* 413 [M ($^{81}Br^{81}Br$) + H]$^+$, 411 [M ($^{79}Br^{81}Br$) + H]$^+$, 409 [M ($^{79}Br^{79}Br$) + H]$^+$.

d. Synthesis of (5*R*,1'*E*,3'*S*)-**6k**, i.e., **xestospongiene E** (reported as **xestospongiene F**)

Synthesis of (S_a,*S*)-methyl 14,14-dibromo-7-methoxy-4,5,13-tetradecatrienoate (S_a,*S*)-**4ck** (zj-7-150)

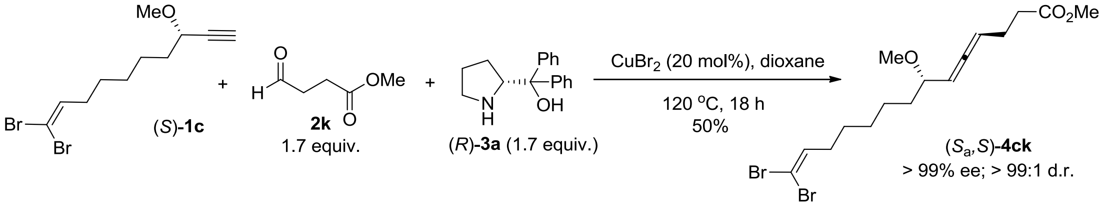

To a dry Schlenk flask were added $CuBr_2$ (0.1540 g, 0.682 mmol, 99%), (*R*)-**3a** (1.4957 g, 5.797 mmol, 98%), (*S*)-**1c** (1.1058 g, 3.41 mmol)/dioxane (20 mL), and **2k** (0.6740 g, 5.797 mmol)/dioxane (14 mL) sequentially under nitrogen atmosphere. After continuous stirring for 18 h at 120 ^{o}C, the reaction was complete as monitored by TLC. Then the resulting mixture was cooled down to room temperature, diluted with ether (60 mL), and washed with an aqueous solution of hydrochloric acid (3 M, 10 mL × 3). The organic layer was separated and the aqueous layer was extracted with Et_2O (10 mL). The combined organic layer was washed with brine and dried over anhydrous Na_2SO_4. After filtration and evaporation, the residue was purified by chromatography [eluent: petroleum ether (60-90 ^{o}C)/ethyl acetate = 50/1 (500 mL) to 30/1 (500 mL) to 20/1 (500 mL)] on silica gel to afford (S_a,*S*)-**4ck** (0.7177 g, 50%, > 99:1 d.r. determined by ^{1}H NMR and HPLC analysis) as a liquid: > 99% ee, > 99:1 d.r. (HPLC conditions: Chiralcel IF column, *n*-hexane/*i*-PrOH = 100/1, 1.0 mL/min, λ = 214 nm, t_R (major) = 14.8 min, t_R (minor) = 13.5 min); $[\alpha]_D^{20}$ = +21.2 (c = 0.98, $CHCl_3$); δ 6.39 (t, *J* = 7.2 Hz, 1H, =CH), 5.28 (q, *J* = 6.2 Hz, 1H, =CH), 5.04-4.95 (m, 1H, =CH), 3.68 (s, 3H, CH_3), 3.58 (q, *J* = 6.9 Hz, 1H, CH), 3.30 (s, 3H, CH_3), 2.47-2.39 (m, 2H, CH_2), 2.38-2.28 (m, 2H, CH_2), 2.10 (q, *J* = 7.3 Hz, 2H, CH_2), 1.70-1.25 (m, 8H, CH_2 × 4); ^{13}C NMR (75 MHz, $CDCl_3$) δ 204.1, 173.1, 138.7, 93.3, 90.3, 88.5, 80.2, 55.9, 51.5, 35.5, 33.0, 32.8, 28.8, 27.6, 25.0, 23.6; IR (neat) ν (cm^{-1}) 2981, 2931, 2857, 2819, 1963, 1740, 1436, 1363, 1254, 1198, 1161, 1109, 1087; MS (ESI) *m/z* 449 [M (^{81}Br^{81}Br) + Na]$^+$, 447 [M (^{79}Br^{81}Br) + Na]$^+$, 445 [M (^{79}Br^{79}Br) + Na]$^+$; HRMS (ESI) calcd for $C_{16}H_{24}{}^{79}Br^{79}BrNaO_3^+$ [M (^{79}Br^{79}Br) + Na]$^+$: 444.9984,

found: 444.9969.

Synthesis of (S_a,S)-14,14-dibromo-7-methoxy-4,5,13-tetradecatrienoic acid (S_a,S)-**5k** (zj-7-156)

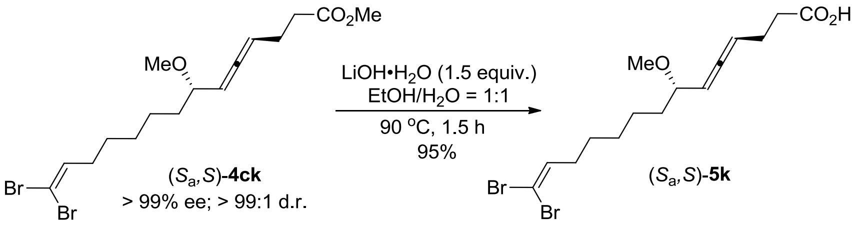

Following **Typical Procedure II**, the reaction of (S_a,S)-**4ck** (0.6575 g, 1.55 mmol), EtOH/H_2O = 1:1 by volume (pre-mixed by using 8 mL of H_2O and 8 mL of EtOH), and LiOH·H_2O (0.1031 g, 2.325 mmol) at 90 ^oC for 1.5 h afforded (S_a,S)-**5k** (0.6022 g, 95%) [eluent: petroleum ether (60-90 ^oC)/ethyl acetate = 10/1 (550 mL) to 8/1 (540 mL) to 5/1 (360 mL)] as an oil: $[\alpha]_D^{20}$ = +19.9 (c = 0.975, $CHCl_3$); ^1H NMR (300 MHz, $CDCl_3$) δ 10.12 (bs, 1H, COOH), 6.39 (t, J = 7.2 Hz, 1H, =CH), 5.30 (q, J = 6.1 Hz, 1H, =CH), 5.07-4.98 (m, 1H, =CH), 3.61 (q, J = 7.0 Hz, 1H, CH), 3.31 (s, 3H, CH_3), 2.49 (t, J = 7.5 Hz, 2H, CH_2), 2.40-2.29 (m, 2H, CH_2), 2.10 (q, J = 7.2 Hz, 2H, CH_2), 1.71-1.24 (m, 8H, CH_2 × 4); ^{13}C NMR (75 MHz, $CDCl_3$) δ 204.3, 178.9, 138.7, 93.5, 90.2, 88.5, 80.3, 56.0, 35.5, 33.1, 32.9, 28.9, 27.7, 25.1, 23.3; IR (neat) ν (cm^{-1}) 3031-3031 (COOH), 2928, 2856, 2662, 1964, 1713, 1417, 1338, 1257, 1202, 1157, 1108, 1084; MS (ESI) *m/z* 435 [M ($^{81}Br^{81}Br$) + Na]$^+$, 433 [M ($^{79}Br^{81}Br$) + Na]$^+$, 431 [M ($^{79}Br^{79}Br$) + Na]$^+$; HRMS (ESI) calcd. for $C_{15}H_{22}{}^{79}Br^{79}Br\ NaO_3{}^+$ [M ($^{79}Br^{79}Br$) + Na]$^+$: 430.9828, found: 430.9825.

Synthesis of (5*R*)-(10’,10’-dibromo-3’(*S*)-methoxy-1’(*E*),9’-decadien-1’-yl)dihydro-2(3*H*)-furanone (5*R*,1’*E*,3’*S*)-**6k**, i.e., **xestospongiene E** (reported as **xestospongiene F**) (zj-7-189)

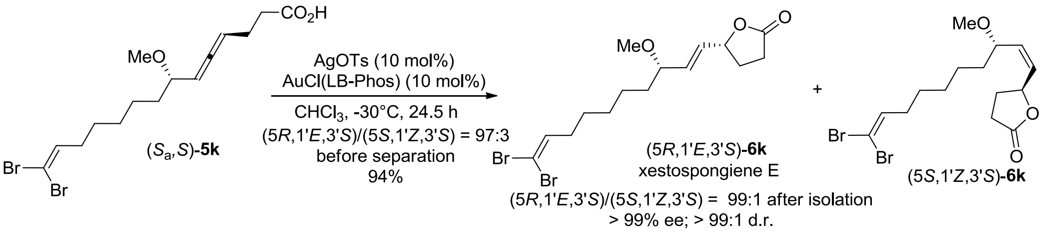

Following **Typical Procedure VIII**, the reaction of AgOTs (0.0285 g, 0.1 mmol, 98%), Au(LB-Phos)Cl (0.0597 g, 0.1 mmol), $CHCl_3$ (5 mL), and (*S*a,*S*)-**5k** (0.4102 g, 1.0 mmol)/$CHCl_3$ (5 mL) at -30 °C for 24.5 h afforded (5*R*,1’*E*,3’*S*)-**6k**,[20] **i.e., xestospongiene E** (0.3851 g, 94%, (5*R*,1’*E*,3’*S*)/(5*S*,1’*Z*,3’*S*) = 99:1 determined by ^{1}H NMR, > 99:1 d.r. determined by SFC analysis) [eluent: petroleum ether (60-90 °C)/ethyl acetate/dichloromethane = 150/10/1 (480 mL) to 100/10/1 (550 mL) to 80/10/1 (540 mL × 3)] ((5*R*,1’*E*,3’*S*)/(5*S*,1’Z,3’*S*) = 97:3 determined by ^{1}H NMR of crude product) as an oil: > 99% ee (SFC: Chiralcel AD-H column, *n*-hexane/*i*-PrOH = 9/1, 2.0 mL/min, λ = 214 nm, t_R (major) = 21.0 min, t_R (minor) = 11.6 min); $[\alpha]_D^{20}$ = -22.3 (c = 1.00, $CHCl_3$); $[\alpha]_D^{20}$ = -27.4 (c = 0.495, MeOH); (Lit.[20] $[\alpha]_D^{20}$ = +45.6 (c = 0.4, MeOH)); ^{1}H NMR (300 MHz, $CDCl_3$) δ 6.39 (t, *J* = 7.2 Hz, 1H, =CH), 5.79-5.59 (m, 2H, =CH × 2), 4.98 (q, *J* = 6.6 Hz, 1H, CH), 3.58 (q, *J* = 6.1 Hz, 1H, CH), 3.27 (s, 3H, CH_3), 2.62-2.38 (m, 3H, CH_2 + one proton of CH_2), 2.15-1.94 (m, 3H, CH_2 + one proton of CH_2), 1.67-1.16 (m, 8H, CH_2 × 4); the following signals are discernible for (5*S*,1’Z,3’*S*)-**6k**: δ 5.56-5.48 (m, 1H, =CH), 5.35-5.25 (m, 1H, CH); ^{13}C NMR (75 MHz, $CDCl_3$) δ 176.6, 138.5, 134.1, 129.5, 88.3, 80.8, 79.7, 56.2, 34.8, 32.6, 28.6, 28.5, 28.2, 27.4, 24.6; IR (neat) ν (cm^{-1}) 2977, 2931, 2857, 2821, 1777, 1460, 1421, 1326, 1296, 1215, 1176, 1112, 1091, 1012; MS (ESI): *m/z* 413 [M (^{81}Br^{81}Br) + H]$^+$, 411 [M (^{79}Br^{81}Br) + H]$^+$, 409 [M (^{79}Br^{79}Br) + H]$^+$.

e. Gram scale synthesis of (5*S*,1’*E*,3’*R*)-**6k** , i.e., **xestospongiene F**)

Synthesis of (*R*a,*R*)-methyl 14,14-dibromo-7-methoxy-4,5,13-tetradecatrienoate (*R*a,*R*)-**4ck** (zj-7-177)

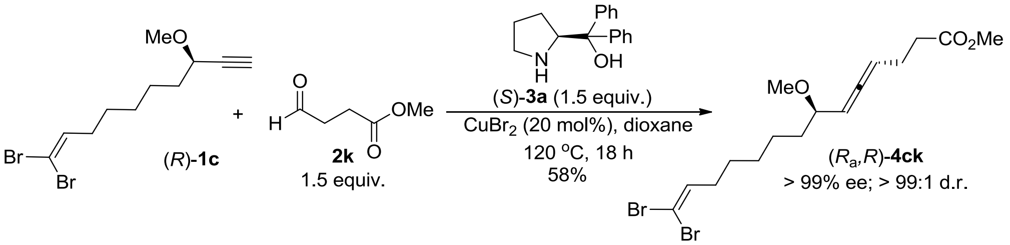

Following **Typical Procedure XIV** (four reactions in parallel), the reaction of $CuBr_2$ (0.0676 g, 0.3 mmol, 99%), (*S*)-**3a** (0.5803 g, 2.25 mmol, 98%), (*R*)-**1c** (0.4862 g, 1.5 mmol)/dioxane (8 mL), and **2k** (0.2609 g, 2.25 mmol) in dioxane/(5 mL) at 120 ^{o}C for 18 h; the reaction of $CuBr_2$ (0.0677 g, 0.3 mmol, 99%), (*S*)-**3a** (0.5804 g, 2.25 mmol, 98%), (*R*)-**1c** (0.4860 g, 1.5 mmol)/dioxane (8 mL), and **2k** (0.2620 g, 2.25 mmol)/dioxane (5 mL) at 120 ^{o}C for 18 h; the reaction of $CuBr_2$ (0.0677 g, 0.3 mmol, 99%), (*S*)-**3a** (0.5810 g, 2.25 mmol, 98%), (*R*)-**1c** (0.4859 g, 1.5 mmol)/dioxane (8 mL), and **2k** (0.2614 g, 2.25 mmol)/dioxane (5 mL) at 120 ^{o}C for 18 h; the reaction of $CuBr_2$ (0.0675 g, 0.3 mmol, 99%), (*S*)-**3a** (0.5810 g, 2.25 mmol, 98%), (*R*)-**1c** (0.4863 g, 1.5 mmol)/dioxane (8 mL), and **2k** (0.2613 g, 2.25 mmol)/dioxane (5 mL) at 120 ^{o}C for 18 h. The four reactions together afforded (R_a,*R*)-**4ck** (1.4779 g, 58%, > 99:1 d.r. determined by ^{1}H NMR and HPLC analysis) [eluent: petroleum ether (60-90 ^{o}C)/ethyl acetate = 50/1 (500 mL) to 30/1 (500 mL) to 20/1 (500 mL)] as a liquid: > 99% ee (HPLC conditions: Chiralcel IF column, *n*-hexane/*i*-PrOH = 100/1, 1.0 mL/min, λ = 214 nm, t_R (major) = 14.0 min, t_R (minor) = 15.0 min), $[\alpha]_D^{20}$ = -21.1 (c = 0.98, $CHCl_3$); ^{1}H NMR (300 MHz, $CDCl_3$) δ 6.39 (t, *J* = 7.2 Hz, 1H, =CH), 5.29 (q, *J* = 6.1 Hz, 1H, CH),5.04-4.95 (m, 1H, =CH), 3.68 (s, 3H, CH_3), 3.58 (q, *J* = 6.7 Hz, 1H, CH),, 3.30 (s, 3H, CH_3), 2.48-2.40 (m, 2H, CH_2), 2.38-2.28 (m, 2H, CH_2), 2.10 (q, *J* = 7.3 Hz, 2H, CH_2), 1.69-1.25 (m, 8H, CH_2 × 4); ^{13}C NMR (75 MHz, $CDCl_3$) δ 204.2, 173.2, 138.7, 93.3, 90.3, 88.5, 80.2, 56.0, 51.5, 35.6, 33.1, 32.8, 28.8, 27.6, 25.1, 23.6.

Synthesis of (R_a,*R*)-14,14-dibromo-7-methoxy-4,5,13-tetradecatrienoic acid (R_a,*R*)-**5k** (zj-7-182)

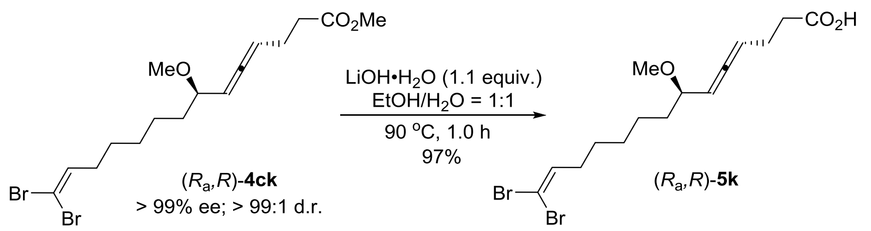

To a round-bottom flask were added (R_a,R)-**4ck** (1.2704 g, 3.0 mmol), EtOH/H_2O = 1:1 by volume (pre-mixed by using 15 mL of H_2O and 15 mL of EtOH) and LiOH·H_2O (0.1460 g, 3.3 mmol, 95%) sequentially. After stirring for 1 h under reflux at 90 ^oC, the reaction was complete as monitored by TLC. Then the mixture was cooled down to room temperature. After evaporation to remove EtOH, the aqueous layer was acidified with an aqueous solution of hydrochloric acid (aq., 3.0 M) until pH = 1. The aqueous layer was then extracted with Et_2O (30 mL × 3). The combined organic layer was washed with brine and dried over anhydrous Na_2SO_4, filtration, evaporation, and column chromatography on silica gel to afford (R_a,R)-**5k** (1.1945 g, 97%) [eluent: petroleum ether (60-90 ^oC)/ethyl acetate = 10/1 (550 mL) to 8/1 (540 mL) to 5/1 (600 mL)] as an oil: $[\alpha]_D^{20}$ = -20.6 (c = 1.01, $CHCl_3$); ^1H NMR (300 MHz, $CDCl_3$) δ 10.37 (bs, 1H, COOH), 6.39 (t, J = 7.4 Hz, 1H, =CH), 5.30 (q, J = 6.1 Hz, 1H, =CH), 5.07-4.98 (m, 1H, =CH), 3.60 (q, J = 6.7 Hz, 1H, CH), 3.31 (s, 3H, CH_3), 2.53-2.44 (m, 2H, CH_2), 2.39-2.28 (m, 2H, CH_2), 2.09 (q, J = 7.2 Hz, 2H, CH_2), 1.71-1.25 (m, 8H, CH_2 × 4); ^{13}C NMR (75 MHz, $CDCl_3$) δ 204.3, 178.9, 138.7, 93.4, 90.2, 88.5, 80.3, 56.0, 35.5, 33.1, 32.9, 28.8, 27.6, 25.1, 23.3.

Gram scale Synthesis of (5*S*)-(10’,10’-dibromo-3’(*R*)-methoxy-1’(*E*),9’-decadien-1’-yl)dihydro-2(3*H*)-furanone (5*S*,1’*E*,3’*R*)-**6k**, **xestospongiene F** (zj-8-011)

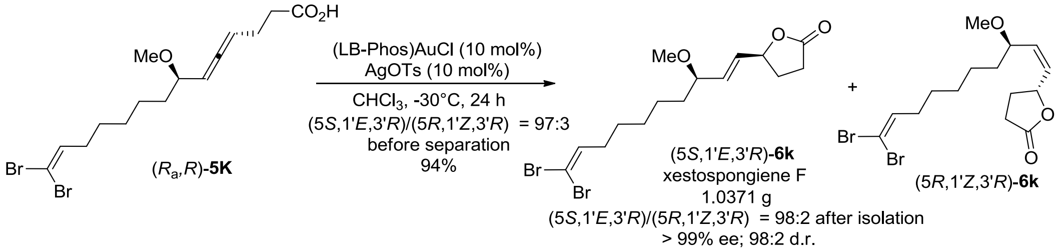

Following **Typical Procedure VIII**, the reaction of AgOTs (0.077 g, 0.27 mmol), Au(LB-Phos)Cl (0.1613 g, 0.27 mmol), $CHCl_3$ (17 mL), and (R_a,R)-**5k** (1.1054 g, 2.7

mmol)/$CHCl_3$ (10 mL) at -30 °C for 24 h afforded impure (5*S*,1'*E*,3'*R*)-**6k**[20] (1.0795 g) [eluent: petroleum ether (60-90 °C)/ethyl acetate/dichloromethane = 150/10/1 (480 mL) to 100/10/1 (550 mL) to 80/10/1 (540 mL × 3)] as an oil, which was then further purified by chromatography [eluent: petroleum ether (60-90 °C)/ethyl acetate/dichloromethane = 150/10/1 (480 mL) to 70/7/1 (550 mL) to 40/5/1 (540 mL) to 24/3/1 (540 mL) to 16/2/1 (540 mL)] to give (5*S*,1'*E*,3'*R*)-**6k**, **xestospongiene F** (1.0371 g, 94%, (5*S*,1'*E*,3'*R*)/(5*R*,1'*Z*,3'*R*) = 98:2 determined by ^{1}H NMR analysis, d.r. = 98:2 determined by SFC analysis) ((5*S*,1'*E*,3'*R*)/(5*R*,1'*Z*,3'*R*) = 97:3 determined by ^{1}H NMR of crude product) as an oil: 99% ee (SFC: Chiralcel AD-H column, *n*-hexane/*i*-PrOH = 9/1, 2.0 mL/min, λ = 214 nm, t_R (major) = 11.0 min, t_R (minor) = 21.8 min); $[\alpha]_D^{20}$ = +21.5 (c = 1.00, $CHCl_3$); $[\alpha]_D^{20}$ = +27.4 (c = 0.43, MeOH); (Lit.[20] $[\alpha]_D^{20}$ = -44.3 (c = 0.5, MeOH)); ^{1}H NMR (300 MHz, $CDCl_3$) δ 6.39 (t, *J* = 7.4 Hz, 1H, =CH), 5.84-5.57 (m, 2H, =CH × 2), 4.98 (q, *J* = 6.7 Hz, 1H, CH), 3.58 (q, *J* = 6.1 Hz, 1H, CH), 3.26 (s, 3H, CH_3), 2.62-2.37 (m, 3H, CH_2 + one proton of CH_2), 2.15-1.94 (m, 3H, CH_2 + one proton of CH_2), 1.66-1.20 (m, 8H, CH_2 × 4); the following signals are discernible for (5*R*,1'*Z*,3'*R*)-**6k**: δ 5.56-5.48 (m, 1H, =CH), 5.34-5.24 (m, 1H, CH); ^{13}C NMR (75 MHz, $CDCl_3$) δ 176.5, 138.5, 134.1, 129.4, 88.2, 80.7, 79.7, 56.1, 34.7, 32.6, 28.6, 28.4, 28.2, 27.3, 24.5.

Synthetic application in the synthesis of naturally occurring γ-alkylic γ-lactones

1. Synthesis of (*R*)-4-tetradecalactone

Synthesis of (R_a)-4,5-tetradecadienoic acid (R_a)-**5j** (zj-4-187,4-190)

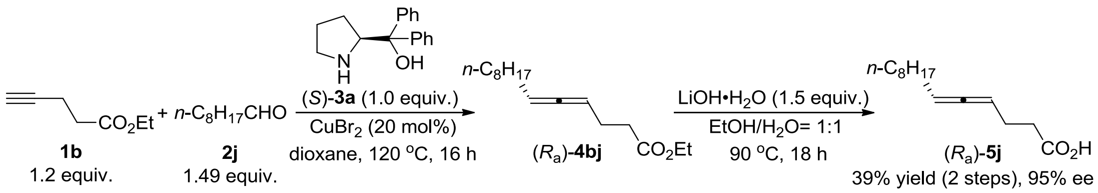

Following **Typical Procedure IV**, the reaction of $CuBr_2$ (0.6764 g, 3.0 mmol, 99%), (*S*)-**3a** (3.8705 g, 15 mmol, 98%), **1b** (2.2730 g, 18 mmol)/dioxane (15 mL), and nonanaldehyde **2j** (4.0 mL, d = 0.8277 g cm^{-3}, 3.1784 g, 22.38 mmol, 96%)/dioxane (10 mL) for 16 h afforded (R_a)-**4bj** (1.6250 g) [(eluent: petroleum ether

(60-90 °C)/diethyl ether = 100/1 (500 mL × 2) to 50/1 (400 mL)] as a liquid, which was then submitted to next step.

Following **Typical Procedure V**, the reaction of (*R*a)-**4bj** (1.6250 g, 6.45 mmol, prepared above), EtOH/H_2O = 1:1 by volume (pre-mixed by using 25 mL of H_2O and 25 mL of EtOH), and LiOH·H_2O (0.4290 g, 9.675 mmol, 95%) for 18 h afforded (*R*a)-**5j**[21] (1.3001 g, 39%, 2 steps) [(eluent: petroleum ether (60-90 °C)/ethyl acetate = 20/1 (440 mL) to 10/1 (550 mL × 3) to 5/1 (600 mL)] as a liquid: 95% ee (determined by the corrsponding esterification product (*R*a)-**4bj**); $[\alpha]_D^{20}$ = -65.3 (*c* = 1.025, $CHCl_3$); ^{1}H NMR (300 MHz, $CDCl_3$) δ 10.35 (bs, 1H, COOH), 5.21-5.11 (m, 2H, =CH × 2), 2.48 (t, *J* = 7.2 Hz, 2H, CH_2), 2.36-2.24 (m, 2H, CH_2); 2.02-1.91 (m, 2H, CH_2), 1.44-1.20 (m, 12H, CH_2 × 6), 0.88 (t, *J* = 6.8 Hz, 3H, CH_3); ^{13}C NMR (75 MHz, $CDCl_3$) δ 203.6, 179.8, 92.9, 89.2, 33.1, 31.9, 29.4, 29.3, 29.15, 29.10, 28.8, 23.5, 22.6, 14.1; IR (neat) ν (cm^{-1}) 3666-2167 (COOH), 3031, 2953, 2925, 2855, 2664, 1964, 1712, 1435, 1411, 1378, 1336, 1279, 1250, 1211, 1173; MS (70 ev, EI) *m/z* (%) 224 (M^+, 8.53), 126 (100), 84 (100), 81 (100).

Esterification for determination of the ee value of (*R*a)-**5j**: synthesis of (*R*a)-ethyl 4,5-tetradeca dienoate (*R*a)-**4bj** (zj-4-198)

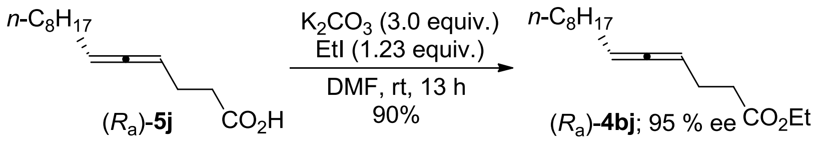

Following **Typical Procedure III**, the reaction of (*R*a)-**5j** (0.1345 g, 0.6 mmol), EtI (0.06 mL, d = 1.94 g cm^{-3}, 0.1152 g, 0.74 mmol, 99%), K_2CO_3 (0.2512 g, 1.8 mmol), and DMF (6 mL) for 13 h afforded (*R*a)-**4bj** (0.1358 g, 90%) [eluent: petroleum ether (60-90 °C)/ethyl acetate = 100/1 (600 mL)] as a liquid: 95% ee (HPLC conditions: Chiralcel OZ-H column, *n*-hexane/*i*-PrOH = 400:1, 0.5 mL/min, λ = 214 nm, t_R (major) = 15.4 min, t_R (minor) = 14.1 min); $[\alpha]_D^{20}$ = -60.1 (*c* = 1.00, $CHCl_3$); ^{1}H NMR (300 MHz, $CDCl_3$) δ 5.18-5.09 (m, 2H, =CH × 2), 4.13 (q, *J* = 7.0 Hz, 2H, OCH_2), 2.46-2.37 (m, 2H, CH_2), 2.34-2.23 (m, 2H, CH_2), 2.02-1.90 (m, 2H, CH_2), 1.44-1.21 (m, 15H, CH_3 + CH_2 × 6), 0.88 (t, *J* = 6.8 Hz, 3H, CH_3); ^{13}C NMR

(75 MHz, $CDCl_3$) δ 203.6, 173.1, 92.5, 89.5, 60.2, 33.4, 31.8, 29.4, 29.3, 29.1, 28.8, 23.9, 22.6, 14.2, 14.1; IR (neat) ν (cm^{-1}) 2956, 2926, 2854, 1963, 1739, 1465, 1447, 1420, 1372, 1350, 1300, 1250, 1159, 1097, 1055, 1037; MS (70 ev, EI) *m/z* (%) 252 (M^+, 4.90), 80 (100); HRMS calcd for $C_{16}H_{28}O_2$ [M^+]: 252.2089, found: 252.2085.

Synthesis of (*S*,*E*)-5-(1-decenyl)dihydro-2(3*H*)-furanone (*S*,*E*)-**6j** (zj-5-017)

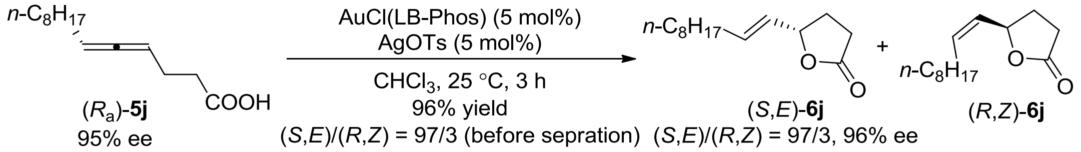

Following **Typical Procedure VI**, the reaction of AgOTs (0.0142 g, 0.05 mmol, 98%), Au(LB-Phos)Cl (0.0299 g, 0.05 mmol), $CHCl_3$ (5 mL), and (R_a)-**5j** (0.2240 g, 1.0 mmol)/$CHCl_3$ (5 mL) for 3.0 h afforded (*S*,*E*)-**6j**[11] (0.2158 g, 96%, (*S*,*E*)/(*R*,*Z*) = 97/3 determined by ^{1}H NMR) [eluent: petroleum ether (60-90 °C)/ethyl acetate = 10/1 (220 mL) to 8/1 (450 mL)] ((*S*,*E*)/(*R*,*Z*) = 97/3 determined by ^{1}H NMR of crude product) as an oil with pleasant flavor: 96% ee (HPLC conditions: Chiralcel OJ-H column, *n*-hexane/*i*-PrOH = 100:1, 1.0 mL/min, λ = 214 nm, t_R(major) = 15.4 min, t_R(minor) = 13.5 min; $[\alpha]_D^{20}$ = +27.3 (*c* = 0.99, $CHCl_3$); (Lit.[11] $[\alpha]_D^{26}$ = +30.2 (c = 2.12, $CHCl_3$)); ^{1}H NMR (300 MHz, $CDCl_3$) δ 5.81 (dtd, J_1 = 15.3 Hz, J_2 = 6.6 Hz, J_3 = 0.9 Hz, 1H, =CH), 5.49 (ddt, J_1 = 15.3 Hz, J_2 = 7.2 Hz, J_3 = 1.1 Hz, 1H, =CH), 4.89 (q, *J* = 7.2 Hz, 1H, CH), 2.58-2.48 (m, 2H, CH_2), 2.46-2.31 (m, 1H, one proton from CH_2), 2.11-1.89 (m, 3H, CH_2 + one proton from CH_2), 1.47-1.18 (m, 12H, CH_2 × 6), 0.88 (t, *J* = 6.6 Hz, 3H, CH_3); the following signals are discernible for (*R*,*Z*)-**6j**: δ 5.71-5.61 (m, 1H, =CH), 5.31-5.21 (m, 1H, CH); ^{13}C NMR (75 MHz, $CDCl_3$) δ 176.8, 135.2, 127.2, 80.8, 31.8, 31.5, 29.1, 28.9, 28.8, 28.5, 28.45, 28.38, 22.3, 13.8; IR (neat) ν (cm^{-1}) 2956, 2925, 2854, 1778, 1673, 1460, 1423, 1378, 1327, 1299, 1216, 1176, 1123, 1011; GC-MS (GC condition: injector: 280 °C; column: DB5 column 30 m × 0.25 mm, temperature programming: 60 °C (2 min), 20 °C/min to 280 °C, 280 °C (30 min); detector: 280 °C) (70 ev, EI) *m/z* (%) for (*S*,*E*)-**6j**: t_R (major) = 4.03 min: 224 (M^+, 0.94), 111 (100); for (*R*,*Z*)-**6j** t_R (minor) = 3.98 min: 224 (M^+, 1.27), 111 (100).

Synthesis of (*R*)-5-decyldihydro-2(3*H*)-furanone (*R*)-**4-tetradecalactone**[4] (zj-5-093)

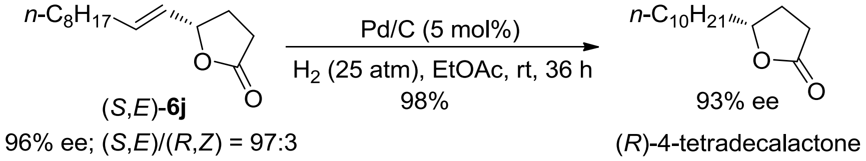

Typical Procedure XV: To a reaction tube were added (*S*,*E*)-**6j** (0.1122 g, 0.5 mmol), EtOAc (3 mL), and Pd/C (10% on C, dry, 0.0266 g, 0.025 mmol) sequentially. Then the reaction tube was placed in an autoclave. The resulting mixture was stirred under H_2 (25 atm) at room temperature for 36 h and then filtered through a short column of silica gel with Et_2O (15 mL × 3). After evaporation, the residue was purified by chromatography [eluent: petroleum ether (60-90 ^oC)/ethyl acetate = 10/1 (550 mL)] on silica gel to afford (*R*)-4-tetradecalactone[22] (0.1109 g, 98%) as an oil with pleasant flavor: 93% ee (GC conditions: CP-Chirasil-DEX CB column (25 m × 0.25 mm × 0.25 μm); carrier = He; injector = 145 ^oC; detector (FID, H_2) = 200 ^oC; oven temperature = 140 ^oC to 200 ^oC (5 ^oC/min); t_R(major) = 61.3 min, t_R(minor) = 62.9 min; $[\alpha]_D^{20}$ = +28.5 (*c* = 0.99, $CHCl_3$); (Lit.[11] $[\alpha]_D^{26}$ = +29.95 (c = 2.739, $CHCl_3$)); ^1H NMR (300 MHz, $CDCl_3$) δ 4.54-4.44 (m, 1H, CH), 2.59-2.48 (m, 2H, CH_2), 2.39-2.26 (m, 1H, one proton from CH_2), 1.93-1.78 (m, 1H, one proton from CH_2), 1.78-1.67 (m, 1H, one proton from CH_2), 1.67-1.53 (m, 1H, one proton from CH_2), 1.51-1.18 (m, 16 H, CH_2 × 8), 0.88 (t, *J* = 6.8 Hz, 3H, CH_3); ^{13}C NMR (75 MHz, $CDCl_3$) δ 177.2, 80.9, 35.4, 31.7, 29.43, 29.39, 29.3, 29.20, 29.17, 28.7, 27.9, 25.1, 22.5, 14.0; IR (neat) ν (cm^{-1}) 2925, 2854, 1779, 1463, 1420, 1376, 1352, 1285, 1216, 1178, 1127, 1019; MS (70 ev, EI) *m/z* (%) 227 (M^+ + 1, 34.74), 226 (M^+, 0.63), 85 (100).

2. Synthesis of (*S*)-4-tetradecalactone

Synthesis of (S_a)-4,5-tetradecadienoic acid (S_a)-**5j** (zj-4-186,4-189)

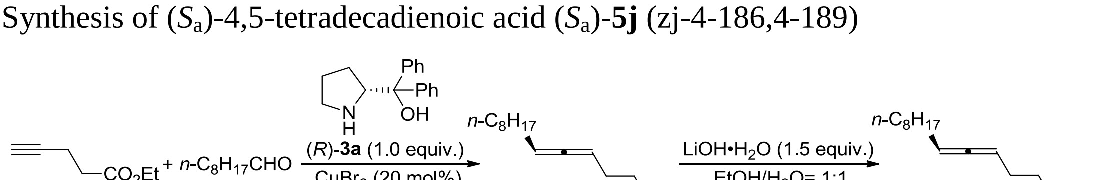

Following **Typical Procedure IV**, the reaction of $CuBr_2$ (0.6764 g, 3.0 mmol,

99%), (*R*)-**3a** (3.8711 g, 15 mmol), **1b** (2.2745 g, 18 mmol)/dioxane (15 mL), and nonanaldehyde **2j** (4.0 mL, d = 0.8277 g cm^{-3}, 3.1784 g, 22.38 mmol)/dioxane (10 mL) for 16 h afforded (S_a)-**4bj** (1.6104 g) [(eluent: petroleum ether (60-90 ^oC)/diethyl ether = 100/1 (500 mL × 2) to 50/1 (400 mL)] as a liquid, which was then submitted to next step without further characterization.

Following **Typical Procedure V**, the reaction of (S_a)-**4bj** (1.6104 g, 6.4 mmol, prepared above), EtOH/H_2O = 1:1 by volume (pre-mixed by using 25 mL of H_2O and 25 mL of EtOH), and LiOH·H_2O (0.4240 g, 9.6 mmol, 95%) for 18 h afforded (S_a)-**5j**[21] (1.2931 g, 39%, 2 steps) [(eluent: petroleum ether (60-90 ^oC)/ethyl acetate = 20/1 (420 mL) to 10/1 (550 mL × 3) to 5/1 (600 mL)] as an oil: 97% ee (determined by the corrsponding esterification product (S_a)-**4bj**); $[\alpha]_D^{20}$ = +66.5 (*c* = 1.005, $CHCl_3$); ^1H NMR (300 MHz, $CDCl_3$) δ 10.15 (bs, 1H, COOH), 5.22-5.10 (m, 2H, =CH × 2), 2.48 (t, *J* = 7.4 Hz, 2H, CH_2), 2.38-2.23 (m, 2H, CH_2), 2.03-1.90 (m, 2H, CH_2), 1.45-1.18 (m, 12H, CH_2 × 6), 0.88 (t, *J* = 6.8 Hz, 3H, CH_3); ^{13}C NMR (75 MHz, $CDCl_3$) δ 203.6, 179.7, 92.9, 89.2, 33.1, 31.9, 29.4, 29.3, 29.14, 29.10, 28.8, 23.5, 22.6, 14.1; IR (neat) ν (cm^{-1}) 3728-2188 (COOH), 3033, 2956, 2925, 2854, 2667, 1964, 1713, 1436, 1412, 1378, 1336, 1278, 1249, 1211, 1180, 1042, 1075; MS (70 ev, EI) *m/z* (%) 224 (M^+, 6.98), 126 (100), 81 (100).

Esterification for determination of the ee value of (S_a)-**5j**: synthesis of (S_a)-ethyl 4,5-tetradeca dienoate (S_a)-**4bj** (zj-4-199)

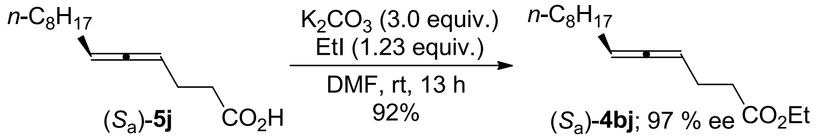

Following **Typical Procedure III**, the reaction of (S_a)-**5j** (0.1340 g, 0.6 mmol), EtI (0.06 mL, d = 1.94 g cm^{-3}, 0.1152 g, 0.74 mmol, 99%), K_2CO_3 (0.2507 g, 1.8 mmol), and DMF (6 mL) for 13 h afforded (S_a)-**4bj** (0.1385 g, 92%) [eluent: petroleum ether (60-90 ^oC)/ethyl acetate = 100/1 (600 mL)] as a liquid: 97% ee (HPLC conditions: Chiralcel OZ-H column, *n*-hexane/*i*-Pr = 400:1, 0.5 mL/min, λ = 214 nm, t_R (major) = 8.9 min, t_R (minor) = 9.4 min); $[\alpha]_D^{20}$ = + 61.6 (*c* = 0.995, $CHCl_3$) ^1H NMR (300 MHz,

$CDCl_3$) δ 5.18-5.09 (m, 2H, =CH × 2), 4.13 (q, J = 7.2 Hz, 2H, OCH_2), 2.46-2.37 (m, 2H, CH_2), 2.35-2.24 (m, 2H, CH_2), 2.02-1.91 (m, 2H, CH_2), 1.45-1.18 (m, 15H, CH_3 + CH_2 × 6), 0.88 (t, J = 6.8 Hz, 3H, CH_3); ^{13}C NMR (75 MHz, $CDCl_3$) δ 203.6, 172.9, 92.3, 89.4, 60.1, 33.3, 31.8, 29.3, 29.2, 29.0, 28.8, 23.8, 22.5, 14.1, 13.9; IR (neat) ν (cm^{-1}) 2957, 2926, 2855, 1963, 1739, 1464, 1418, 1372, 1349, 1300, 1249, 1159, 1097, 1038; MS (70 ev, EI) *m/z* (%) 252 (M^+, 6.68), 80 (100); HRMS calcd for $C_{16}H_{28}O_2$ [M^+]: 252.2089, found: 252.2087.

Synthesis of (*S*)-5-decyldihydro-2(3*H*)-furanone (*S*)-**4-tetradecalactone** (zj-5-090)

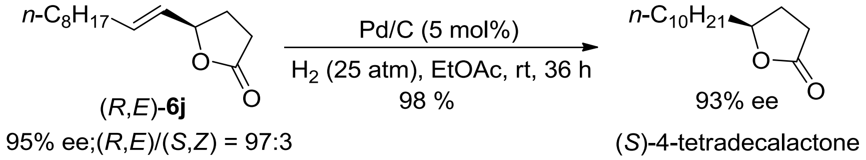

Following **Typical Procedure XV**, the reaction of (*R*,*E*)-**6j** (0.1121 g, 0.5 mmol, prepared above, see page 35)/EtOAc (3 mL) and Pd/C (10% on C, dry, 0.0266 g, 0.025 mmol) for 36 h afforded (*S*)-4-tetradecalactone[11] (0.1113 g, 98%) [eluent: petroleum ether (60-90 °C)/ethyl acetate = 10/1 (550 mL)] as an oil with pleasant flavor: 93% ee (GC conditions: CP-Chirasil-DEX CB column (25 m × 0.25 mm × 0.25 μm); carrier: He; injector: 145 °C; detector (FID, H_2): 200 °C; oven temperature: 140 °C to 200 °C (5 °C/min); t_R(major) = 62.7 min, t_R(minor) = 61.4 min; $[\alpha]_D^{20}$ = -28.6 (c = 1.01, $CHCl_3$); (Lit.[11] $[\alpha]_D^{26}$ = -36.76 (c = 2.4, $CHCl_3$)); ^1H NMR (300 MHz, $CDCl_3$) δ 4.55-4.44 (m, 1H, =CH), 2.61-2.50 (m, 2H, CH_2), 2.40-2.26 (m, 1H, one proton from CH_2), 1.95-1.79 (m, 1H, one proton from CH_2), 1.79-1.67 (m, 1H, one proton from CH_2), 1.66-1.53 (m, 1H, one proton from CH_2), 1.51-1.17 (m, 16 H, CH_2 ×8), 0.88 (t, J = 6.8 Hz, 3H, CH_3); ^{13}C NMR (75 MHz, $CDCl_3$) δ 177.2, 80.9, 35.5, 31.7, 29.43, 29.39, 29.3, 29.20, 29.17, 28.7, 27.9, 25.1, 22.5, 14.0; IR (neat) ν (cm^{-1}) 2956, 2922, 2853, 1754, 1471, 1425, 1384, 1186, 1023, 1008; MS (70 ev, EI) *m/z* (%) 227 (M^+ + 1, 49.33), 226 (M^+, 0.88), 85 (100).

3. Synthesis of (*R*)-γ-palmitolactone

Synthesis of (R_a)-4,5- hexadecadienoic acid (R_a)-**5l** (zj-3-166,3-171)

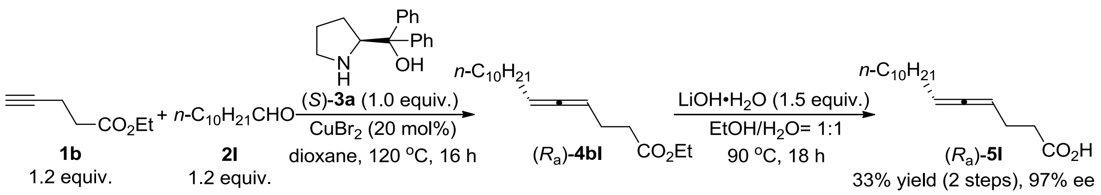

Following **Typical Procedure IV**, the reaction of $CuBr_2$ (0.6766 g, 3.0 mmol, 99%), (*S*)-**3a** (3.8771 g, 15 mmol, 98%), **1b** (2.2785 g, 18 mmol)/dioxane (20 mL), and undecanylaldehyde **2l** (3.8 mL, d = 0.825 g cm^{-3}, 3.135 g, 17.9 mmol)/dioxane (5 mL) for 16 h afforded (R_a)-**4bl** (1.6328 g) [(eluent: petroleum ether (60-90 °C)/diethyl ether = 100/1 (500 mL × 2) to 50/1 (500 mL × 2) to 20/1 (210 mL)] as a liquid, which was then submitted to next step.

Following **Typical Procedure V**, the reaction of (R_a)-**4bl** (1.6328 g, 5.83 mmol, prepared above), $EtOH/H_2O$ = 1:1 by volume (pre-mixed by using 29 mL of H_2O and 29 mL of EtOH), and LiOH·H_2O (0.3869 g, 8.745 mmol, 95%) for 18 h afforded (R_a)-**5l** (1.2436 g, 33%, 2 steps) [(eluent: petroleum ether (60-90 °C)/ethyl acetate = 20/1 (500 mL) to 10/1 (500 mL × 3) to 5/1 (360 mL)] as a liquid: 97% ee (determined by the corrsponding esterification product (R_a)-**4al**); $[\alpha]_D^{20}$ = -59.8 (*c* = 0.99, $CHCl_3$); ^{1}H NMR (300 MHz, $CDCl_3$) δ 10.25 (bs, 1H, COOH), 5.20-5.11 (m, 2H, =CH × 2), 2.48 (t, *J* = 6.9 Hz, 2H, CH_2), 2.36-2.24 (m, 2H, CH_2); 2.02-1.91 (m, 2H, CH_2), 1.44-1.17 (m, 16H, CH_2 × 8), 0.88 (t, *J* = 6.8 Hz, 3H, CH_3); ^{13}C NMR (75 MHz, $CDCl_3$) δ 203.6, 179.7, 92.9, 89.2, 33.1, 31.9, 29.64, 29.61, 29.5, 29.3, 29.15, 29.11, 28.8, 23.5, 22.7, 14.1; IR (neat) ν (cm^{-1}) 3558-2221 (COOH), 3037, 2956, 2925, 2854, 2664, 1963, 1712, 1436, 1412, 1377, 1334, 1279, 1249, 1211, 1173, 1046, 1017; MS (70 ev, EI) *m/z* (%) 252 (M^+, 5.43), 126 (100); HRMS calcd. for $C_{16}H_{28}O_2$ [M + H^+]: 252.2089, found: 252.2088.

Esterification for determination of the ee value of (R_a)-**5l**: synthesis of (R_a)-ethyl 4,5-hexadeca dienoate (R_a)-**4al** [5](zj-3-184)

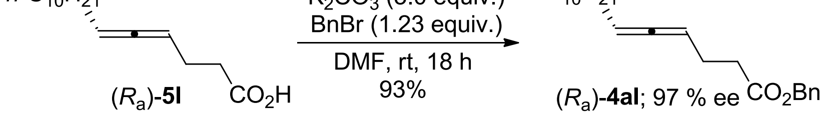

To a Schlenk tube were added (R_a)-**5l** (0.0505 g, 0.2 mmol, prapared above) and DMF (2 mL). Then K_2CO_3 (0.0830 g, 0.6 mmol) and BnBr (0.03 mL, d = 1.43 g cm^{-3}, 0.042 g, 0.246 mmol, 98%) were added sequentially. After continuous stirring at room temperature for 18 h, the reaction was complete as monitored by TLC. After quenching with water (10 mL) and being stirred for 10 min, the aqueous solution was extracted with Et_2O (10 mL × 3). The combined organic layer was washed with H_2O and brine, and dried over anhydrous Na_2SO_4. Filtration, evaporation, and column chromatography on silica gel gave (R_a)-**4al** (0.0635 g, 93%) [eluent: petroleum ether (60-90 ^{o}C)/ethyl acetate = 100/1 (500 mL)] as a liquid: 97% ee (HPLC conditions: Chiralcel IA column, *n*-hexane, 0.8 mL/min, λ = 214 nm, t_R (major) = 20.0 min, t_R (minor) = 22.4 min); $[\alpha]_D^{20}$ = -49.8 (*c* = 1.00, $CHCl_3$); ^{1}H NMR (300 MHz, $CDCl_3$) δ 7.33-7.20 (m, 5H, ArH), 5.11-4.97 (m, 4H, =CH × 2 + CH_2), 2.44-2.35 (m, 2H, CH_2), 2.29-2.19 (m, 2H, CH_2), 1.92-1.80 (m, 2H, CH_2), 1.34-1.11 (m, 16H, CH_2 × 8), 0.80 (t, *J* = 6.6 Hz, 3H, CH_3); ^{13}C NMR (75 MHz, $CDCl_3$) δ 203.6, 172.9, 136.0, 128.5, 128.1, 92.6, 89.4, 66.1, 33.4, 31.9, 29.6, 29.4, 29.3, 29.1, 28.8, 23.8, 22.7, 14.1; IR (neat) ν (cm^{-1}) 3090, 3066, 3033, 2956, 2925, 2853, 1962, 1740, 1498, 1455, 1379, 1353, 1153; MS (70 ev, EI) *m/z* (%) 343 (M^+ + 1, 7.75), 342 (M^+, 4.17), 91 (100); HRMS calcd for $C_{23}H_{34}O_2$ [M^+]: 342.2559, found: 342.2552.

Synthesis of (*S*,*E*)-5-(1-dodecenyl)dihydro-2(3*H*)-furanone (*S*,*E*)-**6l** (zj-3-181)

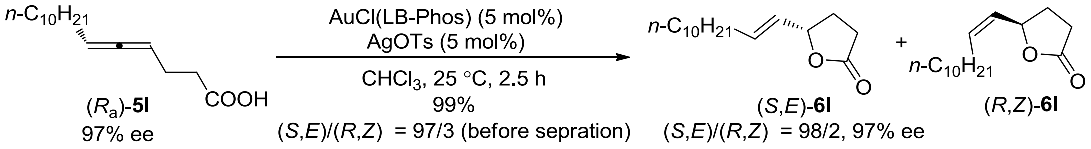

Following **Typical Procedure VI**, the reaction of AgOTs (0.0142 g, 0.05 mmol, 98%), Au(LB-Phos)Cl (0.0299 g, 0.05 mmol), $CHCl_3$ (5 mL), and (R_a)-**5l** (0.2524 g, 1.0 mmol)/$CHCl_3$ (5 mL) for 2.5 h afforded (*S*,*E*)-**6l** (0.2492 g, 99%, (*S*,*E*)/(*R*,*Z*) = 98/2 determined by ^{1}H NMR) [eluent: petroleum ether (60-90 ^{o}C)/ethyl acetate = 10/1 (250 mL) to 8/1 (400 mL)] ((*S*,*E*)/(*R*,*Z*) = 97/3 determined by ^{1}H NMR of crude product) as an oil with pleasant flavor: 97% ee (HPLC conditions: Chiralcel AS-H column, hexane/*i*-PrOH = 95:5, 1.0 mL/min, λ = 214 nm, t_R(major) = 11.8 min,

t_R(minor) = 10.6 min; $[\alpha]_D^{20}$ = +24.2 (c = 1.005, $CHCl_3$); ^{1}H NMR (300 MHz, $CDCl_3$) δ 5.80 (dt, J_1 = 15.3 Hz, J_2 = 7.1 Hz, 1H, =CH), 5.49 (ddt, J_1 = 15.0 Hz, J_2 = 7.1 Hz, J_3 = 1.3 Hz, 1H, =CH), 4.89 (q, J = 6.8 Hz, 1H, CH), 2.57-2.48 (m, 2H, CH_2), 2.43-2.31 (m, 1H, one proton from CH_2), 2.12-1.90 (m, 3H, CH_2 + one proton from CH_2), 1.46-1.17 (m, 16H, CH_2 × 8), 0.88 (t, J = 6.8 Hz, 3H, CH_3); the following signals are discernible for (*R*,*Z*)-**6l**: δ 5.71-5.61 (m, 1H, =CH), 5.30-5.21 (m, 1H, CH); ^{13}C NMR (75 MHz, $CDCl_3$) δ 176.8, 135.3, 127.2, 80.9, 31.8, 31.6, 29.33, 29.30, 29.2, 29.1, 28.8, 28.6, 28.5, 28.4, 22.4, 13.8; IR (neat) ν (cm^{-1}) 2925, 2854, 1778, 1672, 1462, 1421, 1376, 1325, 1300, 1216, 1176, 1123, 1009; GC-MS (GC condition: injector: 280 °C; column: DB5 column 30 m × 0.25 mm, temperature programming: 60 °C (2 min), 20 °C/min to 280 °C, 280 °C (30 min); detector: 280 °C) (70 ev, EI) *m/z* (%) for (*S*,*E*)-**6l**: t_R (major) = 7.8 min: 252 (M^+, 1.16), 111 (100); for (*R*,*Z*)-**6l** t_R (minor) = 7.6 min: 252 (M^+, 3.23), 111 (100); HRMS calcd. for $C_{16}H_{28}O_2$ [M^+]: 252.2089, found: 252.2086.

Synthesis of (*R*)-5-dodecyldihydro-2(3*H*)-furanone (*R*)-**γ-palmitolactone** (zj-5-044)

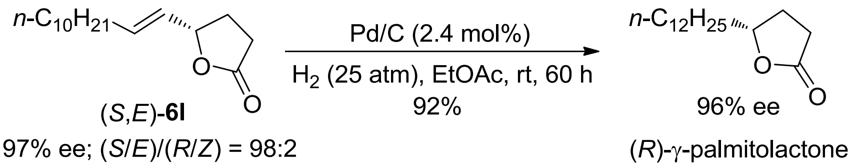

Following **Typical Procedure XV**, the reaction of (*S*,*E*)-**6l** (0.1261 g, 0.5 mmol)/EtOAc (4 mL), Pd/C (0.0128 g, 10% on C, dry, 0.012 mmol), and EtOAc (3 mL) for 60 h afforded (*R*)-γ-palmitolactone[23] (0.1175 g, 92%) [eluent: petroleum ether (60-90 °C)/ethyl acetate = 10/1 (850 mL)] as an oil with pleasant flavor: 96% ee (GC conditions: CP-Chirasil-DEX CB column (25 m × 0.25 mm × 0.25 μm); carrier: He; injector: 145 °C; detector (FID, H_2): 200 °C; oven temperature: 130 °C to 200 °C (5 °C/min); t_R (major) = 121.3 min, t_R (minor) = 124.2 min; $[\alpha]_D^{20}$ = +26.9 (c = 0.99, $CHCl_3$); ^{1}H NMR (300 MHz, $CDCl_3$) δ 4.54-4.43 (m, 1H, CH), 2.57-2.48 (m, 2H, CH_2), 2.38-2.26 (m, 1H, one proton from CH_2), 1.92-1.81 (m, 1H, one proton from CH_2), 1.79-1.67 (m, 1H, one proton from CH_2), 1.65-1.53 (m, 1H, one proton from

CH_2), 1.50-1.18 (m, 20H, CH_2 × 10), 0.88 (t, *J* = 6.8 Hz, 3H, CH_3); ^{13}C NMR (75 MHz, $CDCl_3$) δ 177.1, 80.9, 35.5, 31.8, 29.52, 29.50, 29.4, 29.3, 29.2, 28.7, 27.9, 25.1, 22.5, 14.0; IR (neat) ν (cm^{-1}) 2925, 2854, 1780, 1462, 1421, 1376, 1352, 1285, 1216, 1177, 1127, 1021; MS (70 ev, EI) *m/z* (%) 255 (M^+ + 1, 60.83), 254 (M^+, 1.65), 85 (100).

4. Gram Scale Synthesis of (*R*)-4-Decalactone

Synthesis of (R_a)-4,5-decadienoic acid (R_a)-**5b** (zj-6-108,6-111)

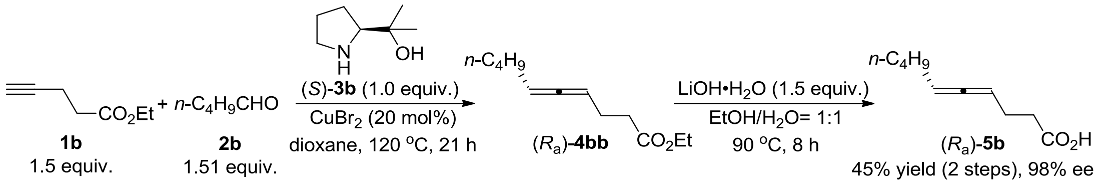

Following **Typical Procedure I**, the reaction of $CuBr_2$ (1.3514 g, 6.0 mmol, 99%), (*S*)-2-(pyrrolidin-2-yl)propan-2-ol (*S*)-**3b** (3.8415 g, 30 mmol), **1b** (5.6719 g, 45 mmol)/dioxane (60 mL), and **2b** (4.8 mL, d = 0.81 g cm^{-3}, 3.888 g, 45.21 mmol)/dioxane (30 mL) afforded (R_a)-**4bb** (3.0621 g) [(eluent: petroleum ether (60-90 °C)/diethyl ether = 100/1 (500 mL × 2) to 50/1 (500 mL) to 30/1 (500 mL)] as a liquid, which was then submitted to next step.

Following **Typical Procedure V**, the reaction of (R_a)-**4bb** (3.0621 g, 15.6 mmol, prepared above), EtOH/H_2O = 1:1 by volume (pre-mixed by using 35 mL of H_2O and 35 mL of EtOH), and LiOH·H_2O (1.0352 g, 23.4 mmol, 95%) afforded (R_a)-**5b** (2.2406 g, 45%, 2 steps) [(eluent: petroleum ether (60-90 °C)/ethyl acetate = 20/1 (420 mL) to 15/1 (480 mL) to 10/1 (660 mL)] as a liquid: 98% ee (determined by the corrsponding esterification product (R_a)-**4bb**); $[\alpha]_D^{20}$ = -77.7 (*c* = 0.99, $CHCl_3$); ^1H NMR (300 MHz, $CDCl_3$) δ 11.55 (bs, 1H, COOH), 5.21-5.11 (m, 2H, =CH × 2), 2.48 (t, *J* = 7.4 Hz, 2H, CH_2), 2.36-2.22 (m, 2H, CH_2), 2.05-1.90 (m, 2H, CH_2), 1.46-1.25 (m, 4H, CH_2 × 2), 0.90 (t, *J* = 7.2 Hz, 3H, CH_3); ^{13}C NMR (75 MHz, $CDCl_3$) δ 203.6, 180.0, 92.8, 89.2, 33.1, 31.2, 28.5, 23.4, 22.1, 13.8.

Esterification for determination of the ee value of (R_a)-**5b**: synthesis of (R_a)-ethyl 4,5-decadienoate (R_a)-**4bb** (zj-6-116)

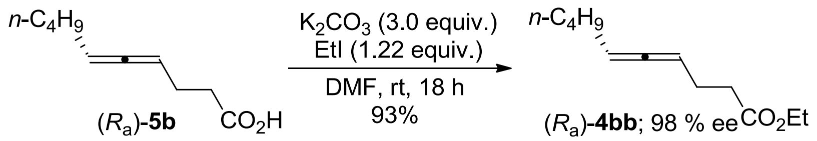

Following **Typical Procedure III**, the reaction of (R_a)-**5b** (0.0831 g, 0.5 mmol)/DMF (5 mL), K_2CO_3 (0.2072 g, 1.5 mmol), and EtI (0.05 mL, d = 1.94 g cm^{-3}, 0.0951 g, 0.61 mmol, 98%) for 18 h afforded (R_a)-**4bb** (0.0905 g, 93%) [eluent: petroleum ether (60-90 ^oC)/ethyl acetate = 100/1 (500 mL) to 50/1 (300 mL)] as a liquid: 98% ee (HPLC conditions: Chiralcel OZ-H column, *n*-hexane, 1.0 mL/min, λ = 214 nm, t_R (major) = 27.1 min, t_R (minor) = 24.5 min); $[\alpha]_D^{20}$ = -69.6 (c = 1.04, $CHCl_3$); ^1H NMR (300 MHz, $CDCl_3$) δ 5.20-5.08 (m, 2H, =CH × 2), 4.13 (q, J = 7.2 Hz, 2H, OCH_2), 2.47-2.37 (m, 2H, CH_2), 2.35-2.24 (m, 2H, CH_2), 2.04-1.92 (m, 2H, CH_2), 1.43-1.30 (m, 4H, CH_2 × 2), 1.26 (t, J = 7.1 Hz, 3H, CH_3) 0.90 (t, J = 6.9 Hz, 3H, CH_3); ^{13}C NMR (75 MHz, $CDCl_3$) δ 203.7, 173.0, 92.4, 89.4, 60.1, 33.4, 31.2, 28.5, 23.8, 22.1, 14.1, 13.8.

Gram Scale Synthesis of (*R*)-5-hexyldihydro-2(3*H*)-furanone (*R*)-**4-Decalactone** (zj-6-170)

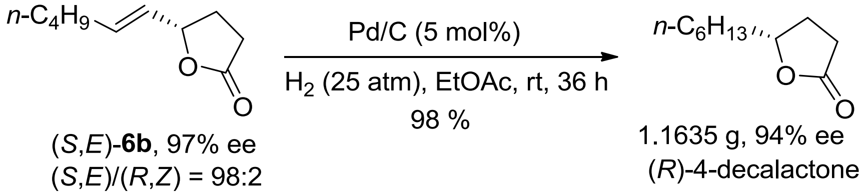

Following **Typical Procedure XV**, the reaction of (*S*,*E*)-**6b** (1.1772 g, 7.0 mmol, prepared above, see page 36)/EtOAc (20 mL), and Pd/C (10% on C, dry, 0.3724 g, 0.35 mmol) for 36 h afforded (*R*)-4-decalactone[24] (1.1635 g, 98%) [eluent: petroleum ether (60-90 ^oC)/diethyl ether = 5/1 (500 mL × 2) to 3/1 (400 mL)] as an oil with pleasant flavor: 94% ee (GC conditions: CP-Chirasil-DEX CB column (25 m × 0.25 mm × 0.25 μm); carrier: N_2 at 10 psi; injector: 250 ^oC; detector (FID, H_2): 250 ^oC; oven temperature: 60 ^oC to 180 ^oC (5 ^oC/min); t_R(major) = 49.6 min, t_R(minor) = 50.1

min); $[\alpha]_D^{20}$ = +40.0 (c = 0.975, $CHCl_3$); (Lit.[24] $[\alpha]_D^{26}$ = +31 (c = 1.2, $CHCl_3$)); ^1H NMR (300 MHz, $CDCl_3$) δ 4.55-4.43 (m, 1H, =CH), 2.60-2.45 (m, 2H, CH_2), 2.40-2.25 (m, 1H, one proton from CH_2), 1.94-1.80 (m, 1H, one proton from CH_2), 1.80-1.67 (m, 1H, one proton from CH_2), 1.66-1.53 (m, 1H, one proton from CH_2), 1.52-1.19 (m, 8 H, CH_2 × 4), 0.89 (t, *J* = 6.9 Hz, 3H, CH_3); ^{13}C NMR (75 MHz, $CDCl_3$) δ 177.2, 81.0, 35.5, 31.5, 28.7, 27.9, 25.1, 22.4, 13.9; IR (neat) ν (cm^{-1}) 2956, 2932, 2858, 1778, 1461, 1422, 1379, 1351, 1292, 1218, 1181, 1127, 1022; MS (70 ev, EI) *m/z* (%) 171 (M^+ + 1, 30.46), 170 (M^+, 0.13), 85 (100).

Supplementary Figures

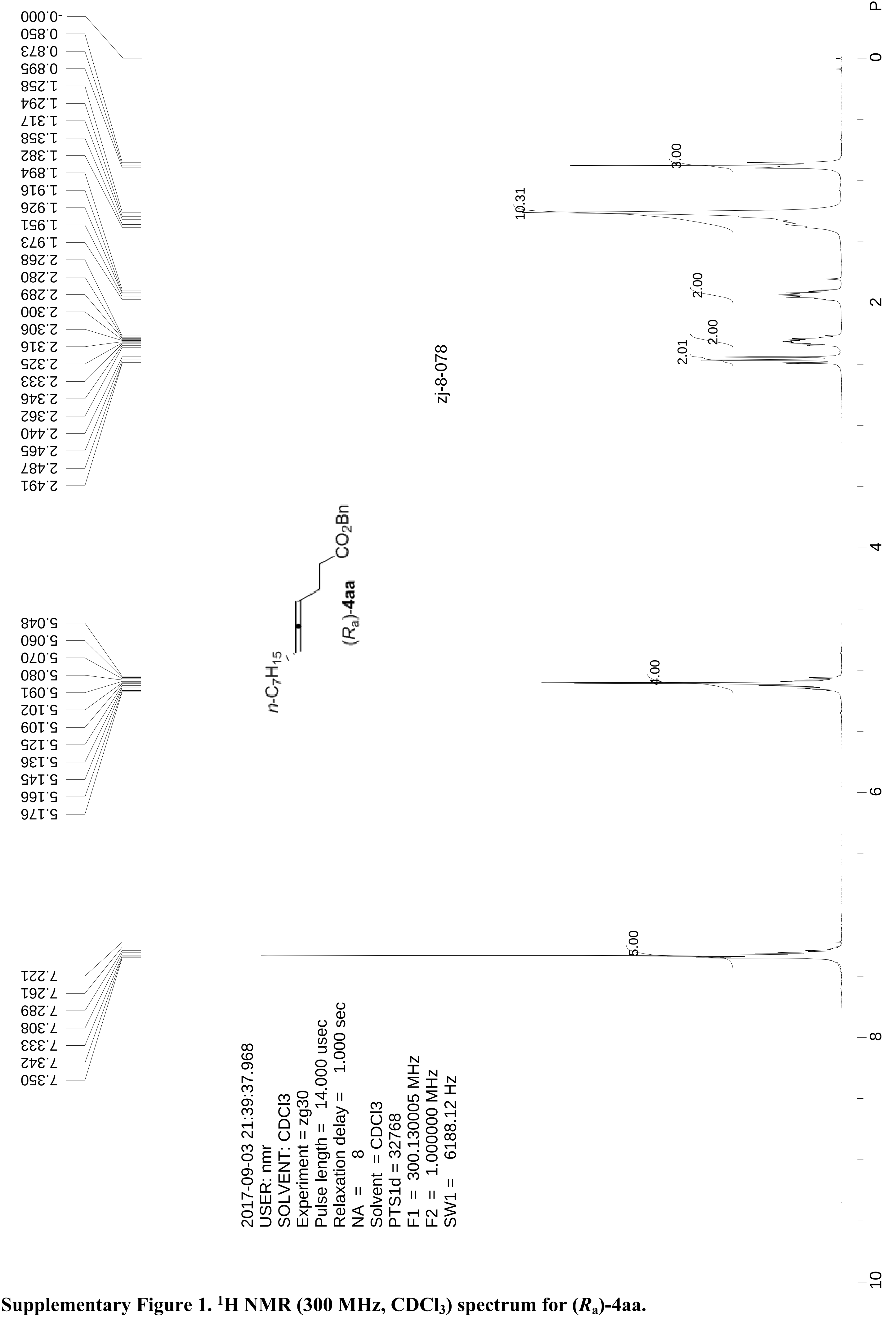

Supplementary Figure 1. **^{1}H NMR (300 MHz, $CDCl_3$) spectrum for (*R*$_a$)-4aa.**

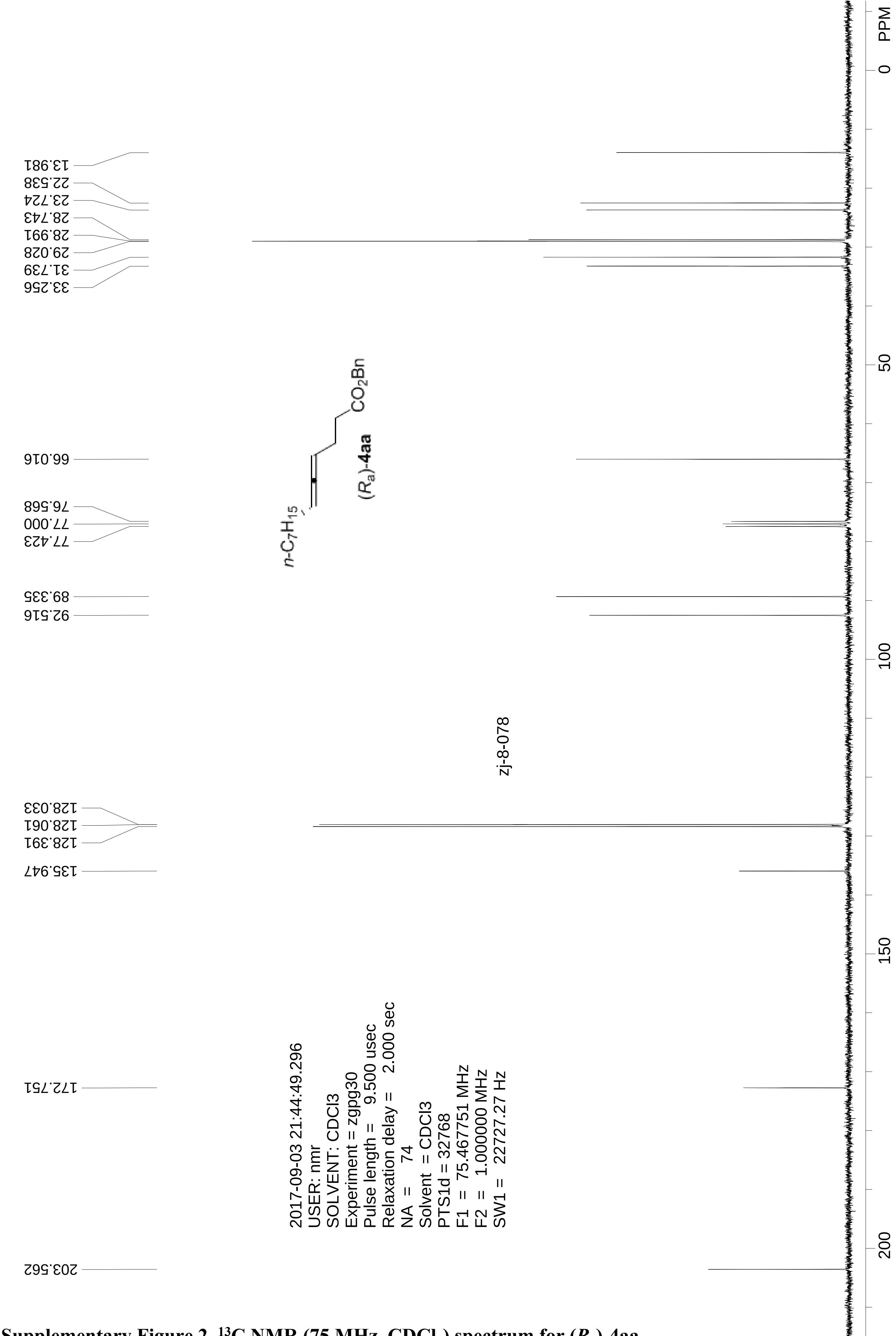

Supplementary Figure 2. ^{13}C NMR (75 MHz, $CDCl_3$) spectrum for (R_a)-4aa.

zj-8-078

实验时间：2017-09-03,21:25:58
谱图文件:D:\浙大智达\N2000\样品\S20170903212558.org
方法文件:D:\浙大智达\N2000\djx.mtd

实验者：zj
报告时间：2017-09-04,10:31:35
积分方法:面积归一法

实验内容简介：
OJ-H,214,n-hexane/i-PrOH = 200/1, 0.8

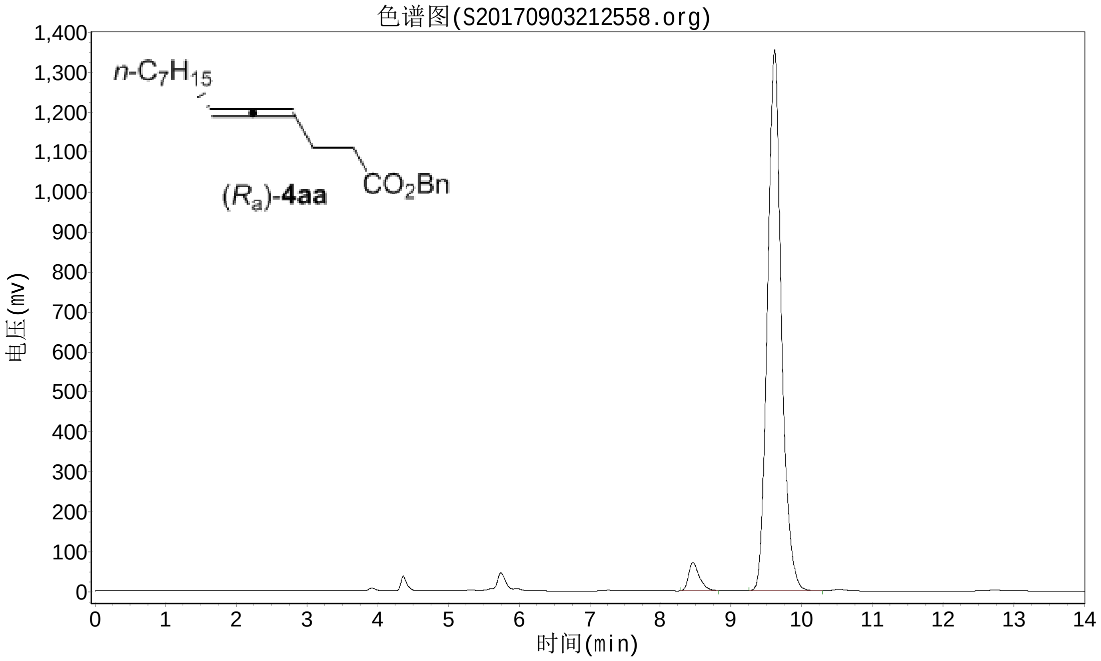

分析结果表

峰号	峰名	保留时间	峰高	峰面积	含量
1		8.462	70784.430	573324.122	2.5642
2		9.617	1354498.625	21785467.000	97.4358
总计			1425283.055	19287909.438	100.0000

Supplementary Figure 3. HPLC spectrum for (R_a)-4aa.

zj-8-079

实验时间：2017-09-03,21:05:00
谱图文件:D:\浙大智达\N2000\样品\S20170903210500.org
方法文件:D:\浙大智达\N2000\djx.mtd

实验者：zj
报告时间：2017-09-04,10:21:11
积分方法:面积归一法

实验内容简介：
OJ-H,214,n-hexane/i-PrOH = 200/1, 0.8

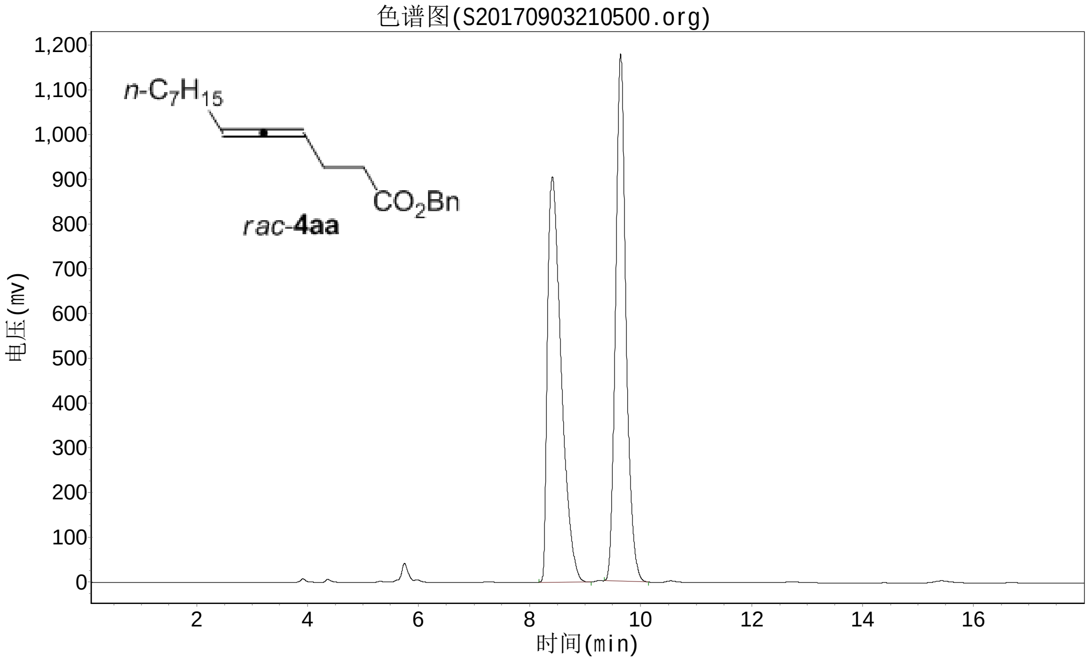

分析结果表

峰号	峰名	保留时间	峰高	峰面积	含量
1		8.408	906405.375	15571489.000	50.2998
2		9.640	1178140.750	15385857.000	49.7002
总计			2084546.125	30957346.000	100.0000

Supplementary Figure 4. HPLC spectrum for *rac*-4aa.

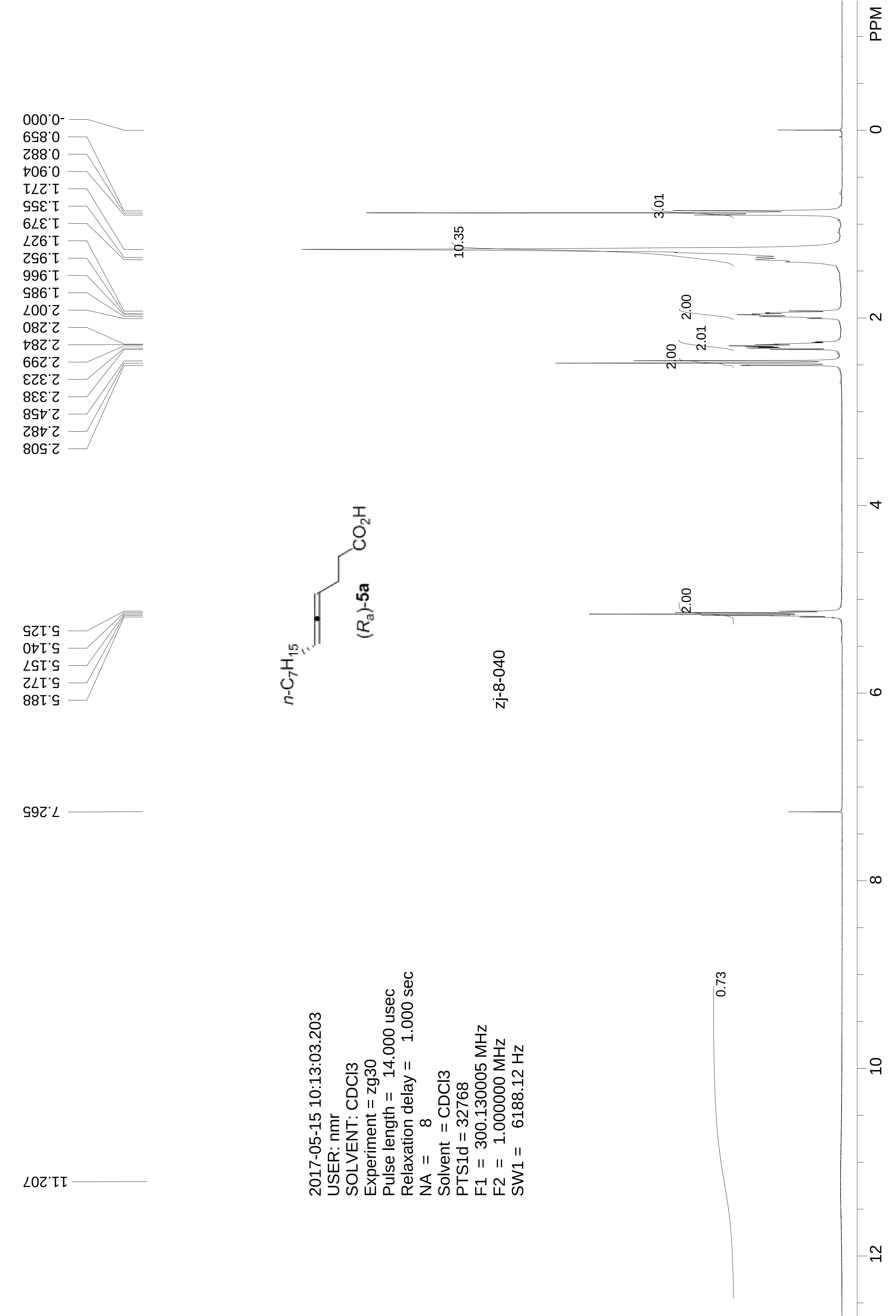

Supplementary Figure 5. **^{1}H NMR (300 MHz, $CDCl_3$) spectrum for (R_a)-5a.**

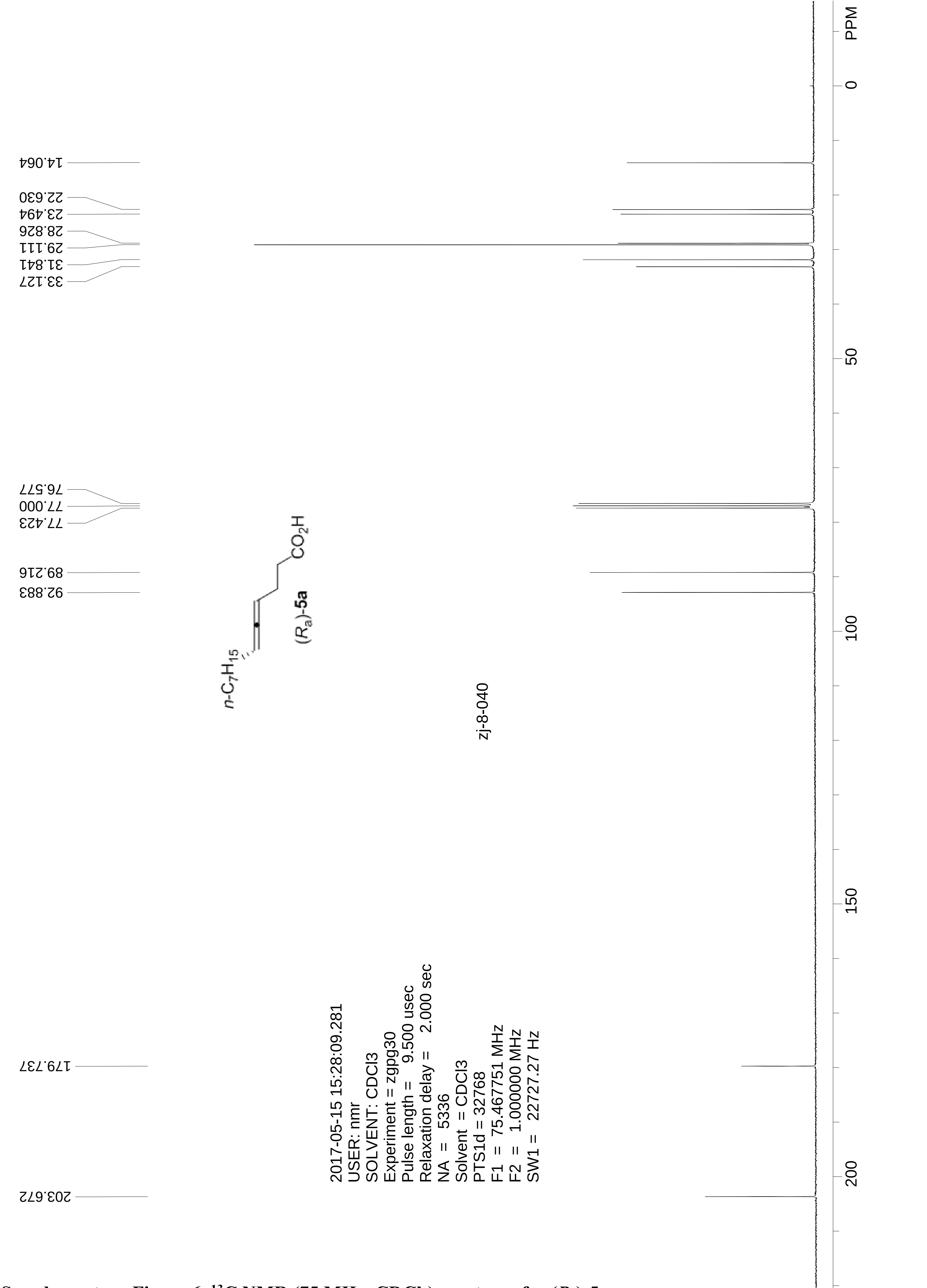

Supplementary Figure 6. ^{13}C NMR (75 MHz, $CDCl_3$) spectrum for (R_a)-5a.

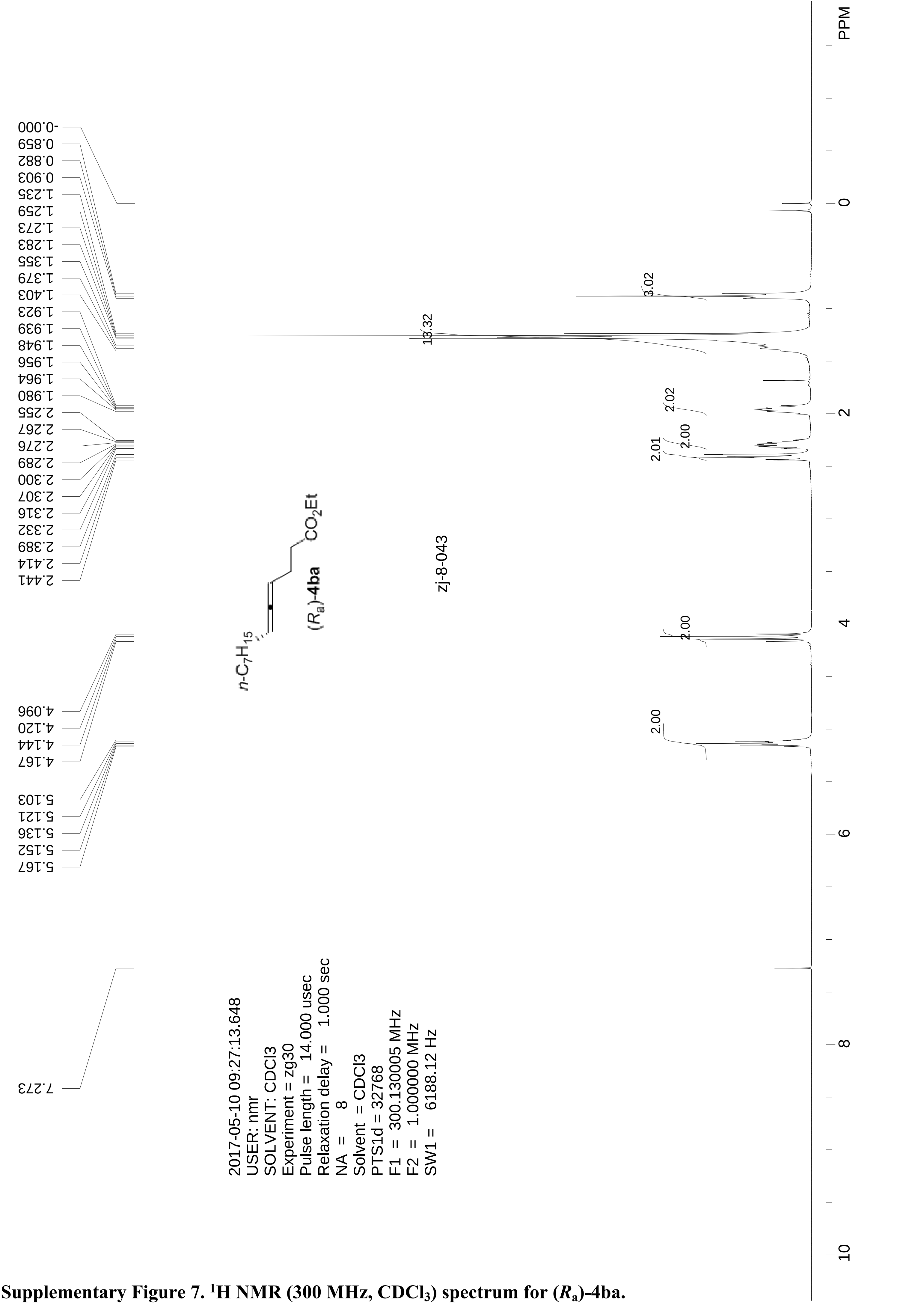

Supplementary Figure 7. ^{1}H NMR (300 MHz, $CDCl_3$) spectrum for (R_a)-4ba.

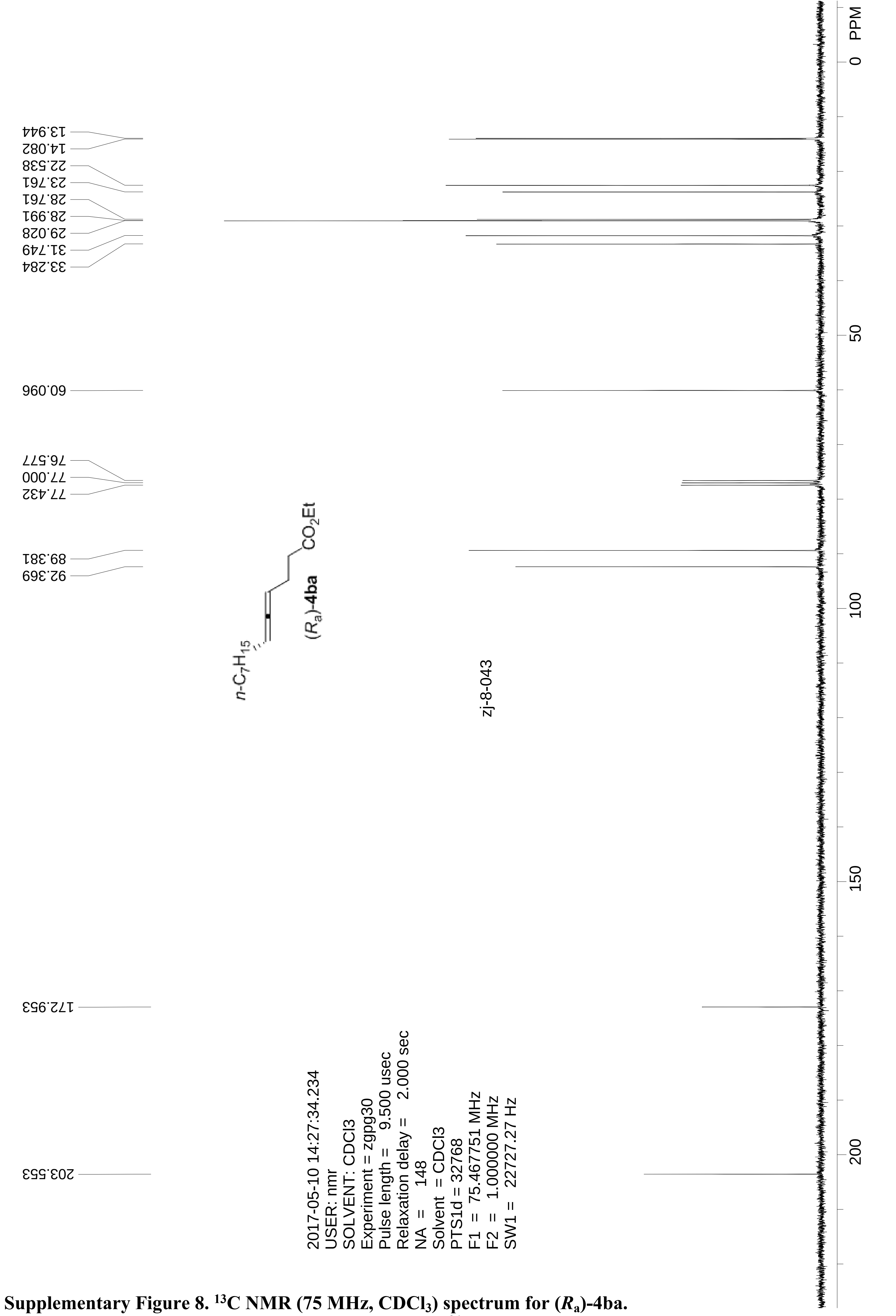

Supplementary Figure 8. ^{13}C NMR (75 MHz, $CDCl_3$) spectrum for (R_a)-4ba.

zj-8-043-od-h-100-0-0.5-214

实验时间：2017-5-25,16:18:55　　　　报告时间：2017-5-25,17:01:38
谱图文件:D:\zhuguangjiong\zj\20170525\zj-8-043-od-h-100-0-0.5-214..org

实验内容简介：

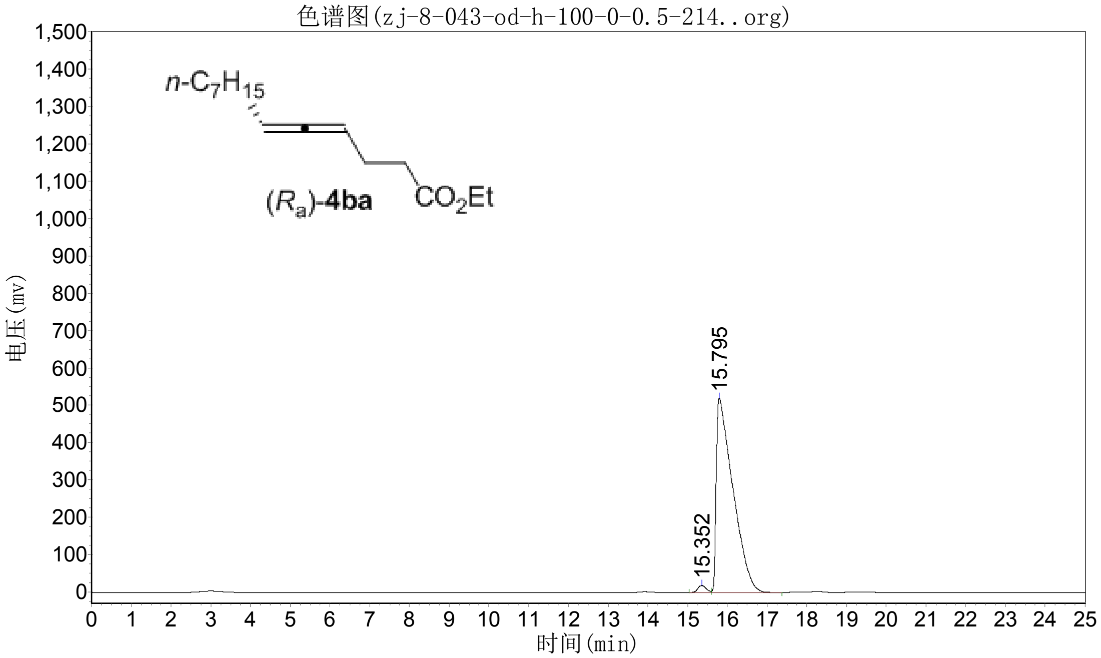

分析结果表

峰号	峰名	保留时间	峰高	峰面积	含量
1		15.352	18955.471	279392.031	1.8106
2		15.795	520920.969	15151092.000	98.1894
总计			539876.439	15430484.031	100.0000

Supplementary Figure 9. HPLC spectrum for (*R*$_a$)-4ba.

zj-8-020-od-h-100-0-0.5-214

实验时间：2017-5-25,15:06:21　　报告时间：2017-5-25,16:33:57
谱图文件:D:\zhuguangjiong\zj\20170525\zj-8-020-od-h-100-0-0.5-214.org

实验内容简介：

色谱图(zj-8-020-od-h-100-0-0.5-214.org)

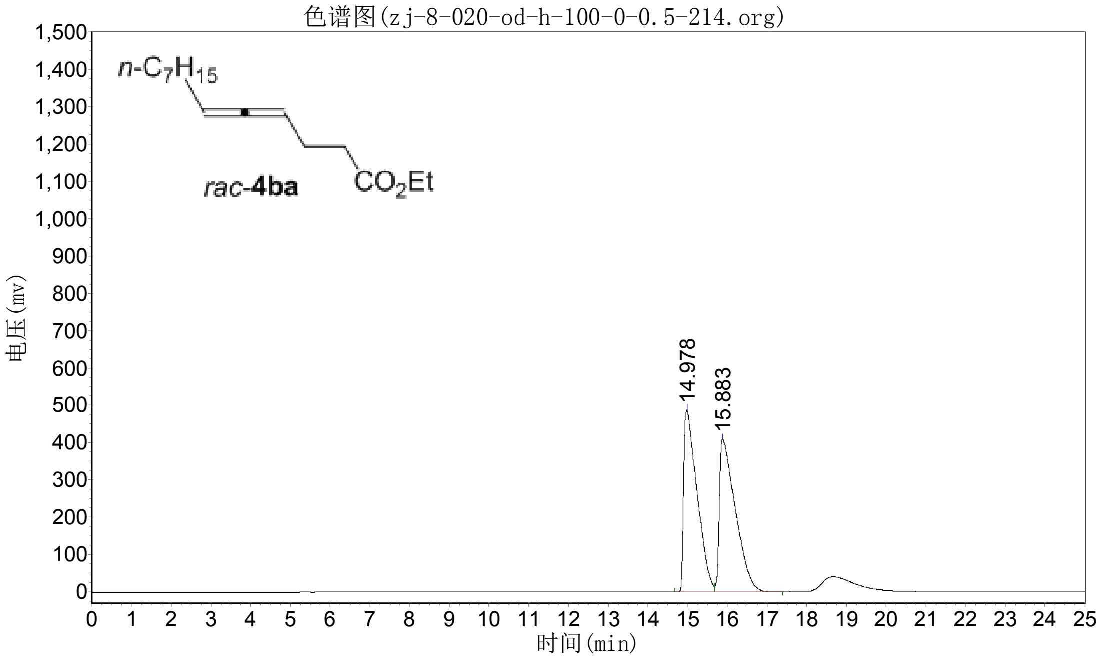

分析结果表

峰号	峰名	保留时间	峰高	峰面积	含量
1		14.978	487824.688	11432328.000	49.6573
2		15.883	409940.844	11590101.000	50.3427
总计			897765.531	23022429.000	100.0000

Supplementary Figure 10. HPLC spectrum for *rac*-4ba.

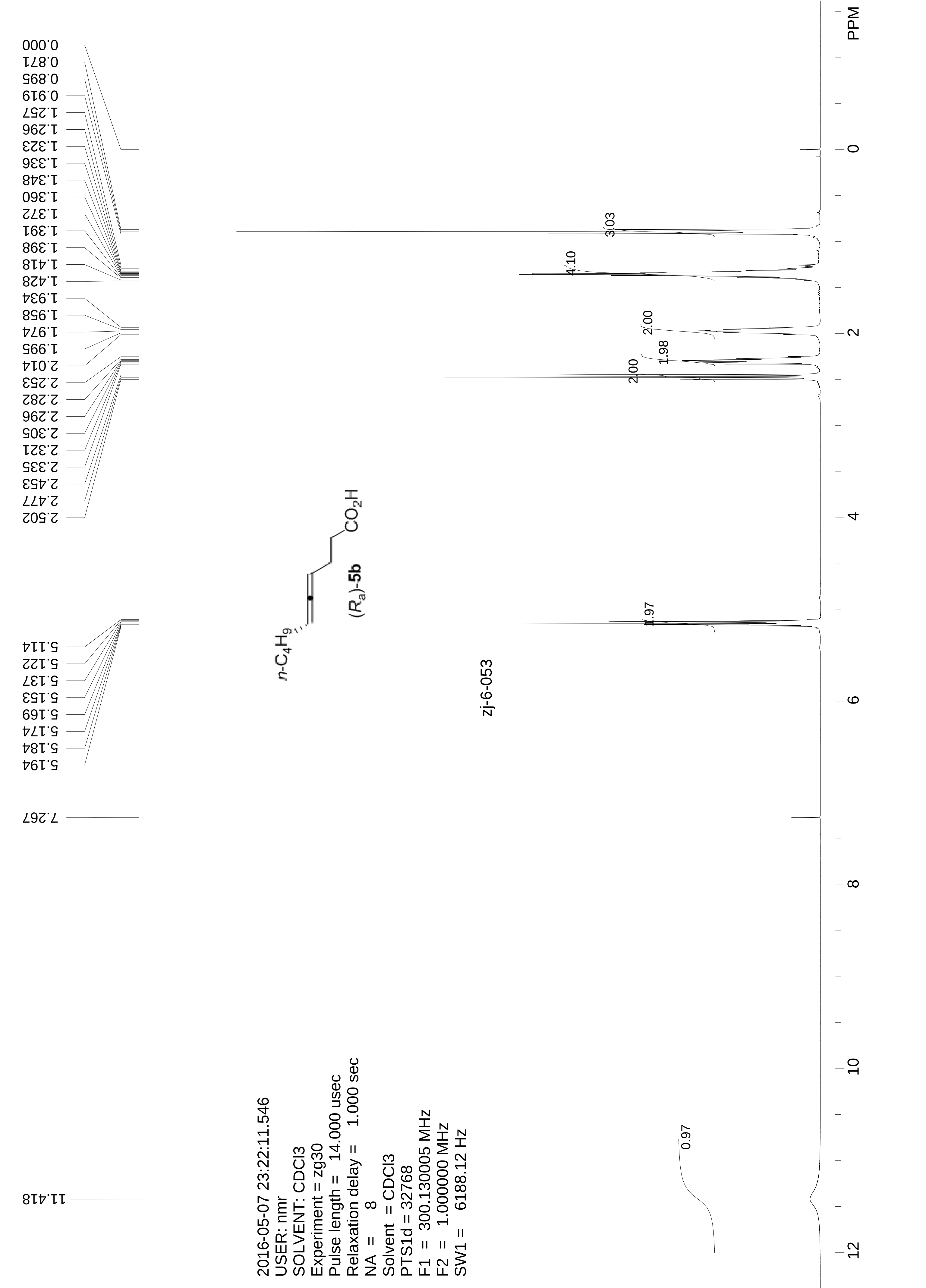

Supplementary Figure 11. ^{1}H NMR (300 MHz, $CDCl_3$) spectrum for (R_a)-5b.

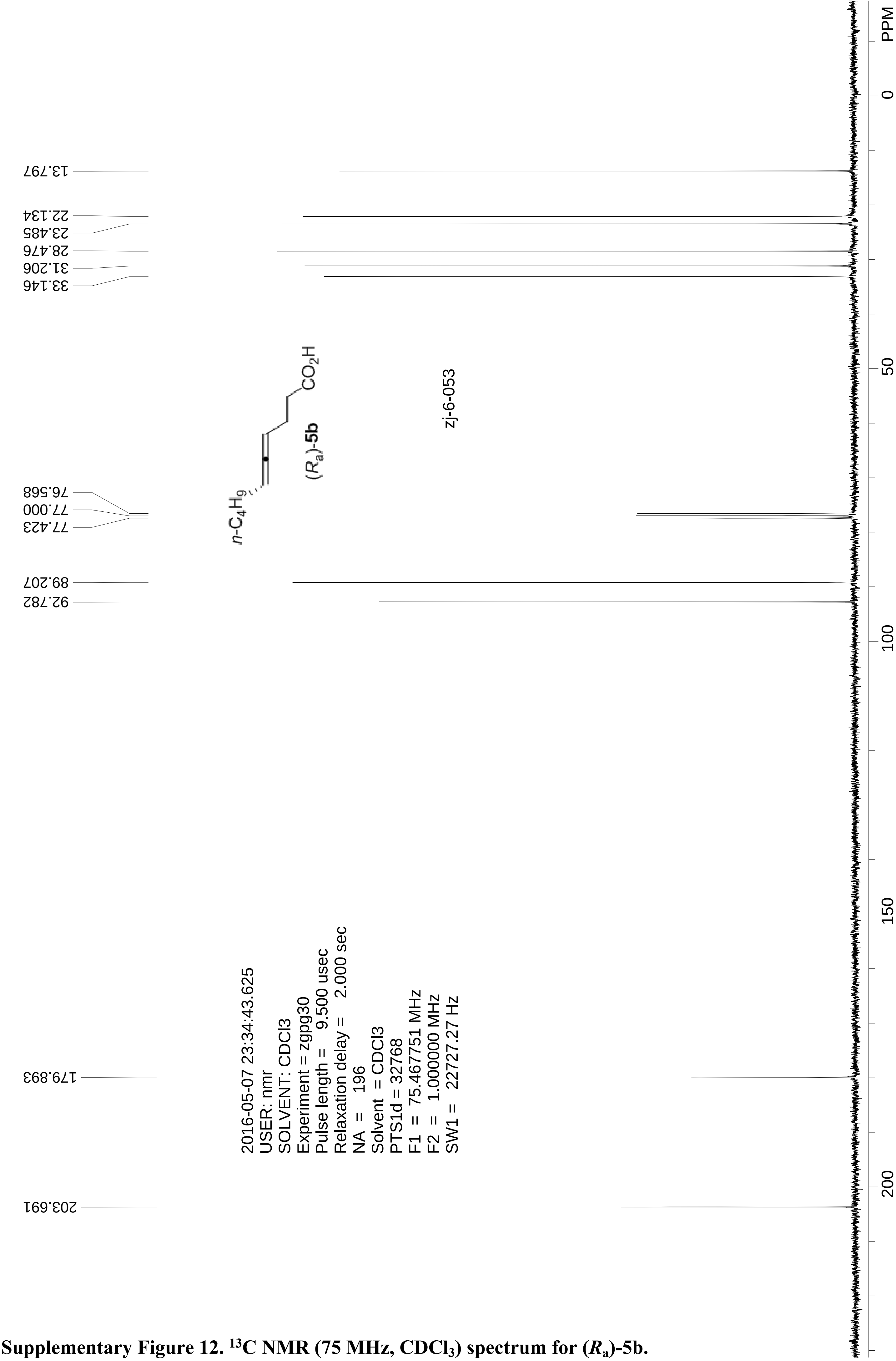

Supplementary Figure 12. ^{13}C NMR (75 MHz, $CDCl_3$) spectrum for (R_a)-5b.

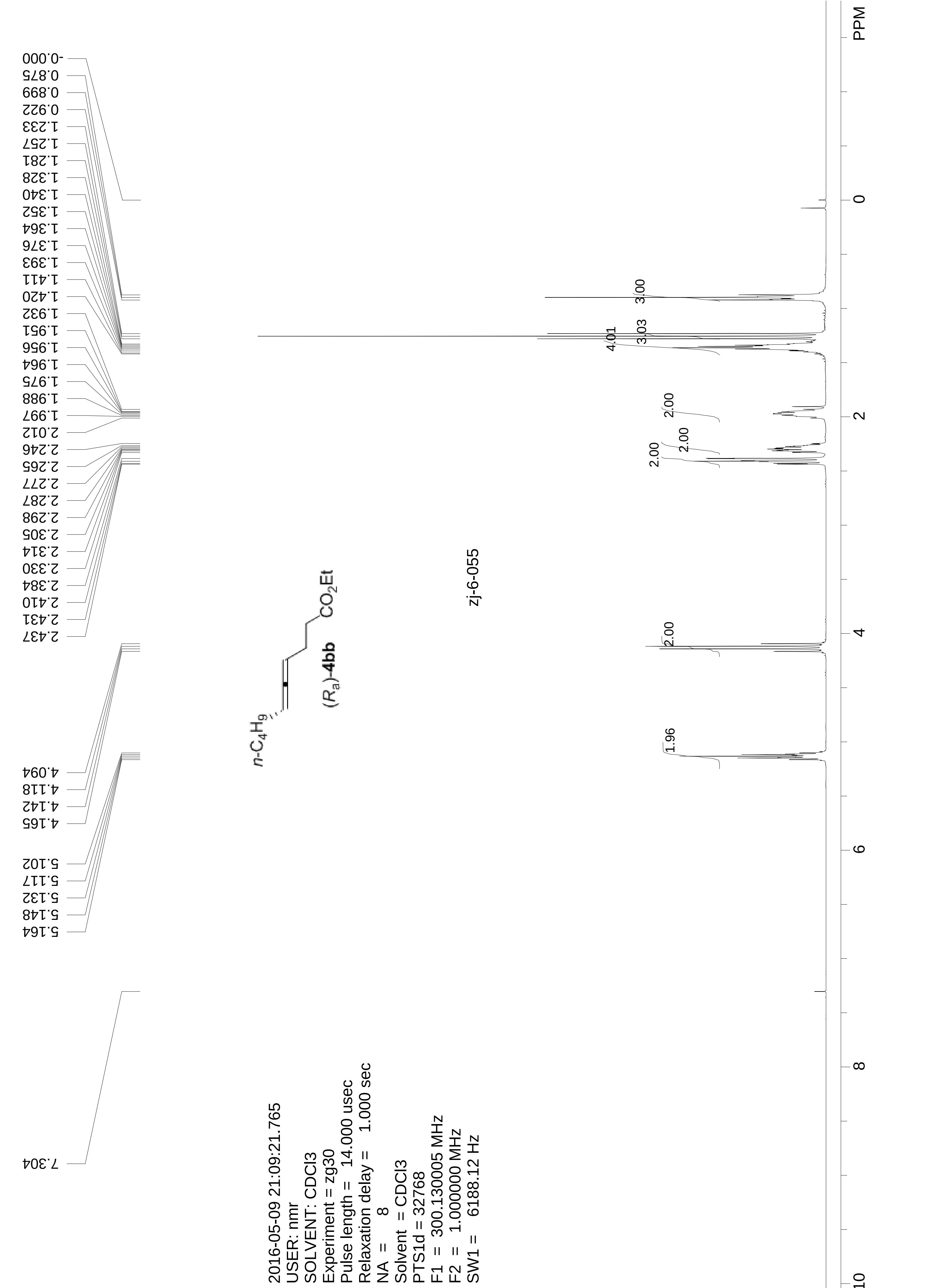

Supplementary Figure 13. ^{1}**H NMR (300 MHz, CDCl$_3$) spectrum for (*R*$_a$)-4bb.**

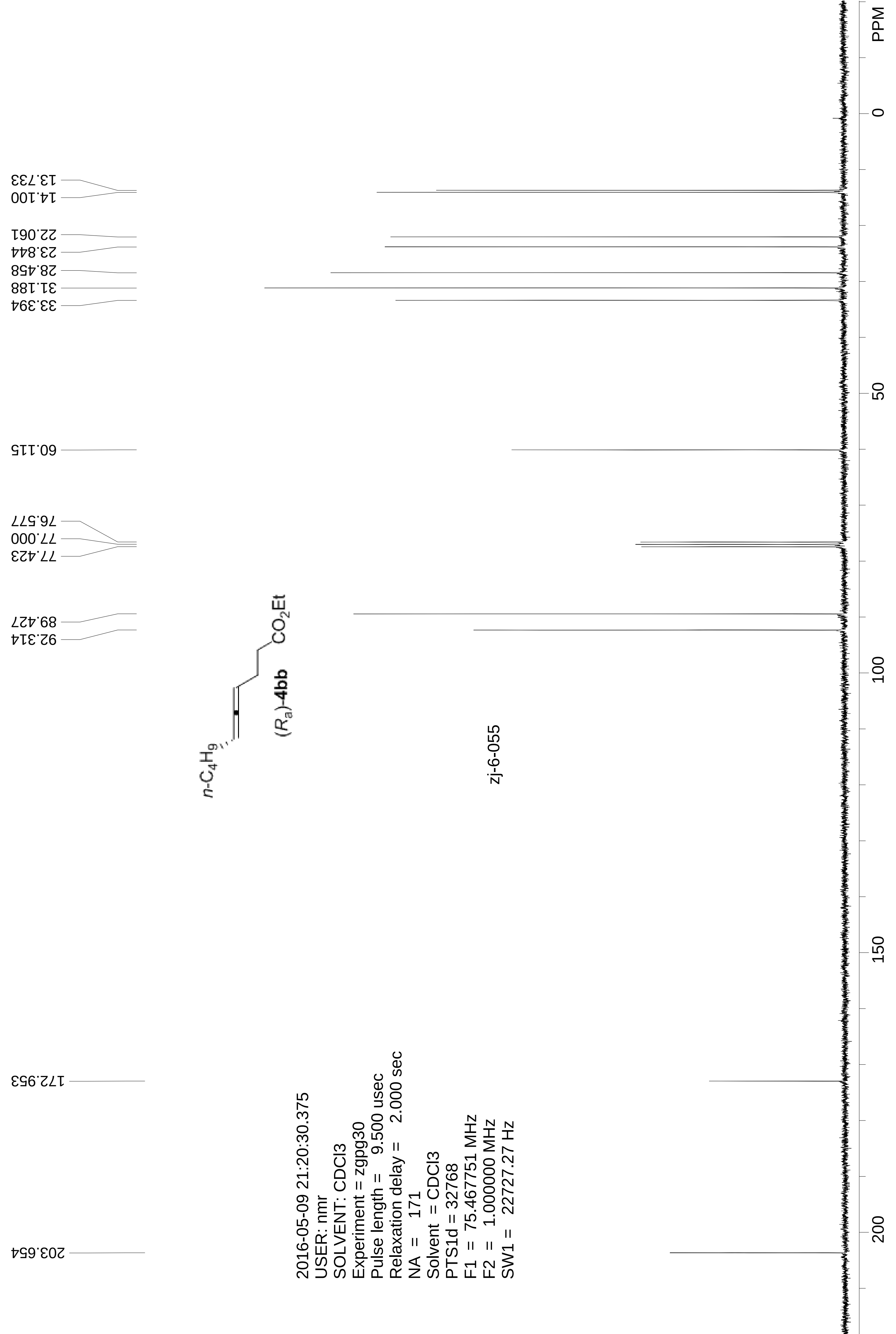

Supplementary Figure 14. ^{13}**C NMR (75 MHz, CDCl$_3$) spectrum for (*R*$_a$)-4bb.**

zj-6-055-pc-2-100-0-0.7-214

实验时间：2016-06-23,13:21:42　　　　报告时间：2016-06-23,14:23:27
谱图文件:D:\zhuguangjiong\zj\20160617\zj-6-055-pc-2-100-0-0.7-214.org

实验内容简介：

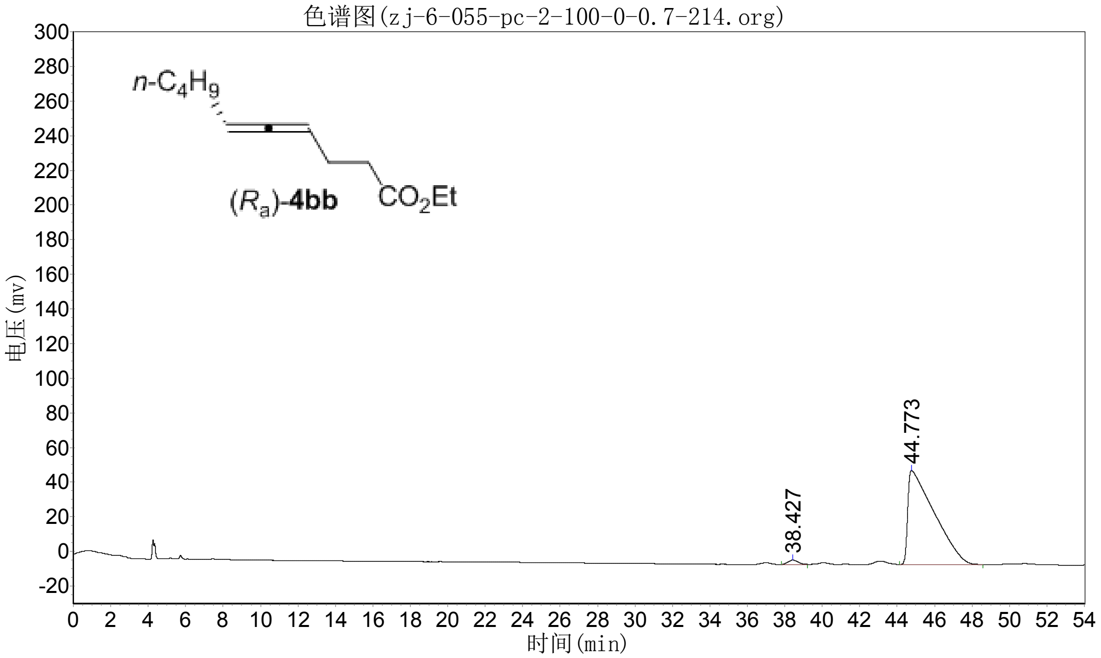

分析结果表

峰号	峰名	保留时间	峰高	峰面积	含量
1		38.427	2442.978	90536.805	1.6766
2		44.773	54326.734	5309446.500	98.3234
总计			56769.713	5399983.305	100.0000

Supplementary Figure 15. HPLC spectrum for (R_a)-4bb.

zj-6-054-pc-2-100-0-0.7-214

实验时间：2016-06-23,12:22:21 报告时间：2016-06-23,14:18:40
谱图文件:D:\zhuguangjiong\zj\20160617\zj-6-054-pc-2-100-0-0.7-214.org

实验内容简介：

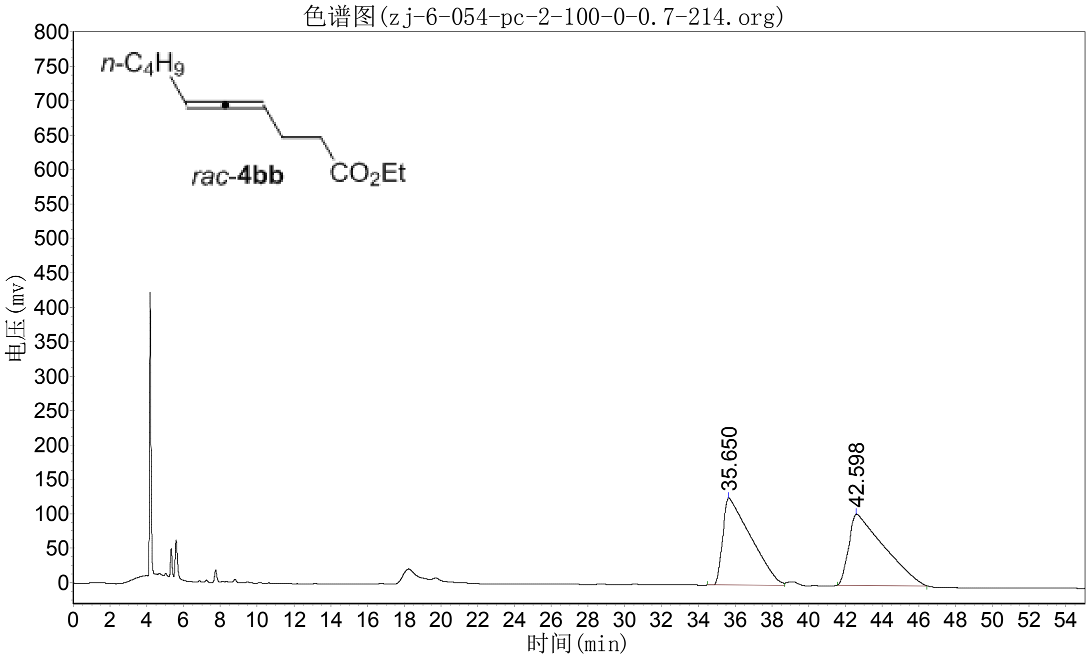

分析结果表

峰号	峰名	保留时间	峰高	峰面积	含量
1		35.650	127000.523	13396767.000	49.5270
2		42.598	103346.539	13652672.000	50.4730
总计			230347.063	27049439.000	100.0000

Supplementary Figure 16. HPLC spectrum for *rac*-4bb.

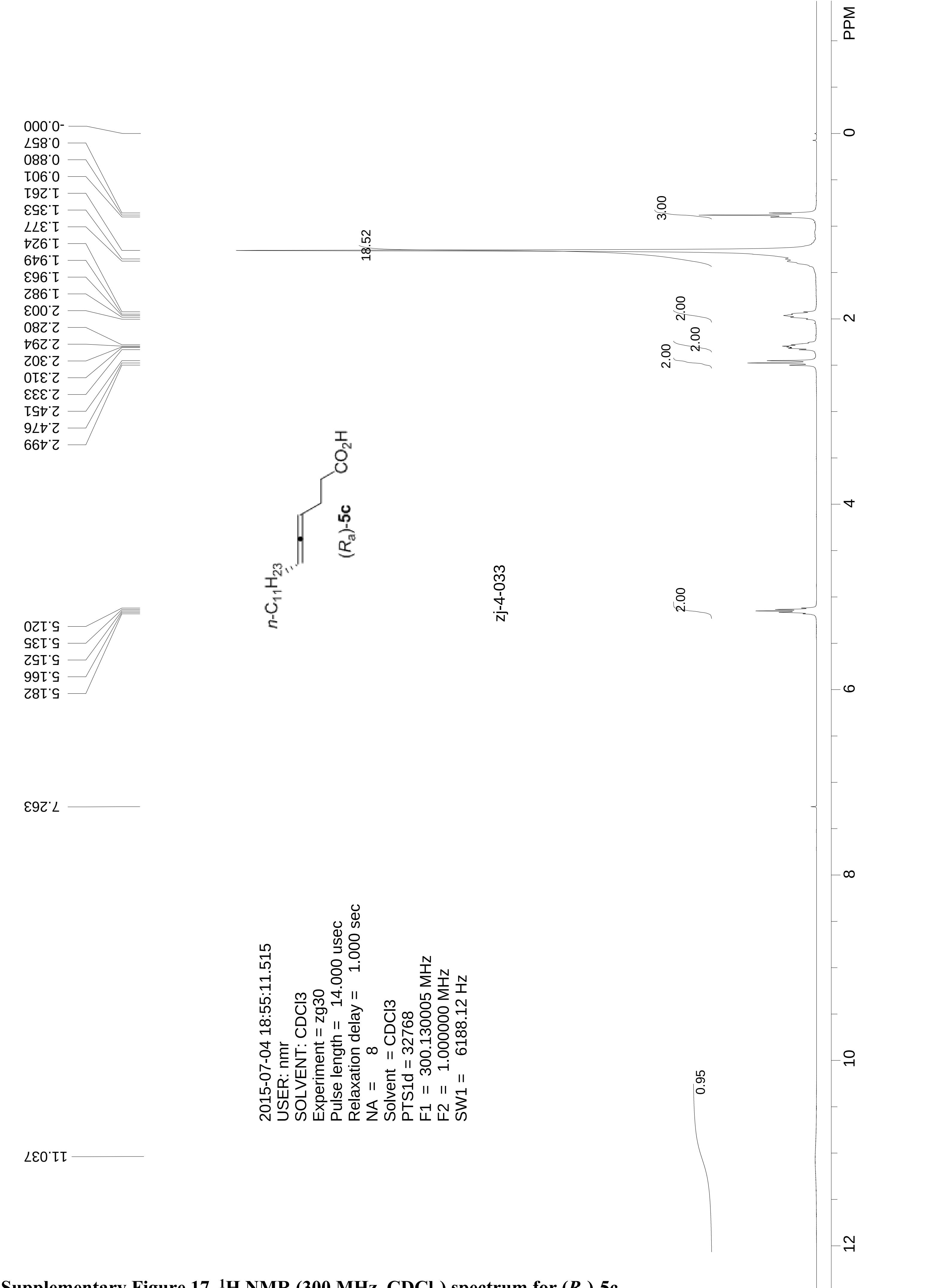

Supplementary Figure 17. ^{1}**H NMR (300 MHz, CDCl$_3$) spectrum for (*R*$_a$)-5c.**

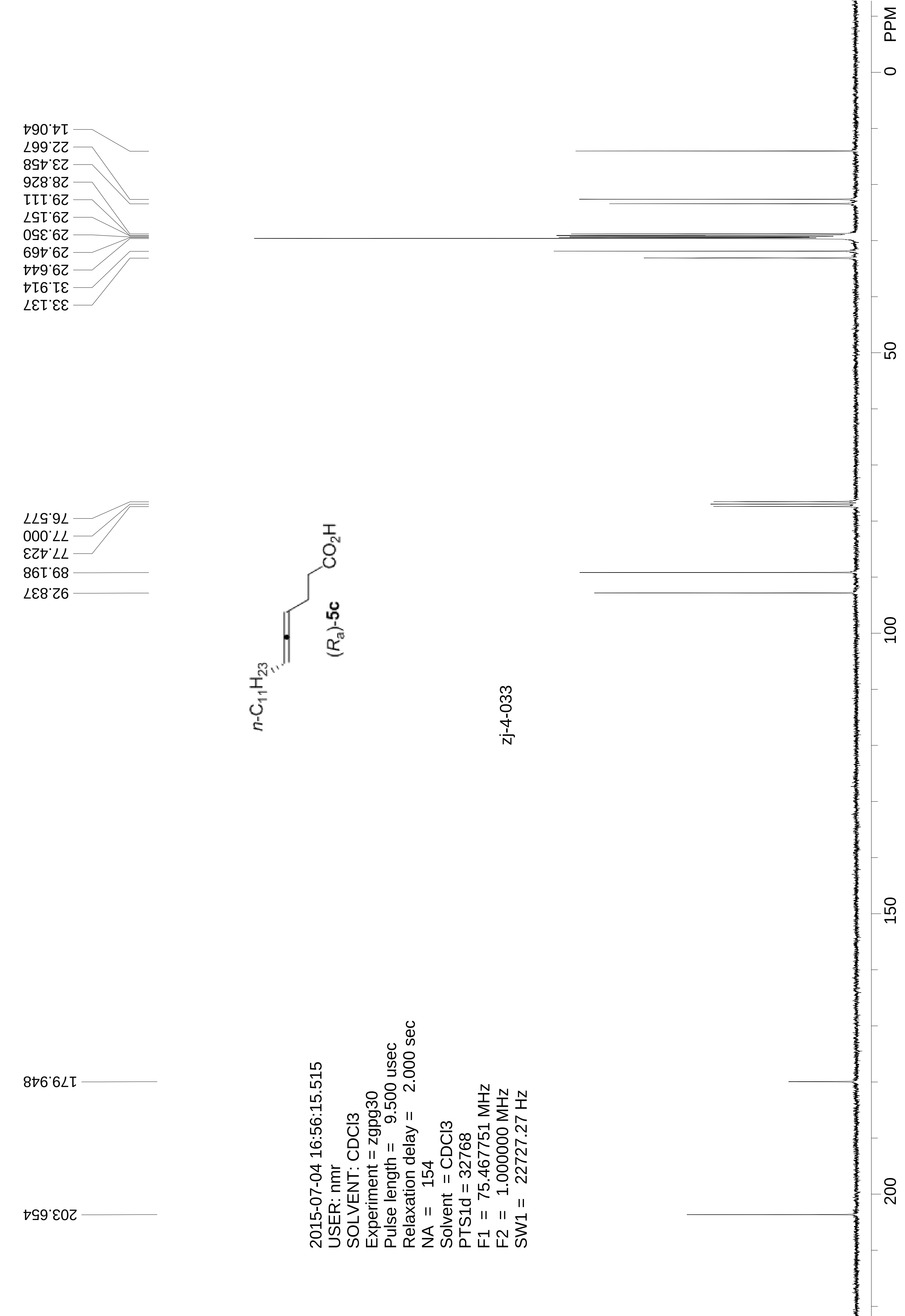

Supplementary Figure 18. ^{13}C NMR (75 MHz, $CDCl_3$) spectrum for (R_a)-5c.

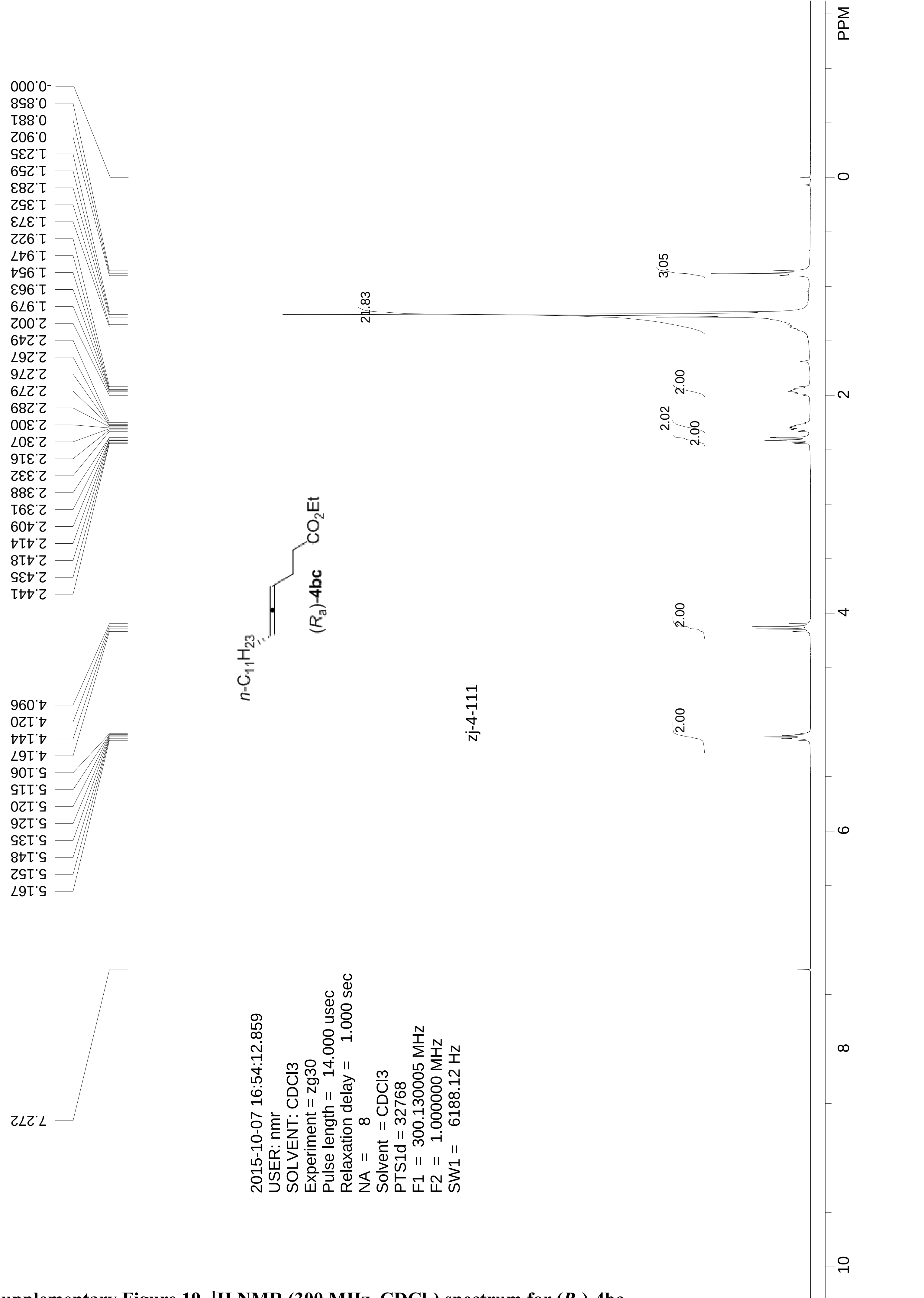

Supplementary Figure 19. ^{1}H NMR (300 MHz, CDCl$_3$) spectrum for (R_a)-4bc.

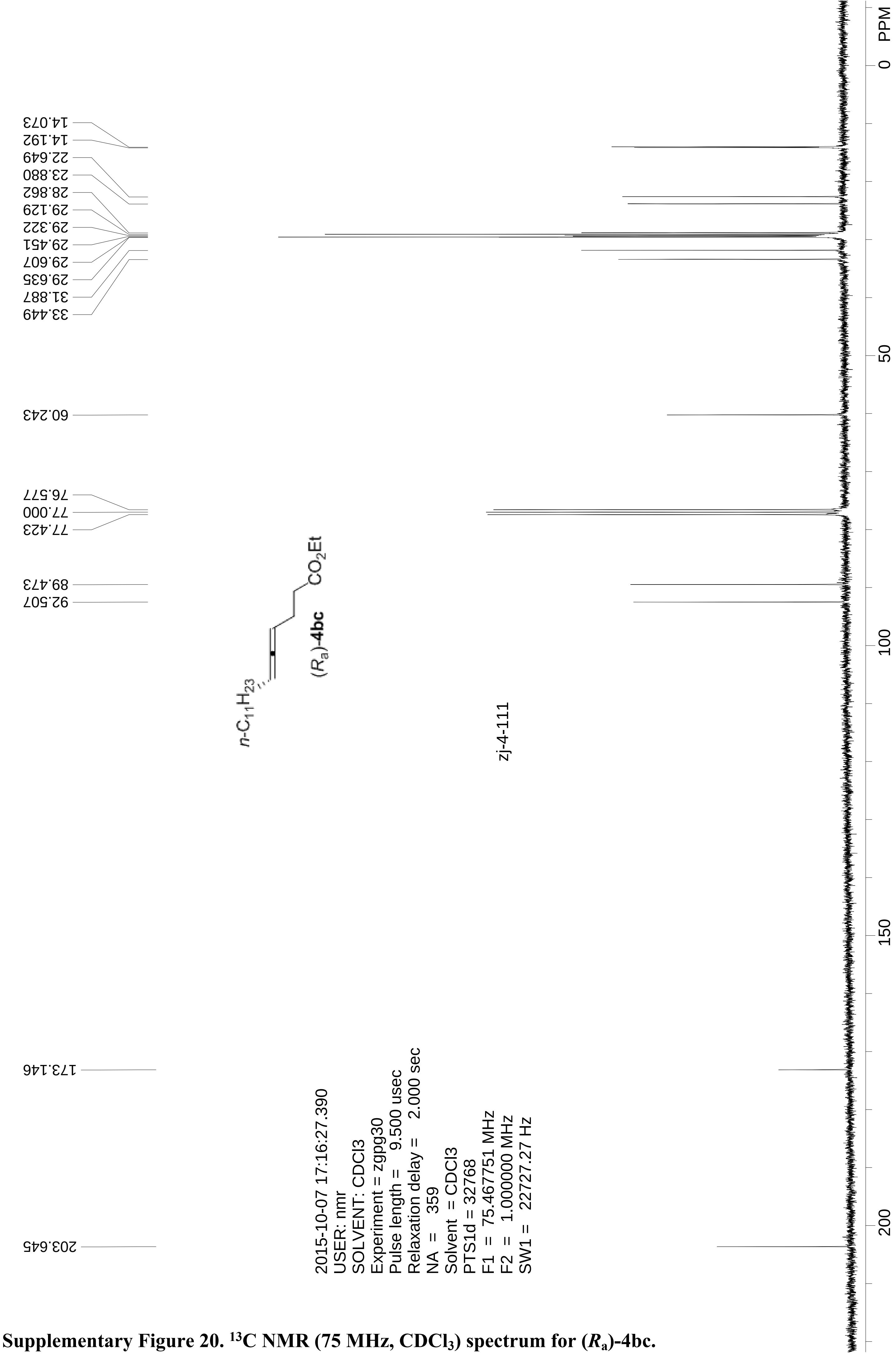

Supplementary Figure 20. ^{13}**C NMR (75 MHz, CDCl$_3$) spectrum for (*R*$_a$)-4bc.**

zj-4-111-od-h-100-0-0.7-214

实验时间：2015-10-13,9:01:11　　　　报告时间：2015-10-13,10:14:25
谱图文件:D:\zhuguangjiong\zj\20151010\zj-4-111-od-h-100-0-0.7-214.org

实验内容简介：

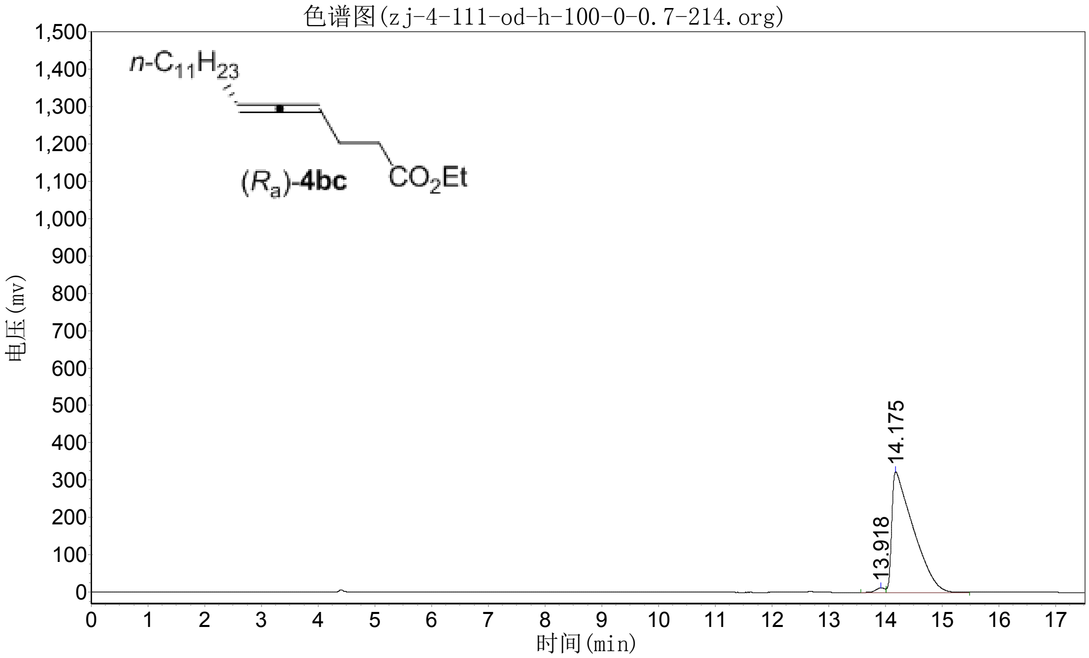

分析结果表

峰号	峰名	保留时间	峰高	峰面积	含量
1		13.918	12605.106	135971.844	1.5729
2		14.175	322828.813	8508424.000	98.4271
总计			335433.919	8644395.844	100.0000

Supplementary Figure 21. HPLC spectrum for (R_a)-4bc.

zj-4-110-od-h-100-0-0.7-214

实验时间：2015-10-13,9:39:25　　　　报告时间：2015-10-13,10:12:11
谱图文件:D:\zhuguangjiong\zj\20151010\zj-4-110-od-h-100-0-0.7-214...org

实验内容简介：

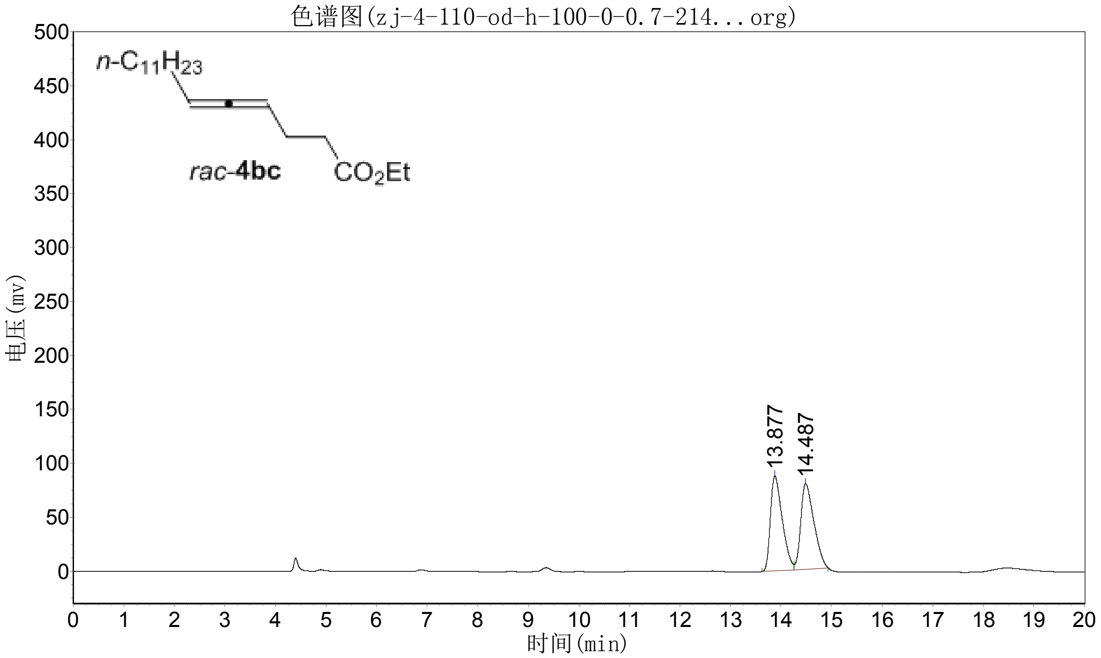

分析结果表

峰号	峰名	保留时间	峰高	峰面积	含量
1		13.877	87863.875	1429751.875	49.7726
2		14.487	79448.938	1442813.875	50.2274
总计			167312.813	2872565.750	100.0000

Supplementary Figure 22. HPLC spectrum for *rac*-4bc.

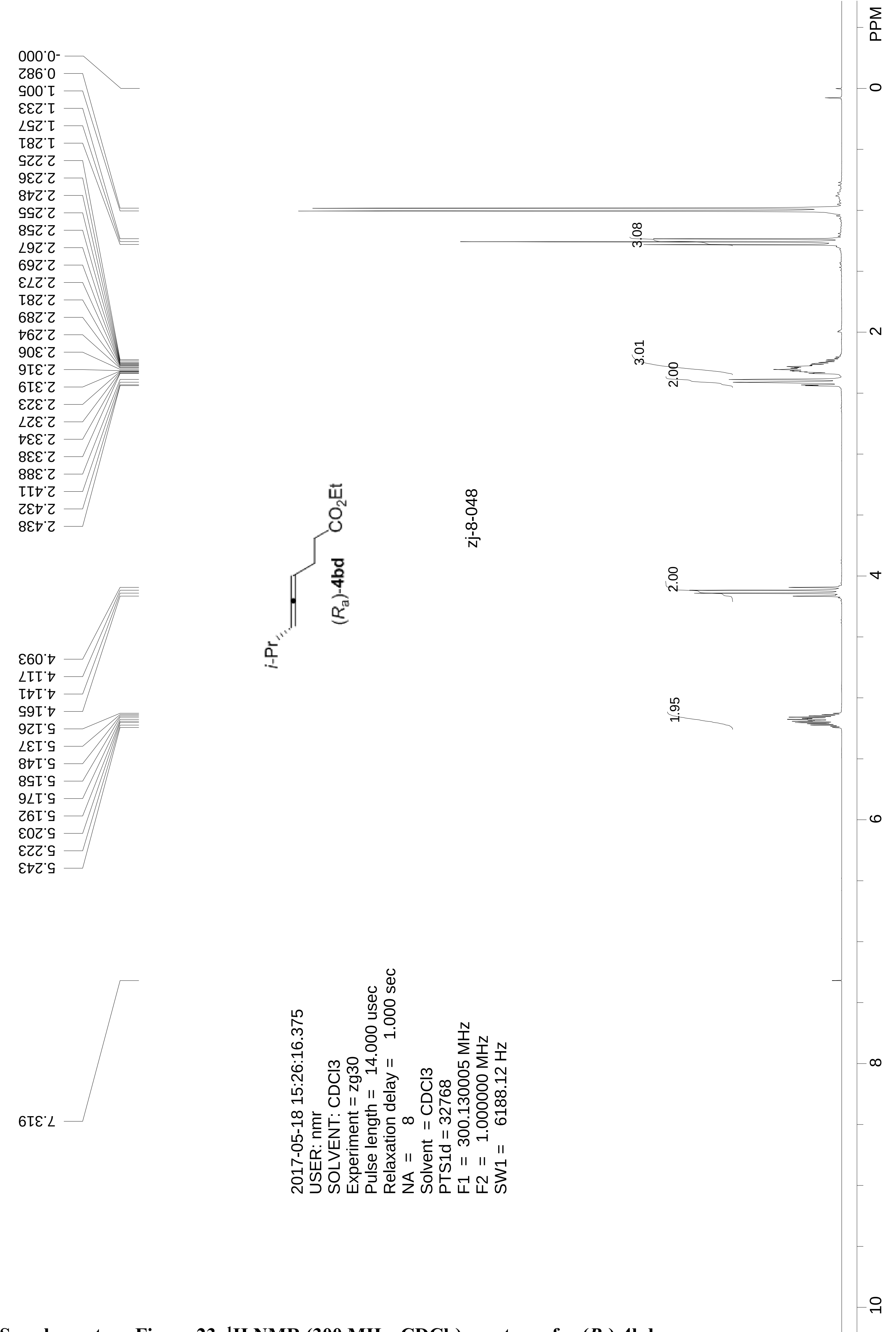

Supplementary Figure 23. ^{1}H NMR (300 MHz, $CDCl_3$) spectrum for (R_a)-4bd.

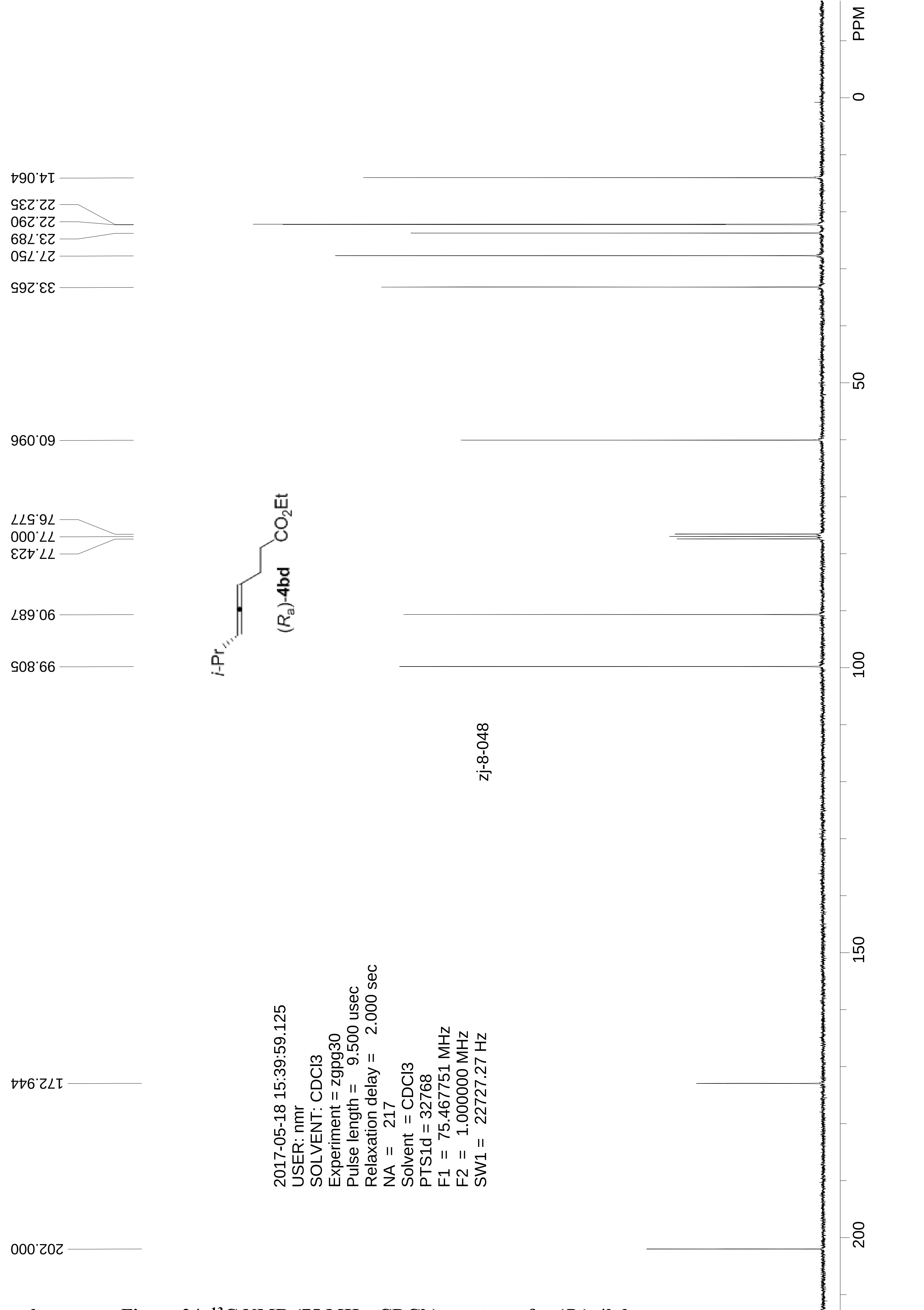

Supplementary Figure 24. ^{13}C NMR (75 MHz, $CDCl_3$) spectrum for (R_a)-4bd.

中国科学院上海有机化学研究所

Project Name: defaults for copy

Reported by User: Breeze user (Breeze)

Breeze 2 HPLC System

SAMPLE INFORMATION

Sample Name:	zj-8-048-od-h-100-0-1-214	Acquired By:	Breeze
Sample Type:	未知	Date Acquired:	2017/5/22 11:13:54 CST
Vial:	999	Acq. Method:	zgj100
Injection #:	89	Date Processed:	2017/5/22 17:00:05 CST
Injection Volume:	10.00 ul	Channel Name:	W2489 ChA
Run Time:	20.00 Minutes	Channel Desc.:	W2489 ChA 214nm
Column Type:		Sample Set Name:	

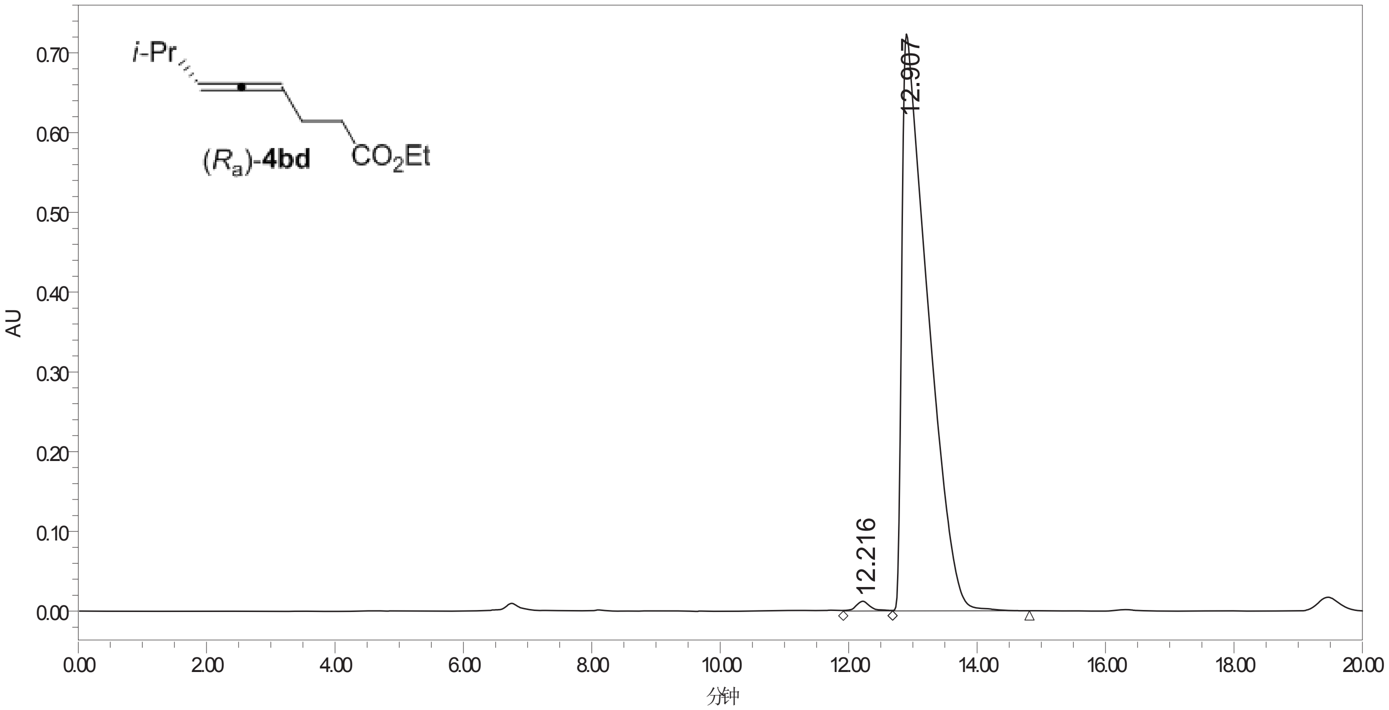

	RT (min)	Area (礦sec)	% Area	Height (礦)	% Height
1	12.216	193423	0.94	12225	1.66
2	12.907	20285610	99.06	725351	98.34

Report Method: Individual Report ASC

Page: 1 (共计 1)

Printed: 2017/5/22 17:00:13 PRC

Supplementary Figure 25. HPLC spectrum for (*R*$_a$)-4bd.

中国科学院上海有机化学研究所

Project Name: defaults for copy

Reported by User: Breeze user (Breeze)

SAMPLE INFORMATION

Sample Name:	zj-8-047-od-h-100-0-1-214	Acquired By:	Breeze
Sample Type:	未知	Date Acquired:	2017/5/22 10:23:24 CST
Vial:	999	Acq. Method:	zgj100
Injection #:	87	Date Processed:	2017/5/22 16:59:49 CST
Injection Volume:	10.00 ul	Channel Name:	W2489 ChA
Run Time:	65.00 Minutes	Channel Desc.:	W2489 ChA 214nm
Column Type:		Sample Set Name:	

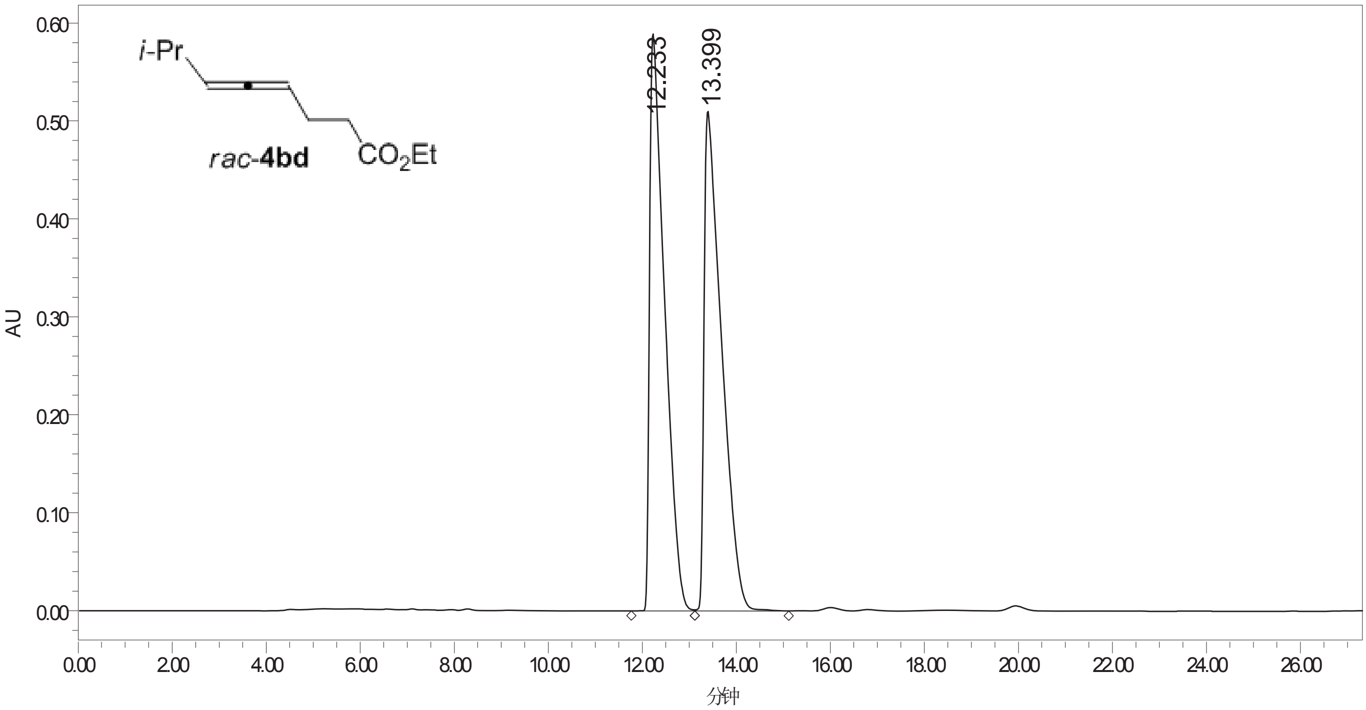

	RT (min)	Area (礦sec)	% Area	Height (礦)	% Height
1	12.233	13051544	49.68	589317	53.59
2	13.399	13222295	50.32	510277	46.41

Report Method: Individual Report ASC

Printed: 2017/5/22 17:00:45 PRC

Page: 1 (共计 1)

Supplementary Figure 26. HPLC spectrum for *rac*-4bd.

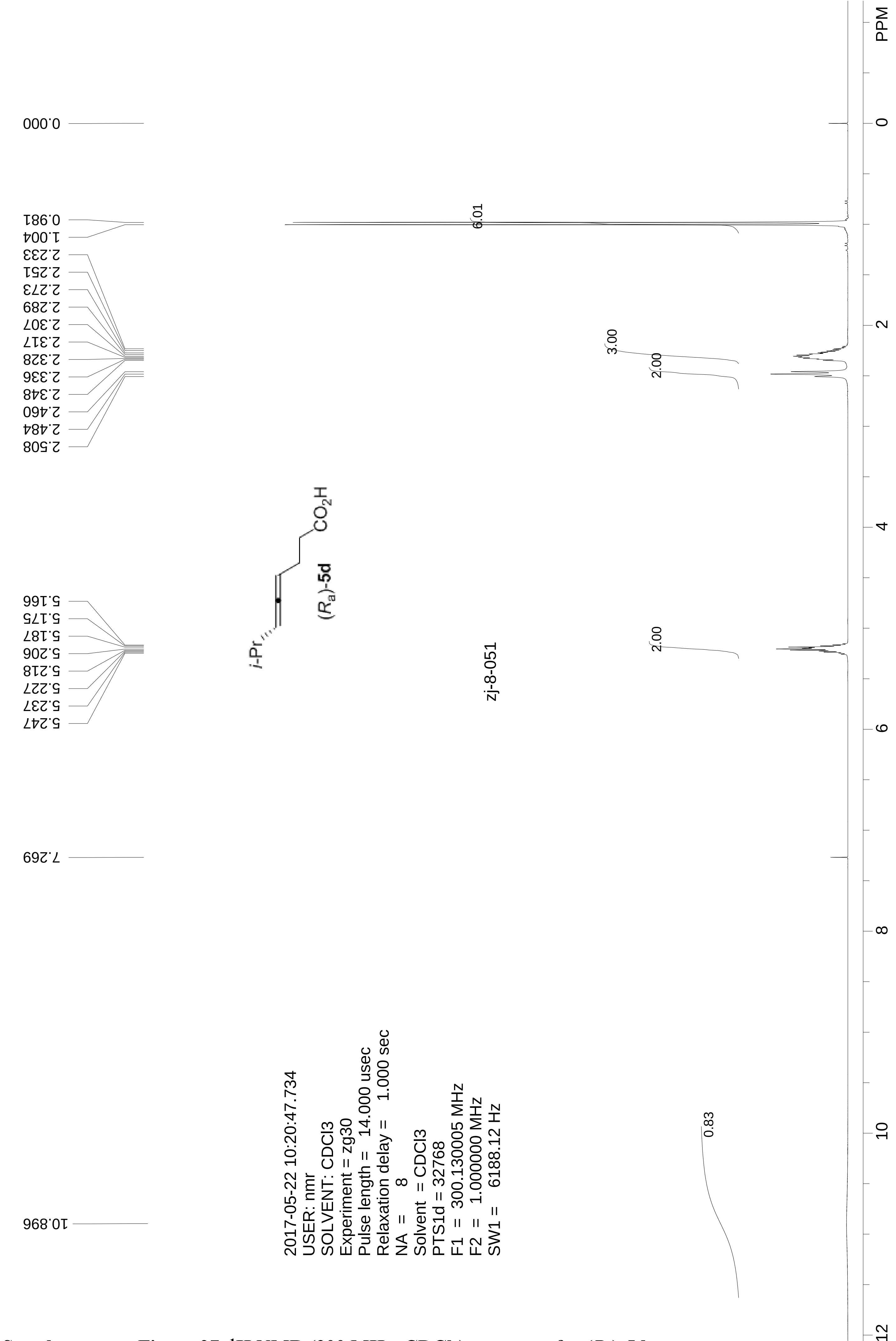

Supplementary Figure 27. ^{1}H NMR (300 MHz, $CDCl_3$) spectrum for (R_a)-5d.

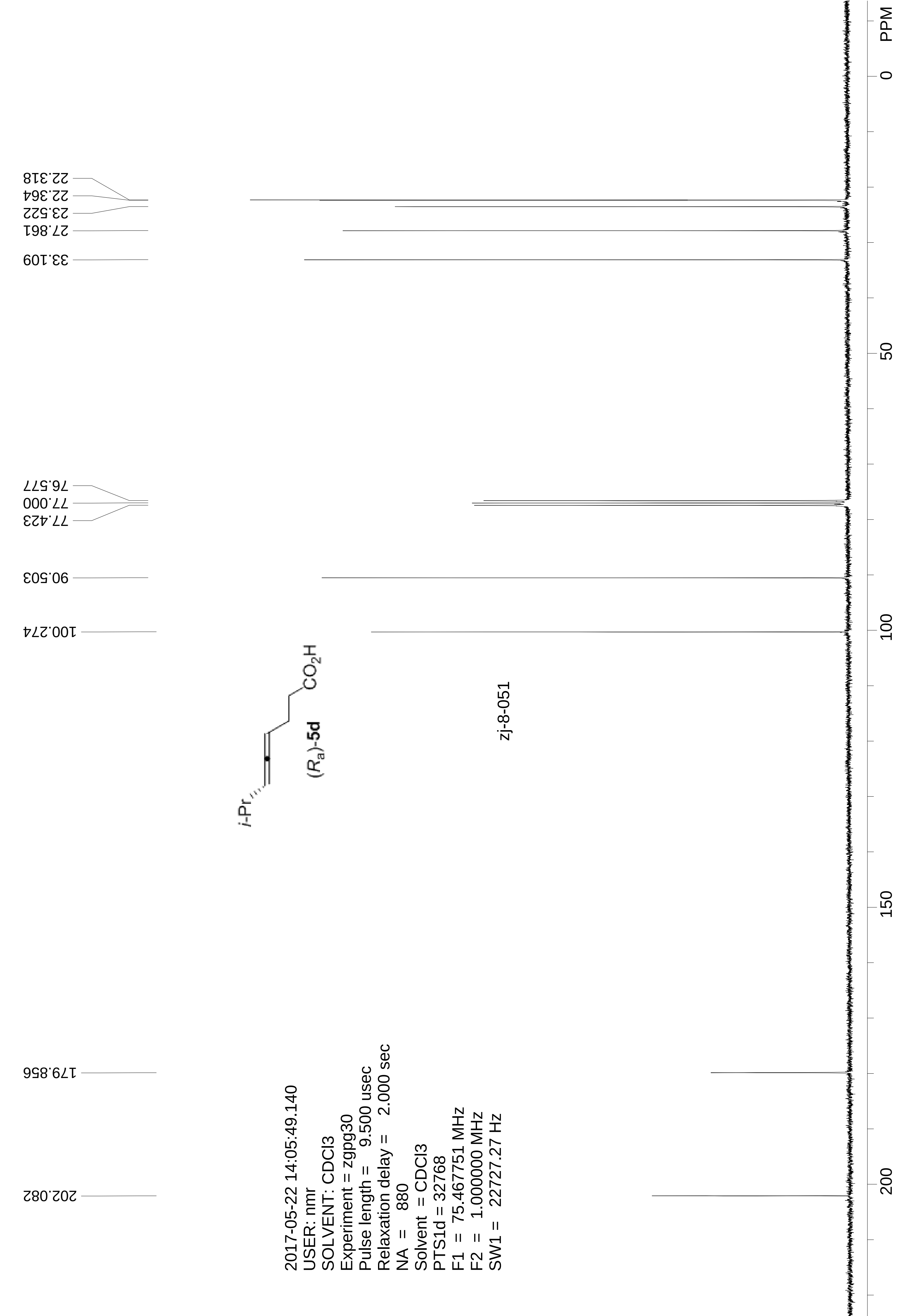

Supplementary Figure 28. ^{13}**C NMR (75 MHz, CDCl$_3$) spectrum for (*R*$_a$)-5d.**

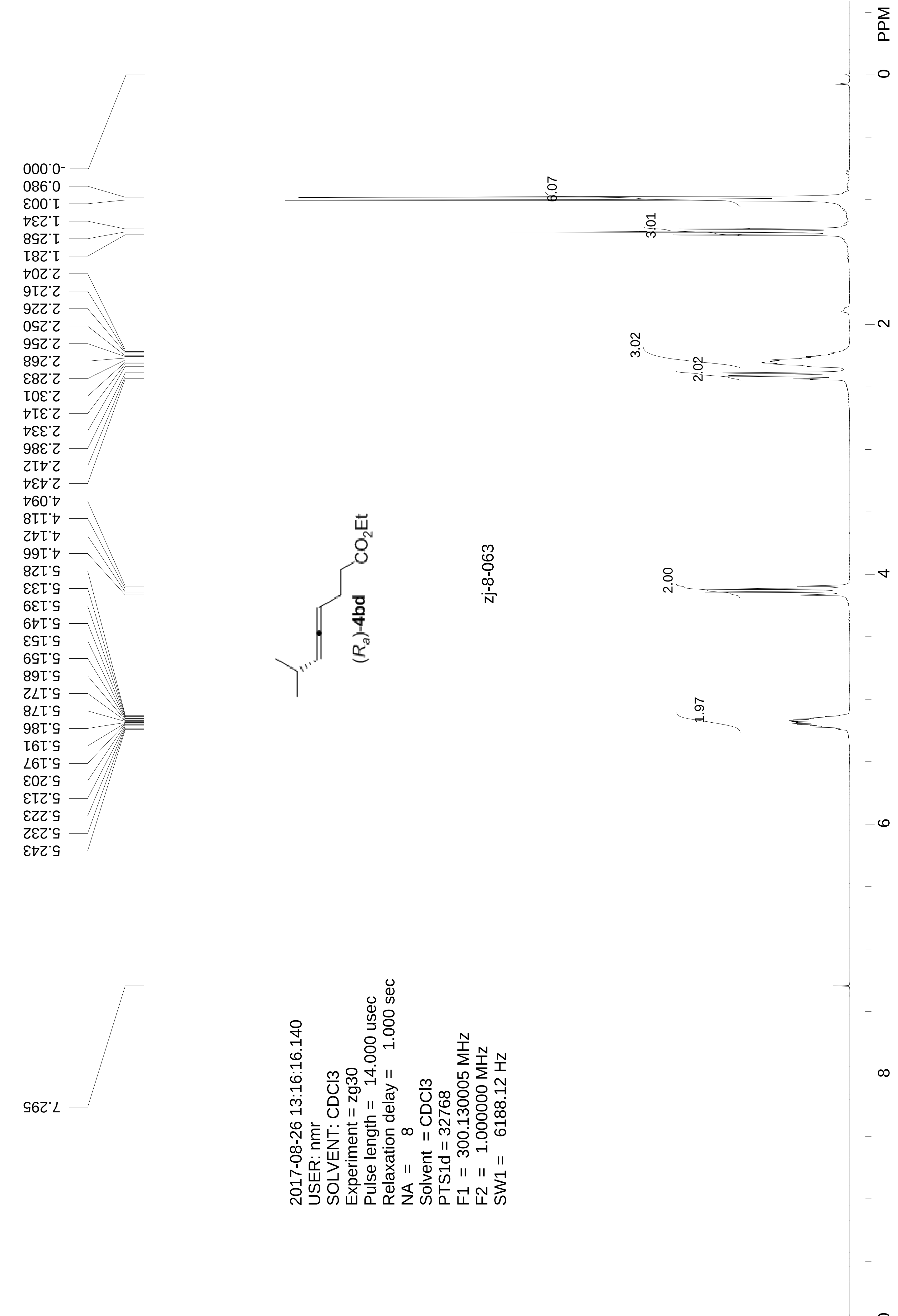

Supplementary Figure 29. ^{1}H NMR (300 MHz, $CDCl_3$) spectrum for (R_a)-4bd.

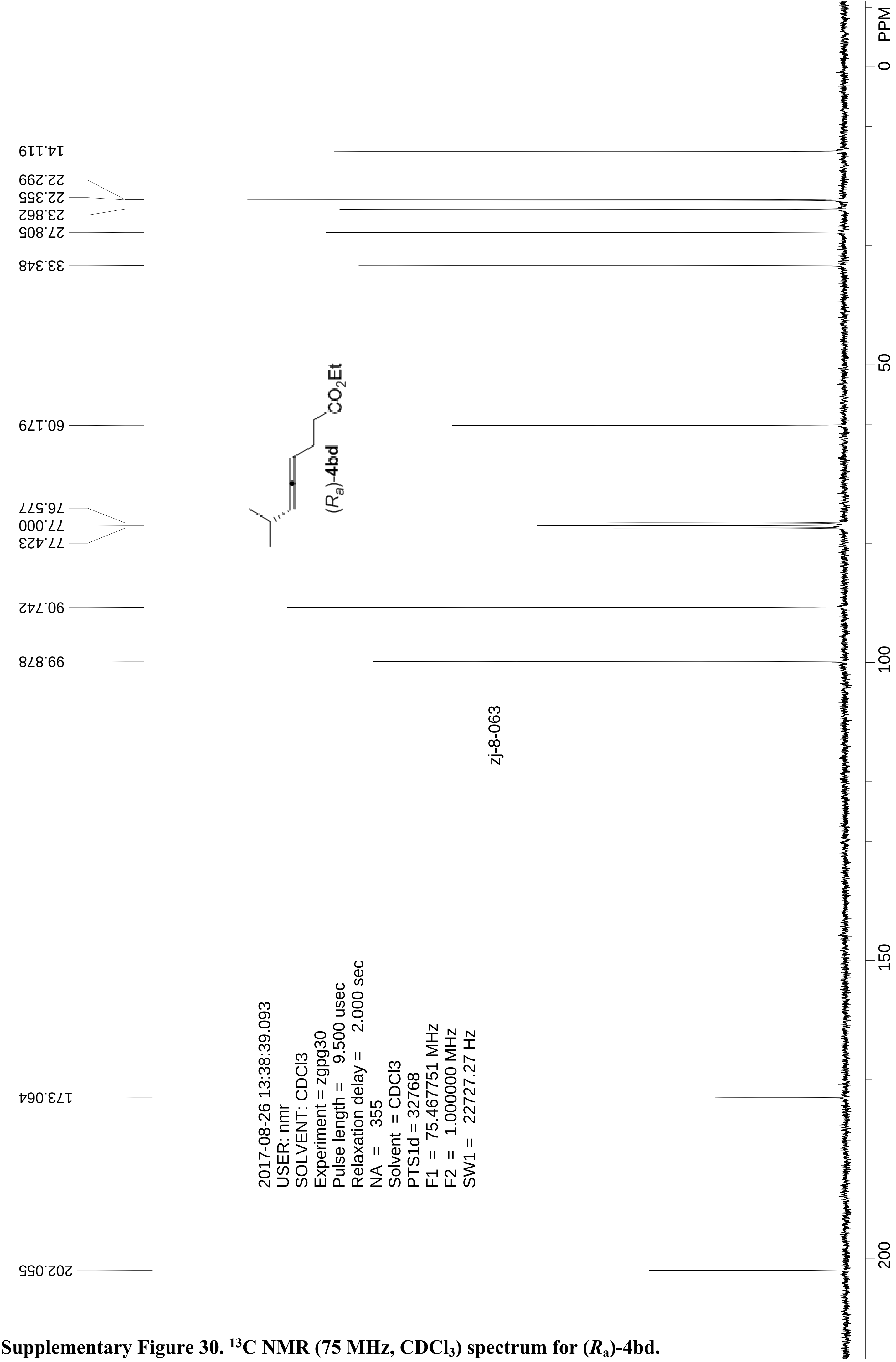

Supplementary Figure 30. ^{13}C NMR (75 MHz, $CDCl_3$) spectrum for (R_a)-4bd.

中国科学院上海有机化学研究所

Project Name: defaults for copy

Reported by User: Breeze user (Breeze)

SAMPLE INFORMATION

Sample Name:	zj-8-063-od-h-100-0-1-1214	Acquired By:	Breeze
Sample Type:	未知	Date Acquired:	2017/8/24 3:27:41 CST
Vial:	999	Acq. Method:	zgj100
Injection #:	3	Date Processed:	2017/8/24 8:02:28 CST
Injection Volume:	10.00 ul	Channel Name:	W2489 ChA
Run Time:	18.00 Minutes	Channel Desc.:	W2489 ChA 214nm
Column Type:		Sample Set Name:	

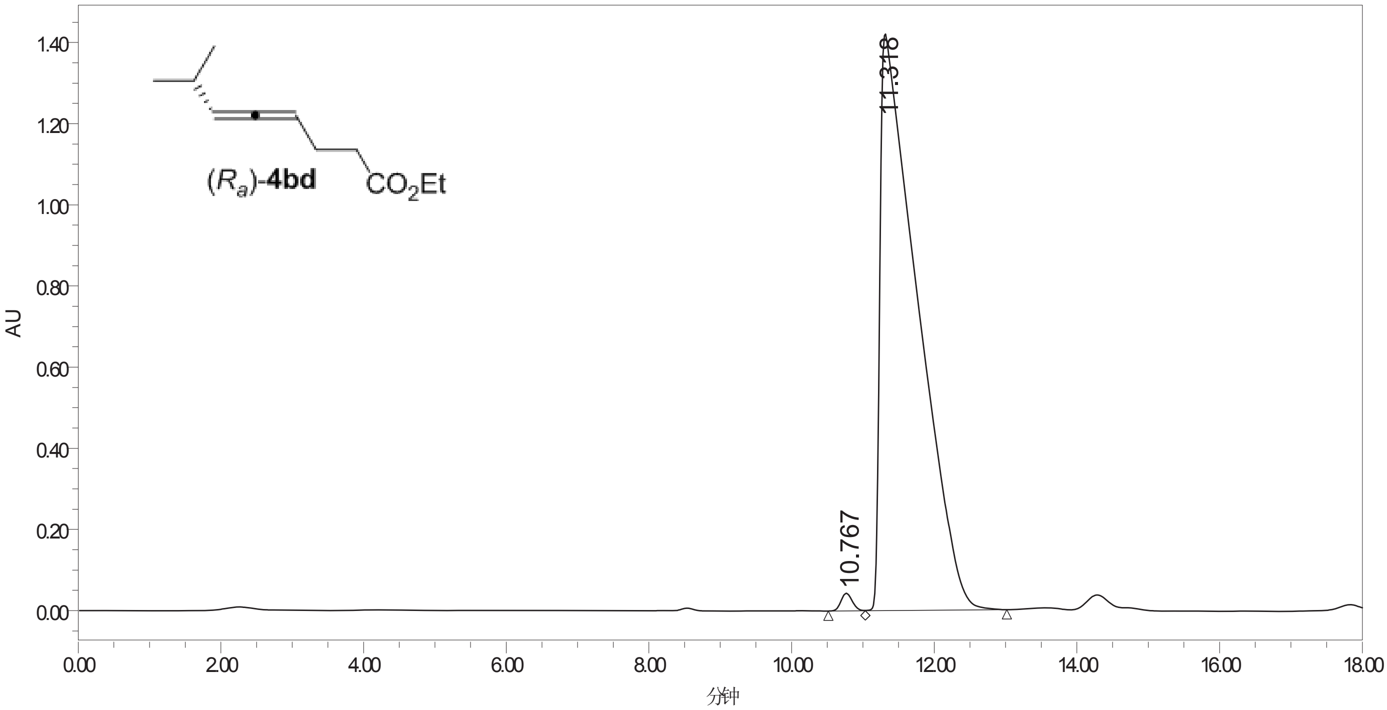

	RT (min)	Area (礦sec)	% Area	Height (礦)	% Height
1	10.767	478724	0.92	43506	2.97
2	11.318	51490950	99.08	1422087	97.03

Report Method: Individual Report ASC

Page: 1 (共计 1)

Printed: 2017/8/24 8:03:15 PRC

Supplementary Figure 31. HPLC spectrum for (R_a)-4bd.

中国科学院上海有机化学研究所

Project Name: defaults for copy

Reported by User: Breeze user (Breeze)

SAMPLE INFORMATION

Sample Name:	zj-8-047-od-h-100-0-1-1214	Acquired By:	Breeze
Sample Type:	未知	Date Acquired:	2017/8/24 3:05:51 CST
Vial:	999	Acq. Method:	zgj100
Injection #:	2	Date Processed:	2017/8/24 8:02:09 CST
Injection Volume:	10.00 ul	Channel Name:	W2489 ChA
Run Time:	18.00 Minutes	Channel Desc.:	W2489 ChA 214nm
Column Type:		Sample Set Name:	

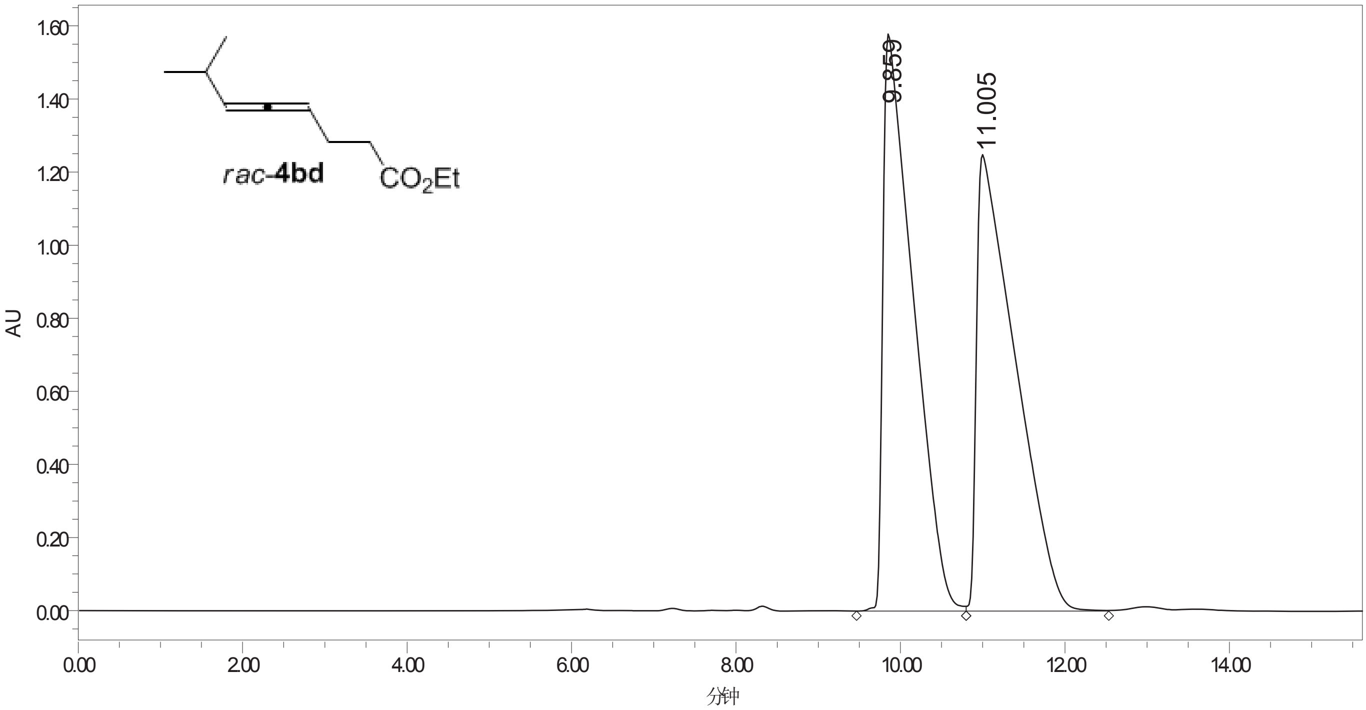

	RT (min)	Area (μV*sec)	% Area	Height (μV)	% Height
1	9.859	40242740	49.56	1586708	55.91
2	11.005	40952424	50.44	1251038	44.09

Supplementary Figure 32. HPLC spectrum for *rac*-4bd.

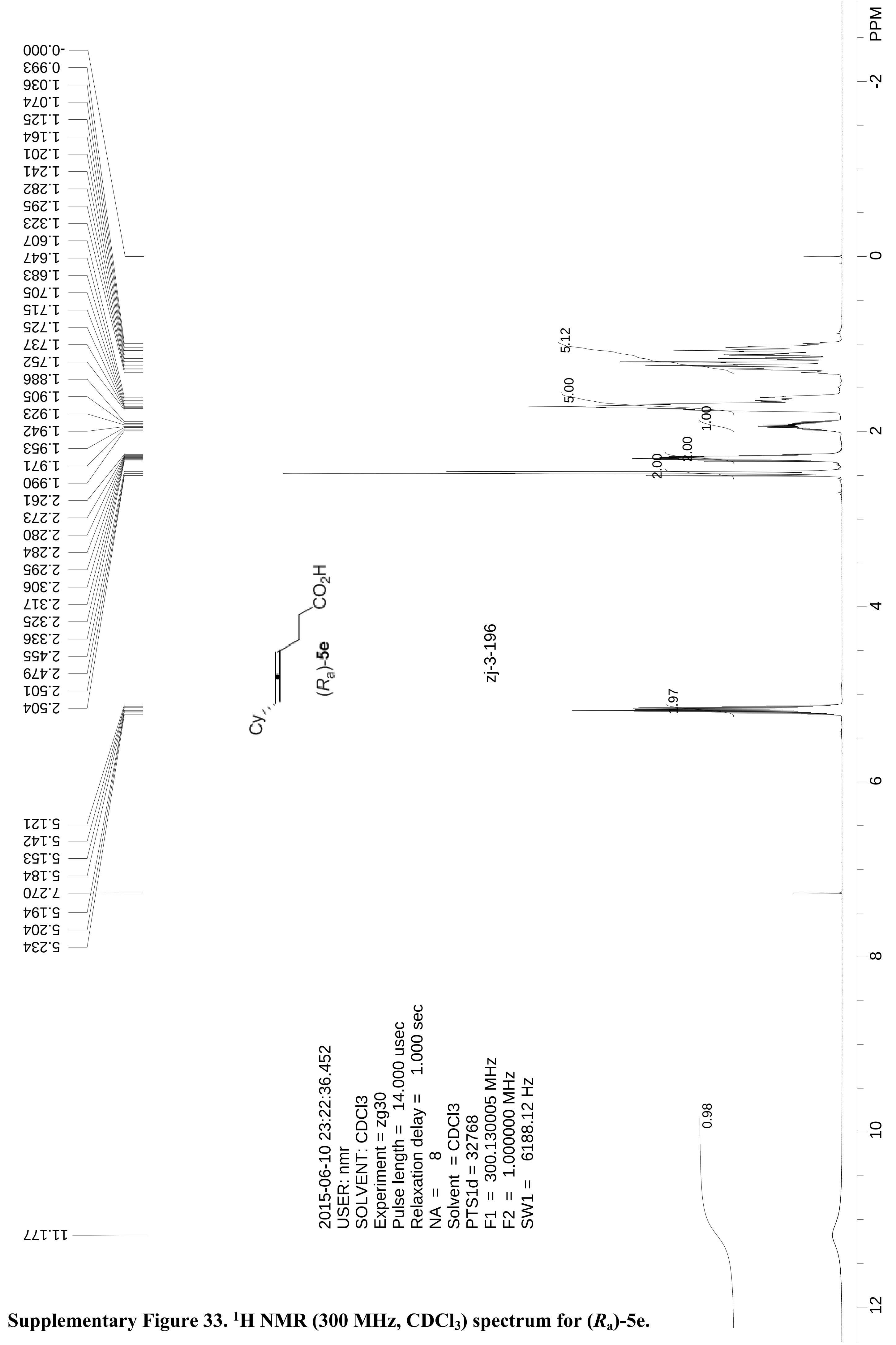

Supplementary Figure 33. ^{1}H NMR (300 MHz, CDCl$_3$) spectrum for (*R*$_a$)-5e.

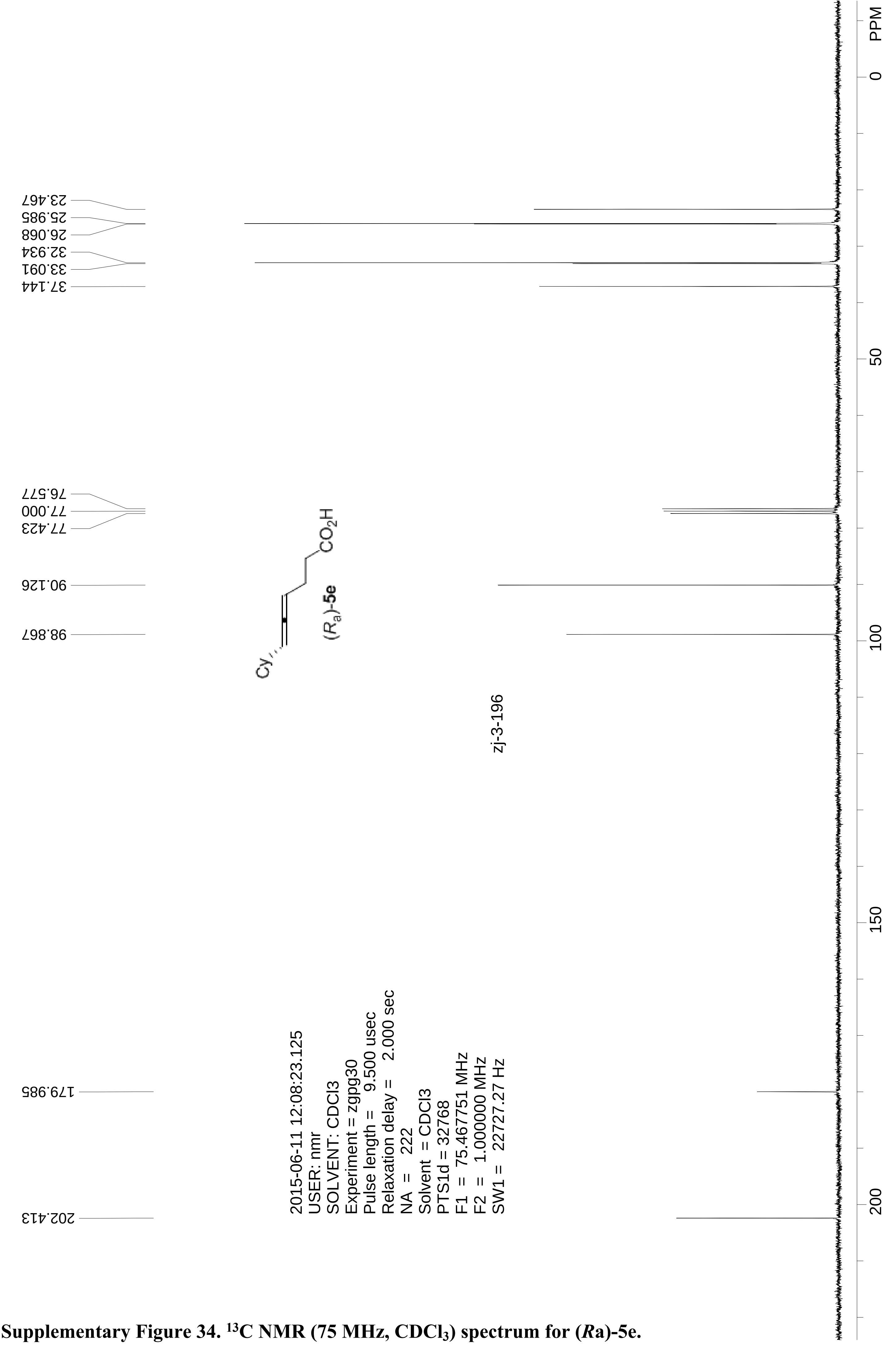

Supplementary Figure 34. ^{13}C NMR (75 MHz, $CDCl_3$) spectrum for (*R*a)-5e.

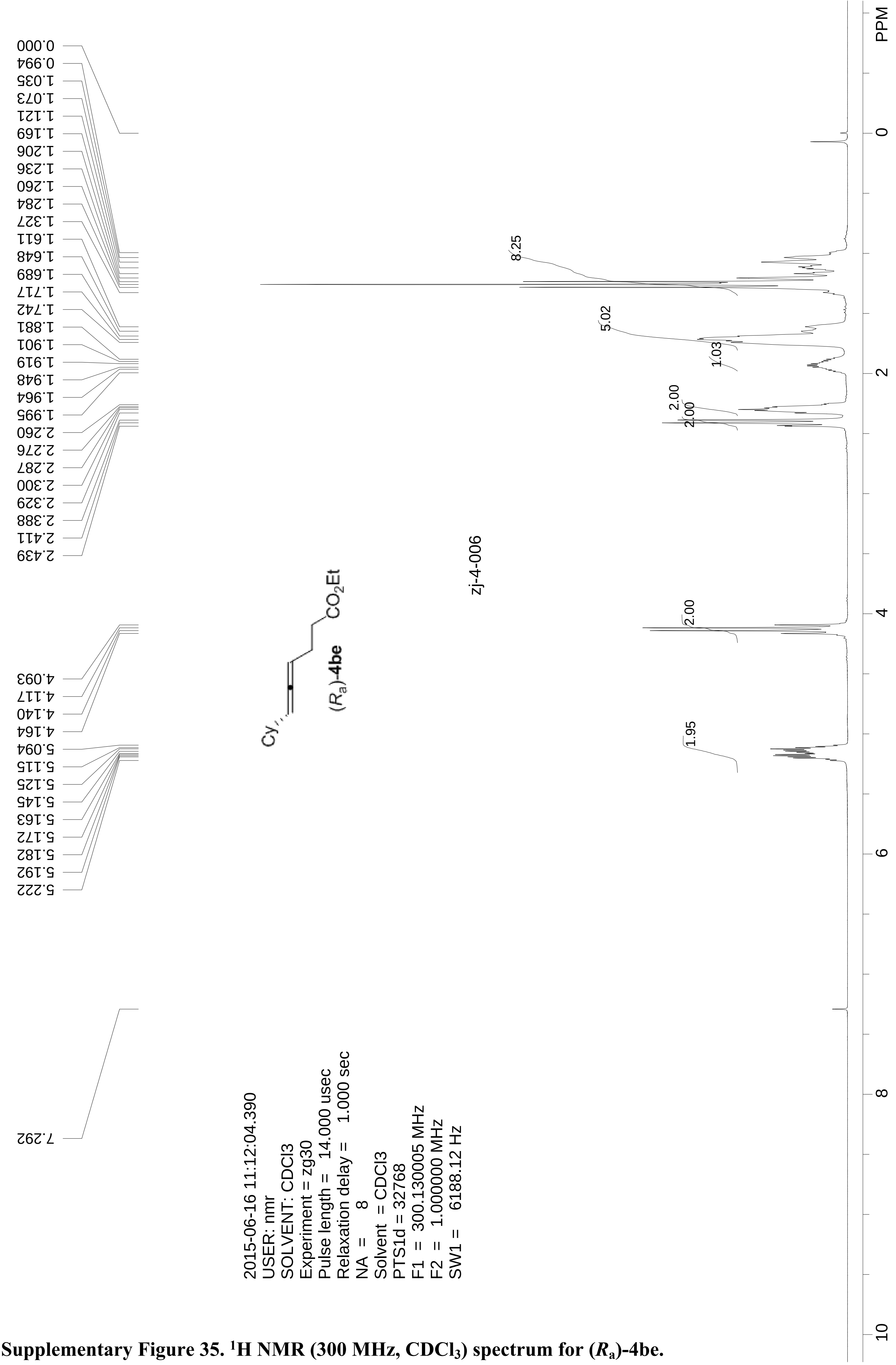

Supplementary Figure 35. ^{1}H NMR (300 MHz, $CDCl_3$) spectrum for (R_a)-4be.

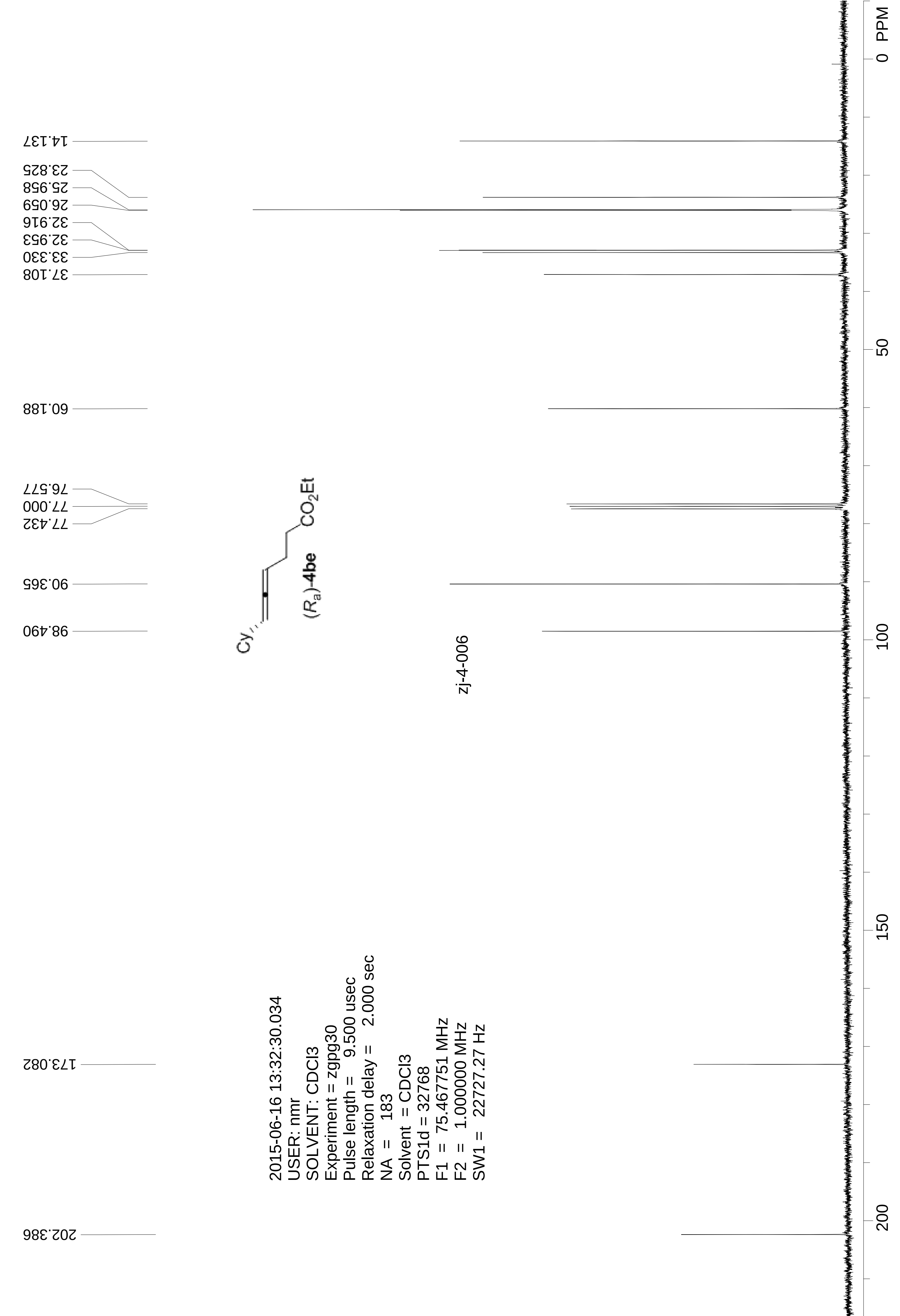

Supplementary Figure 36. ^{13}C NMR (75 MHz, $CDCl_3$) spectrum for (R_a)-4be.

zj-4-006-ay-100-0-0.6-214

实验时间：2015/7/9,12:05:37　　　　报告时间：2015/7/9,18:01:10
谱图文件:D:\zhuguangjiong\zj\20150706\zj-4-006-ay-100-0-0.6-214..org

实验内容简介：

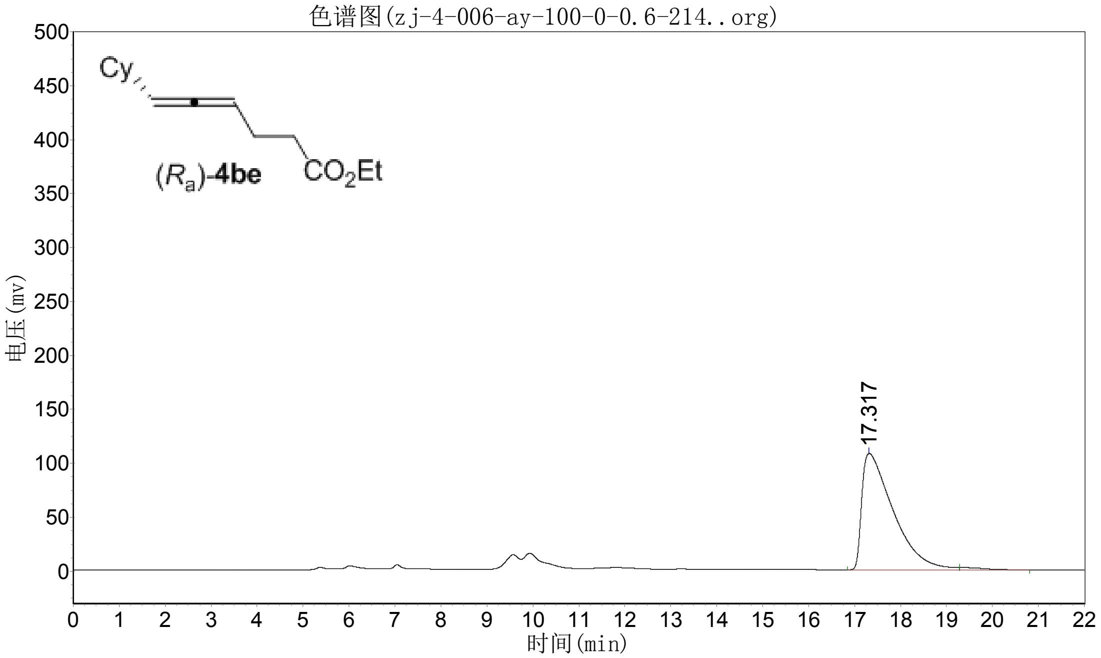

分析结果表

峰号	峰名	保留时间	峰高	峰面积	含量
1		17.317	107960.531	5137819.000	98.3231
2		17.317	2235.882	87625.016	1.6769
总计			110196.414	5225444.016	100.0000

Supplementary Figure 37. HPLC spectrum for (R_a)-4be.

zj-4-005-ay-100-0-0.6-214

实验时间：2015/7/9,11:06:55　　　　　　　　　　　　　　　报告时间：2015/7/9,17:59:35
谱图文件:D:\zhuguangjiong\zj\20150706\zj-4-005-ay-100-0-0.6-214.org

实验内容简介：

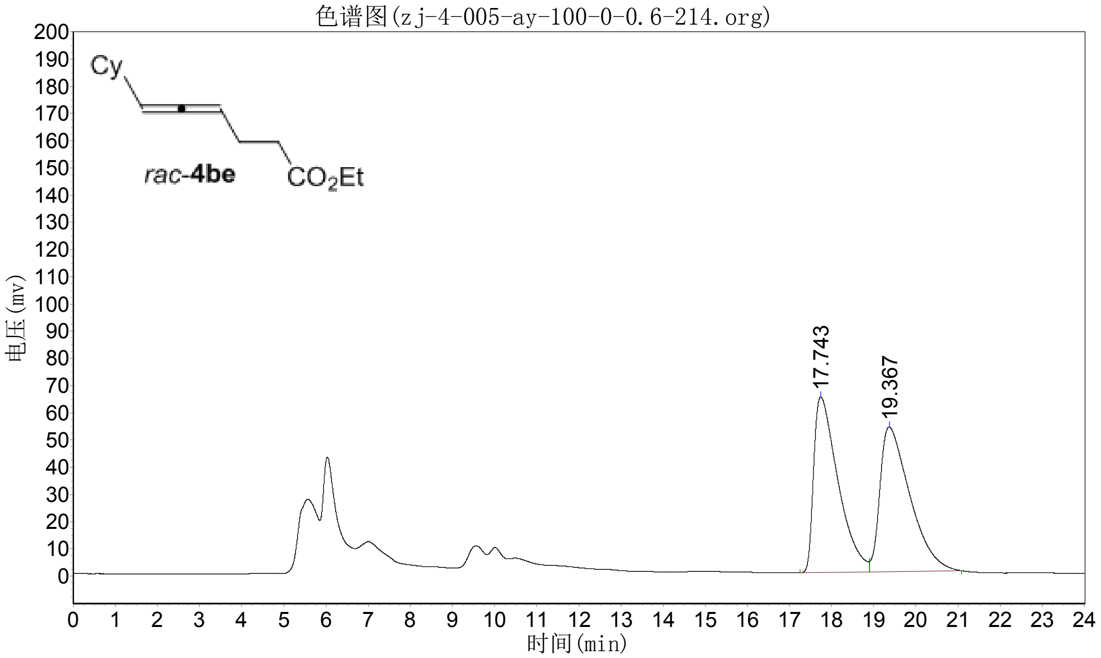

分析结果表

峰号	峰名	保留时间	峰高	峰面积	含量
1		17.743	64498.359	2592152.750	49.7180
2		19.367	53120.230	2621561.000	50.2820
总计			117618.590	5213713.750	100.0000

Supplementary Figure 38. HPLC spectrum for *rac*-4be.

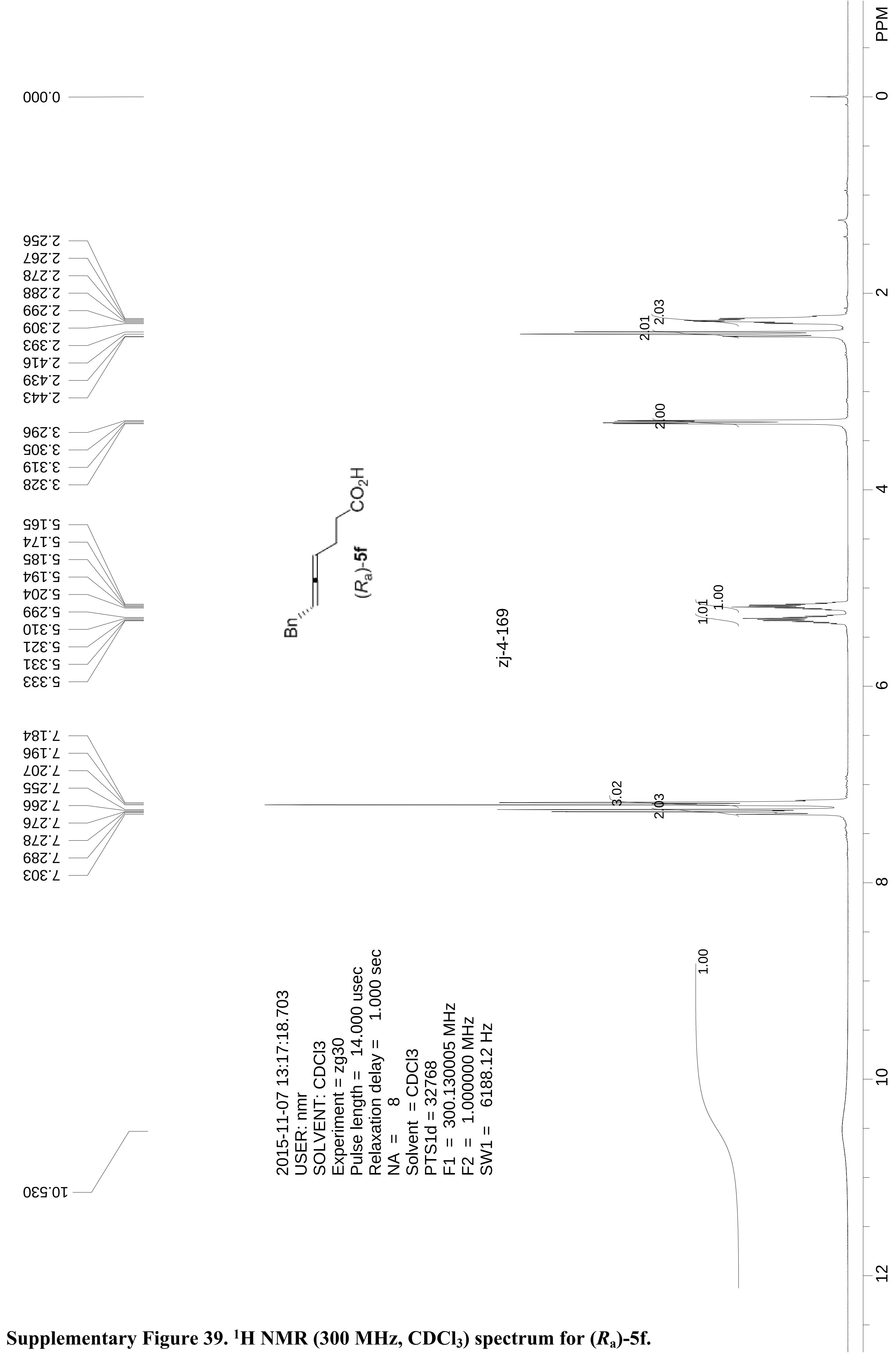

Supplementary Figure 39. ^{1}H NMR (300 MHz, $CDCl_3$) spectrum for (R_a)-5f.

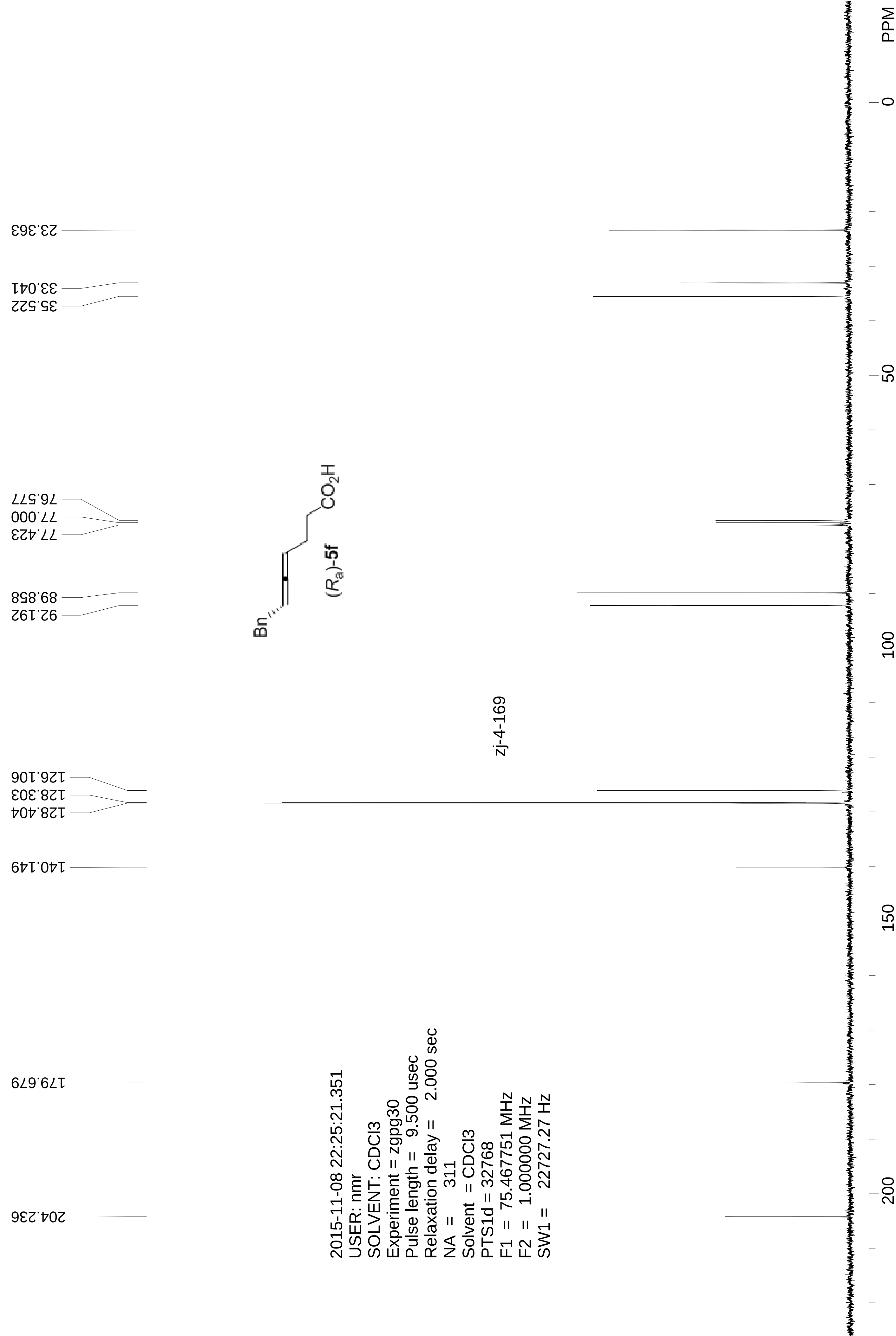

Supplementary Figure 40. ^{13}C NMR (75 MHz, $CDCl_3$) spectrum for (*R*$_a$)-5f.

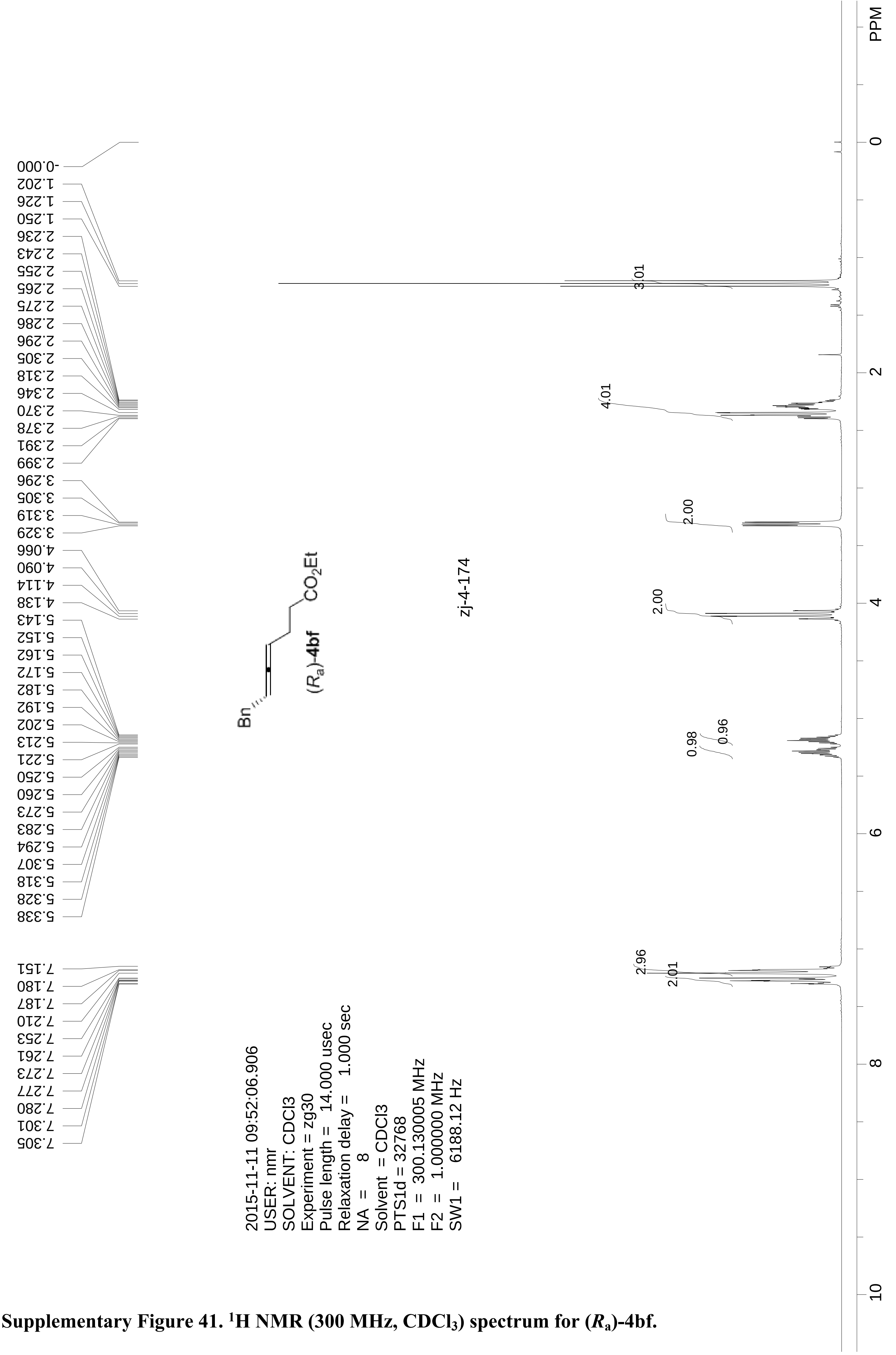

Supplementary Figure 41. ^{1}H NMR (300 MHz, $CDCl_3$) spectrum for (R_a)-4bf.

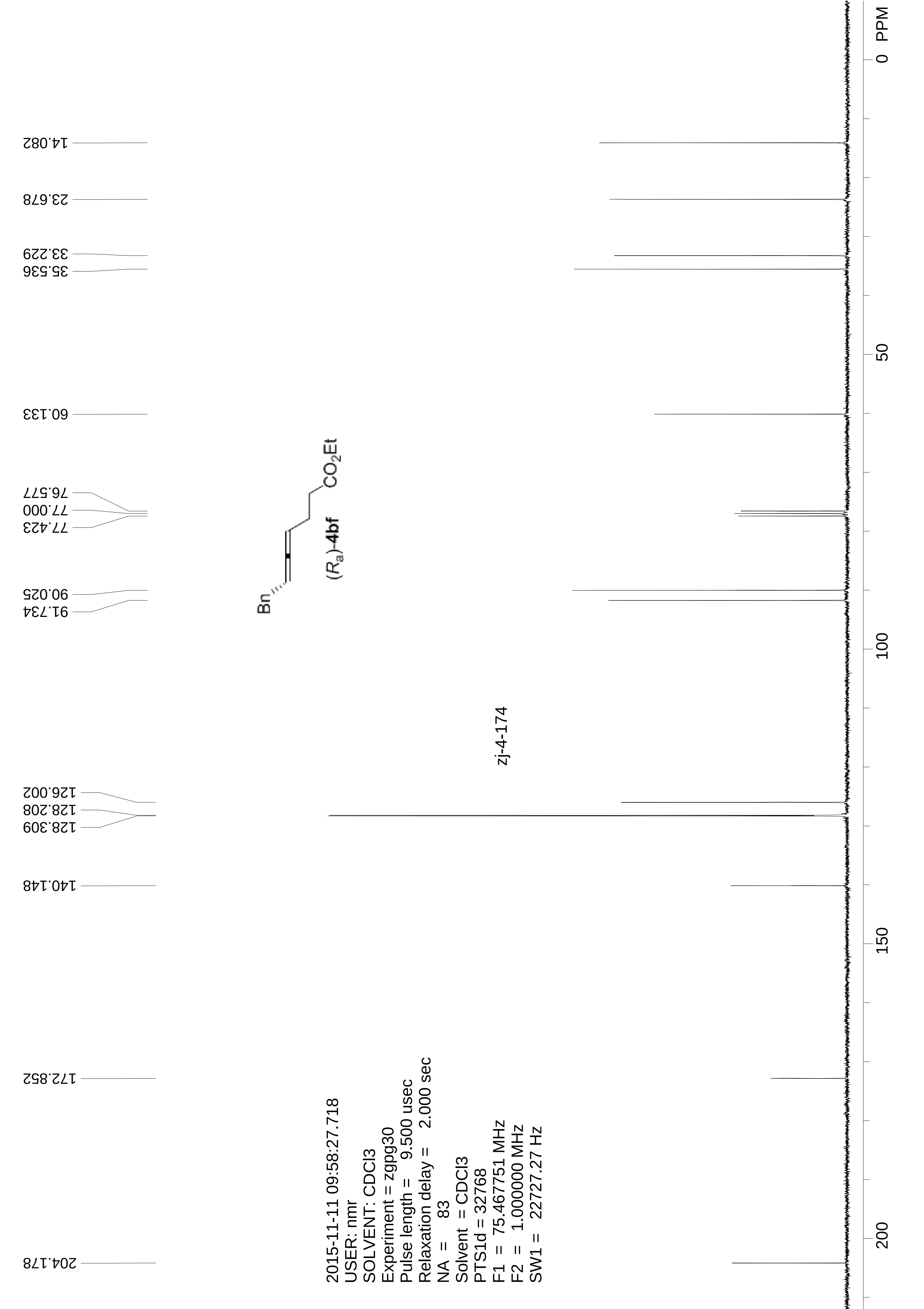

Supplementary Figure 42. ^{13}C NMR (75 MHz, $CDCl_3$) spectrum for (R_a)-4bf.

zj-4-174-as-h-100-0-1-214

实验时间：2015/11/17,11:15:06　　　　　　　　　　　　报告时间：2015/11/17,16:56:02
谱图文件:D:\zhuguangjiong\zj\20151117\zj-4-174-as-h-100-0-1-214..org

实验内容简介：

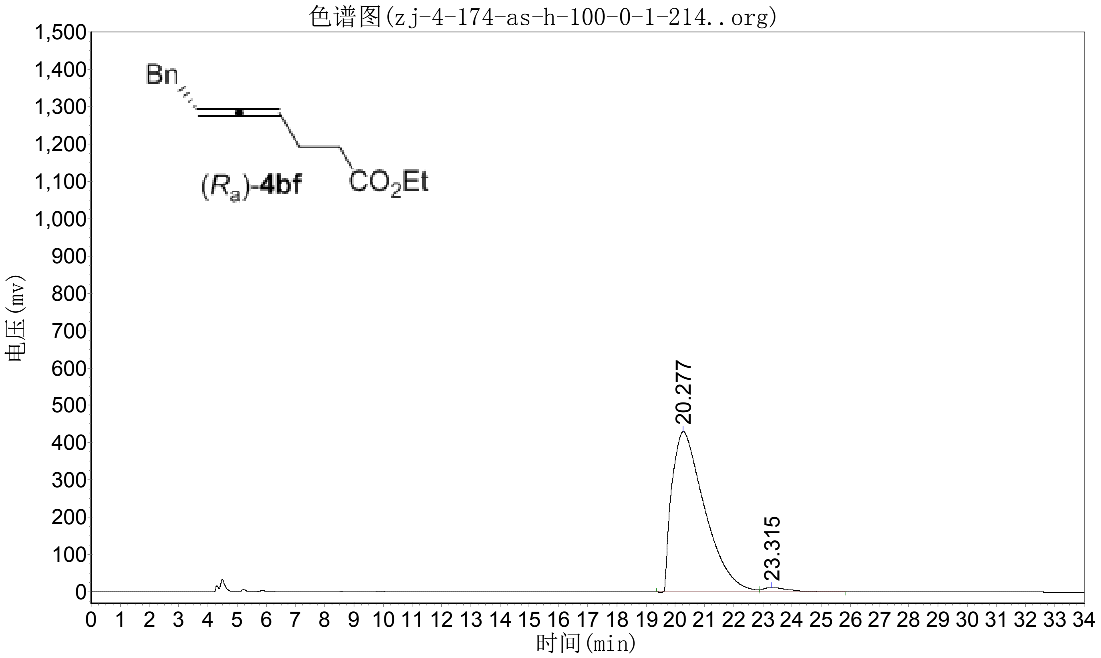

分析结果表

峰号	峰名	保留时间	峰高	峰面积	含量
1		20.277	430079.688	33621564.000	97.7530
2		23.315	11803.181	772857.188	2.2470
总计			441882.868	34394421.188	100.0000

Supplementary Figure 43. HPLC spectrum for (R_a)-4bf.

zj-4-021-as-h-100-0-1-214

实验时间：2015/11/17,10:10:53　　报告时间：2015/11/17,16:54:51
谱图文件:D:\zhuguangjiong\zj\20151117\zj-4-021-as-h-100-0-1-214.org

实验内容简介：

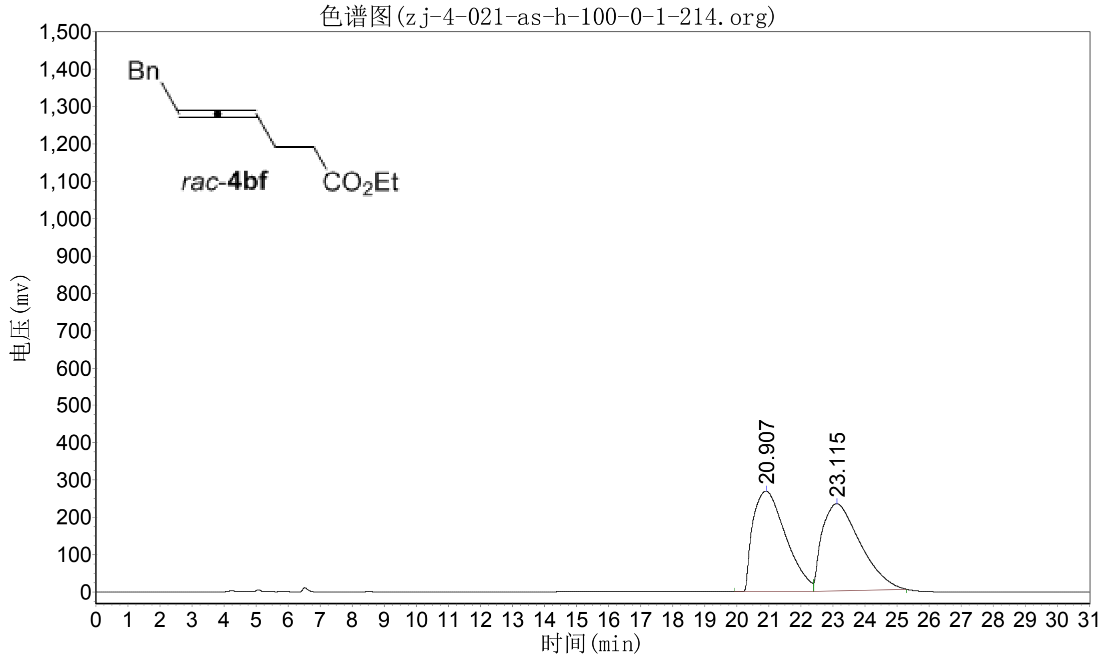

分析结果表

峰号	峰名	保留时间	峰高	峰面积	含量
1		20.907	269109.406	19191692.000	49.8792
2		23.115	233418.578	19284672.000	50.1208
总计			502527.984	38476364.000	100.0000

Supplementary Figure 44. HPLC spectrum for *rac*-4bf.

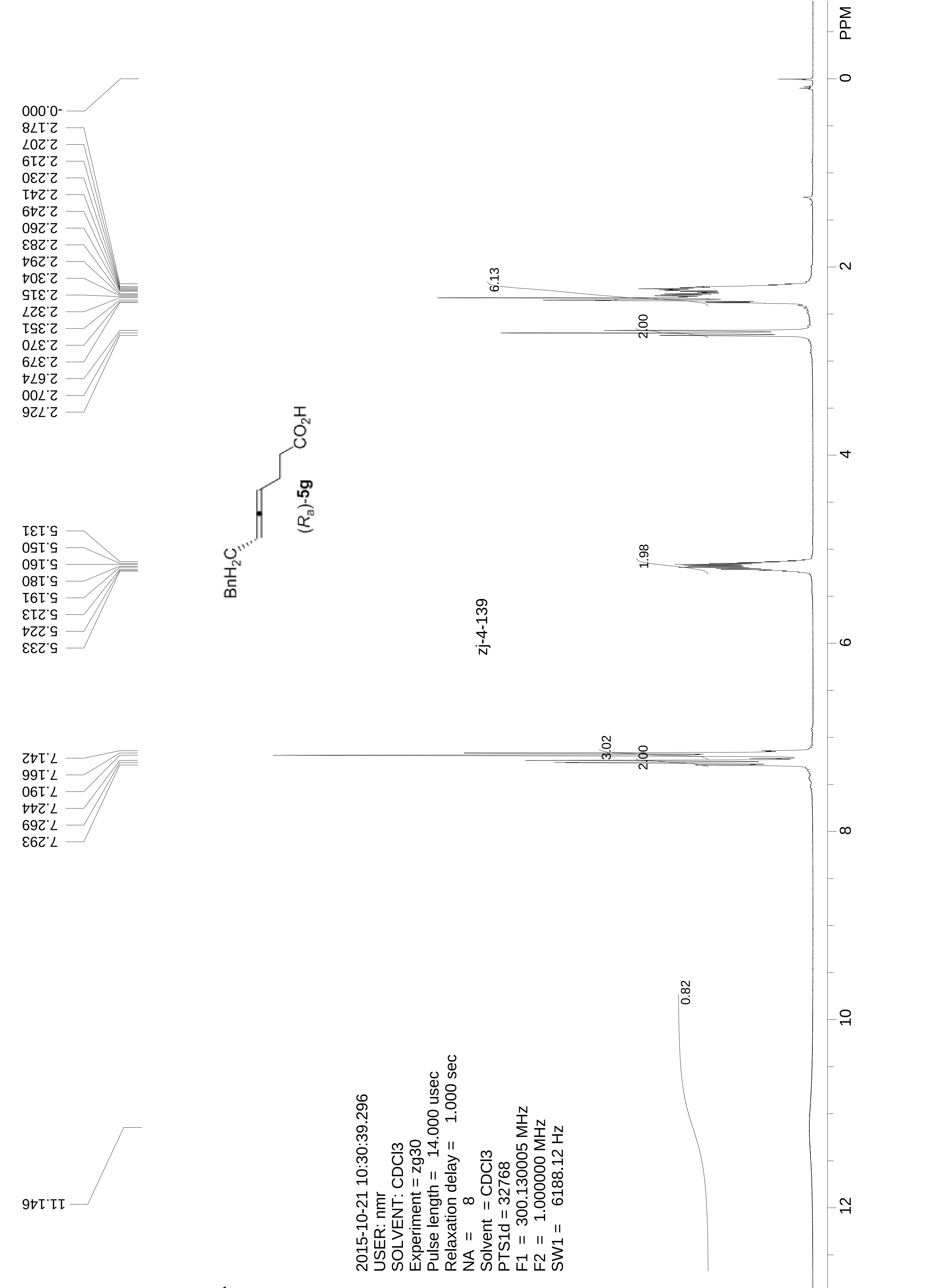

Supplementary Figure 45. ¹H NMR (300 MHz, CDCl$_3$) spectrum for (R_a)-5g.

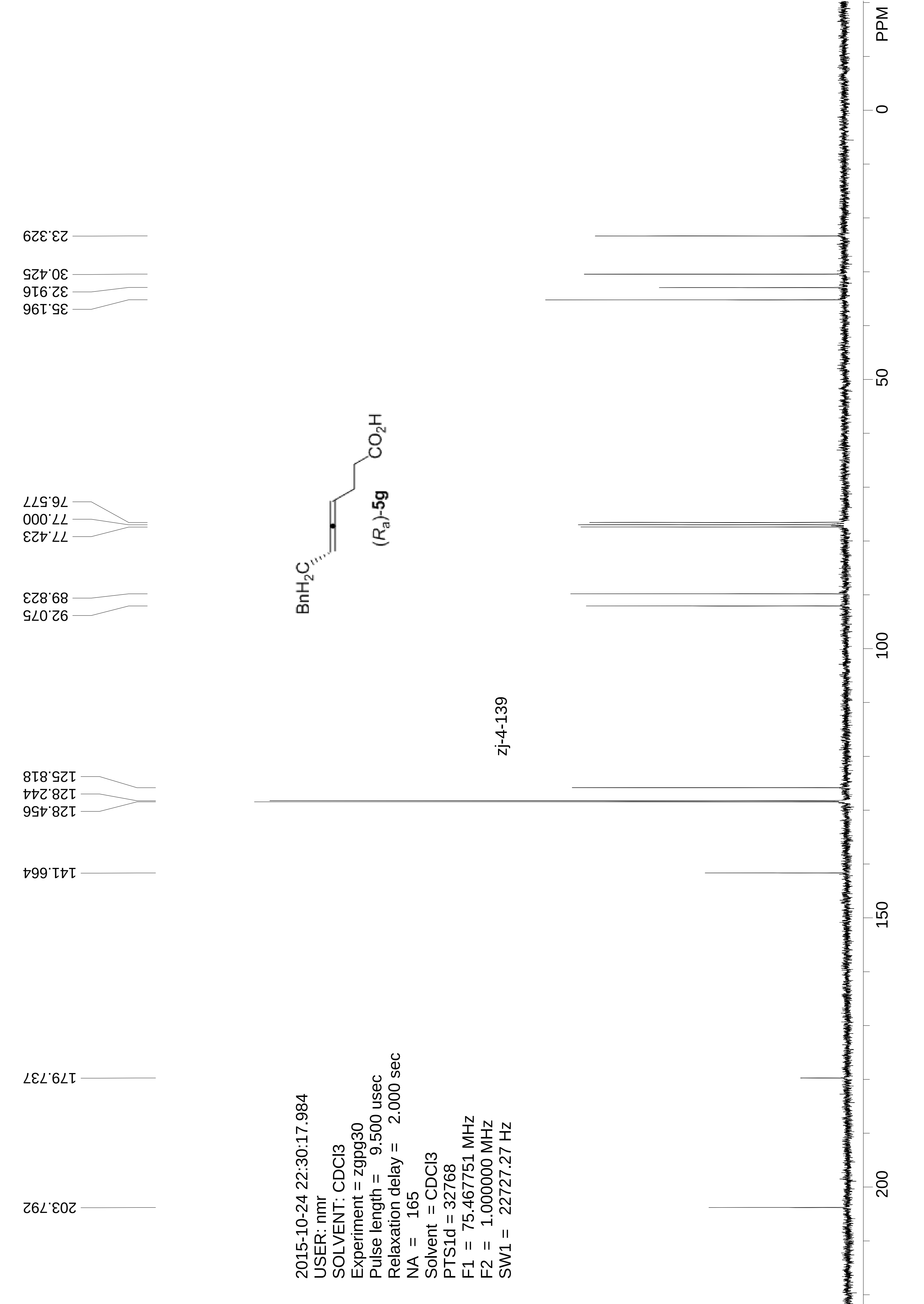

Supplementary Figure 46. ^{13}C **NMR (75 MHz,** $CDCl_3$**) spectrum for (*R*a)-5g.**

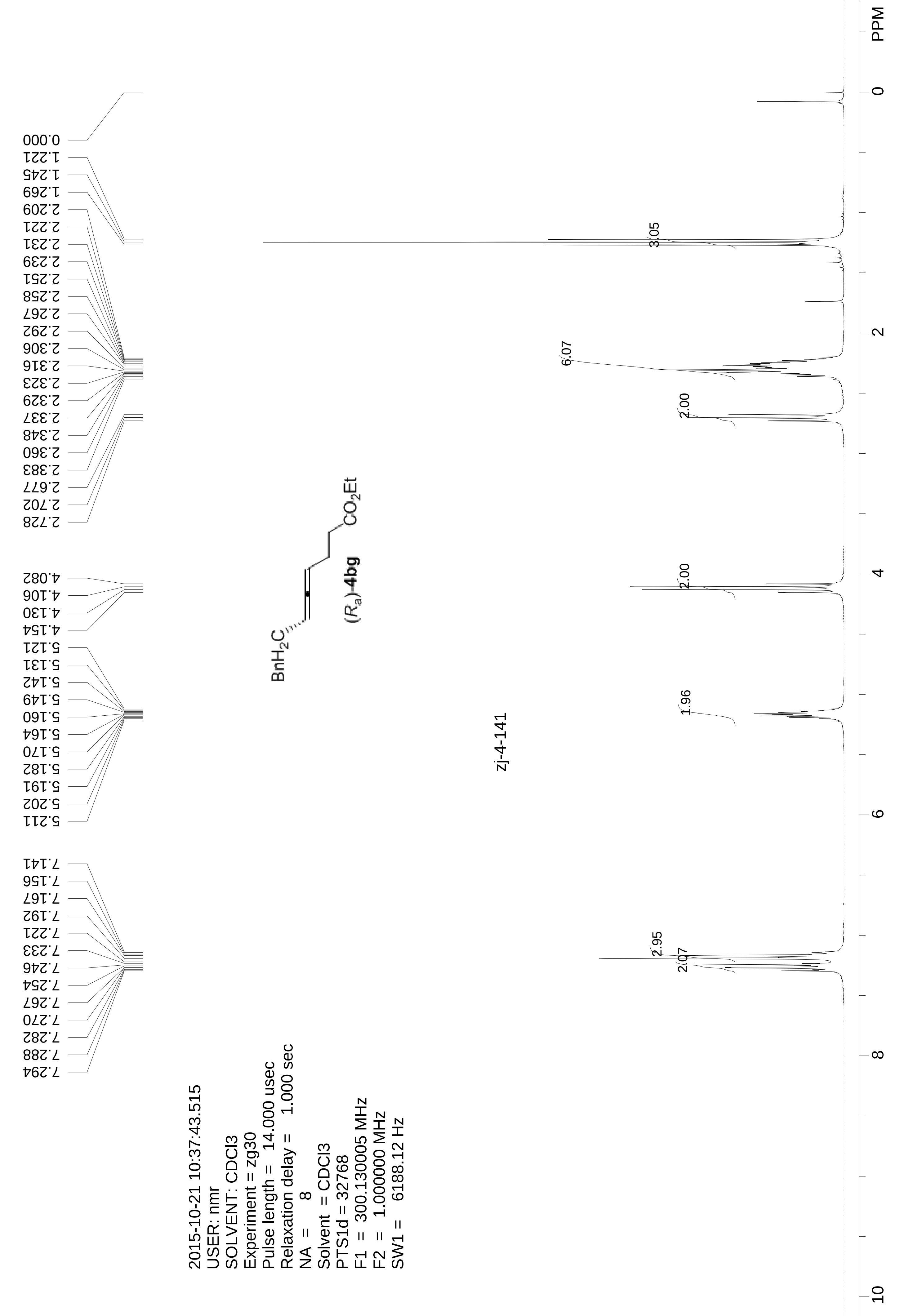

Supplementary Figure 47. ^{1}H NMR (300 MHz, $CDCl_3$) spectrum for (R_a)-4bg.

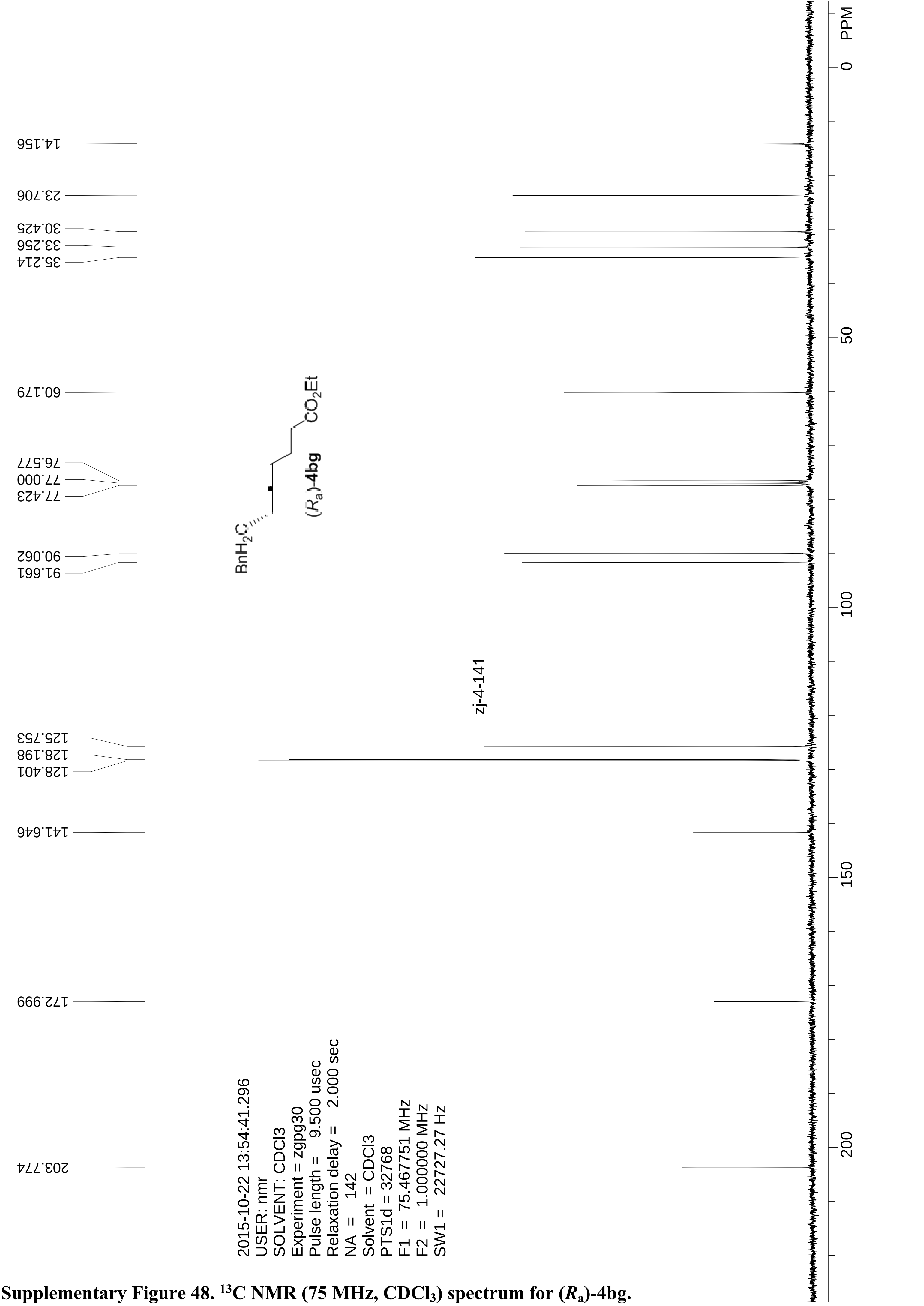

Supplementary Figure 48. ^{13}C NMR (75 MHz, $CDCl_3$) spectrum for (R_a)-4bg.

zj-4-141-as-h-100-0-0.7-214

实验时间：2015-11-04,12:42:36　　　　报告时间：2015-11-04,13:53:04
谱图文件:D:\zhuguangjiong\zj\20151103\zj-4-141-as-h-100-0-0.7-214...org

实验内容简介：

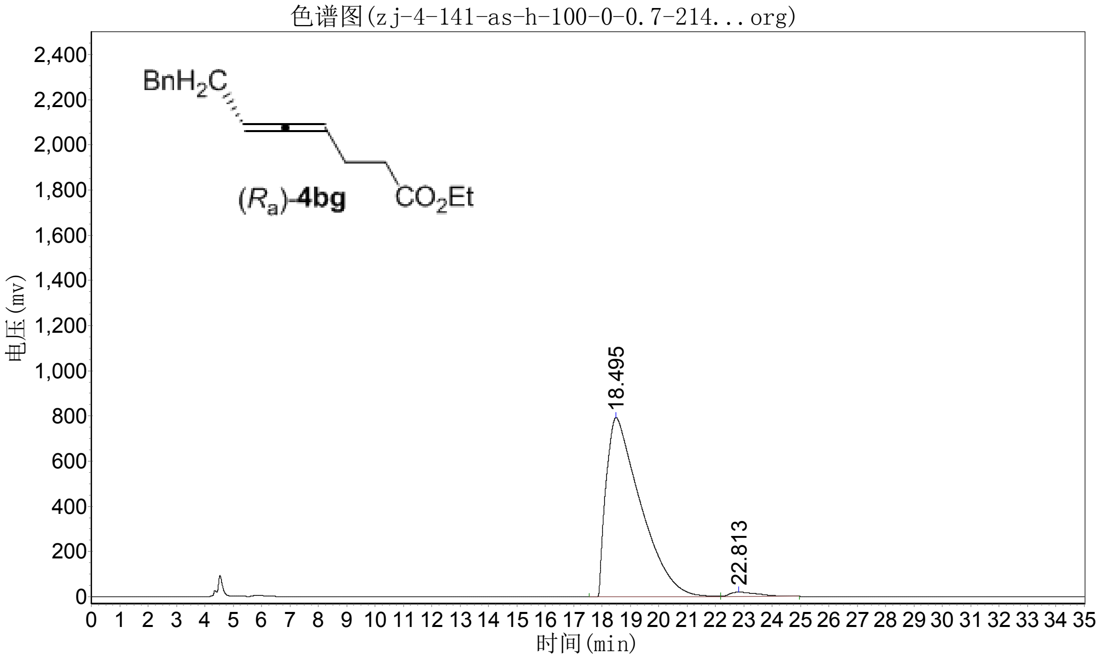

分析结果表

峰号	峰名	保留时间	峰高	峰面积	含量
1		18.495	793333.813	66945280.000	97.9254
2		22.813	19756.412	1418243.375	2.0746
总计			813090.225	68363523.375	100.0000

Supplementary Figure 49. HPLC spectrum for (R_a)-4bg.

zj-4-142-as-h-100-0-0.7-214

实验时间：2015-11-04,10:35:23　　　　报告时间：2015-11-04,13:52:18
谱图文件:d:\zhuguangjiong\zj\20151103\zj-4-142-as-h-100-0-0.7-214.org

实验内容简介：

色谱图(zj-4-142-as-h-100-0-0.7-214.org)

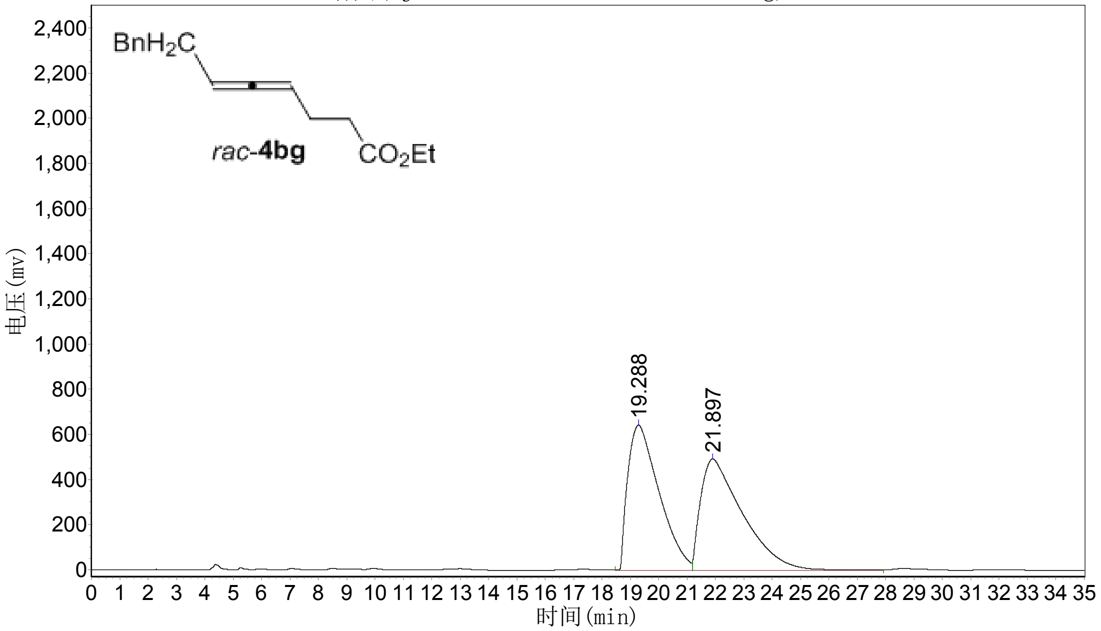

分析结果表

峰号	峰名	保留时间	峰高	峰面积	含量
1		19.288	642363.875	49289944.000	49.4214
2		21.897	492664.531	50444076.000	50.5786
总计			1135028.406	99734020.000	100.0000

Supplementary Figure 50. HPLC spectrum for *rac*-4bg.

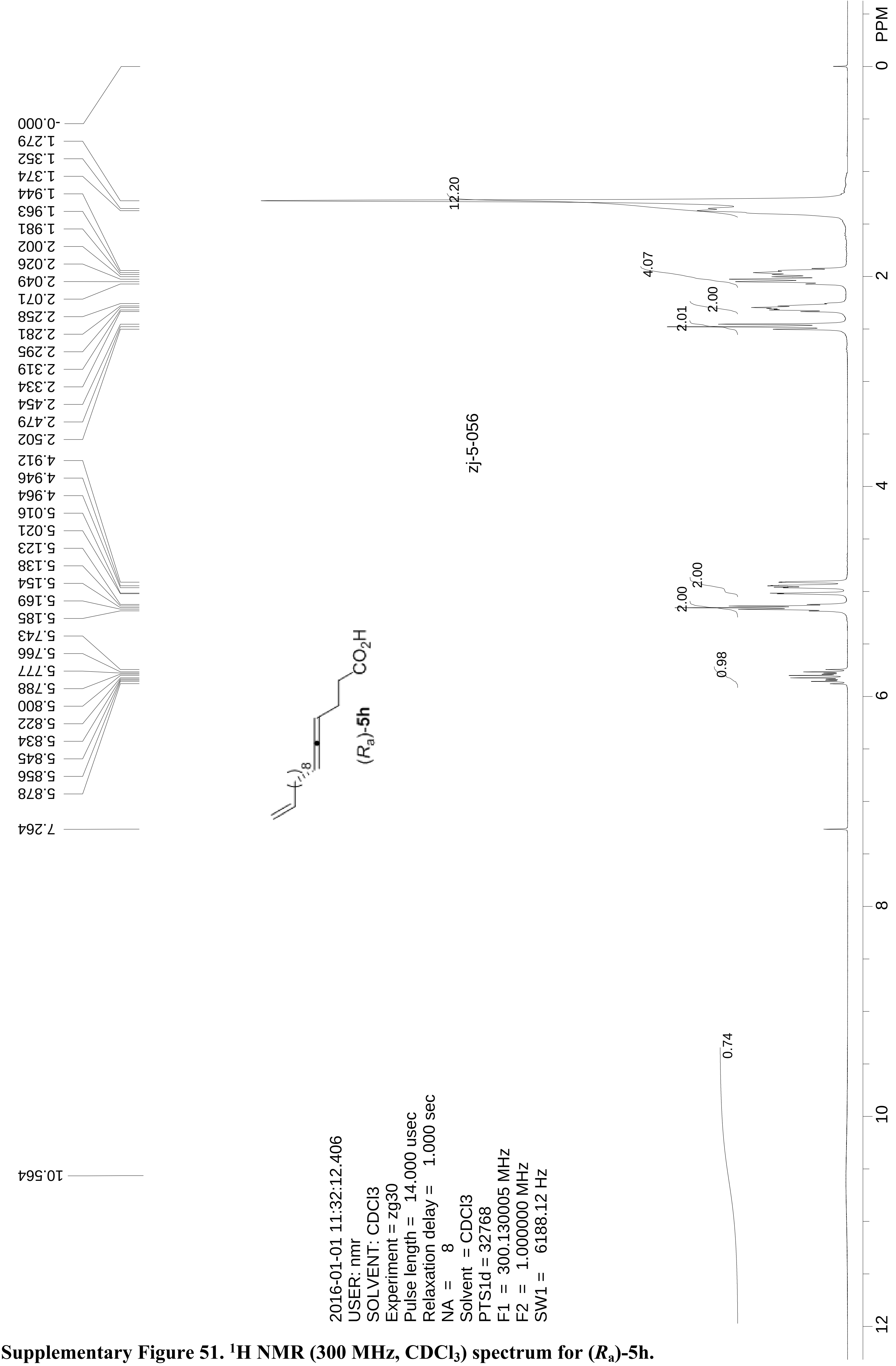

Supplementary Figure 51. ^{1}**H NMR (300 MHz, CDCl$_3$) spectrum for (*R*$_a$)-5h.**

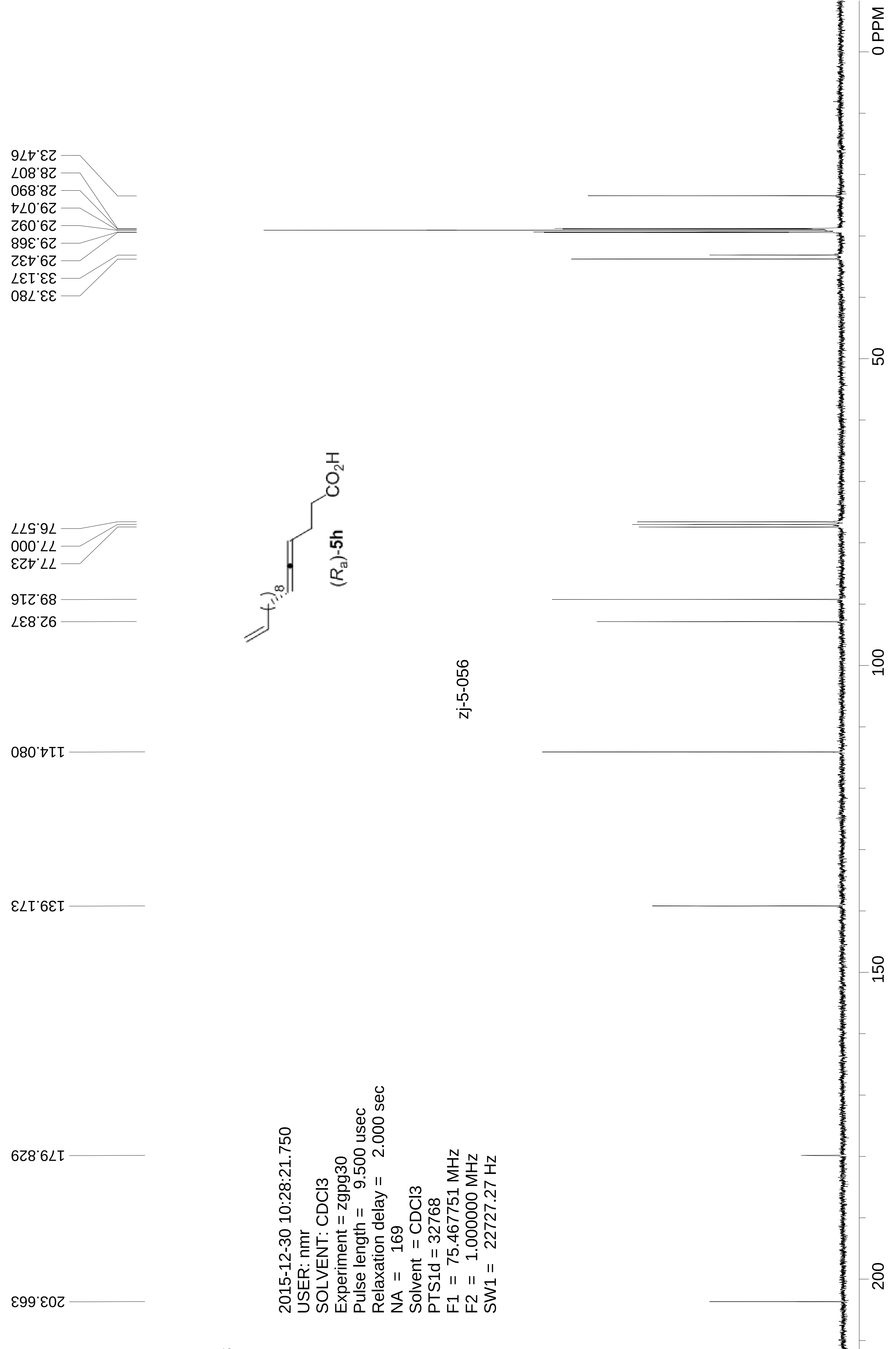

Supplementary Figure 52. ^{13}C NMR (75 MHz, $CDCl_3$) spectrum for (R_a)-5h.

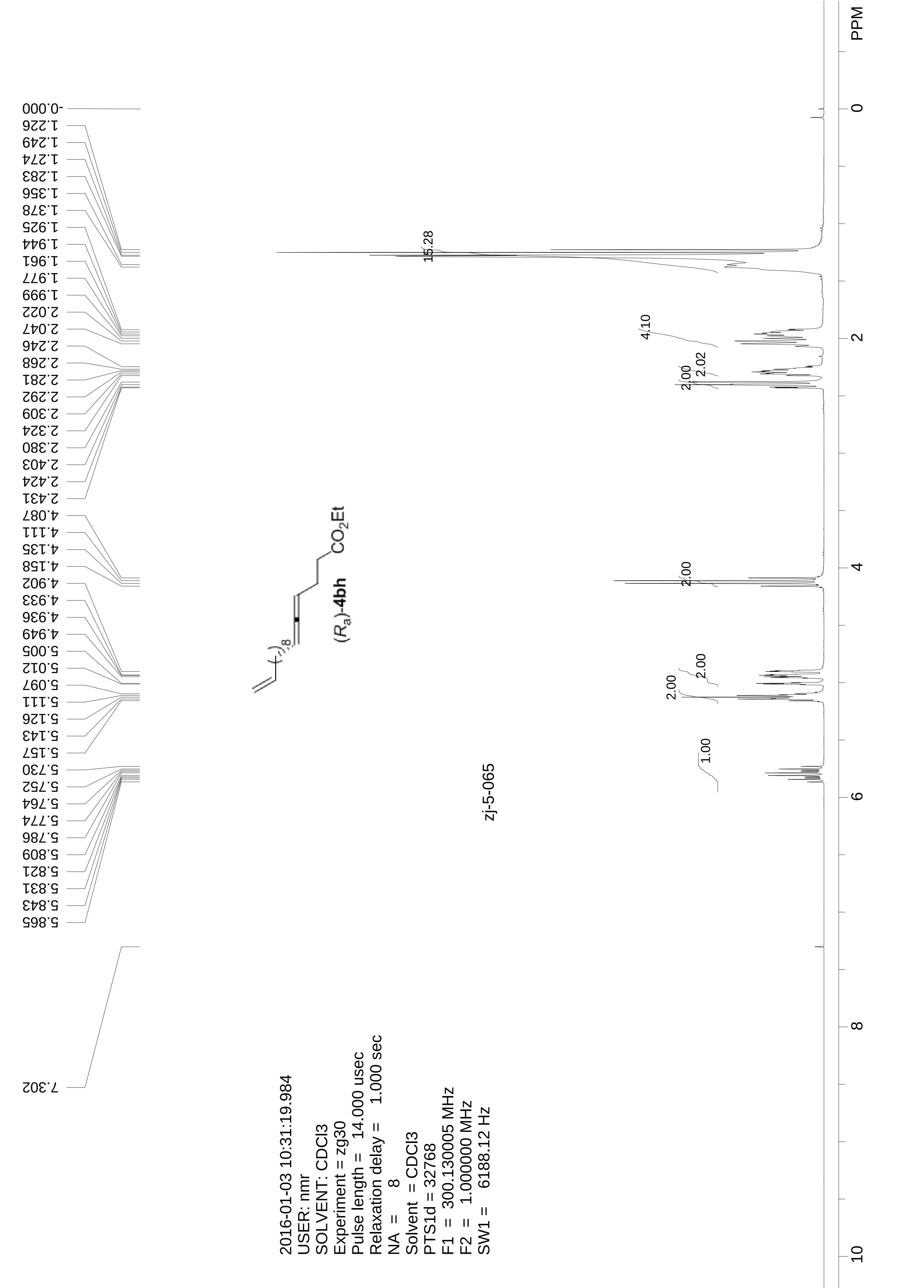

Supplementary Figure 53. ^{1}H NMR (300 MHz, $CDCl_3$) spectrum for (R_a)-4bh.

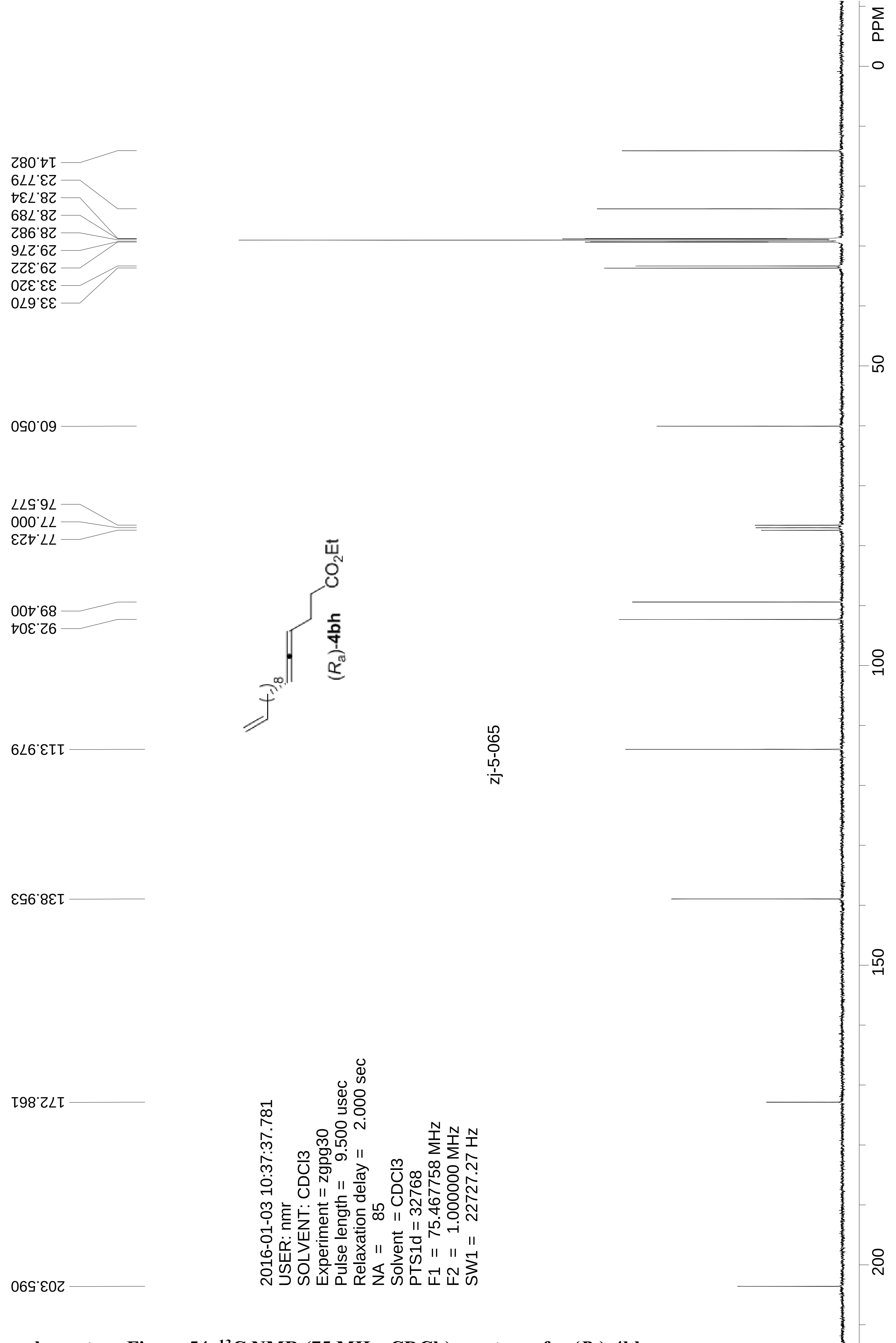

Supplementary Figure 54. ^{13}C NMR (75 MHz, $CDCl_3$) spectrum for (R_a)-4bh.

中国科学院上海有机化学研究所

Project Name: defaults for copy

Reported by User: Breeze user (Breeze)

Breeze 2 HPLC System

SAMPLE INFORMATION

Sample Name:	zj-5-065-oz-100-0-0.5-214	Acquired By:	Breeze
Sample Type:	未知	Date Acquired:	2016/3/28 12:58:28 CST
Vial:	21	Acq. Method:	zgj100
Injection #:	1	Date Processed:	2016/3/28 13:57:58 CST
Injection Volume:	10.00 ul	Channel Name:	W2489 ChA
Run Time:	30.00 Minutes	Channel Desc.:	W2489 ChA 214nm
Column Type:		Sample Set Name:	

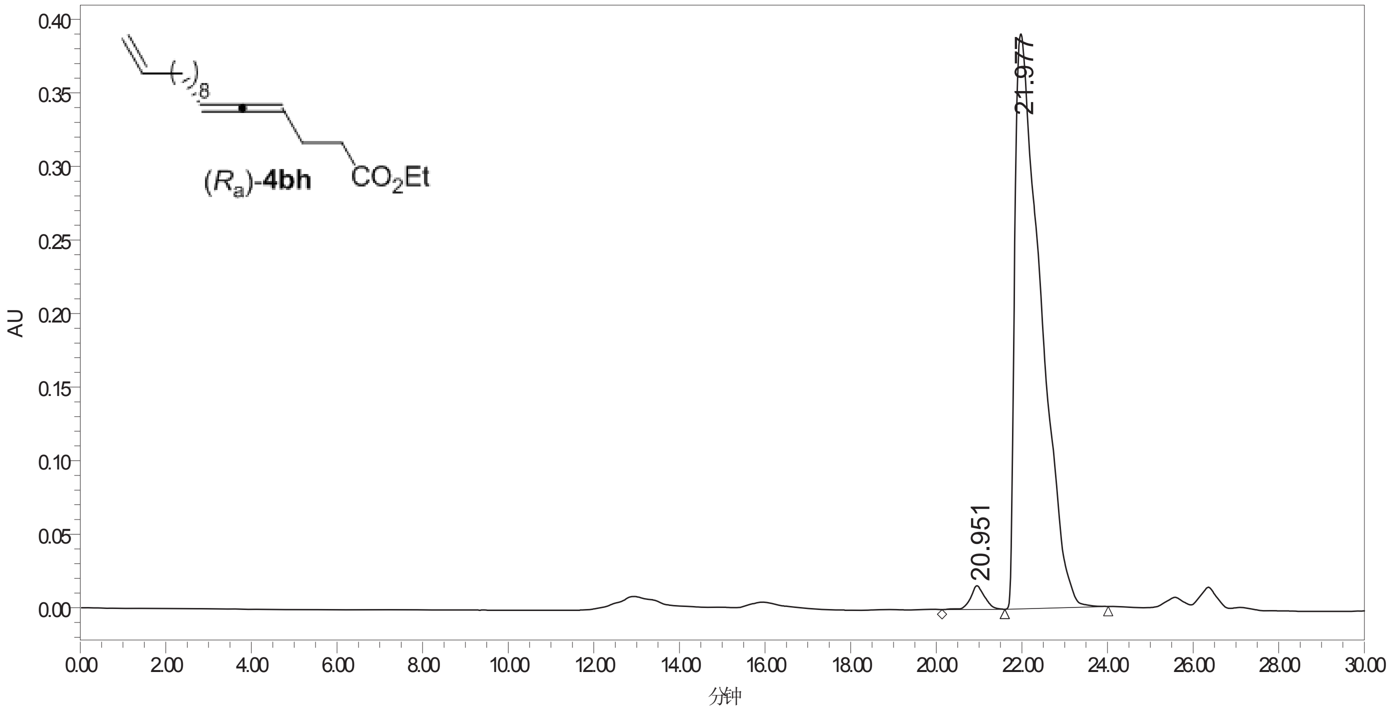

	RT (min)	Area (微伏*sec)	% Area	Height (微伏)	% Height
1	20.951	372530	2.23	16150	3.97
2	21.977	16308317	97.77	390846	96.03

Report Method: Individual Report ASC

Page: 1 (共计 1)

Printed: 2016/3/28 16:08:30 PRC

Supplementary Figure 55. HPLC spectrum for (*R*a)-4bh.

中国科学院上海有机化学研究所

Project Name: defaults for copy

Reported by User: Breeze user (Breeze)

SAMPLE INFORMATION

Sample Name:	zj-5-064-oz-100-0-0.5-214	Acquired By:	Breeze
Sample Type:	未知	Date Acquired:	2016/3/28 12:25:44 CST
Vial:	20	Acq. Method:	zgj100
Injection #:	1	Date Processed:	2016/3/28 12:56:54 CST
Injection Volume:	10.00 ul	Channel Name:	W2489 ChA
Run Time:	65.00 Minutes	Channel Desc.:	W2489 ChA 214nm
Column Type:		Sample Set Name:	

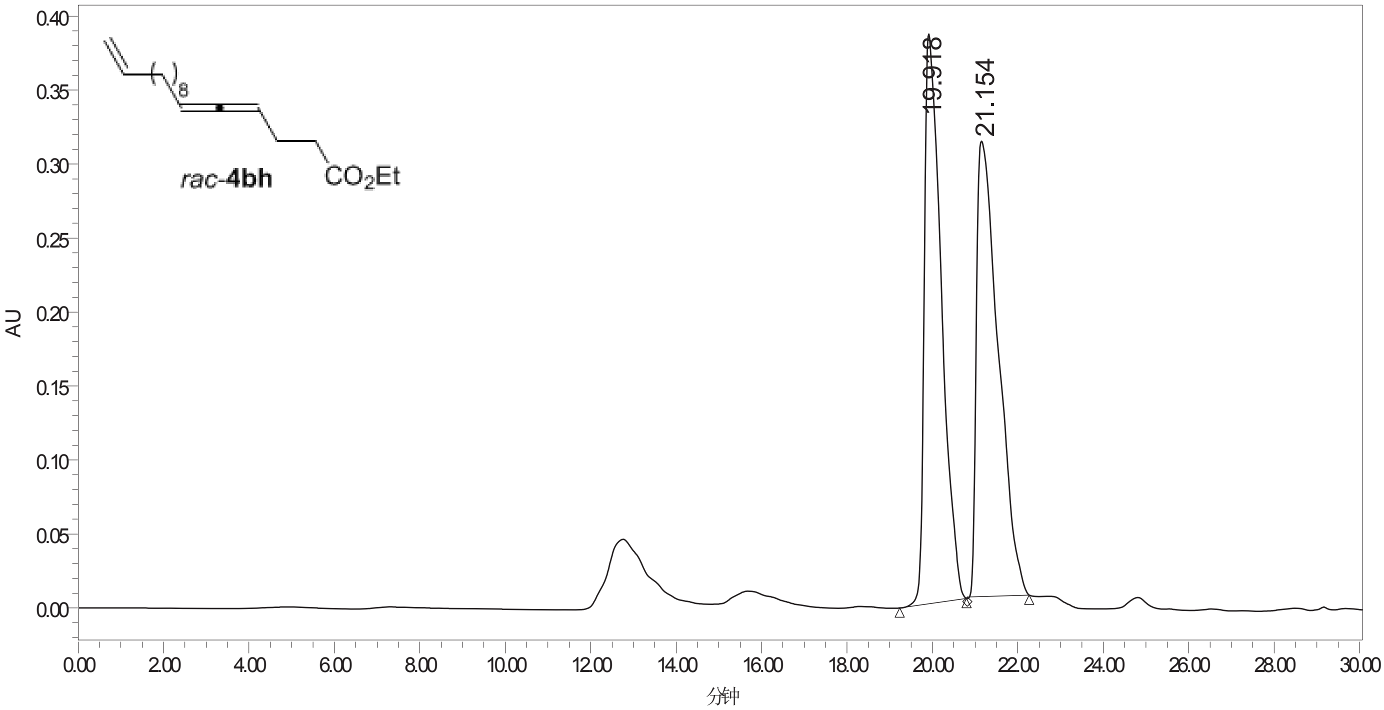

	RT (min)	Area (微伏*sec)	% Area	Height (微伏)	% Height
1	19.918	10697324	49.50	385164	55.58
2	21.154	10912720	50.50	307851	44.42

Report Method: Individual Report ASC

Page: 1 (共计 1)

Printed: 2016/3/28 16:07:46 PRC

Supplementary Figure 56. HPLC spectrum for *rac*-4bh.

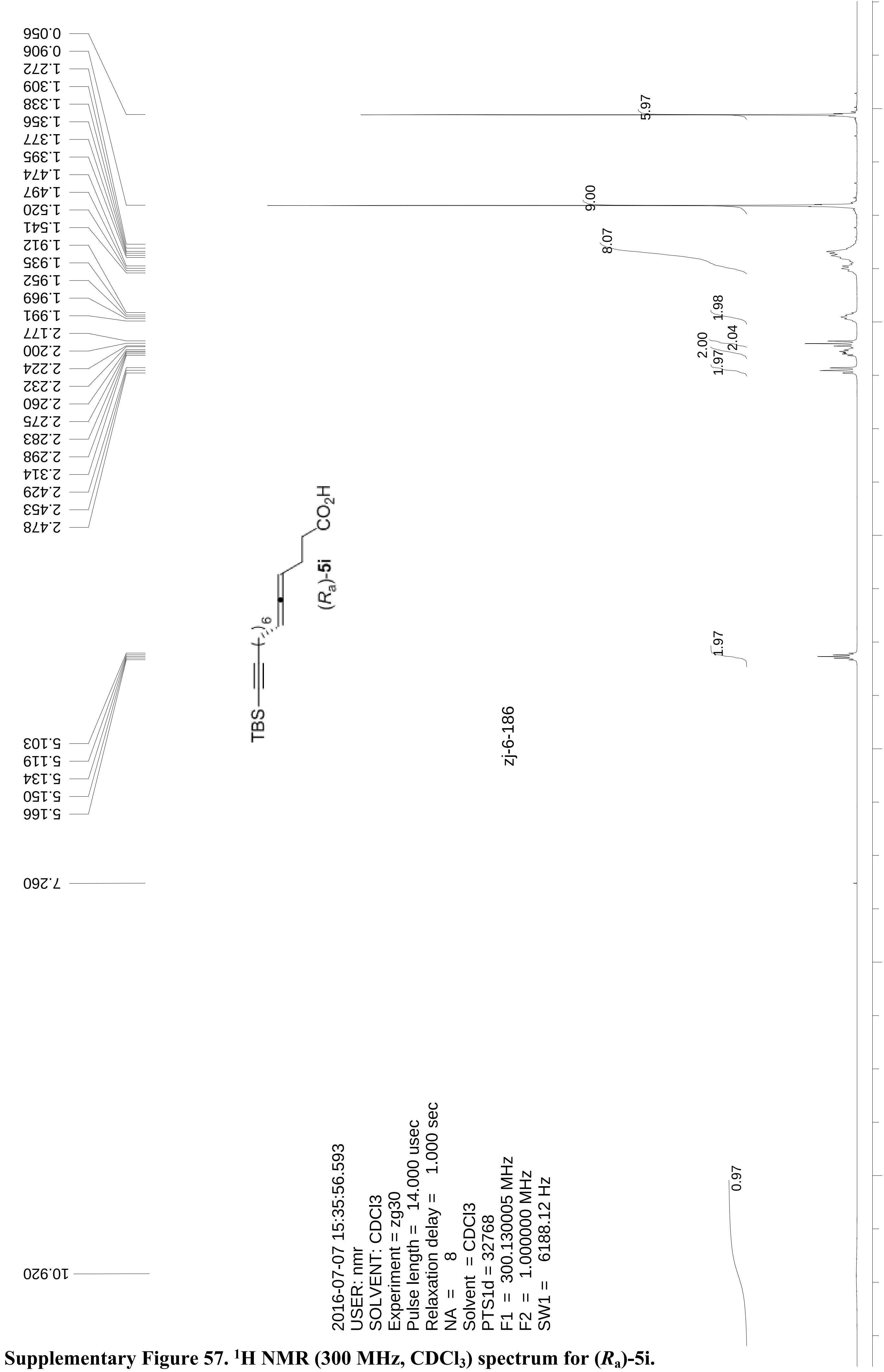

Supplementary Figure 57. ^{1}H NMR (300 MHz, $CDCl_3$) spectrum for (R_a)-5i.

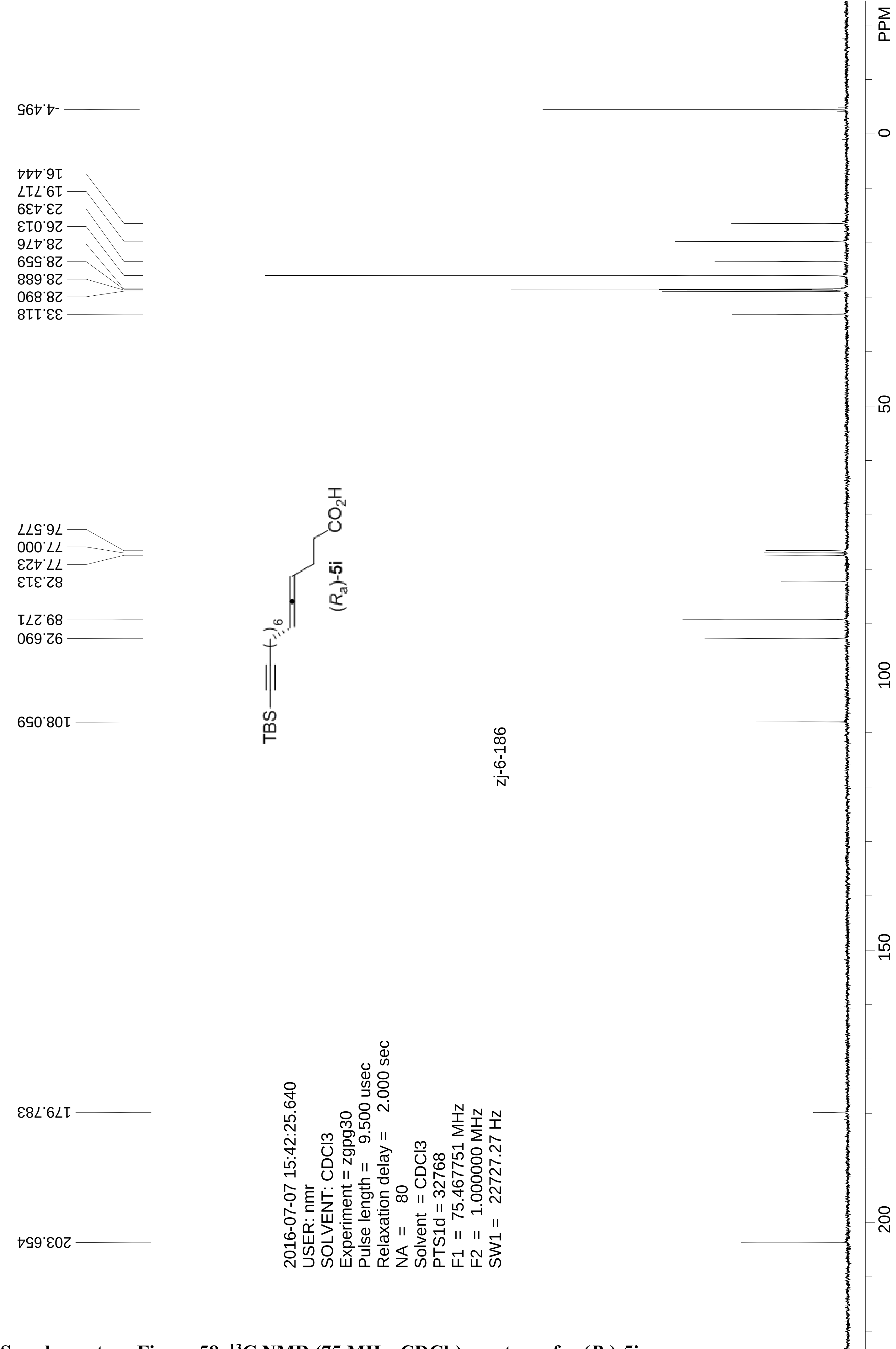

Supplementary Figure 58. ^{13}**C NMR (75 MHz,** $CDCl_3$**) spectrum for (*R*$_a$)-5i.**

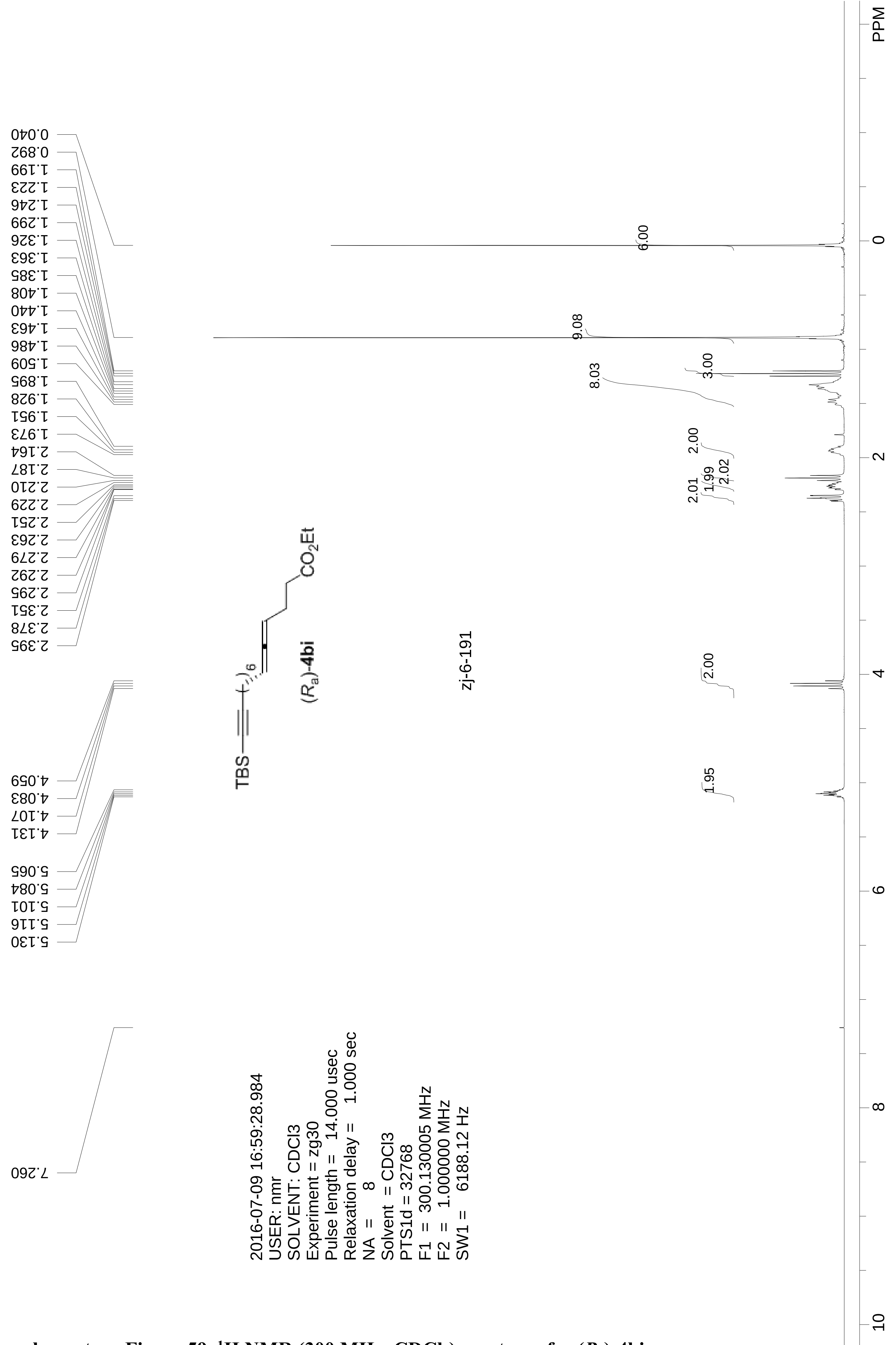

Supplementary Figure 59. ^{1}H NMR (300 MHz, $CDCl_3$) spectrum for (R_a)-4bi.

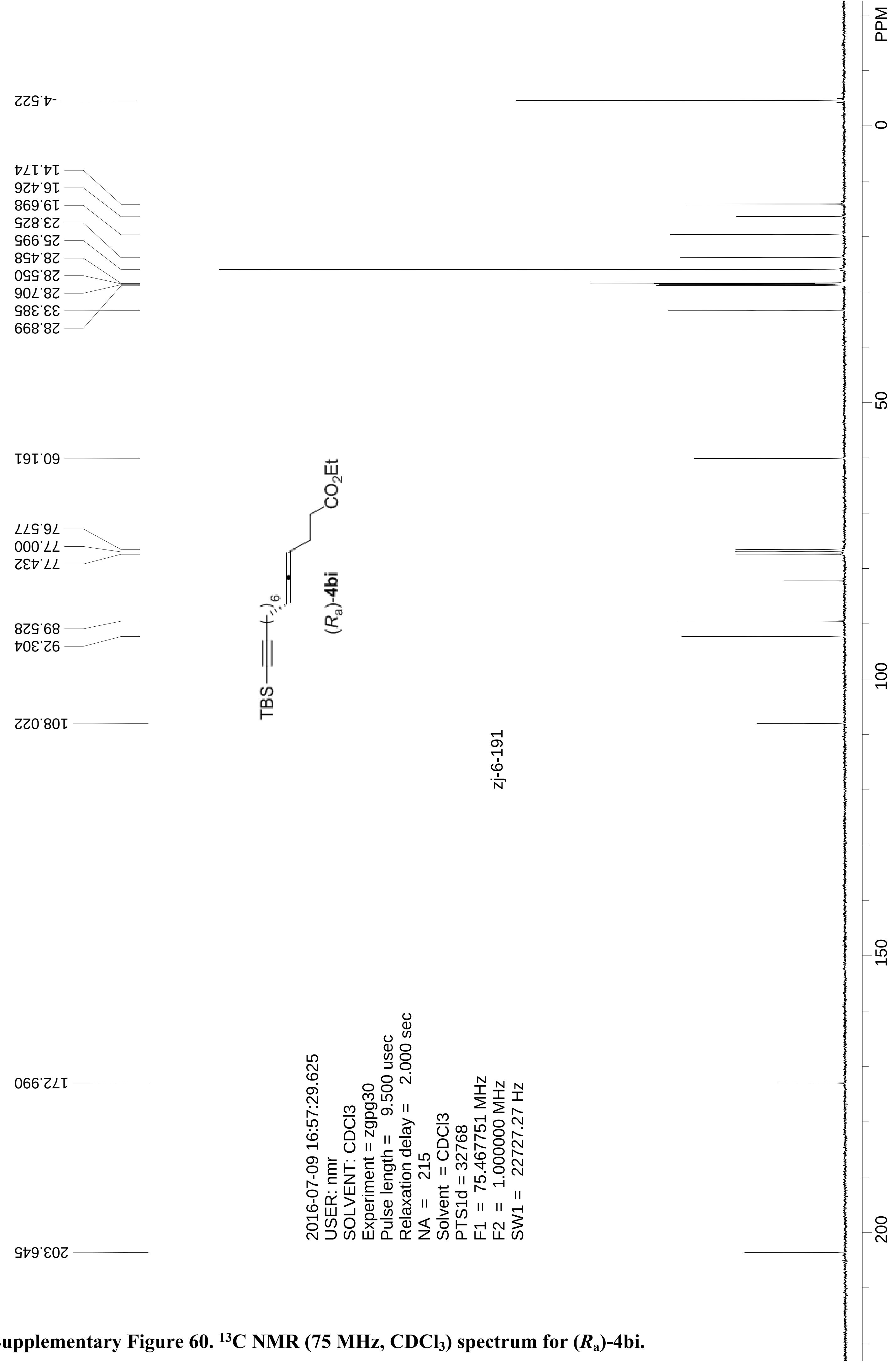

Supplementary Figure 60. ^{13}C NMR (75 MHz, $CDCl_3$) spectrum for (R_a)-4bi.

zj-6-191-oz-h-100-0-1-214

实验时间：2016-07-20,14:32:36　　报告时间：2016-07-20,16:37:47
谱图文件:D:\zhuguangjiong\zj\20160719\zj-6-191-oz-h-100-0-1-214.....org

实验内容简介：

色谱图(zj-6-191-oz-h-100-0-1-214.....org)

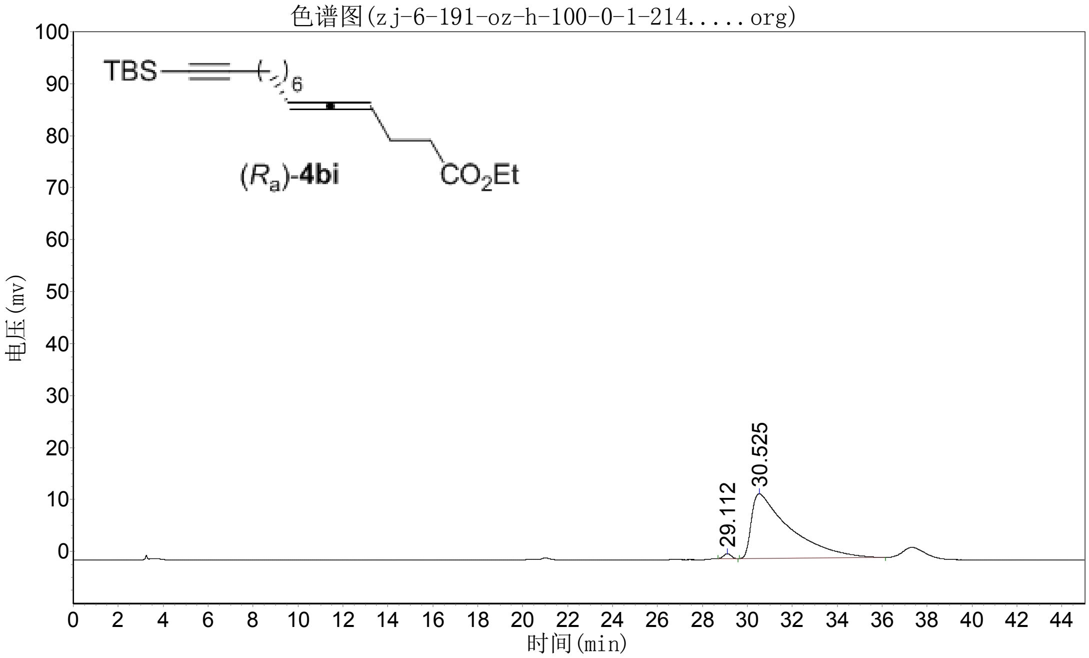

分析结果表

峰号	峰名	保留时间	峰高	峰面积	含量
1		29.112	916.083	21239.408	1.4259
2		30.525	12404.063	1468265.625	98.5741
总计			13320.146	1489505.033	100.0000

Supplementary Figure 61. HPLC spectrum for (R_a)-4bi.

zj-6-190-oz-h-100-0-1-214

实验时间：2016-07-20, 9:47:40　　　　报告时间：2016-07-20, 16:39:03
谱图文件:D:\zhuguangjiong\zj\20160719\zj-6-190-oz-h-100-0-1-214..org

实验内容简介：

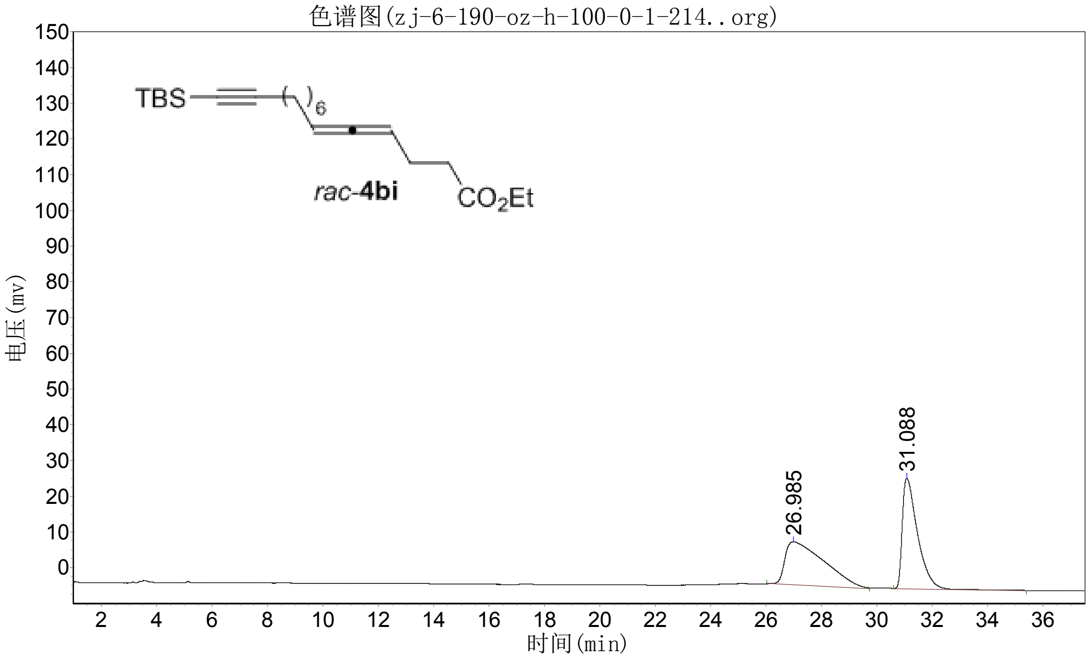

分析结果表

峰号	峰名	保留时间	峰高	峰面积	含量
1		26.985	12032.708	1188864.500	50.4579
2		31.088	30860.033	1167286.250	49.5421
总计			42892.741	2356150.750	100.0000

Supplementary Figure 62. HPLC spectrum for *rac*-4bi.

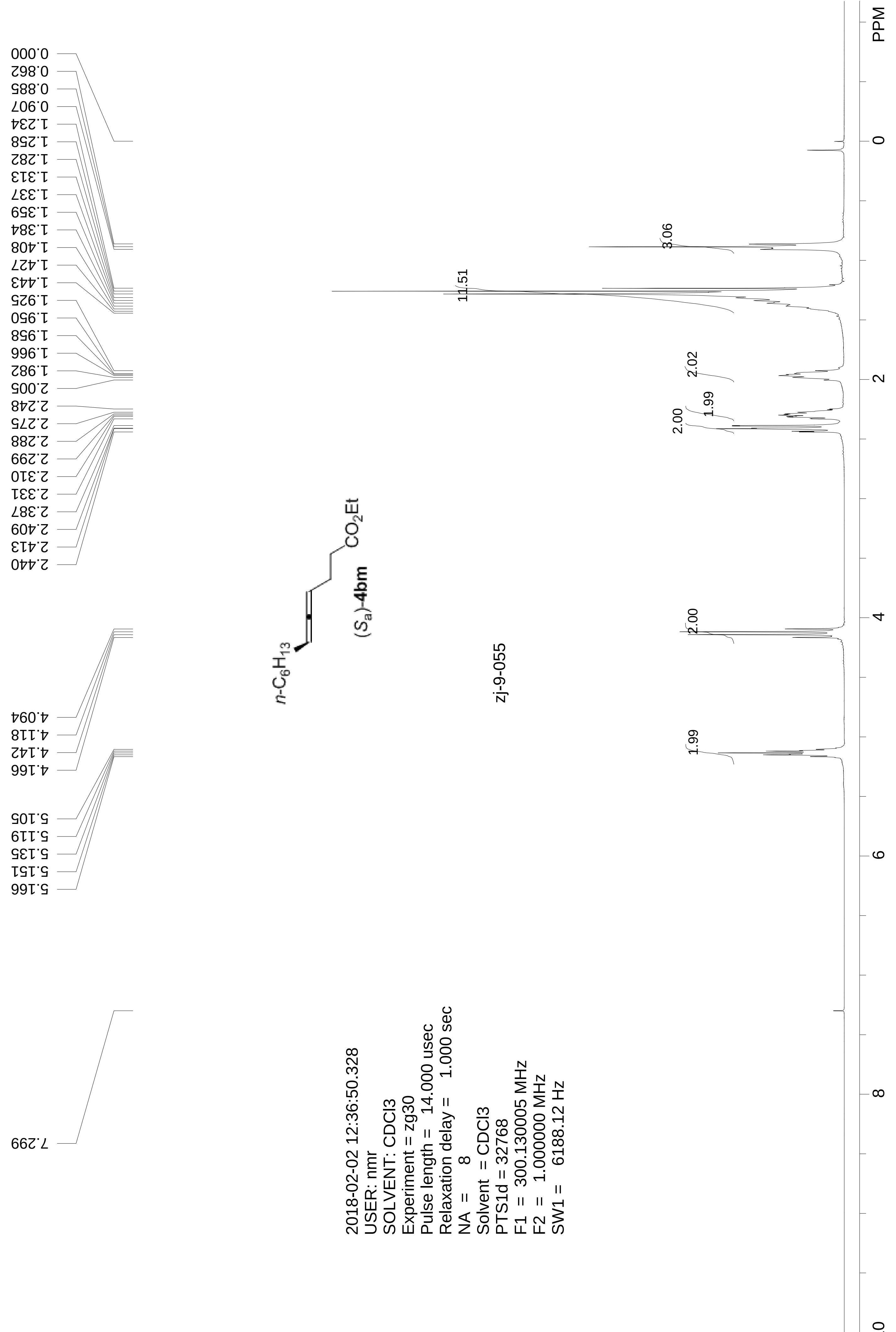

Supplementary Figure 63. ^{1}H NMR (300 MHz, $CDCl_3$) spectrum for (*S*$_a$)-4bm.

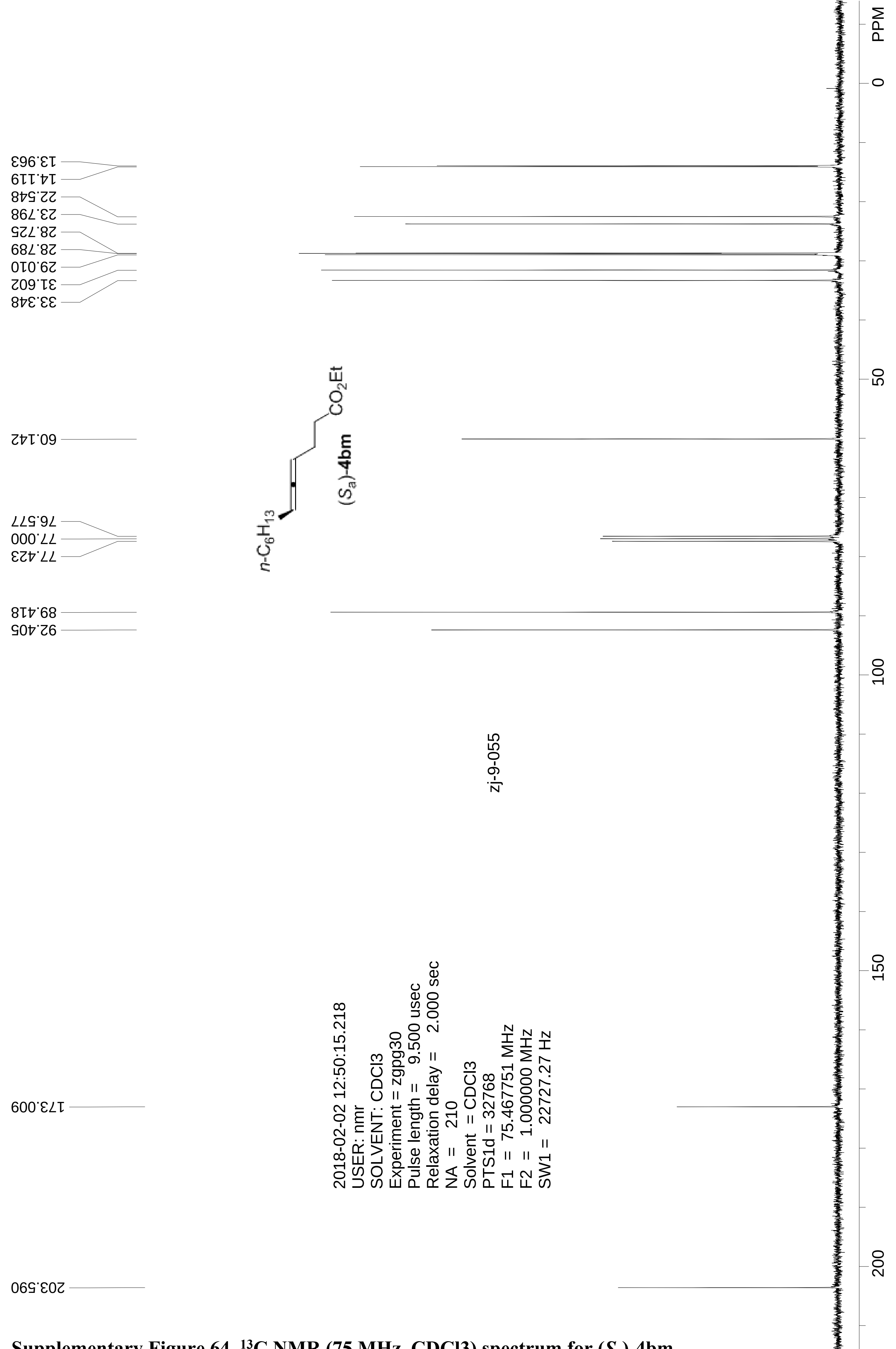

Supplementary Figure 64. ^{13}C NMR (75 MHz, CDCl3) spectrum for (*S*$_a$)-4bm.

中国科学院上海有机化学研究所

Project Name: defaults for copy

Reported by User: Breeze user (Breeze)

SAMPLE INFORMATION

Sample Name:	zj-9-055-od-h-100-0-1-214	Acquired By:	Breeze
Sample Type:	未知	Date Acquired:	2018/2/2 17:44:04 CST
Vial:	999	Acq. Method:	zgj100
Injection #:	321	Date Processed:	2018/2/2 18:34:39 CST
Injection Volume:	10.00 ul	Channel Name:	W2489 ChA
Run Time:	35.00 Minutes	Channel Desc.:	W2489 ChA 214nm
Column Type:		Sample Set Name:	

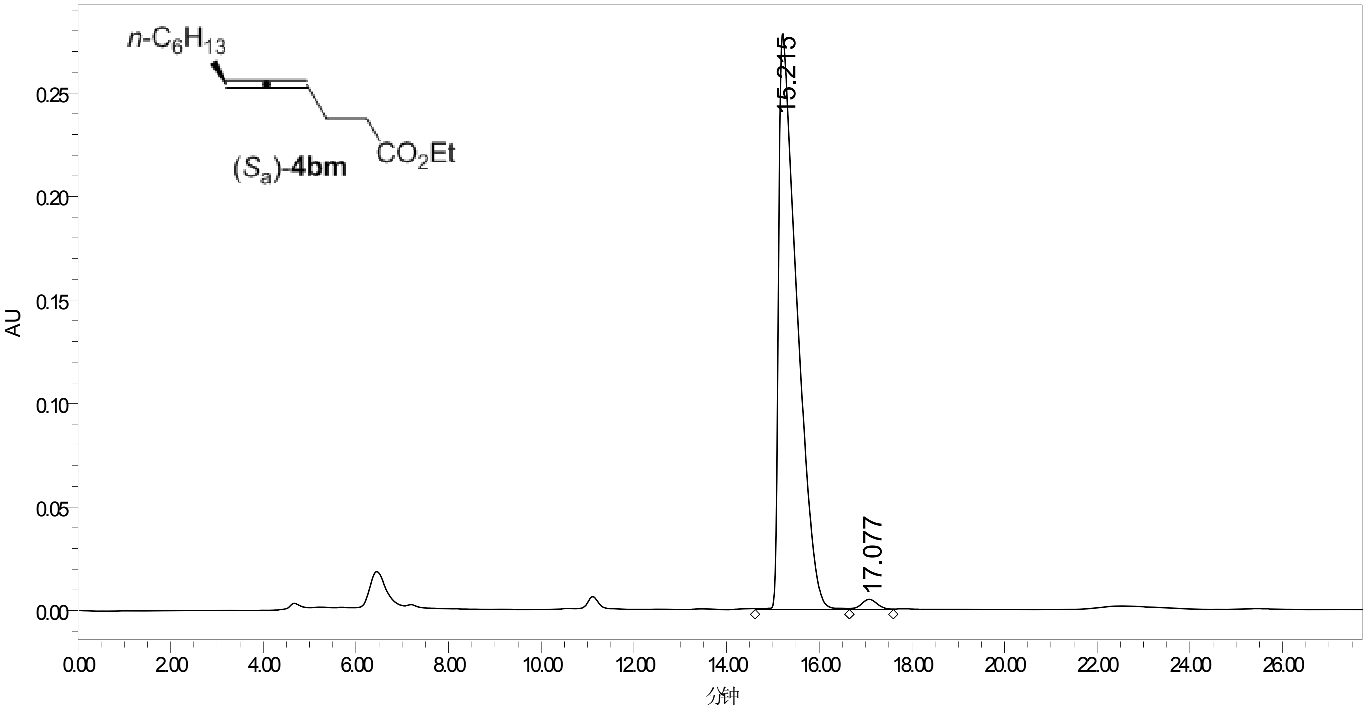

	RT (min)	Area (微伏*sec)	% Area	Height (微伏)	% Height
1	15.215	7641392	98.45	278172	98.26
2	17.077	120136	1.55	4919	1.74

Report Method: Individual Report ASC

Printed: 2018/2/2 18:35:25 PRC

Page: 1 (共计 1)

Supplementary Figure 65. HPLC spectrum for (S_a)-4bm.

中国科学院上海有机化学研究所

Project Name: defaults for copy

Reported by User: Breeze user (Breeze)

SAMPLE INFORMATION

Sample Name:	zj-9-056-od-h-100-0-1-214	Acquired By:	Breeze
Sample Type:	未知	Date Acquired:	2018/2/2 18:12:00 CST
Vial:	999	Acq. Method:	zgj100
Injection #:	322	Date Processed:	2018/2/2 18:34:23 CST
Injection Volume:	10.00 ul	Channel Name:	W2489 ChA
Run Time:	35.00 Minutes	Channel Desc.:	W2489 ChA 214nm
Column Type:		Sample Set Name:	

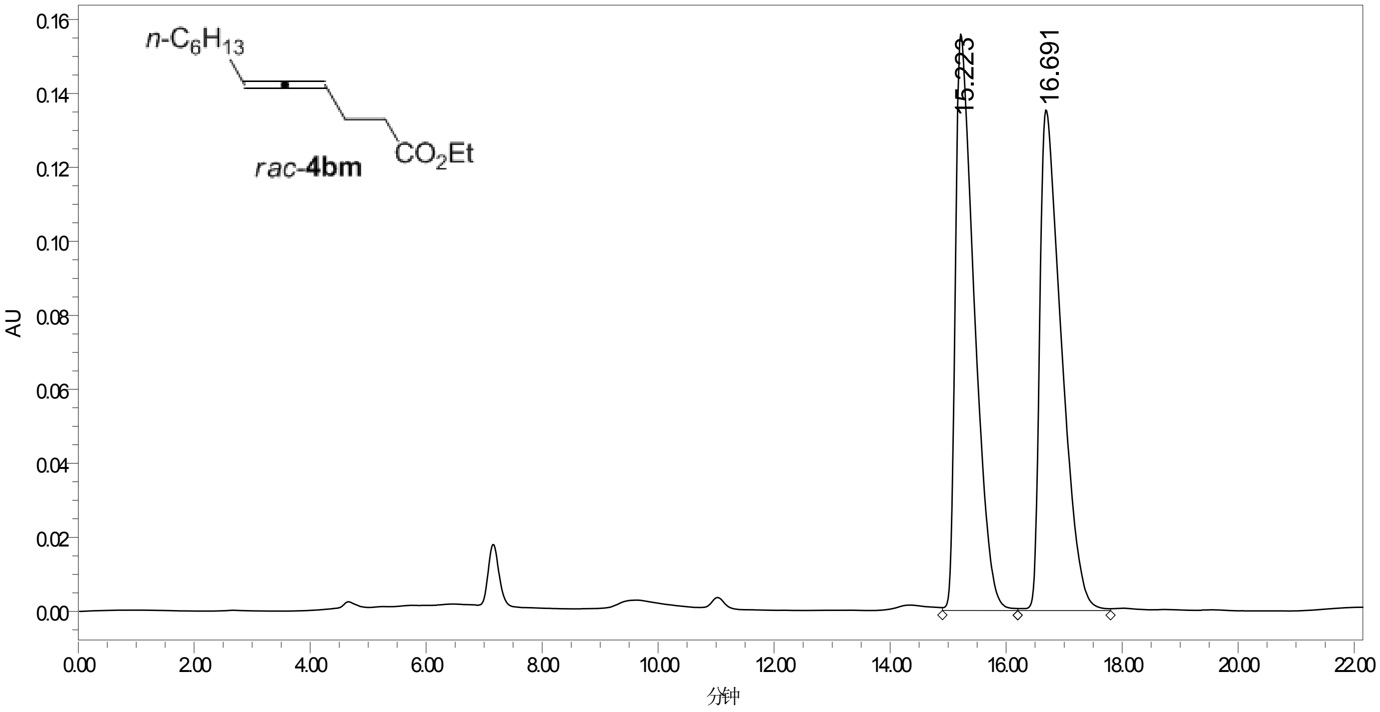

	RT (min)	Area (礦sec)	% Area	Height (礦)	% Height
1	15.223	3551394	50.20	156009	53.51
2	16.691	3523692	49.80	135550	46.49

Report Method: Individual Report ASC

Printed: 2018/2/2 18:34:50 PRC

Page: 1 (共计 1)

Supplementary Figure 66. HPLC spectrum for *rac*-4bm.

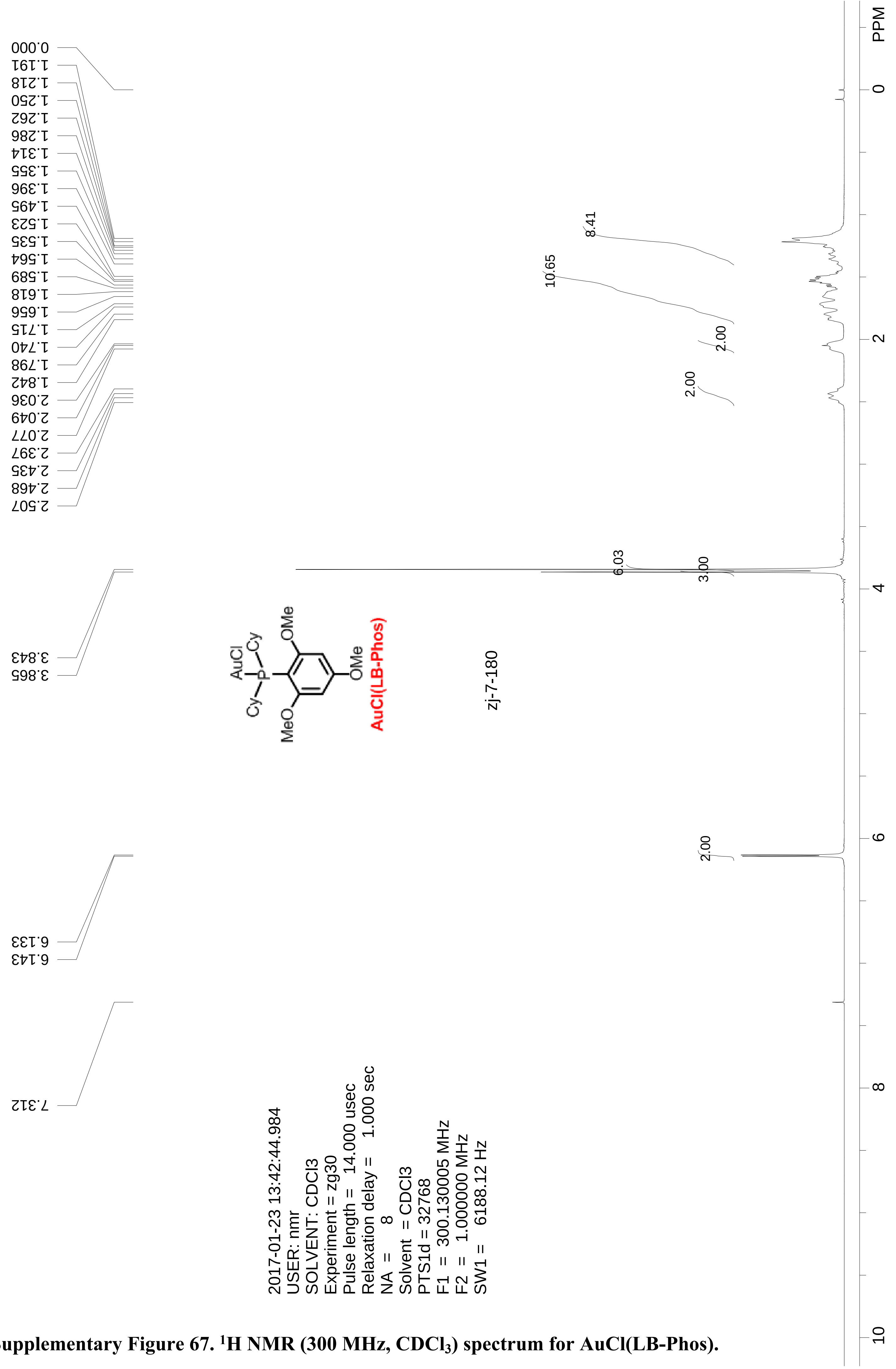

Supplementary Figure 67. **^{1}H NMR (300 MHz, $CDCl_3$) spectrum for AuCl(LB-Phos).**

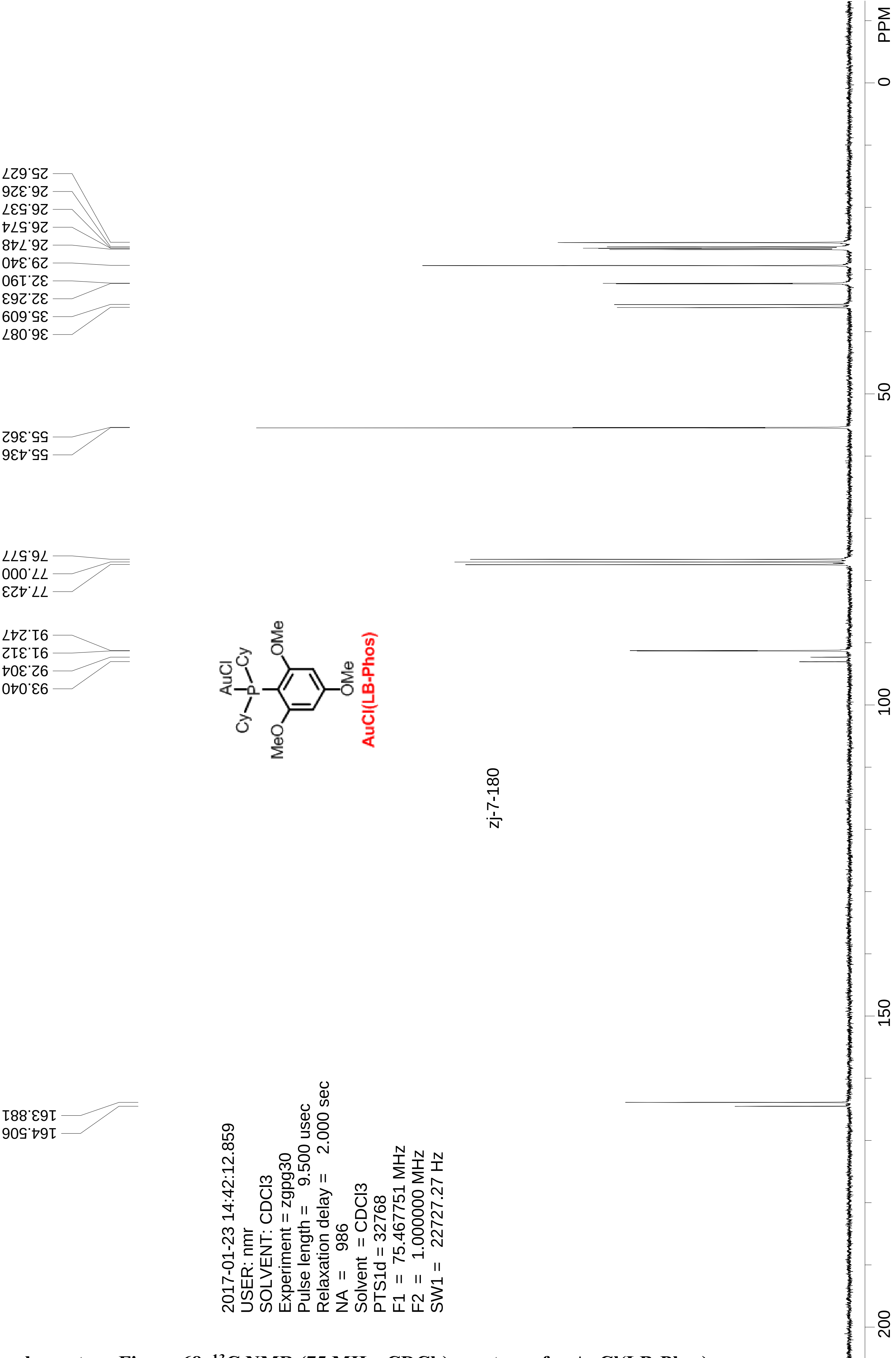

Supplementary Figure 68. ^{13}**C NMR (75 MHz, CDCl$_3$) spectrum for AuCl(LB-Phos).**

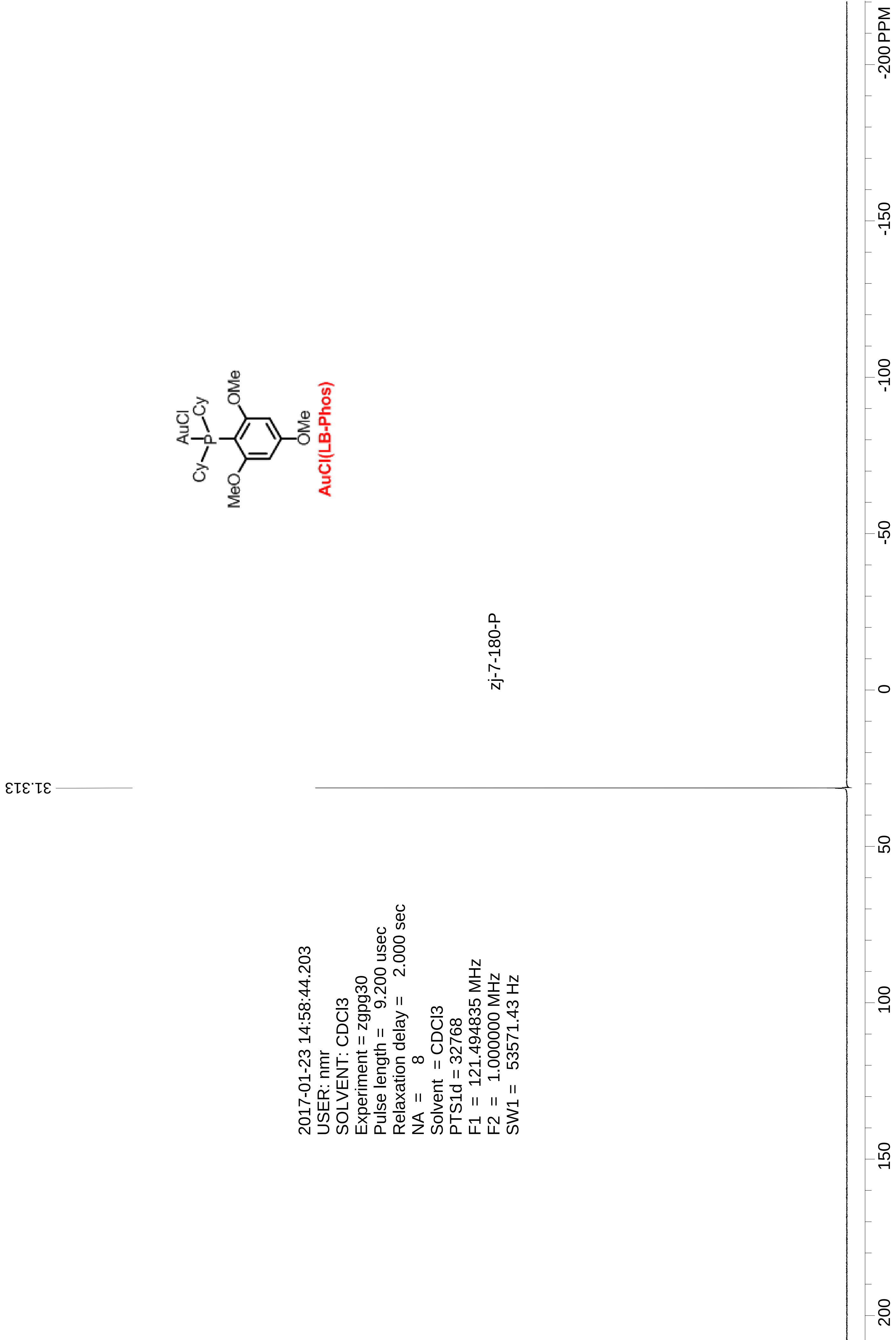

Supplementary Figure 69. ^{31}P NMR (121.5 MHz, $CDCl_3$) spectrum for AuCl(LB-Phos).

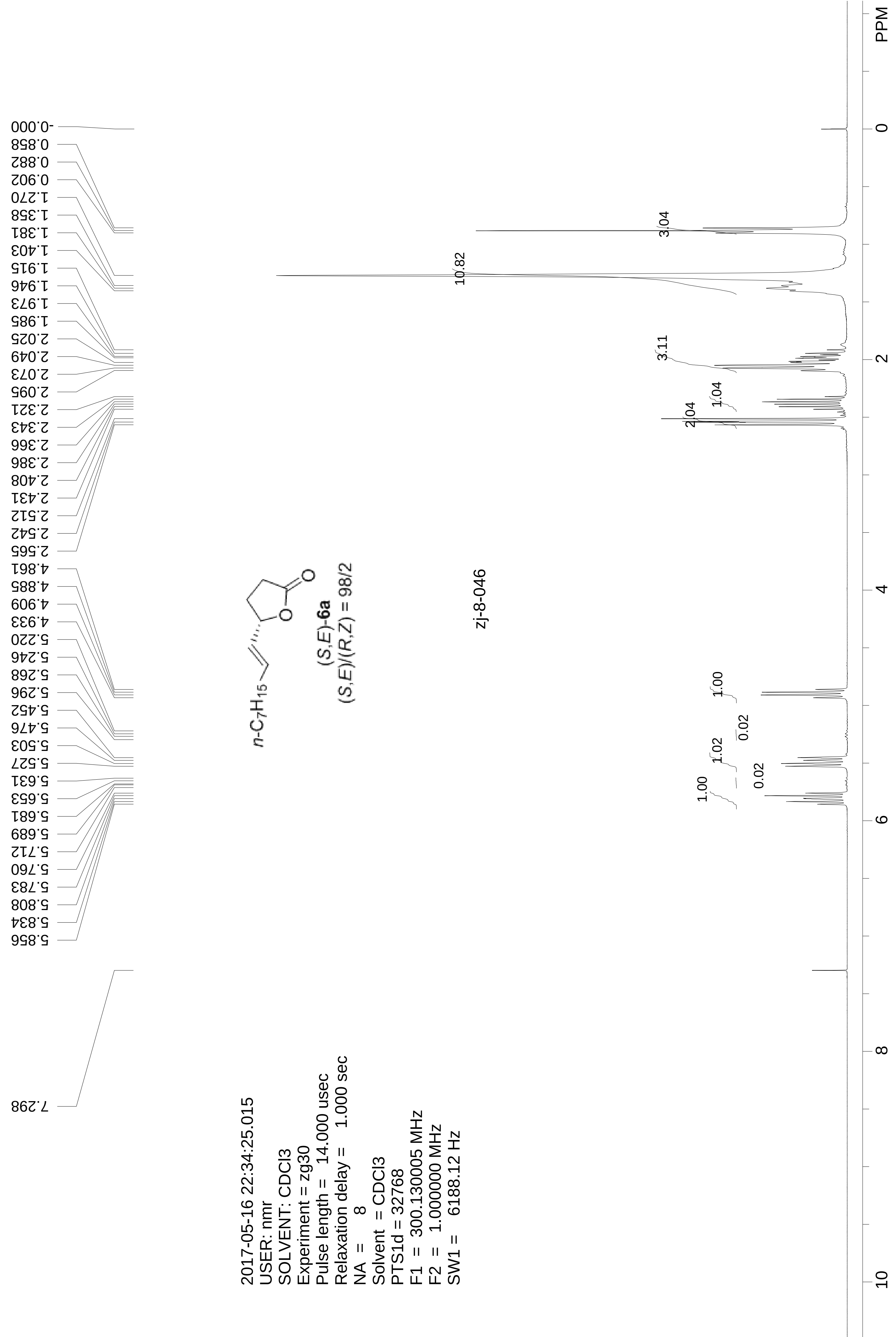

Supplementary Figure 70. ^{1}**H NMR (300 MHz, CDCl$_3$) spectrum for (*S*,*E*)-6a.**

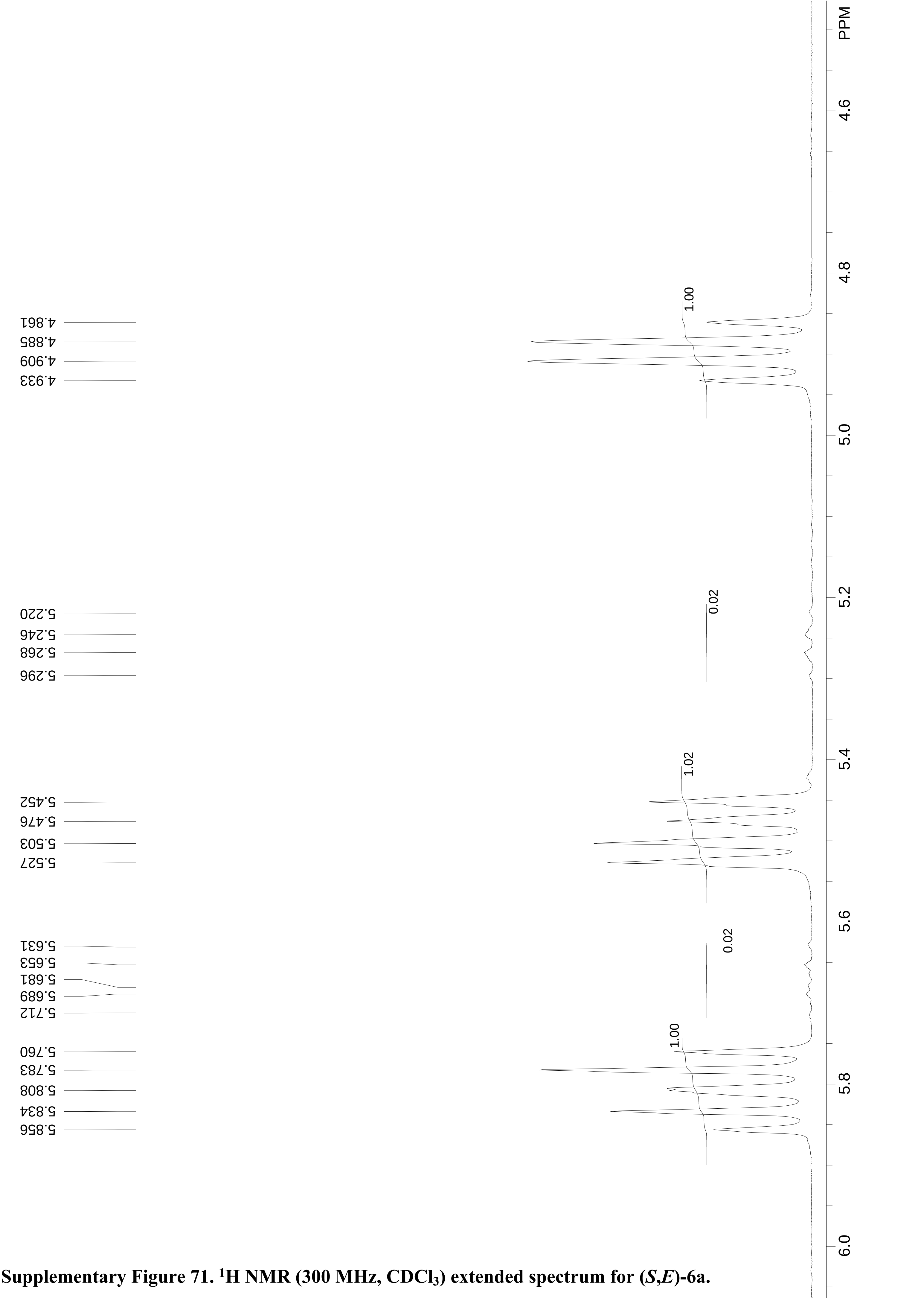

Supplementary Figure 71. ^{1}H NMR (300 MHz, $CDCl_3$) extended spectrum for (*S*,*E*)-6a.

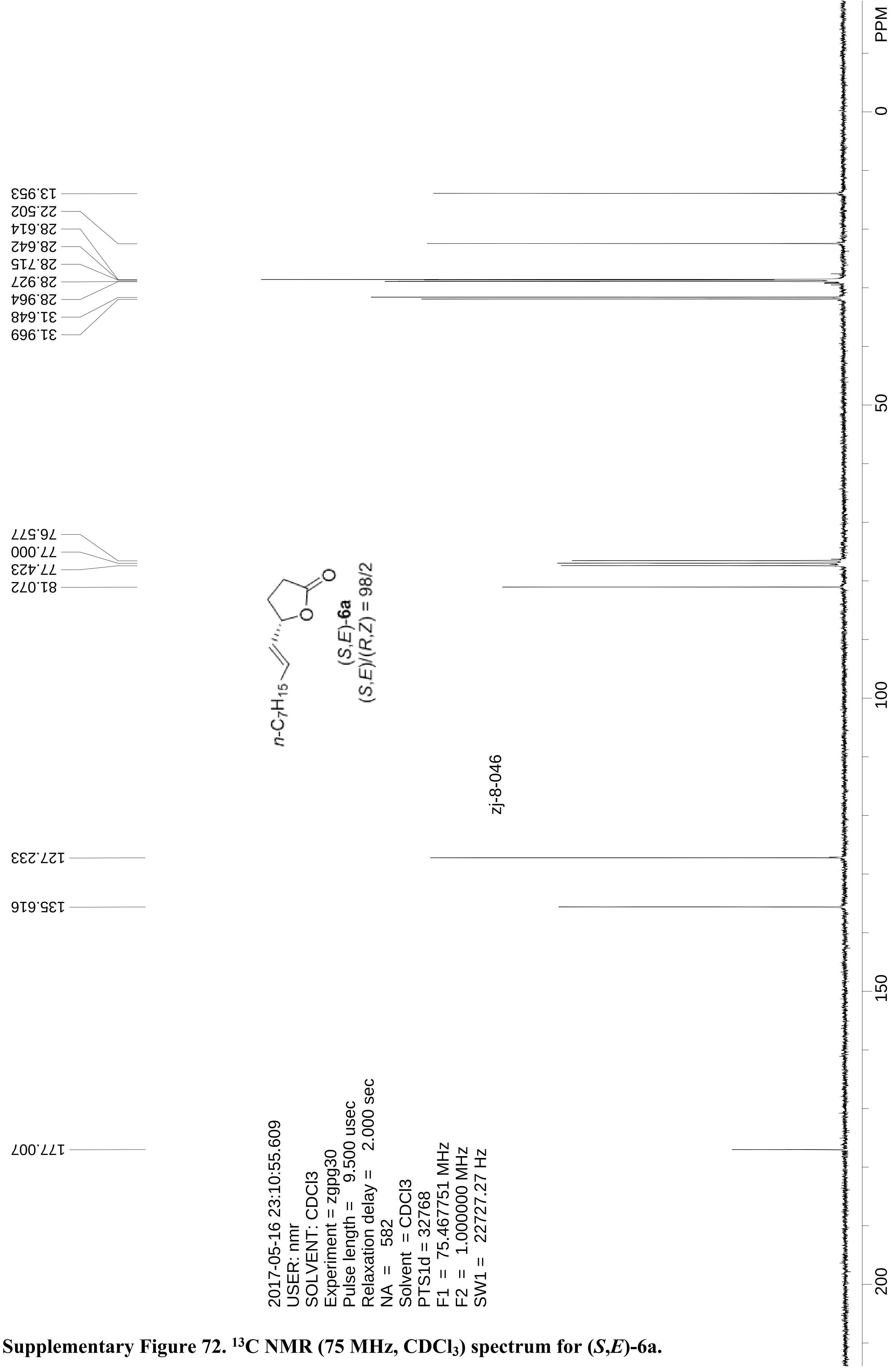

Supplementary Figure 72. ^{13}C NMR (75 MHz, $CDCl_3$) spectrum for (*S*,*E*)-6a.

zj-8-046-oj-h-200-1-1-214

实验时间：2017-5-24,16:53:49　　报告时间：2017-5-24,17:33:30
谱图文件:D:\zhuguangjiong\zj\20170524\zj-8-046-oj-h-200-1-1-214..org

实验内容简介：

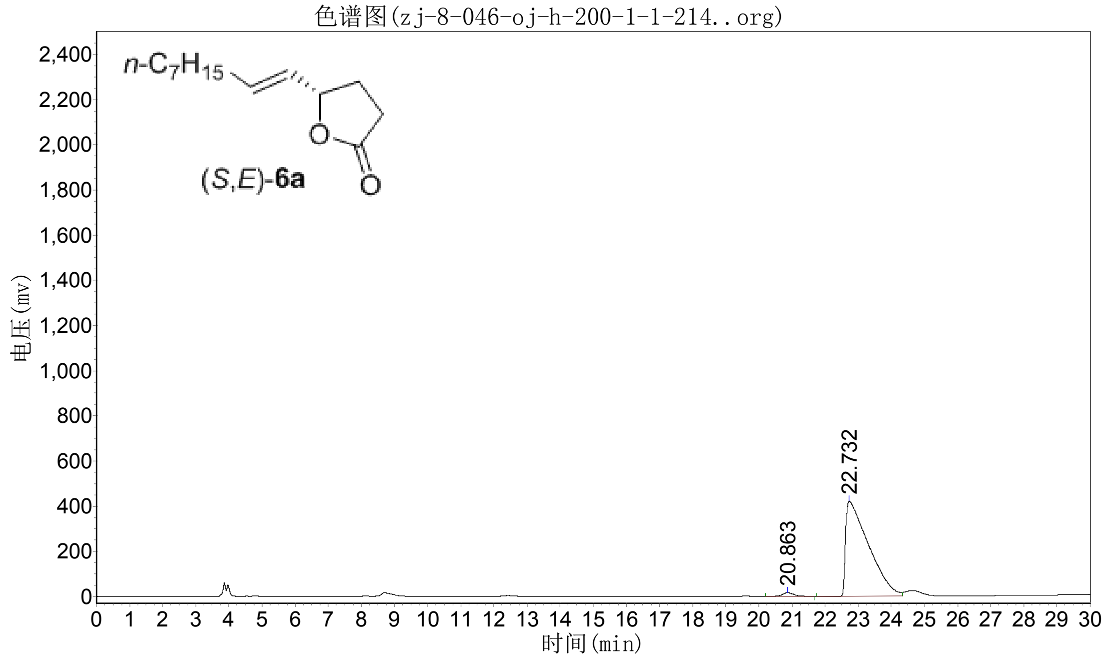

分析结果表

峰号	峰名	保留时间	峰高	峰面积	含量
1		20.863	16647.076	395363.438	1.9169
2		22.732	420144.281	20229394.000	98.0831
总计			436791.357	20624757.438	100.0000

Supplementary Figure 73. HPLC spectrum for (*S*,*E*)-6a.

zj-8-029-oj-h-200-1-1-214

实验时间：2017-5-24，15:52:47　　　　　　　　　　　　　　　　报告时间：2017-5-24，17:31:23
谱图文件:D:\zhuguangjiong\zj\20170524\zj-8-029-oj-h-200-1-1-214..org

实验内容简介：

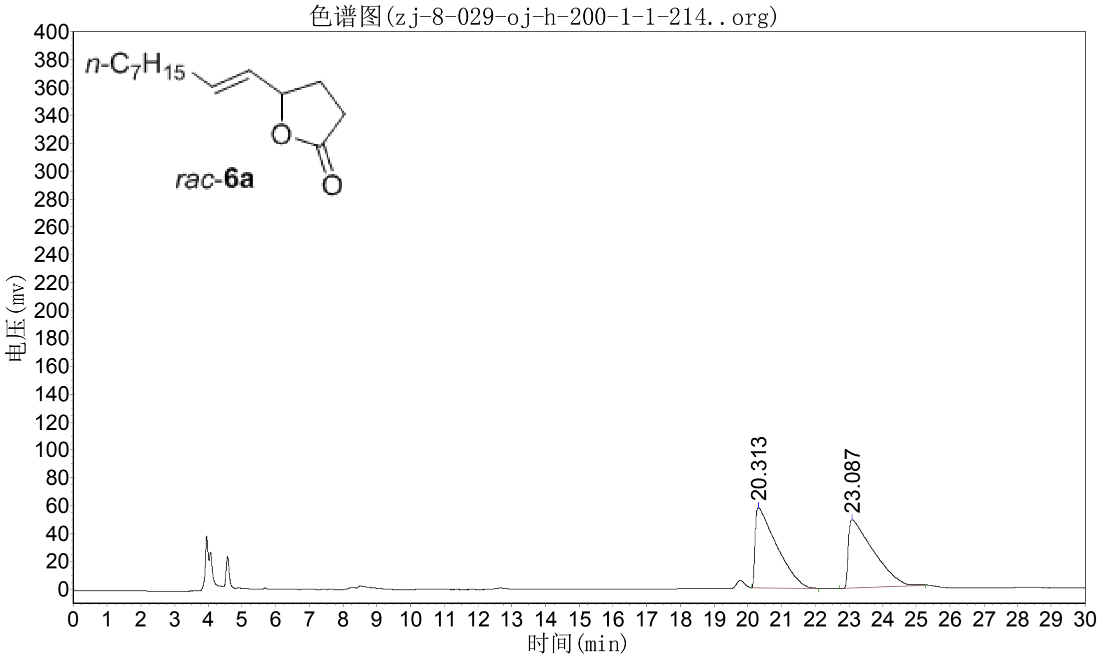

分析结果表

峰号	峰名	保留时间	峰高	峰面积	含量
1		20.313	57729.738	2545566.250	49.9868
2		23.087	49412.555	2546912.000	50.0132
总计			107142.293	5092478.250	100.0000

Supplementary Figure 74. HPLC spectrum for *rac*-6a.

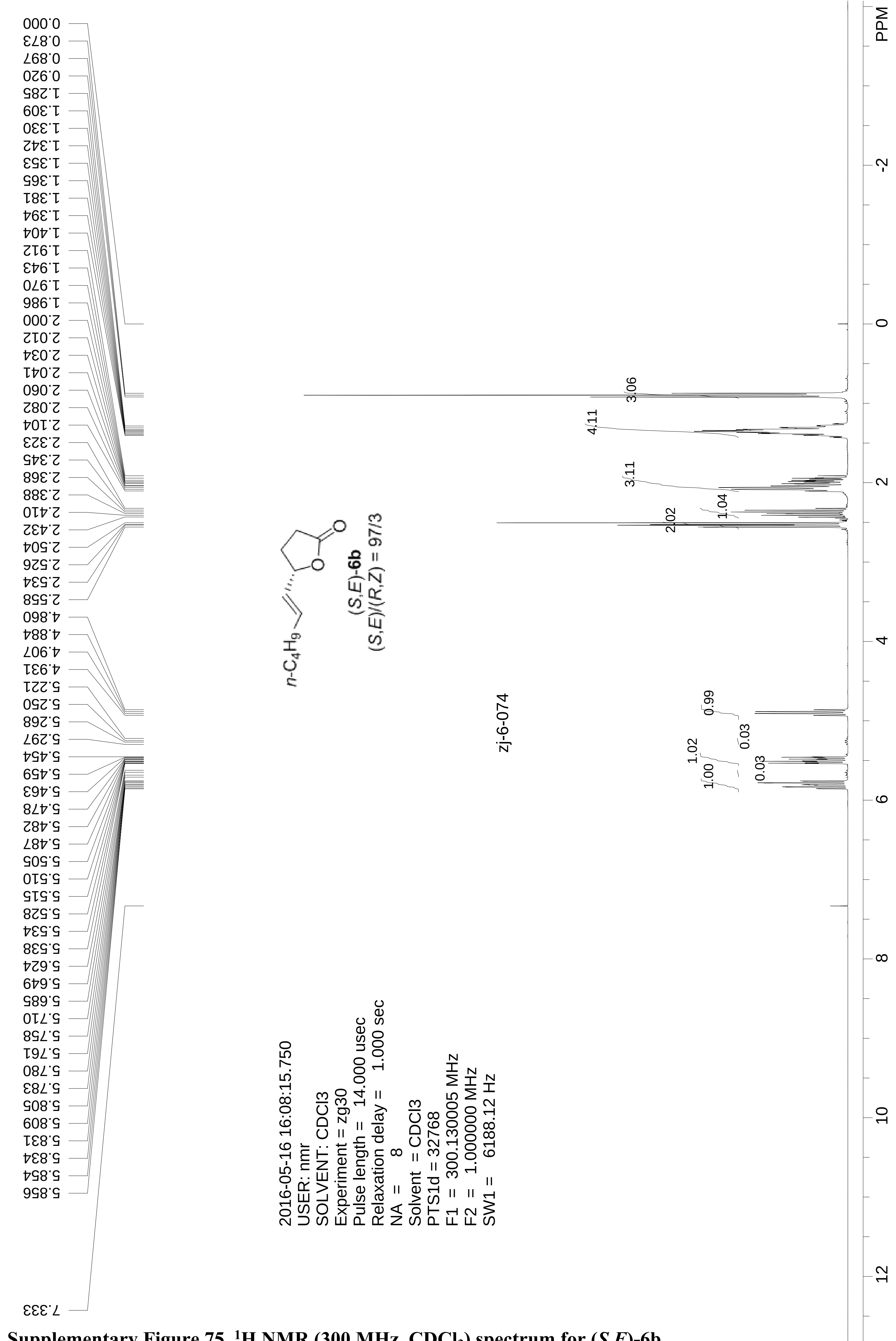

Supplementary Figure 75. ^{1}H NMR (300 MHz, $CDCl_3$) spectrum for (*S*,*E*)-6b.

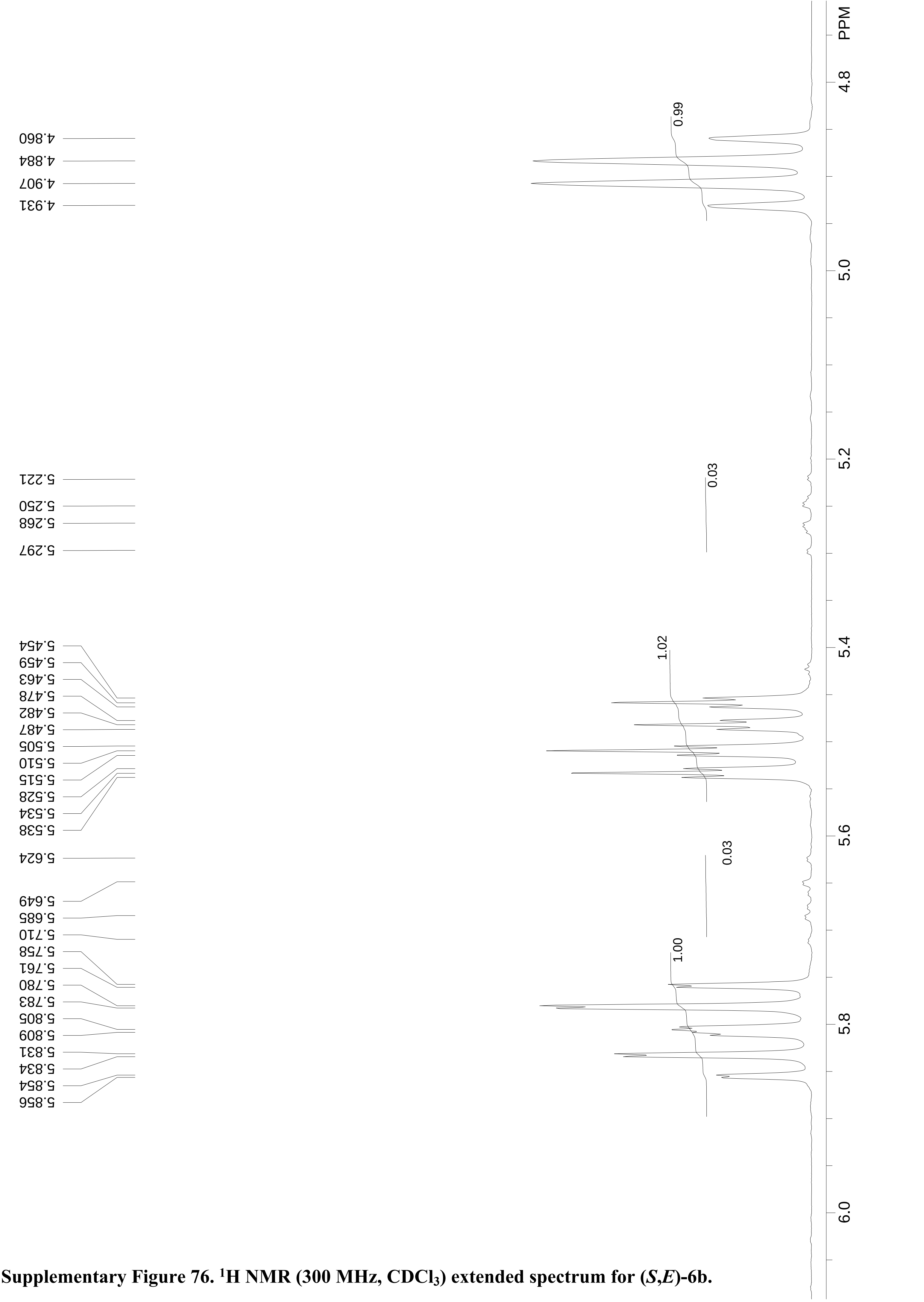

Supplementary Figure 76. ^{1}H NMR (300 MHz, $CDCl_3$) extended spectrum for (*S*,*E*)-6b.

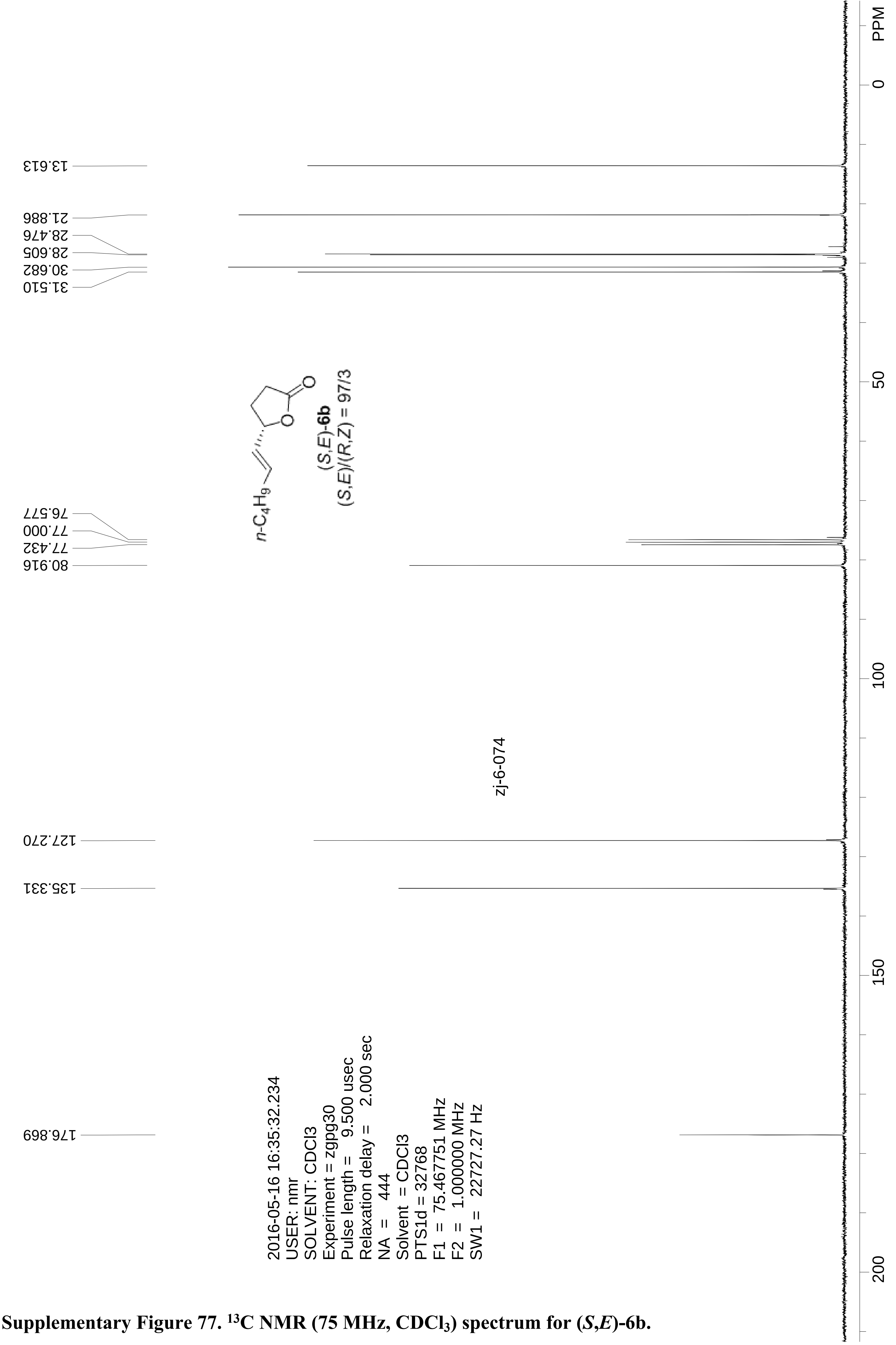

Supplementary Figure 77. ^{13}C NMR (75 MHz, $CDCl_3$) spectrum for (*S*,*E*)-6b.

zj-6-074-oj-h-200-1-1-214

实验时间：2016-05-31,12:17:54　　　　　　　　　　报告时间：2016-05-31,17:14:56
谱图文件:D:\zhuguangjiong\zj\20160531\zj-6-074-oj-h-200-1-1-214.org

实验内容简介：

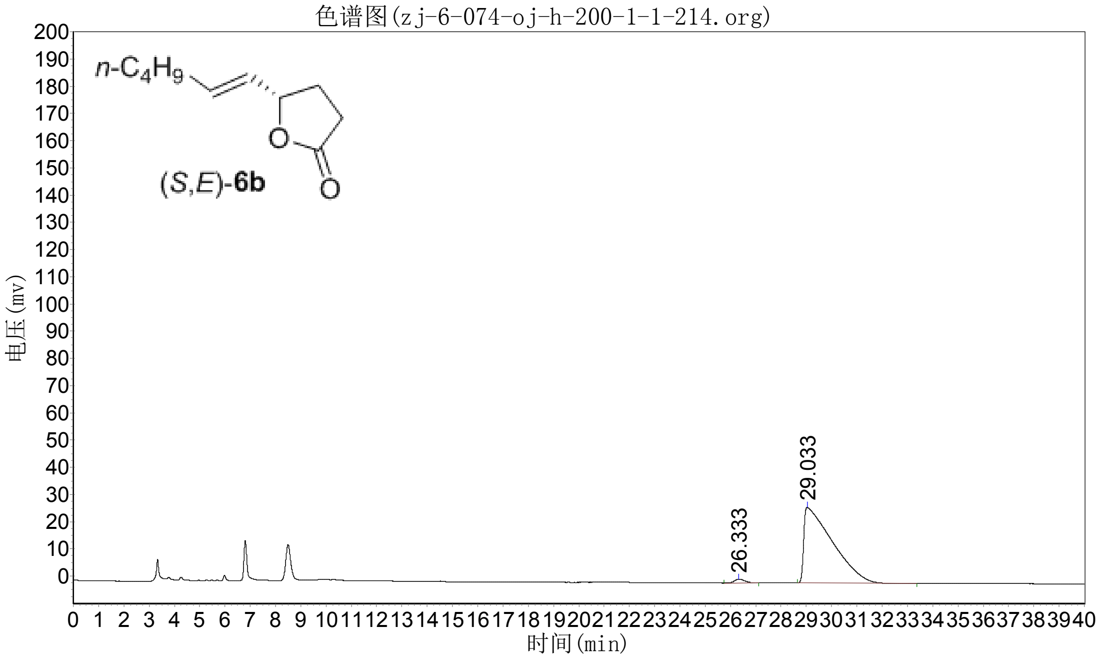

分析结果表

峰号	峰名	保留时间	峰高	峰面积	含量
1		26.333	1418.779	39042.988	1.7508
2		29.033	27742.568	2190942.750	98.2492
总计			29161.348	2229985.738	100.0000

Supplementary Figure 78. HPLC spectrum for (*S*,*E*)-6b.

zj-6-056-oj-h-200-1-1-214

实验时间：2016-05-31,14:08:40　　报告时间：2016-05-31,17:13:13
谱图文件:D:\zhuguangjiong\zj\20160531\zj-6-056-oj-h-200-1-1-214..org

实验内容简介：

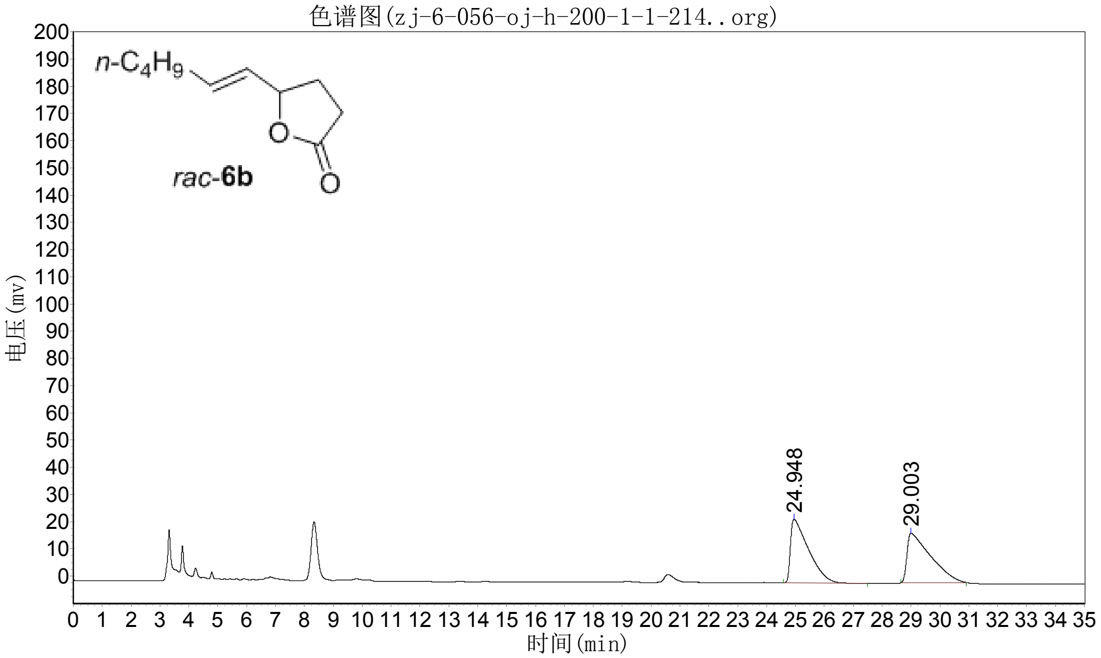

分析结果表

峰号	峰名	保留时间	峰高	峰面积	含量
1		24.948	23389.479	1017822.750	49.7277
2		29.003	18196.986	1028969.125	50.2723
总计			41586.465	2046791.875	100.0000

Supplementary Figure 79. HPLC spectrum for *rac*-6b.

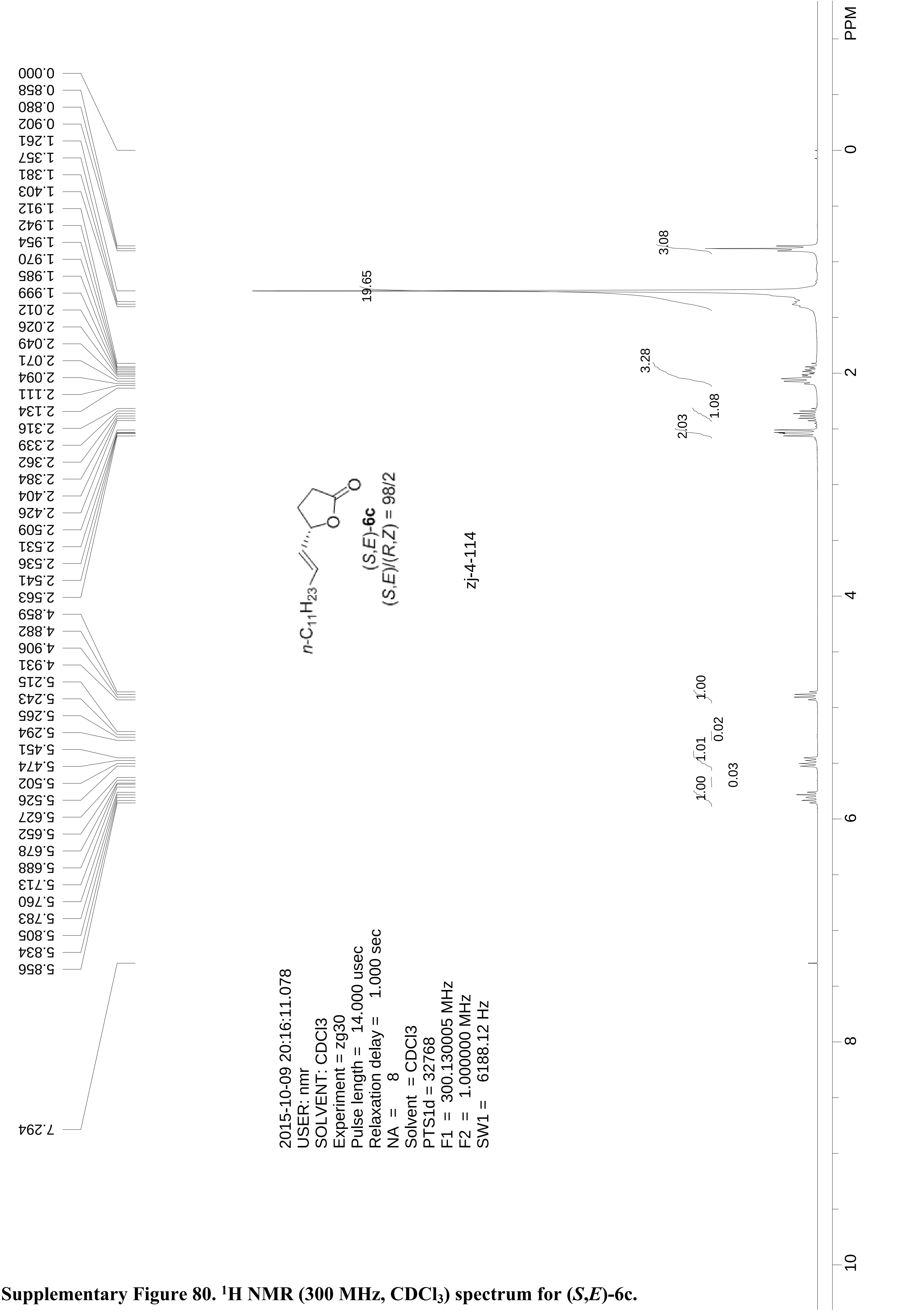

Supplementary Figure 80. ^{1}H NMR (300 MHz, $CDCl_3$) spectrum for (*S*,*E*)-6c.

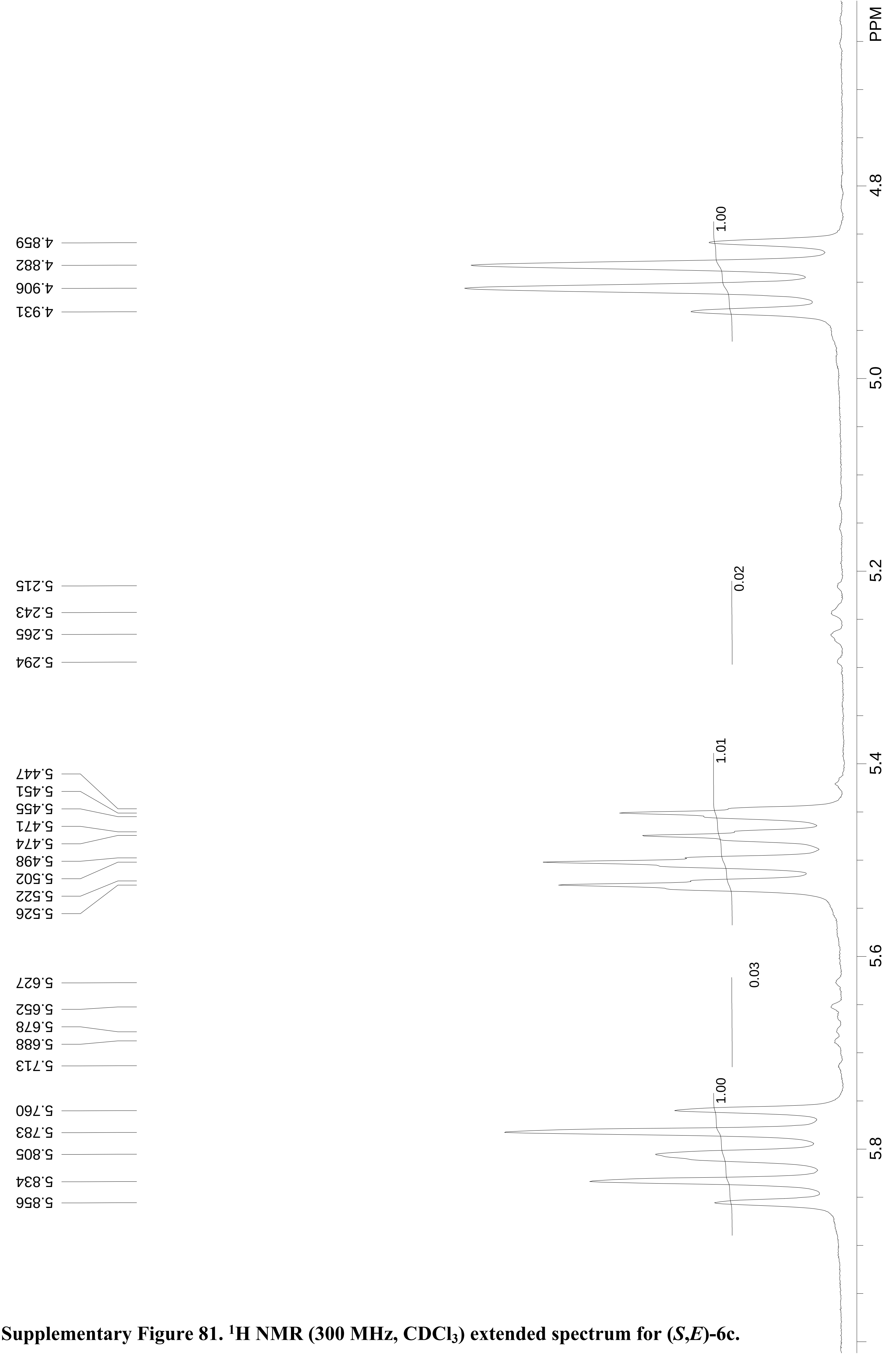

Supplementary Figure 81. ^{1}H NMR (300 MHz, $CDCl_3$) extended spectrum for (*S*,*E*)-6c.

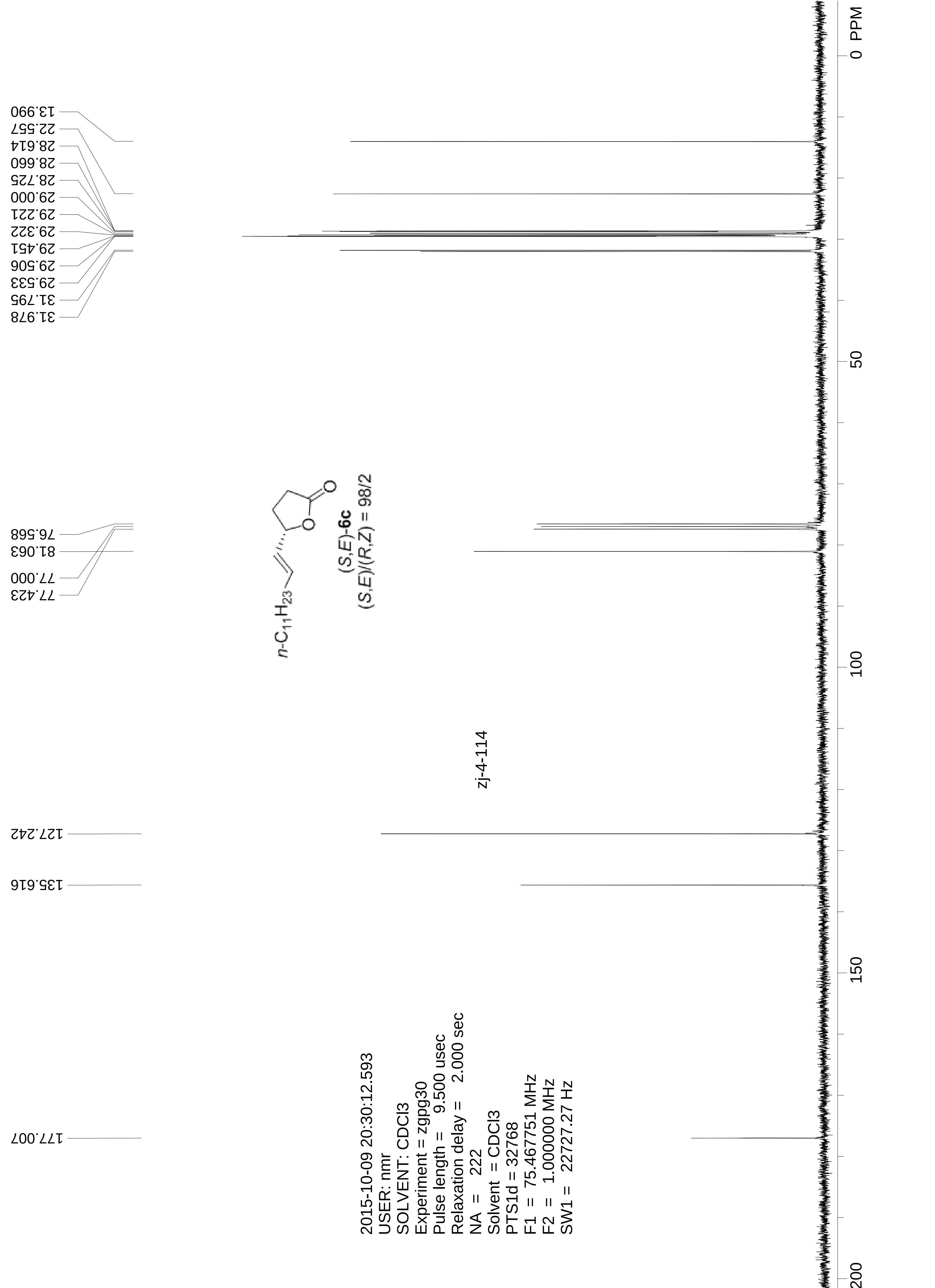

Supplementary Figure 82. ^{13}C NMR (75 MHz, $CDCl_3$) spectrum for (*S*,*E*)-6c.

zj-4-114-as-h-95-5-1-214

实验时间：2015-10-12,14:36:42　　　　　　　　　　　报告时间：2015-10-13,17:39:52
谱图文件:D:\zhuguangjiong\zj\20151010\zj-4-114-as-h-95-5-1-214..org

实验内容简介：

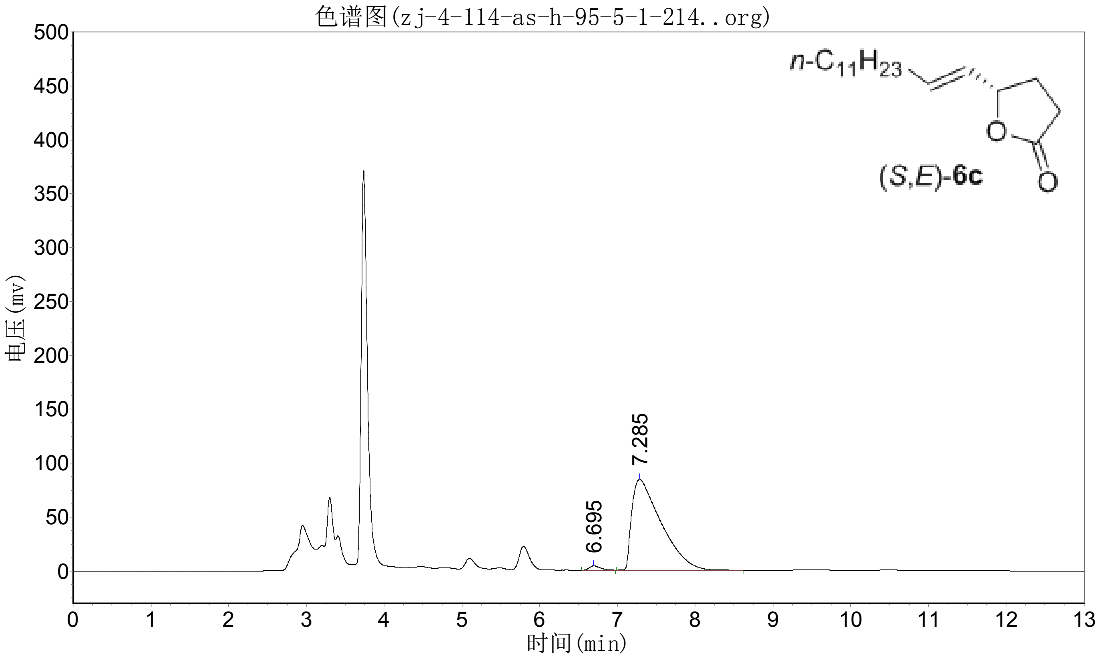

分析结果表

峰号	峰名	保留时间	峰高	峰面积	含量
1		6.695	4003.269	40812.492	1.8042
2		7.285	84500.078	2221214.250	98.1958
总计			88503.347	2262026.742	100.0000

Supplementary Figure 83. HPLC spectrum for (*S*,*E*)-6c.

zj-4-113-as-h-95-5-1-214

实验时间：2015-10-12, 15:35:07　　　　　　报告时间：2015-10-13, 17:38:42
谱图文件:D:\zhuguangjiong\zj\20151010\zj-4-113-as-h-95-5-1-214....org

实验内容简介：

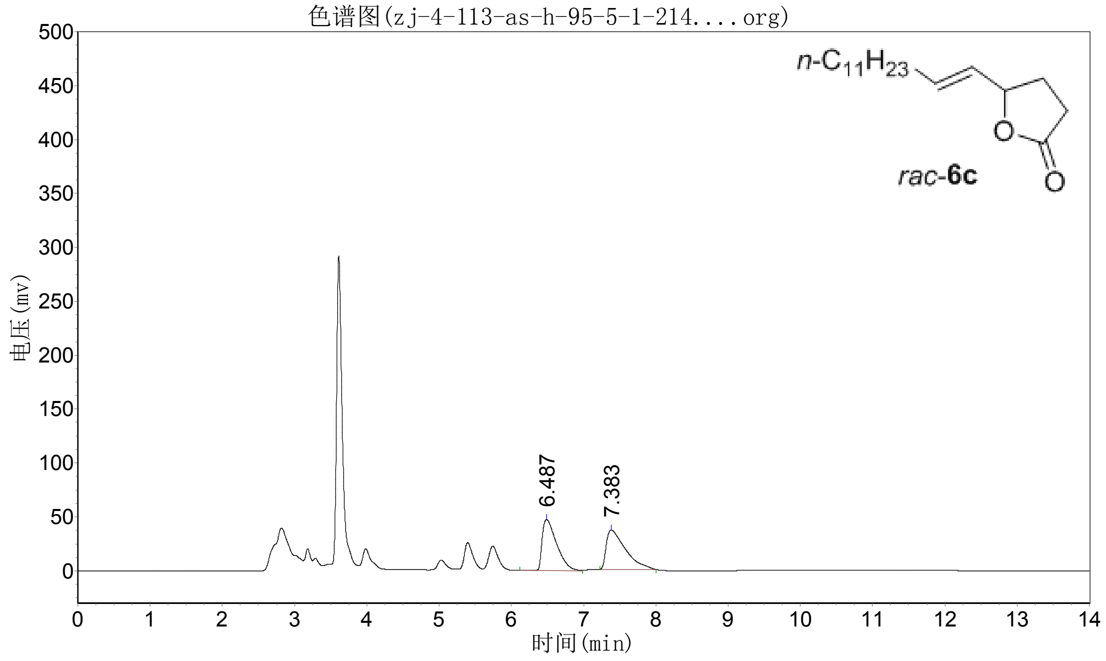

分析结果表

峰号	峰名	保留时间	峰高	峰面积	含量
1		6.487	47584.324	661526.563	49.6280
2		7.383	36512.277	671442.813	50.3720
总计			84096.602	1332969.375	100.0000

Supplementary Figure 84. HPLC spectrum for *rac*-6c.

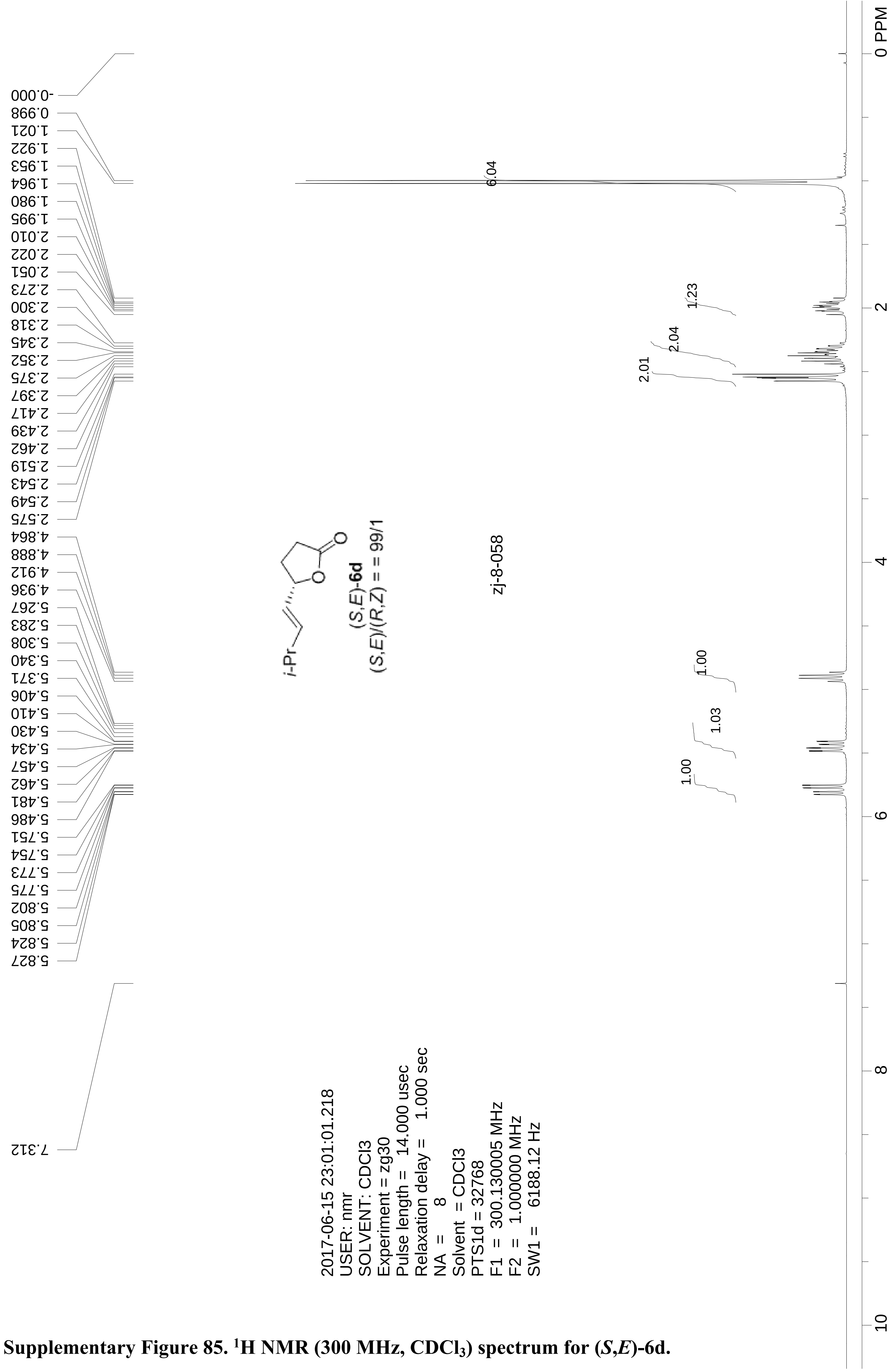

Supplementary Figure 85. ^{1}H NMR (300 MHz, $CDCl_3$) spectrum for (*S*,*E*)-6d.

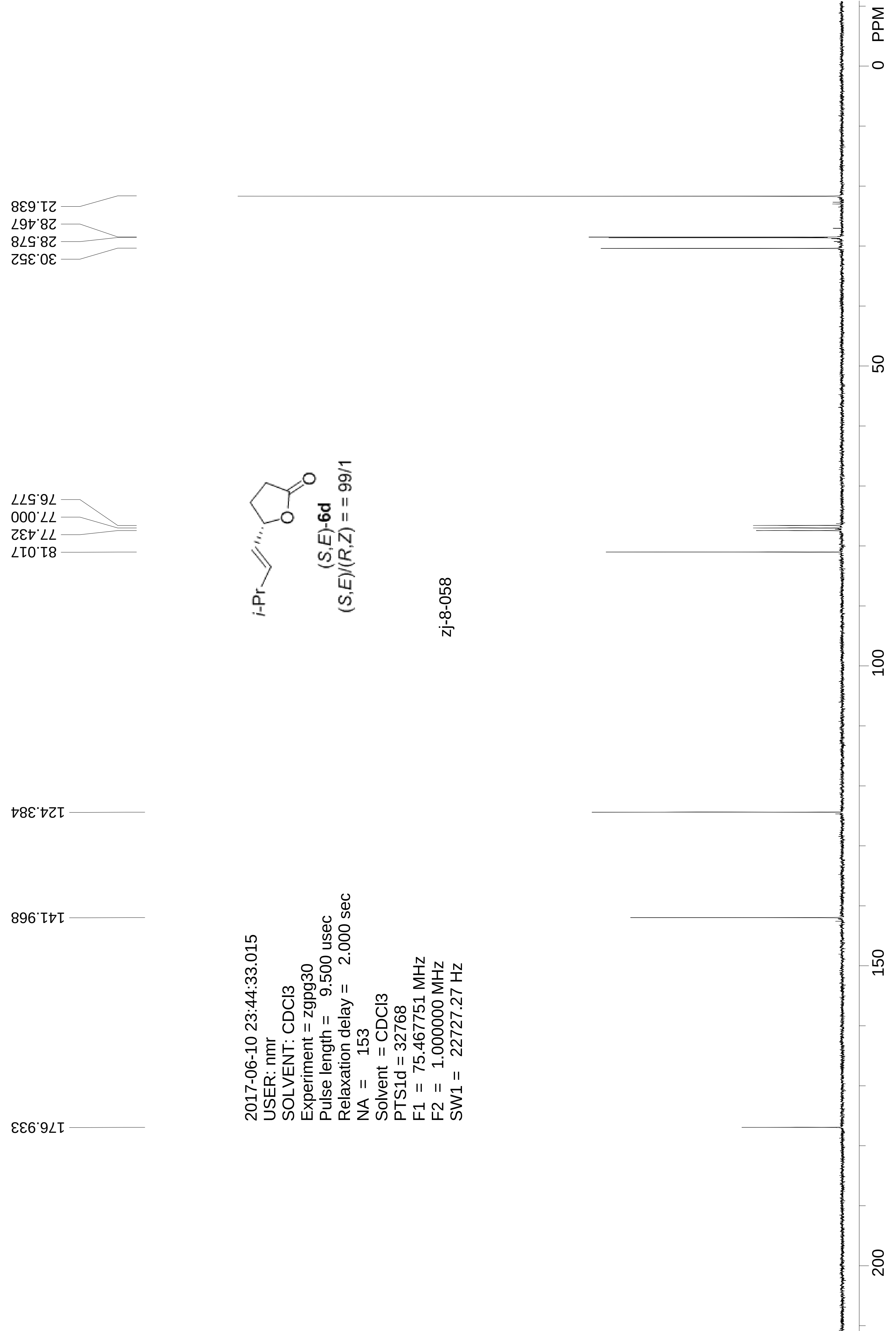

Supplementary Figure 86. **^{13}C NMR (75 MHz, CDCl$_3$) spectrum for (*S*,*E*)-6d.**

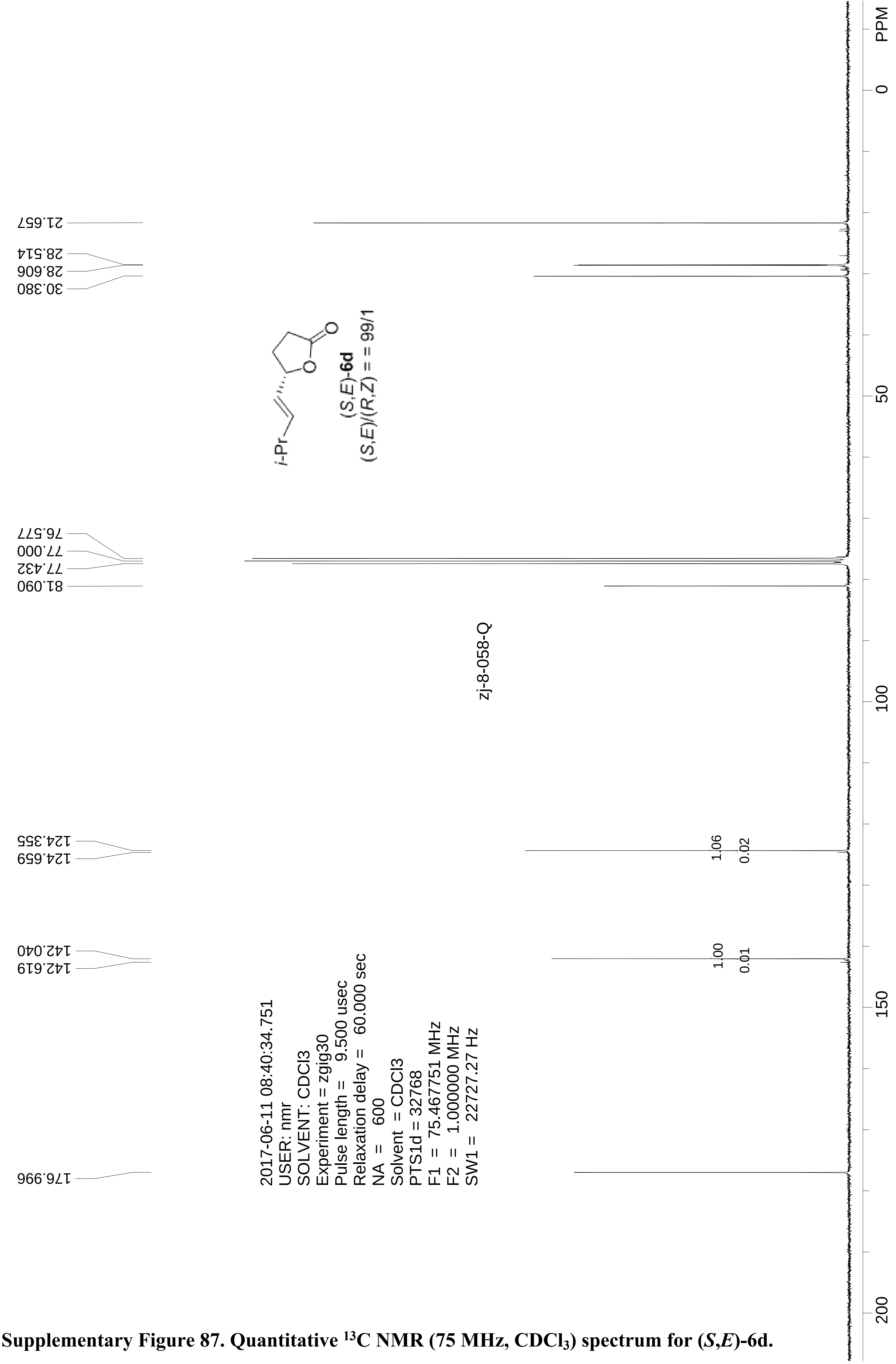

Supplementary Figure 87. Quantitative ^{13}C NMR (75 MHz, $CDCl_3$) spectrum for (*S*,*E*)-6d.

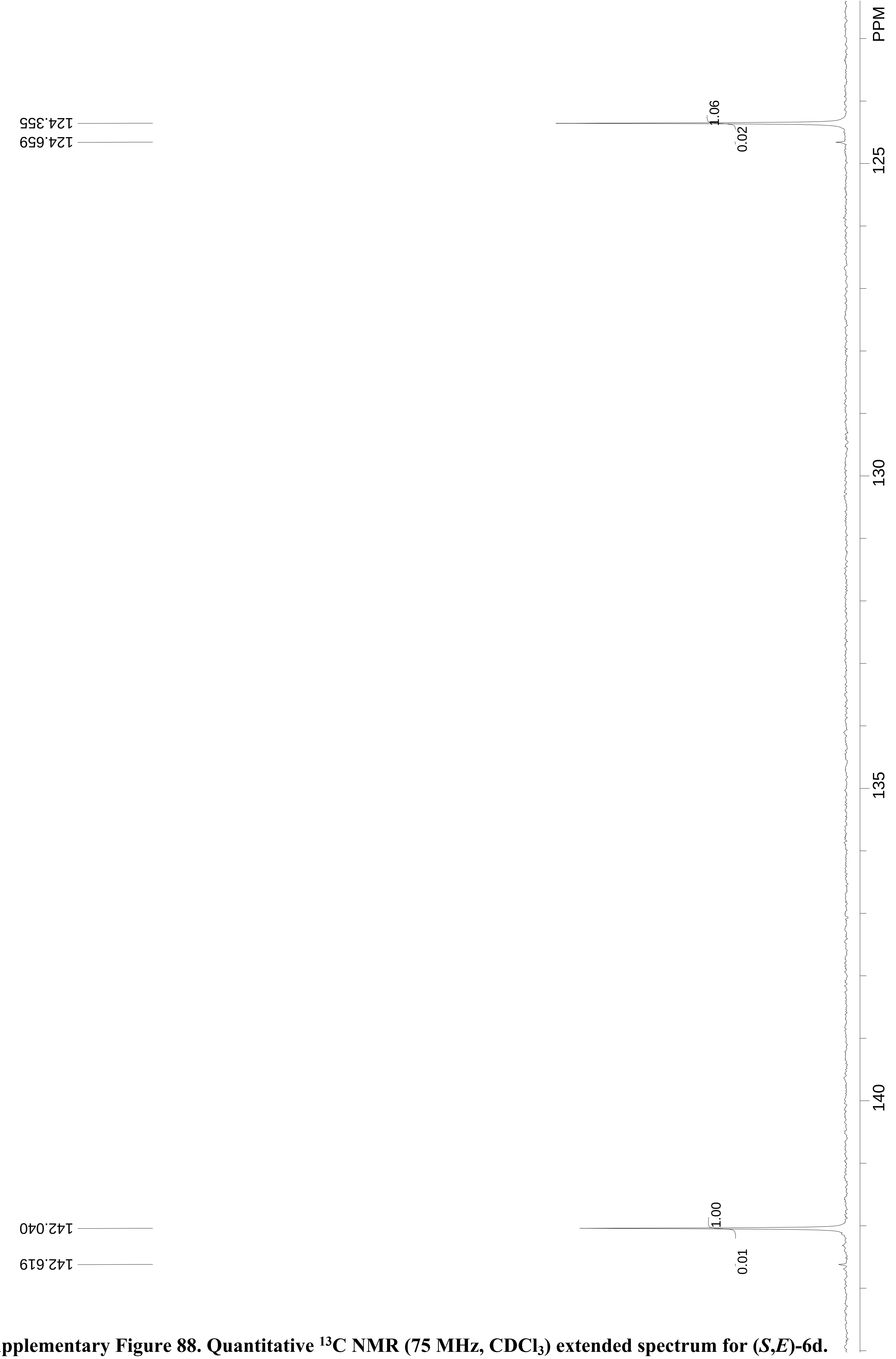

Supplementary Figure 88. Quantitative ^{13}C NMR (75 MHz, $CDCl_3$) extended spectrum for (*S*,*E*)-6d.

zj-8-058-as-h-98-2-1-210

实验时间：2017-06-16, 16:50:01　　　　　　　　　　　　报告时间：2017-06-16, 18:08:14
谱图文件:D:\zhuguangjiong\zj\20170616\zj-8-058-as-h-98-2-1-214..org

实验内容简介：

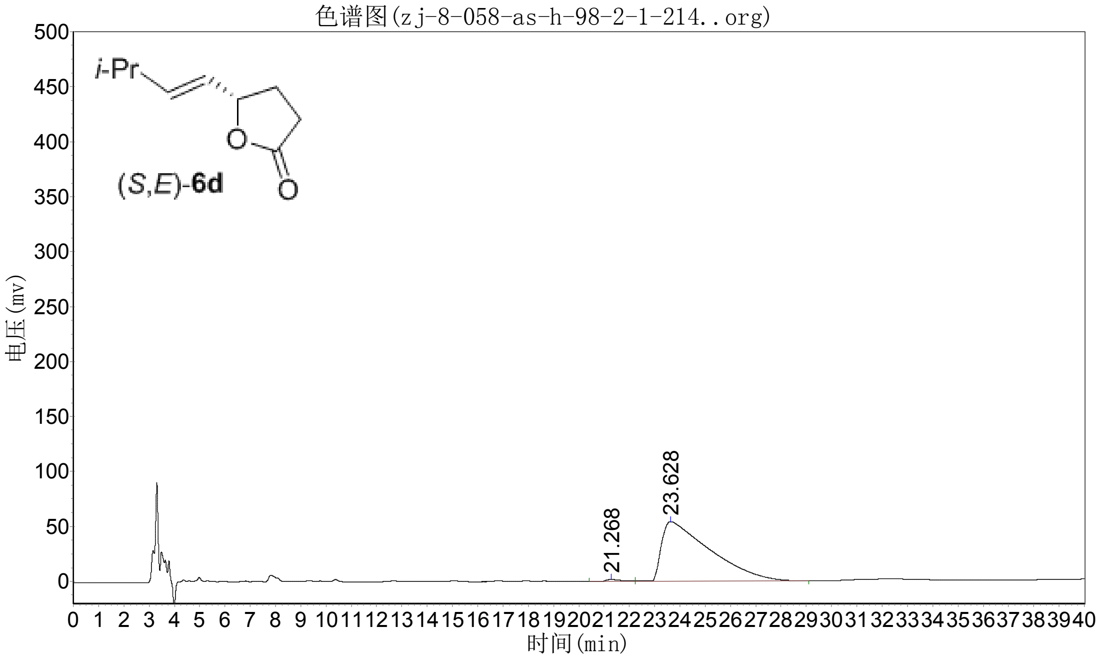

分析结果表

峰号	峰名	保留时间	峰高	峰面积	含量
1		21.268	1837.642	79774.930	1.0875
2		23.628	54305.750	7255602.500	98.9125
总计			56143.392	7335377.430	100.0000

Supplementary Figure 89. HPLC spectrum for (*S*,*E*)-6d.

zj-8-054-as-h-98-2-1-210

实验时间：2017-06-16,17:31:45　　　　报告时间：2017-06-16,18:09:54
谱图文件:D:\zhuguangjiong\zj\20170616\zj-8-054-as-h-98-2-1-214..org

实验内容简介：

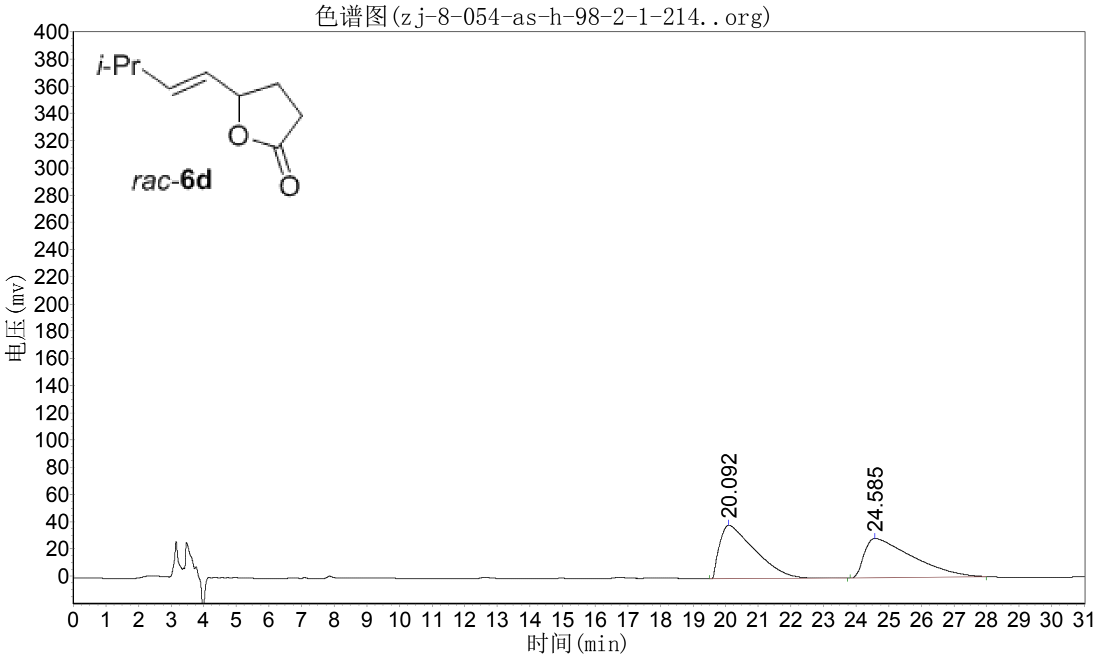

分析结果表

峰号	峰名	保留时间	峰高	峰面积	含量
1		20.092	39390.332	2938623.000	49.8361
2		24.585	28990.822	2957957.000	50.1639
总计			68381.154	5896580.000	100.0000

Supplementary Figure 90. HPLC spectrum for *rac*-6d.

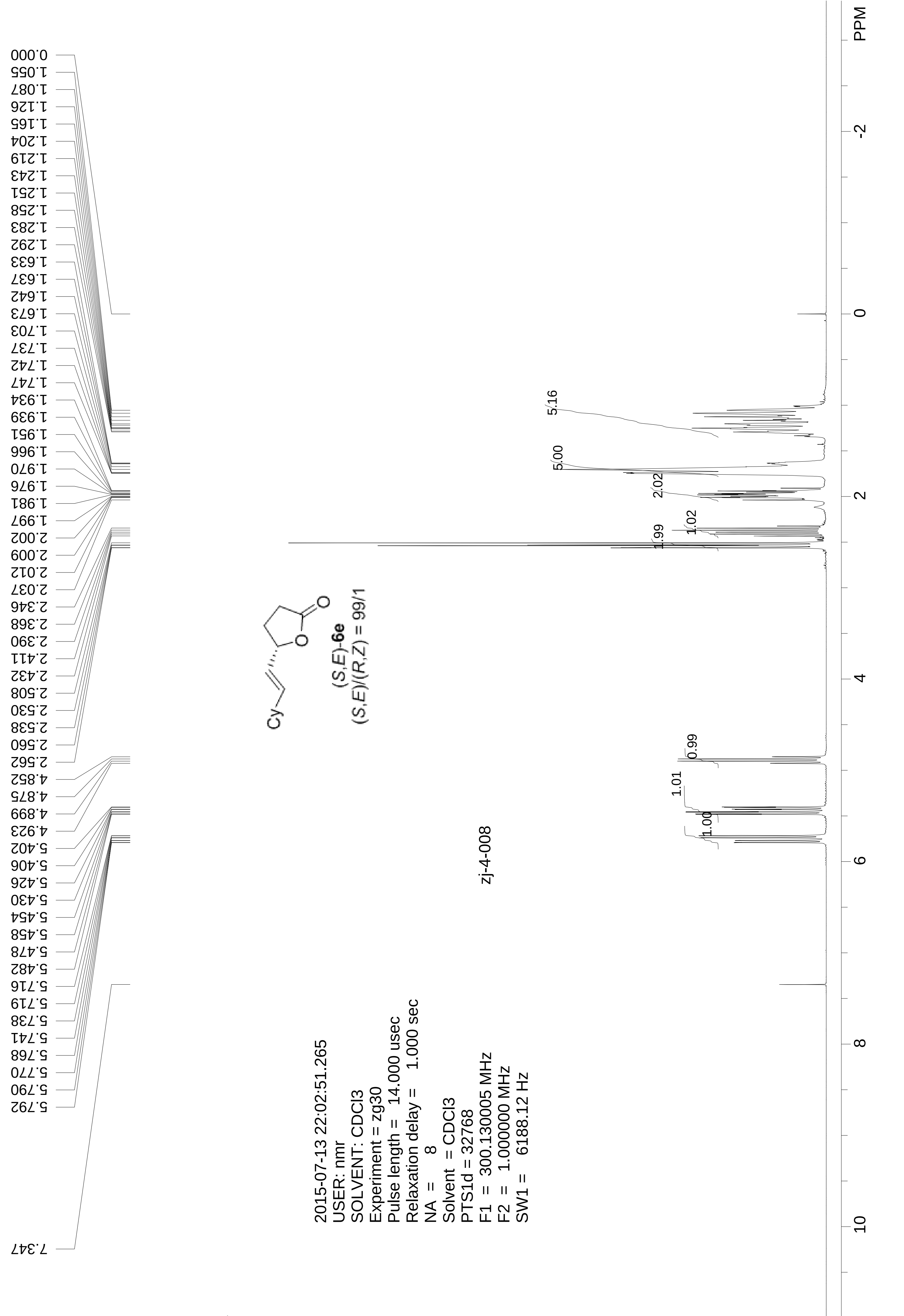

Supplementary Figure 91. ^{1}H NMR (300 MHz, $CDCl_3$) spectrum for (*S*,*E*)-6e.

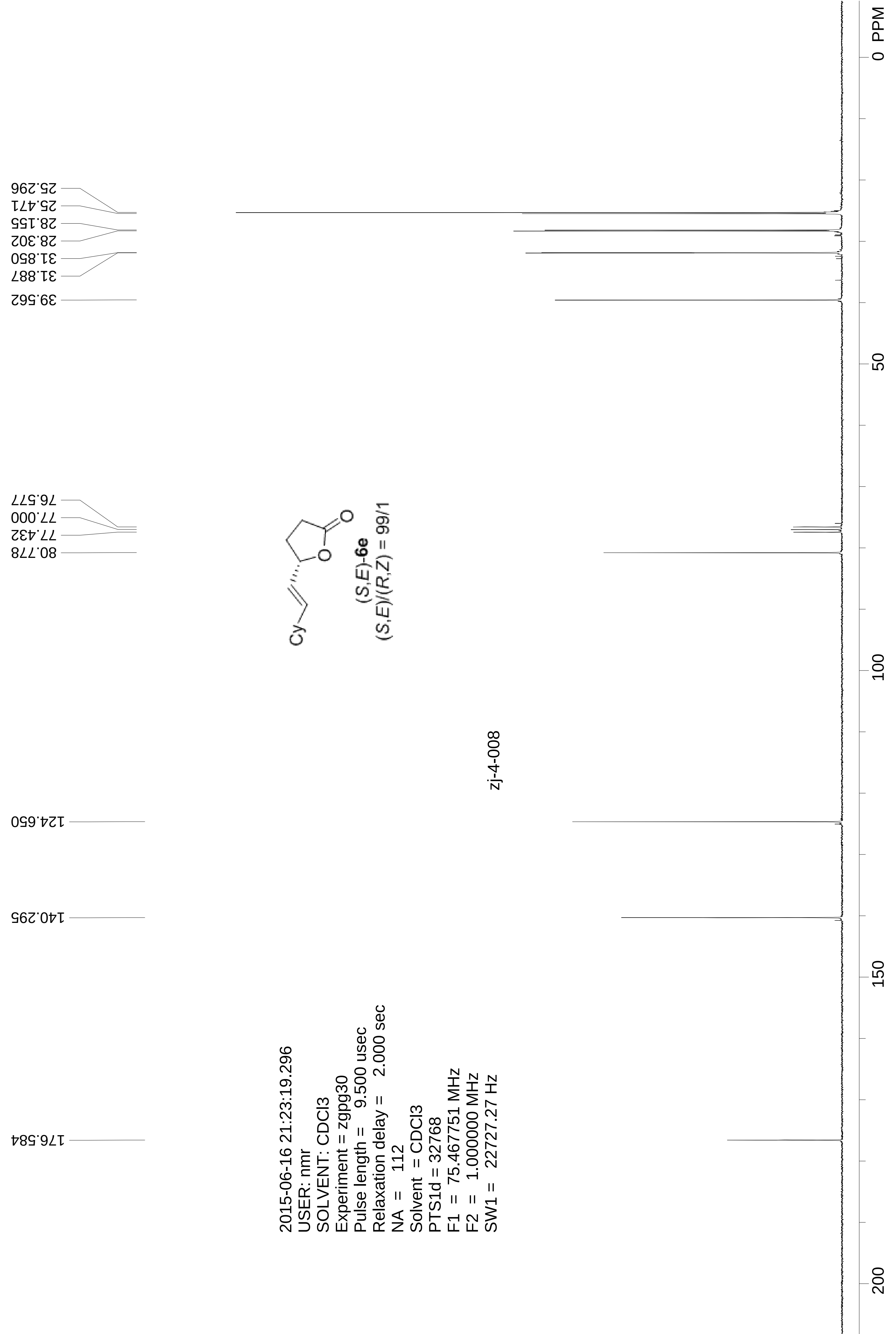

Supplementary Figure 92. ^{13}**C NMR (75 MHz, CDCl$_3$) spectrum for (*S*,*E*)-6e.**

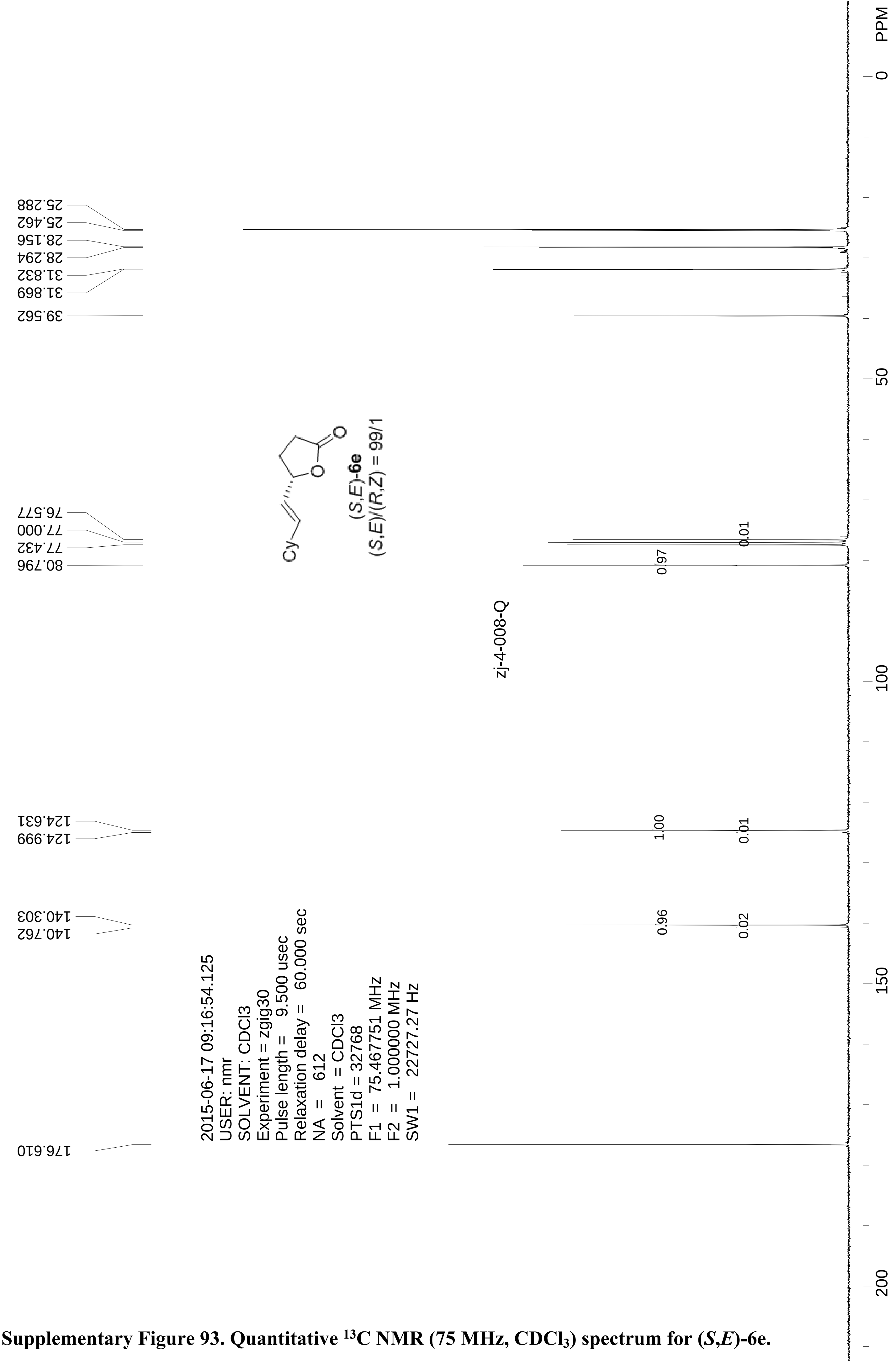

Supplementary Figure 93. Quantitative ^{13}C NMR (75 MHz, $CDCl_3$) spectrum for (*S*,*E*)-6e.

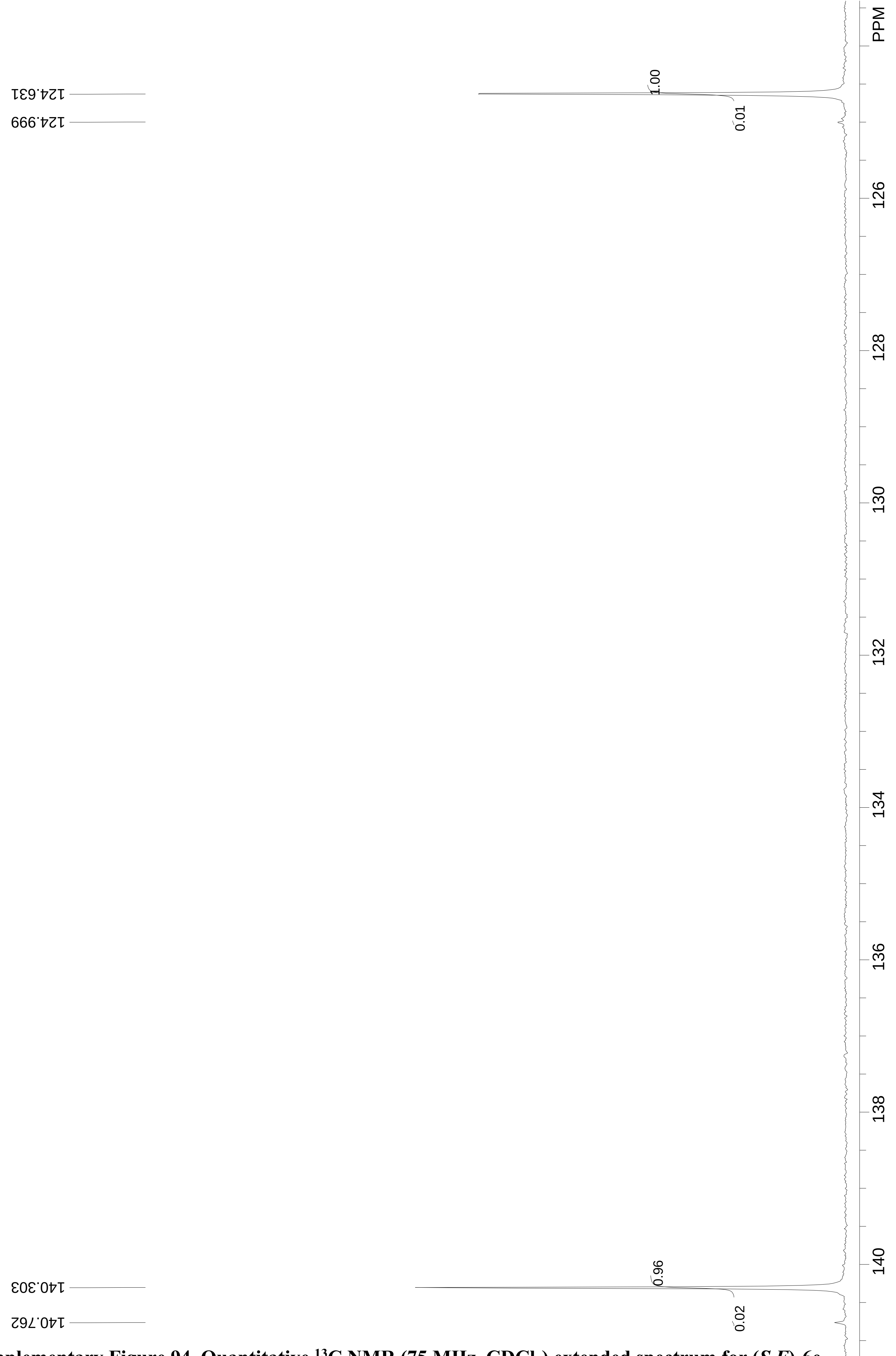

Supplementary Figure 94. Quantitative ^{13}C NMR (75 MHz, $CDCl_3$) extended spectrum for (*S*,*E*)-6e.

zj-4-008-oj-h-95-5-1-214

实验时间：2015-07-23, 15:56:37　　　　报告时间：2015-07-23, 17:03:34
谱图文件:D:\zhuguangjiong\zj\20150720\zj-4-008-oj-h-95-5-1-214..org

实验内容简介：

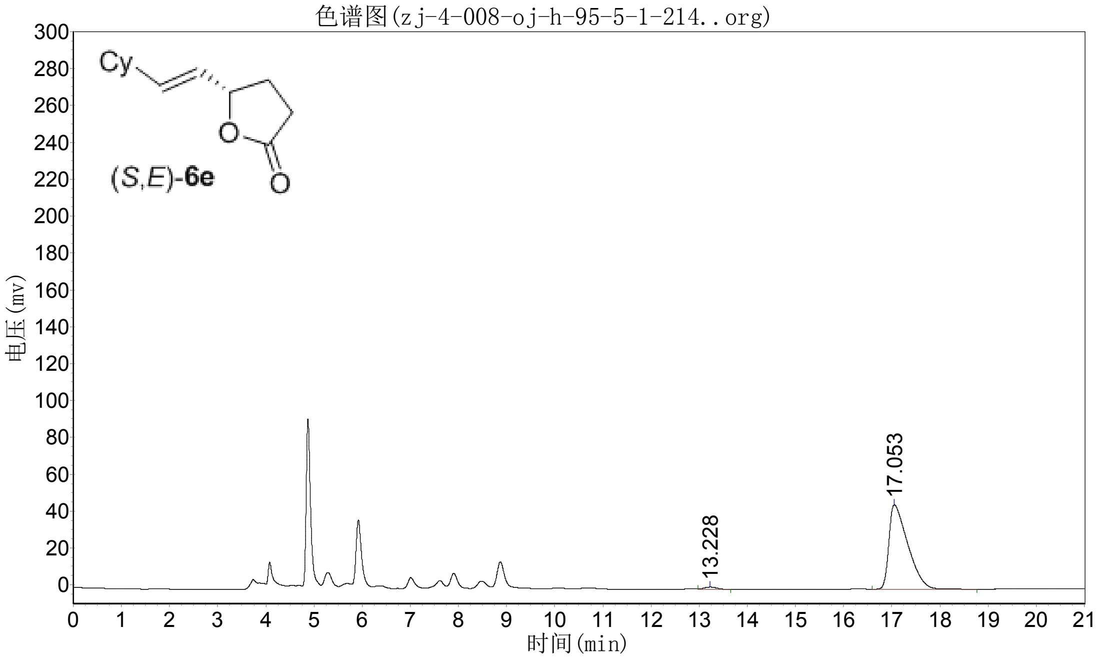

分析结果表

峰号	峰名	保留时间	峰高	峰面积	含量
1		13.228	1296.325	24145.711	1.8860
2		17.053	45912.770	1256106.750	98.1140
总计			47209.095	1280252.461	100.0000

Supplementary Figure 95. HPLC spectrum for (*S*,*E*)-6e.

zj-4-007-oj-h-95-5-1-214

实验时间：2015-07-23, 14:50:22　　　　报告时间：2015-07-23, 17:02:36
谱图文件:D:\zhuguangjiong\zj\20150720\zj-4-007-oj-h-95-5-1-214..org

实验内容简介：

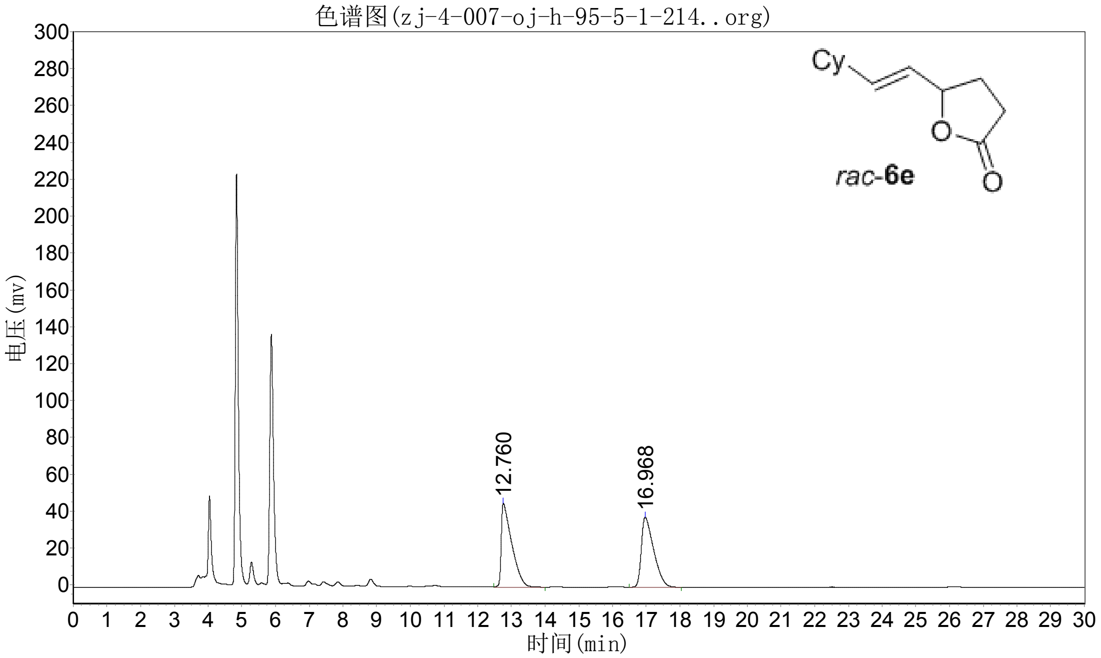

分析结果表

峰号	峰名	保留时间	峰高	峰面积	含量
1		12.760	45605.098	983555.813	50.4990
2		16.968	37913.973	964117.250	49.5010
总计			83519.070	1947673.063	100.0000

Supplementary Figure 96. HPLC spectrum for *rac*-6e.

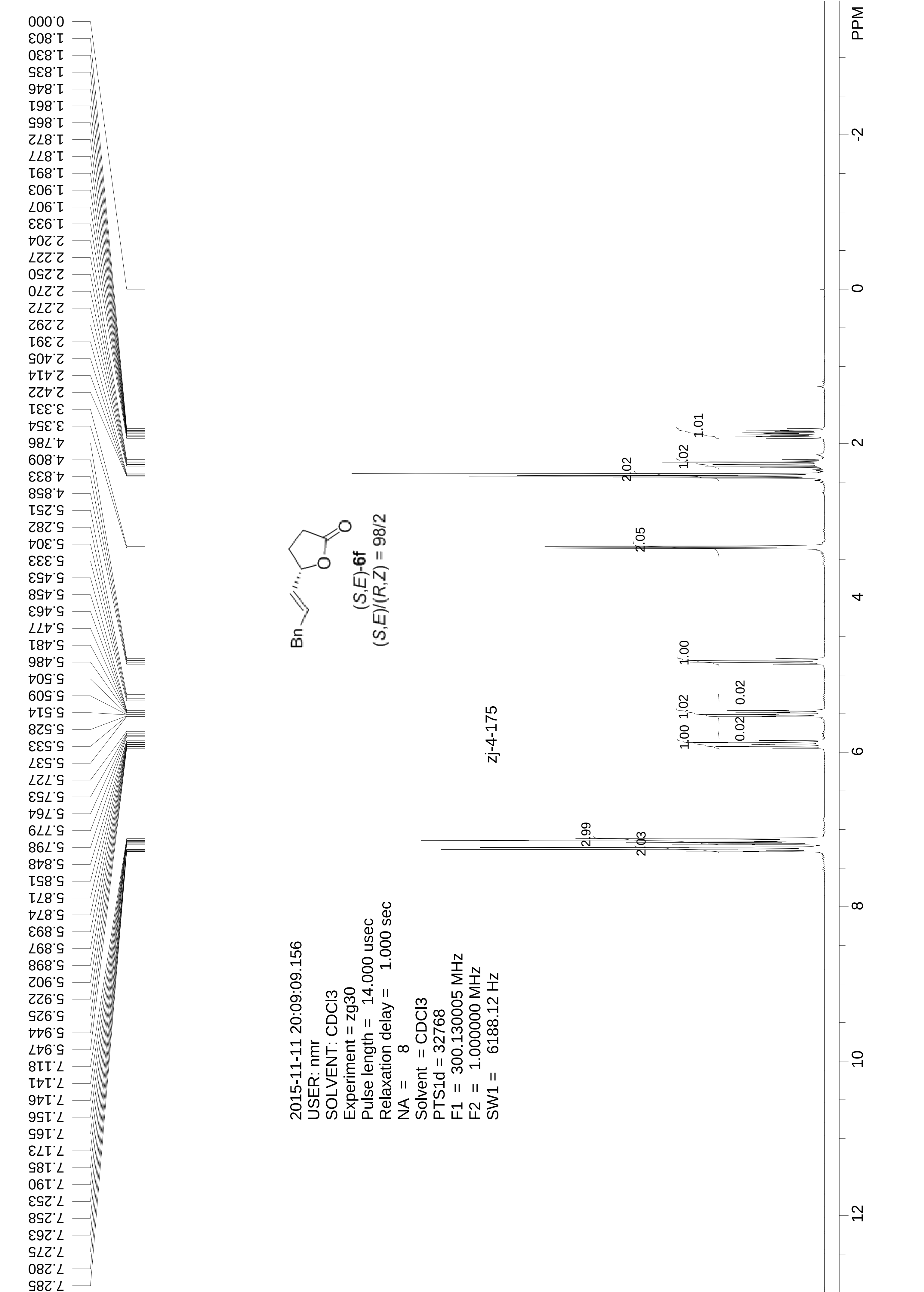

Supplementary Figure 97. ^{1}H NMR (300 MHz, $CDCl_3$) spectrum for (*S*,*E*)-6f.

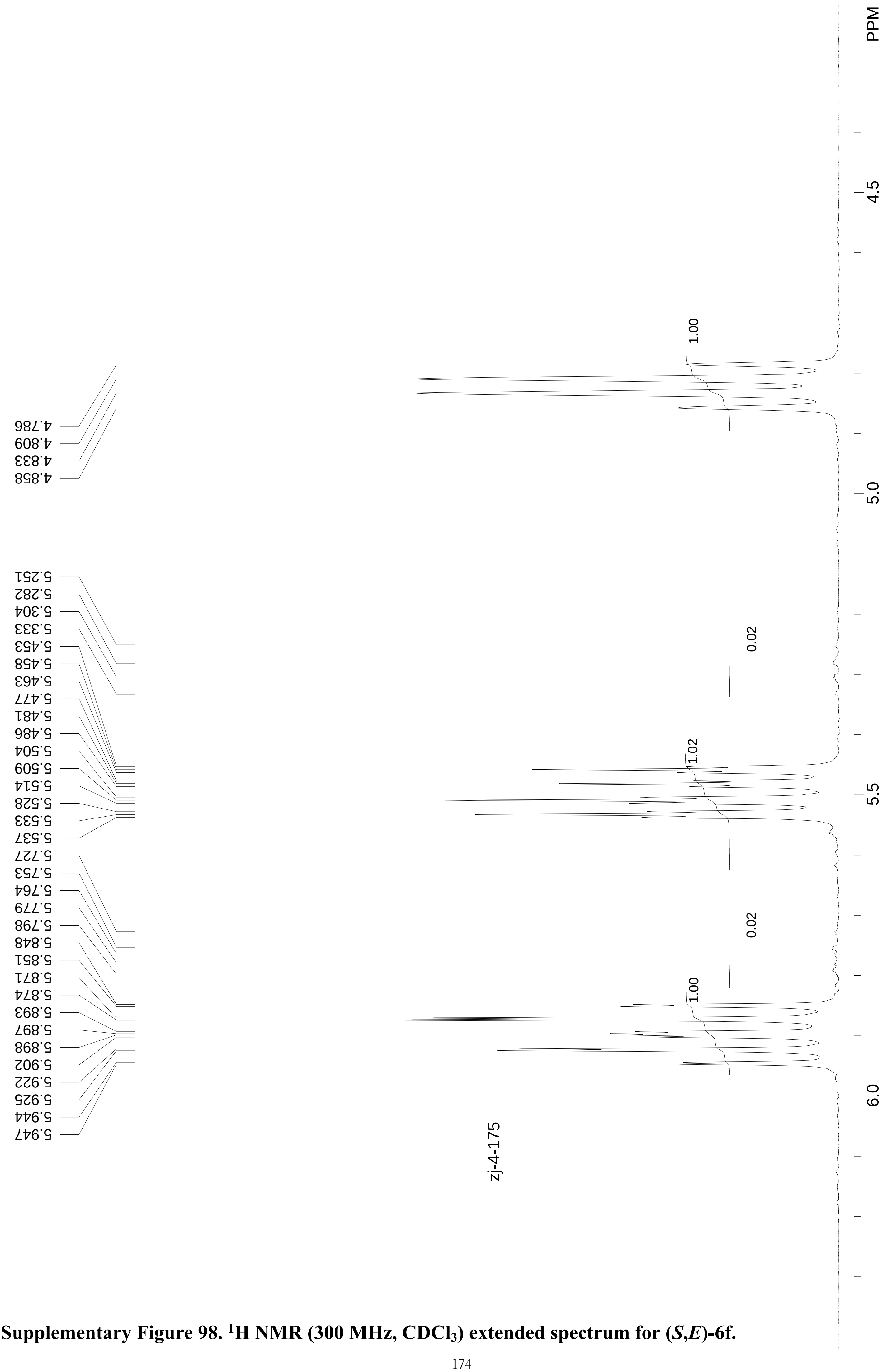

Supplementary Figure 98. ^{1}H NMR (300 MHz, $CDCl_3$) extended spectrum for (*S*,*E*)-6f.

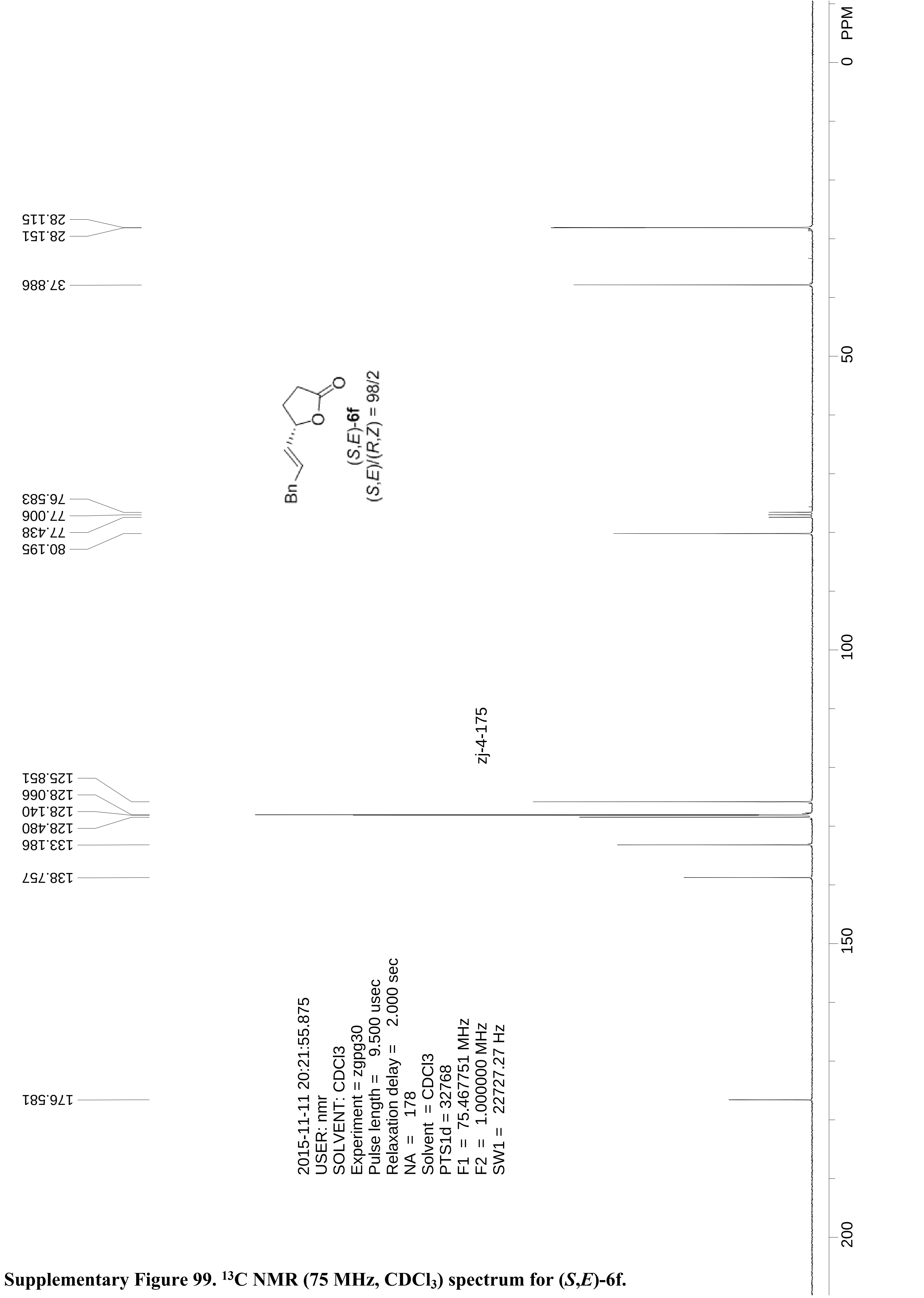

Supplementary Figure 99. ^{13}C NMR (75 MHz, $CDCl_3$) spectrum for (*S*,*E*)-6f.

SAMPLE INFORMATION

Sample Name:	zj-4-175-ib-98-2-1-214	Acquired By:	Breeze
Sample Type:	未知	Date Acquired:	2015/11/18 14:52:12 CST
Vial:	1	Acq. Method:	zgj98
Injection #:	26	Date Processed:	2015/11/18 16:40:28 CST
Injection Volume:	25.00 ul	Channel Name:	W2489 ChA
Run Time:	60.00 Minutes	Channel Desc.:	W2489 ChA 214nm
Column Type:		Sample Set Name:	

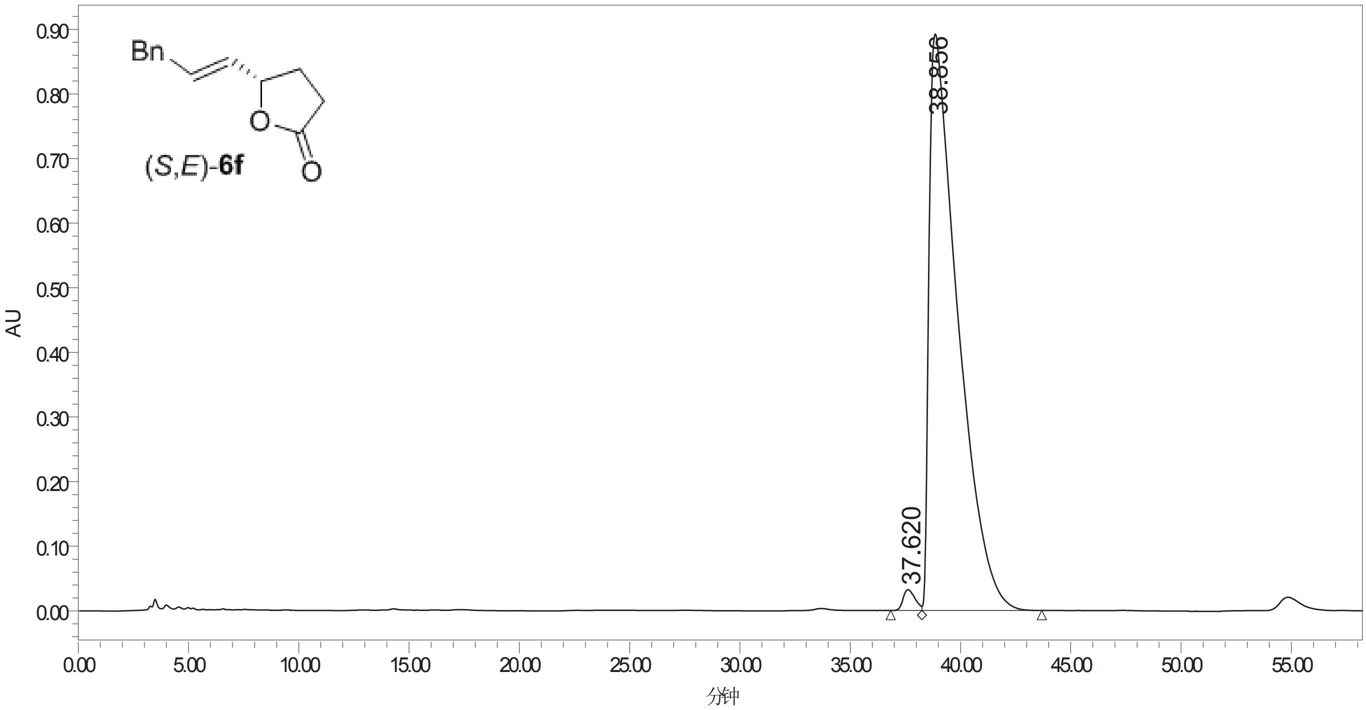

	RT (min)	Area (礦sec)	% Area	Height (礦)	% Height
1	37.620	1210782	1.48	32191	3.48
2	38.856	80621974	98.52	892039	96.52

Supplementary Figure 100. HPLC spectrum for (*S*,*E*)-6f.

中国科学院上海有机化学研究所

Project Name: defaults for copy

Reported by User: Breeze user (Breeze)

SAMPLE INFORMATION

Sample Name:	zj-4-180-ib-98-2-1-214	Acquired By:	Breeze
Sample Type:	未知	Date Acquired:	2015/11/18 13:55:48 CST
Vial:	1	Acq. Method:	zgj98
Injection #:	25	Date Processed:	2015/11/18 14:50:43 CST
Injection Volume:	25.00 ul	Channel Name:	W2489 ChA
Run Time:	60.00 Minutes	Channel Desc.:	W2489 ChA 214nm
Column Type:		Sample Set Name:	

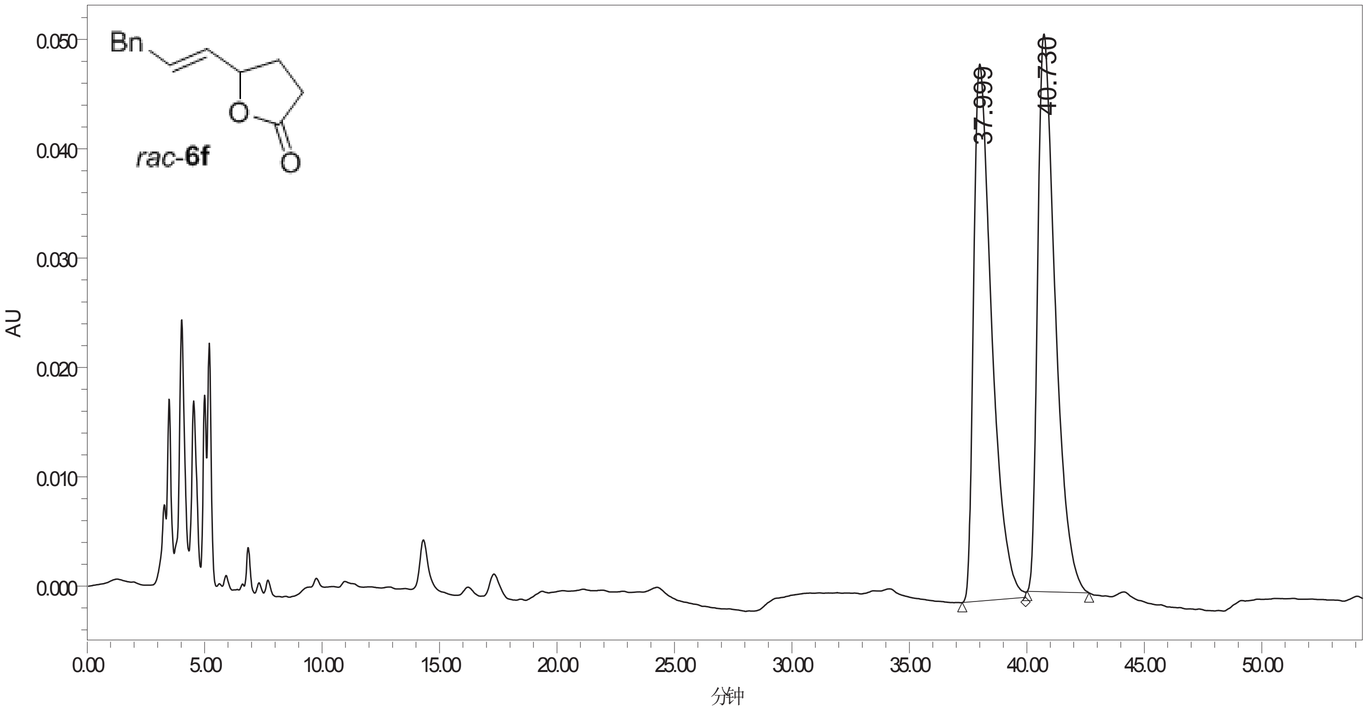

	RT (min)	Area (磺sec)	% Area	Height (磺)	% Height
1	37.999	2647274	49.80	49115	49.06
2	40.730	2668538	50.20	51007	50.94

Report Method: Individual Report ASC

Page: 1 (共计 1)

Printed: 2015/11/18 16:41:39 PRC

Supplementary Figure 101. HPLC spectrum for *rac*-6f.

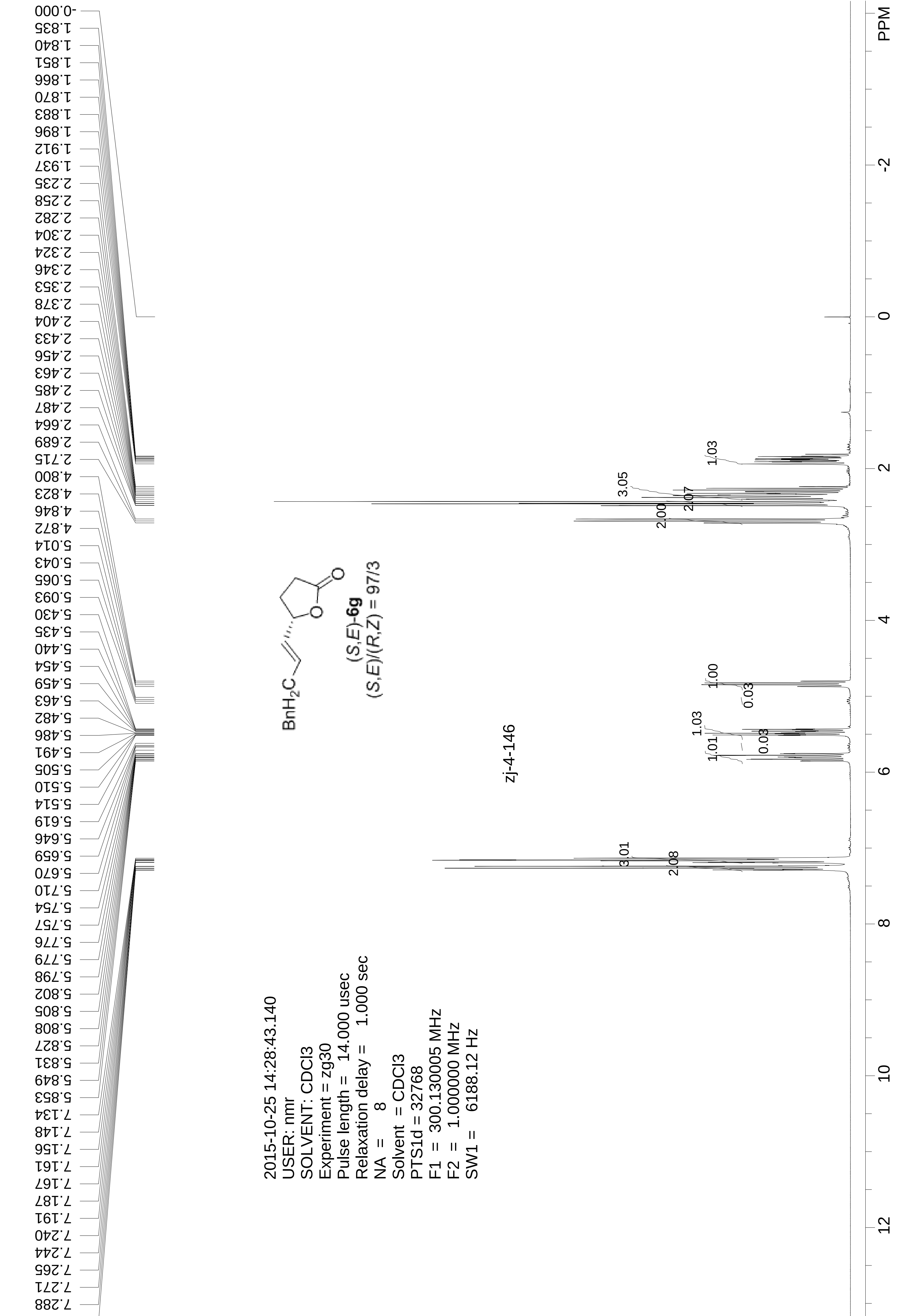

Supplementary Figure 102. **^{1}H NMR (300 MHz, $CDCl_3$) spectrum for (*S*,*E*)-6g.**

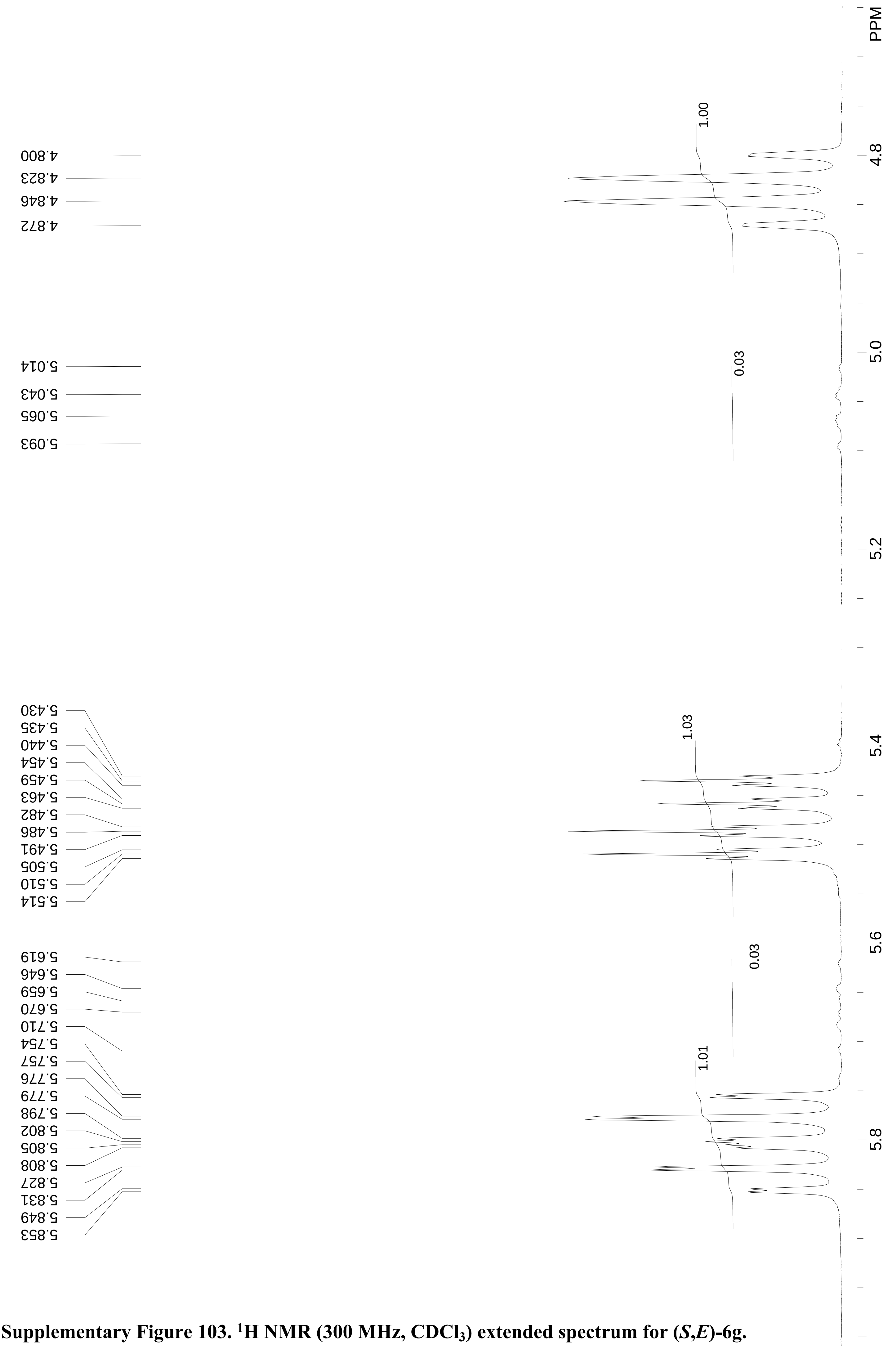

Supplementary Figure 103. ^{1}H NMR (300 MHz, CDCl$_3$) extended spectrum for (*S*,*E*)-6g.

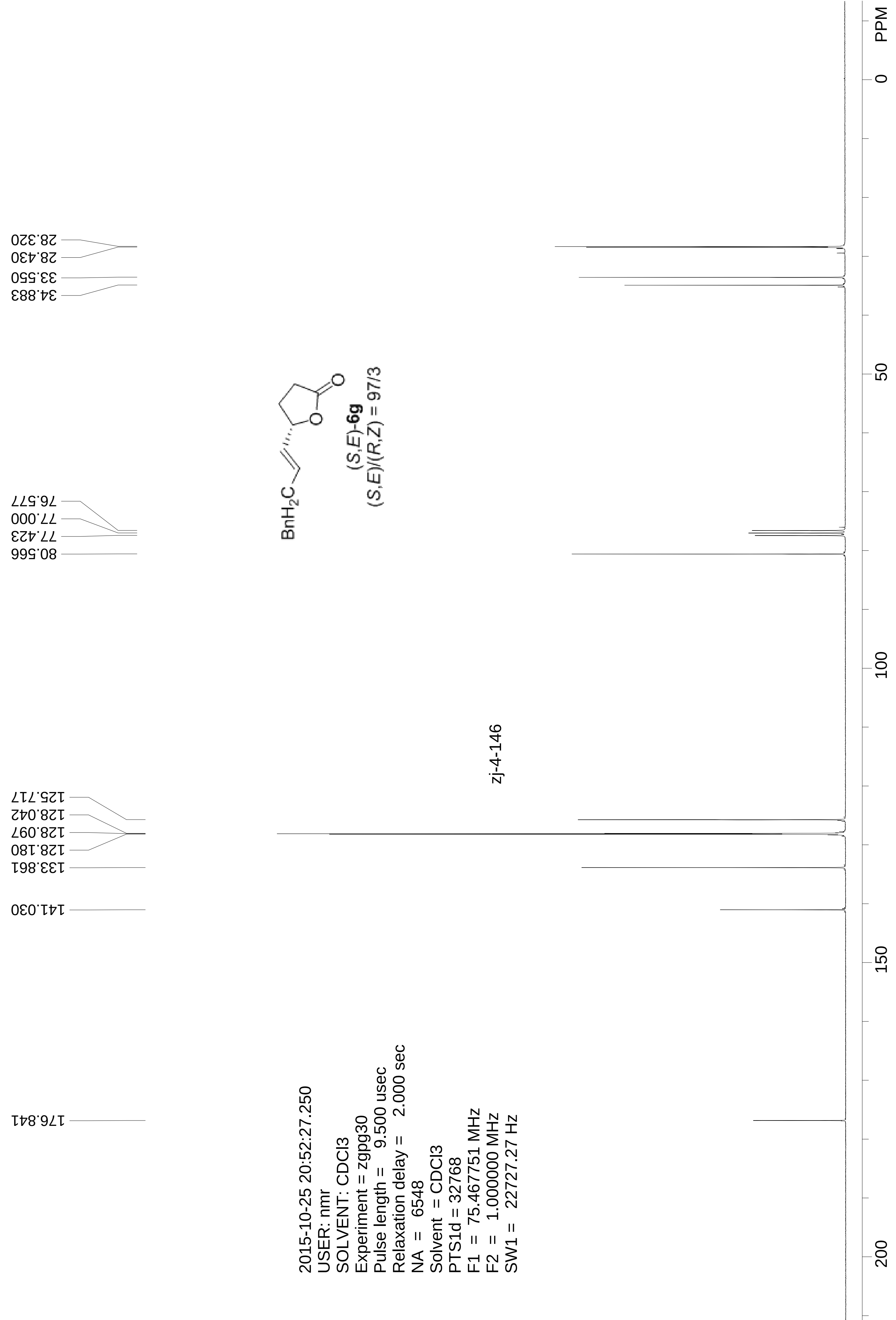

Supplementary Figure 104. ^{13}C NMR (75 MHz, $CDCl_3$) spectrum for (*S*,*E*)-6g.

中国科学院上海有机化学研究所

Project Name: defaults for copy

Reported by User: Breeze user (Breeze)

Breeze 2 HPLC System

SAMPLE INFORMATION

Sample Name:	zj-4-146-od-90-10-0.7-214	Acquired By:	Breeze
Sample Type:	未知	Date Acquired:	2015/10/28 17:23:39 CST
Vial:	1	Acq. Method:	zgj90
Injection #:	62	Date Processed:	2015/10/28 19:40:21 CST
Injection Volume:	25.00 ul	Channel Name:	W2489 ChA
Run Time:	45.00 Minutes	Channel Desc.:	W2489 ChA 214nm
Column Type:		Sample Set Name:	

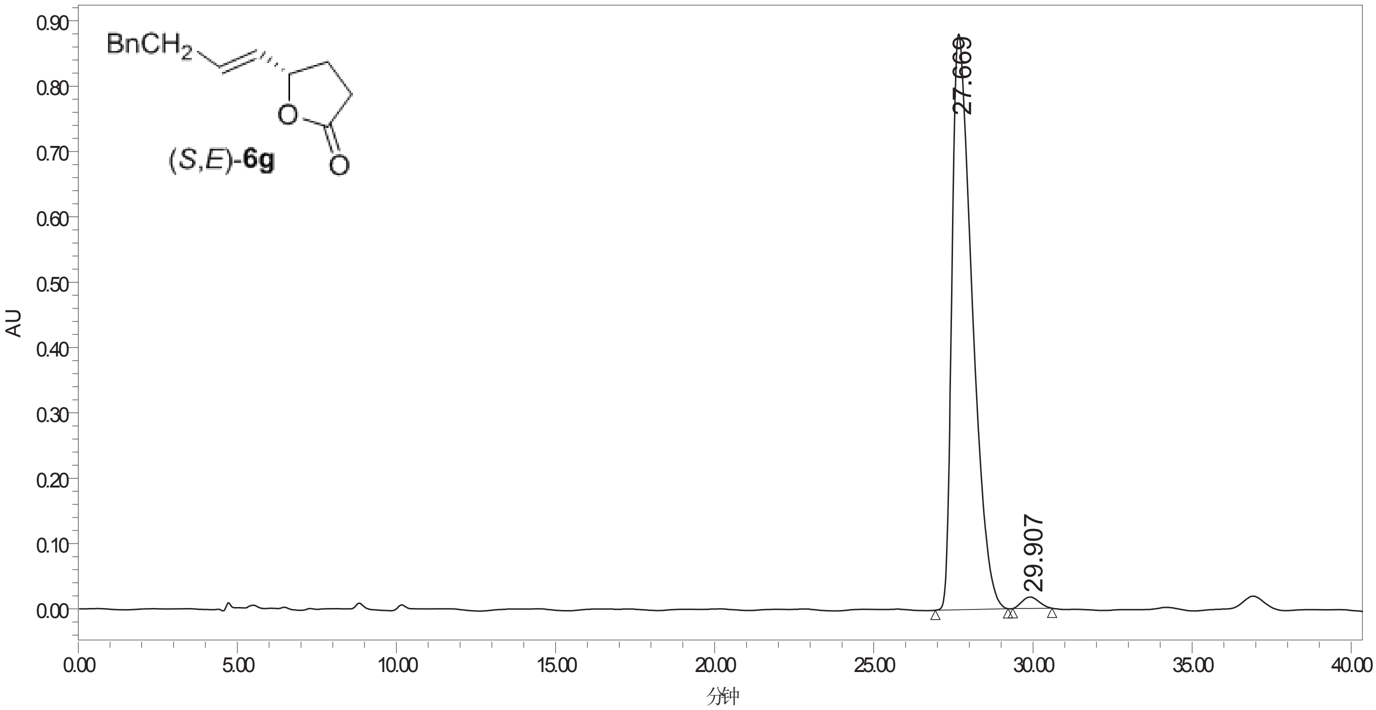

	RT (min)	Area (微伏*sec)	% Area	Height (微伏)	% Height
1	27.669	38977022	98.36	880919	98.06
2	29.907	648033	1.64	17390	1.94

Report Method: Individual Report ASC

Page: 1 (共计 1)

Printed: 2015/10/28 20:40:08 PRC

Supplementary Figure 105. HPLC spectrum for (*S*,*E*)-6g.

中国科学院上海有机化学研究所

Project Name: defaults for copy

Reported by User: Breeze user (Breeze)

Breeze 2 HPLC System

SAMPLE INFORMATION

Sample Name:	zj-4-144-od-90-10-0.7-214	Acquired By:	Breeze
Sample Type:	未知	Date Acquired:	2015/10/28 16:42:27 CST
Vial:	1	Acq. Method:	zgj90
Injection #:	61	Date Processed:	2015/10/28 19:37:38 CST
Injection Volume:	25.00 ul	Channel Name:	W2489 ChA
Run Time:	45.00 Minutes	Channel Desc.:	W2489 ChA 214nm
Column Type:		Sample Set Name:	

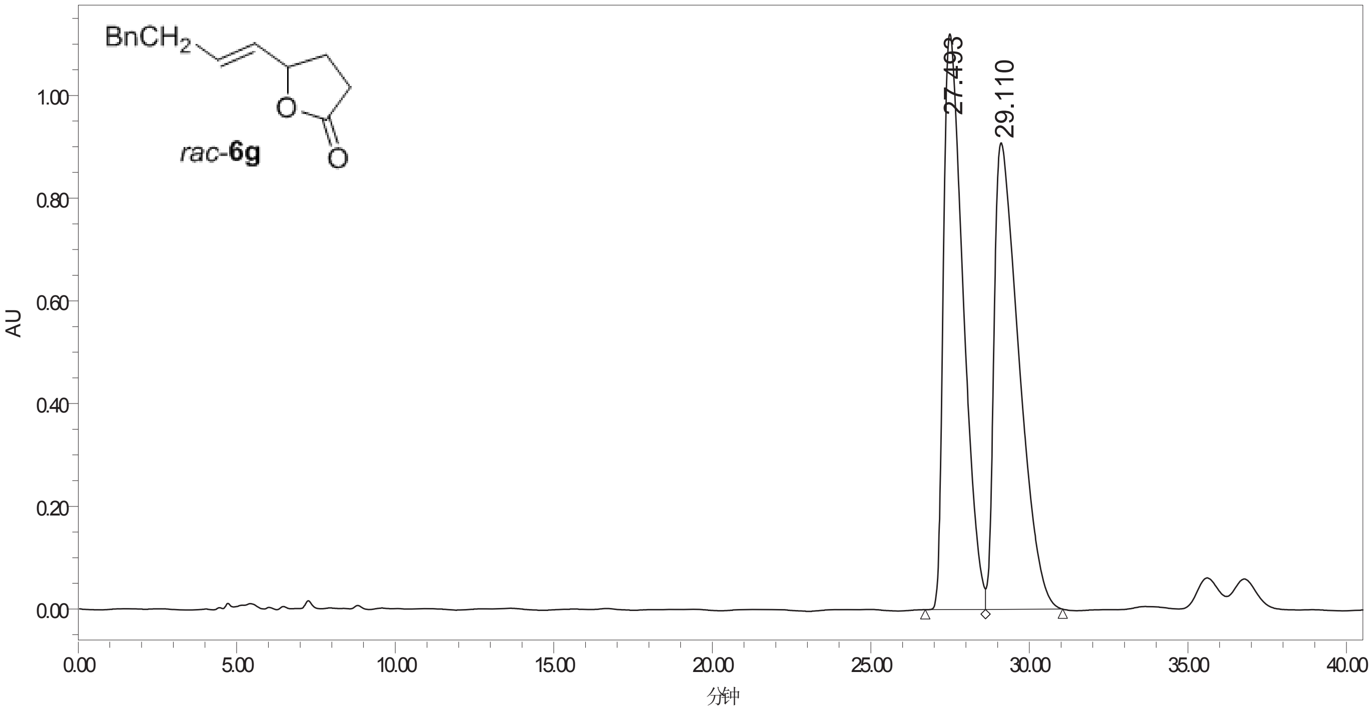

	RT (min)	Area (磺*sec)	% Area	Height (磺)	% Height
1	27.493	48700978	49.51	1120879	55.23
2	29.110	49662673	50.49	908598	44.77

Report Method: Individual Report ASC

Page: 1 (共计 1)

Printed: 2015/10/28 20:40:22 PRC

Supplementary Figure 106. HPLC spectrum for *rac*-6g.

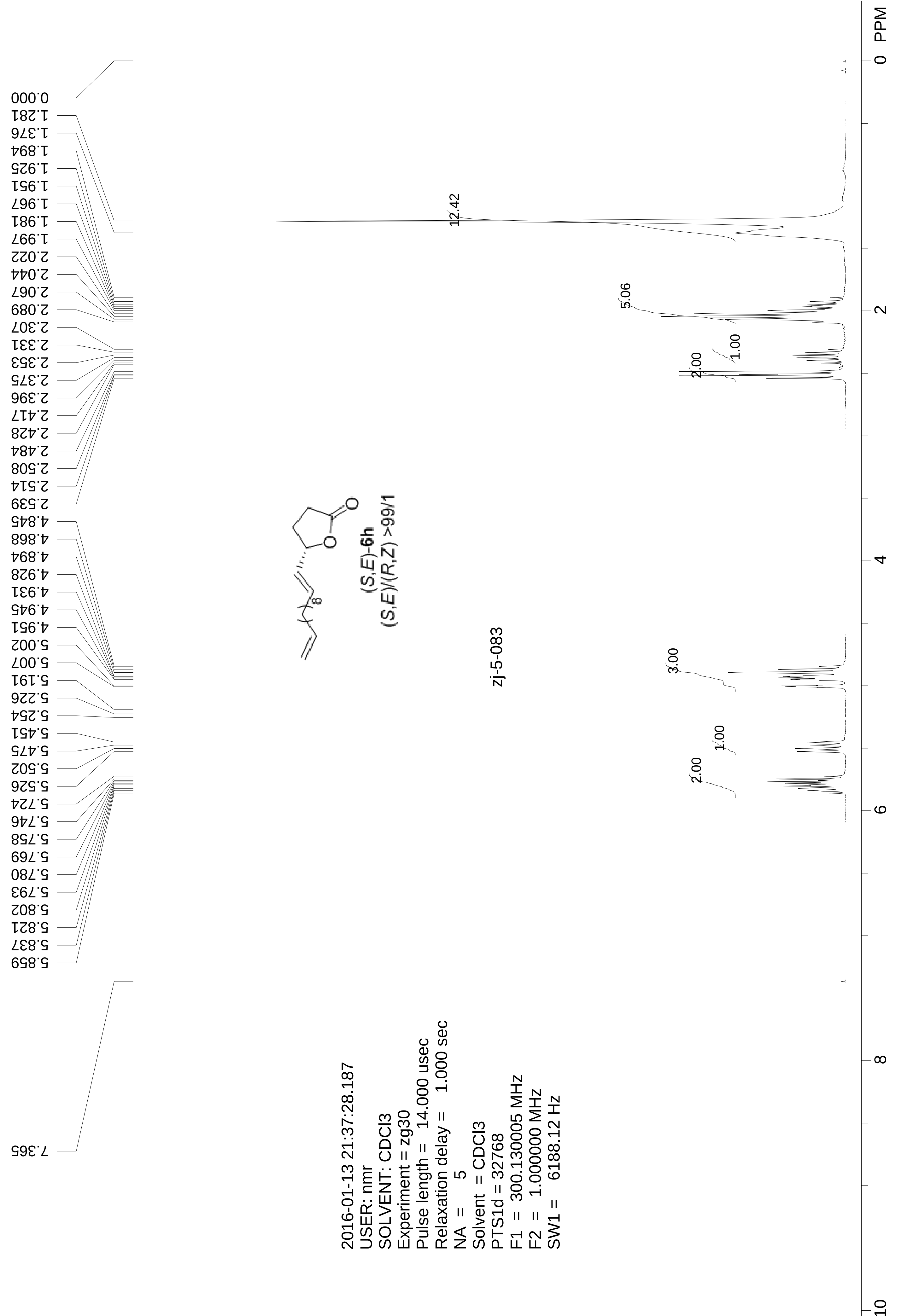

Supplementary Figure 107. ^{1}H NMR (300 MHz, $CDCl_3$) spectrum for (*S*,*E*)-6h.

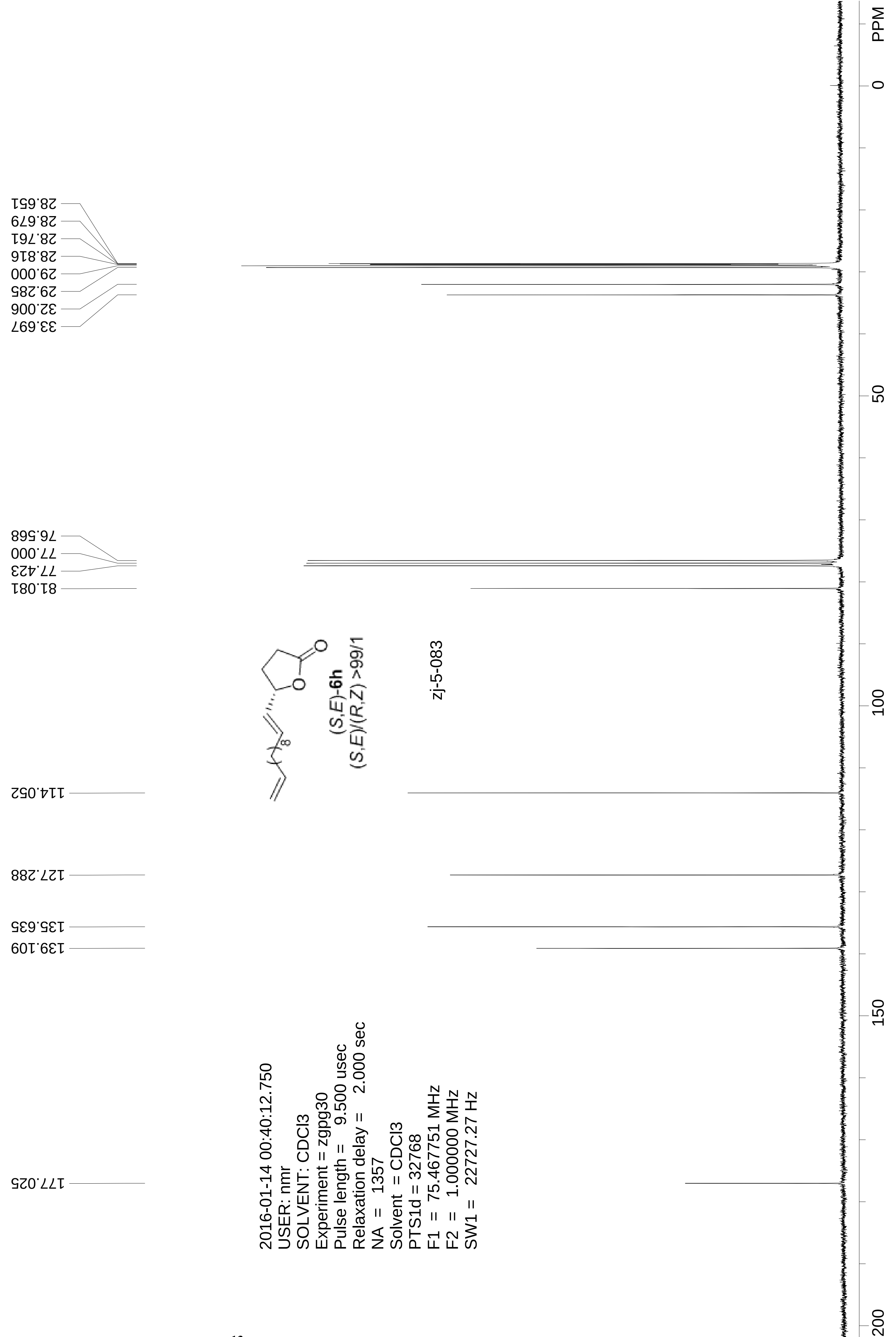

Supplementary Figure 108. ^{13}C NMR (75 MHz, $CDCl_3$) spectrum for (*S*,*E*)-6h.

842 ZJ-5-83 ADH 982 214 0.7

Sample Name:	**ZJ-5-83 ADH 982 214 0.7**	*Injection Volume:*	**9.0**
Vial Number:	**RD5**	*Channel:*	**UV_VIS_2**
Sample Type:	**unknown**	*Wavelength:*	**214.0**
Control Program:	**test-dad**	*Bandwidth:*	**4**
Quantif. Method:	**WXL**	*Dilution Factor:*	**1.0000**
Recording Time:	**2016-1-25 10:47**	*Sample Weight:*	**1.0000**
Run Time (min):	**21.00**	*Sample Amount:*	**1.0000**

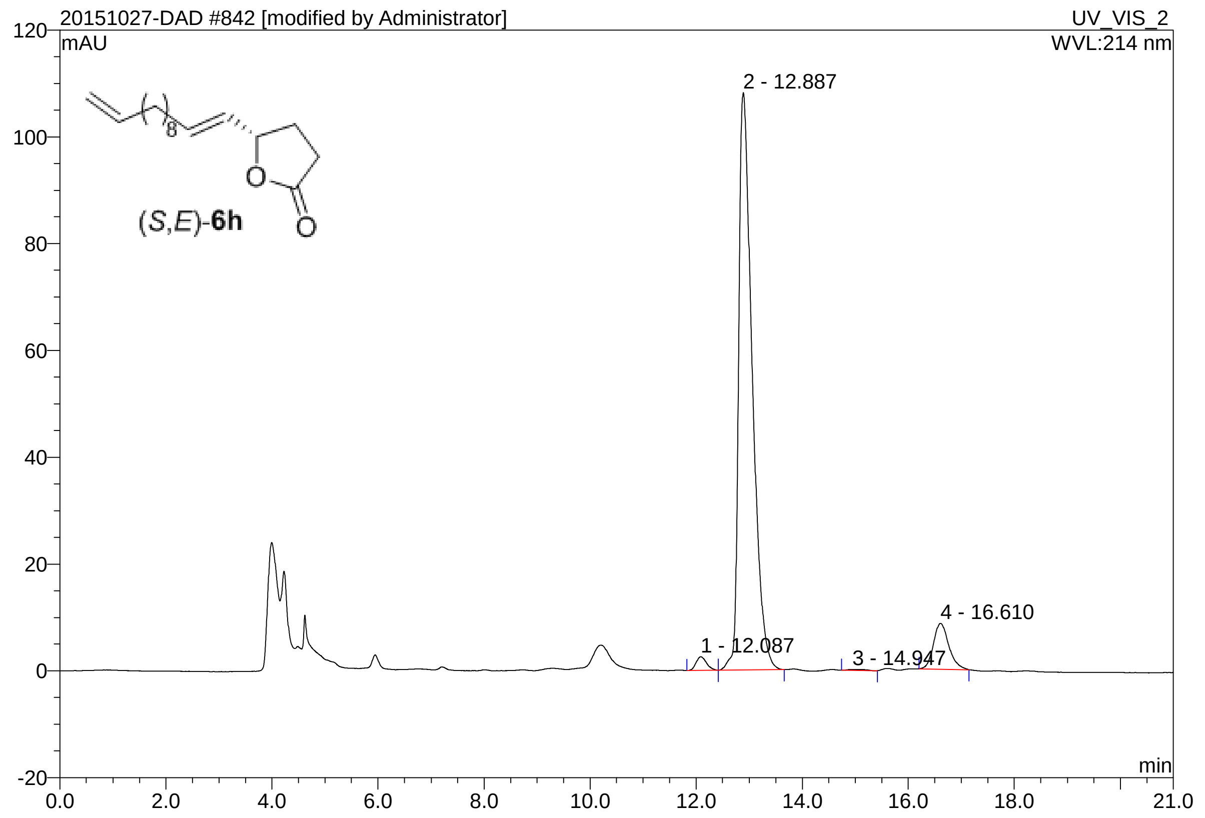

No.	Ret.Time min	Peak Name	Height mAU	Area mAU*min	Rel.Area %	Amount	Type
1	12.09	n.a.	2.565	0.587	1.65	n.a.	BM
2	12.89	n.a.	108.157	32.052	90.05	n.a.	MB
3	14.95	n.a.	0.180	0.064	0.18	n.a.	BMB*
4	16.61	n.a.	8.639	2.889	8.12	n.a.	BMB
**Total:**			119.541	35.592	100.00	0.000	

Supplementary Figure 109. SFC (supercritical fluid chromatogram) spectrum for (*S*,*E*)-6h.

841 ZJ-5-82+- ADH 982 214 0.7

Sample Name:	**ZJ-5-82+- ADH 982 214 0.7**	*Injection Volume:*	**9.0**
Vial Number:	**RE5**	*Channel:*	**UV_VIS_2**
Sample Type:	**unknown**	*Wavelength:*	**214.0**
Control Program:	**test-dad**	*Bandwidth:*	**4**
Quantif. Method:	**WXL**	*Dilution Factor:*	**1.0000**
Recording Time:	**2016-1-25 10:25**	*Sample Weight:*	**1.0000**
Run Time (min):	**21.00**	*Sample Amount:*	**1.0000**

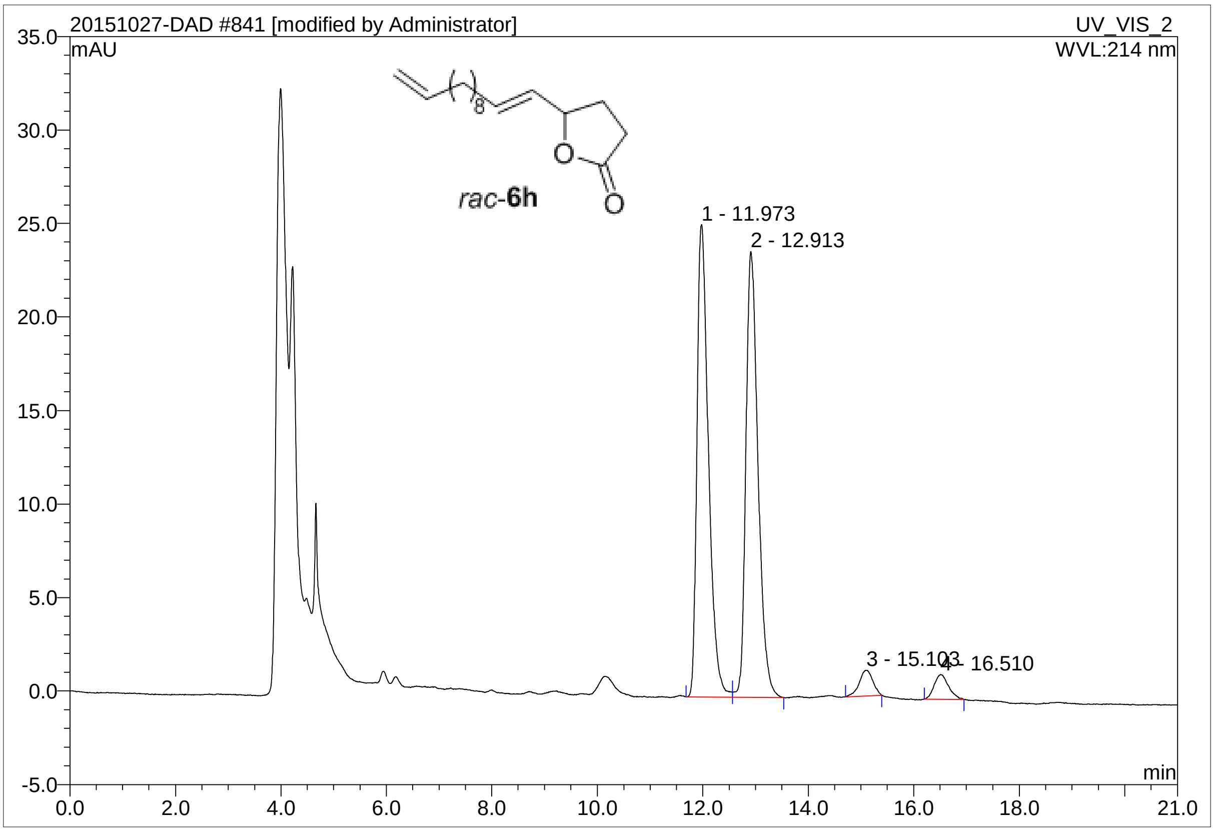

No.	Ret.Time min	Peak Name	Height mAU	Area mAU*min	Rel.Area %	Amount	Type
1	11.97	n.a.	25.262	6.383	47.00	n.a.	BM
2	12.91	n.a.	23.836	6.366	46.88	n.a.	MB
3	15.10	n.a.	1.374	0.413	3.04	n.a.	BMB
4	16.51	n.a.	1.322	0.418	3.08	n.a.	BMB
**Total:**			51.794	13.579	100.00	0.000	

Supplementary Figure 110. SFC spectrum for *rac*-6h.

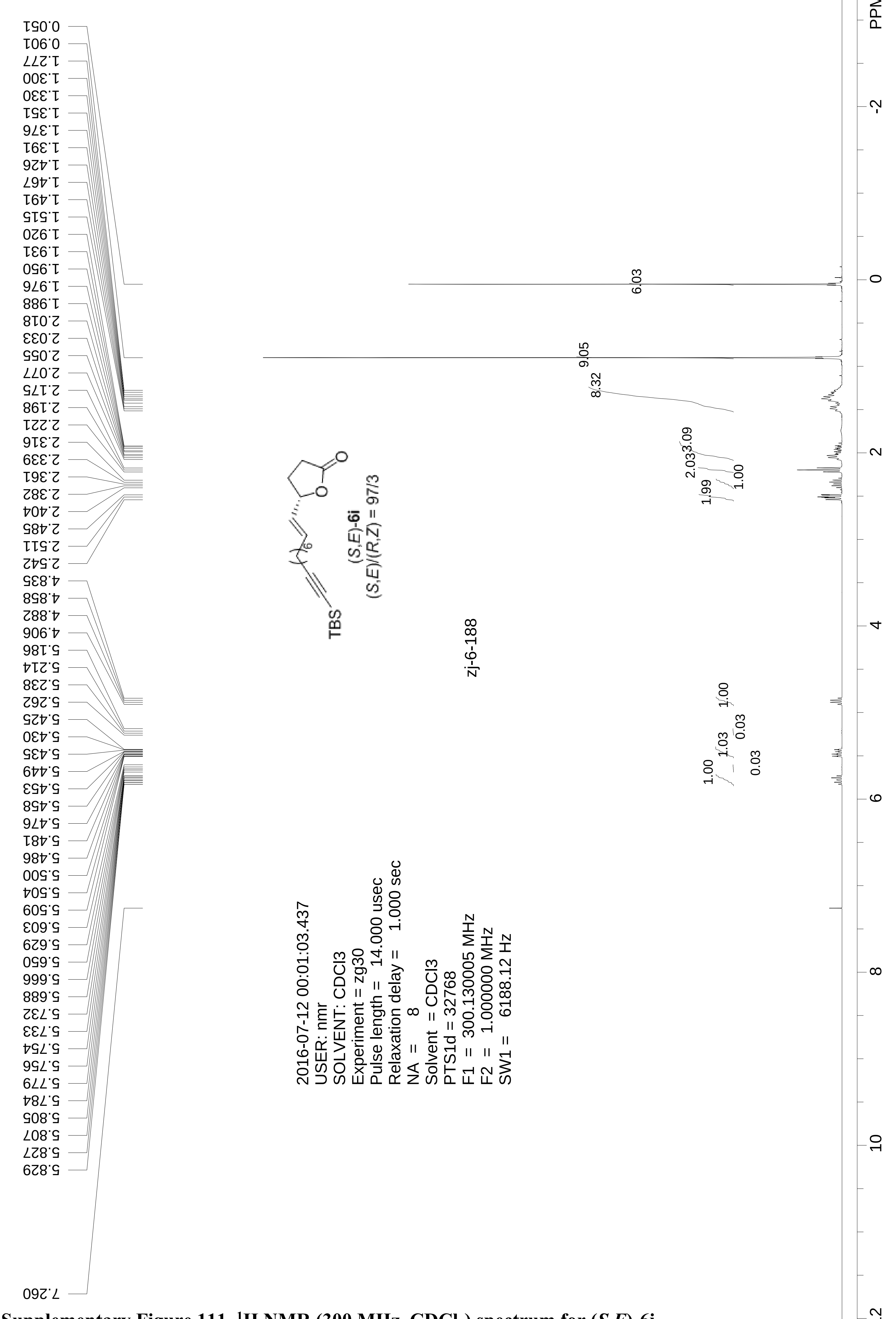

Supplementary Figure 111. ^{1}H NMR (300 MHz, $CDCl_3$) spectrum for (*S*,*E*)-6i.

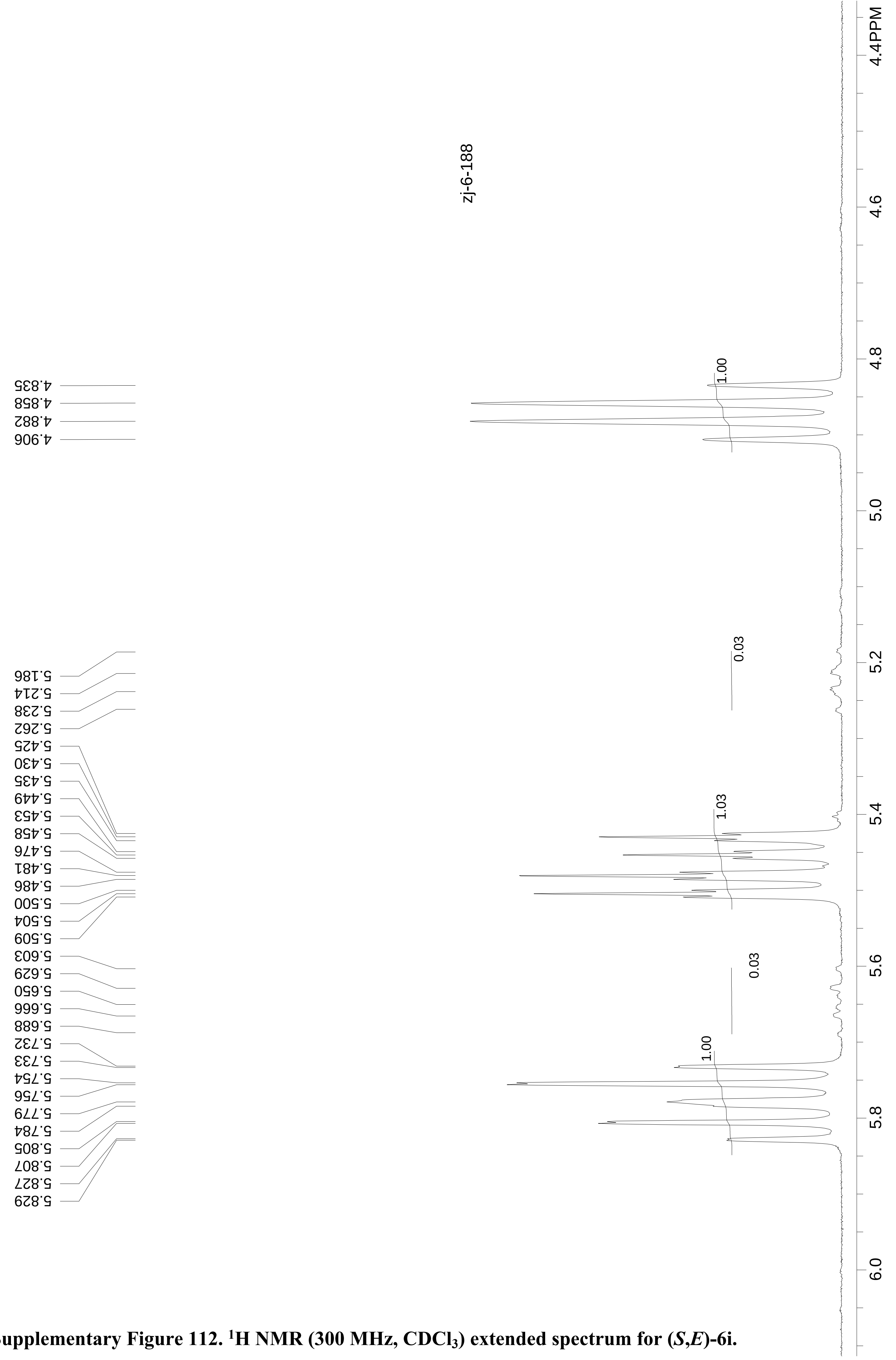

Supplementary Figure 112. ^{1}H NMR (300 MHz, $CDCl_3$) extended spectrum for (*S*,*E*)-6i.

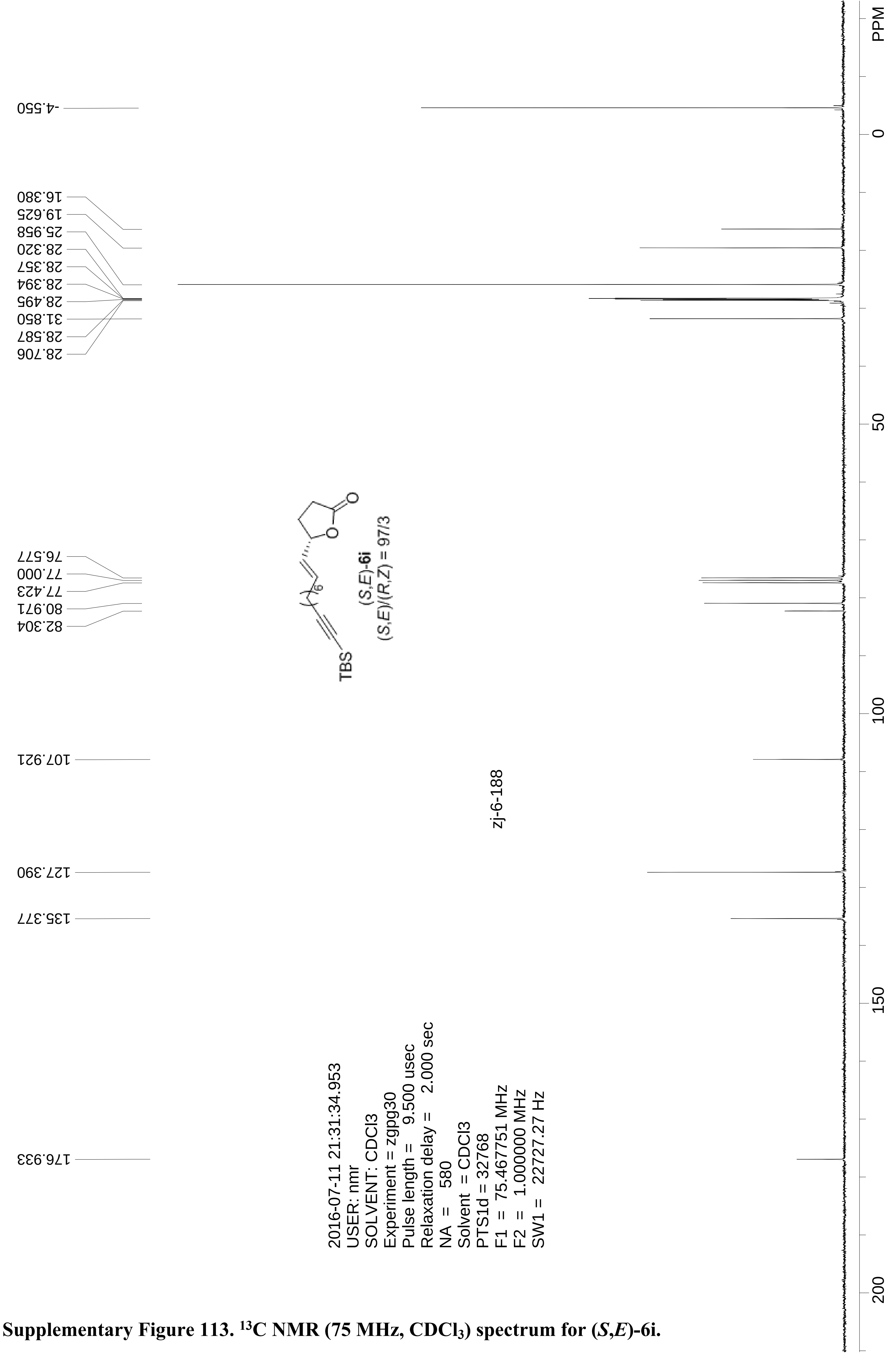

Supplementary Figure 113. ^{13}C NMR (75 MHz, $CDCl_3$) spectrum for (*S*,*E*)-6i.

zj-6-188-oj-h-100-1-0.3-214

实验时间：2016-07-14,12:49:48　　　　报告时间：2016-07-14,17:18:57
谱图文件:D:\zhuguangjiong\zj\20160712\zj-6-188-oj-h-100-1-0.3-214.org

实验内容简介：

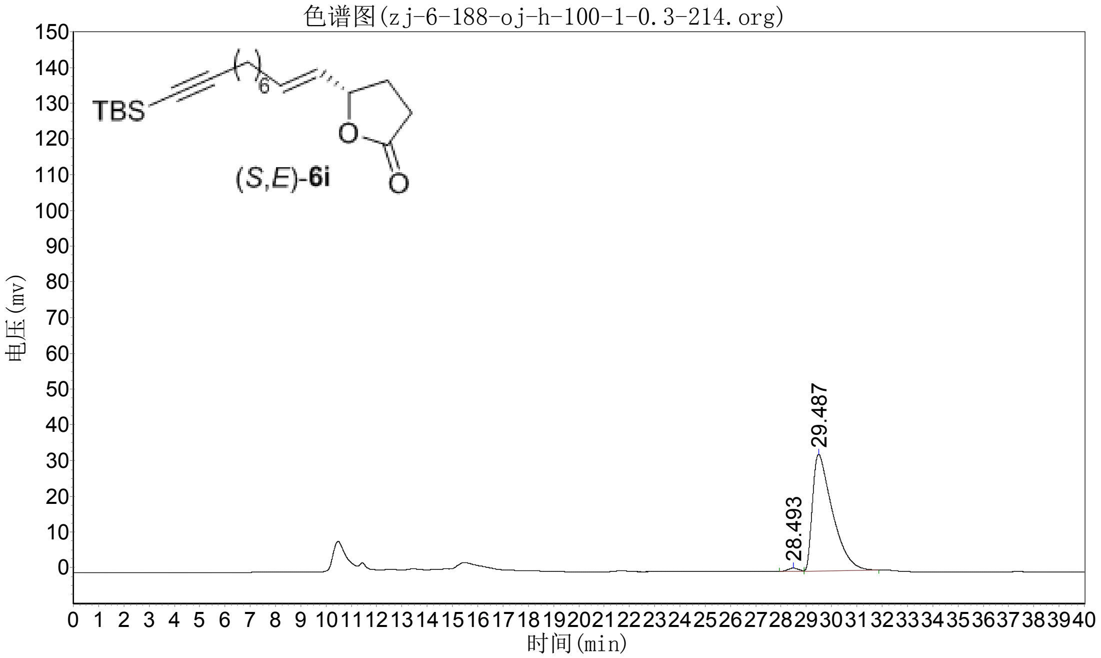

分析结果表

峰号	峰名	保留时间	峰高	峰面积	含量
1		28.493	883.960	26267.666	1.4491
2		29.487	32668.090	1786474.625	98.5509
总计			33552.050	1812742.291	100.0000

Supplementary Figure 114. HPLC spectrum for (*S*,*E*)-6i.

zj-6-182-oj-h-100-1-0.3-214

实验时间：2016-07-14,10:44:07 报告时间：2016-07-14,17:17:55
谱图文件:D:\zhuguangjiong\zj\20160712\zj-6-182-oj-100-1-0.3-214.org

实验内容简介：

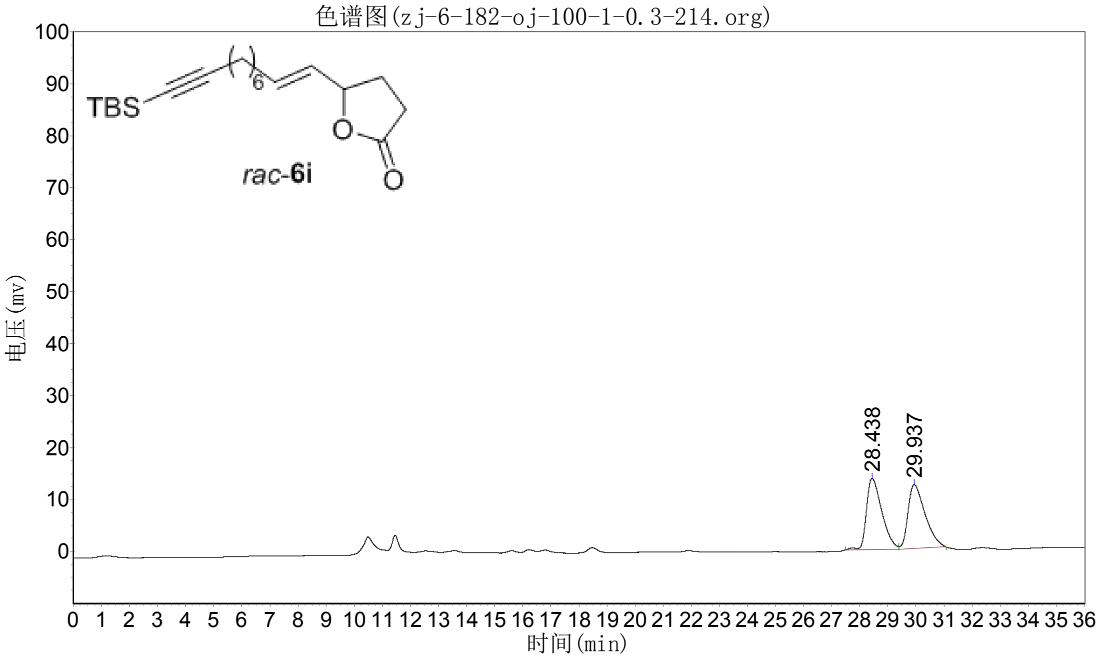

分析结果表

峰号	峰名	保留时间	峰高	峰面积	含量
1		28.438	13724.570	524178.875	50.1513
2		29.937	12299.934	521015.469	49.8487
总计			26024.504	1045194.344	100.0000

Supplementary Figure 115. HPLC spectrum for *rac*-6i.

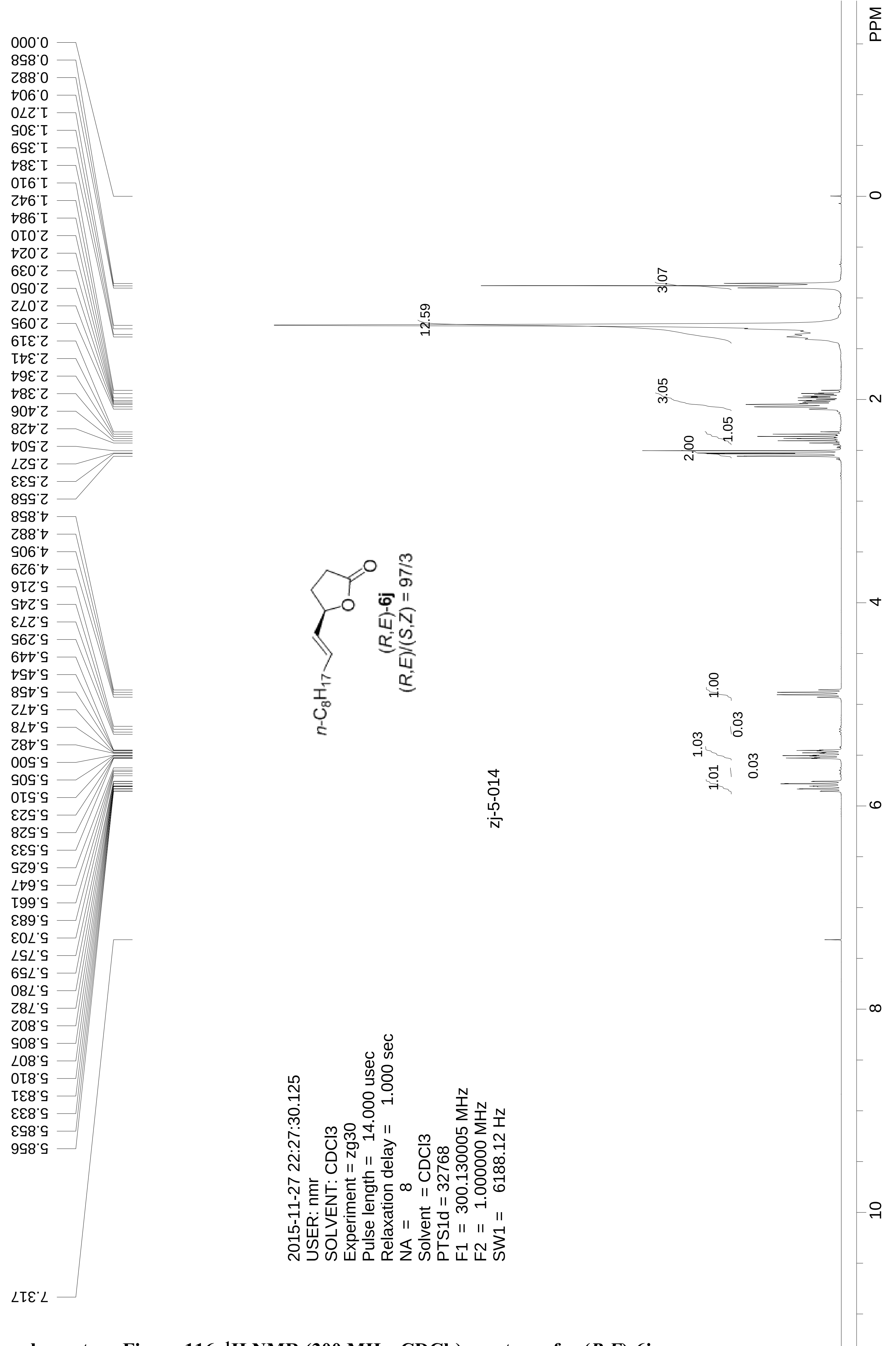

Supplementary Figure 116. ^{1}**H NMR (300 MHz, CDCl$_3$) spectrum for (*R*,*E*)-6j.**

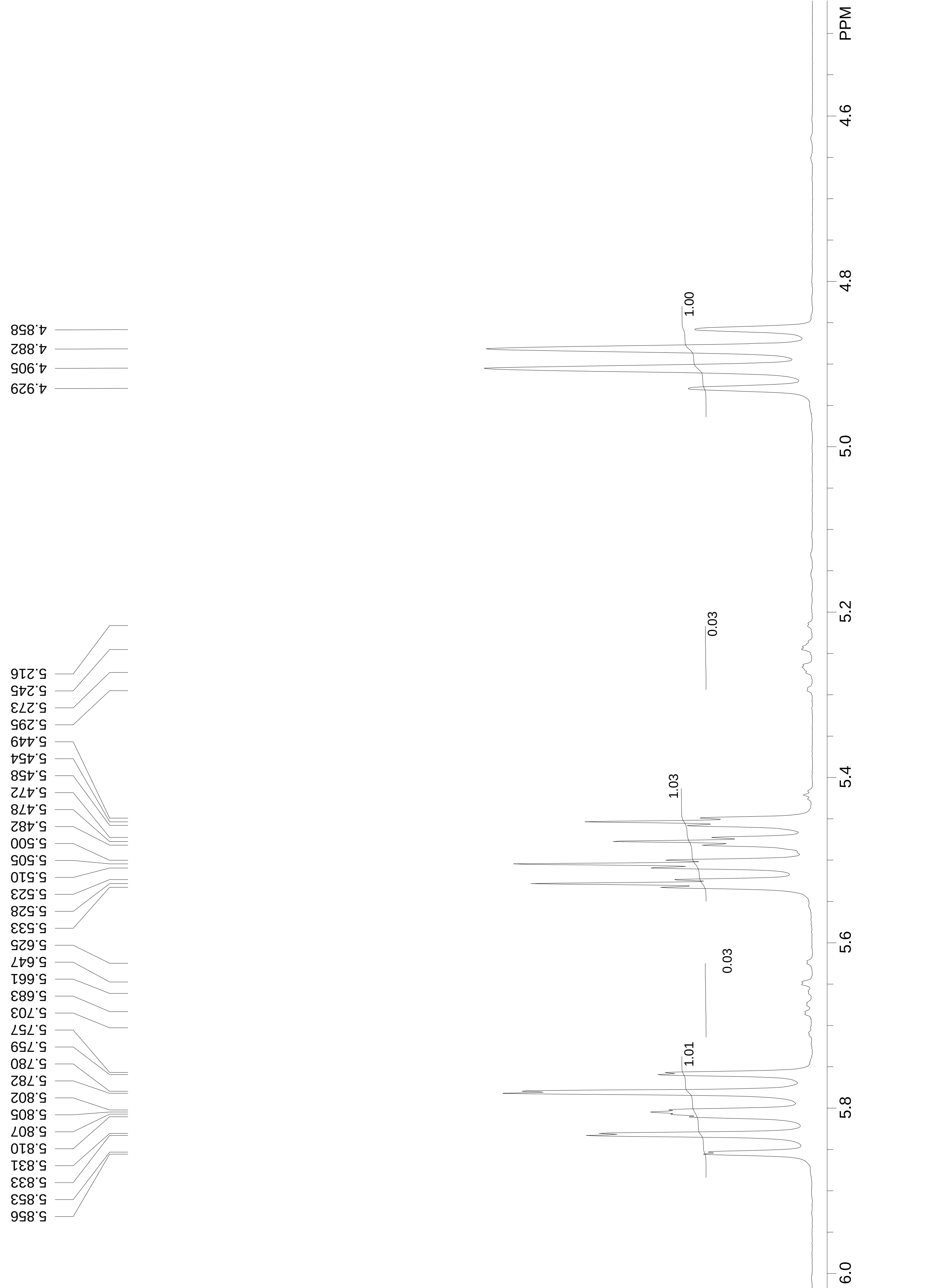

Supplementary Figure 117. ^{1}H NMR (300 MHz, $CDCl_3$) extended spectrum for (*R*,*E*)-6j.

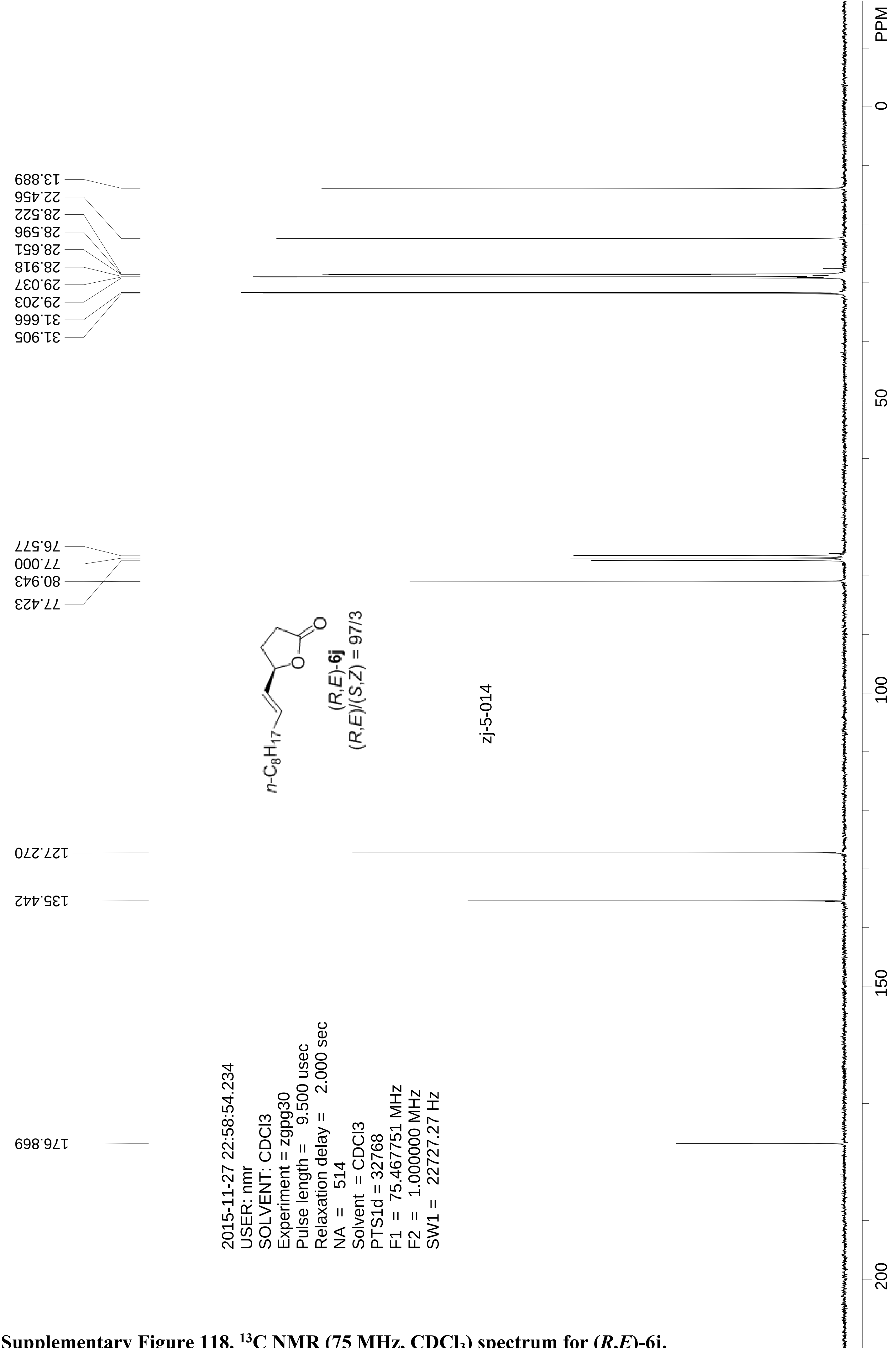

Supplementary Figure 118. ^{13}C NMR (75 MHz, CDCl$_3$) spectrum for (*R*,*E*)-6j.

zj-5-014-oj-h-100-1-1-214

实验时间：2016/1/7,15:21:56　　　　报告时间：2016/1/7,17:02:24
谱图文件:d:\zhuguangjiong\zj\20160106\zj-5-014-oj-100-1-1-214..org

实验内容简介：

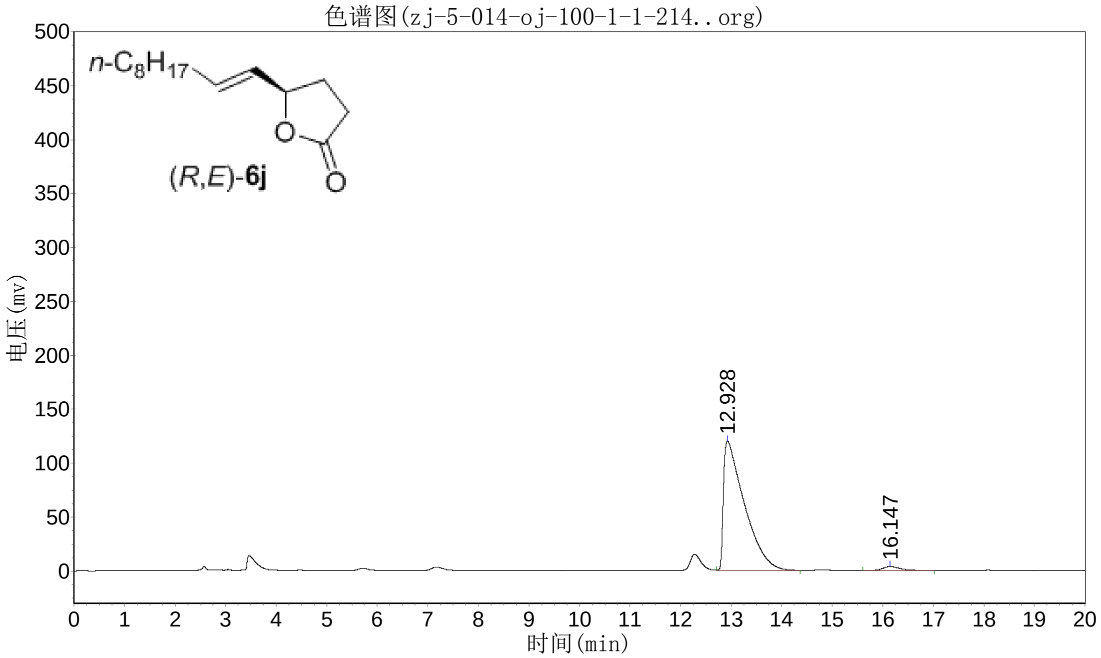

分析结果表

峰号	峰名	保留时间	峰高	峰面积	含量
1		12.928	120117.664	3456854.500	97.5368
2		16.147	3762.432	87297.750	2.4631
总计			123880.096	3544152.250	100.0000

Supplementary Figure 119. HPLC spectrum for (*R*,*E*)-6j.

zj-5-013-oj-h-100-1-1-214

实验时间：2016/1/7,13:38:12　　　　报告时间：2016/1/7,17:01:04
谱图文件:d:\zhuguangjiong\zj\20160106\zj-5-013-oj-100-1-1-214.org

实验内容简介：

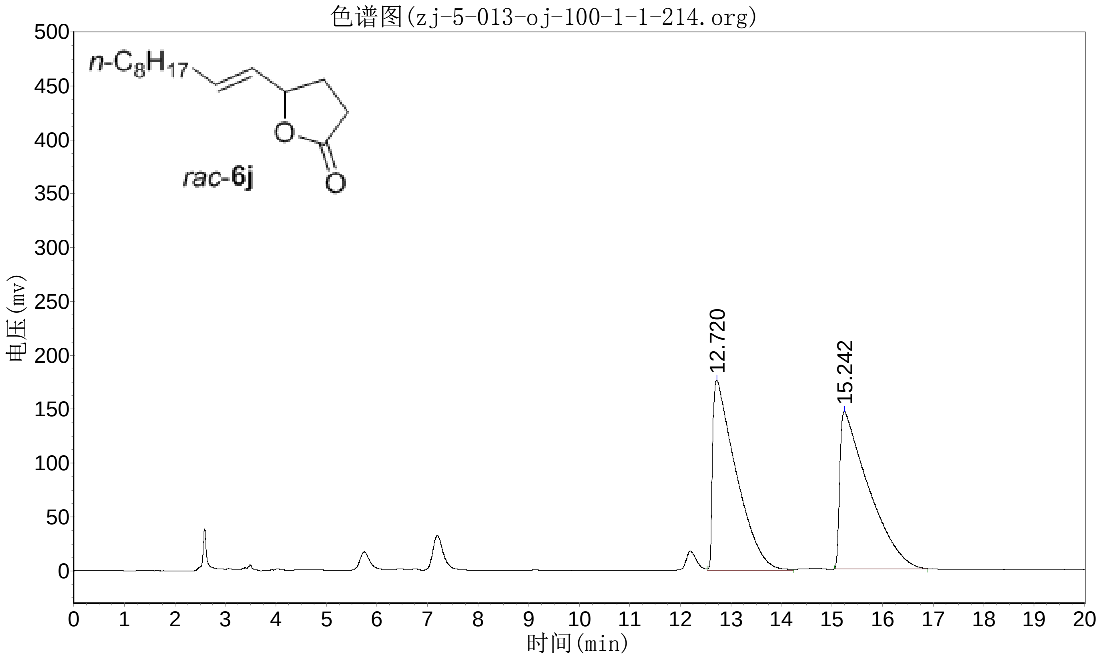

分析结果表

峰号	峰名	保留时间	峰高	峰面积	含量
1		12.720	176572.141	5660659.000	49.5398
2		15.242	146212.047	5765820.000	50.4602
总计			322784.188	11426479.000	100.0000

Supplementary Figure 120. HPLC spectrum for *rac*-6j.

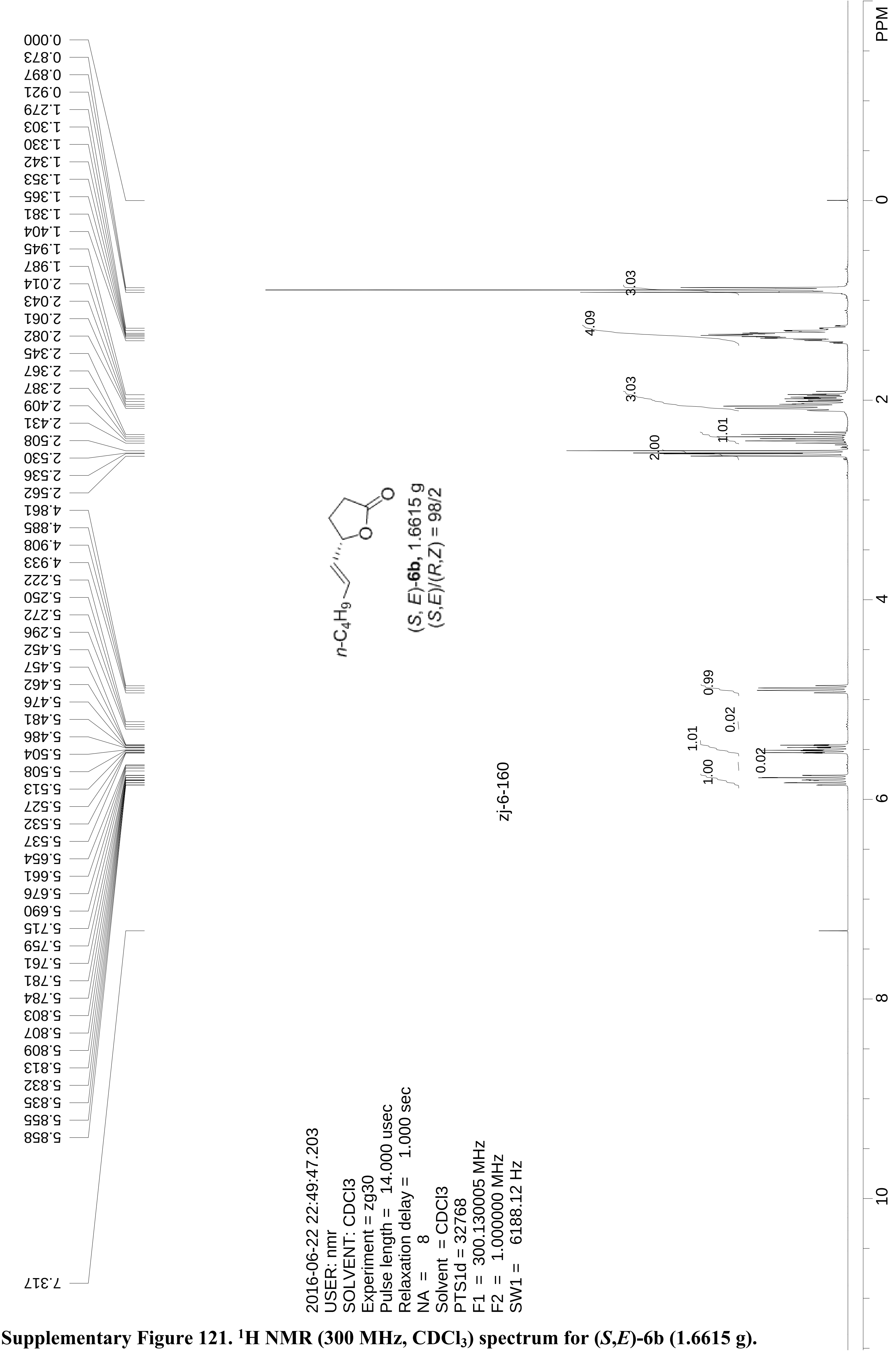

Supplementary Figure 121. ^{1}H NMR (300 MHz, $CDCl_3$) spectrum for (*S*,*E*)-6b (1.6615 g).

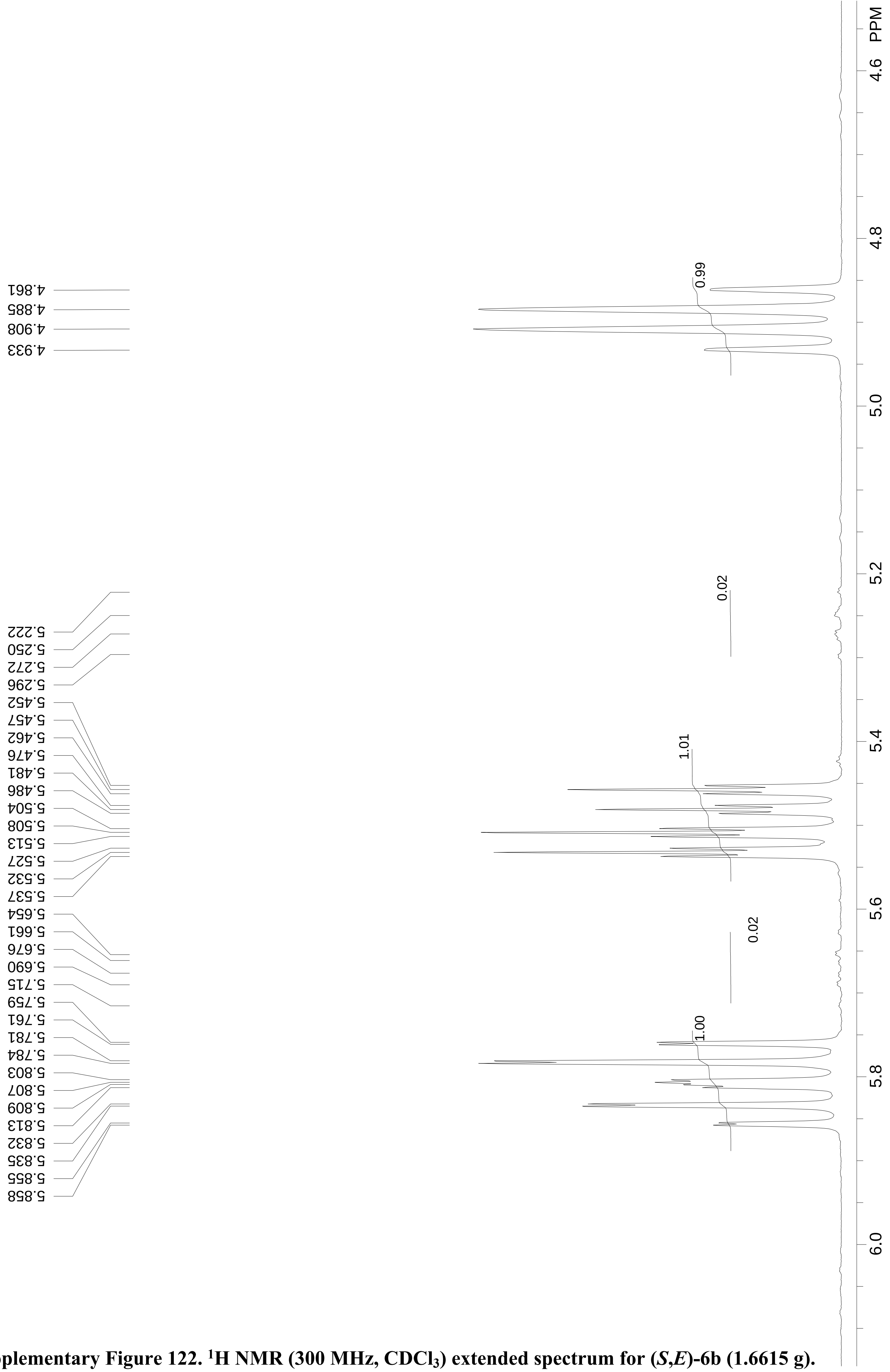

Supplementary Figure 122. ^{1}H NMR (300 MHz, $CDCl_3$) extended spectrum for (*S*,*E*)-6b (1.6615 g).

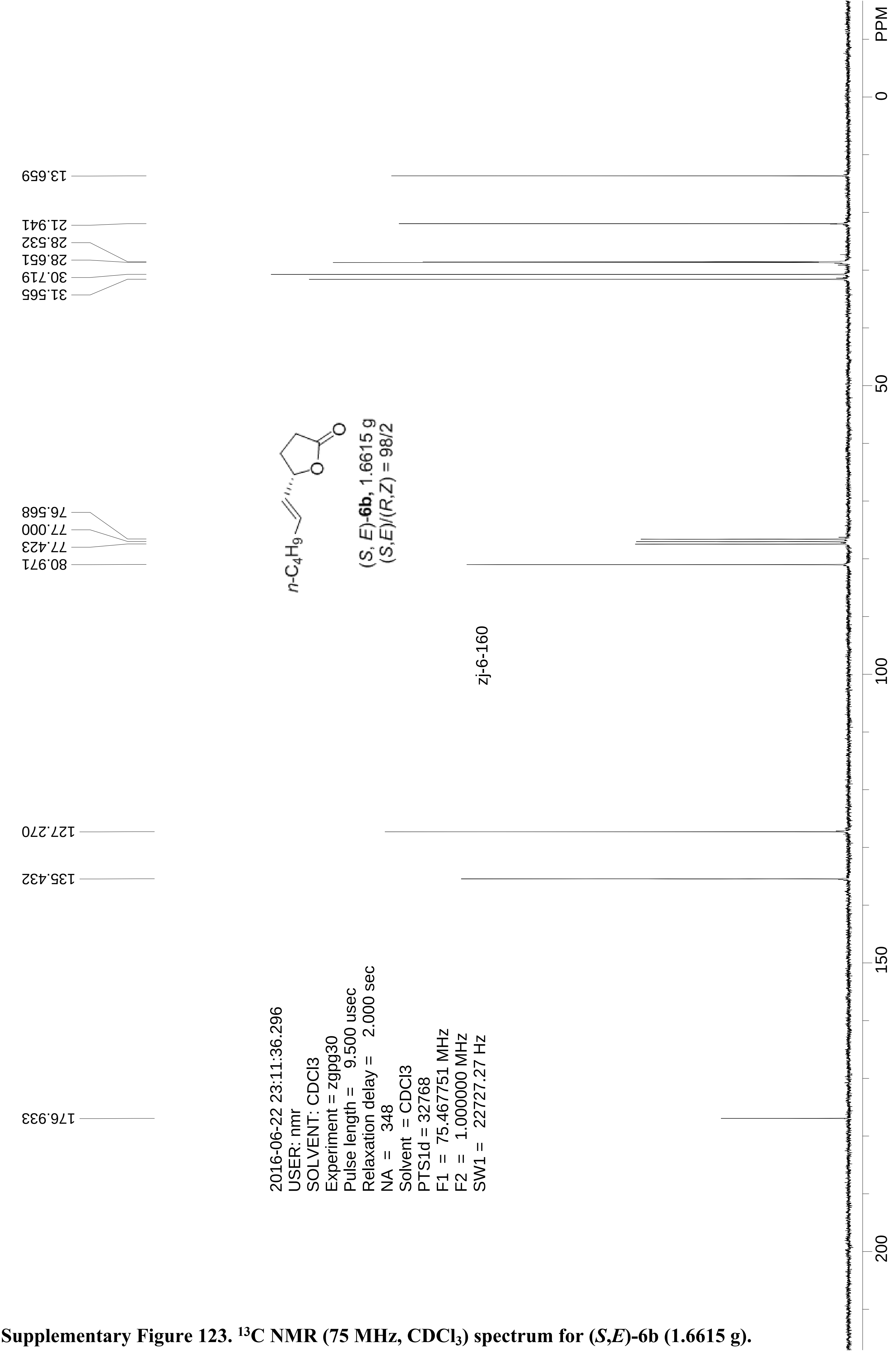

Supplementary Figure 123. ^{13}**C NMR (75 MHz, CDCl$_3$) spectrum for (*S*,*E*)-6b (1.6615 g).**

zj-6-160-oj-h-200-1-1-214

实验时间：2016-07-01,10:35:50 报告时间：2016-07-01,12:50:43
谱图文件:D:\zhuguangjiong\zj\20160630\ZJ-6-160-oj-h-200-1-1-214.org

实验内容简介：

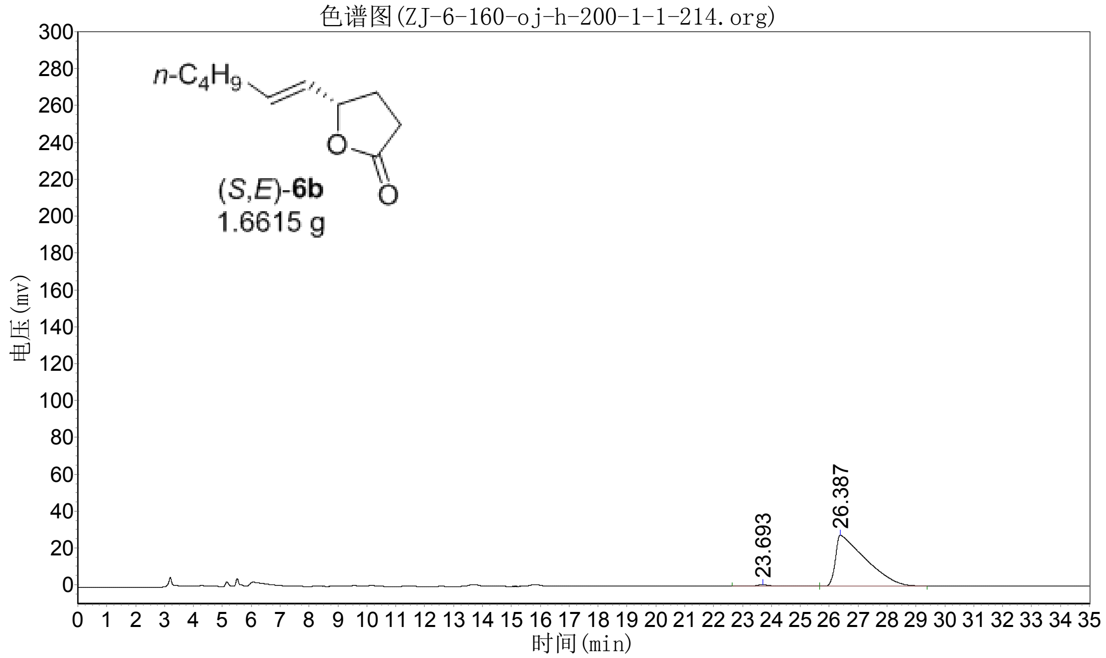

分析结果表

峰号	峰名	保留时间	峰高	峰面积	含量
1		23.693	844.168	31765.662	1.5876
2		26.387	27539.016	1969061.375	98.4124
总计			28383.184	2000827.037	100.0000

Supplementary Figure 124. HPLC spectrum for (*S*,*E*)-6b (1.6615 g).

zj-6-056-oj-h-200-1-1-214

实验时间：2016-07-01,12:14:25　　　　　　　　　　　　报告时间：2016-07-01,12:49:09
谱图文件:D:\zhuguangjiong\zj\20160630\ZJ-6-056-oj-h-200-1-1-214..org

实验内容简介：

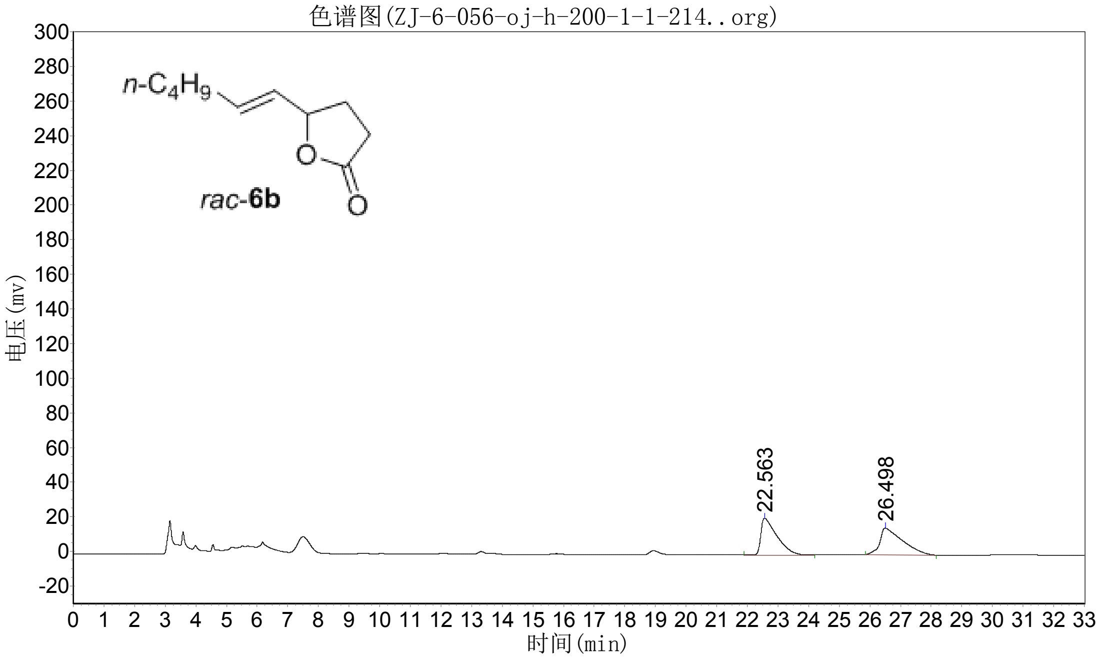

分析结果表

峰号	峰名	保留时间	峰高	峰面积	含量
1		22.563	21248.332	782160.188	49.8864
2		26.498	15297.864	785722.813	50.1136
总计			36546.196	1567883.000	100.0000

Supplementary Figure 125. HPLC spectrum for *rac*-6b.

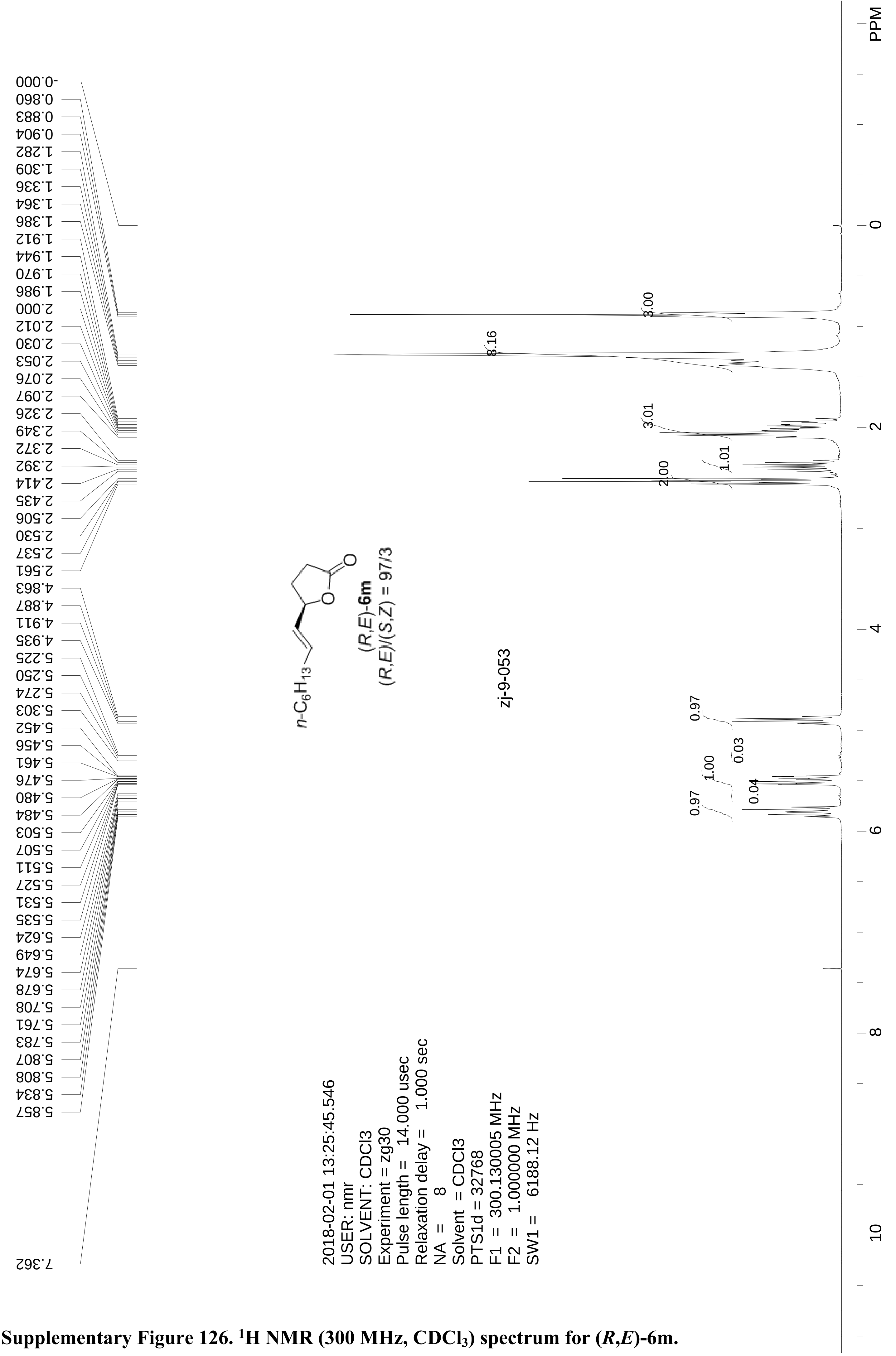

Supplementary Figure 126. ^{1}**H NMR (300 MHz,** $CDCl_3$**) spectrum for (*R*,*E*)-6m.**

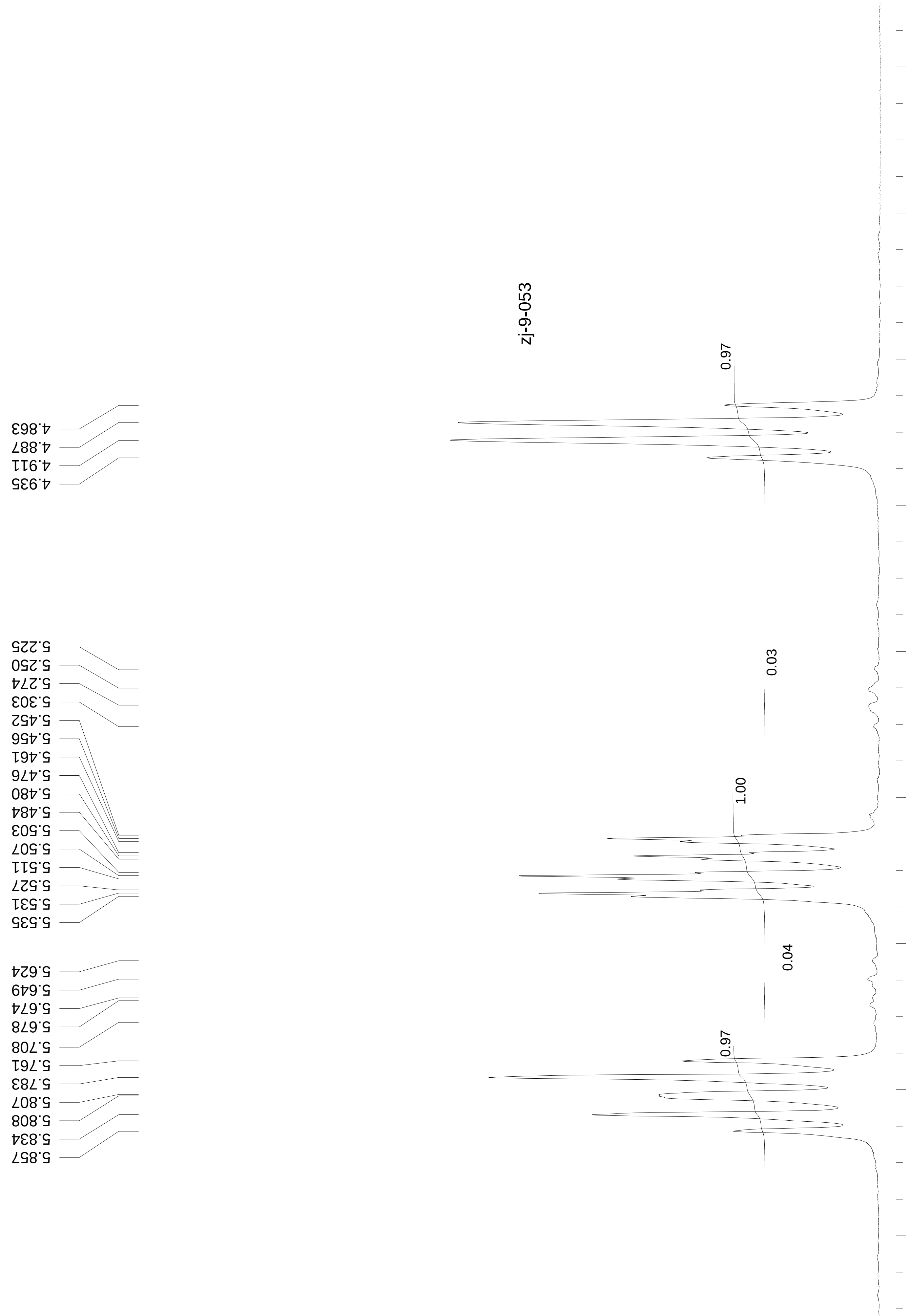

Supplementary Figure 127. ^{1}H NMR (300 MHz, $CDCl_3$) extended spectrum for (*R*,*E*)-6m.

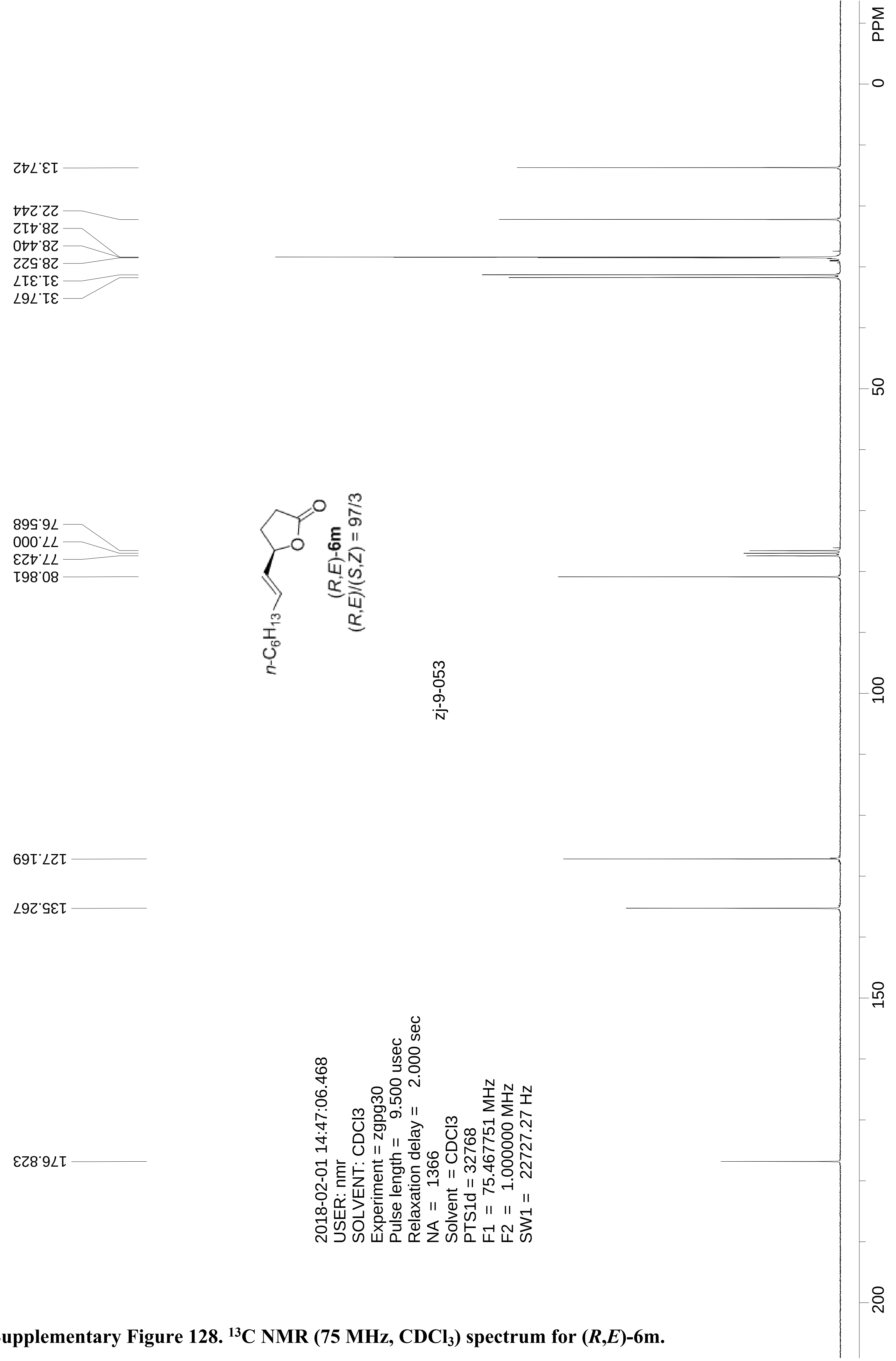

Supplementary Figure 128. ^{13}C NMR (75 MHz, $CDCl_3$) spectrum for (*R*,*E*)-6m.

zj-9-053-oj-h-97-3-1-214

实验时间：2018-02-02,18:50:03　　　　　　　　　　报告时间：2018-02-02,19:10:10
谱图文件:D:\zhuguangjiong\zj\20180202\zj-9-053-oj-h-97-3-1-214..org

实验内容简介：

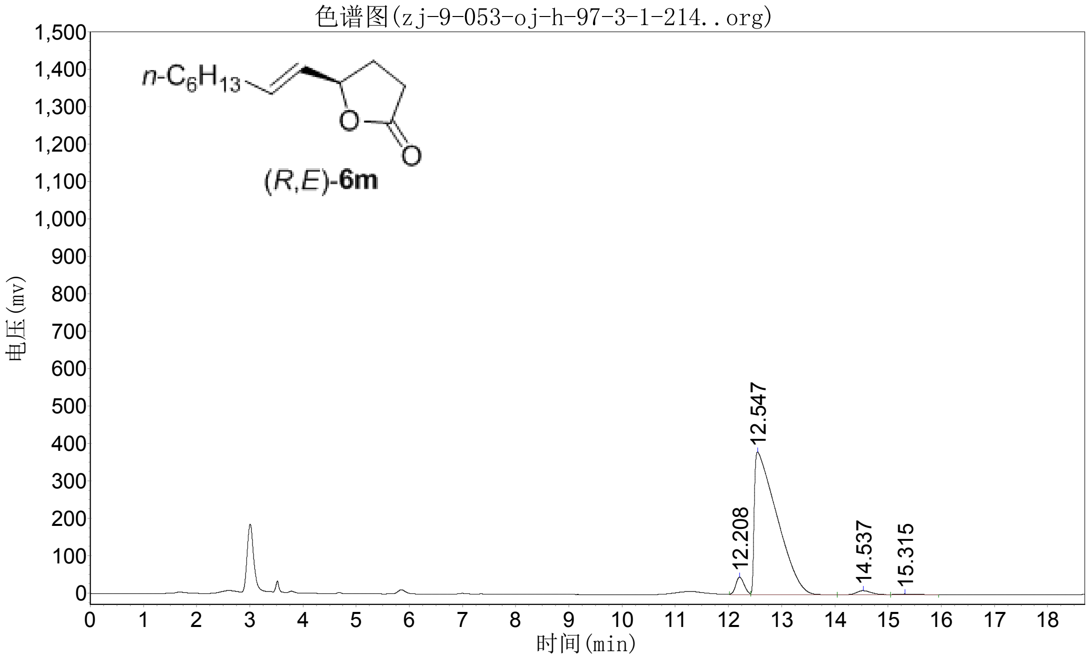

分析结果表

峰号	峰名	保留时间	峰高	峰面积	含量
1		12.208	46765.355	516480.156	4.2844
2		12.547	381413.406	11254235.000	93.3573
3		14.537	10995.621	219209.844	1.8184
4		15.315	2304.944	65094.563	0.5400
总计			441479.326	12055019.563	100.0000

Supplementary Figure 129. HPLC spectrum for (*R*,*E*)-6m.

zj-9-054-oj-h-97-3-1-214

实验时间：2018-02-02,18:12:35　　　　　　　　　　报告时间：2018-02-02,18:40:58
谱图文件:D:\zhuguangjiong\zj\20180202\zj-9-054-oj-h-97-3-1-214..org

实验内容简介：

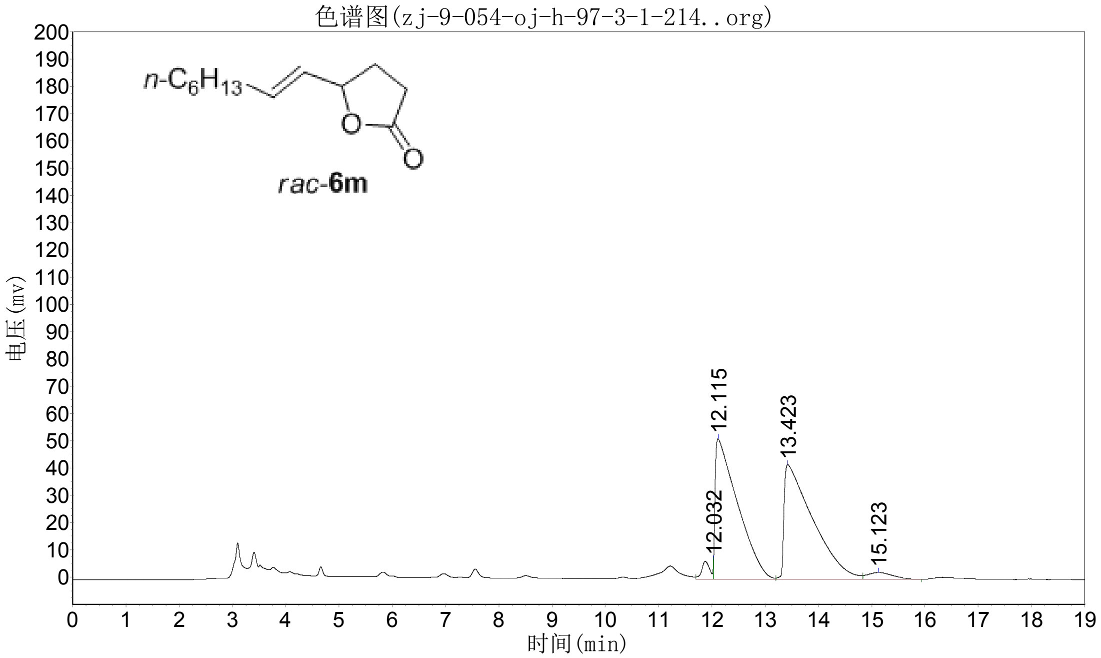

分析结果表

峰号	峰名	保留时间	峰高	峰面积	含量
1		12.032	6901.926	69434.695	2.1527
2		12.115	51452.254	1499887.000	46.5016
3		13.423	41955.156	1575010.000	48.8306
4		15.123	2490.805	81123.406	2.5151
总计			102800.141	3225455.102	100.0000

Supplementary Figure 130. HPLC spectrum for *rac*-6m.

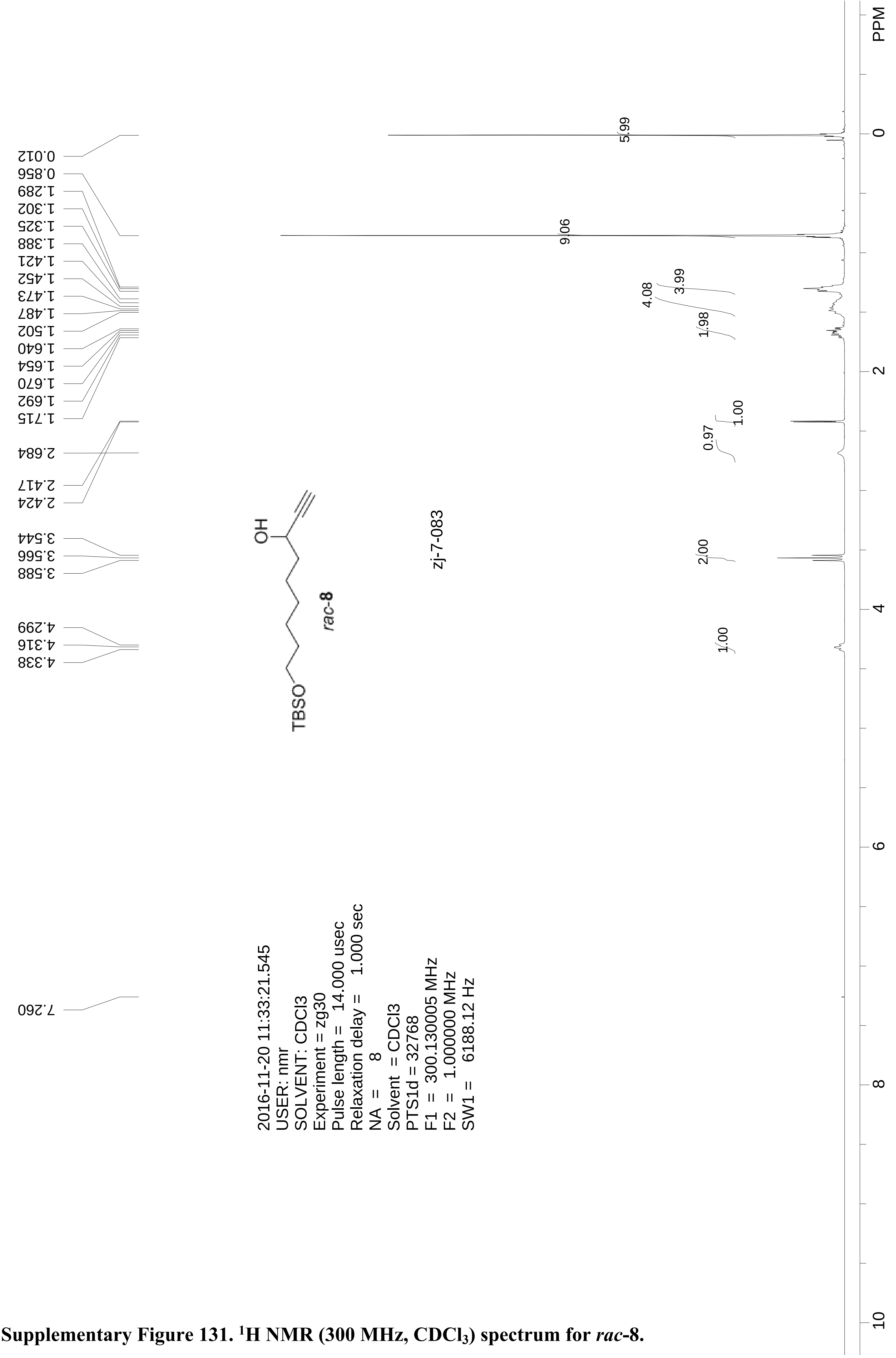

Supplementary Figure 131. ^{1}H NMR (300 MHz, $CDCl_3$) spectrum for *rac*-8.

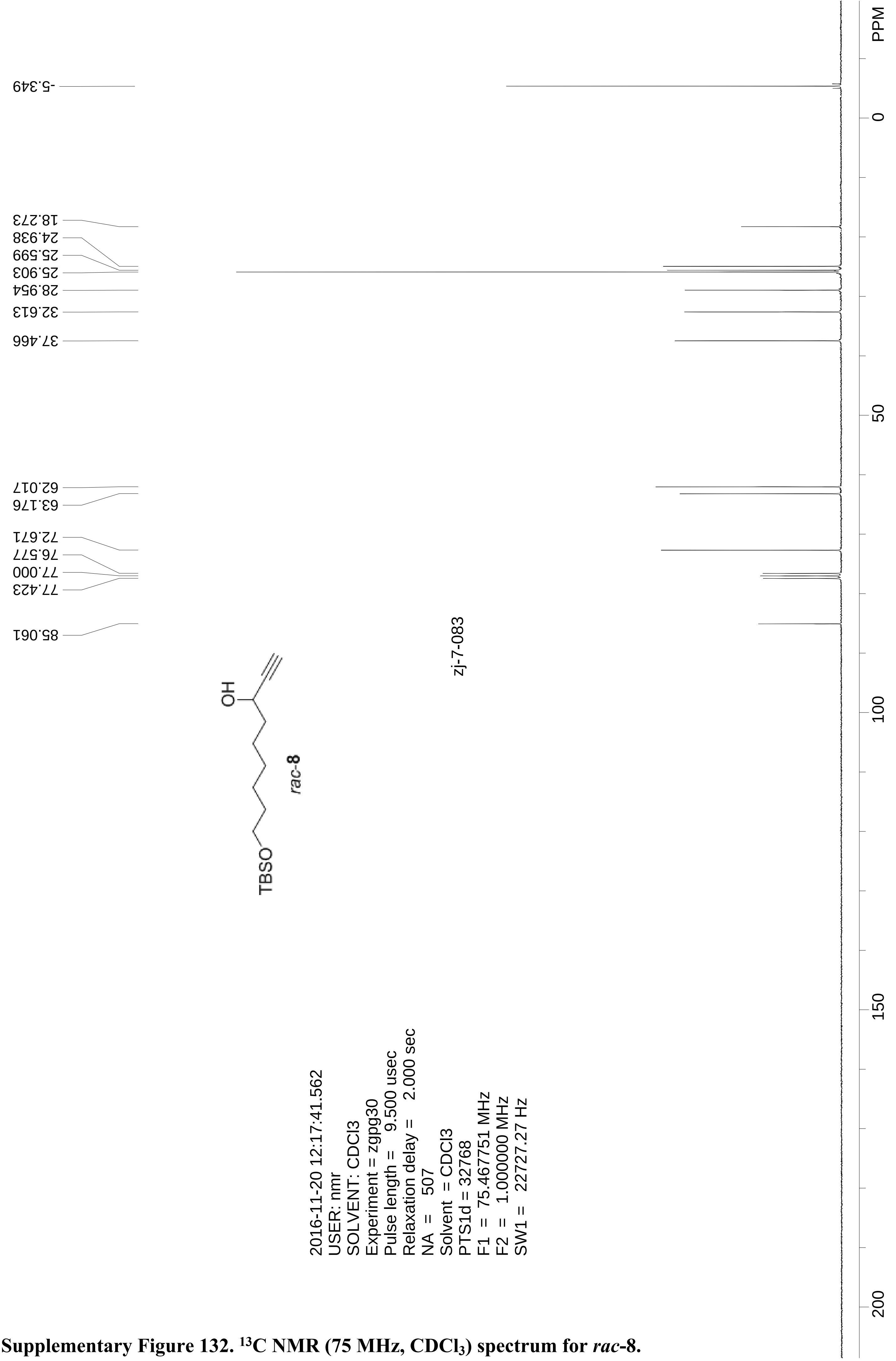

Supplementary Figure 132. ^{13}C NMR (75 MHz, CDCl$_3$) spectrum for *rac*-8.

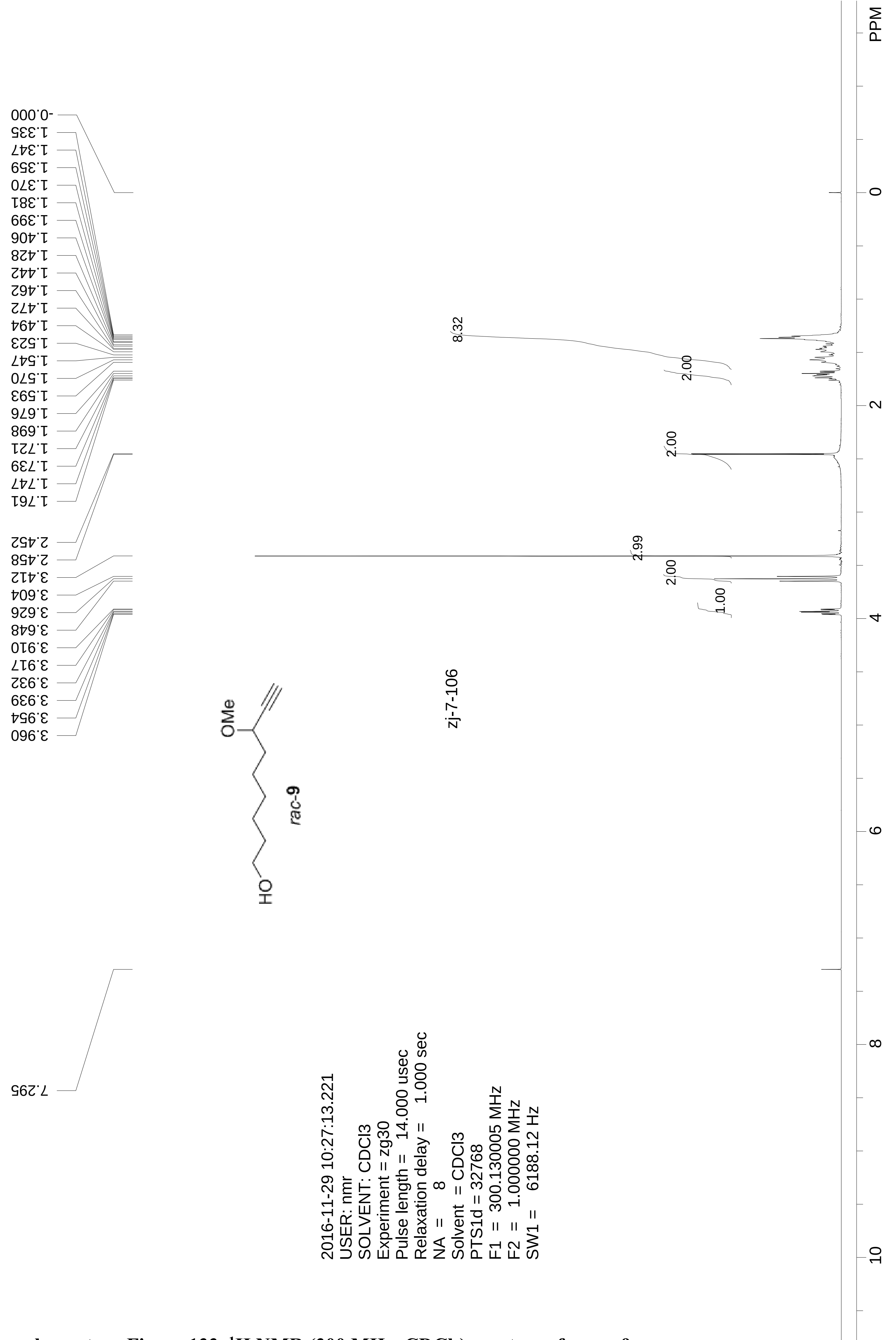

Supplementary Figure 133. ^{1}H NMR (300 MHz, $CDCl_3$) spectrum for *rac*-9.

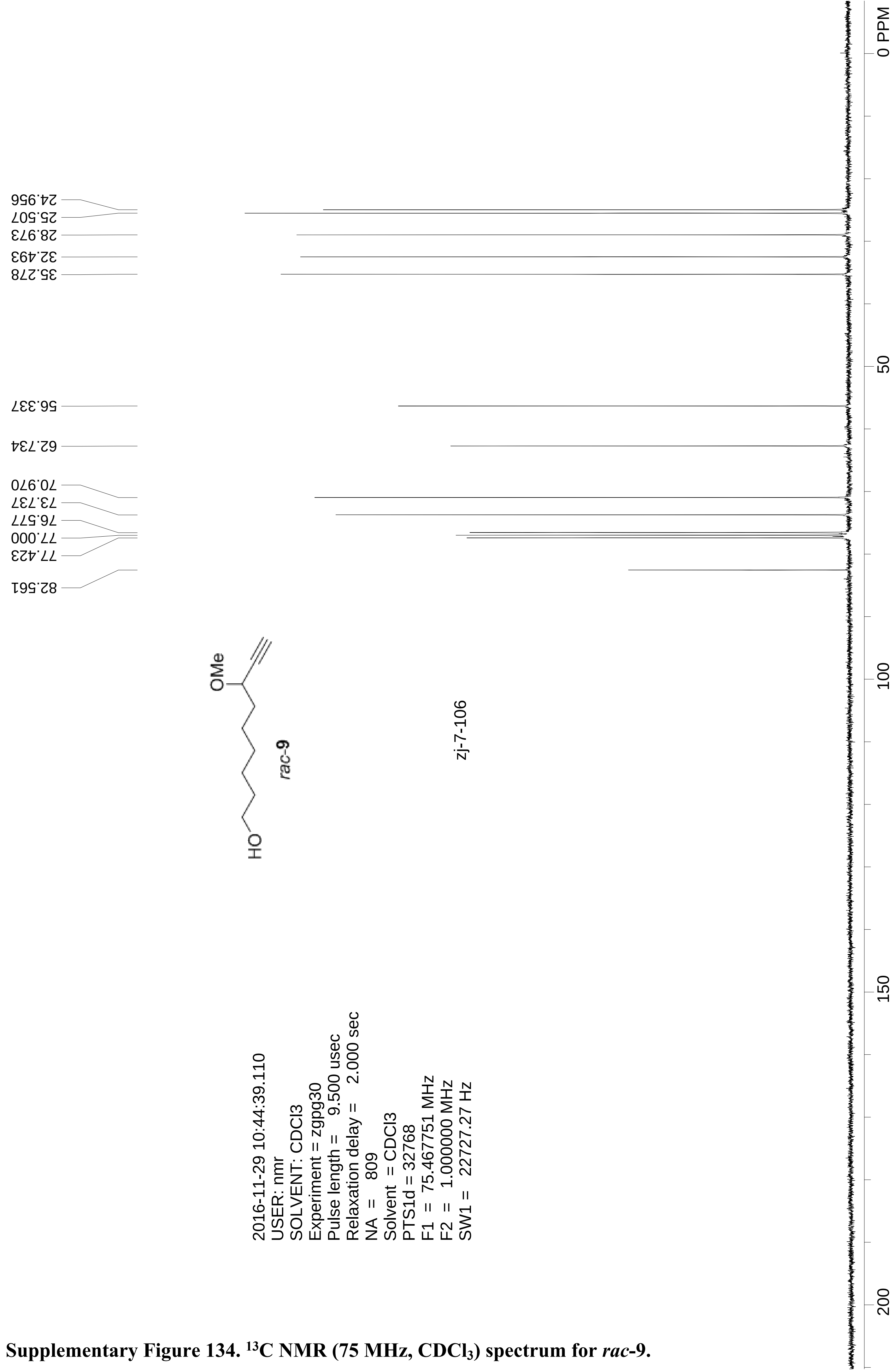

Supplementary Figure 134. ^{13}C NMR (75 MHz, $CDCl_3$) spectrum for *rac*-9.

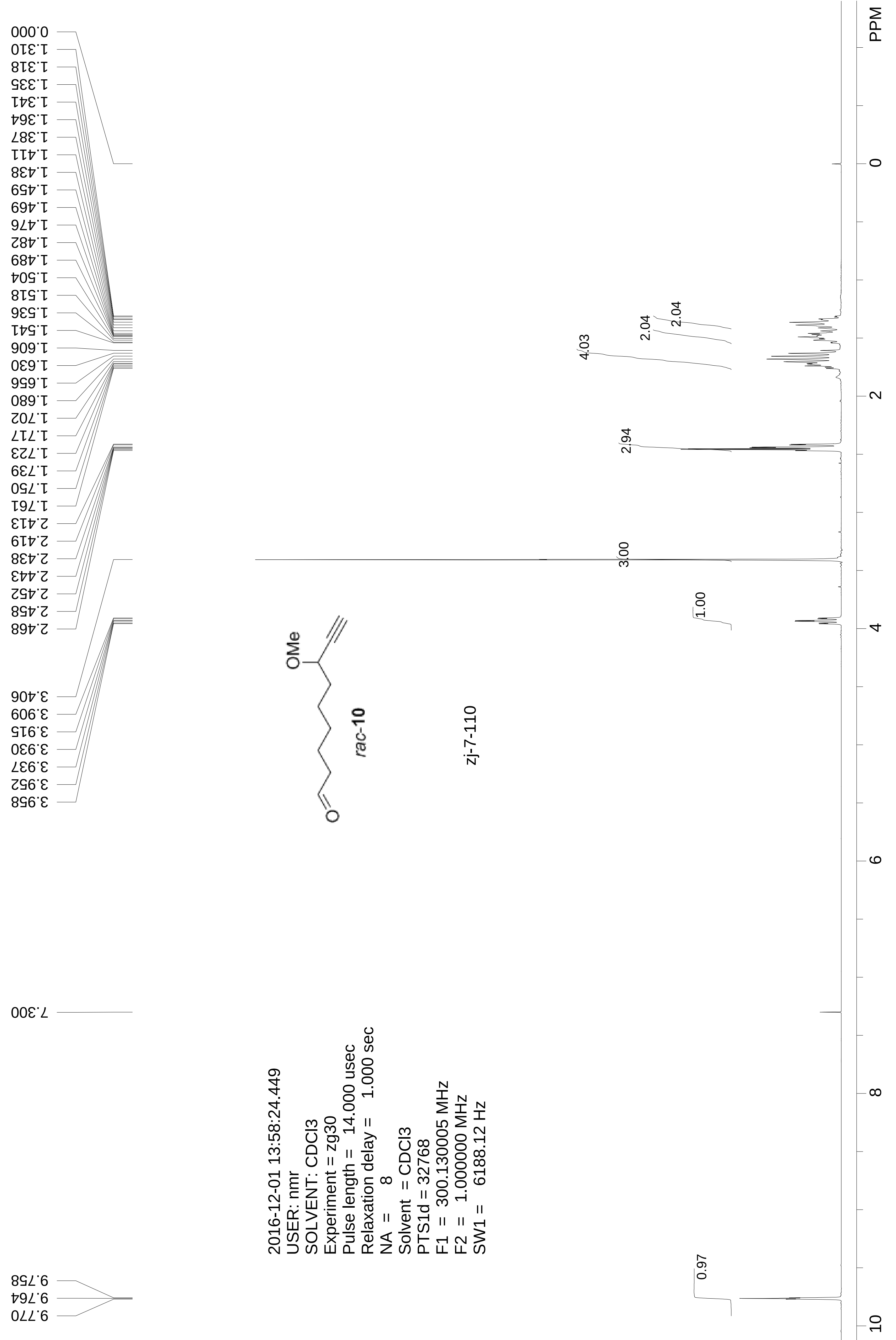

Supplementary Figure 135. ^{1}H NMR (300 MHz, $CDCl_3$) spectrum for *rac*-10.

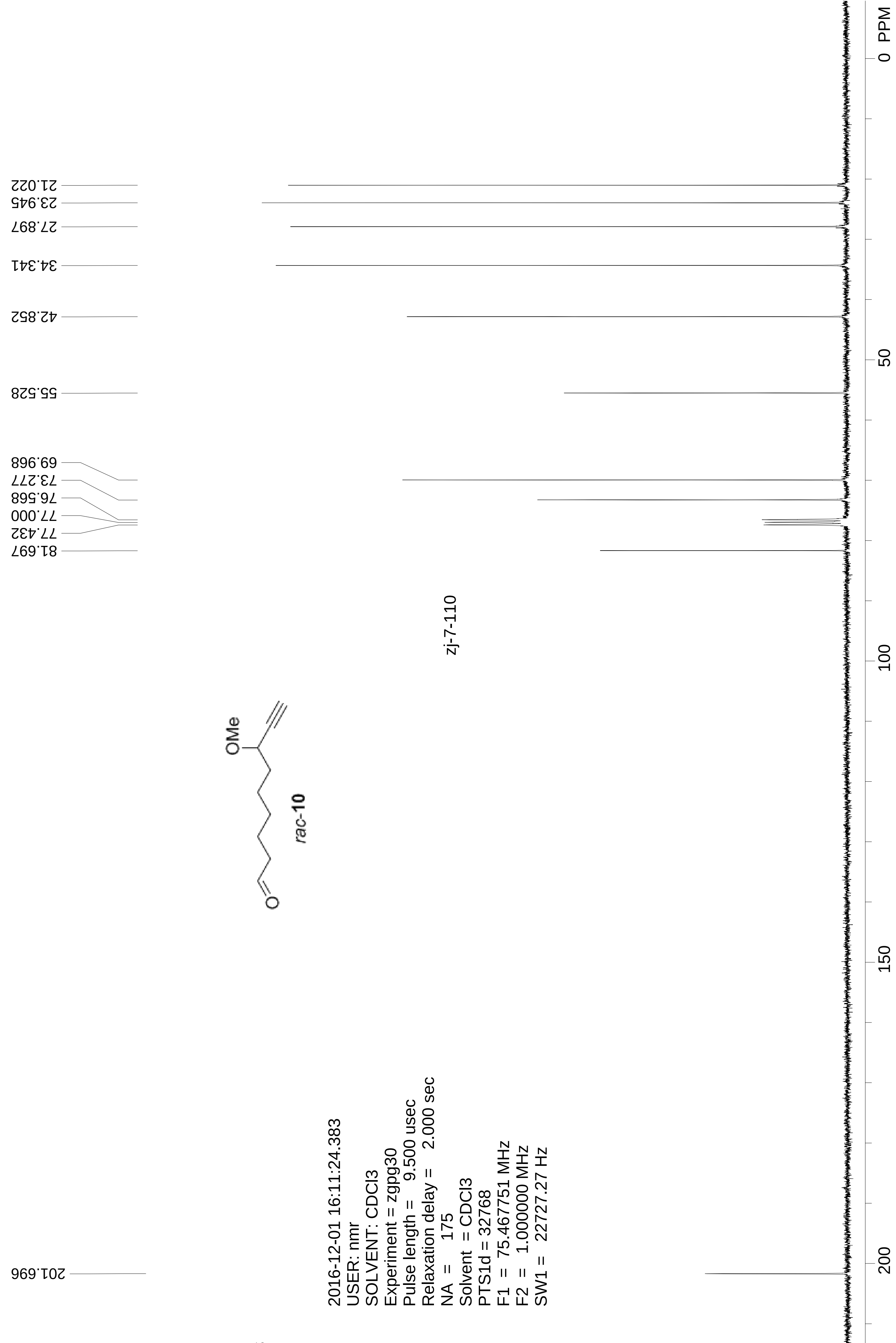

Supplementary Figure 136. ^{13}C NMR (75 MHz, $CDCl_3$) spectrum for *rac*-10.

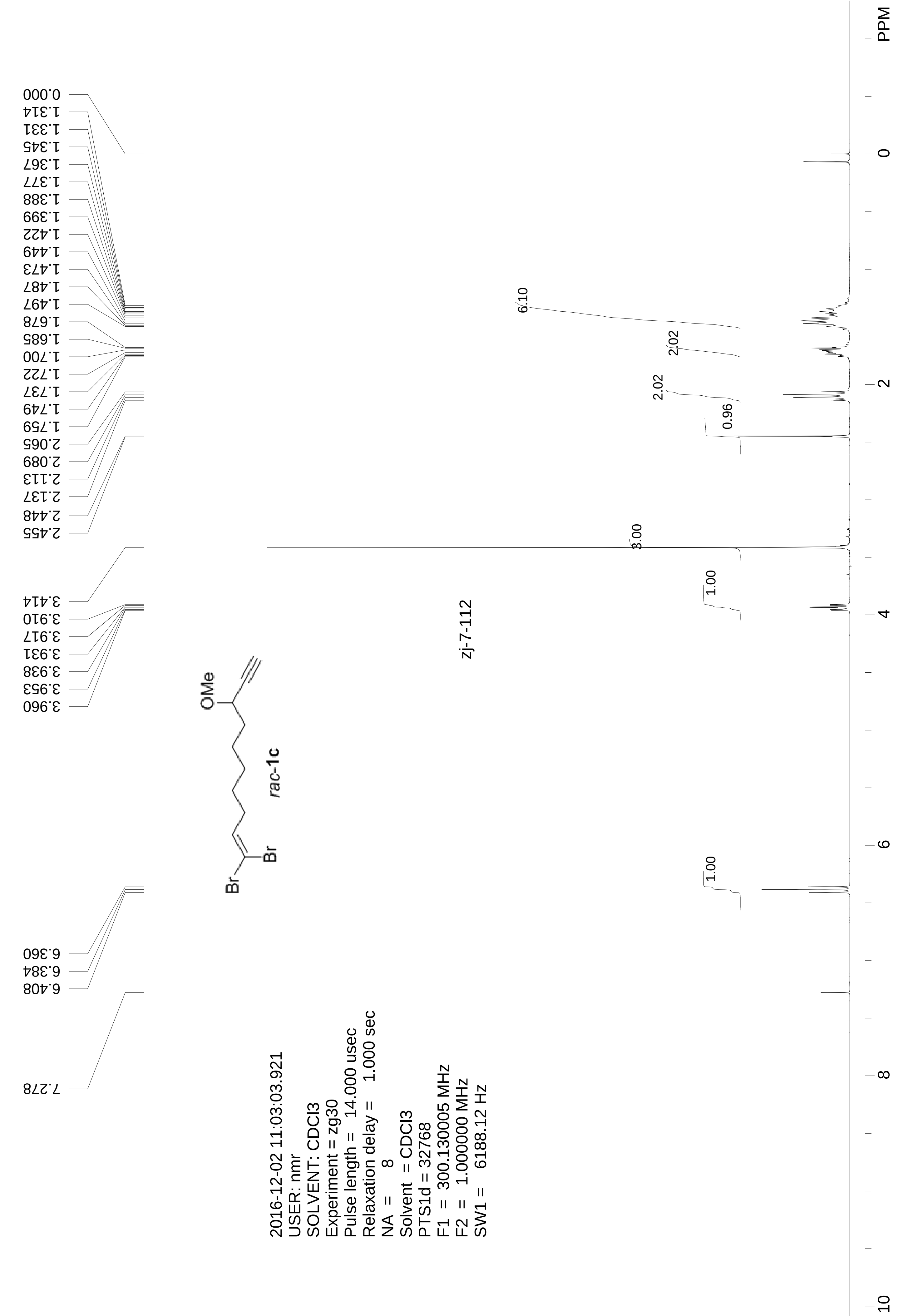

Supplementary Figure 137. ^{1}H NMR (300 MHz, $CDCl_3$) spectrum for *rac*-1c.

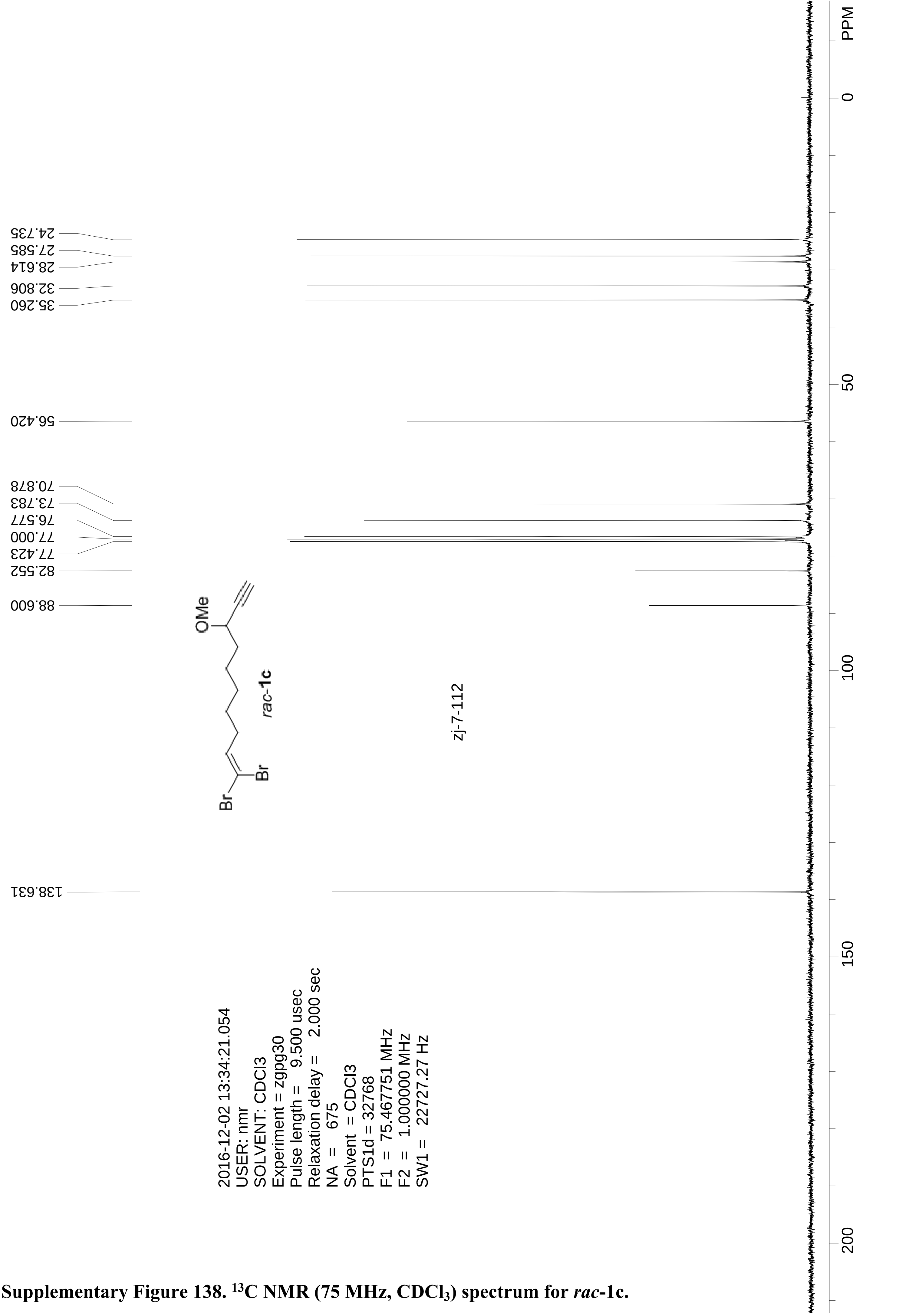

Supplementary Figure 138. ^{13}**C NMR (75 MHz, CDCl$_3$) spectrum for** ***rac*****-1c.**

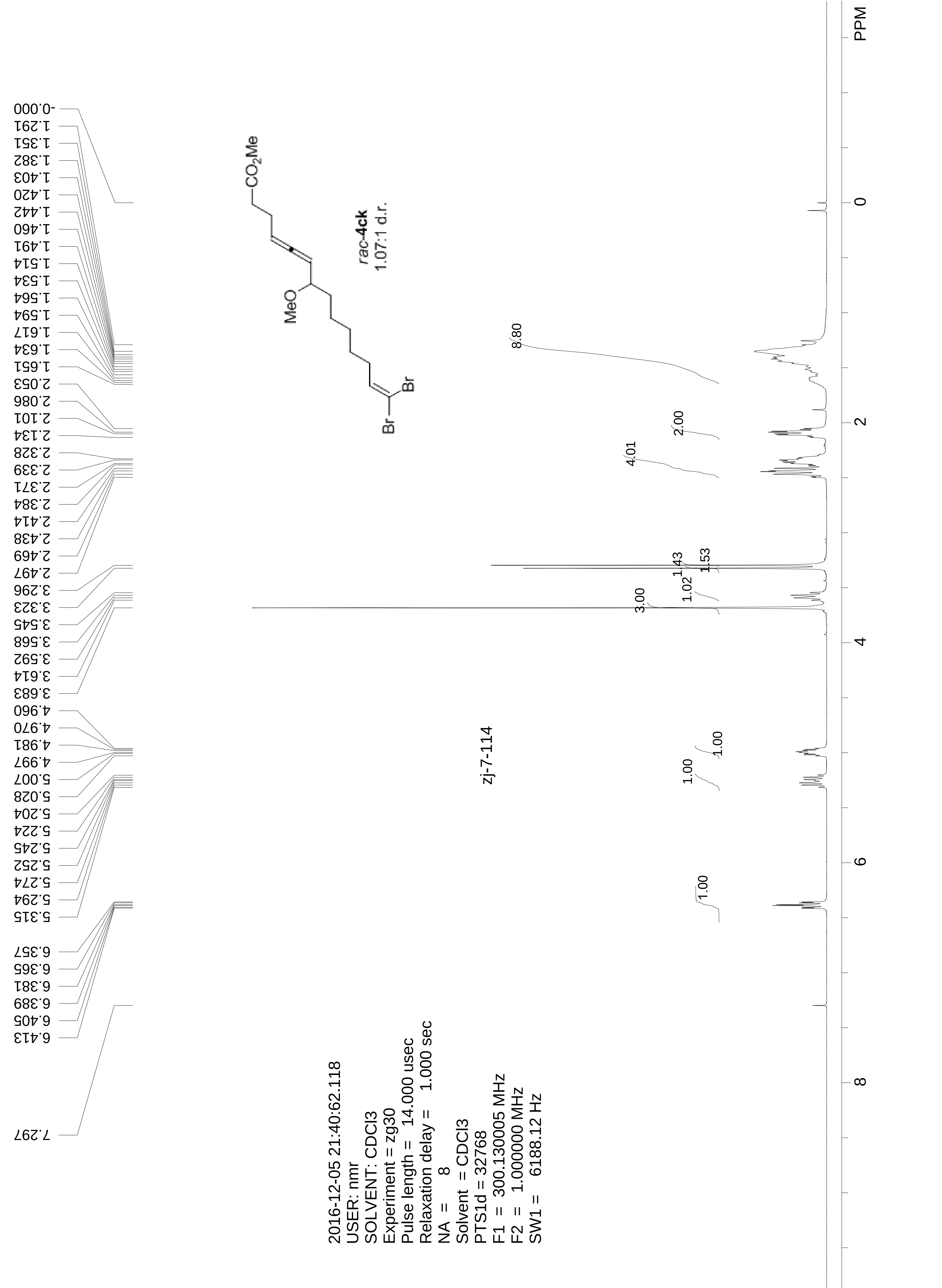

Supplementary Figure 139. ^{1}H NMR (300 MHz, $CDCl_3$) spectrum for *rac*-4ck.

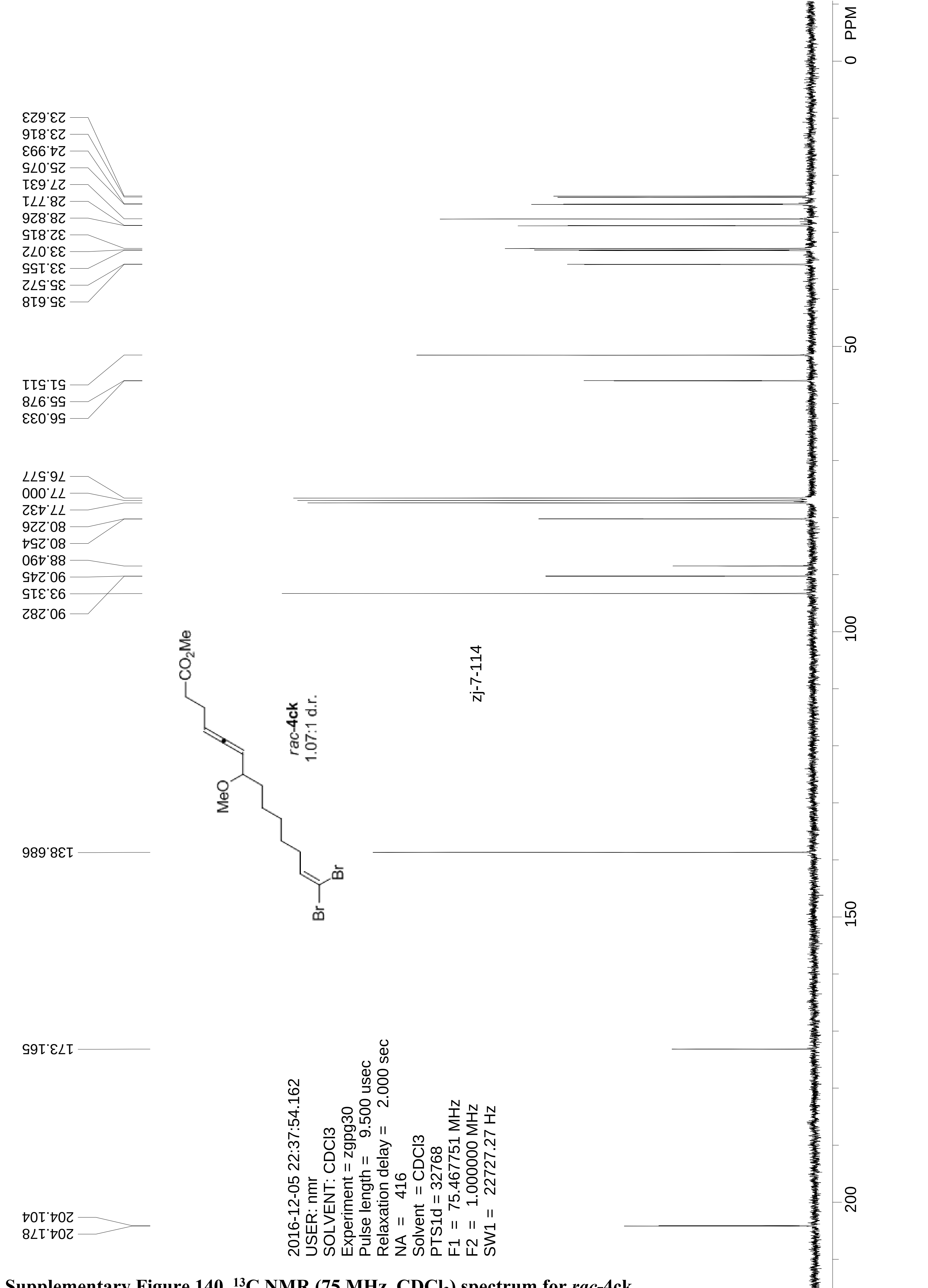

Supplementary Figure 140. ^{13}C NMR (75 MHz, $CDCl_3$) spectrum for *rac*-4ck.

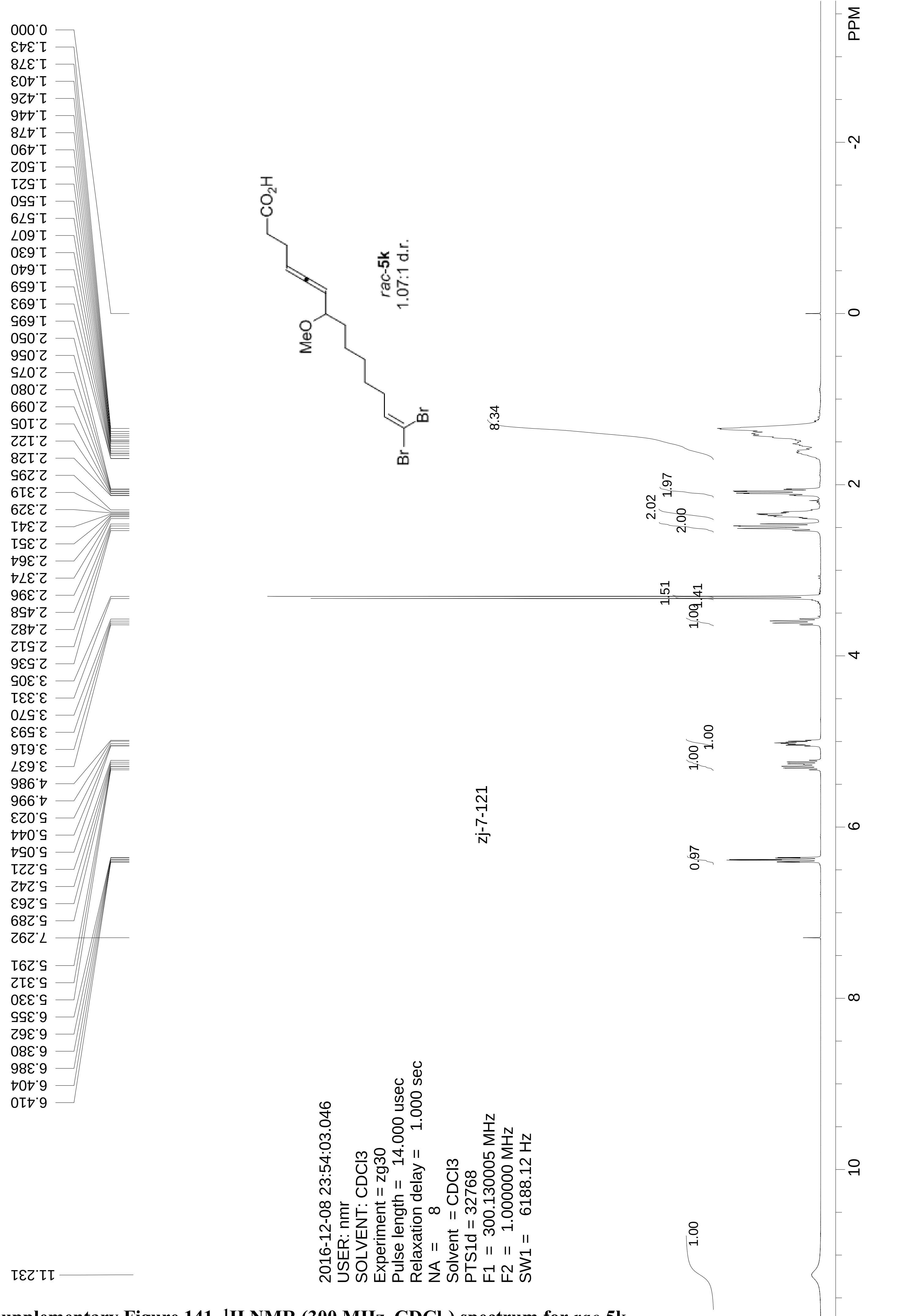

Supplementary Figure 141. ^{1}H NMR (300 MHz, $CDCl_3$) spectrum for *rac*-5k.

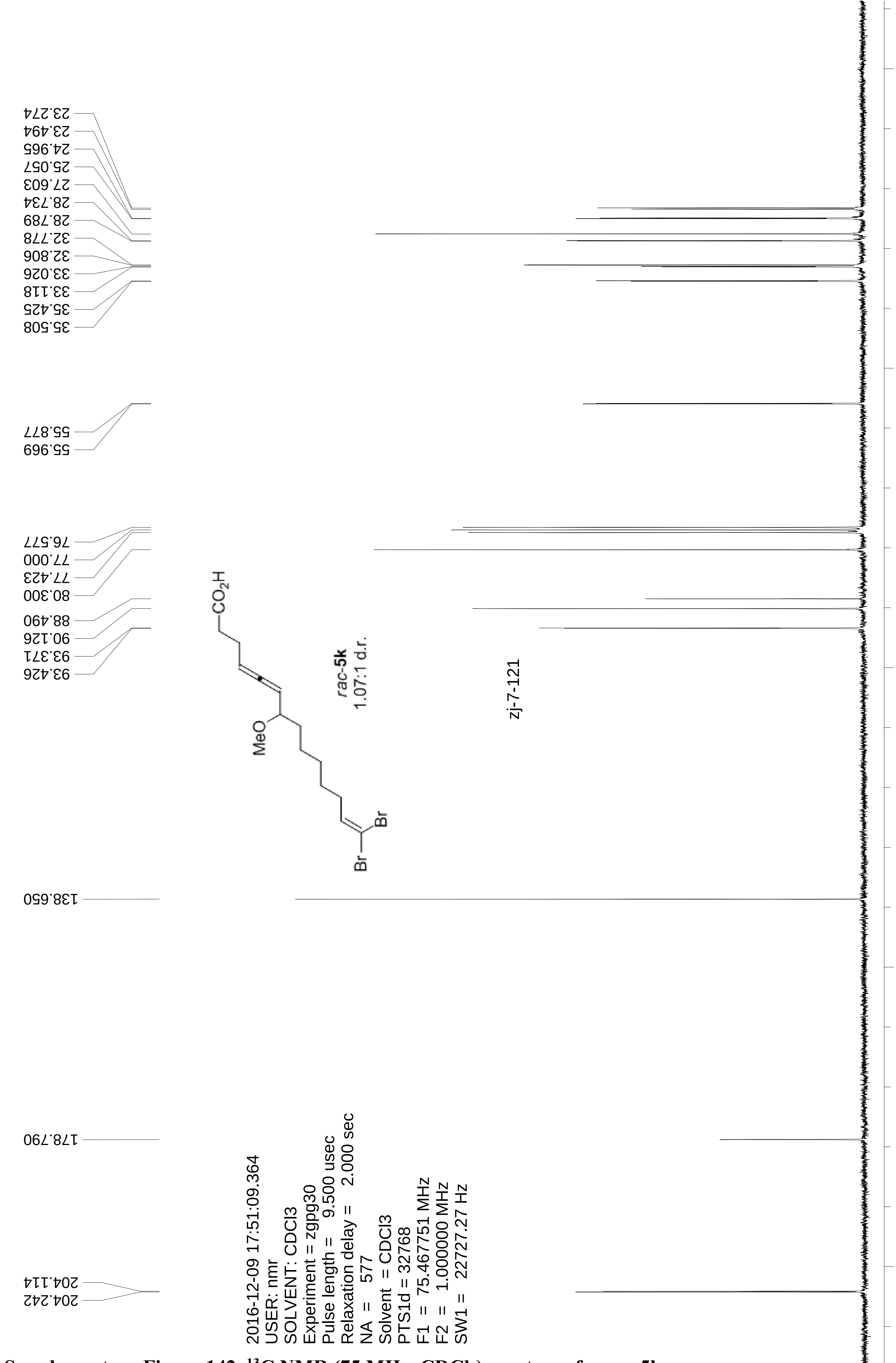

Supplementary Figure 142. ^{13}**C NMR (75 MHz, CDCl**$_3$**) spectrum for** ***rac*****-5k.**

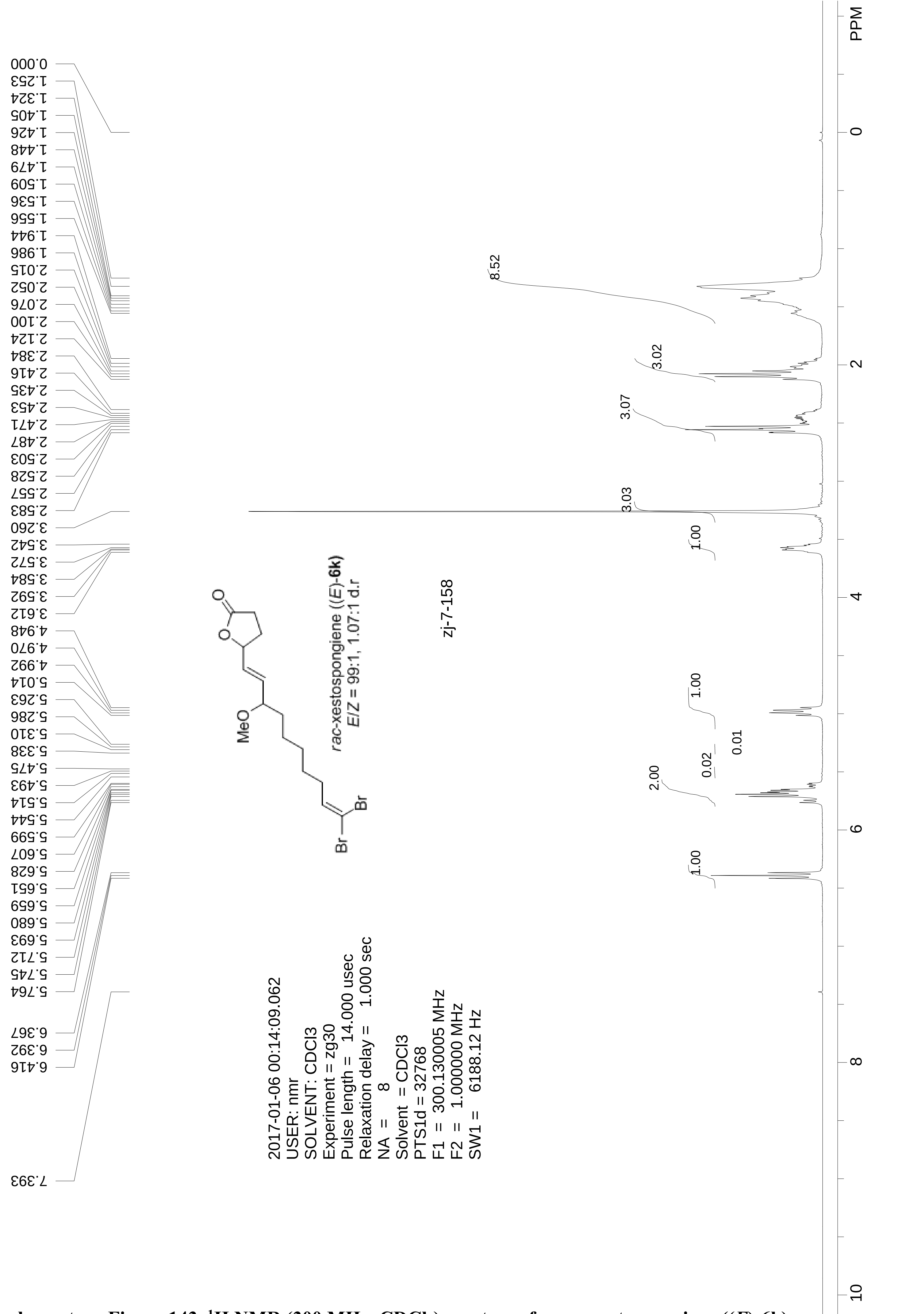

Supplementary Figure 143. ^{1}H NMR (300 MHz, $CDCl_3$) spectrum for *rac*-xestospongiene ((*E*)-6k).

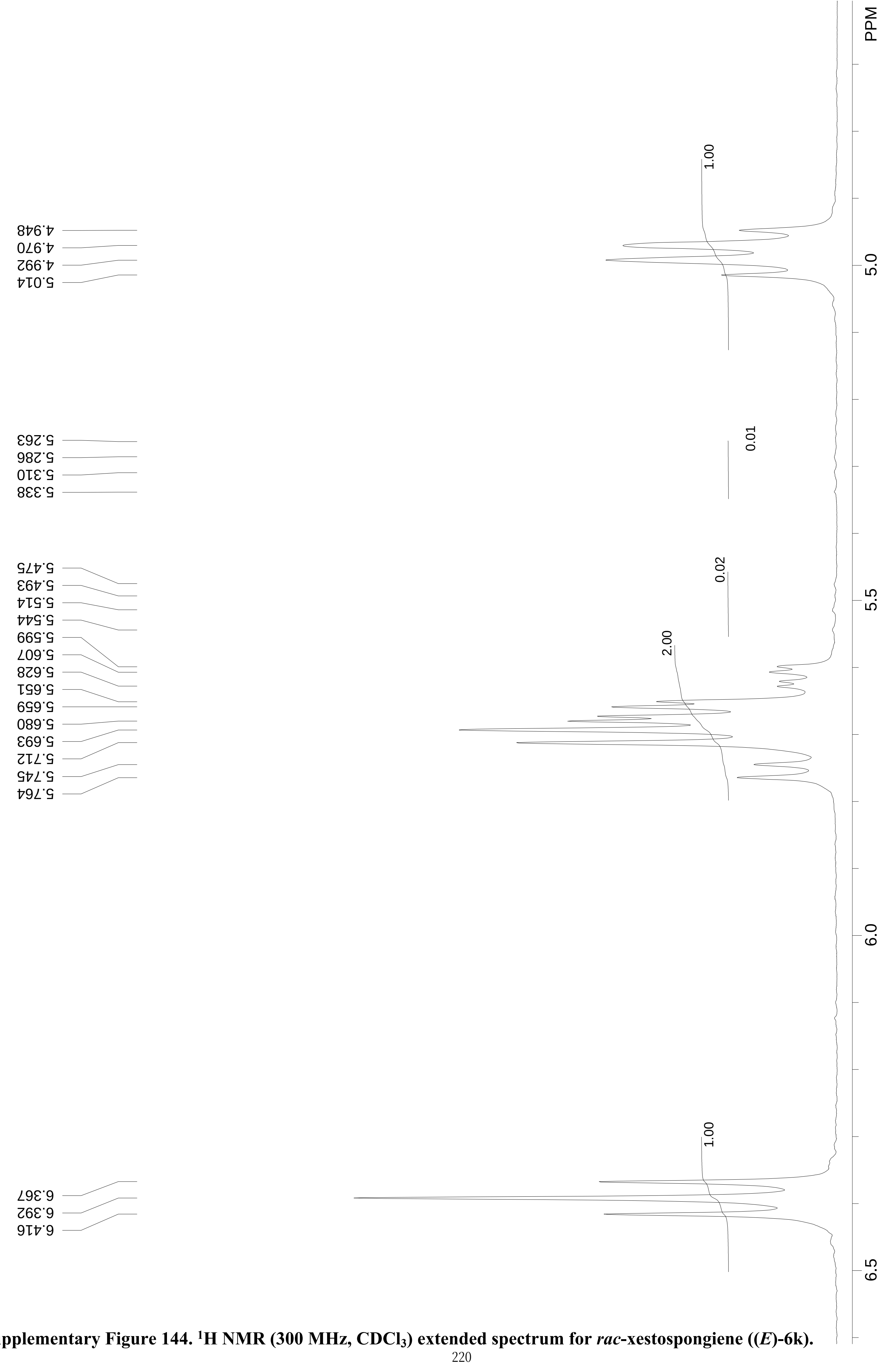

Supplementary Figure 144. ^{1}H NMR (300 MHz, $CDCl_3$) extended spectrum for *rac*-xestospongiene ((*E*)-6k).

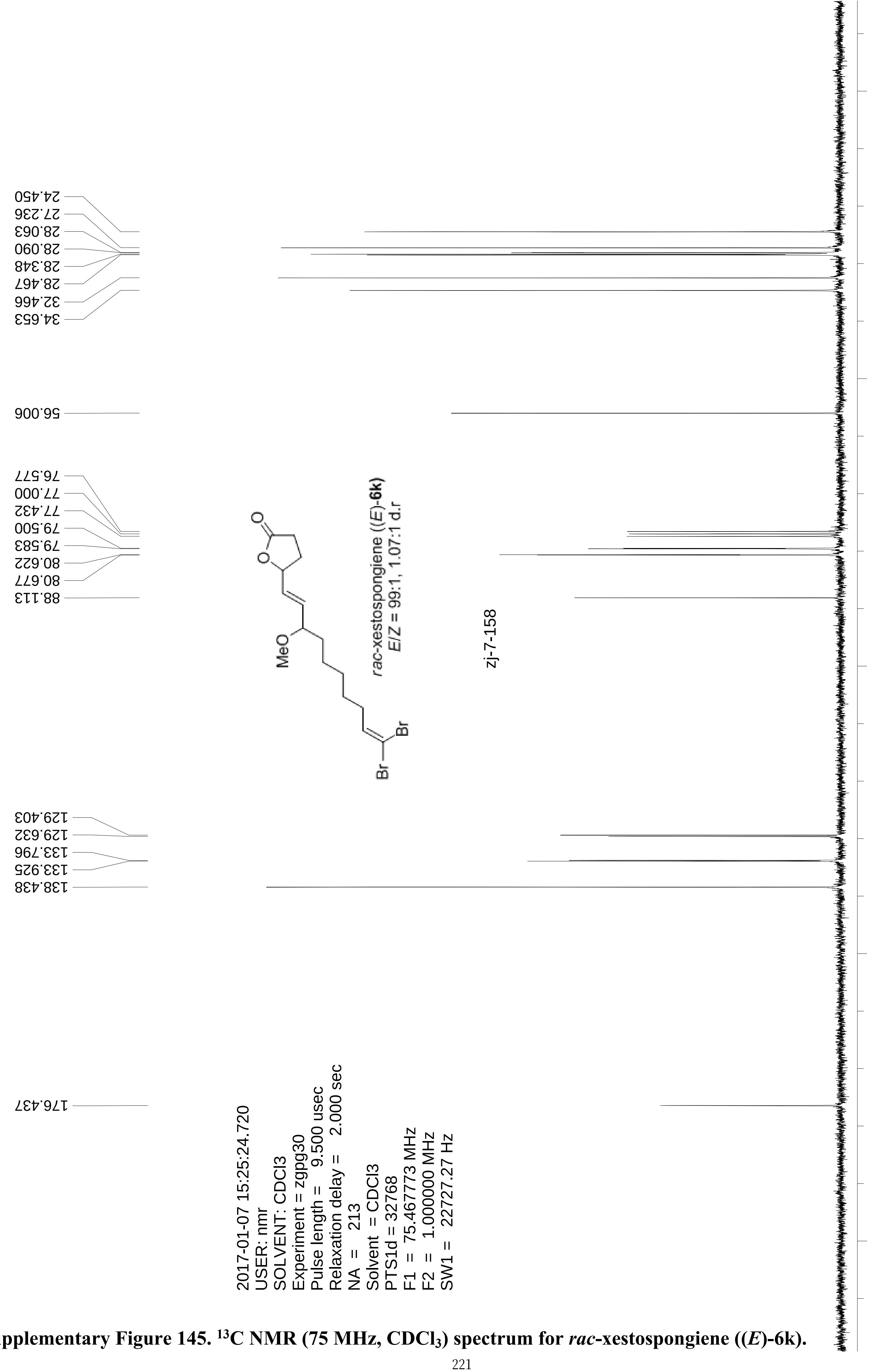

Supplementary Figure 145. **^{13}C NMR (75 MHz, $CDCl_3$) spectrum for *rac*-xestospongiene ((*E*)-6k).**

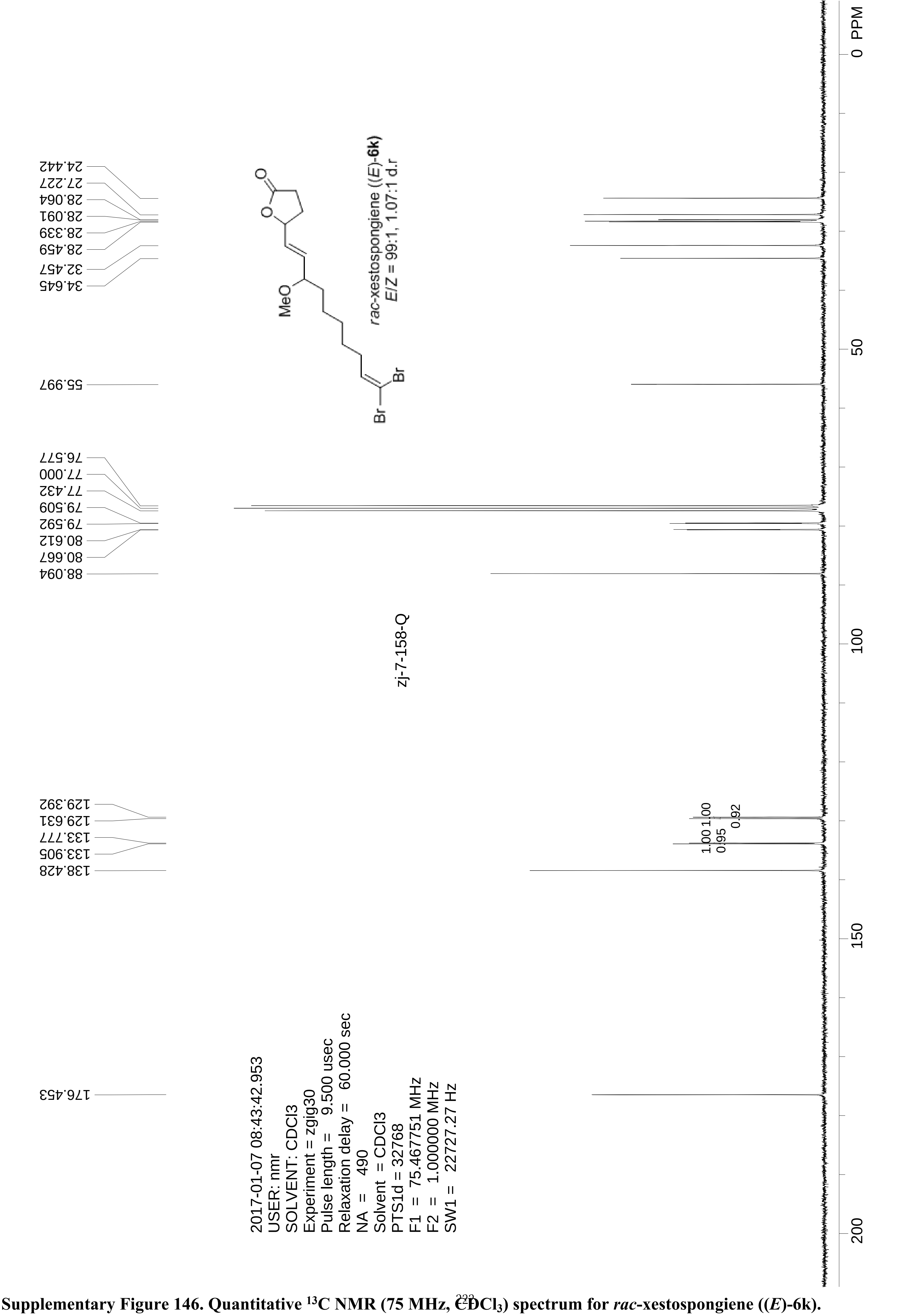

Supplementary Figure 146. Quantitative ^{13}C NMR (75 MHz, $CDCl_3$) spectrum for *rac*-xestospongiene ((*E*)-6k).

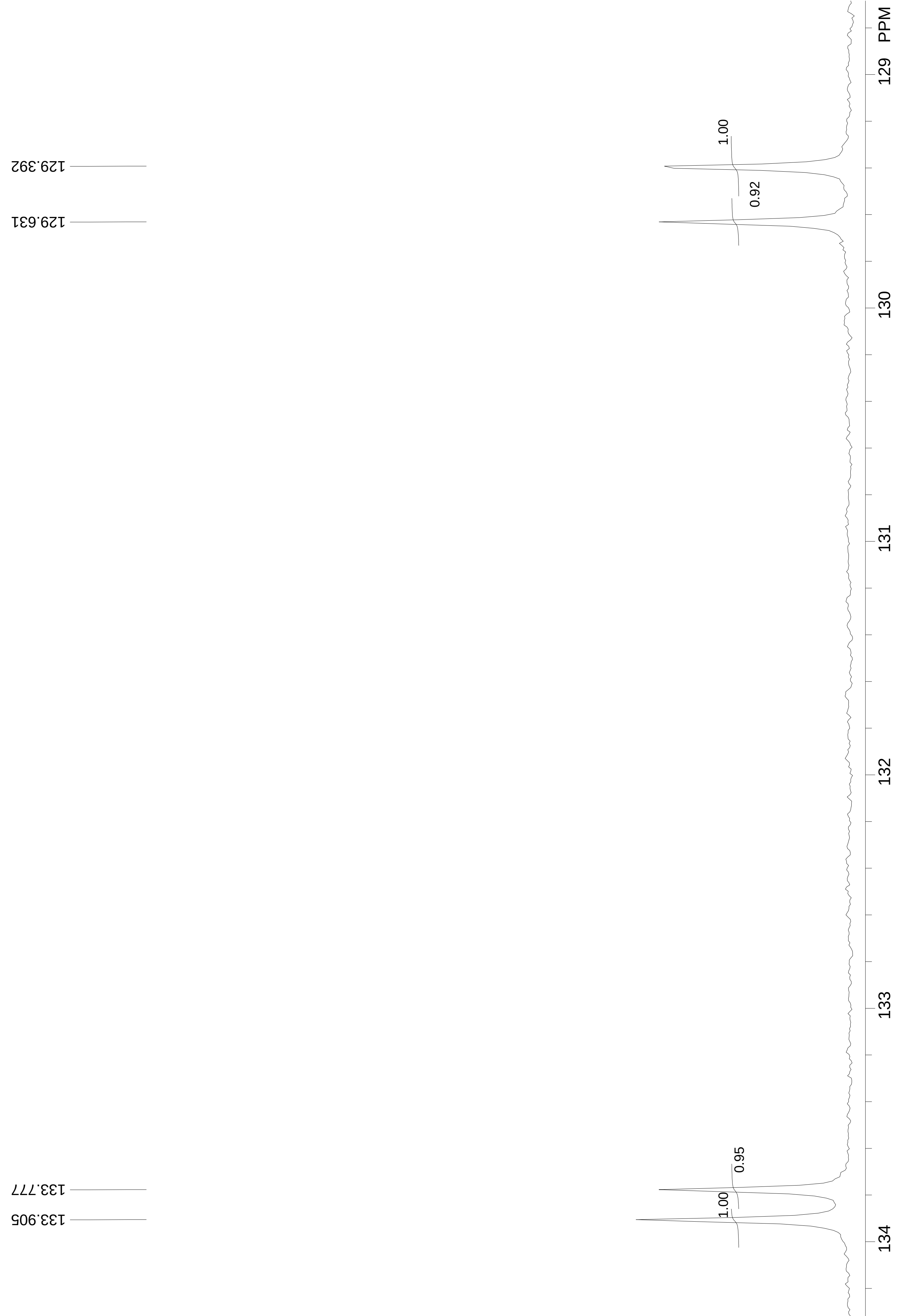

Supplementary Figure 147. Quantitative ^{13}C NMR (75 MHz, $CDCl_3$) extended spectrum for *rac*-xestospongiene ((*E*)-6k).

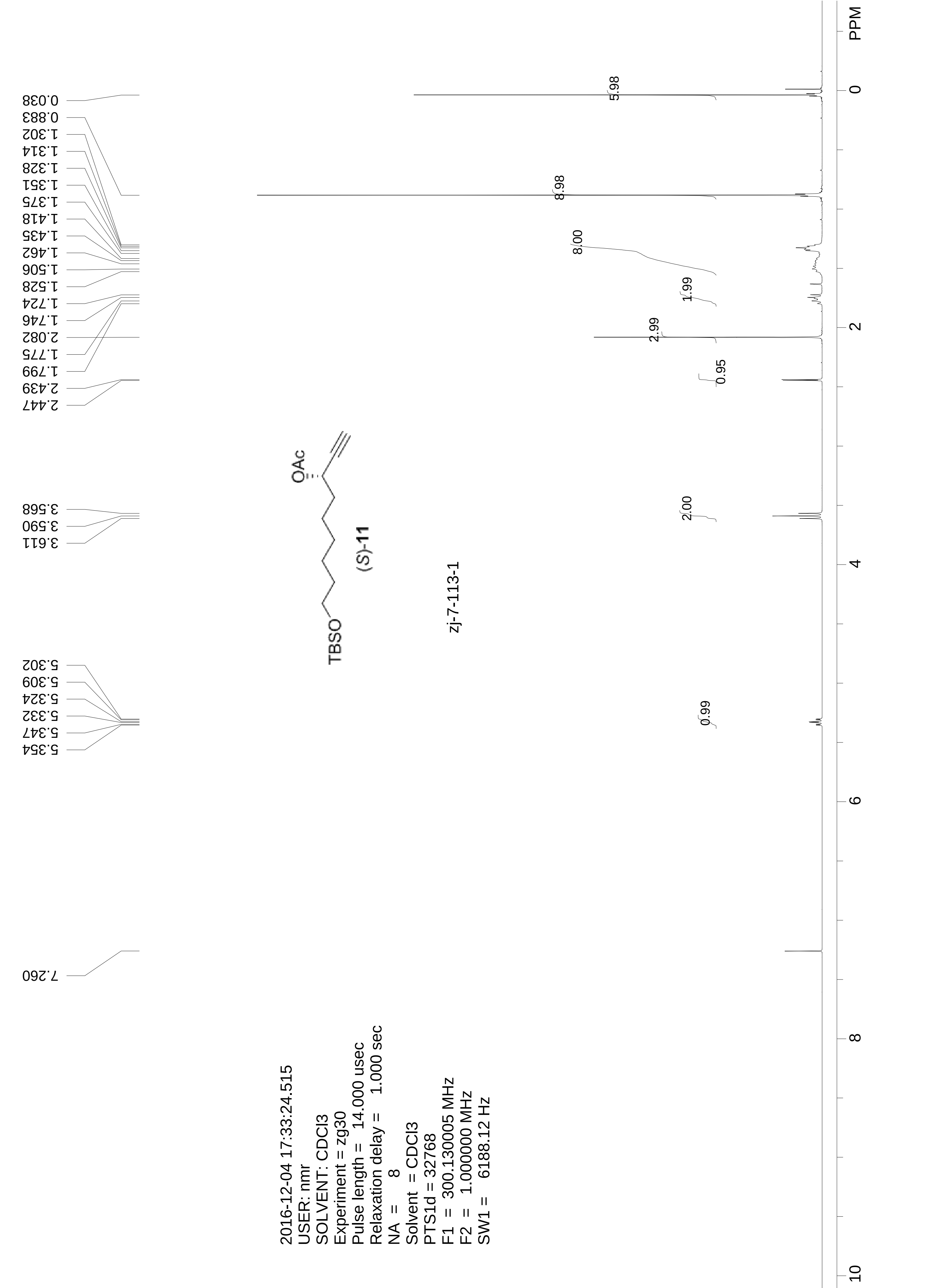

Supplementary Figure 148. ^{1}H NMR (300 MHz, $CDCl_3$) spectrum for (*S*)-11.

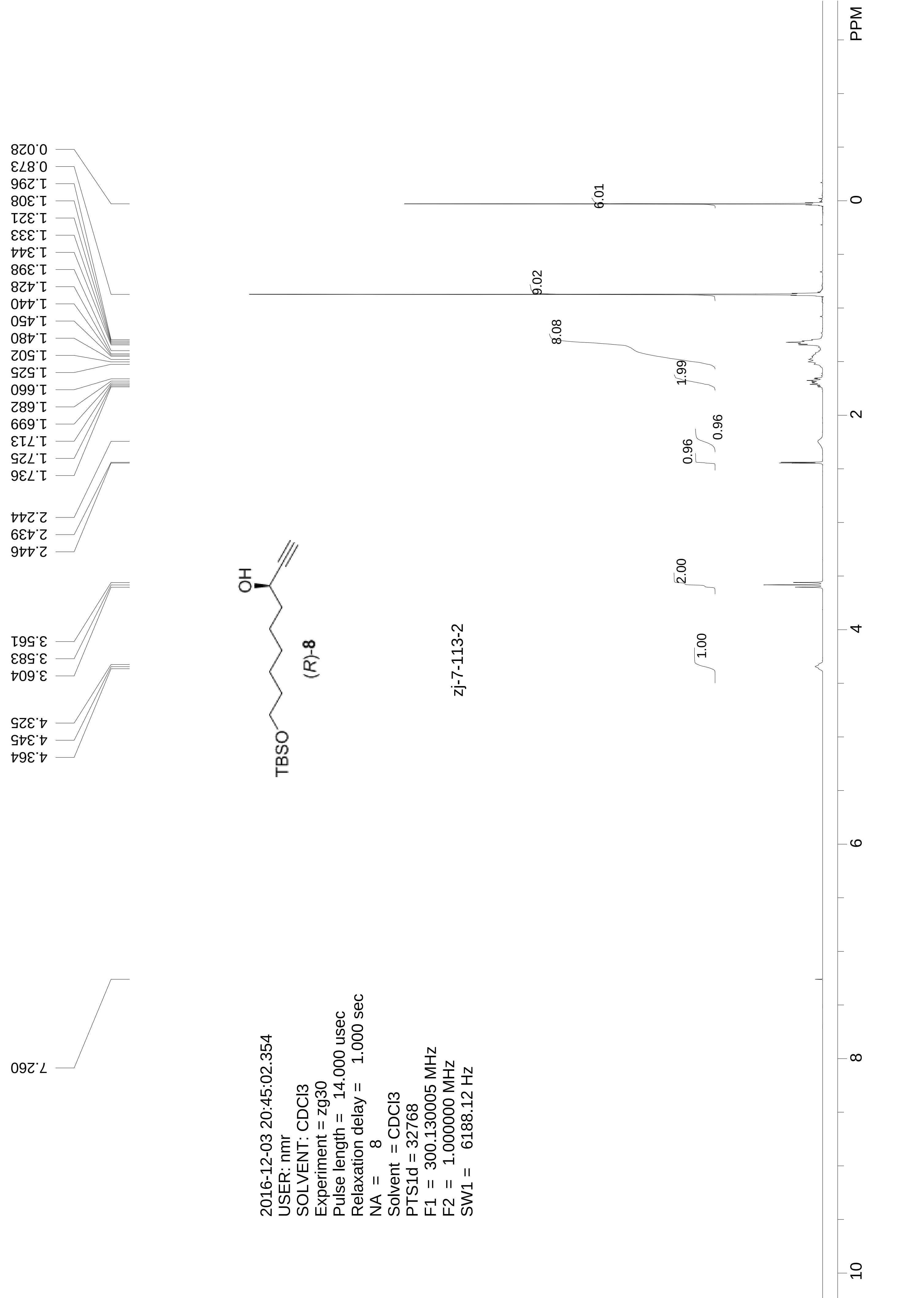

Supplementary Figure 149. ^{1}**H NMR (300 MHz, CDCl$_3$) spectrum for (*R*)-8.**

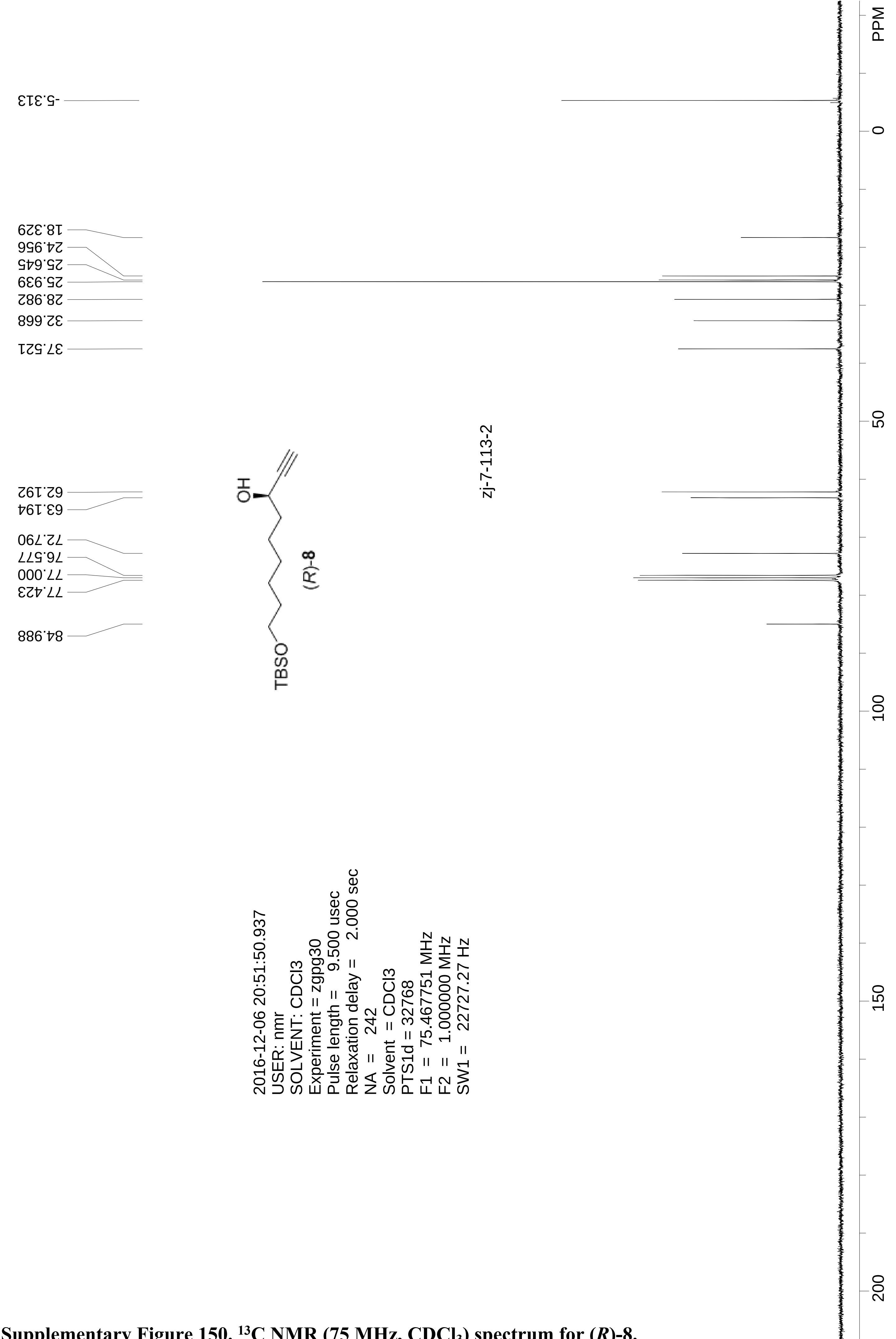

Supplementary Figure 150. ^{13}**C NMR (75 MHz, CDCl**$_3$**) spectrum for (*R*)-8.**

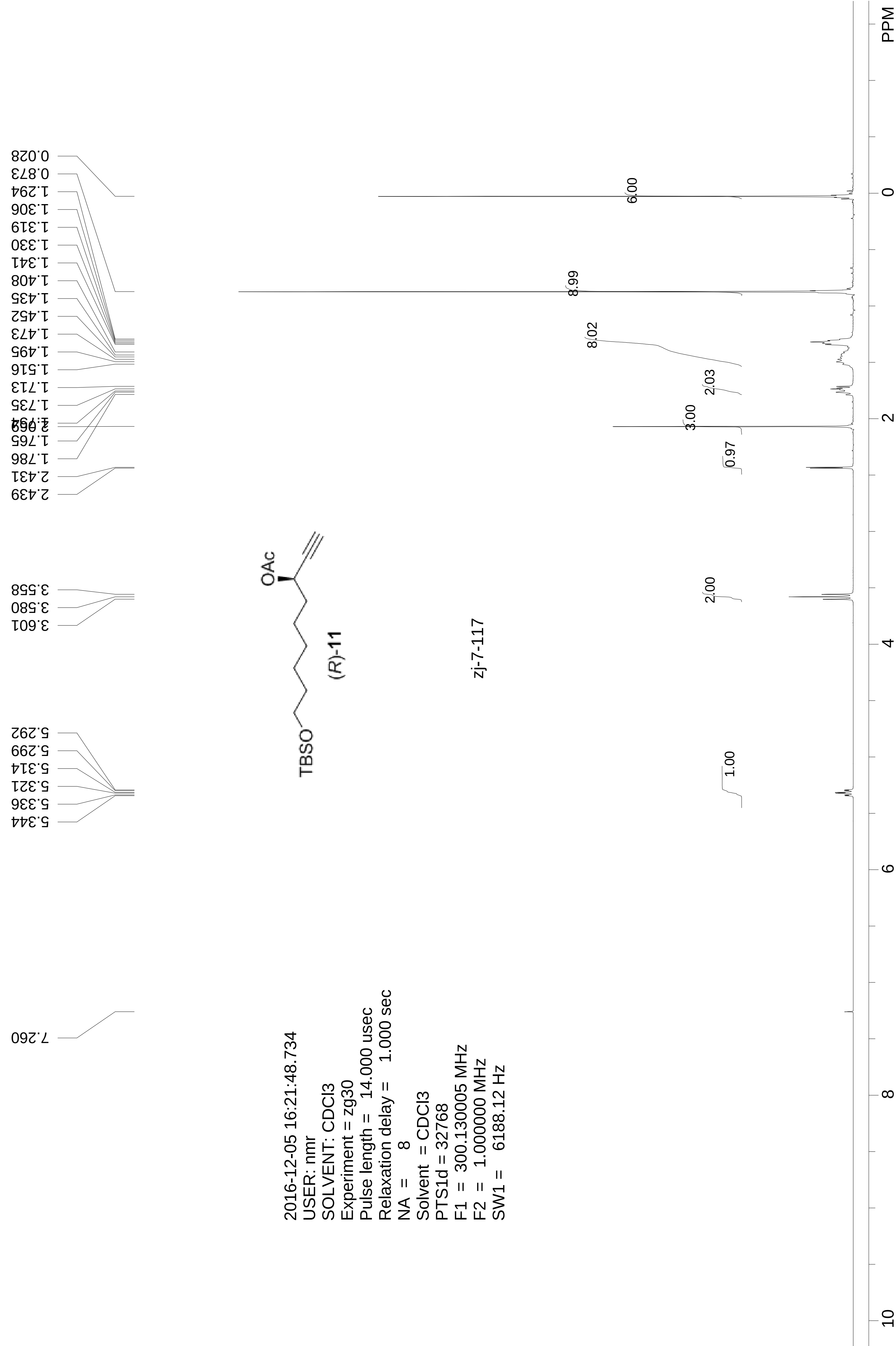

Supplementary Figure 151. ^{1}**H NMR (300 MHz,** $CDCl_3$**) spectrum for (*R*)-11.**

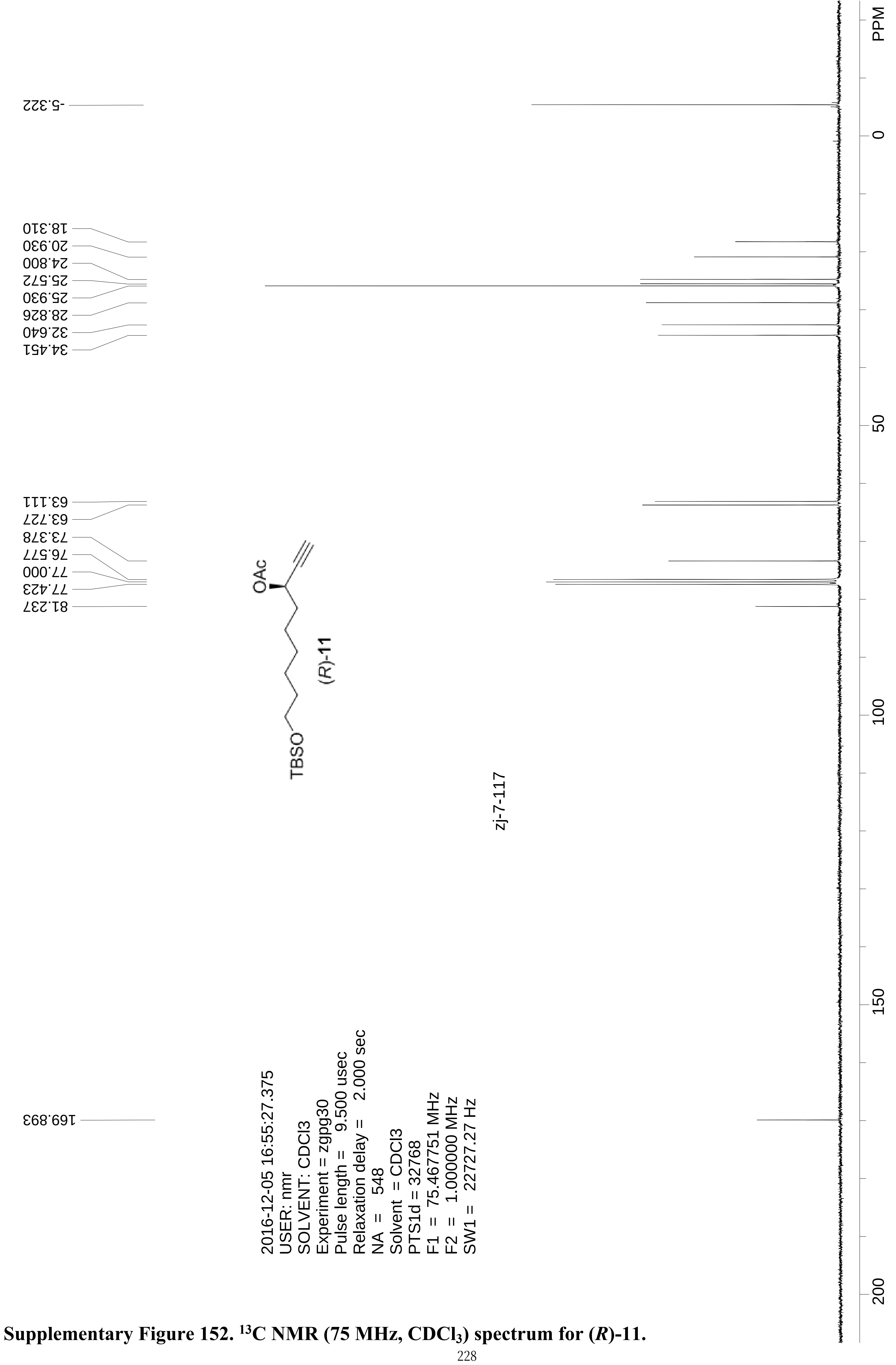

Supplementary Figure 152. ^{13}**C NMR (75 MHz,** $CDCl_3$**) spectrum for (*R*)-11.**

Data File C:\CHEM32\1\DATA\SIG10189.D
Sample Name: 7-117

===

Acq. Operator :
Acq. Instrument : Instrument 1 Location : Vial 1
Injection Date : 12/13/2016 10:18:05 AM
Inj Volume : Manually
Acq. Method : C:\CHEM32\1\METHODS\DEF_GC.M
Last changed : 12/13/2016 10:18:04 AM
(modified after loading)
Analysis Method : C:\CHEM32\1\METHODS\DEF_GC.M
Last changed : 12/13/2016 2:19:39 PM
(modified after loading)
Sample Info : dexcb

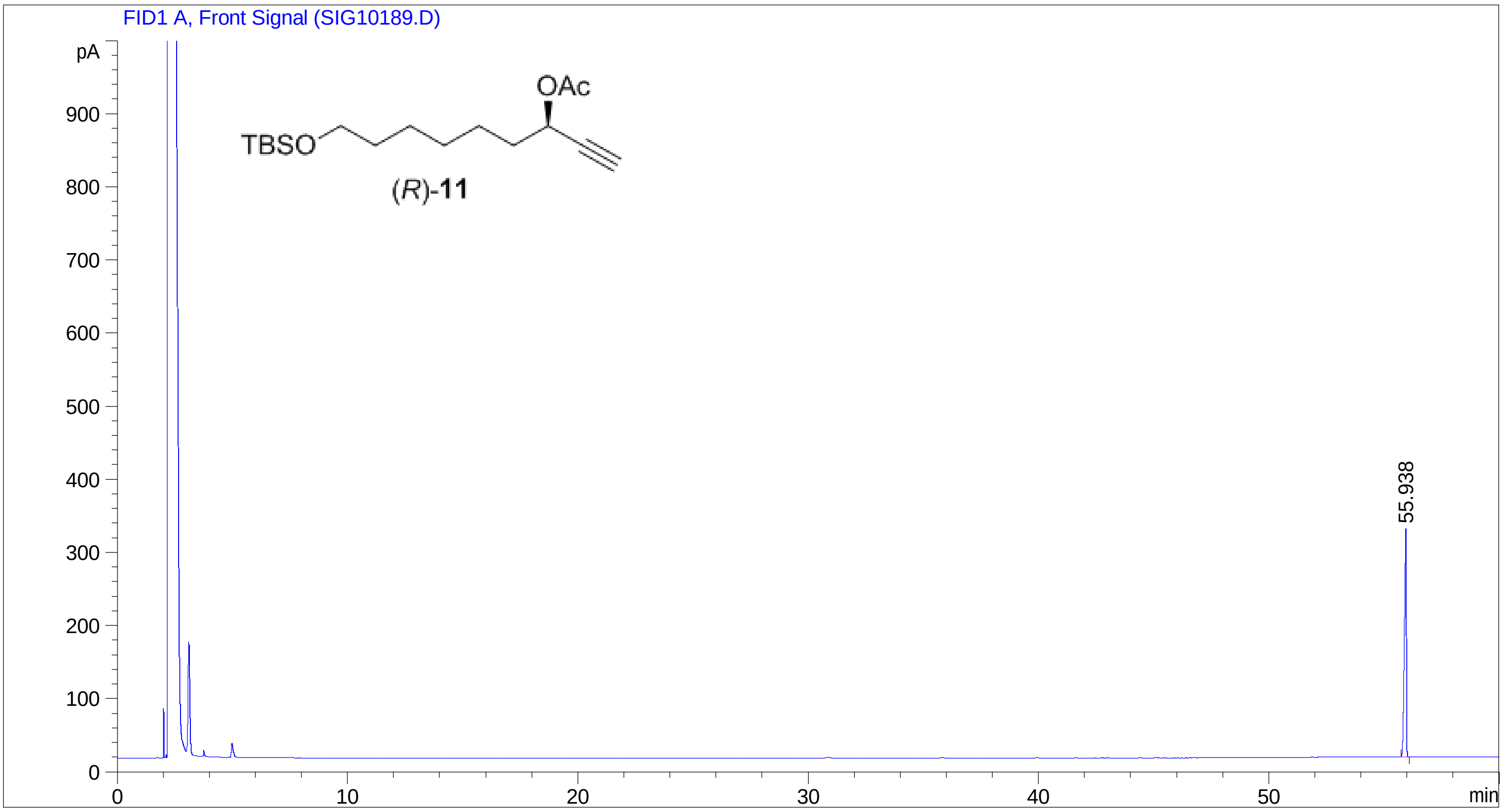

===
Area Percent Report
===

Sorted By : Signal
Multiplier: : 1.0000
Dilution: : 1.0000
Use Multiplier & Dilution Factor with ISTDs

Signal 1: FID1 A, Front Signal

Peak #	RetTime [min]	Type	Width [min]	Area [pA*s]	Height [pA]	Area %
1	55.938	BB	0.0854	1869.97827	310.96283	1.000e2

Totals : 1869.97827 310.96283

Supplementary Figure 153. GC spectrum for (*R*)-11.

===

Acq. Operator :
Acq. Instrument : Instrument 1 Location : Vial 1
Injection Date : 12/13/2016 9:09:27 AM
Inj Volume : Manually
Acq. Method : C:\CHEM32\1\METHODS\DEF_GC.M
Last changed : 12/13/2016 9:09:26 AM
(modified after loading)
Analysis Method : C:\CHEM32\1\METHODS\DEF_GC.M
Last changed : 12/13/2016 2:18:41 PM
(modified after loading)
Sample Info : dexcb

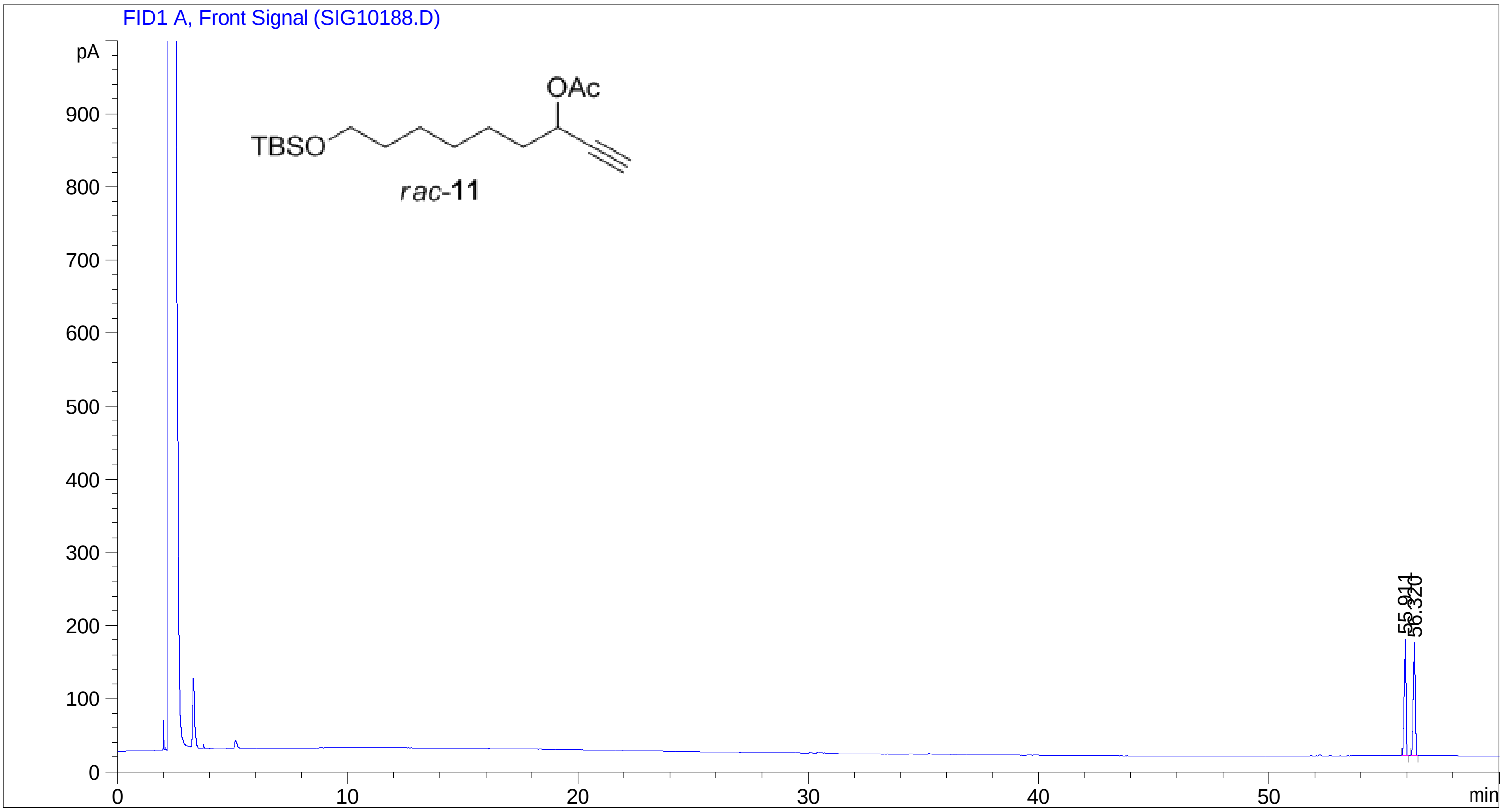

===

Area Percent Report

===

Sorted By : Signal
Multiplier: : 1.0000
Dilution: : 1.0000
Use Multiplier & Dilution Factor with ISTDs

Signal 1: FID1 A, Front Signal

Peak #	RetTime [min]	Type	Width [min]	Area [pA*s]	Height [pA]	Area %
1	55.911	BB	0.0877	874.01947	158.17586	49.57828
2	56.320	BB	0.0881	888.88849	154.14984	50.42172

Totals : 1762.90796 312.32570

Supplementary Figure 154. GC spectrum for *rac*-11.

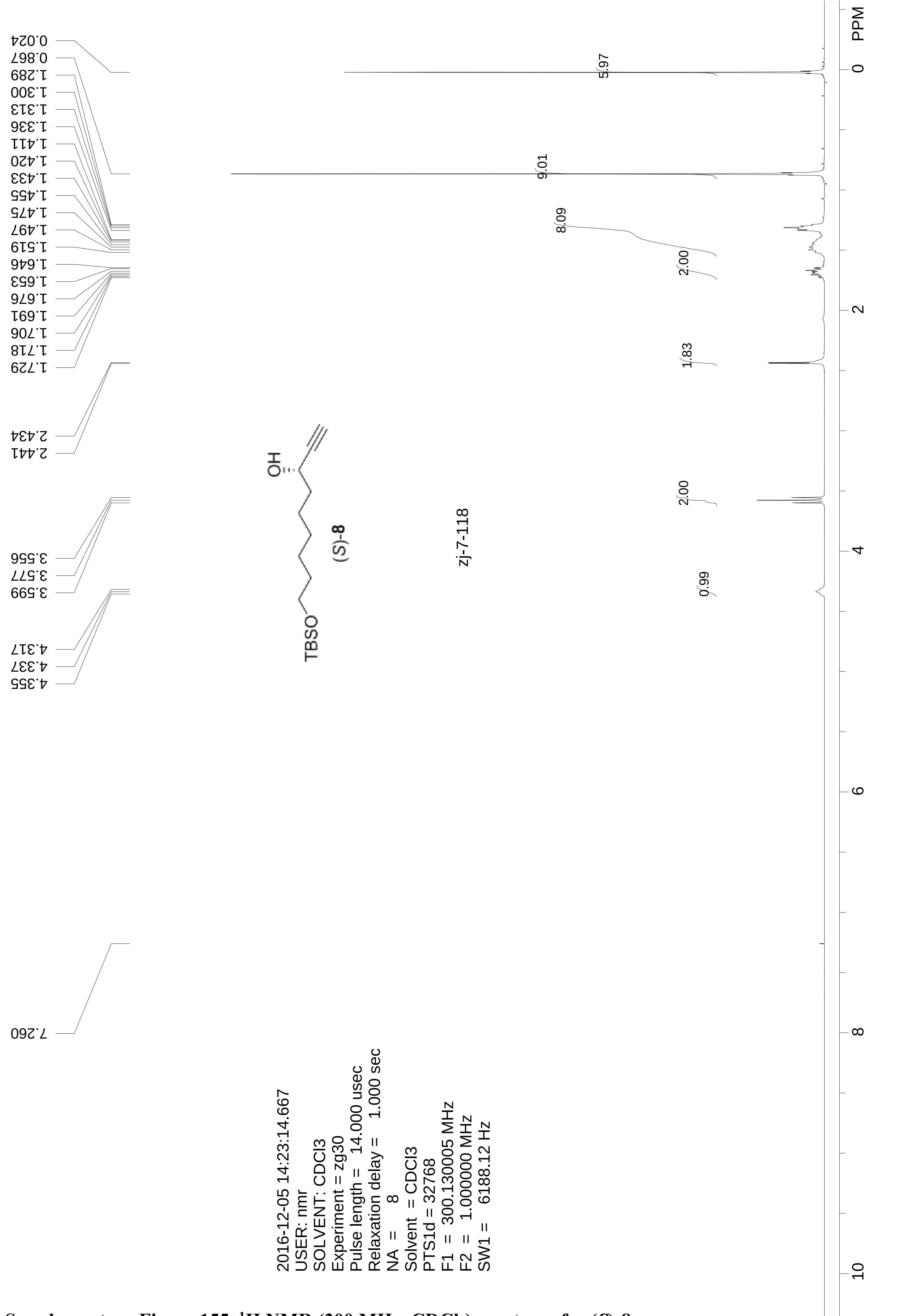

Supplementary Figure 155. ^{1}**H NMR (300 MHz, CDCl$_3$) spectrum for (*S*)-8.**

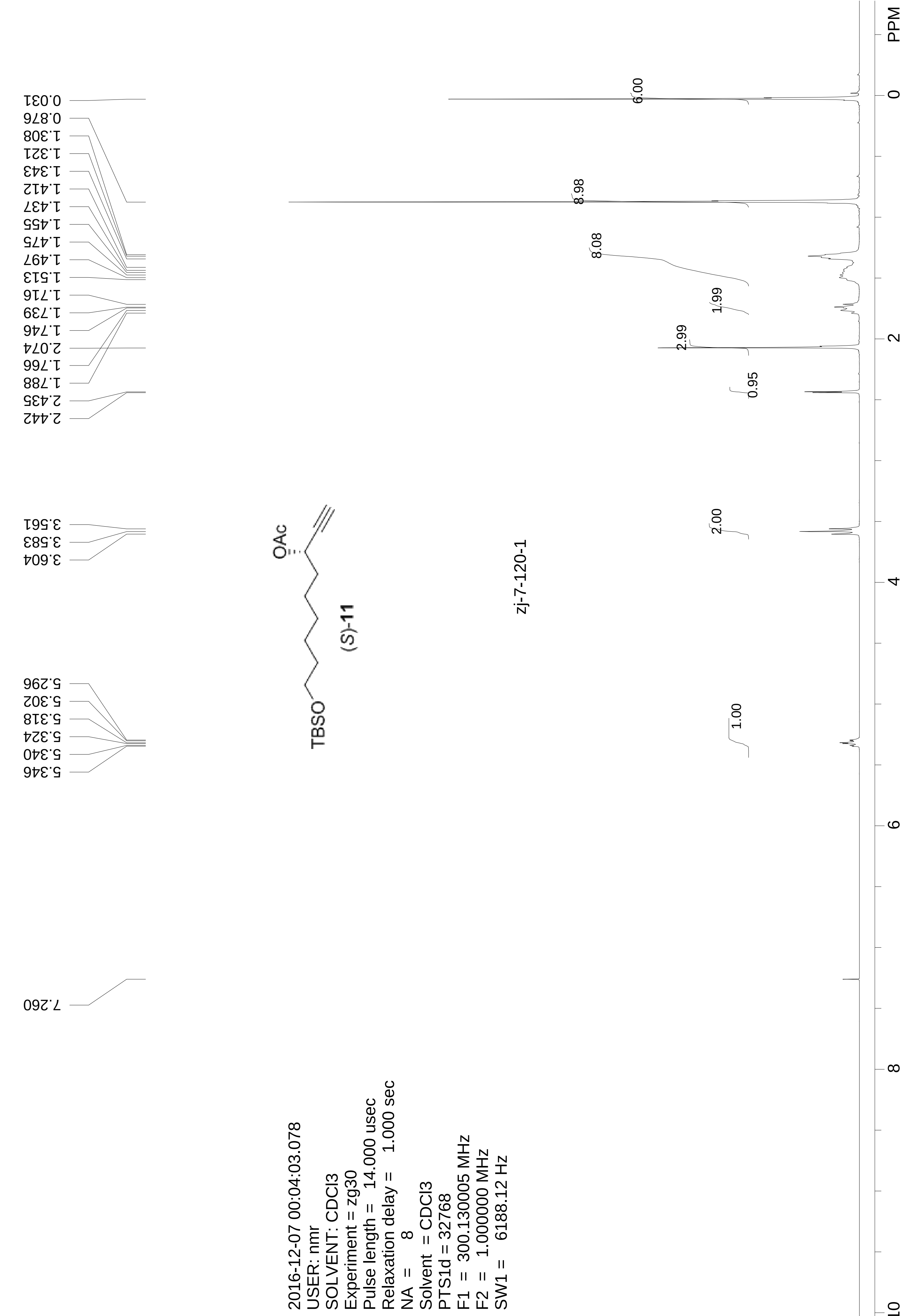

Supplementary Figure 156. ^{1}H NMR (300 MHz, $CDCl_3$) spectrum for (*S*)-11.

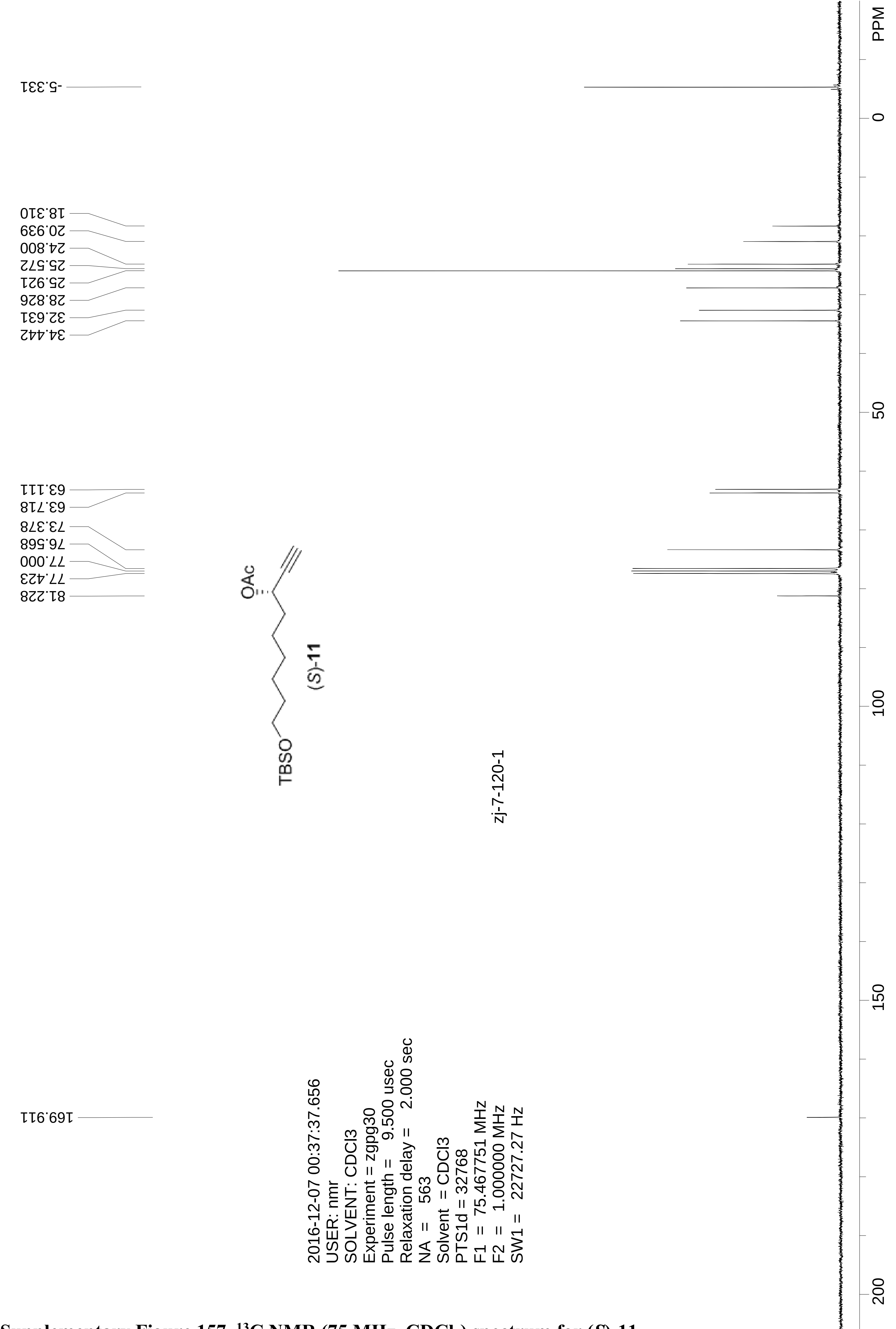

Supplementary Figure 157. ^{13}C **NMR (75 MHz,** $CDCl_3$**) spectrum for (*S*)-11.**

Data File C:\CHEM32\1\DATA\SIG10190.D
Sample Name: 7-120-1

===

Acq. Operator :
Acq. Instrument : Instrument 1 Location : Vial 1
Injection Date : 12/13/2016 11:32:29 AM
Inj Volume : Manually
Acq. Method : C:\CHEM32\1\METHODS\DEF_GC.M
Last changed : 12/13/2016 11:32:28 AM
(modified after loading)
Analysis Method : C:\CHEM32\1\METHODS\DEF_GC.M
Last changed : 12/13/2016 2:20:26 PM
(modified after loading)
Sample Info : dexcb

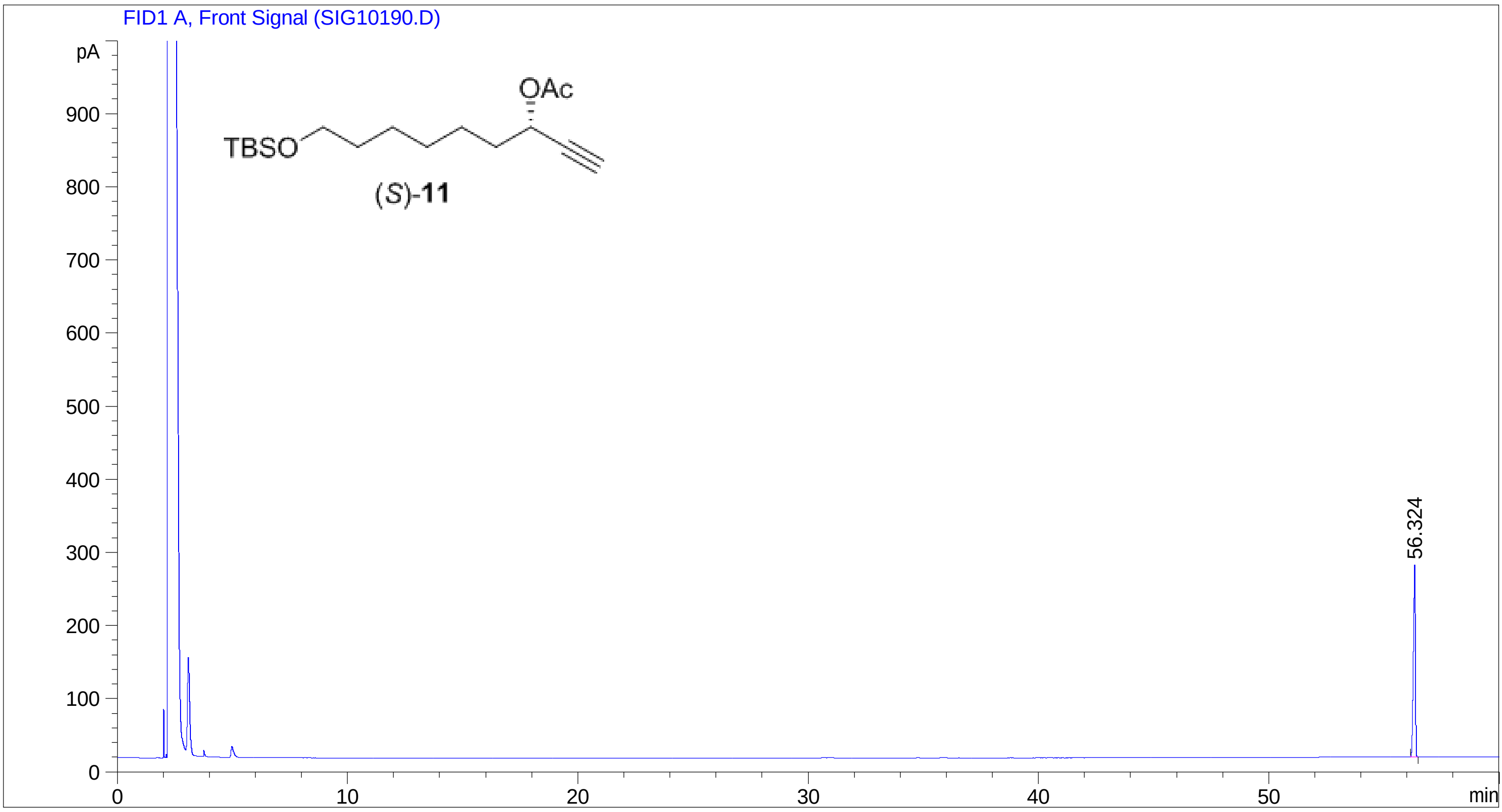

===

Area Percent Report

===

Sorted By : Signal
Multiplier: : 1.0000
Dilution: : 1.0000
Use Multiplier & Dilution Factor with ISTDs

Signal 1: FID1 A, Front Signal

Peak #	RetTime [min]	Type	Width [min]	Area [pA*s]	Height [pA]	Area %
1	56.324	BB	0.0918	1594.40137	262.14978	1.000e2

Totals : 1594.40137 262.14978

Supplementary Figure 158. GC spectrum for (*S*)-11.

Data File C:\CHEM32\1\DATA\SIG10188.D
Sample Name: 7-111-rac

===

Acq. Operator :
Acq. Instrument : Instrument 1 Location : Vial 1
Injection Date : 12/13/2016 9:09:27 AM
Inj Volume : Manually
Acq. Method : C:\CHEM32\1\METHODS\DEF_GC.M
Last changed : 12/13/2016 9:09:26 AM
(modified after loading)
Analysis Method : C:\CHEM32\1\METHODS\DEF_GC.M
Last changed : 12/13/2016 2:18:41 PM
(modified after loading)
Sample Info : dexcb

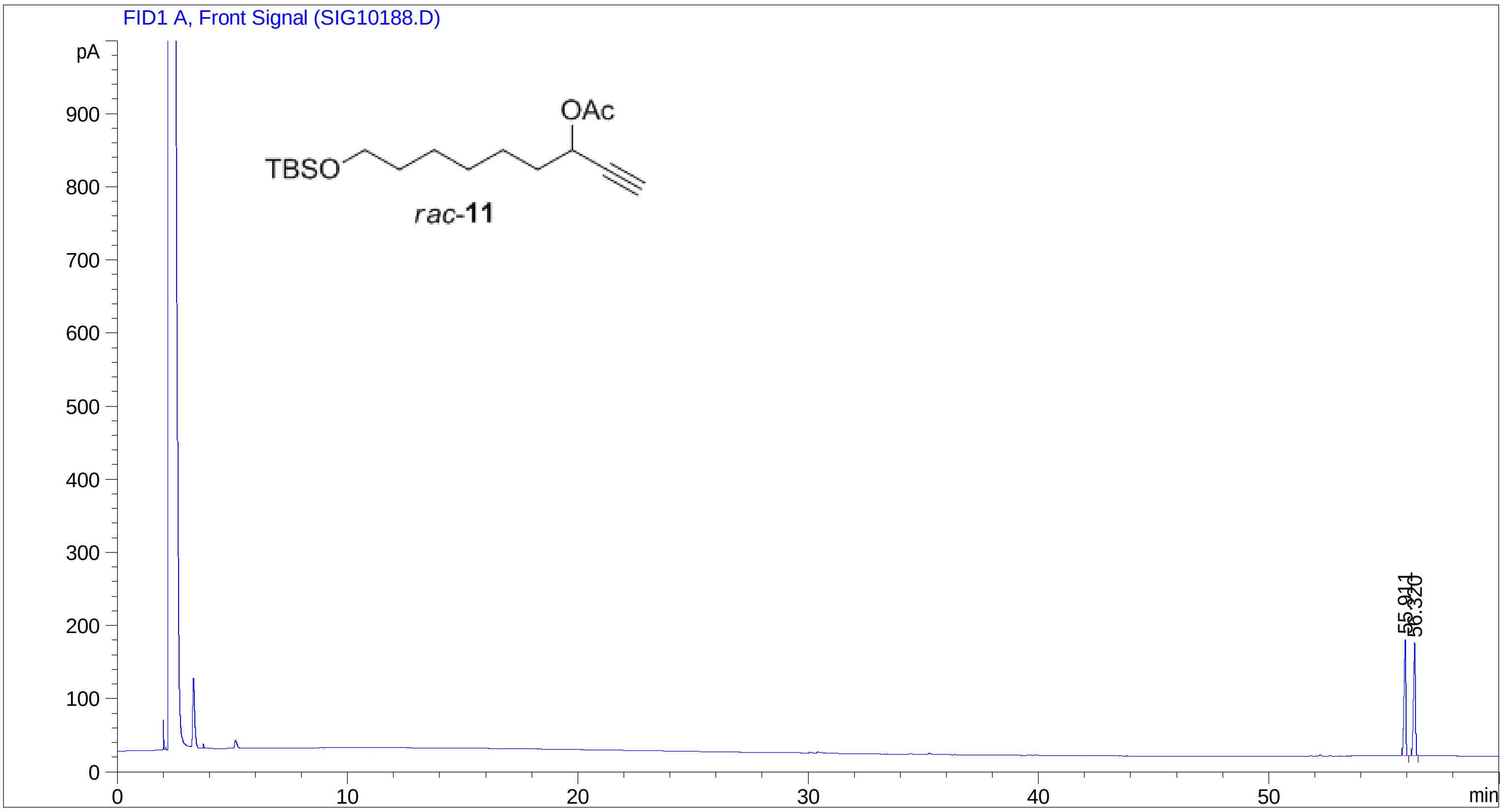

===
Area Percent Report
===

Sorted By : Signal
Multiplier: : 1.0000
Dilution: : 1.0000
Use Multiplier & Dilution Factor with ISTDs

Signal 1: FID1 A, Front Signal

Peak #	RetTime [min]	Type	Width [min]	Area [pA*s]	Height [pA]	Area %
1	55.911	BB	0.0877	874.01947	158.17586	49.57828
2	56.320	BB	0.0881	888.88849	154.14984	50.42172

Totals : 1762.90796 312.32570

Supplementary Figure 159. GC spectrum for *rac*-11.

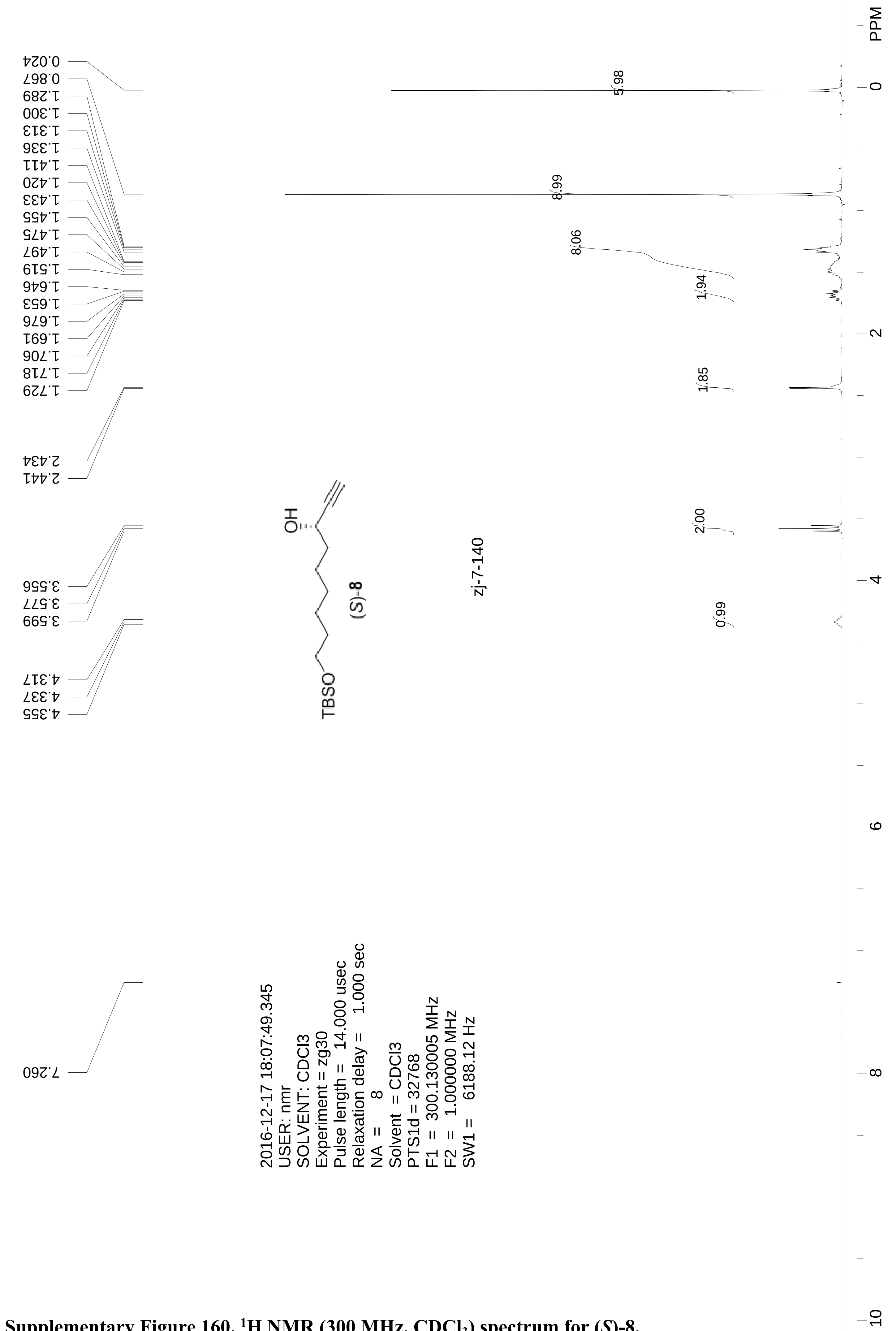

Supplementary Figure 160. ^{1}H NMR (300 MHz, $CDCl_3$) spectrum for (*S*)-8.

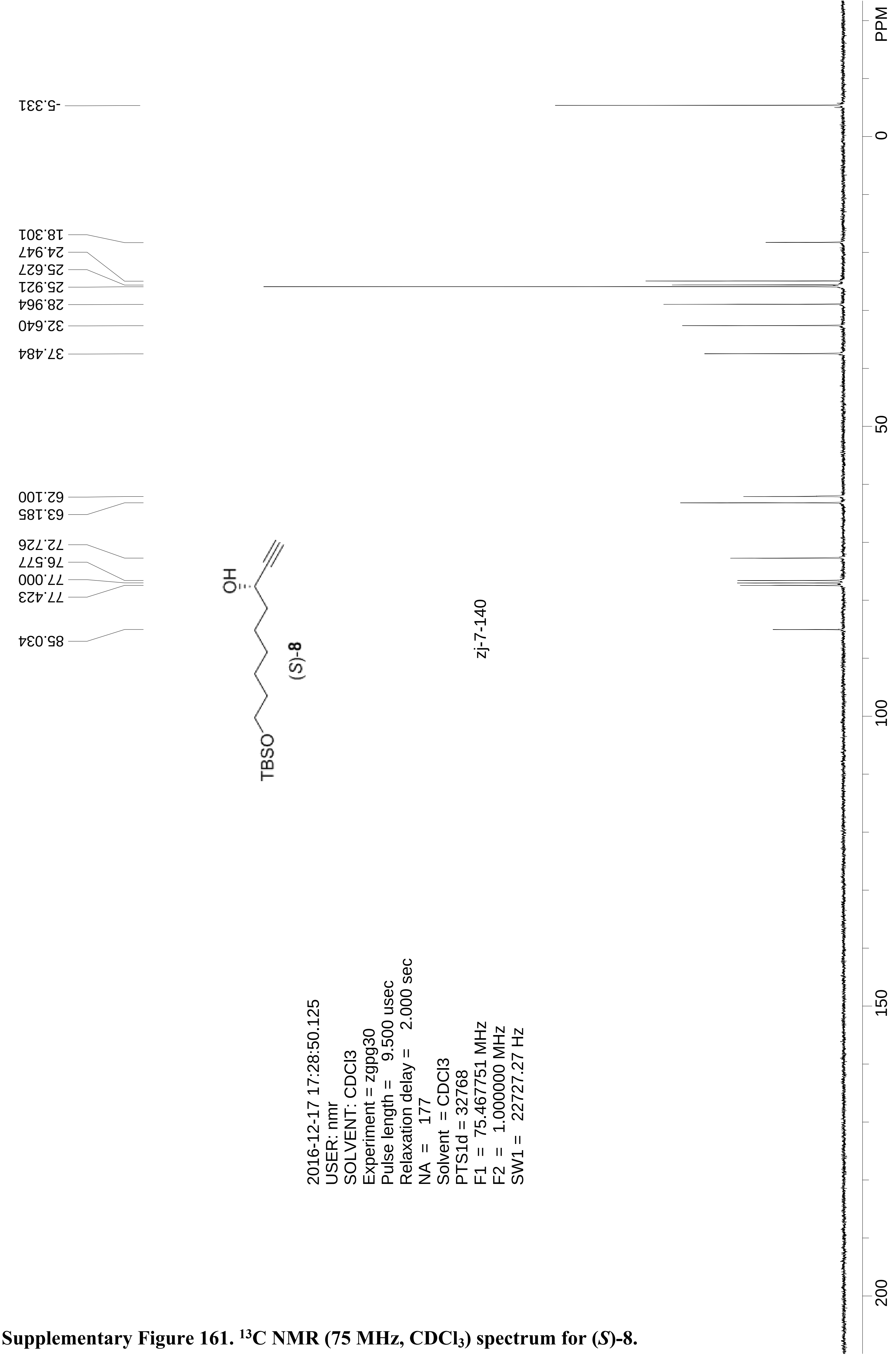

Supplementary Figure 161. ^{13}**C NMR (75 MHz, CDCl$_3$) spectrum for (*S*)-8.**

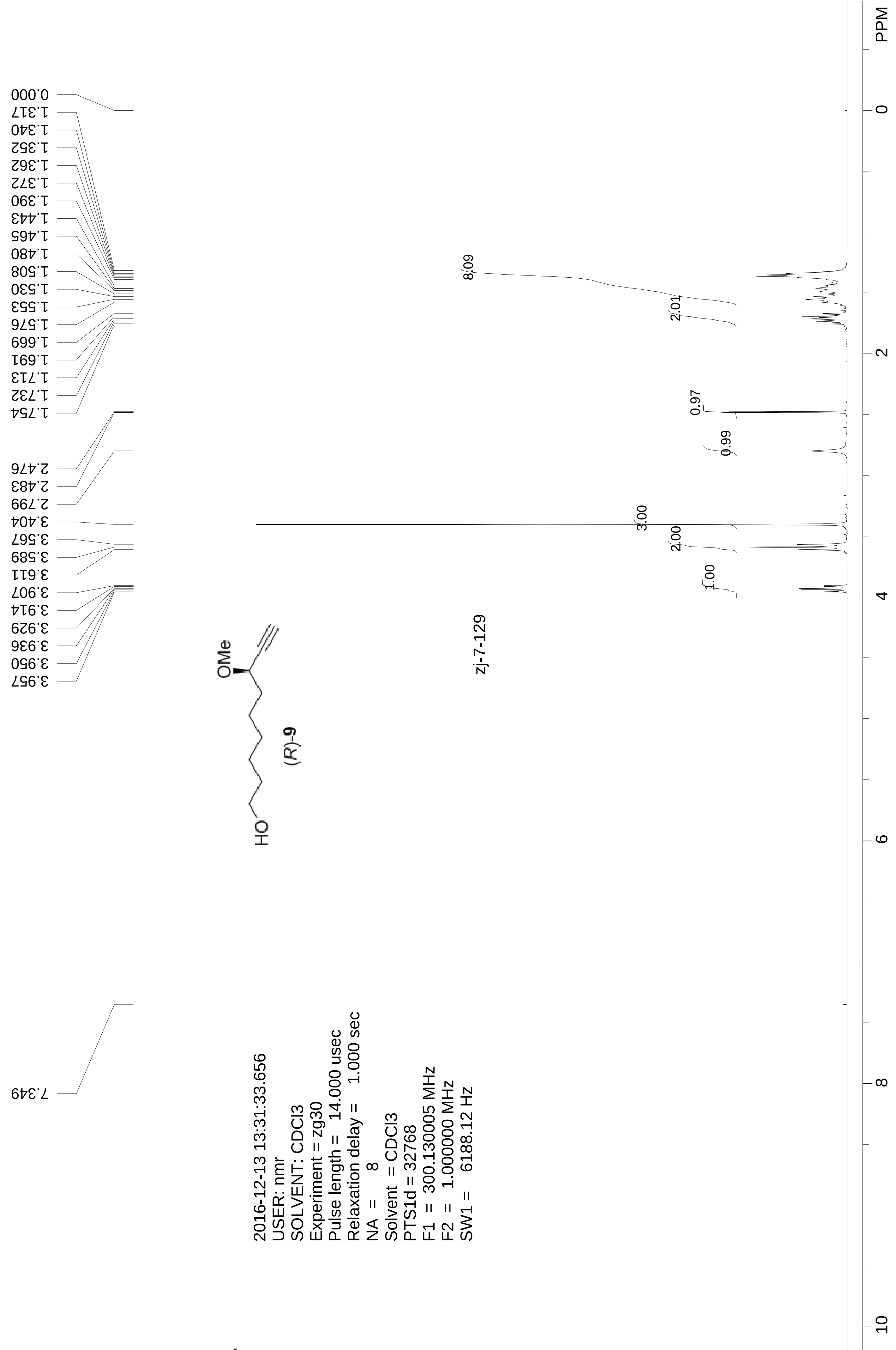

Supplementary Figure 162. ^{1}H NMR (300 MHz, $CDCl_3$) spectrum for (*R*)-9.

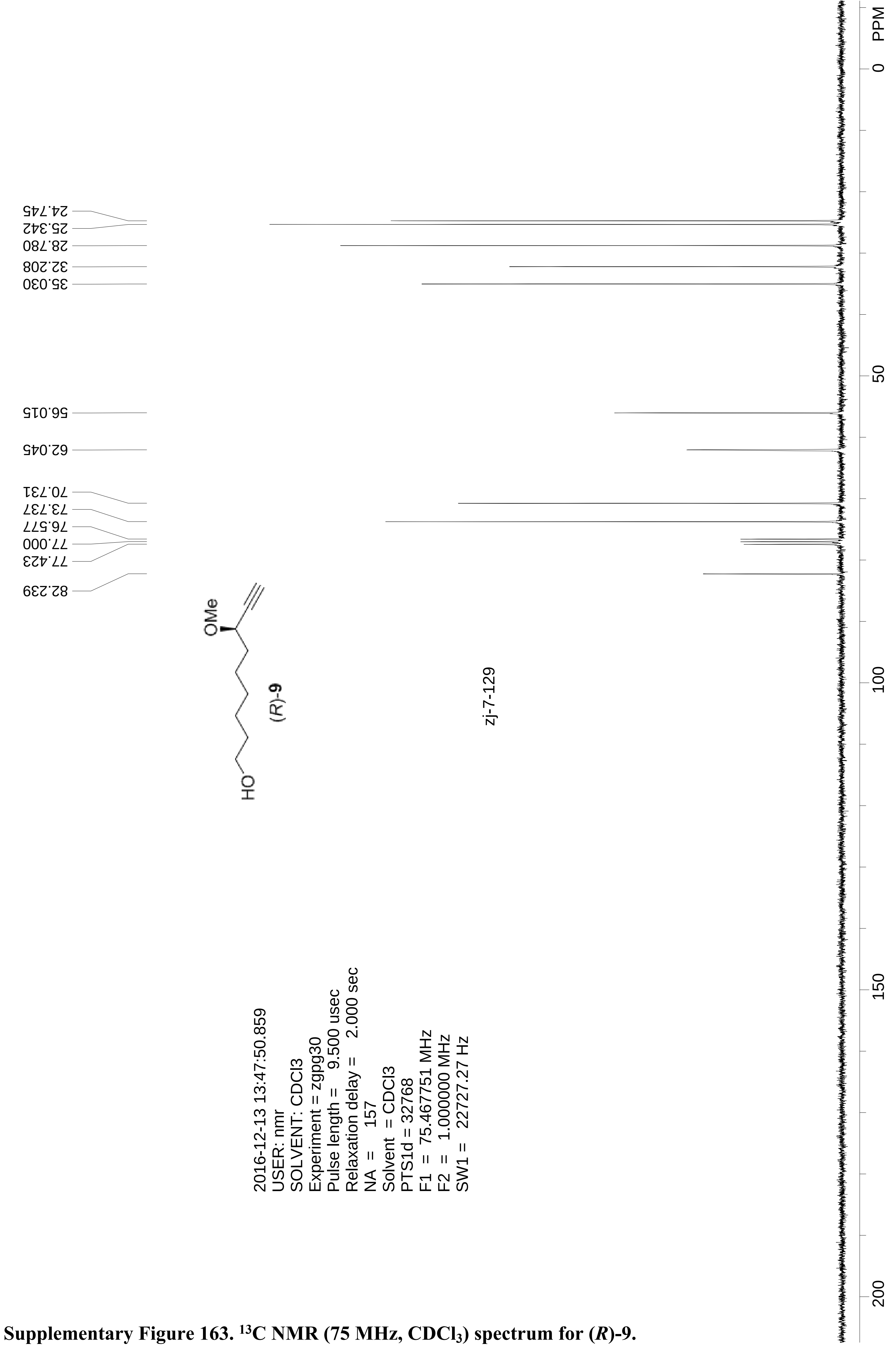

Supplementary Figure 163. ^{13}**C NMR (75 MHz,** $CDCl_3$**) spectrum for (*R*)-9.**

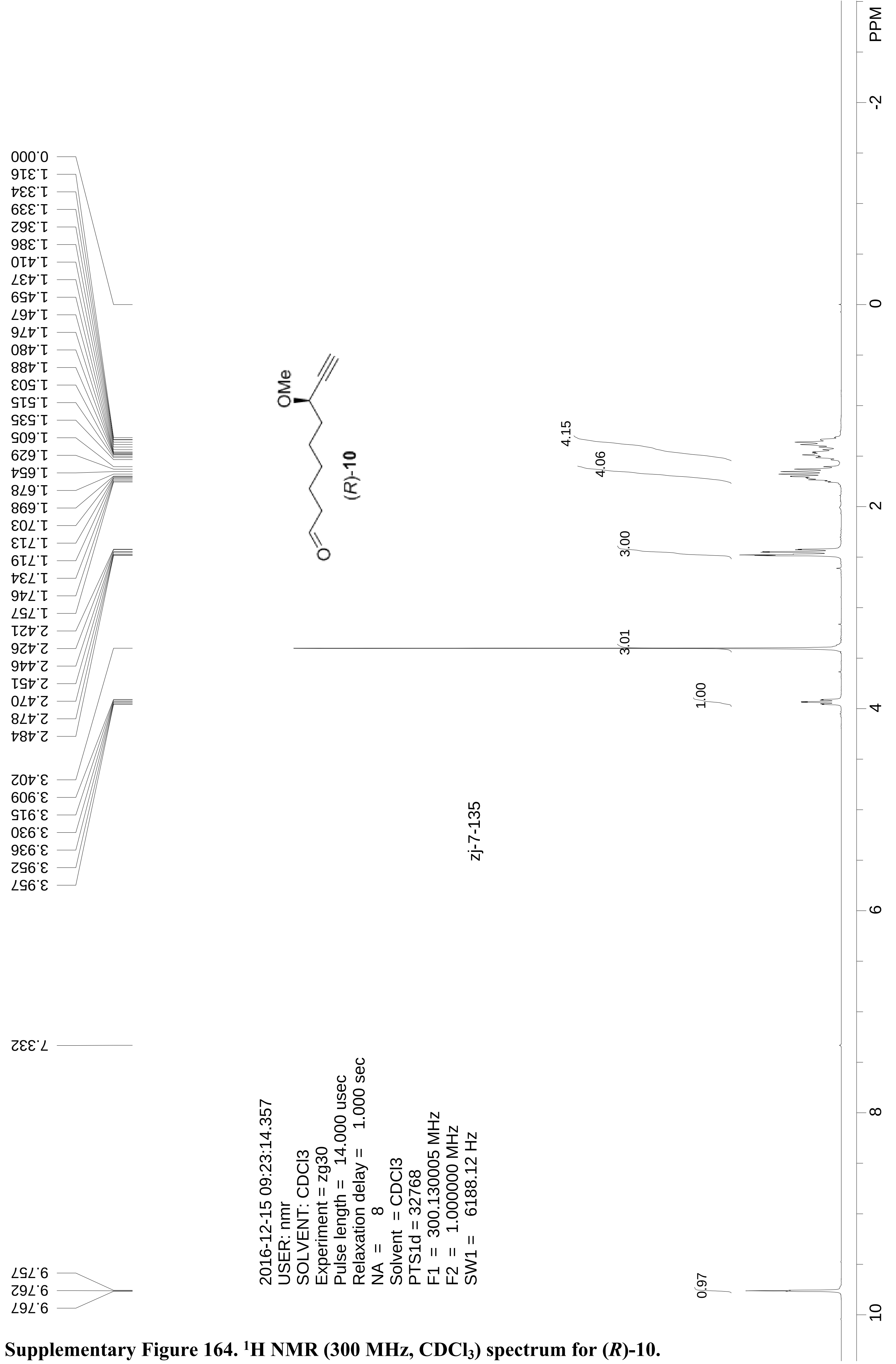

Supplementary Figure 164. ^{1}H NMR (300 MHz, $CDCl_3$) spectrum for (*R*)-10.

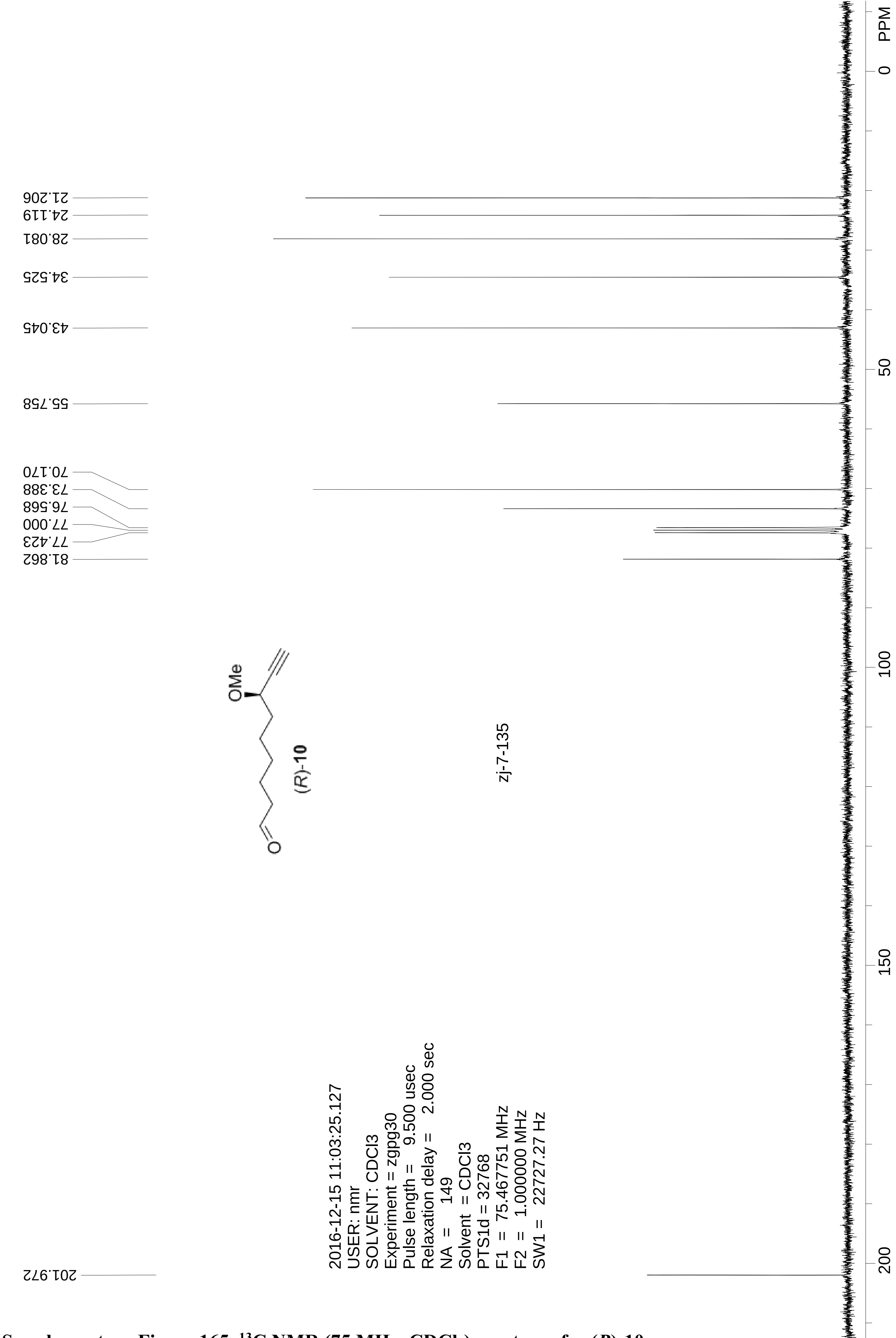

Supplementary Figure 165. **^{13}C NMR (75 MHz, $CDCl_3$) spectrum for (*R*)-10.**

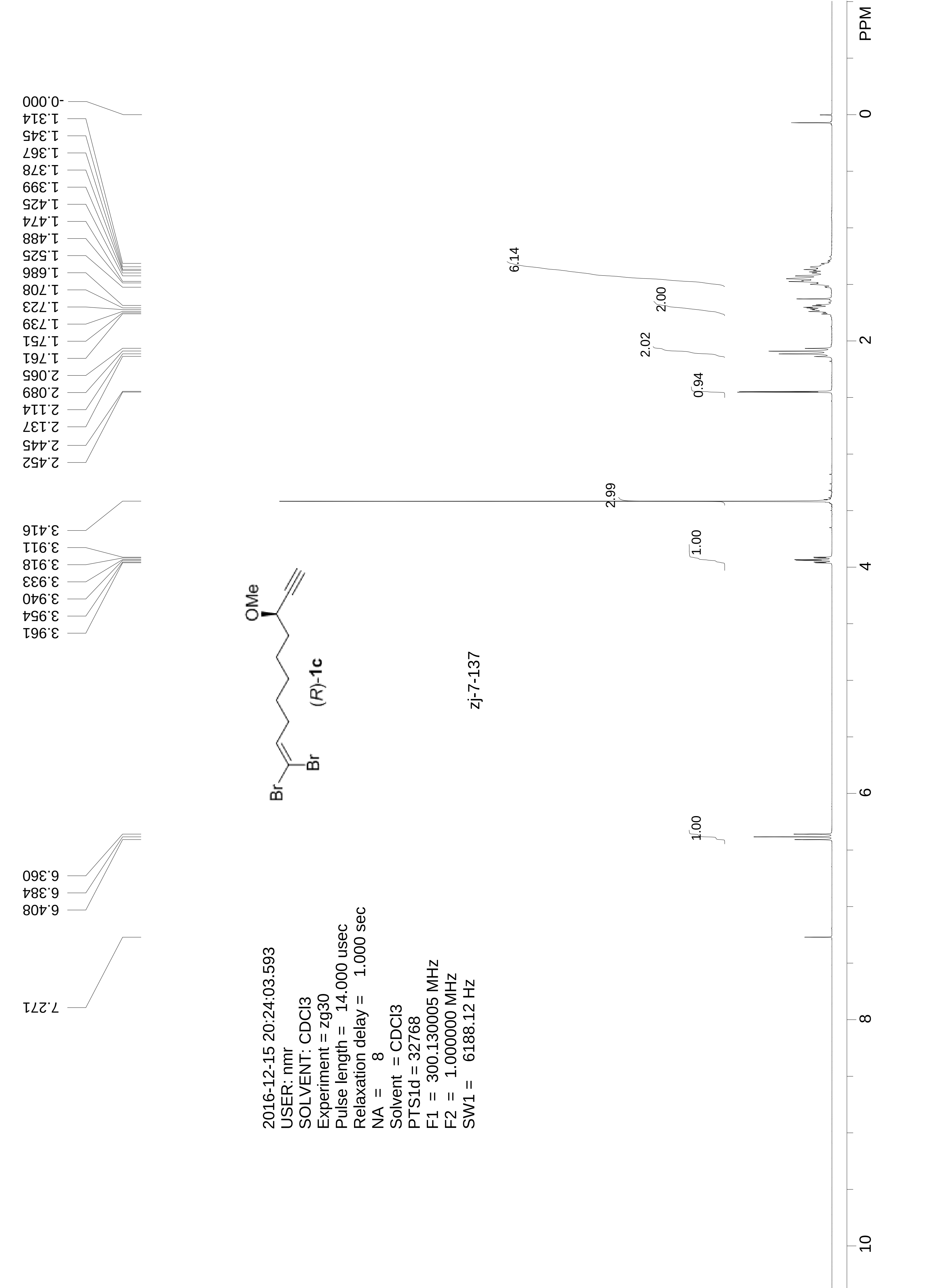

Supplementary Figure 166. ^{1}H NMR (300 MHz, $CDCl_3$) spectrum for (*R*)-1c.

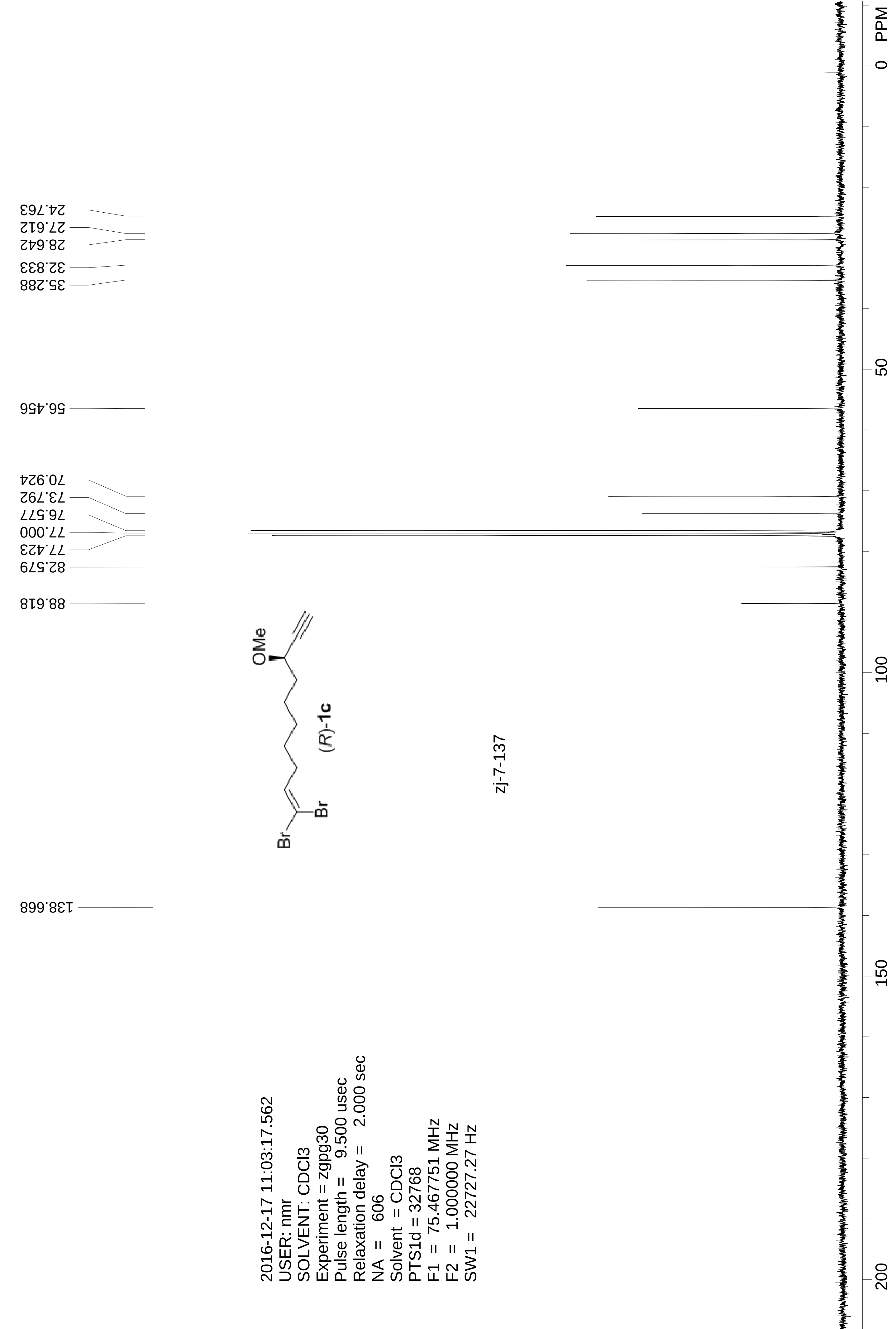

Supplementary Figure 167. ^{13}C NMR (75 MHz, $CDCl_3$) spectrum for (*R*)-1c.

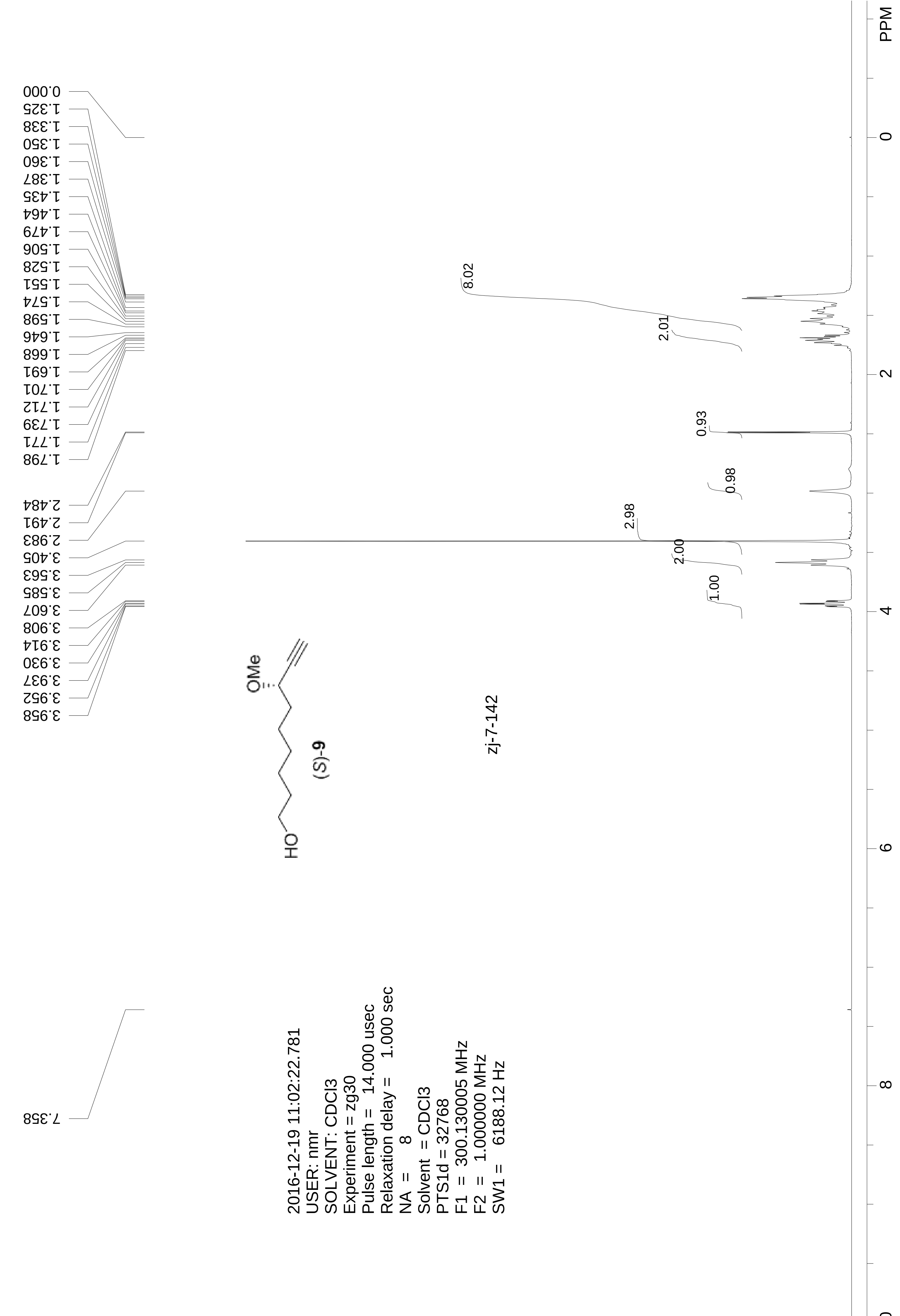

Supplementary Figure 168. ^{1}H NMR (300 MHz, $CDCl_3$) spectrum for (*S*)-9.

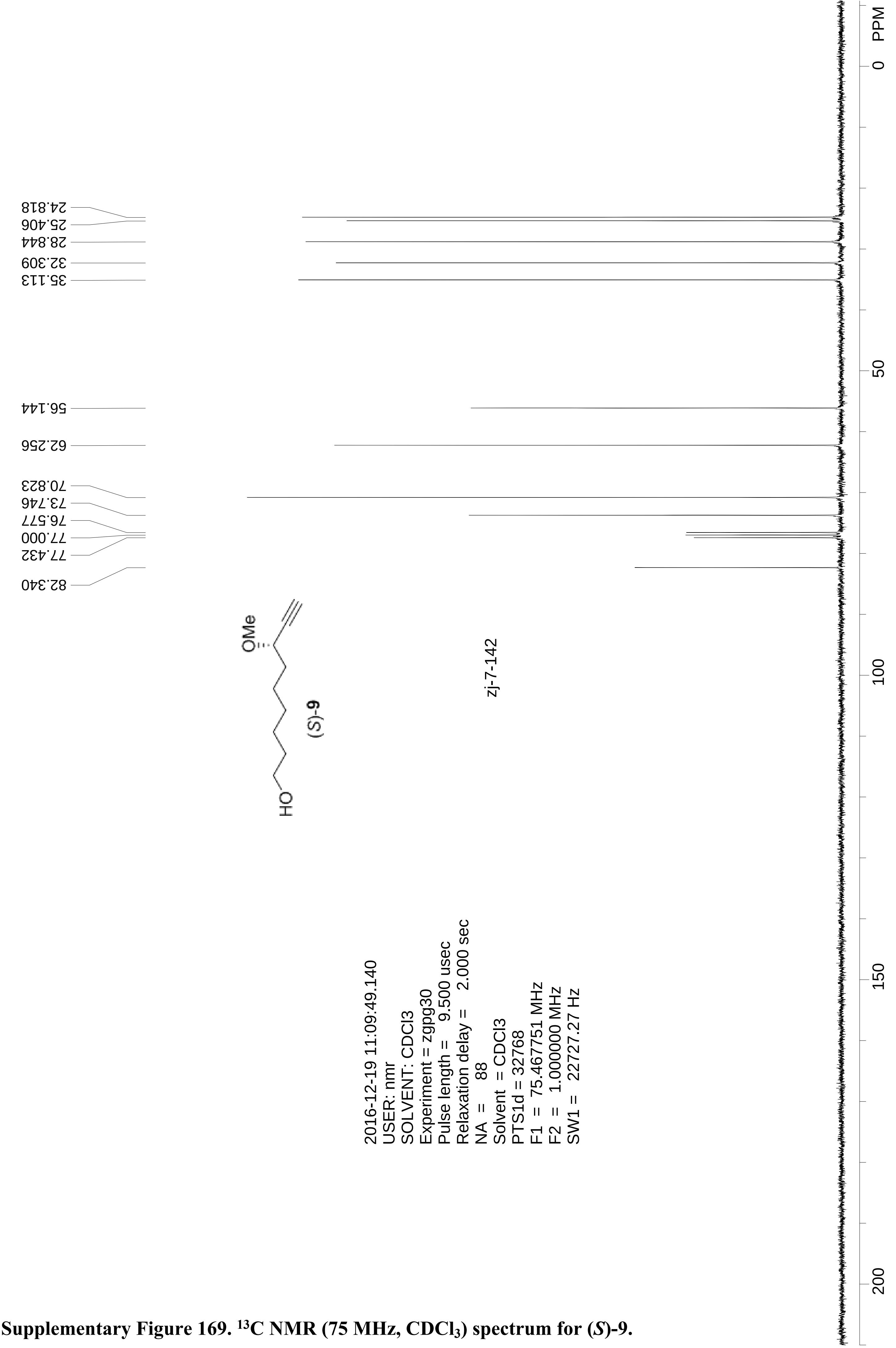

Supplementary Figure 169. ^{13}C NMR (75 MHz, $CDCl_3$) spectrum for (*S*)-9.

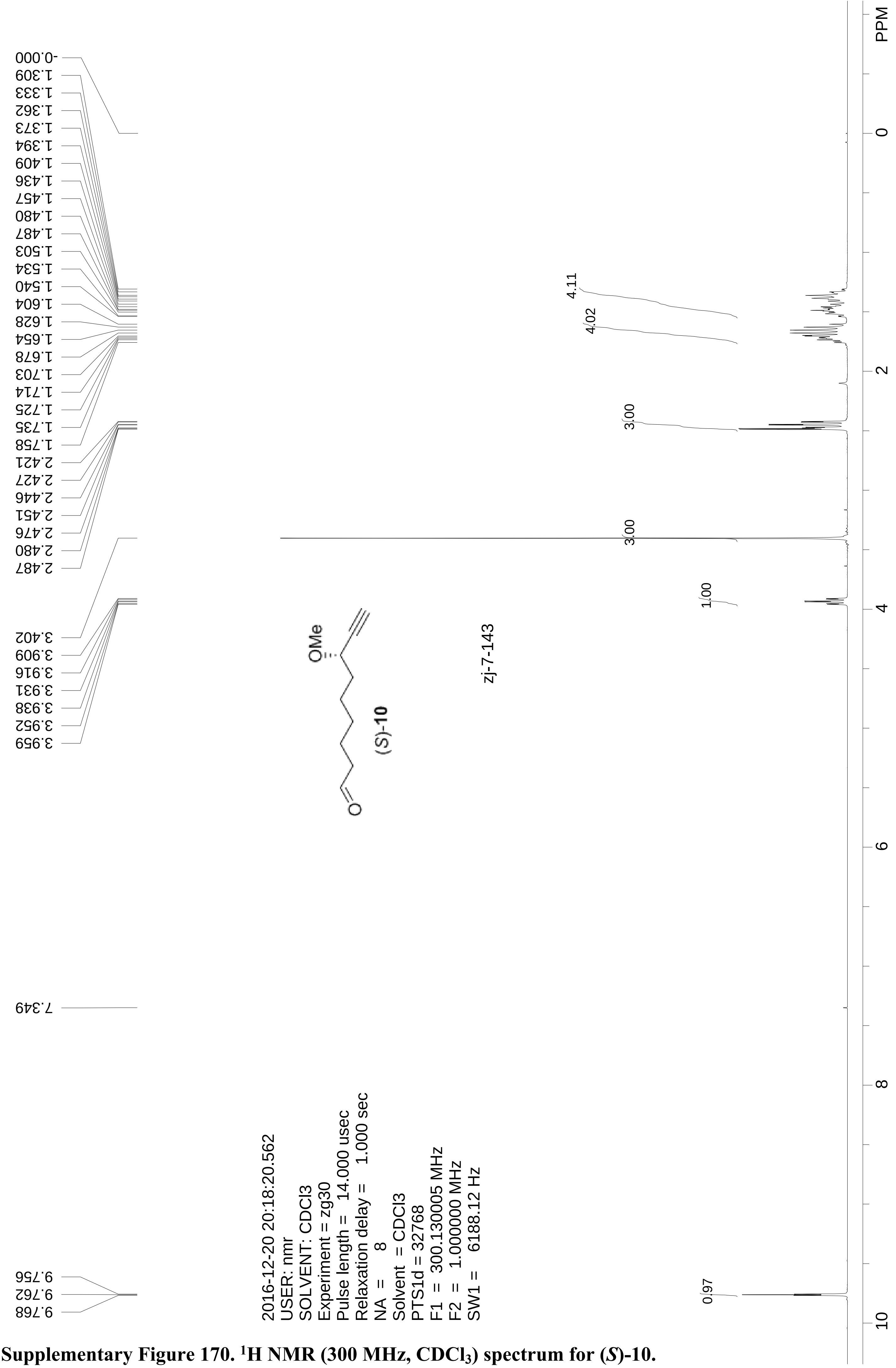

Supplementary Figure 170. ^{1}H NMR (300 MHz, $CDCl_3$) spectrum for (*S*)-10.

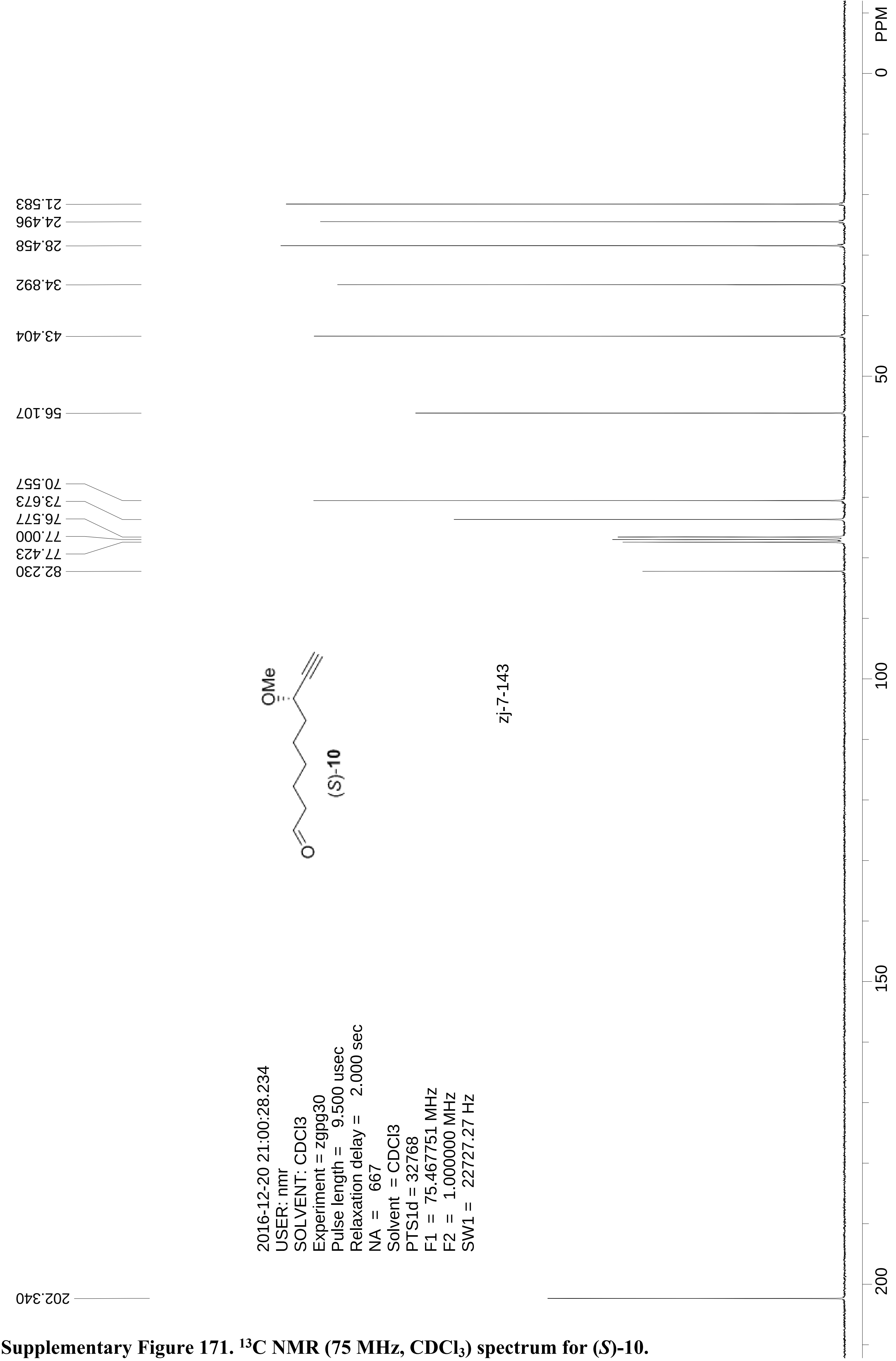

Supplementary Figure 171. **^{13}C NMR (75 MHz, $CDCl_3$) spectrum for (*S*)-10.**

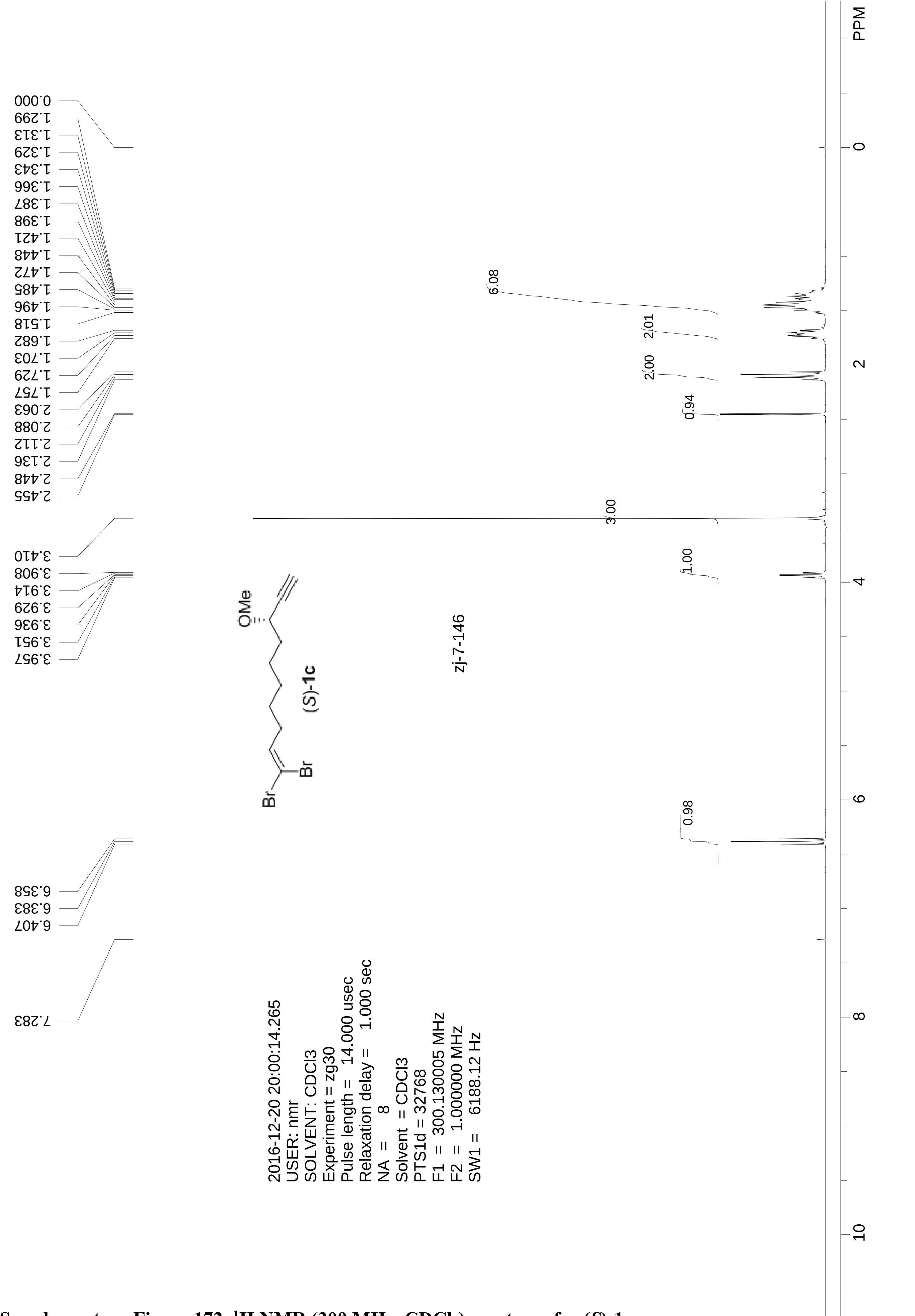

Supplementary Figure 172. ^{1}H NMR (300 MHz, $CDCl_3$) spectrum for (*S*)-1c.

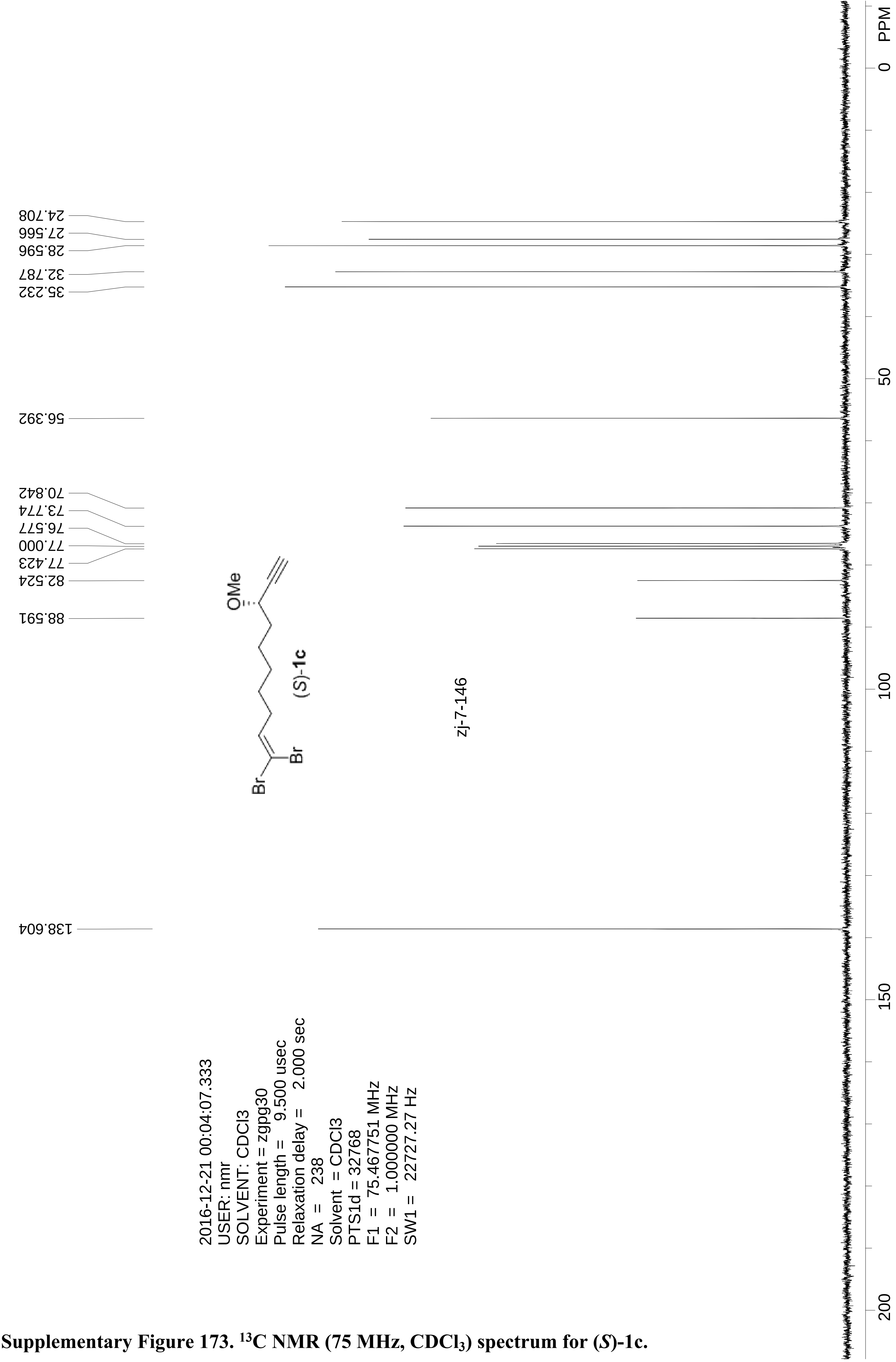

Supplementary Figure 173. ^{13}**C NMR (75 MHz, CDCl$_3$) spectrum for (*S*)-1c.**

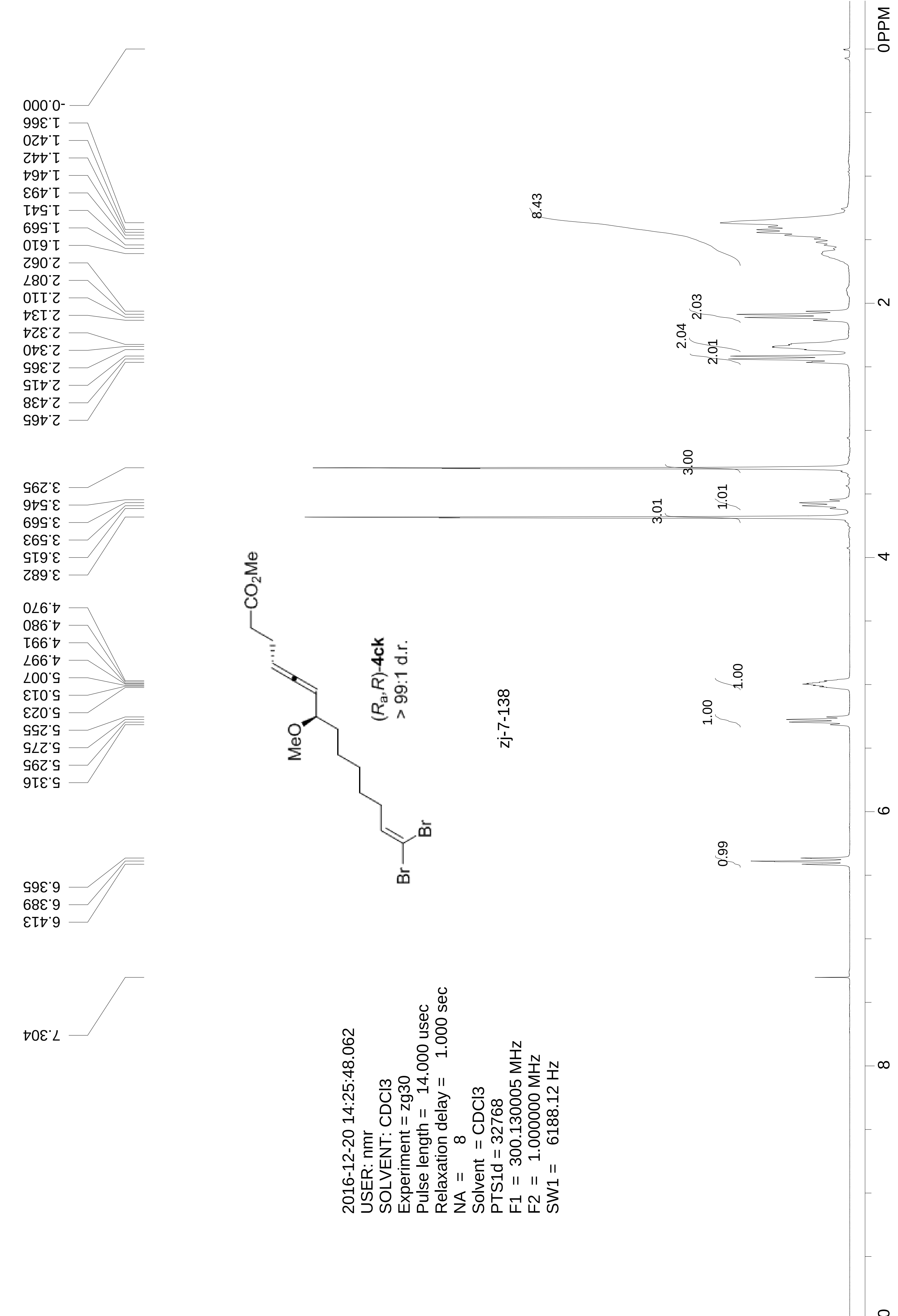

Supplementary Figure 174. ^{1}H NMR (300 MHz, $CDCl_3$) spectrum for (R_a,R)-4ck.

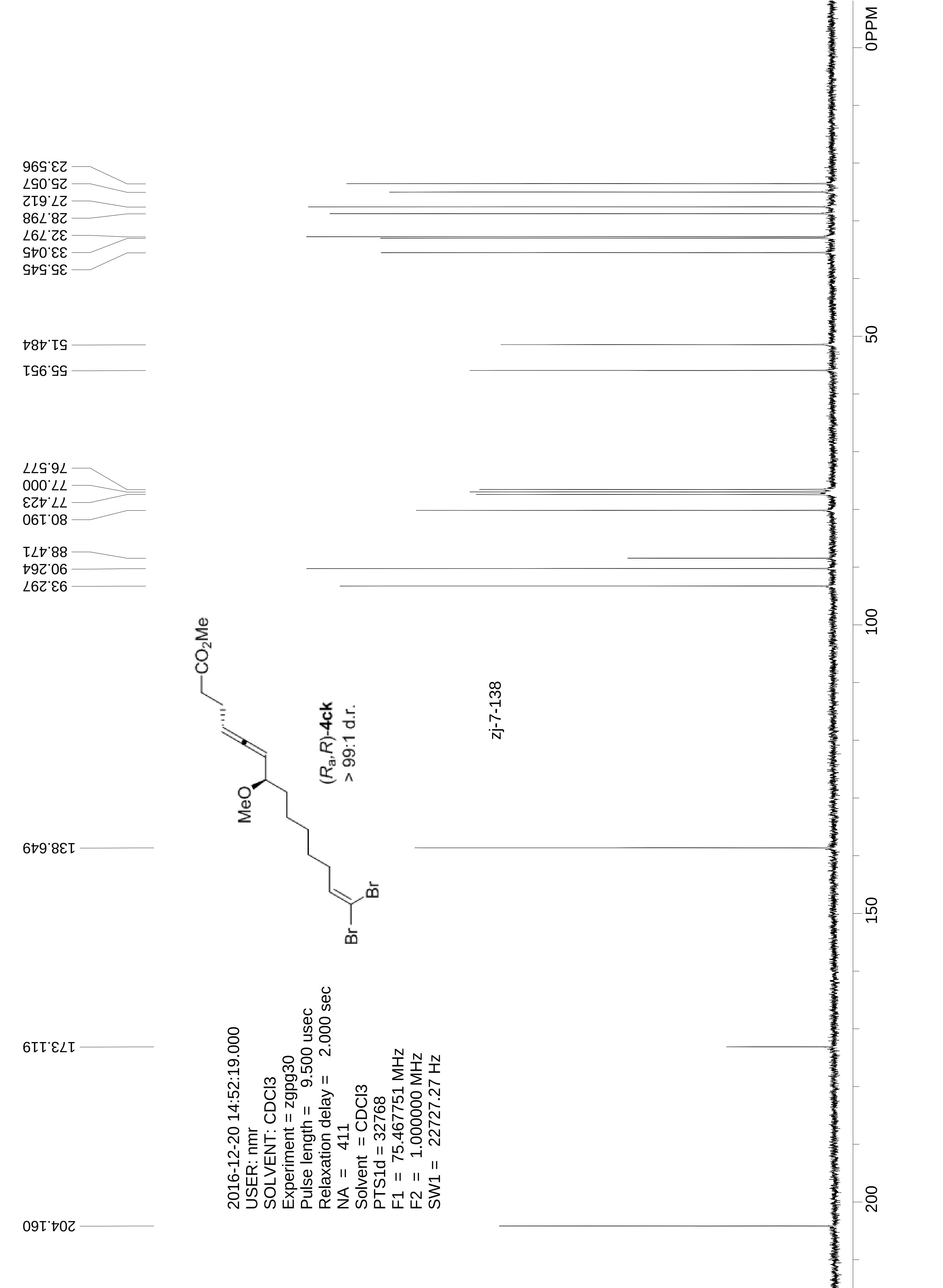

Supplementary Figure 175. **^{13}C NMR (75 MHz, CDCl$_3$) spectrum for (*R*$_a$,*R*)-4ck.**

zj-7-138-if-100-1-1-214

实验时间：2017-03-02, 12:21:23　　　　报告时间：2017-03-02, 18:32:20
谱图文件:D:\zhuguangjiong\zj\20170302\zj-7-138-if-100-1-1-214.org

实验内容简介：

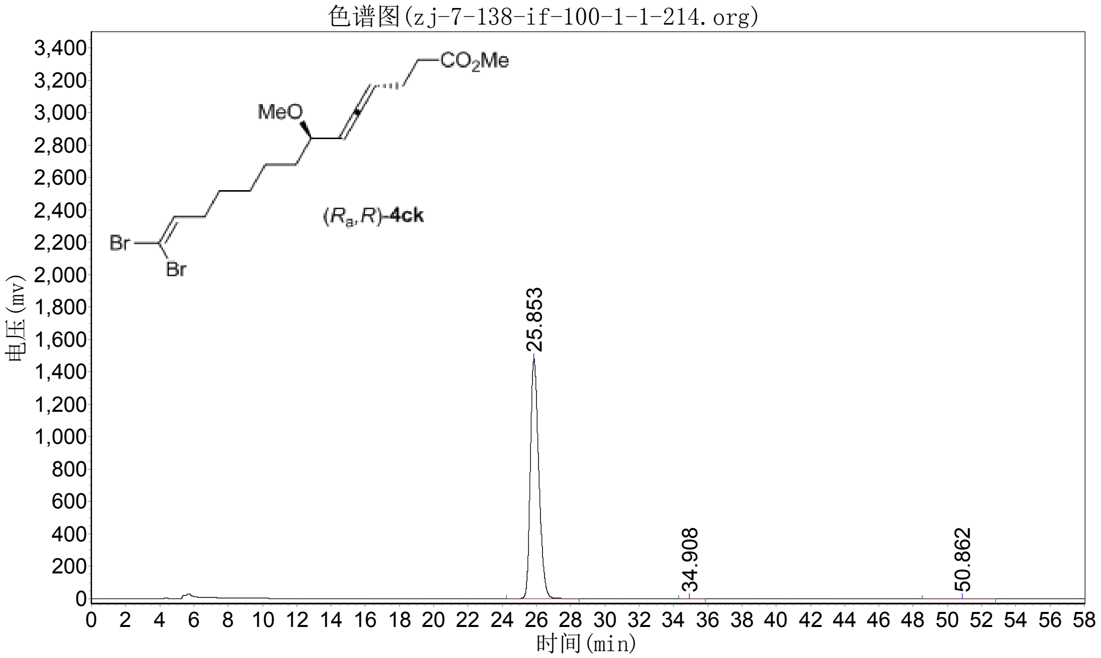

分析结果表

峰号	峰名	保留时间	峰高	峰面积	含量
1		25.853	1479945.500	51123048.000	99.5423
2		34.908	950.974	43397.344	0.0845
3		50.862	1857.505	191656.438	0.3732
总计			1482753.979	51358101.781	100.0000

Supplementary Figure 176. HPLC spectrum for (*R*$_a$,*R*)-4ck.

zj-7-114-if-100-1-1-214

实验时间：2017-03-02, 11:12:11 报告时间：2017-03-02, 18:33:43
谱图文件:D:\zhuguangjiong\zj\20170302\zj-7-114-if-100-1-1-214.org

实验内容简介：

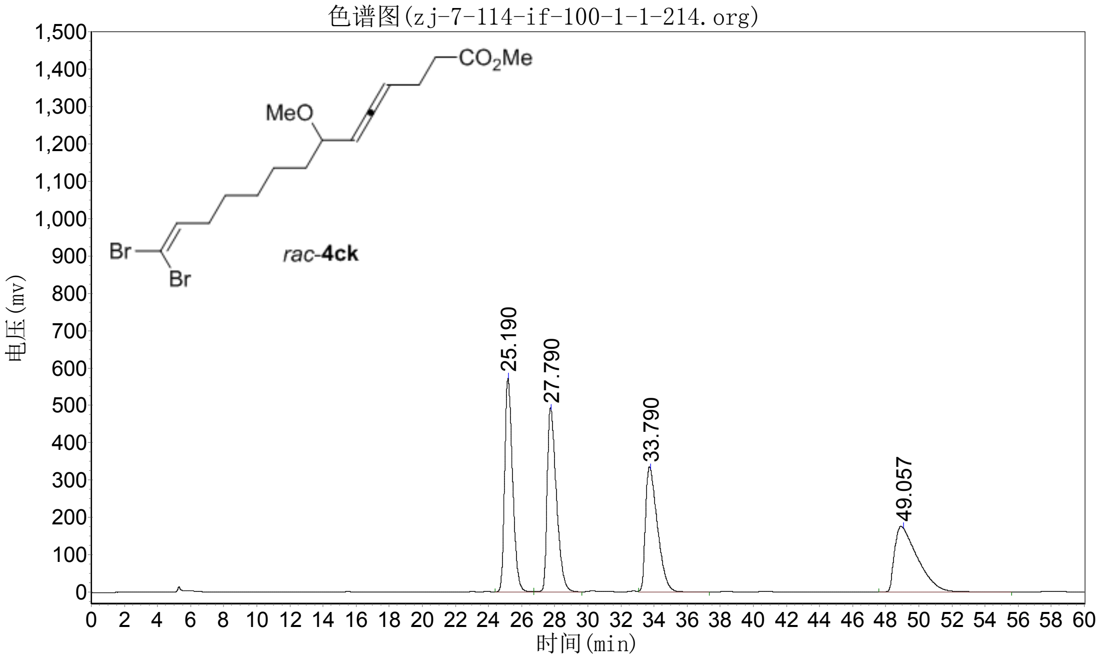

分析结果表

峰号	峰名	保留时间	峰高	峰面积	含量
1		25.190	568287.688	19152964.000	26.5667
2		27.790	492501.188	19221222.000	26.6613
3		33.790	333279.406	16885436.000	23.4214
4		49.057	173895.328	16834350.000	23.3506
总计			1567963.609	72093972.000	100.0000

Supplementary Figure 177. HPLC spectrum for *rac*-4ck.

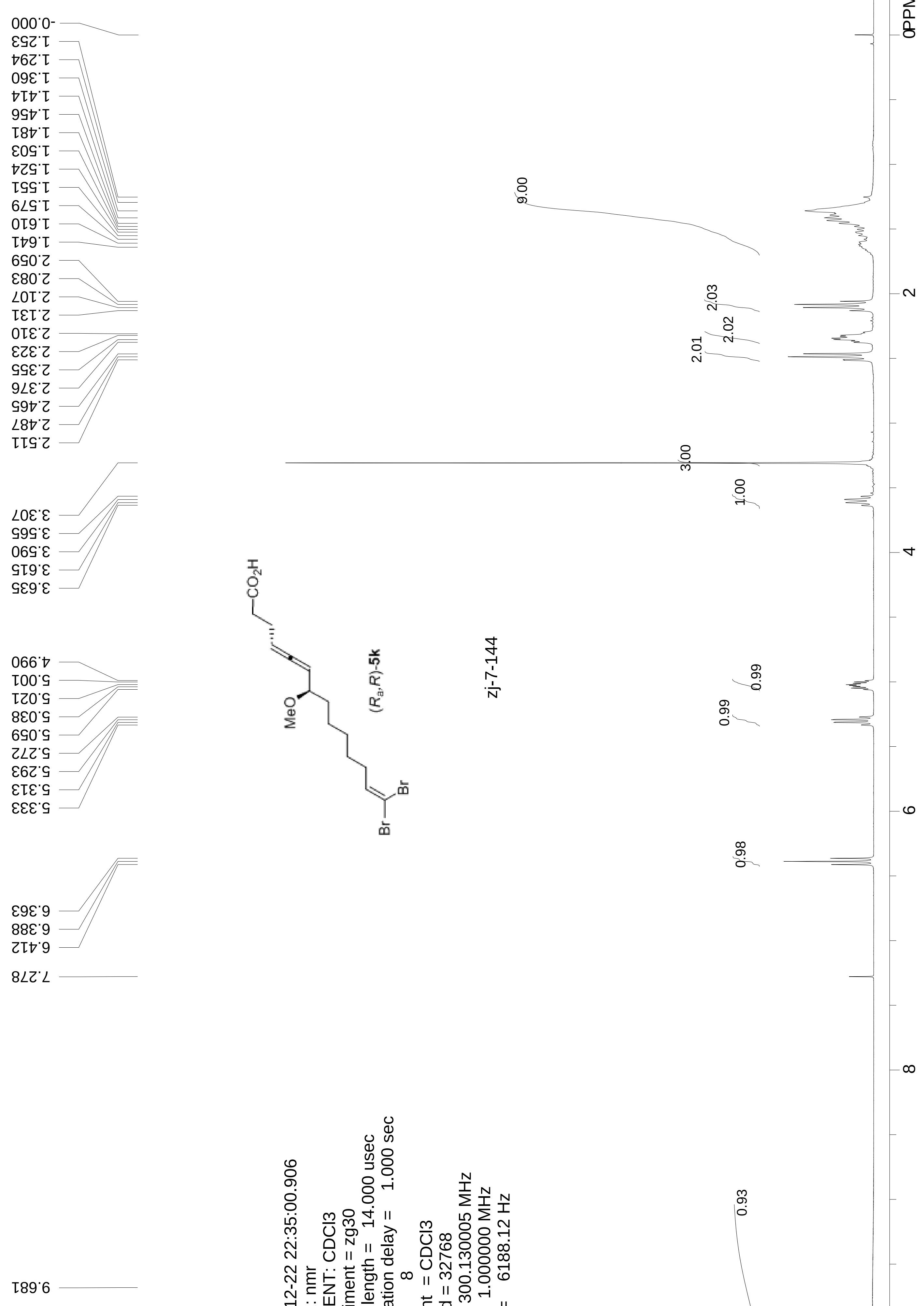

Supplementary Figure 178. **^{1}H NMR (300 MHz, $CDCl_3$) spectrum for (R_a,R)-5k.**

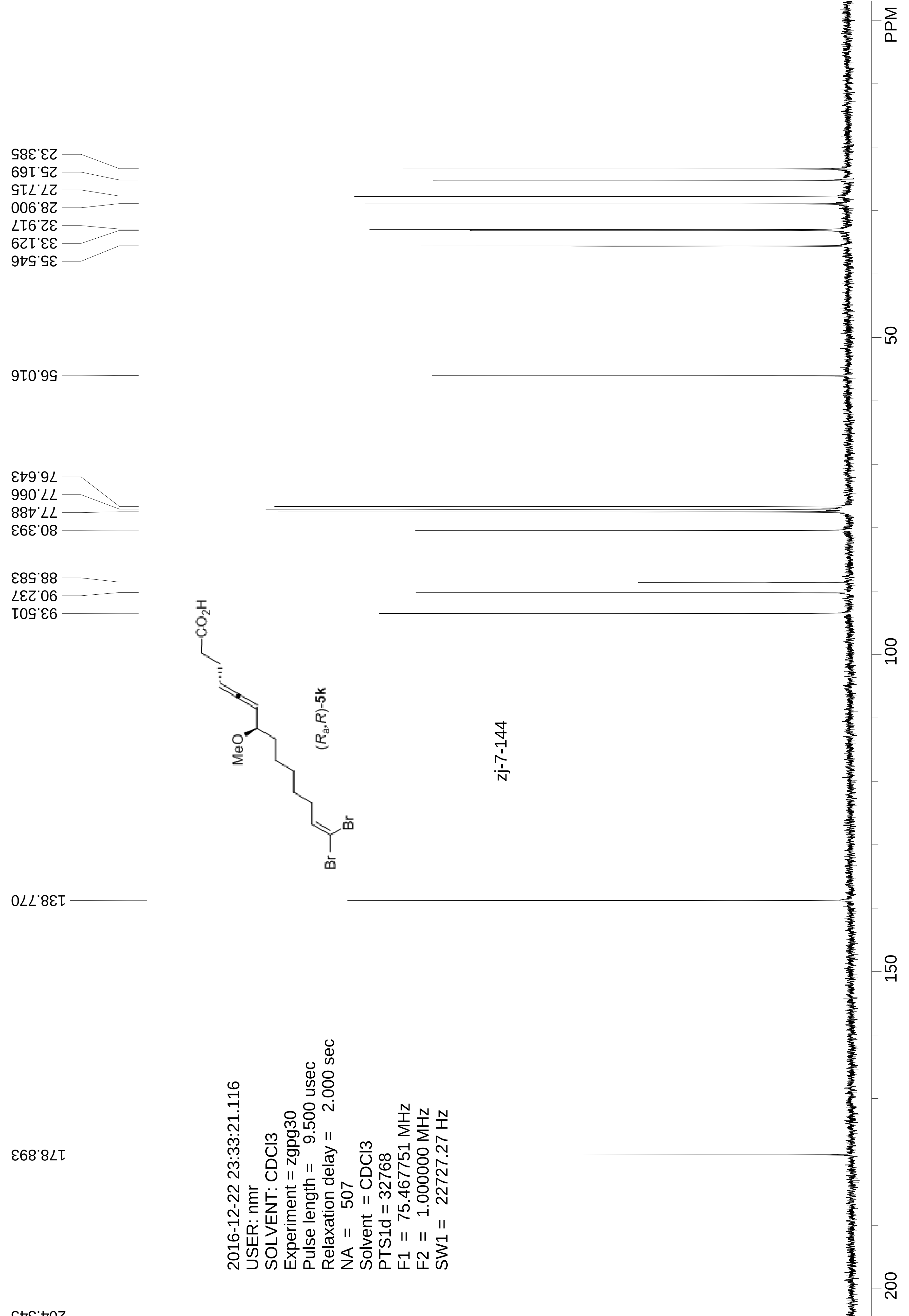

Supplementary Figure 179. ^{13}C NMR (75 MHz, $CDCl_3$) spectrum for (R_a,R)-5k.

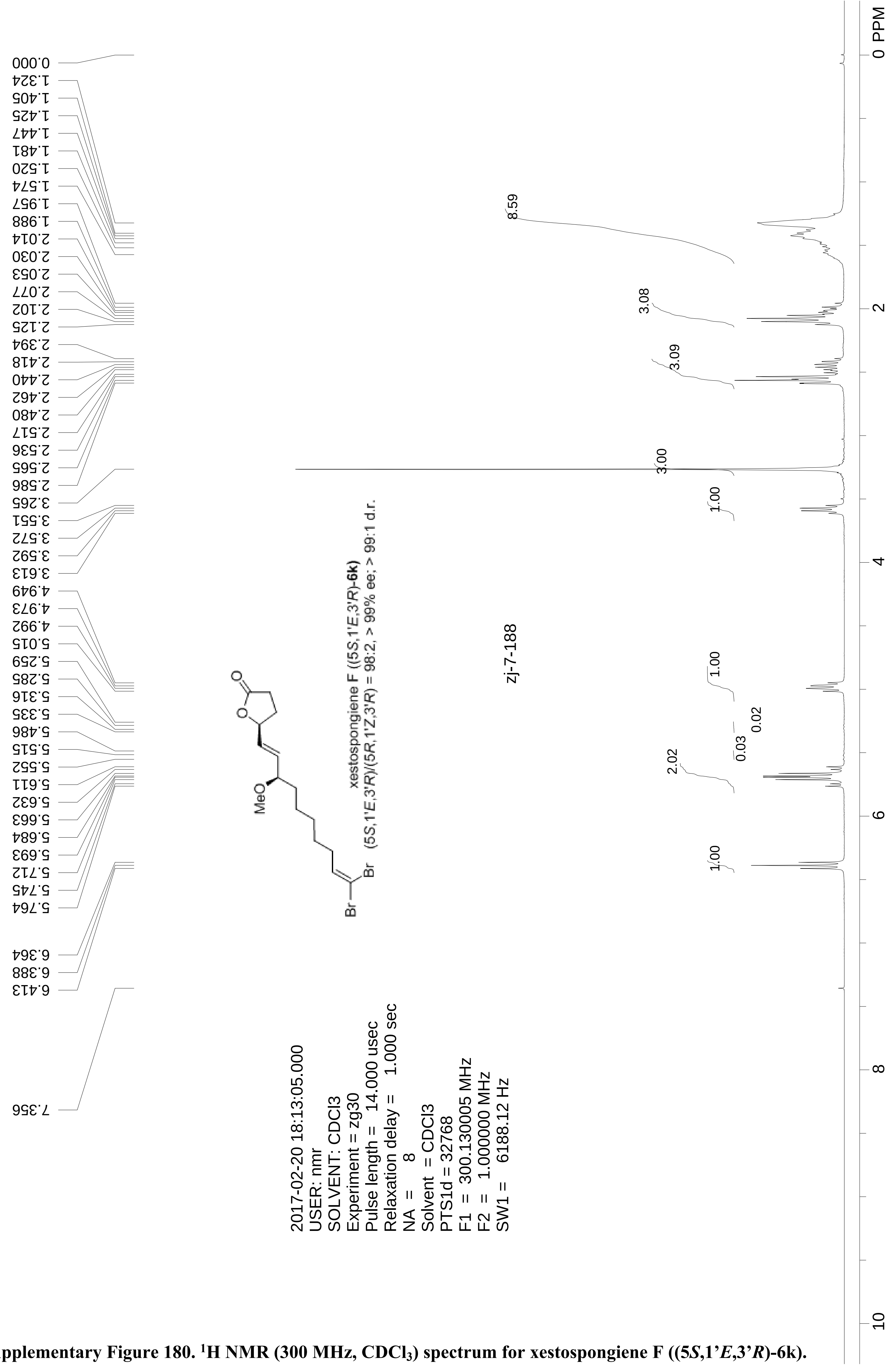

Supplementary Figure 180. ^{1}**H NMR (300 MHz, CDCl$_3$) spectrum for xestospongiene F ((5*S*,1'*E*,3'*R*)-6k).**

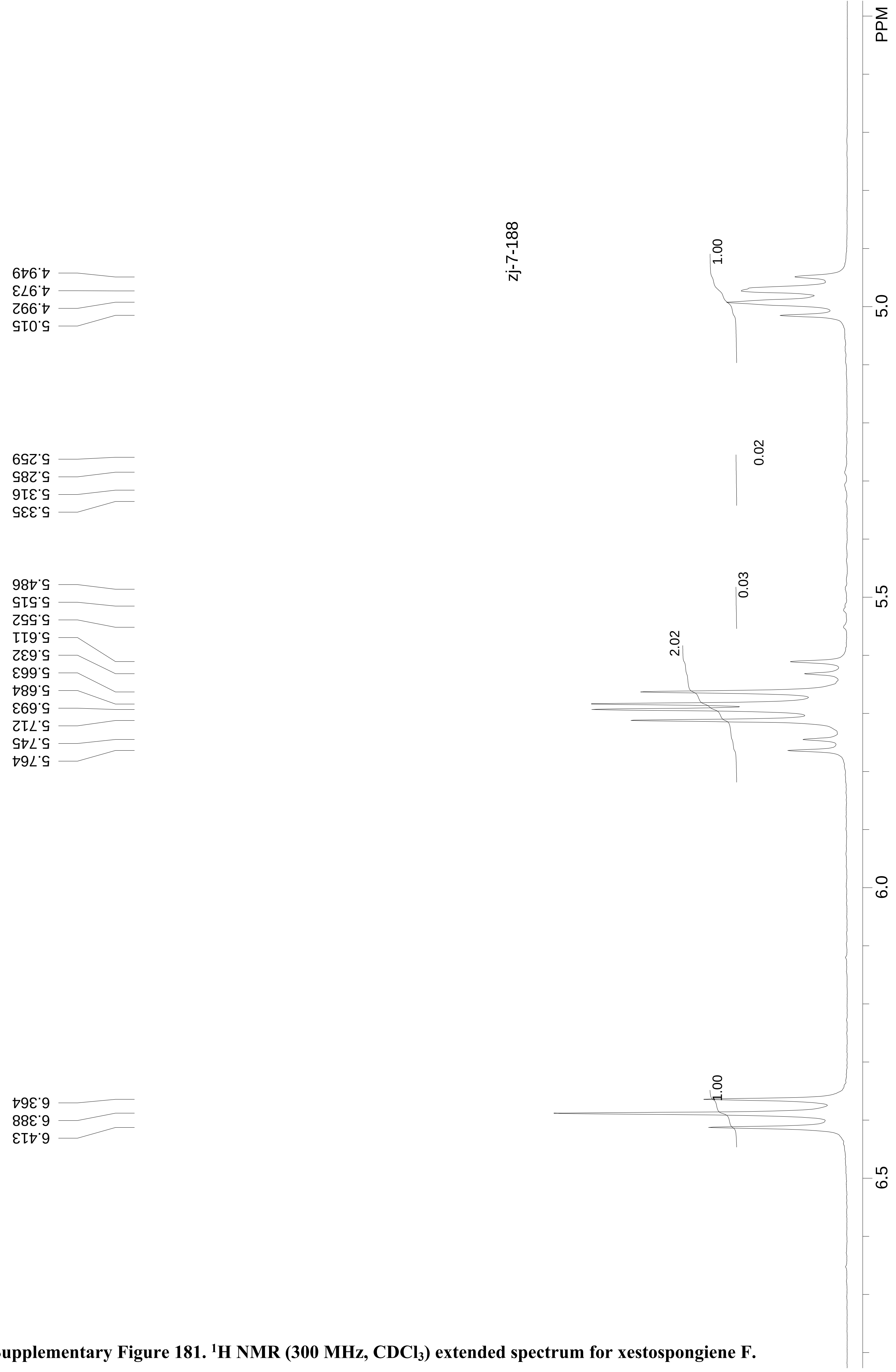

Supplementary Figure 181. ^{1}H NMR (300 MHz, $CDCl_3$) extended spectrum for xestospongiene F.

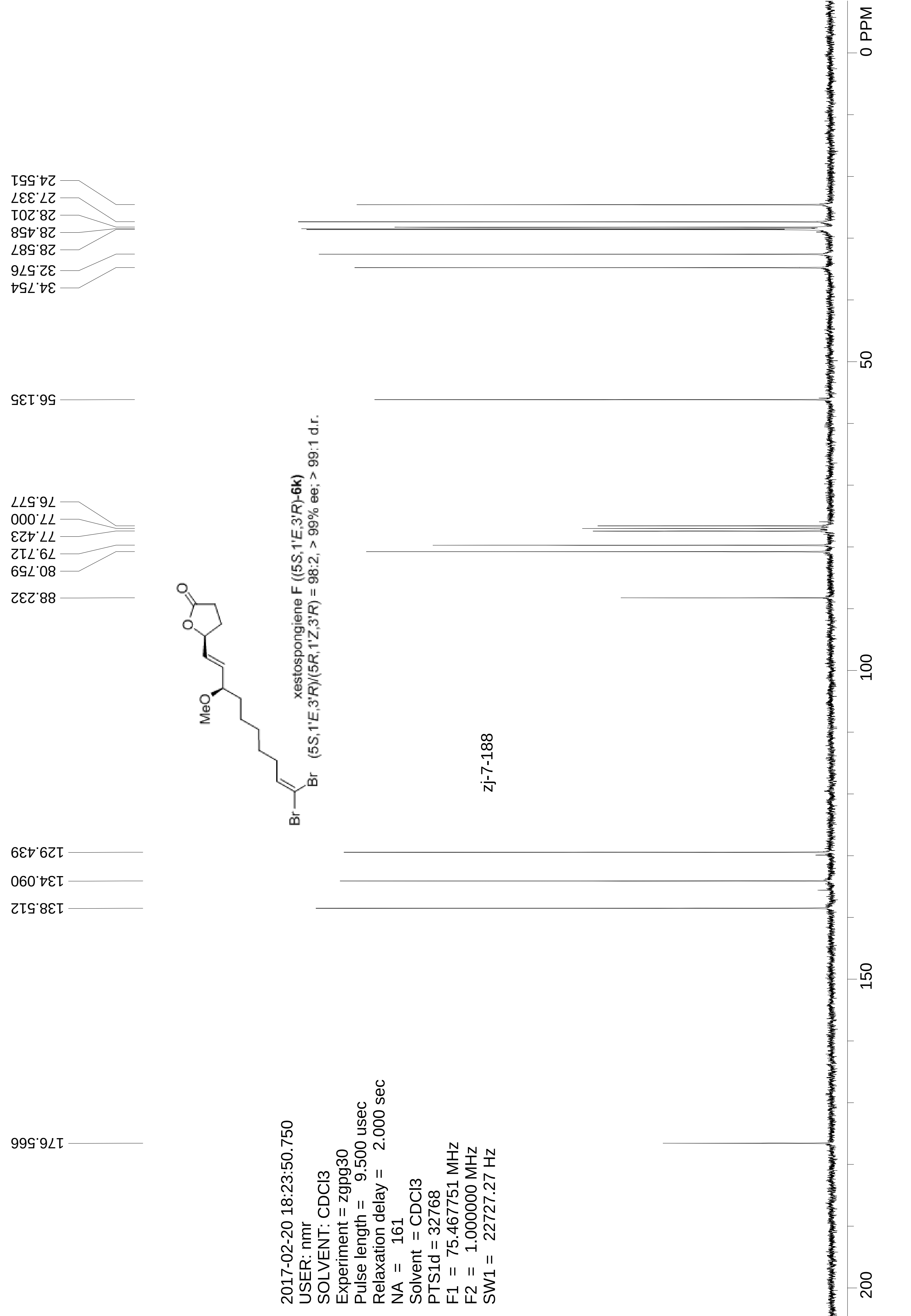

Supplementary Figure 182. ^{13}**C NMR (75 MHz, CDCl$_3$) spectrum for xestospongiene F ((5*S*,1'*E*,3'*R*)-6k).**

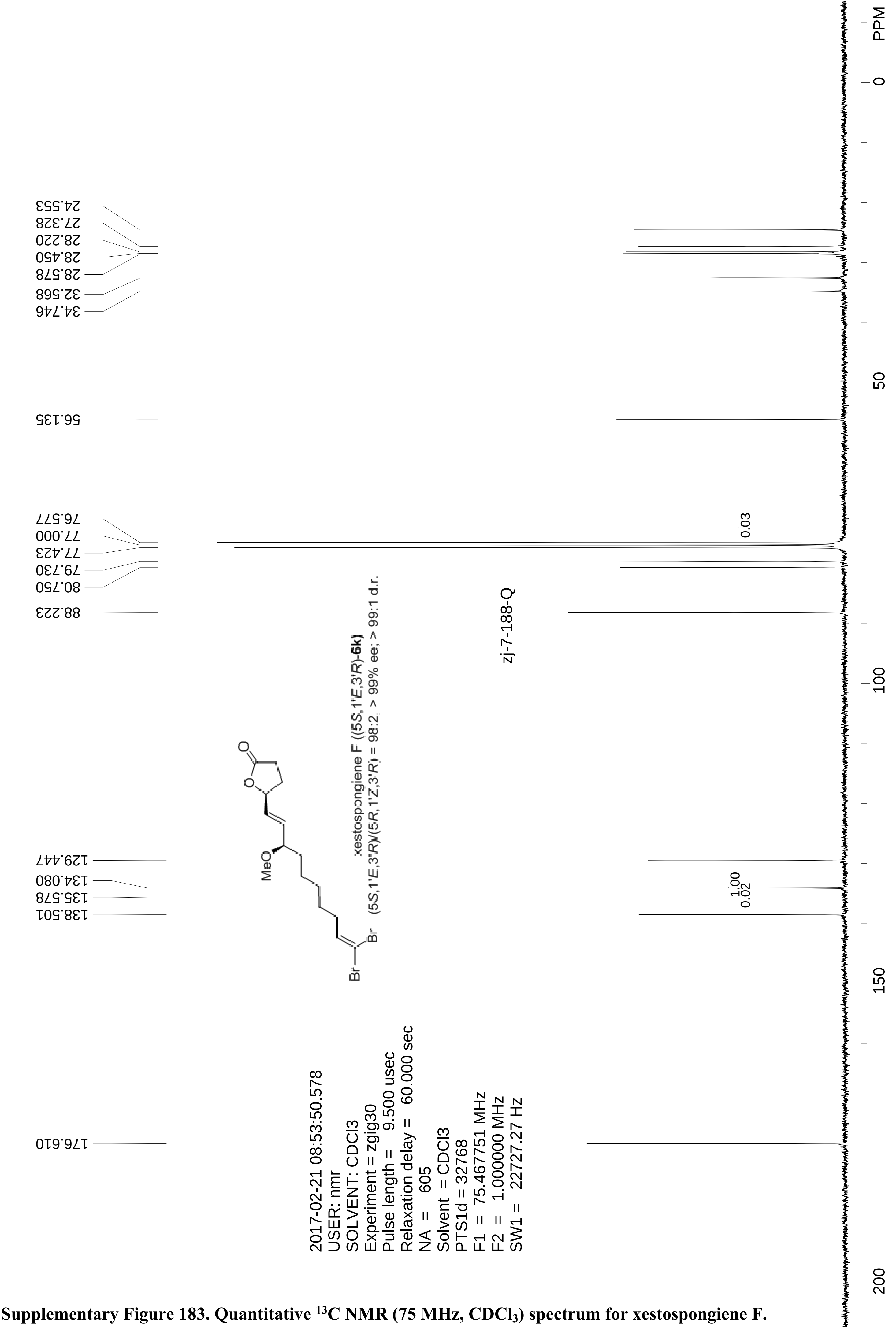

Supplementary Figure 183. Quantitative ^{13}C NMR (75 MHz, $CDCl_3$) spectrum for xestospongiene F.

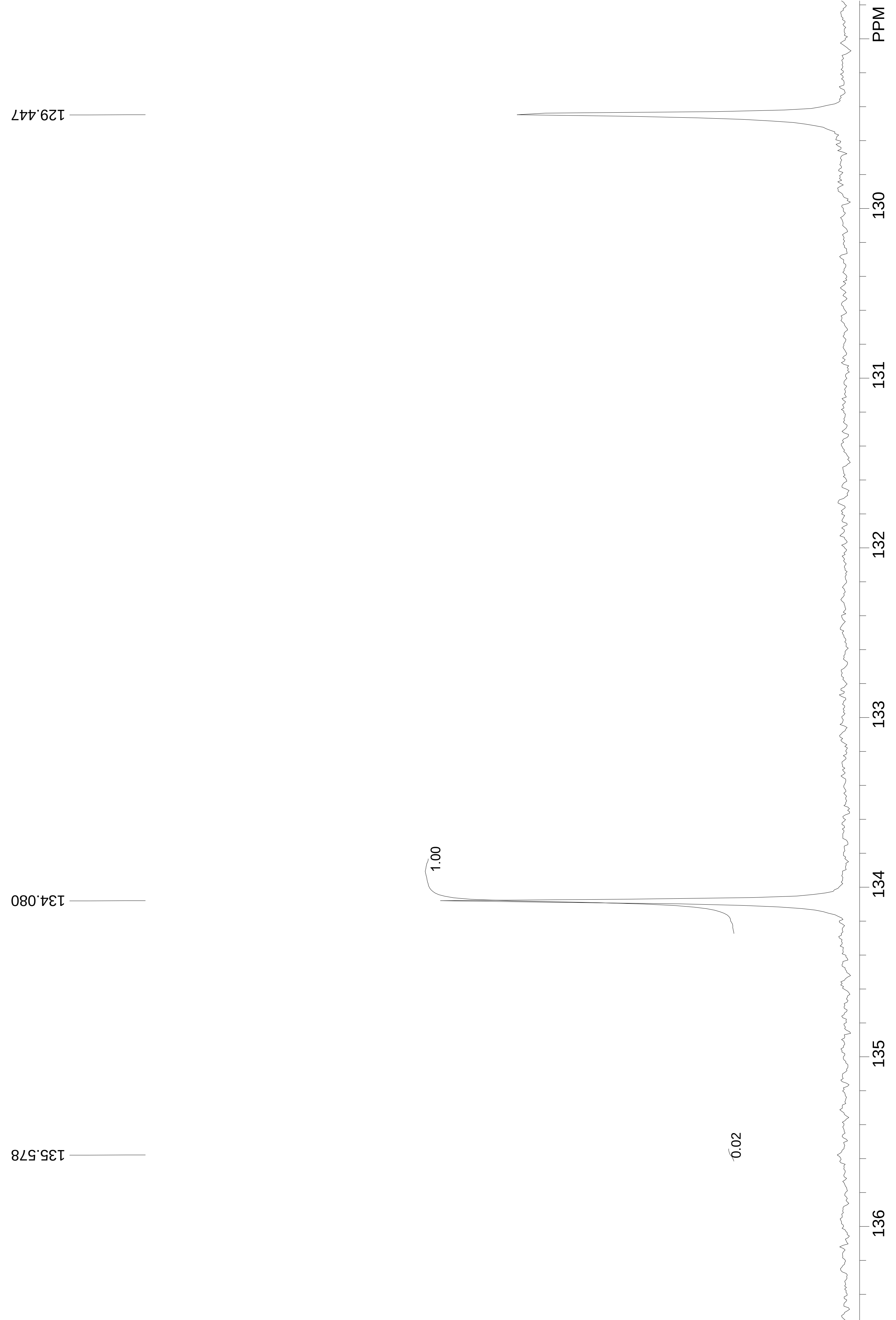

Supplementary Figure 184. Quantitative ^{13}C NMR (75 MHz, $CDCl_3$) extended spectrum for xestospongiene F.

Data File C:\CHEM32\1\DATA\2014-44651.D
Sample Name: zj-7-188

===

Acq. Operator : 系统
Sample Operator : 系统
Acq. Instrument : SFC   Location : Vial 46
Injection Date : 10/03/2017 15:47:57
Inj Volume : 5.000 µl
Acq. Method : C:\CHEM32\1\METHODS\DEF_LC-TEST-2016.M
Last changed : 10/03/2017 15:12:15 by 系统
(modified after loading)
Analysis Method : C:\CHEM32\1\METHODS\DEF_LC-TEST-2016.M
Last changed : 13/03/2017 09:52:48 by 系统
(modified after loading)
Additional Info : Peak(s) manually integrated

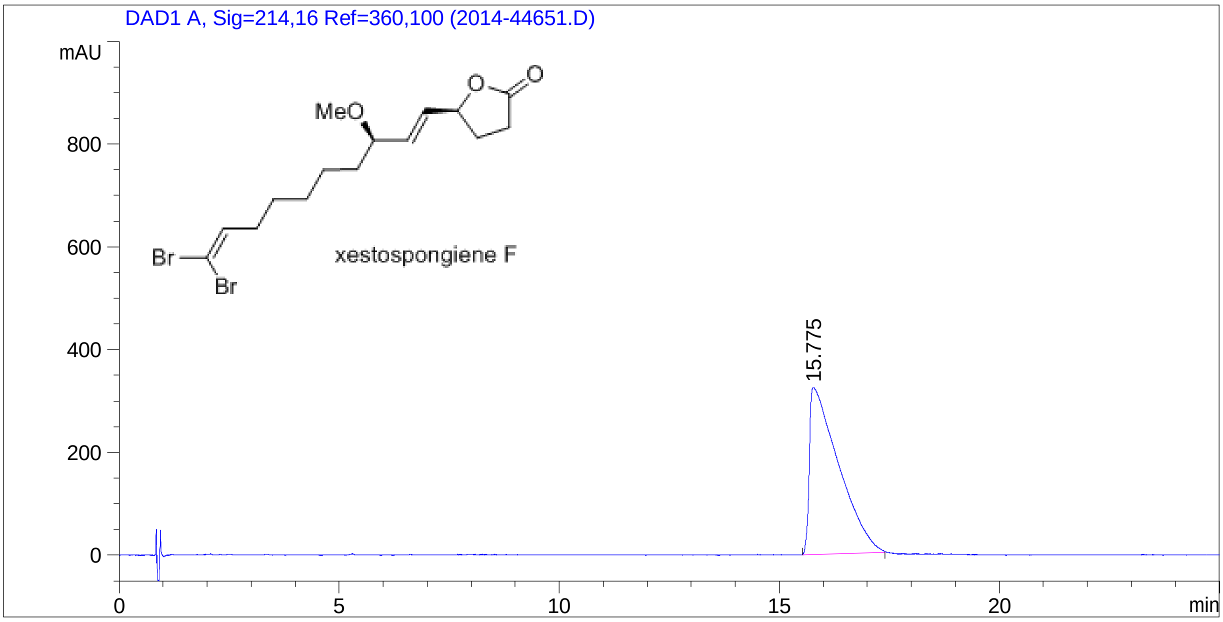

===

Area Percent Report

===

Sorted By : Signal
Multiplier : 1.0000
Dilution : 1.0000
Do not use Multiplier & Dilution Factor with ISTDs

Signal 1: DAD1 A, Sig=214,16 Ref=360,100

Peak #	RetTime [min]	Type	Width [min]	Area [mAU*s]	Height [mAU]	Area %
1	15.775	VV	0.5656	1.49946e4	325.08957	100.0000

Totals : 1.49946e4 325.08957

===

*** End of Report ***

Supplementary Figure 185. SFC spectrum for xestospongiene F.

Data File C:\CHEM32\1\DATA\2014-44652.D
Sample Name: zj-7-158-rac-ad-h-sfc-97-3-1.3-214

===

Acq. Operator : 系统
Sample Operator : 系统
Acq. Instrument : SFC Location : Vial 41
Injection Date : 10/03/2017 16:22:52
Inj Volume : 5.000 µl
Acq. Method : C:\CHEM32\1\METHODS\DEF_LC-TEST-2016.M
Last changed : 10/03/2017 16:20:37 by 系统
(modified after loading)
Analysis Method : C:\CHEM32\1\METHODS\DEF_LC-TEST-2016.M
Last changed : 13/03/2017 09:50:59 by 系统
(modified after loading)
Additional Info : Peak(s) manually integrated

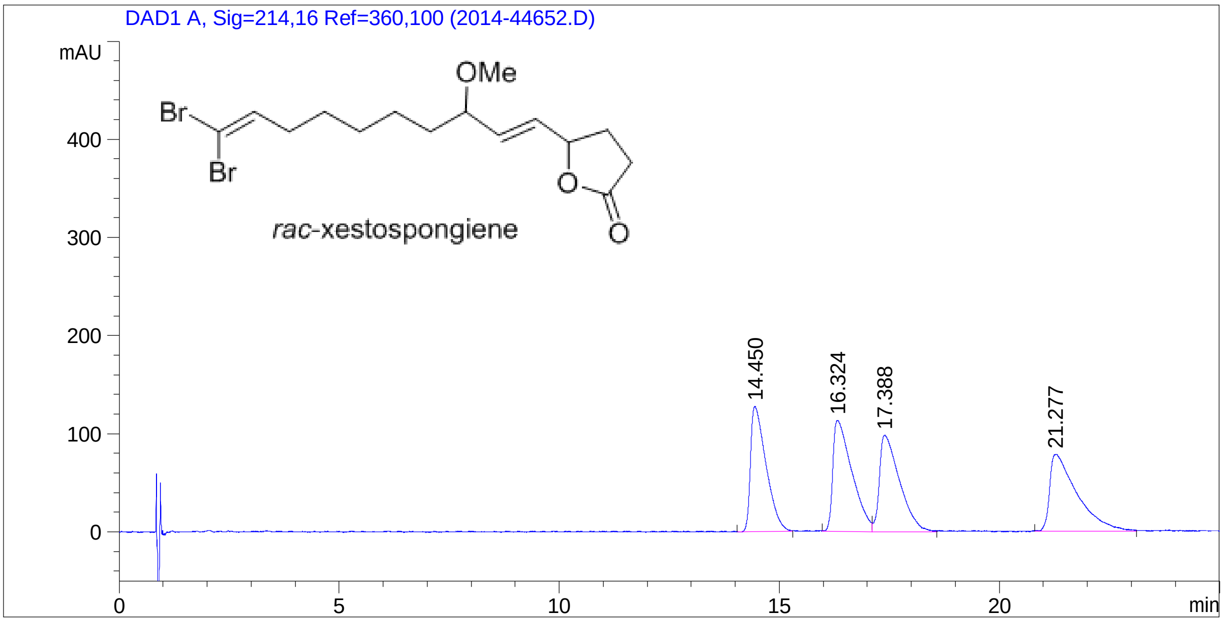

===

Area Percent Report

===

Sorted By : Signal
Multiplier : 1.0000
Dilution : 1.0000
Do not use Multiplier & Dilution Factor with ISTDs

Signal 1: DAD1 A, Sig=214,16 Ref=360,100

Peak #	RetTime [min]	Type	Width [min]	Area [mAU*s]	Height [mAU]	Area %
1	14.450	MM R	0.3913	3141.53149	127.69143	24.0477
2	16.324	MF R	0.4819	3396.79004	113.02892	26.0016
3	17.388	FM R	0.5002	3149.34937	98.45376	24.1075
4	21.277	MM R	0.6807	3376.07983	78.39977	25.8431

Totals : 1.30638e4 417.57388

===

*** End of Report ***

Supplementary Figure 186. SFC spectrum for *rac*-xestospongiene.

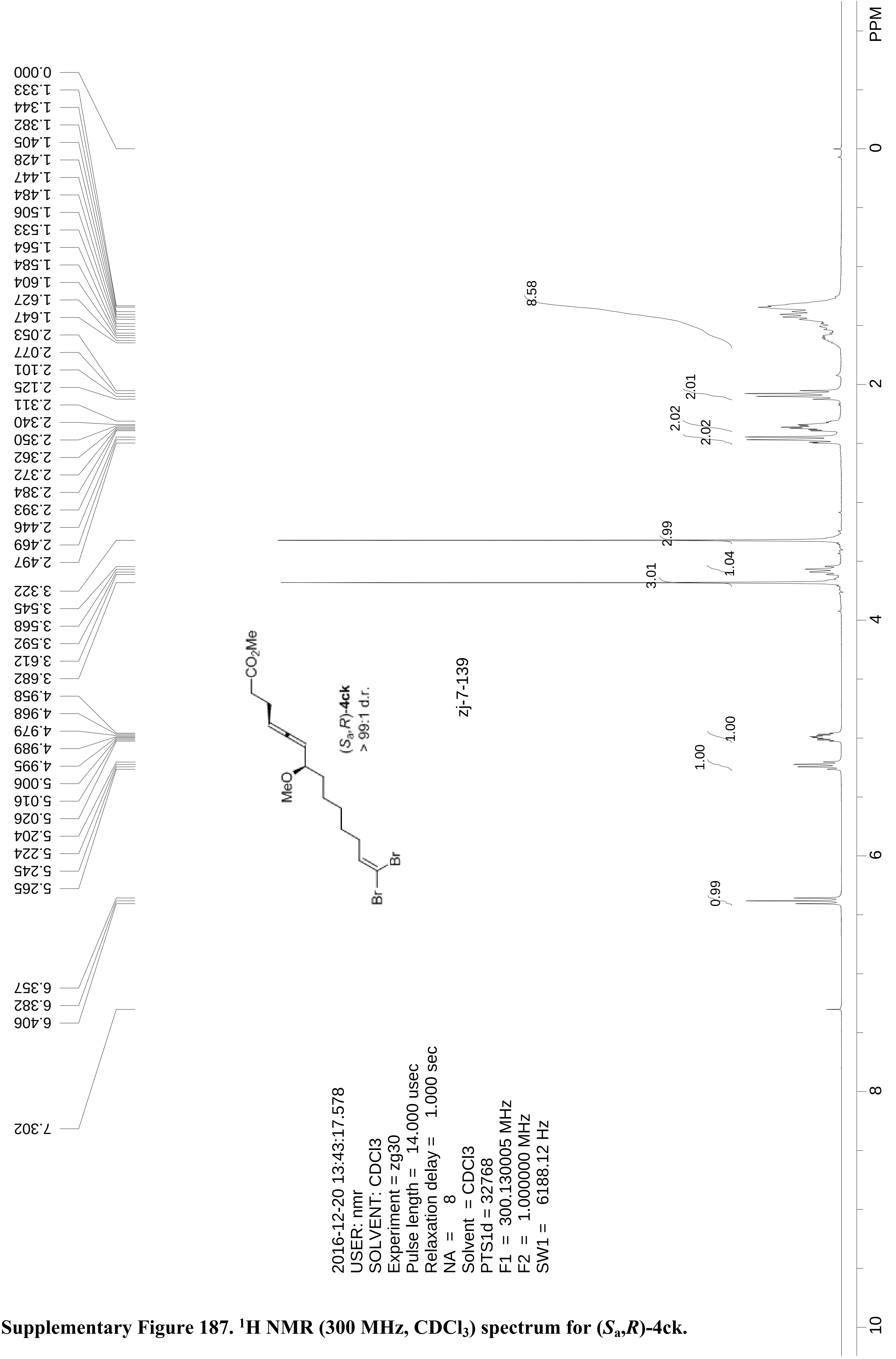

Supplementary Figure 187. ^{1}H NMR (300 MHz, CDCl$_3$) spectrum for (S_a,R)-4ck.

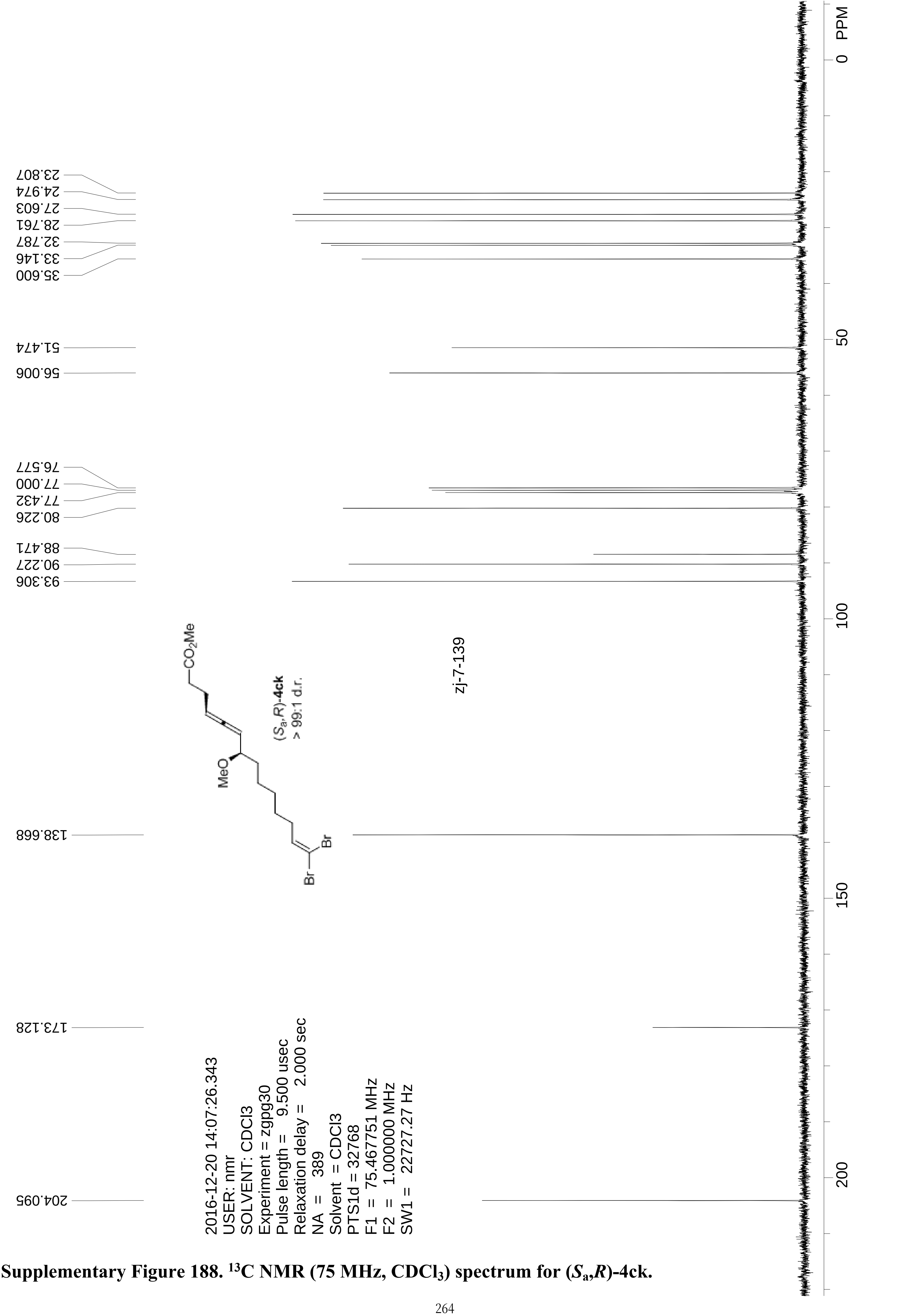

Supplementary Figure 188. ^{13}C NMR (75 MHz, $CDCl_3$) spectrum for (S_a,R)-4ck.

zj-7-139-if-100-1-1-214

实验时间：2016-12-29,16:30:56　　　　　　　　报告时间：2016-12-29,17:41:44
谱图文件:F:\zhuguangjiong\zj\20161229\zj-7-139-if-100-1-1-214.org

实验内容简介：

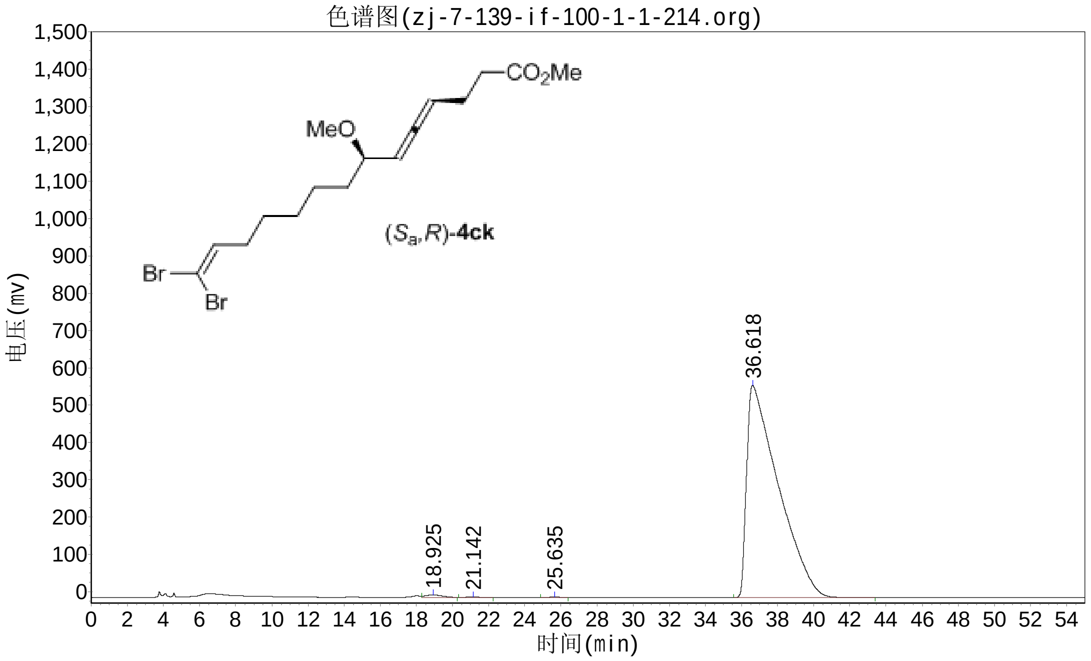

分析结果表

峰号	峰名	保留时间	峰高	峰面积	含量
1		18.925	7426.935	442951.219	0.6339
2		21.142	1433.861	62236.391	0.0891
3		25.635	2646.305	77021.773	0.1102
4		36.618	568388.500	69295464.000	99.1668
总计			579895.601	69877673.383	100.0000

Supplementary Figure 189. HPLC spectrum for (S_a,R)-4ck.

zj-7-114-if-100-1-1-214

实验时间：2016-12-29,13:10:36 报告时间：2016-12-29,17:38:32
谱图文件:F:\zhuguangjiong\zj\20161229\zj-7-114-if-100-1-1-214.org

实验内容简介：

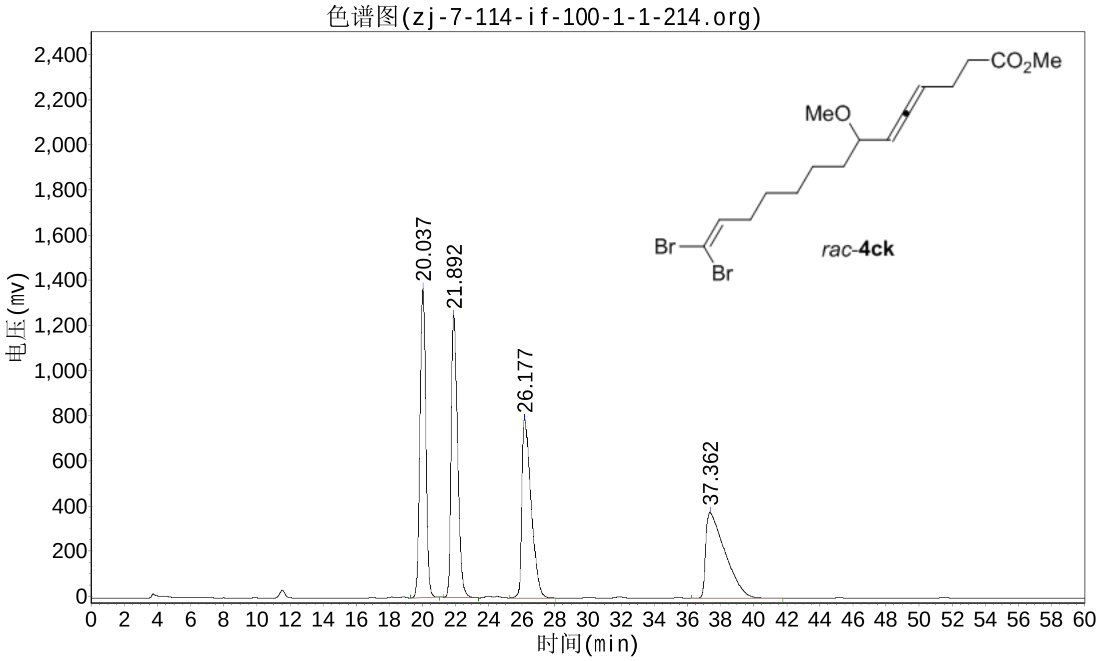

分析结果表

峰号	峰名	保留时间	峰高	峰面积	含量
1		20.037	1370312.625	33698112.000	25.9878
2		21.892	1249851.375	34018172.000	26.2346
3		26.177	792350.000	31096976.000	23.9818
4		37.362	380083.000	30855660.000	23.7957
总计			3792597.000	129668920.000	100.0000

Supplementary Figure 190. HPLC spectrum for *rac*-4ck.

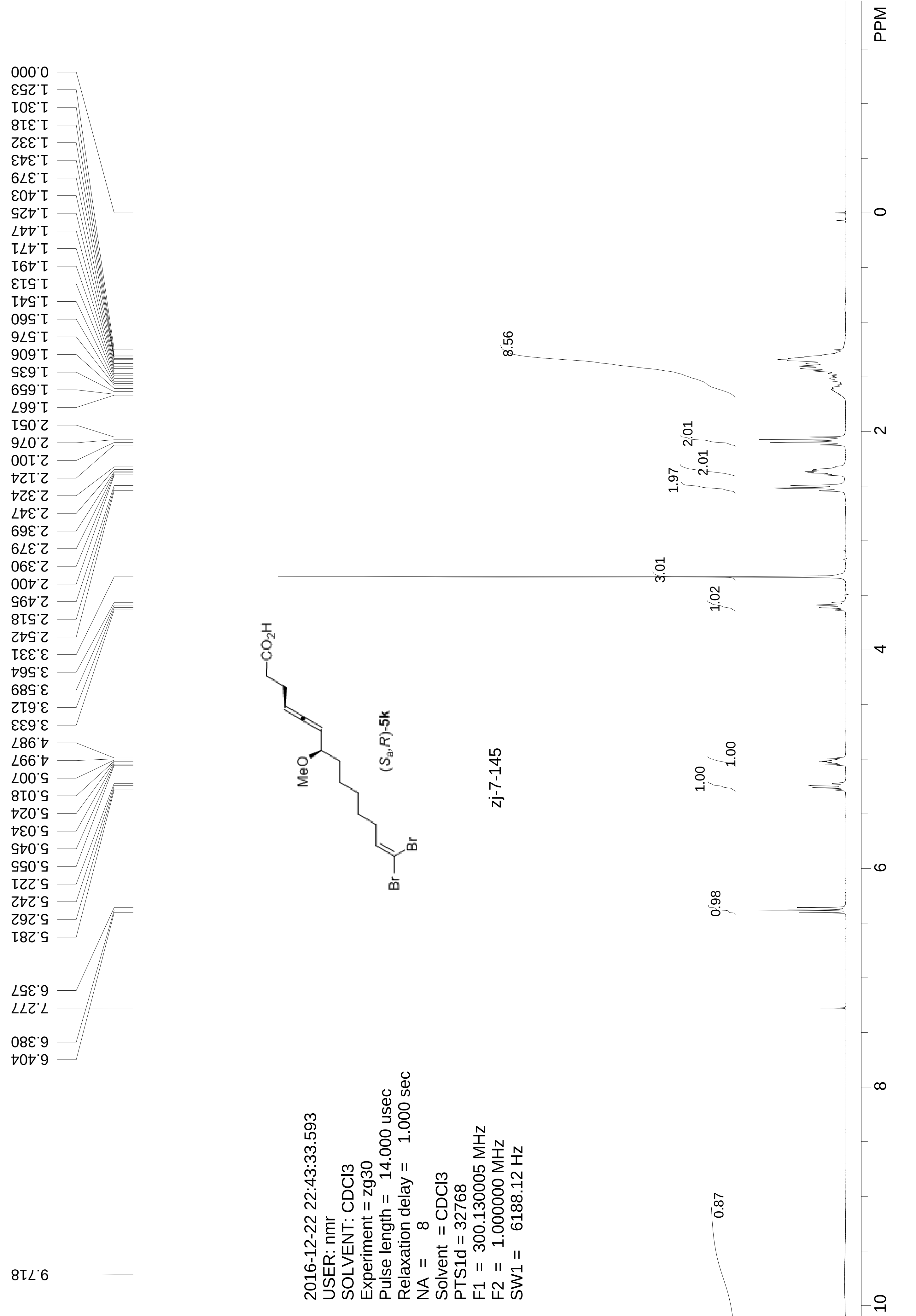

Supplementary Figure 191. ^{1}H NMR (300 MHz, $CDCl_3$) spectrum for (S_a,R)-5k.

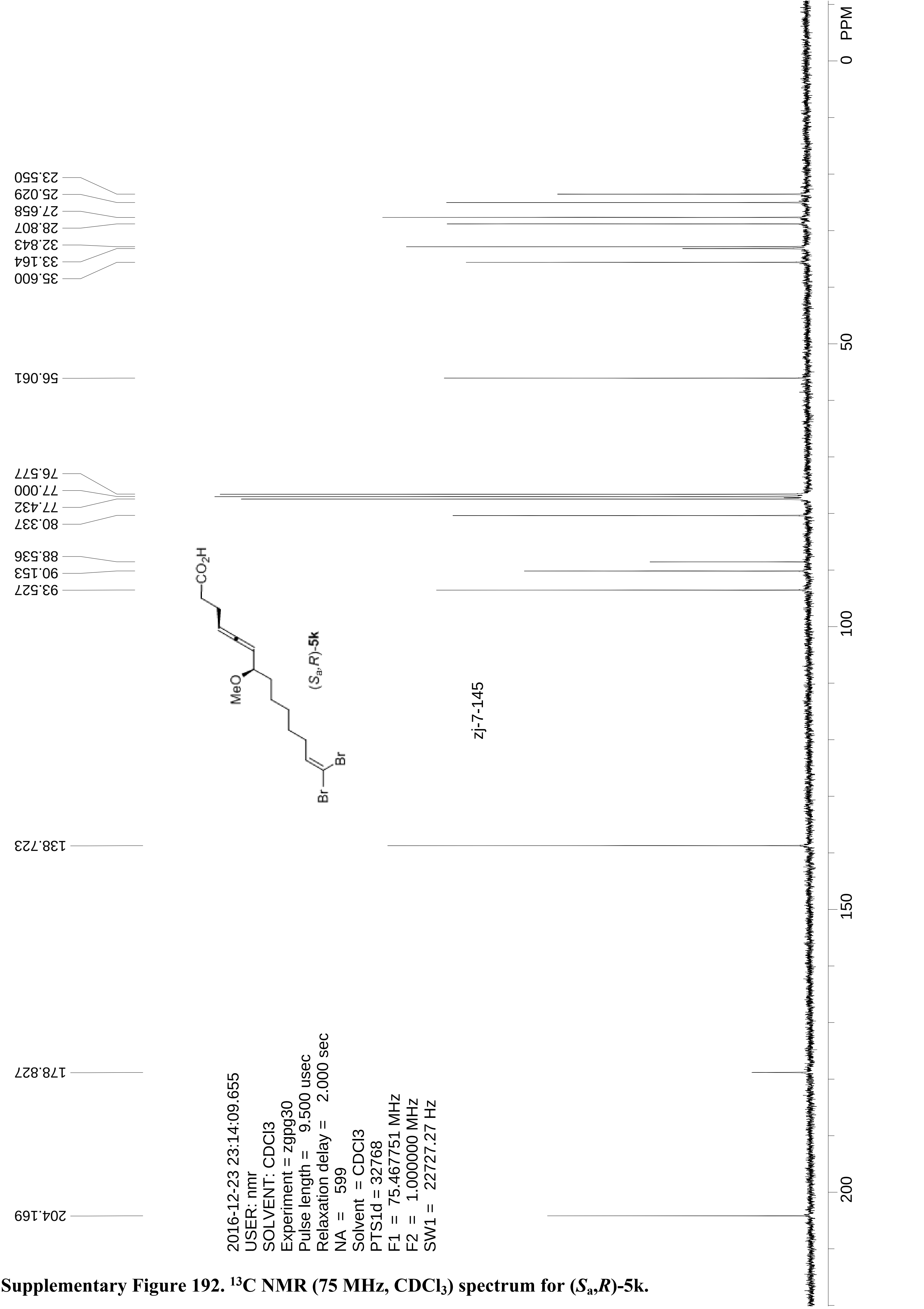

Supplementary Figure 192. ^{13}C NMR (75 MHz, $CDCl_3$) spectrum for (S_a,R)-5k.

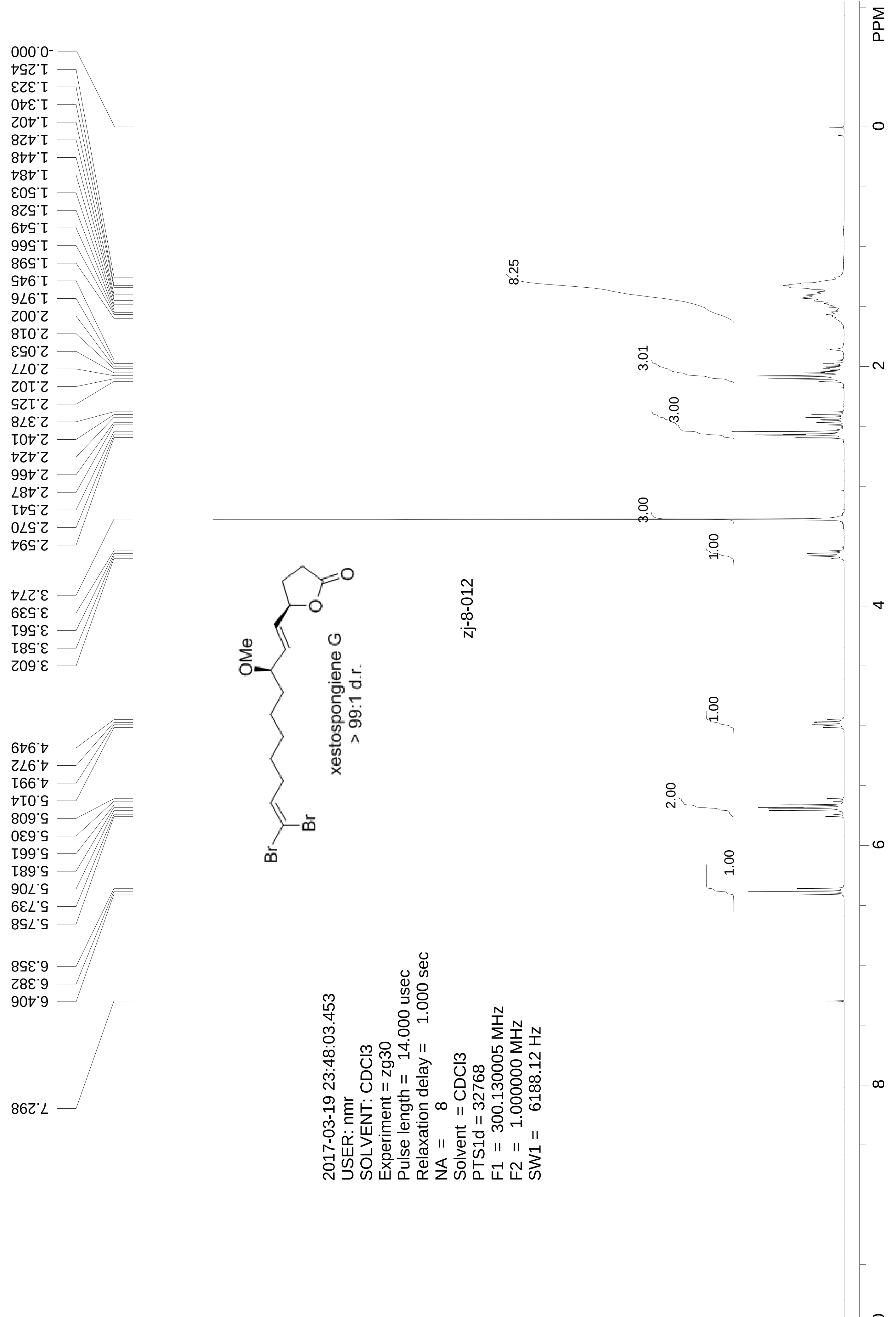

Supplementary Figure 193. ^{1}**H NMR (300 MHz, CDCl**$_3$**) spectrum for xestospongiene G.**

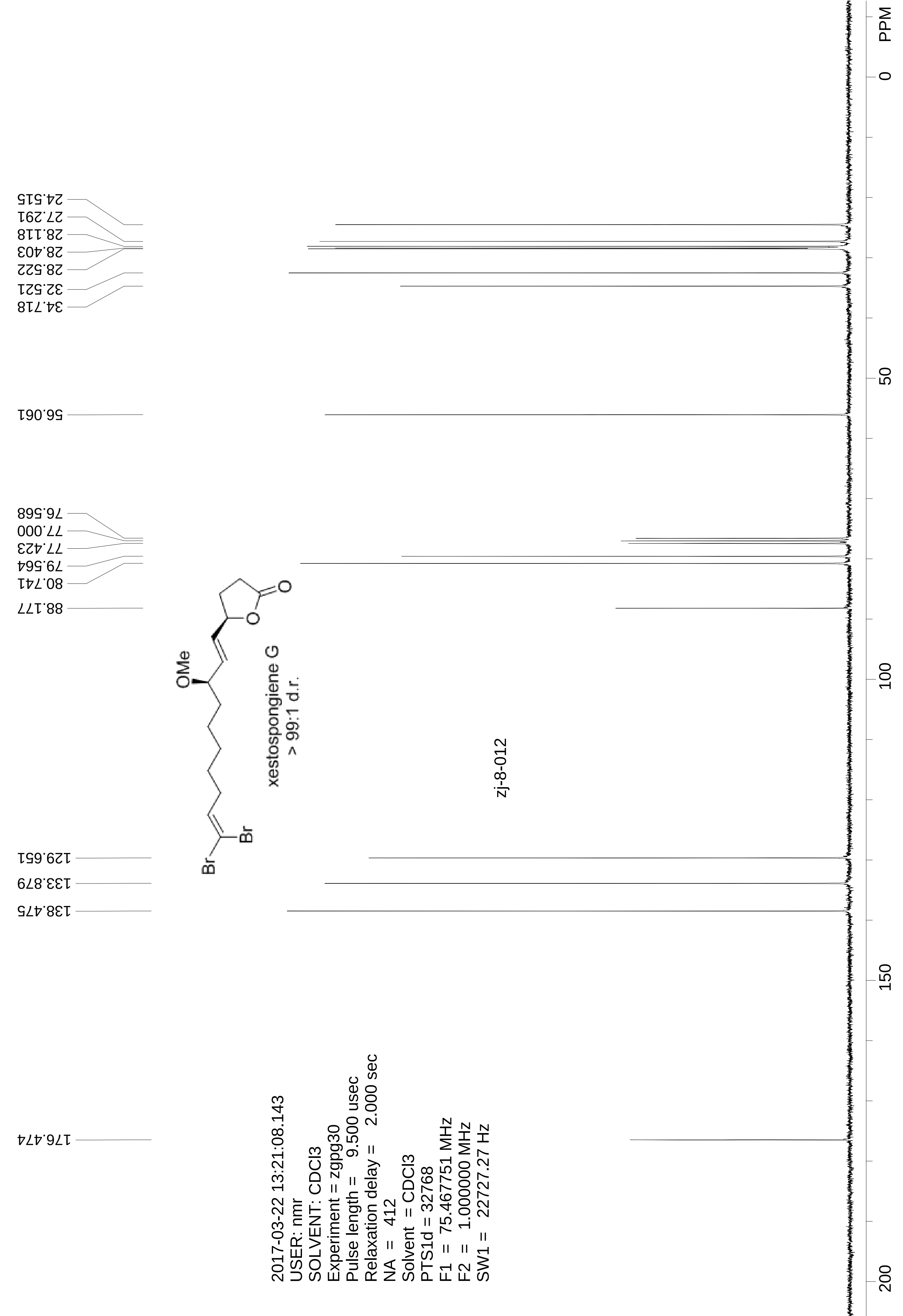

Supplementary Figure 194. ^{13}C NMR (75 MHz, $CDCl_3$) spectrum for xestospongiene G.

SAMPLE INFORMATION

Sample Name:	ZJ8012 ADH912200040	Acquired By:	System
Sample Type:		Date Acquired:	2017/3/22 18:02:58 CST
Vial:	1:A,5	Acq. Method Set:	chiral_isocratic
Injection:	1	Date Processed:	2017/3/23 14:19:41 CST
Injection Volume:	3.00 ul	Processing Methoc	1
Run Time:	30.0 Minutes	Channel Name:	PDA Ch1 214 nm@1.2 nm
Sample Set Name	20170322	Proc. Chnl. Descr:	PDA Ch1 214 nm@1.2 nm

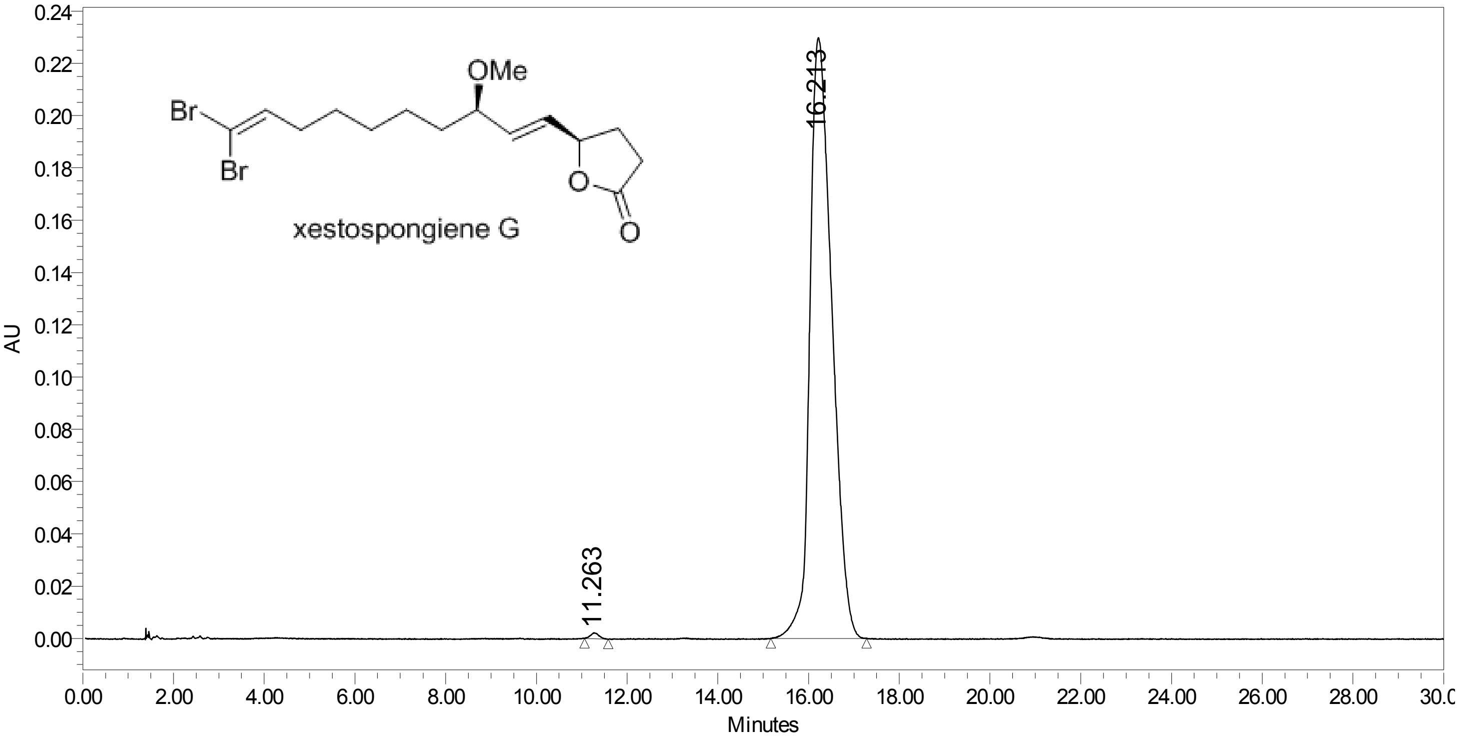

Peak Results

	RT	Area	Height	% Area
1	11.263	29947	2316	0.39
2	16.213	7743535	229889	99.61

Supplementary Figure 195. SFC spectrum for xestospongiene G.

SAMPLE INFORMATION

Sample Name:	ZJ7-158 adh912214200040	Acquired By:	System
Sample Type:		Date Acquired:	2017/3/22 14:45:20 CST
Vial:	1:A,1	Acq. Method Set:	chiral_isocratic
Injection:	1	Date Processed:	2017/3/23 14:07:00 CST
Injection Volume:	5.00 ul	Processing Methoc	1
Run Time:	30.0 Minutes	Channel Name:	PDA Ch1 214 nm@1.2 nm
Sample Set Name	20170213	Proc. Chnl. Descr:	PDA Ch1 214 nm@1.2 nm

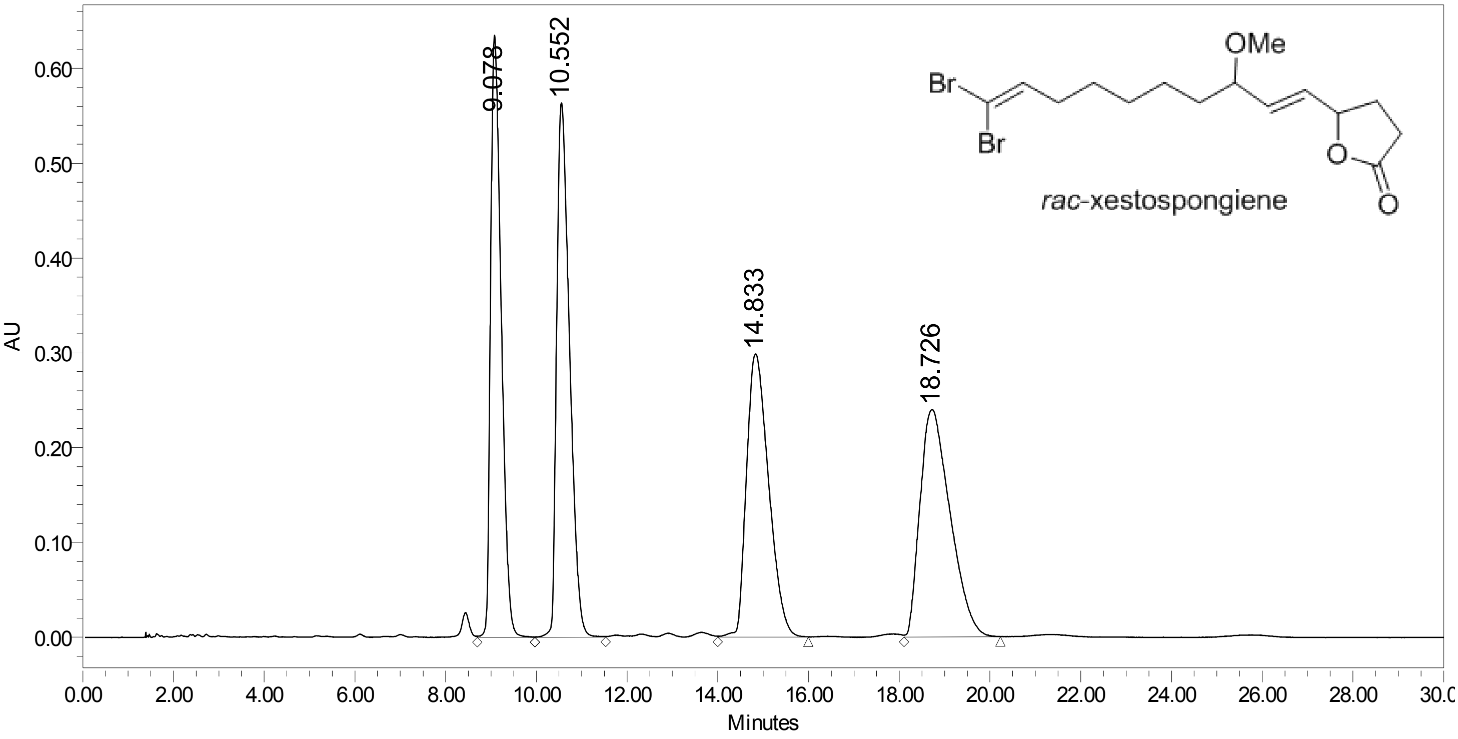

Peak Results

	RT	Area	Height	% Area
1	9.078	10369086	635306	24.26
2	10.552	1117745[illegible]	563713	26.15
3	14.833	10145220	298583	23.73
4	18.726	1105206[illegible]	239962	25.86

Supplementary Figure 196. SFC spectrum for *rac*-xestospongiene.

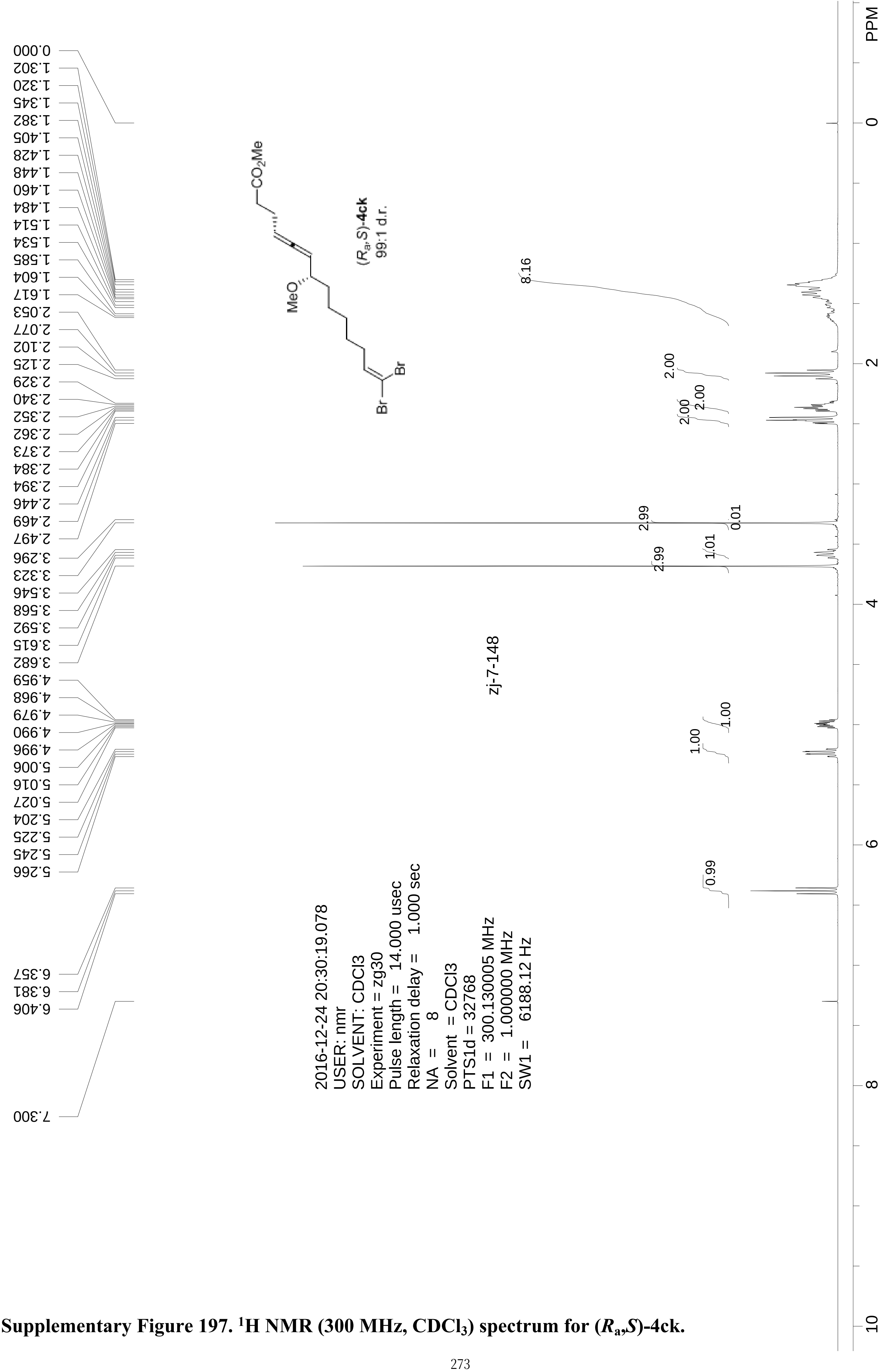

Supplementary Figure 197. ^{1}H NMR (300 MHz, $CDCl_3$) spectrum for (R_a,S)-4ck.

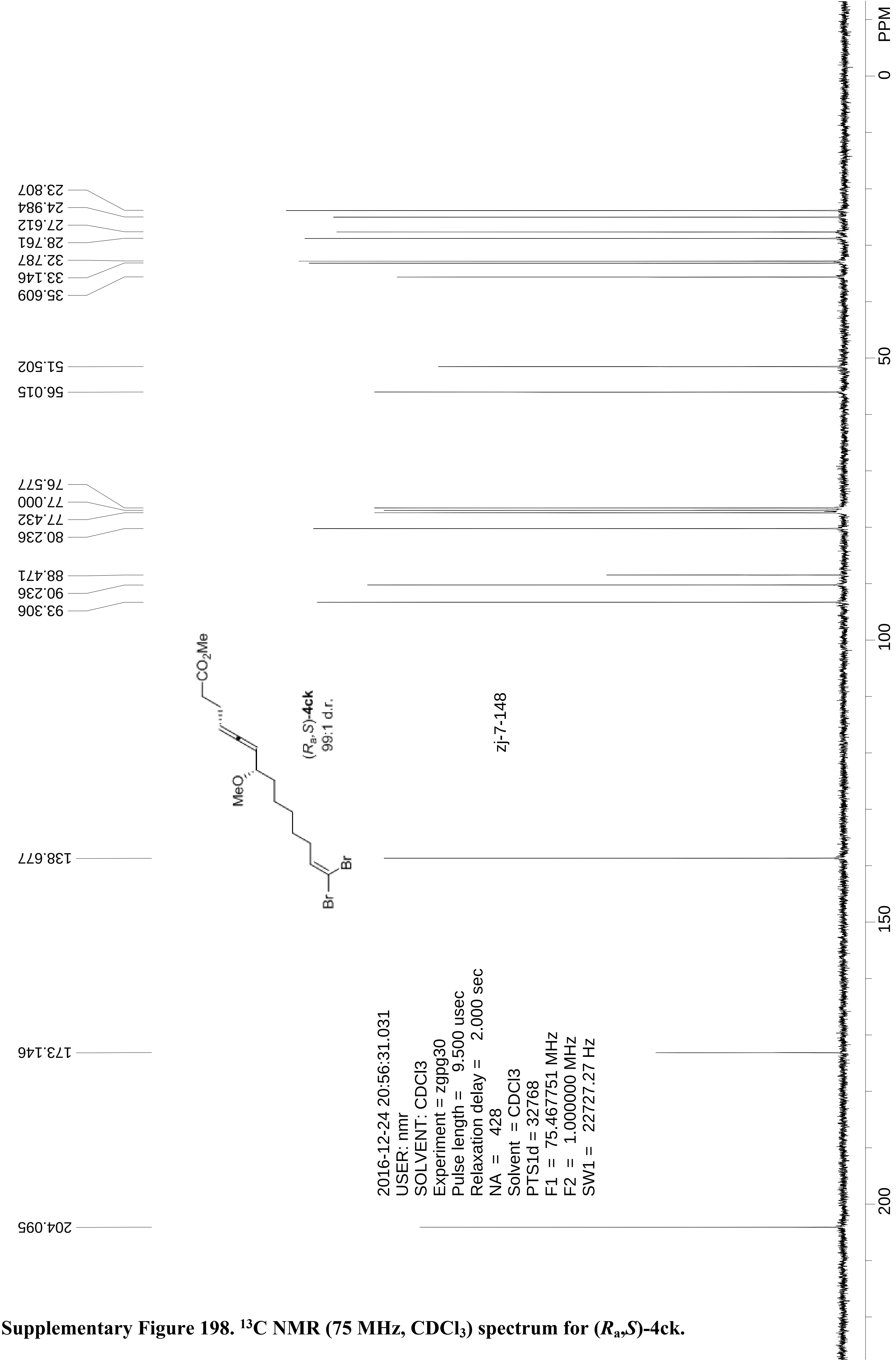

Supplementary Figure 198. ^{13}C NMR (75 MHz, $CDCl_3$) spectrum for (R_a,S)-4ck.

zj-7-148-if-100-1-1-214

实验时间：2016-12-29,14:26:02　　报告时间：2016-12-29,17:43:32
谱图文件:F:\zhuguangjiong\zj\20161229\zj-7-148-if-100-1-1-214.org

实验内容简介：

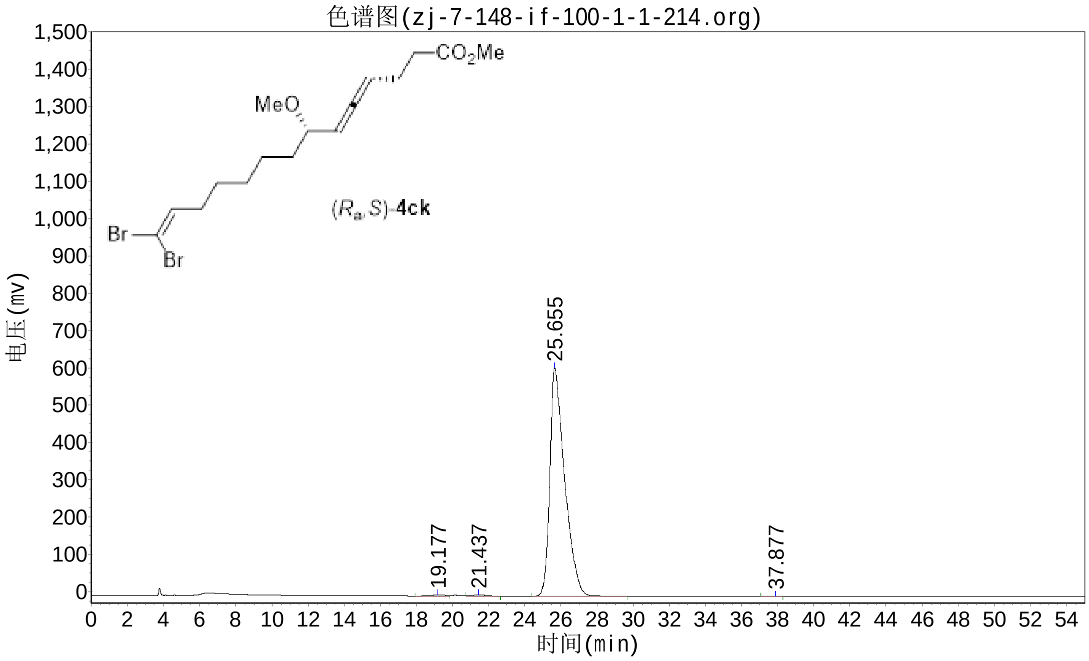

分析结果表

峰号	峰名	保留时间	峰高	峰面积	含量
1		19.177	2891.311	167002.906	0.4745
2		21.437	3269.852	175523.078	0.4987
3		25.655	611125.375	34820224.000	98.9392
4		37.877	730.933	30796.018	0.0875
总计			618017.471	35193546.002	100.0000

Supplementary Figure 199. HPLC spectrum for (*R*$_a$,*S*)-4ck.

zj-7-114-if-100-1-1-214

实验时间：2016-12-29,13:10:36　　　　　　　　　　　　　　　　报告时间：2016-12-29,17:38:32
谱图文件:F:\zhuguangjiong\zj\20161229\zj-7-114-if-100-1-1-214.org

实验内容简介：

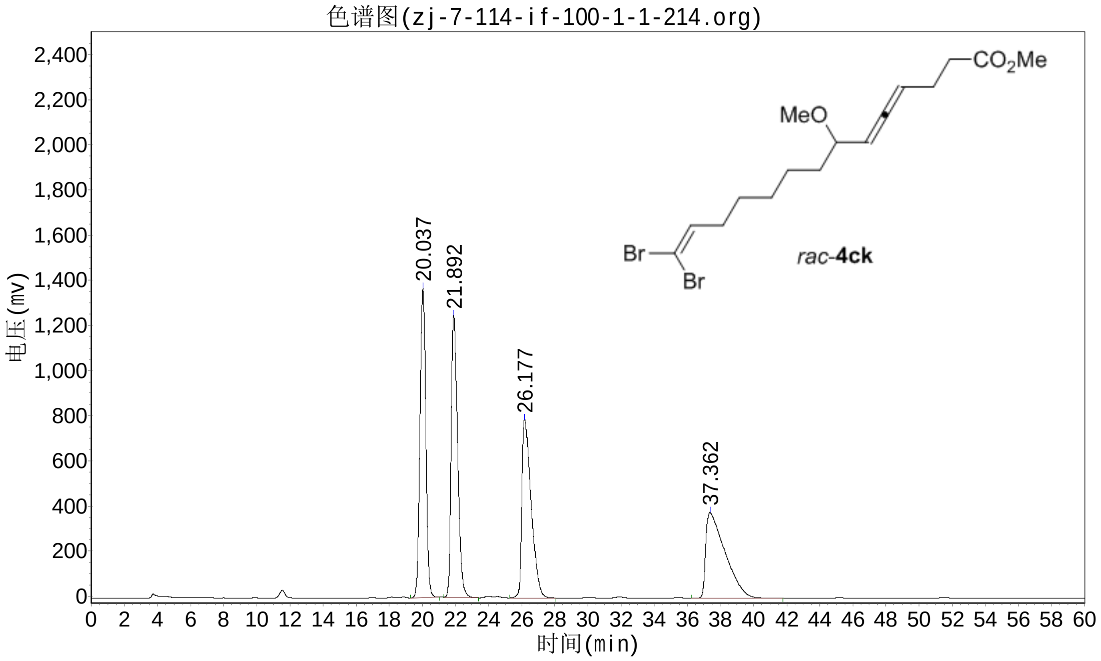

分析结果表

峰号	峰名	保留时间	峰高	峰面积	含量
1		20.037	1370312.625	33698112.000	25.9878
2		21.892	1249851.375	34018172.000	26.2346
3		26.177	792350.000	31096976.000	23.9818
4		37.362	380083.000	30855660.000	23.7957
总计			3792597.000	129668920.000	100.0000

Supplementary Figure 200. HPLC spectrum for *rac*-4ck.

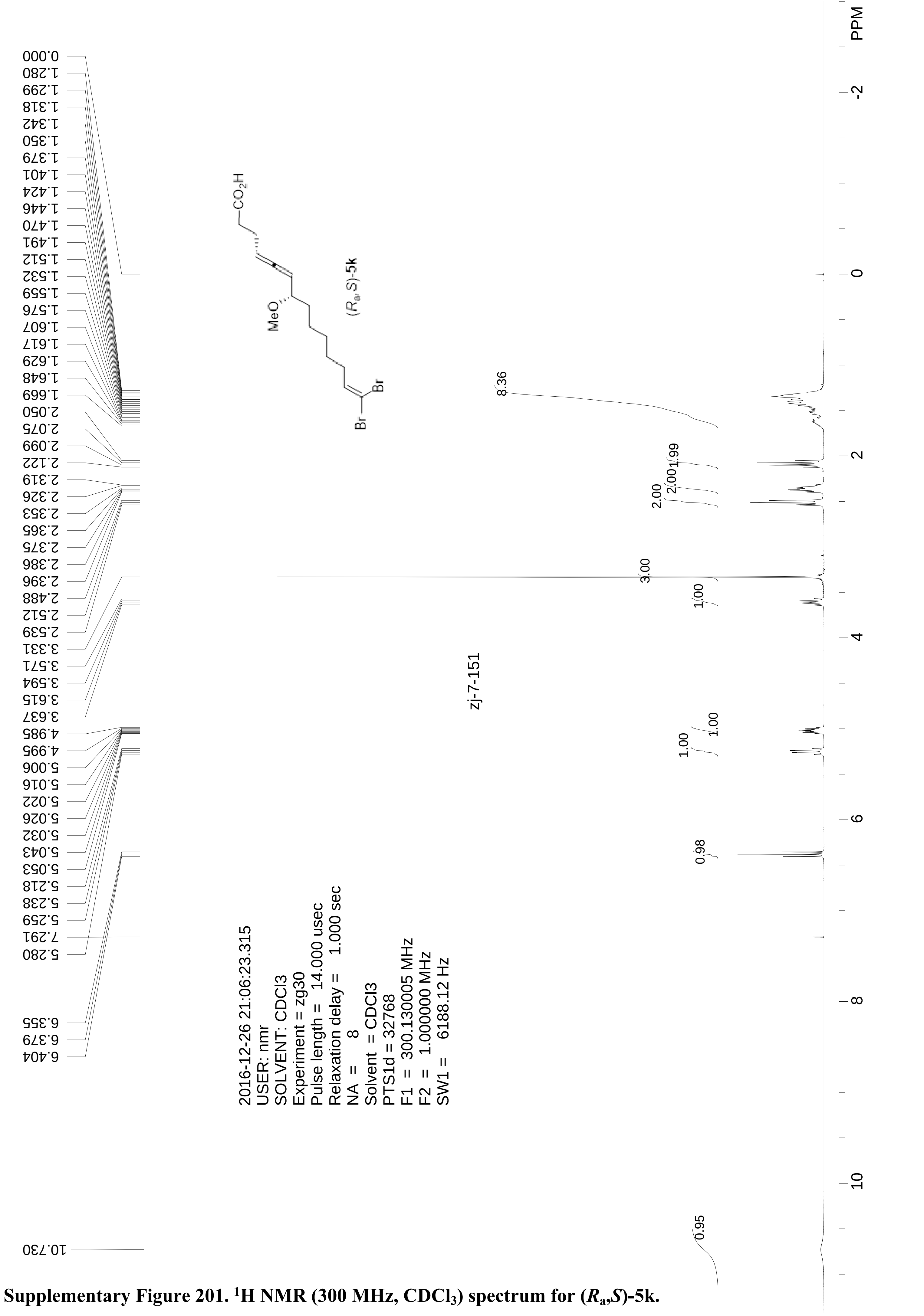

Supplementary Figure 201. ^1H NMR (300 MHz, $CDCl_3$) spectrum for (R_a,S)-5k.

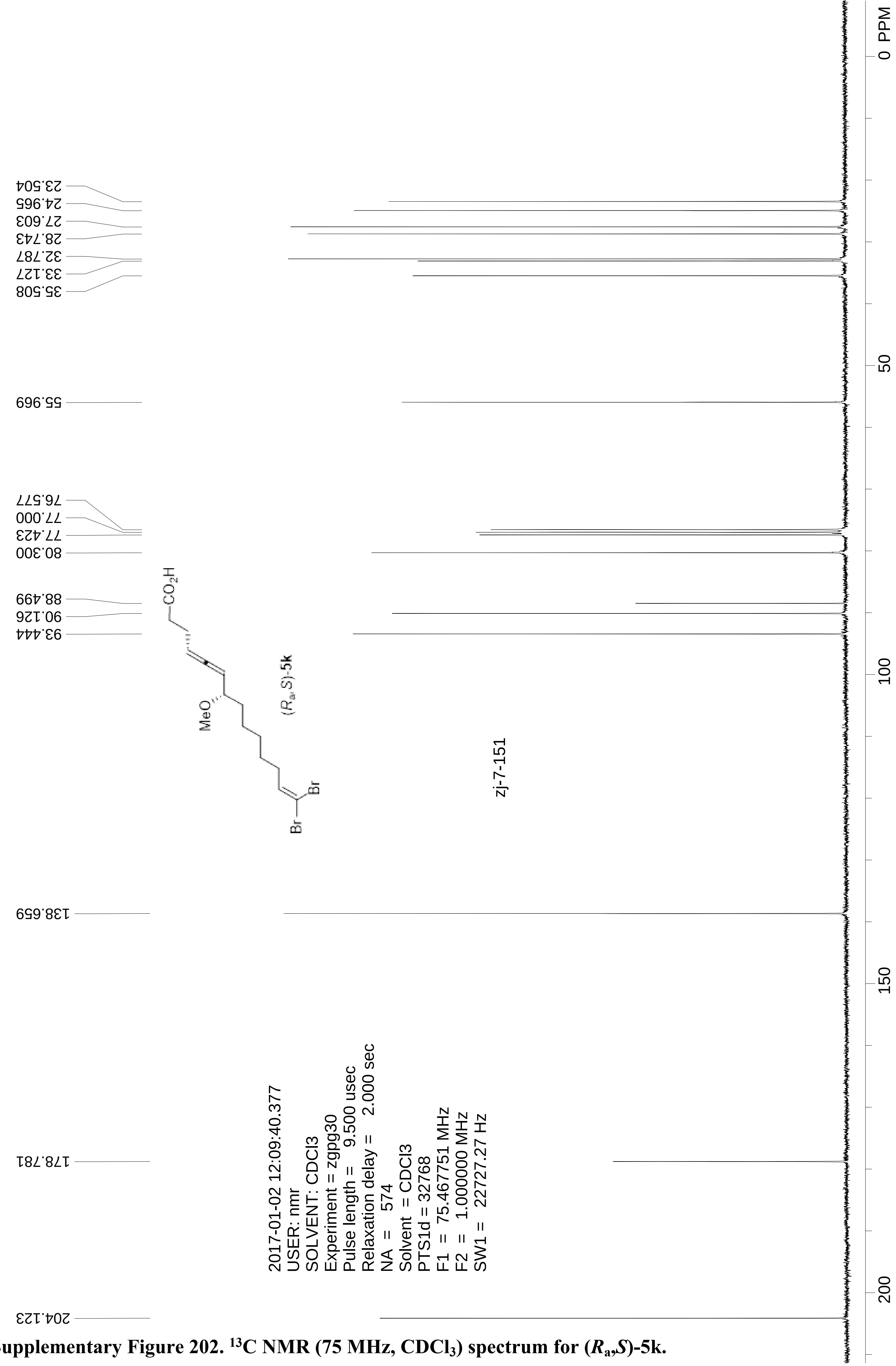

Supplementary Figure 202. ^{13}C NMR (75 MHz, $CDCl_3$) spectrum for (R_a,S)-5k.

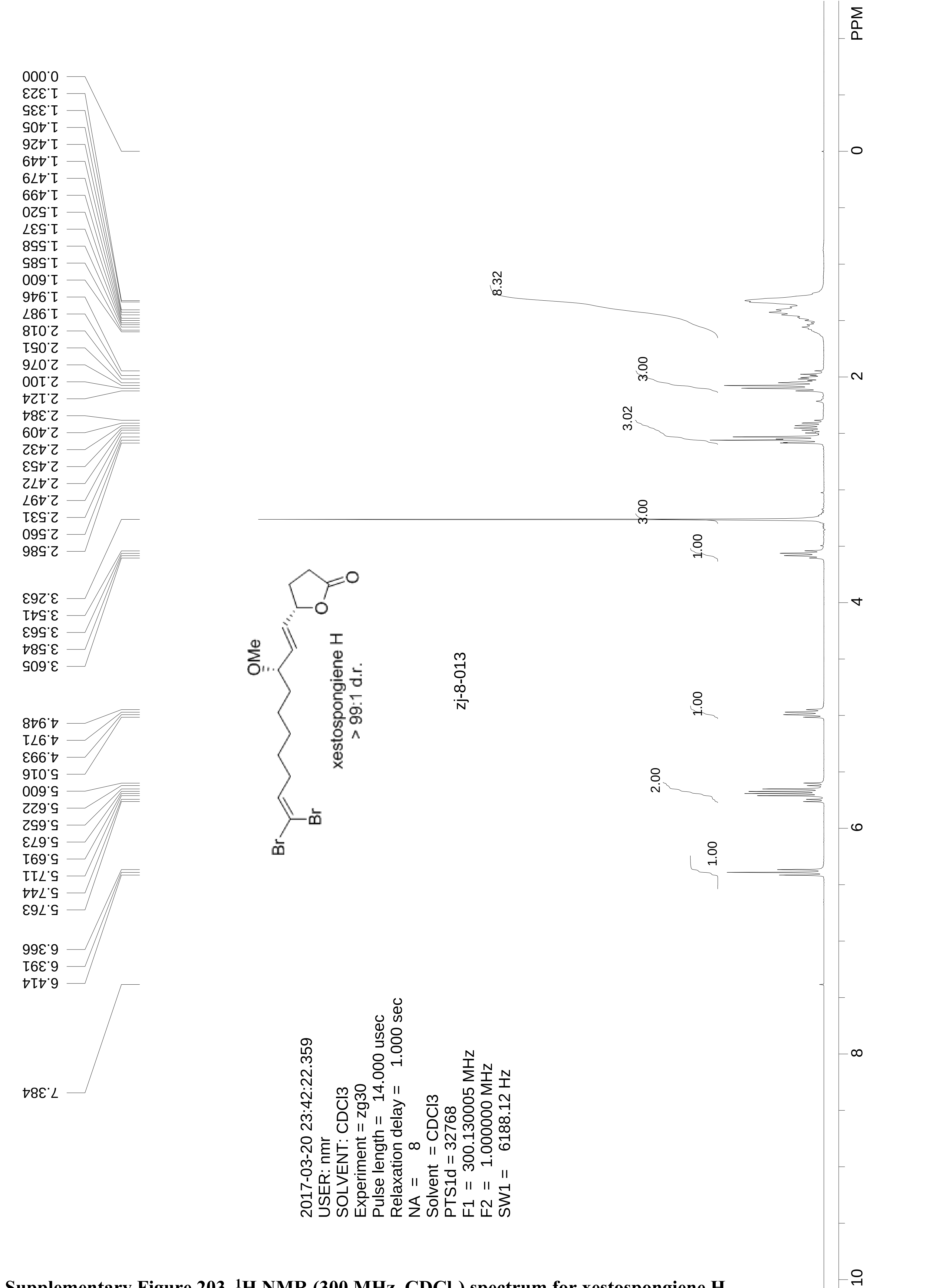

Supplementary Figure 203. ^{1}H NMR (300 MHz, $CDCl_3$) spectrum for xestospongiene H.

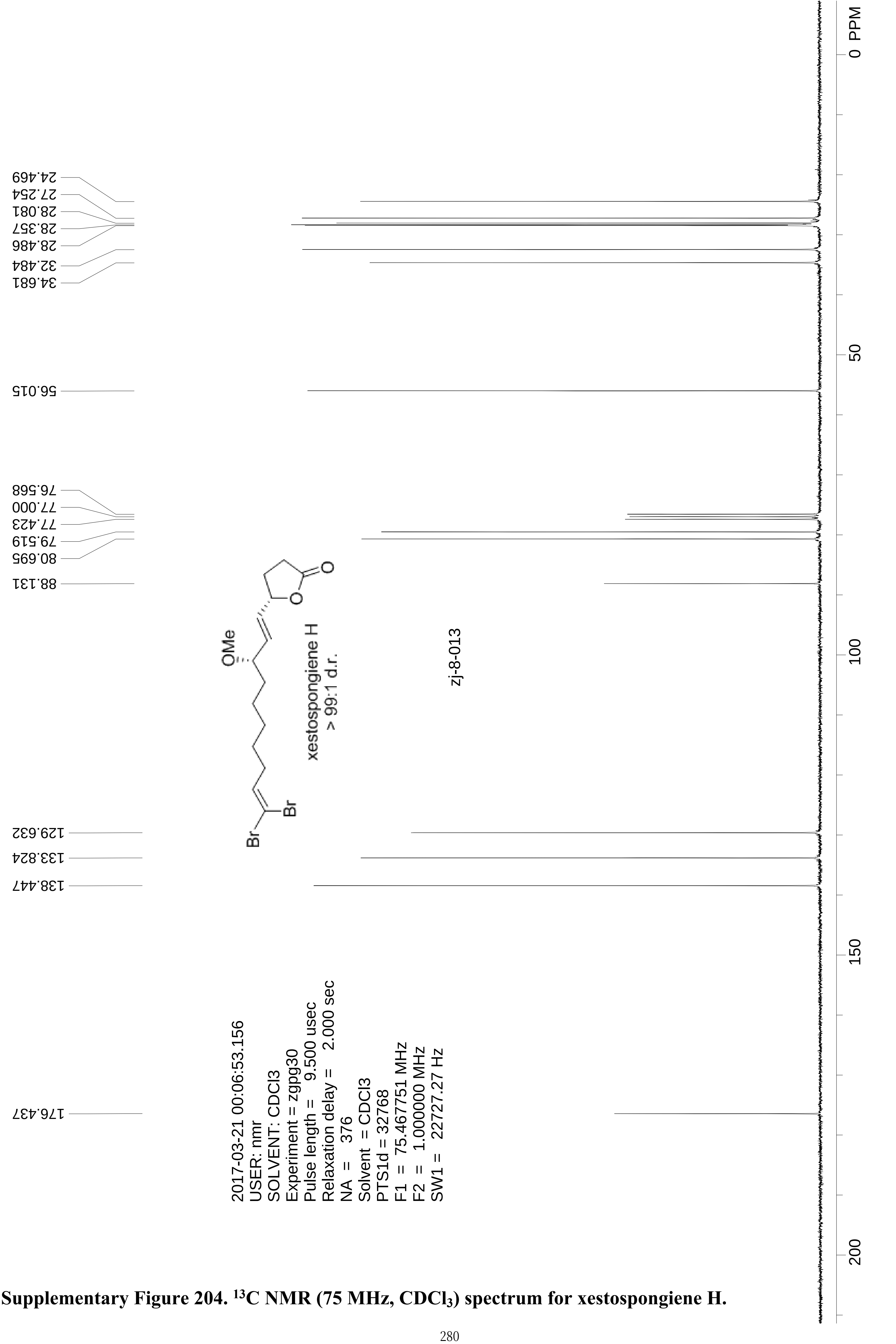

Supplementary Figure 204. ^{13}C NMR (75 MHz, $CDCl_3$) spectrum for xestospongiene H.

Empower™ 3 SOFTWARE

SAMPLE INFORMATION

Sample Name:	ZJ8013 ADH912200040	Acquired By:	System
Sample Type:		Date Acquired:	2017/3/22 18:33:53 CST
Vial:	1:A,6	Acq. Method Set:	chiral_isocratic
Injection:	1	Date Processed:	2017/3/23 14:20:07 CST
Injection Volume:	3.00 ul	Processing Methoc	1
Run Time:	30.0 Minutes	Channel Name:	PDA Ch1 214 nm@1.2 nm
Sample Set Name	20170322	Proc. Chnl. Descr:	PDA Ch1 214 nm@1.2 nm

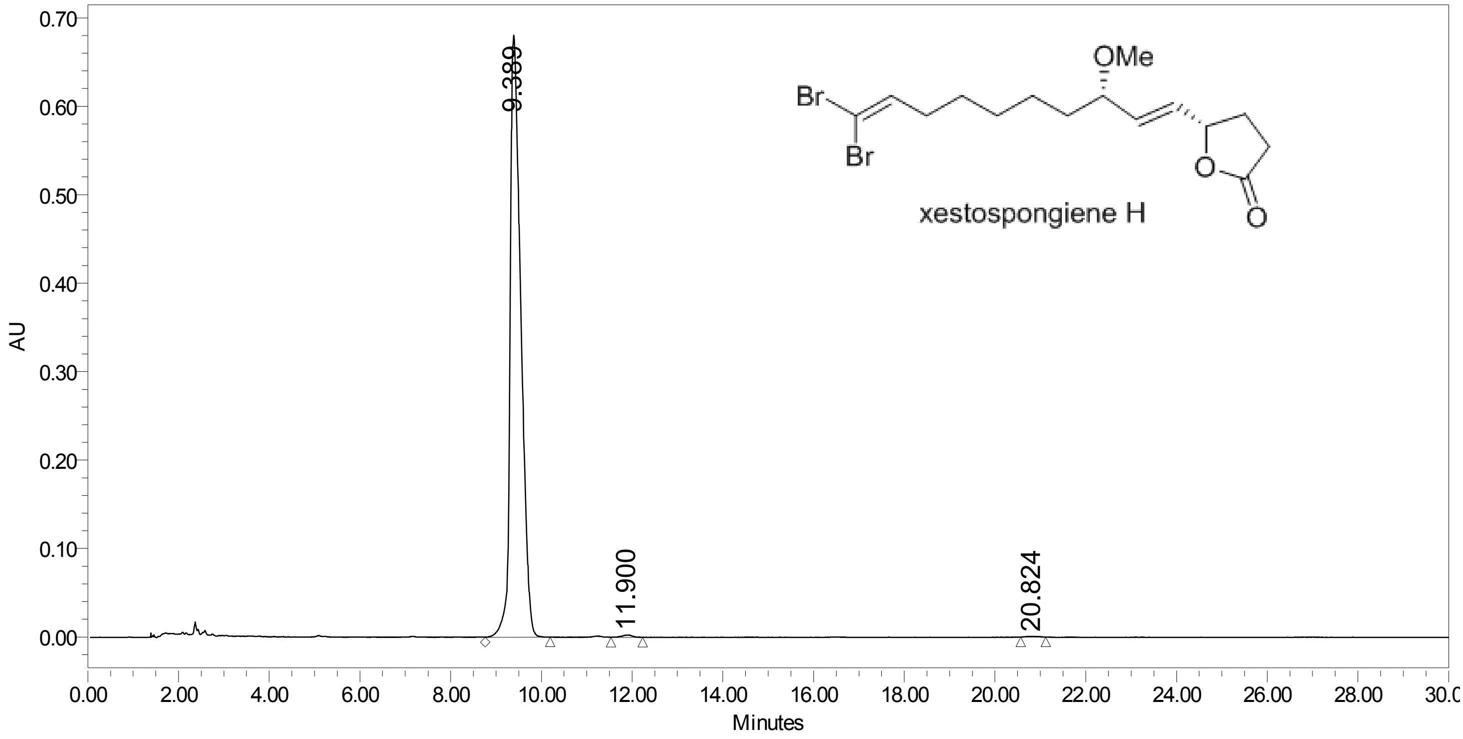

Peak Results

	RT	Area	Height	% Area
1	9.389	11206012	680745	99.48
2	11.900	42814	2644	0.38
3	20.824	15359	822	0.14

Supplementary Figure 205. SFC spectrum for xestospongiene H.

Empower™ 3 SOFTWARE

SAMPLE INFORMATION

Sample Name:	ZJ7-158 adh912214200040	Acquired By:	System
Sample Type:		Date Acquired:	2017/3/22 14:45:20 CST
Vial:	1:A,1	Acq. Method Set:	chiral_isocratic
Injection:	1	Date Processed:	2017/3/23 14:07:00 CST
Injection Volume:	5.00 ul	Processing Methoc	1
Run Time:	30.0 Minutes	Channel Name:	PDA Ch1 214 nm@1.2 nm
Sample Set Name	20170213	Proc. Chnl. Descr:	PDA Ch1 214 nm@1.2 nm

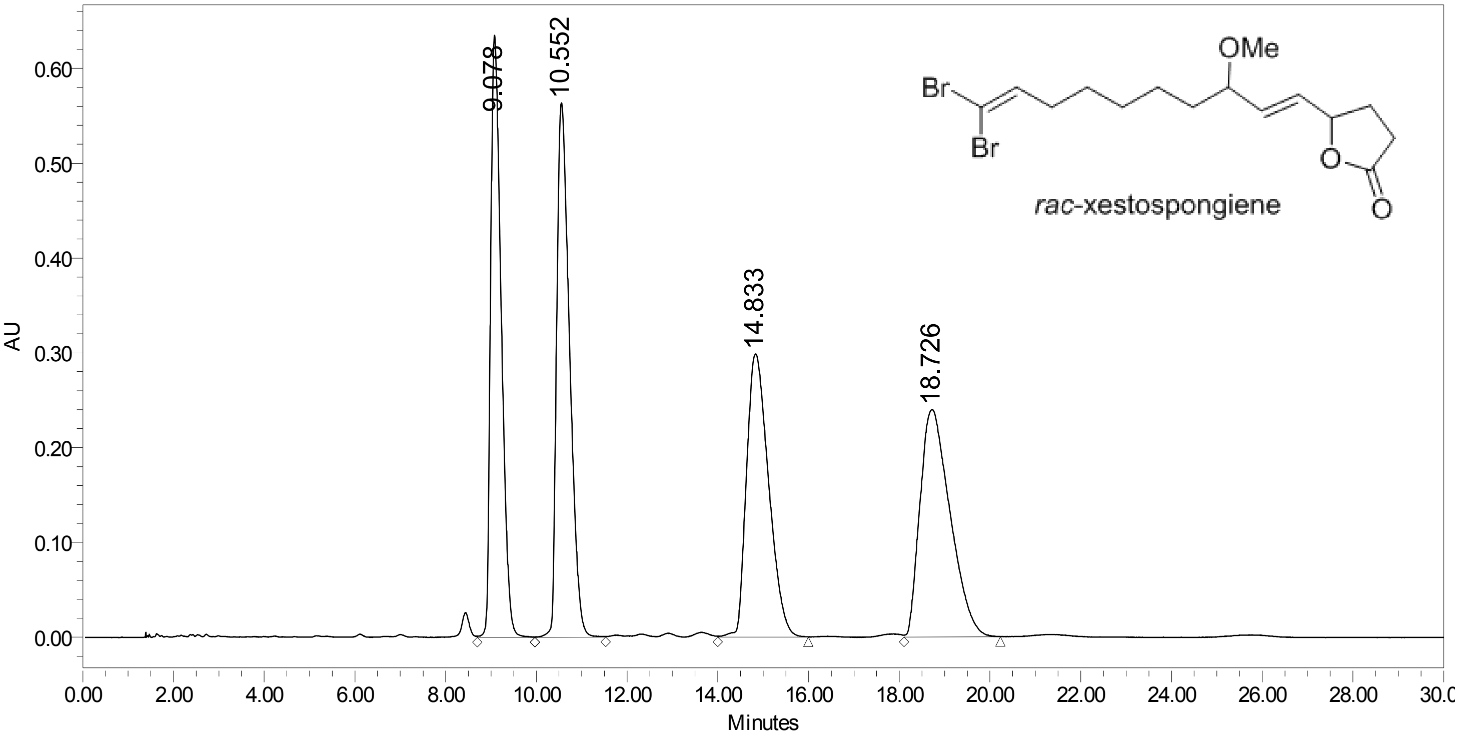

Peak Results

	RT	Area	Height	% Area
1	9.078	10369086	635306	24.26
2	10.552	1117745[illegible]	563713	26.15
3	14.833	10145220	298583	23.73
4	18.726	1105206[illegible]	239962	25.86

Supplementary Figure 206. SFC spectrum for *rac*-xestospongiene.

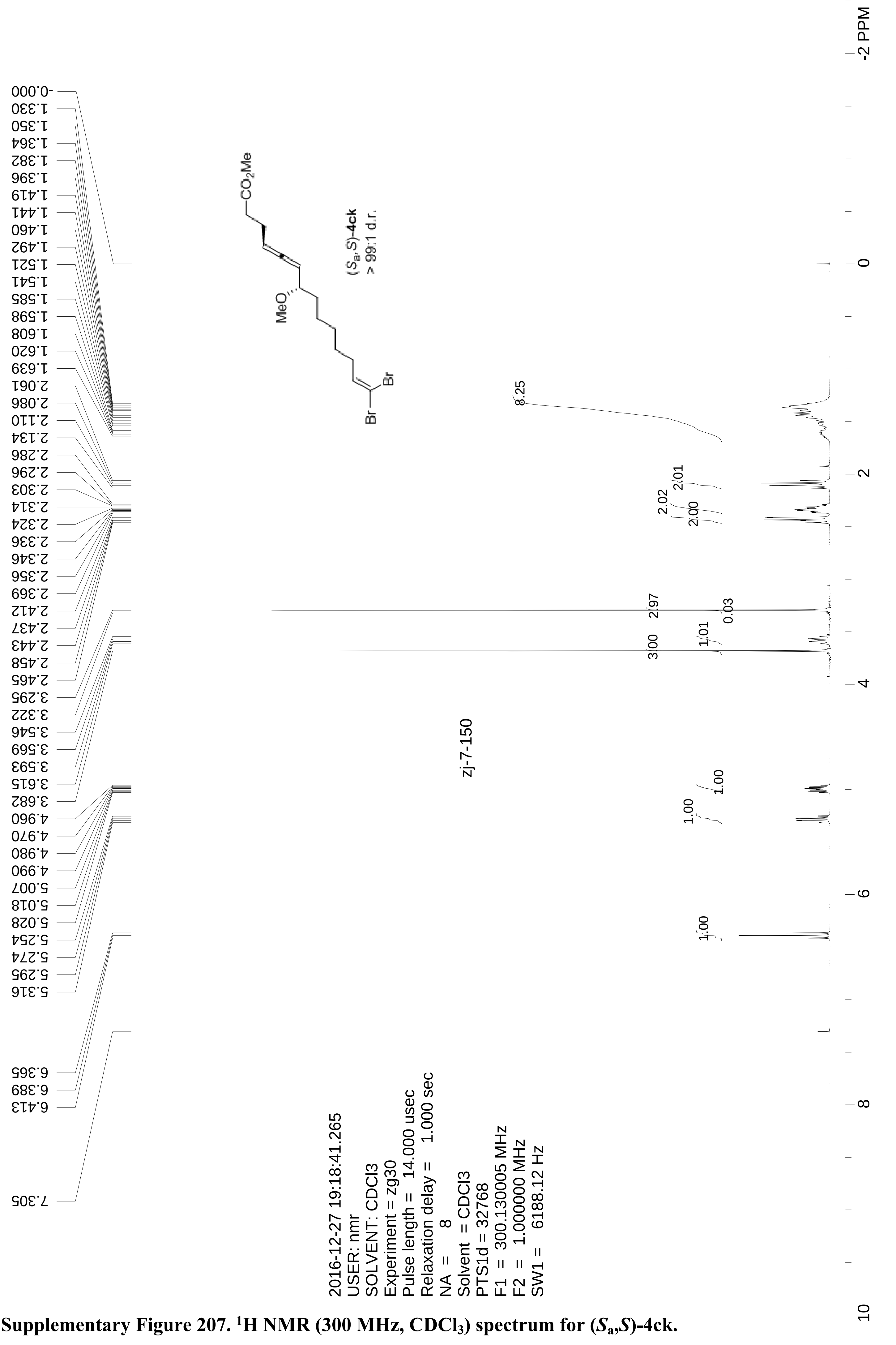

Supplementary Figure 207. ^{1}H **NMR (300 MHz,** $CDCl_3$**) spectrum for (*S*$_a$,*S*)-4ck.**

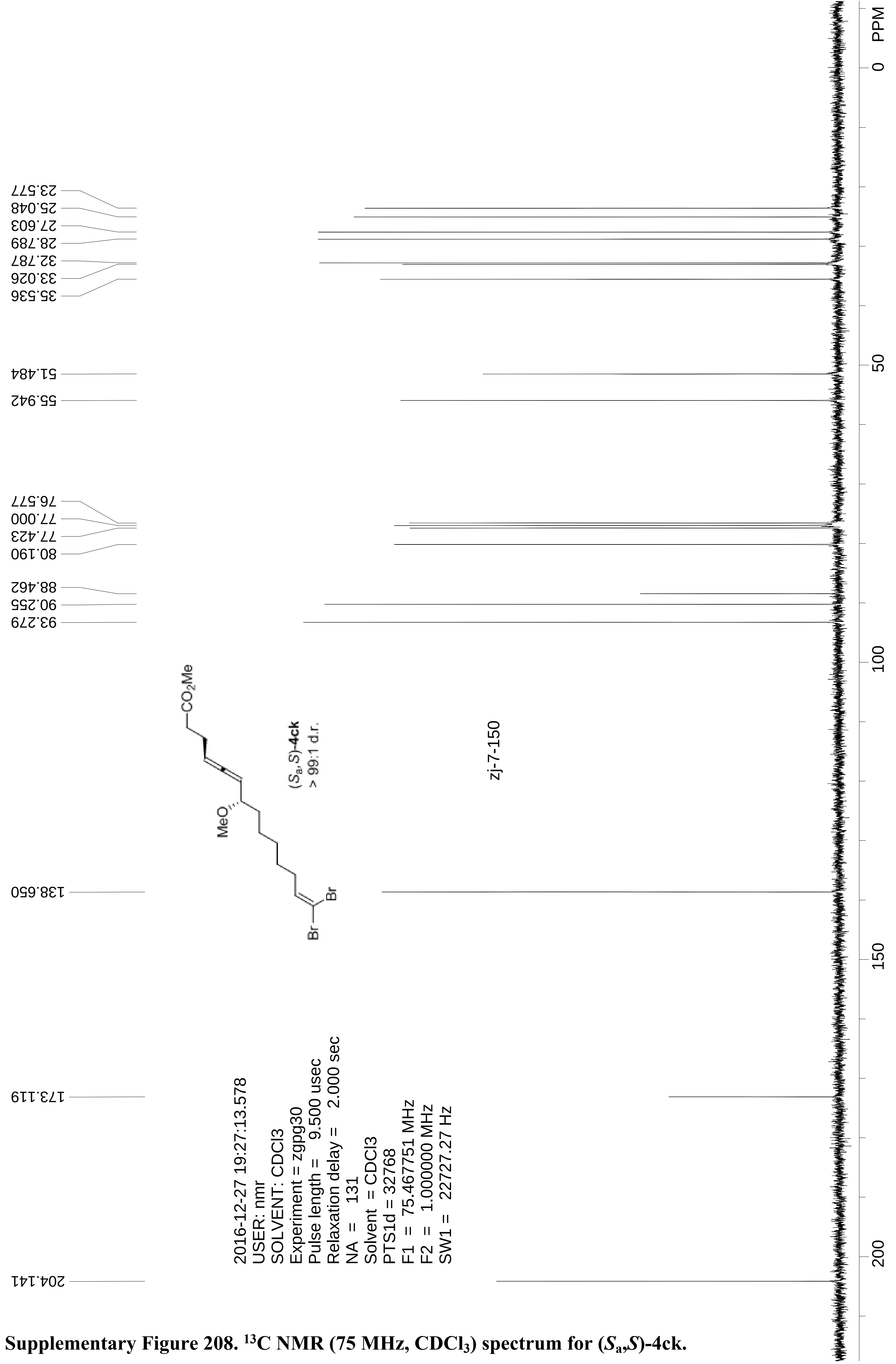

Supplementary Figure 208. ^{13}C NMR (75 MHz, $CDCl_3$) spectrum for (S_a,S)-4ck.

zj-7-150-if-3-100-1-1-214

实验时间：2017-01-13,18:09:09　　报告时间：2017-01-13,19:51:03
谱图文件:F:\zhuguangjiong\zj\20170113\zj-7-150-if-3-100-1-1-214.org

实验内容简介：

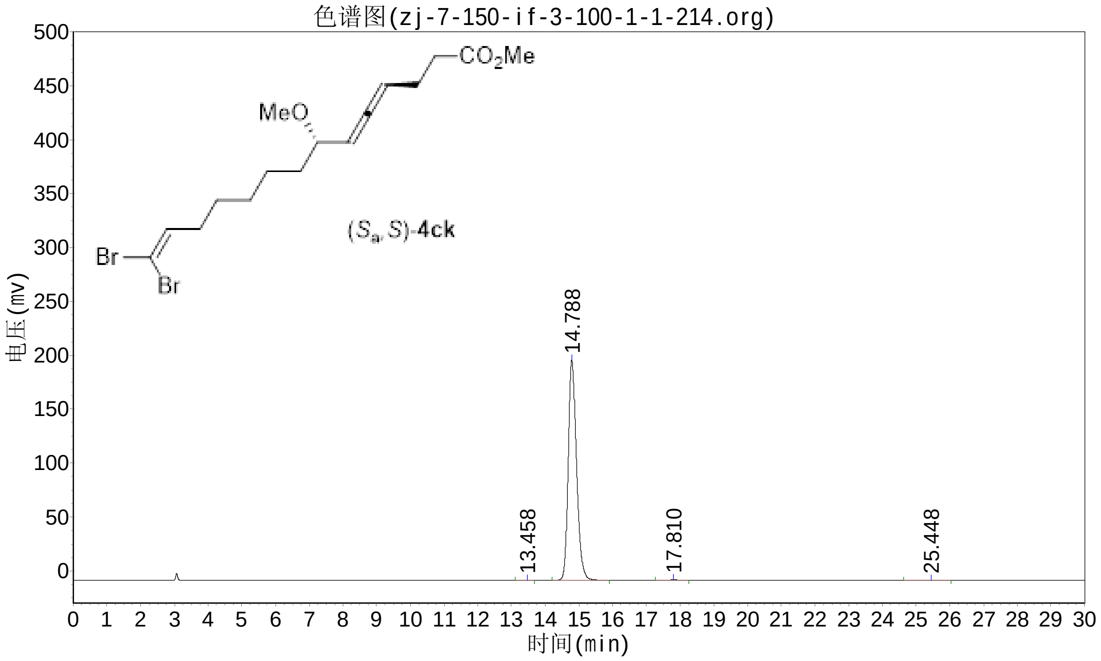

分析结果表

峰号	峰名	保留时间	峰高	峰面积	含量
1		13.458	103.315	1663.211	0.0472
2		14.788	204454.266	3509839.000	99.5168
3		17.810	476.592	9957.236	0.2823
4		25.448	177.939	5422.999	0.1538
总计			205212.112	3526882.446	100.0000

Supplementary Figure 209. HPLC spectrum for (S_a,S)-4ck.

zj-7-114-if-3-100-1-1-214

实验时间：2017-01-13,19:04:56　　　　　　　　　　　　　报告时间：2017-01-13,19:48:04
谱图文件:F:\zhuguangjiong\zj\20170113\zj-7-114-if-3-100-1-1-214.org

实验内容简介：

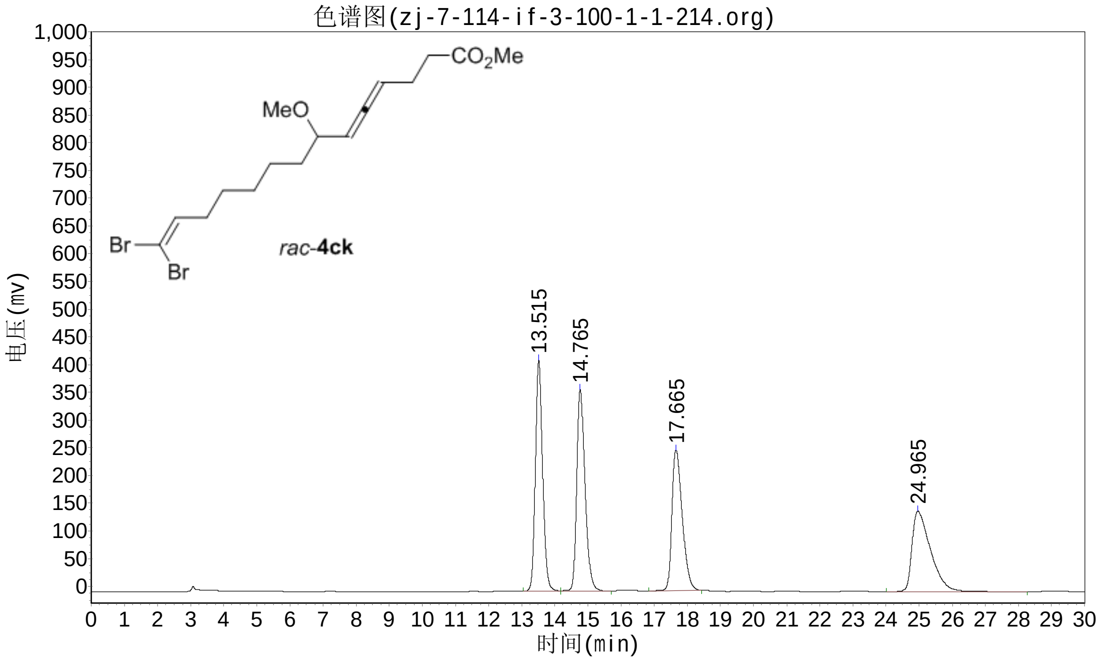

分析结果表

峰号	峰名	保留时间	峰高	峰面积	含量
1		13.515	415700.125	6377944.000	26.6744
2		14.765	363298.375	6413361.000	26.8225
3		17.665	253848.797	5528617.000	23.1223
4		24.965	144203.688	5590452.500	23.3809
总计			1177050.984	23910374.500	100.0000

Supplementary Figure 210. HPLC spectrum for *rac*-4ck.

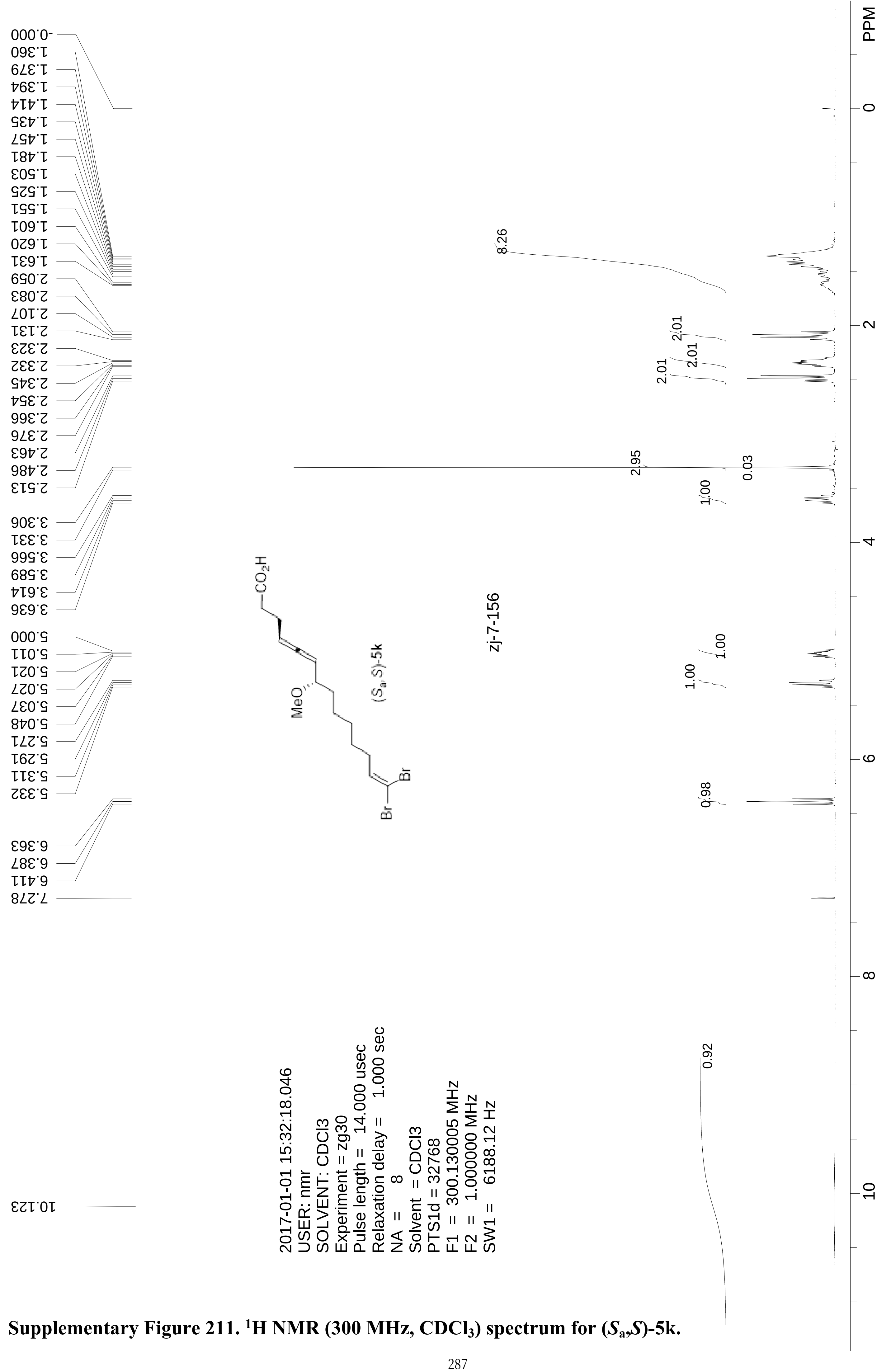

Supplementary Figure 211. ^{1}**H NMR (300 MHz, CDCl$_3$) spectrum for (*S*$_a$,*S*)-5k.**

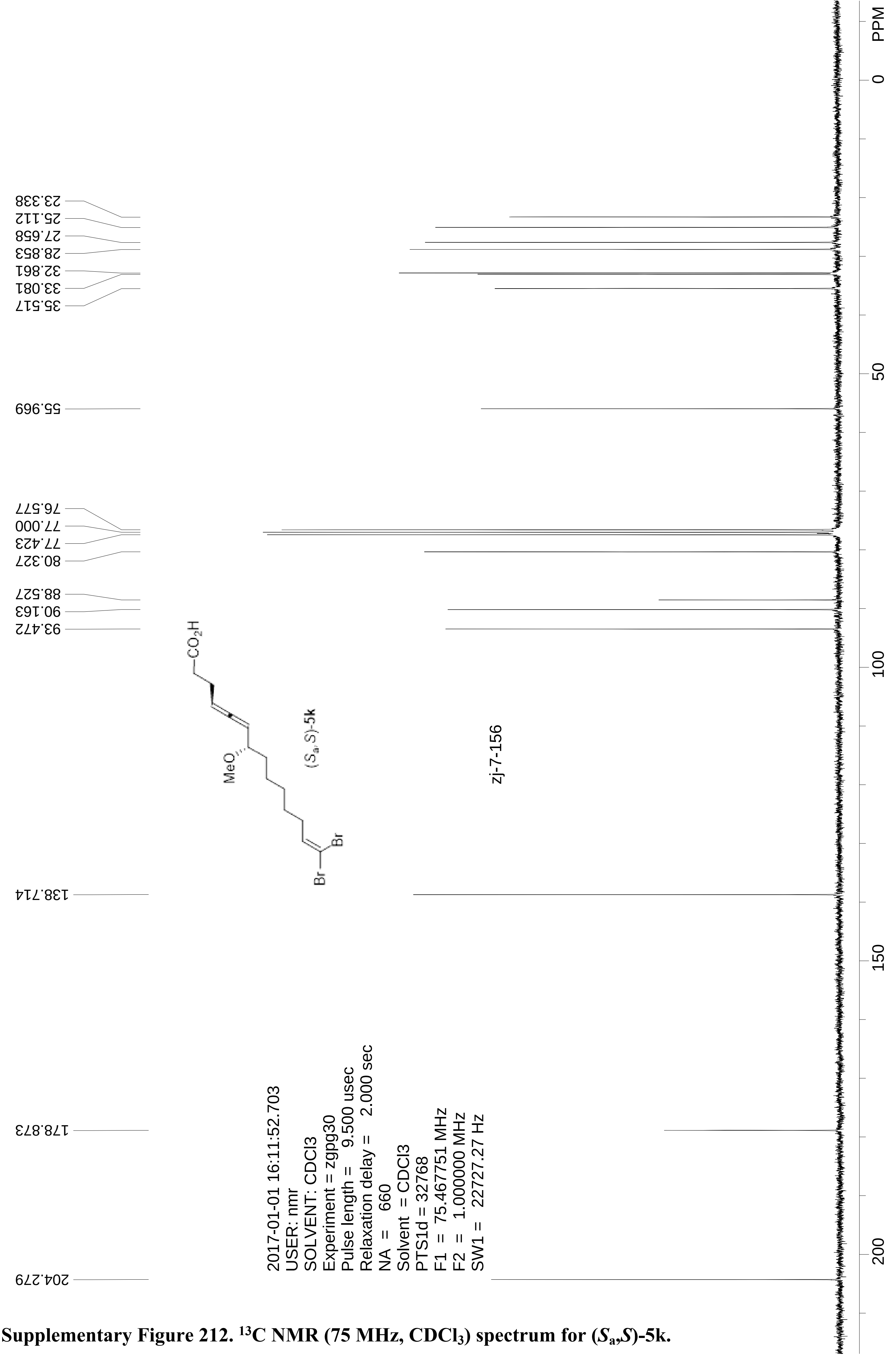

Supplementary Figure 212. **^{13}C NMR (75 MHz, CDCl$_3$) spectrum for (*S*$_a$,*S*)-5k.**

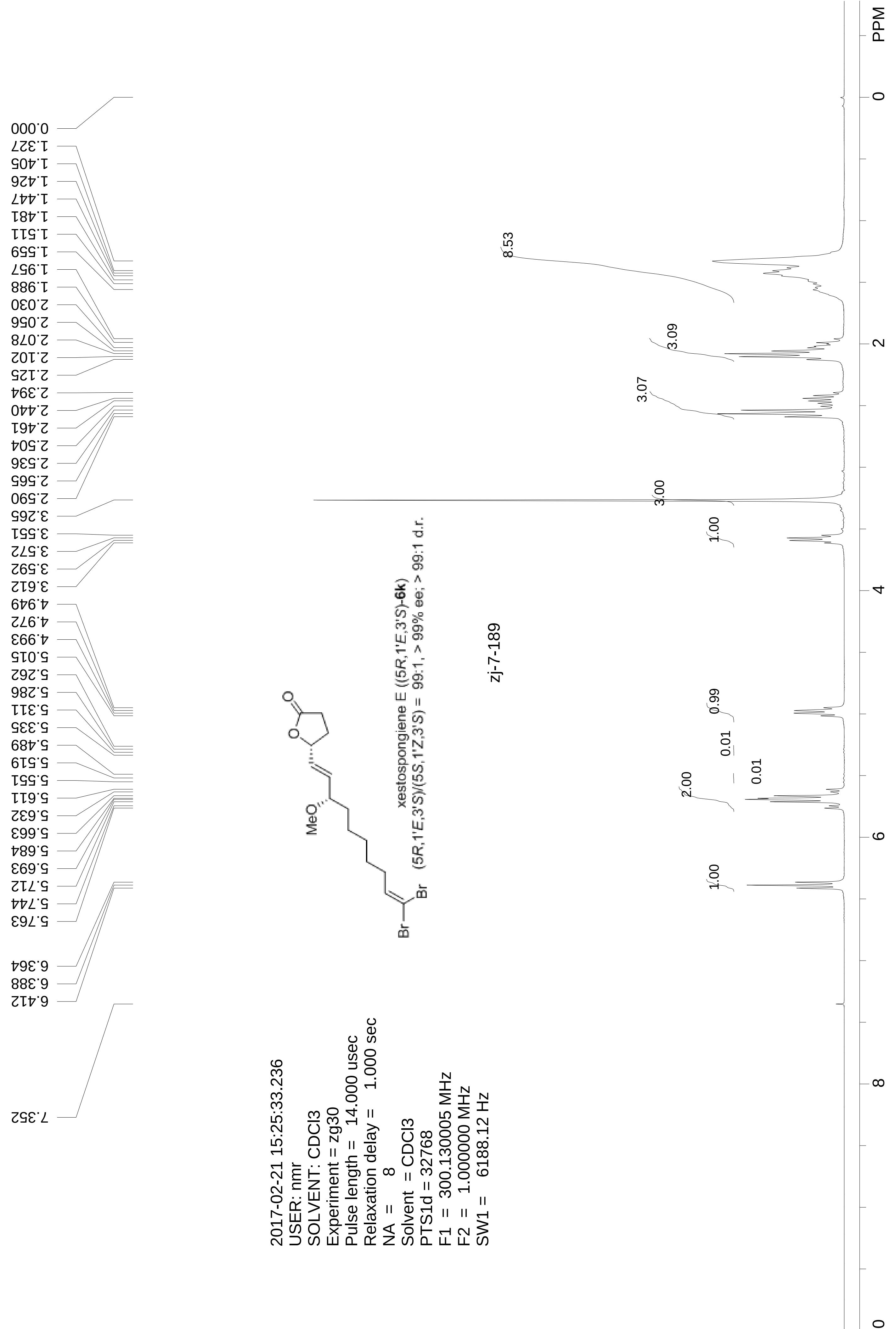

Supplementary Figure 213. ^{1}H NMR (300 MHz, $CDCl_3$) spectrum for xestospongiene E ((5*R*,1'*E*,3'*S*)-6k).

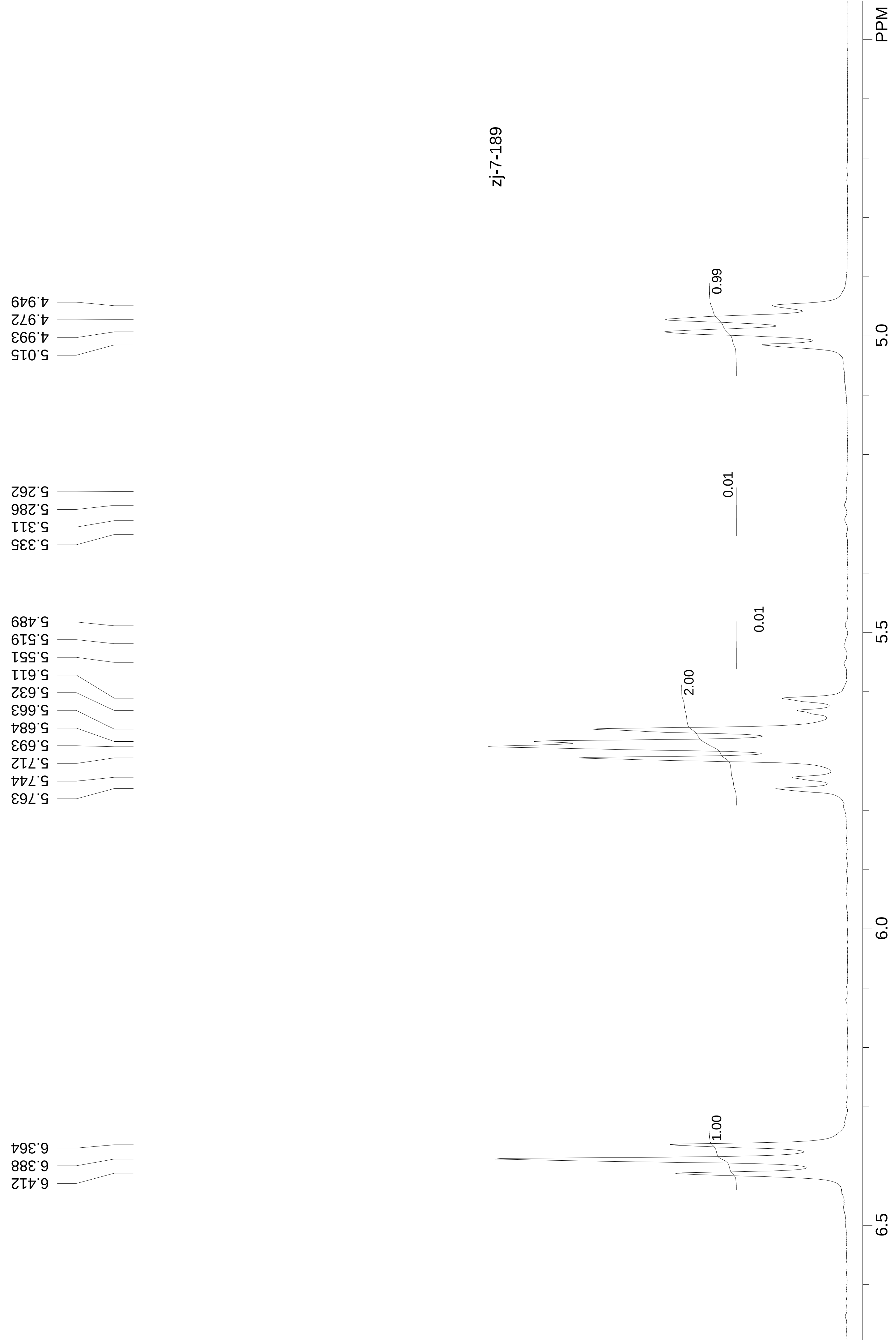

Supplementary Figure 214. ^{1}H NMR (300 MHz, $CDCl_3$) extended spectrum for xestospongiene E.

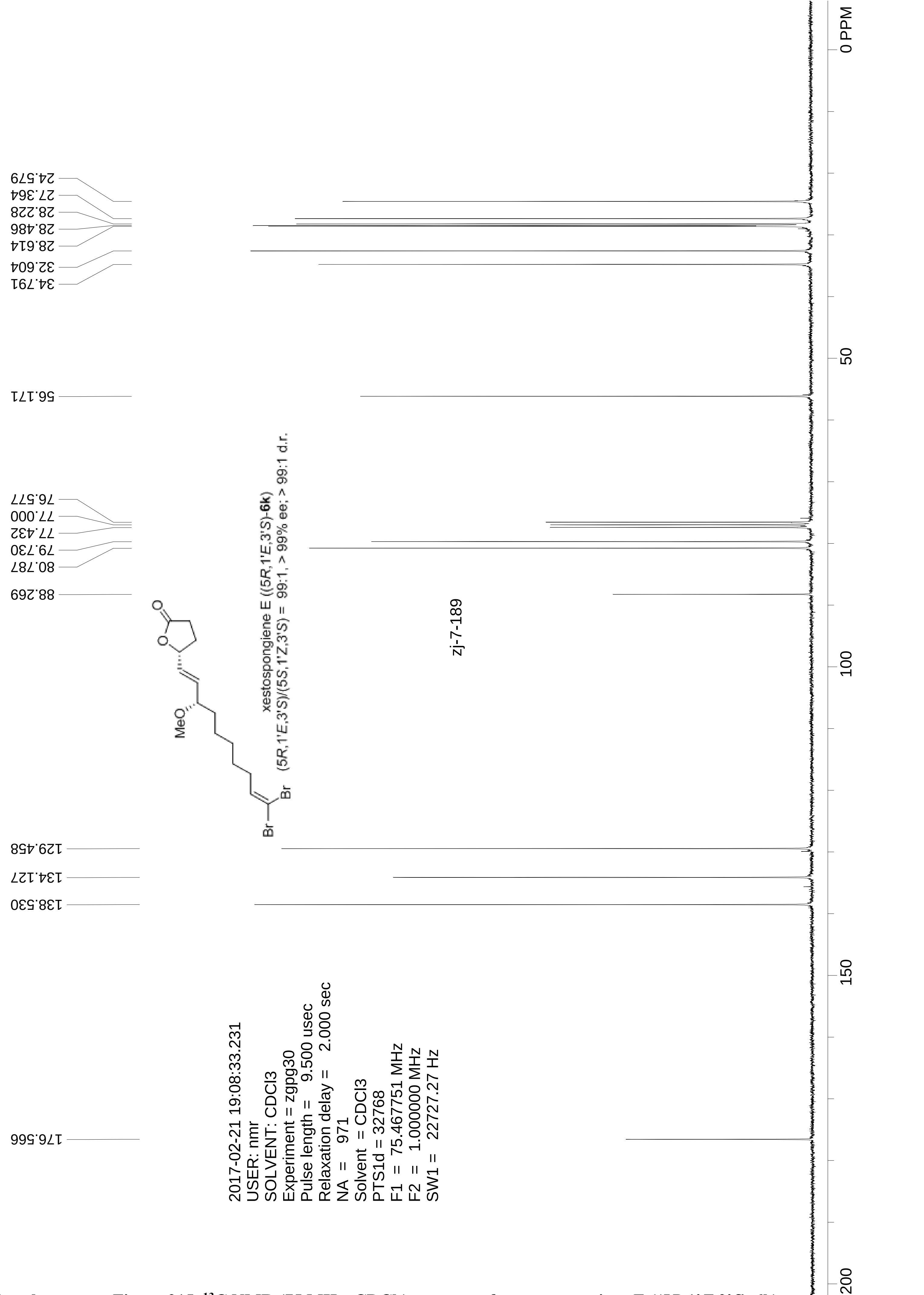

Supplementary Figure 215. ^{13}C NMR (75 MHz, $CDCl_3$) spectrum for xestospongiene E ((5*R*,1'*E*,3'*S*)-6k).

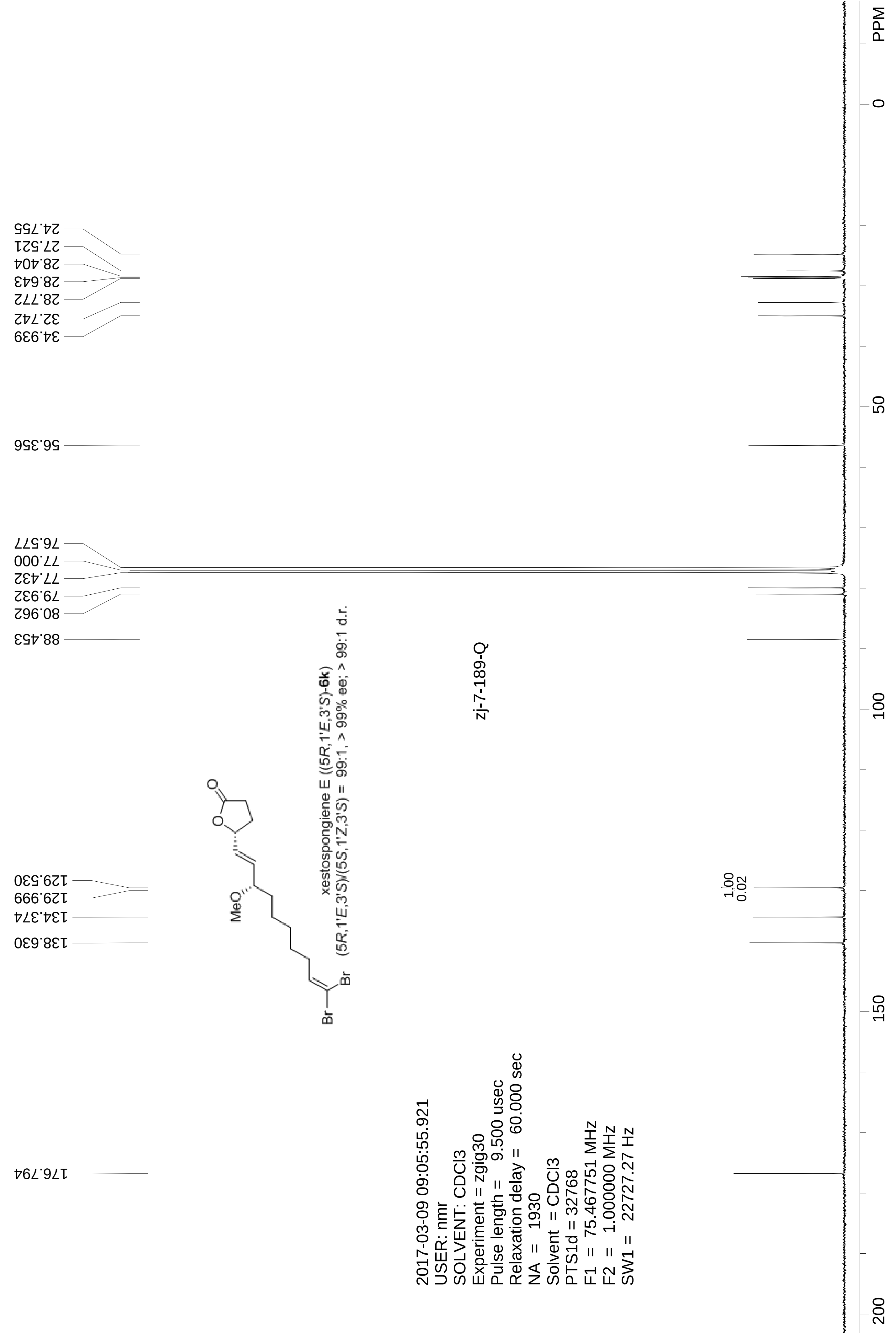

Supplementary Figure 216. Quantitative ^{13}C NMR (75 MHz, $CDCl_3$) spectrum for xestospongiene E.

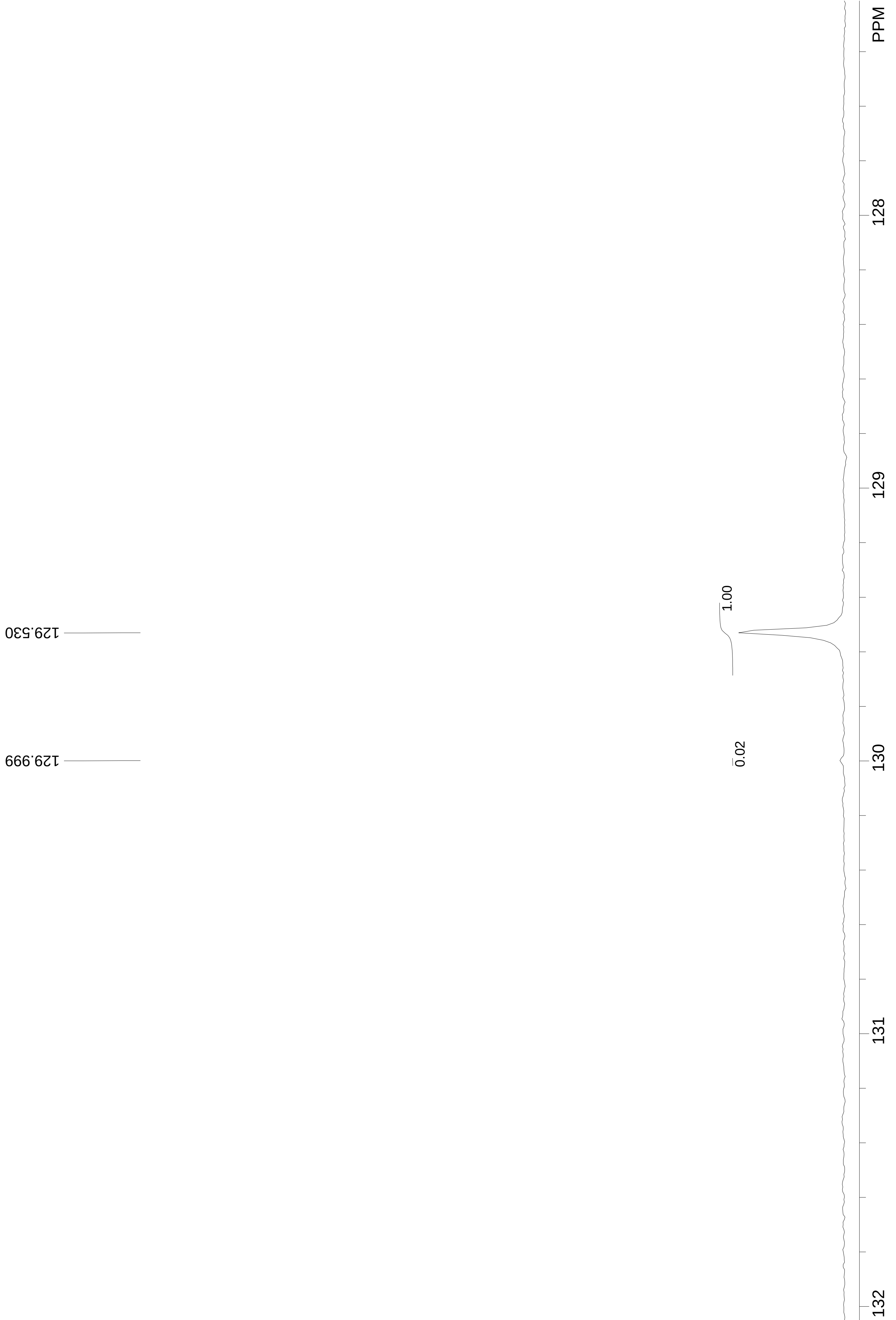

Supplementary Figure 217. Quantitative ^{13}C NMR (75 MHz, $CDCl_3$) extended spectrum for xestospongiene E.

Empower™ 3 SOFTWARE

SAMPLE INFORMATION

Sample Name:	ZJ7-189 ADH912200040	Acquired By:	System
Sample Type:		Date Acquired:	2017/3/22 16:53:38 CST
Vial:	1:A,3	Acq. Method Set:	chiral_isocratic
Injection:	1	Date Processed:	2017/3/23 14:18:28 CST
Injection Volume:	3.00 ul	Processing Methoc	1
Run Time:	30.0 Minutes	Channel Name:	PDA Ch1 214 nm@1.2 nm
Sample Set Name	20170322	Proc. Chnl. Descr:	PDA Ch1 214 nm@1.2 nm

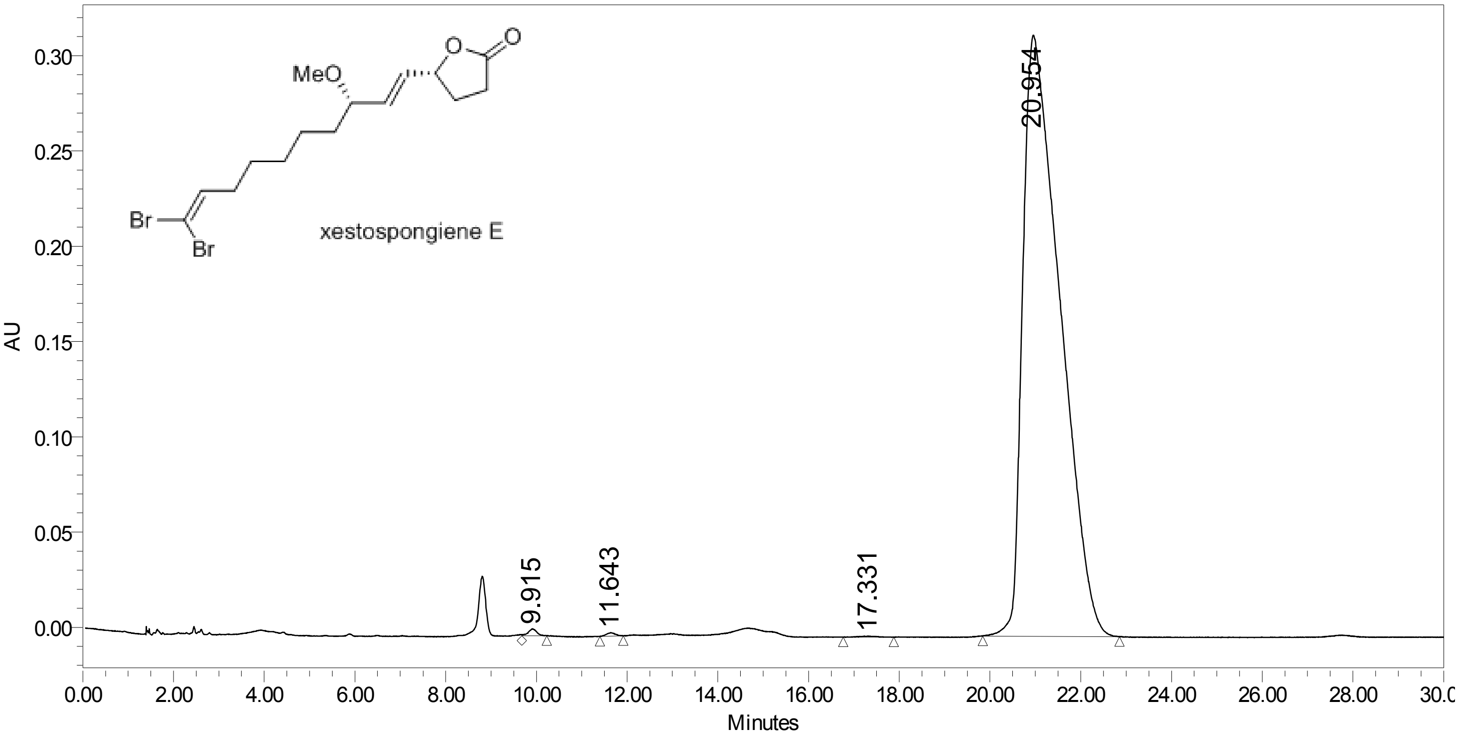

Peak Results

	RT	Area	Height	% Area
1	9.915	47327	3602	0.26
2	11.643	24645	1837	0.14
3	17.331	10850	486	0.06
4	20.954	17914997	315459	99.54

Supplementary Figure 218. SFC spectrum for xestospongiene E.

Empower™ 3 SOFTWARE

SAMPLE INFORMATION

Sample Name:	ZJ7-158 adh912214200040	Acquired By:	System
Sample Type:		Date Acquired:	2017/3/22 14:45:20 CST
Vial:	1:A,1	Acq. Method Set:	chiral_isocratic
Injection:	1	Date Processed:	2017/3/23 14:07:00 CST
Injection Volume:	5.00 ul	Processing Methoc	1
Run Time:	30.0 Minutes	Channel Name:	PDA Ch1 214 nm@1.2 nm
Sample Set Name	20170213	Proc. Chnl. Descr:	PDA Ch1 214 nm@1.2 nm

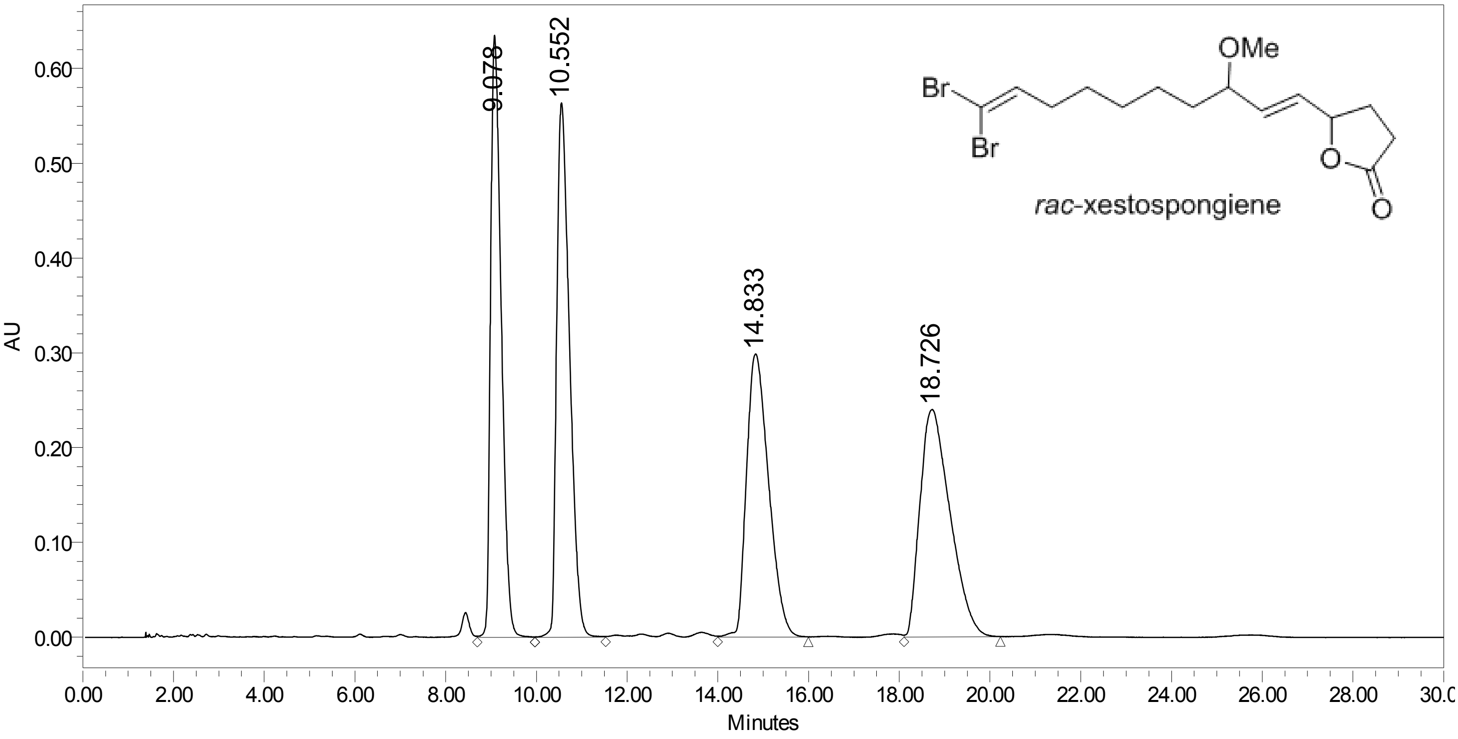

Peak Results

	RT	Area	Height	% Area
1	9.078	10369086	635306	24.26
2	10.552	1117745[illegible]	563713	26.15
3	14.833	10145220	298583	23.73
4	18.726	1105206[illegible]	239962	25.86

Supplementary Figure 219. SFC spectrum for *rac*-xestospongiene.

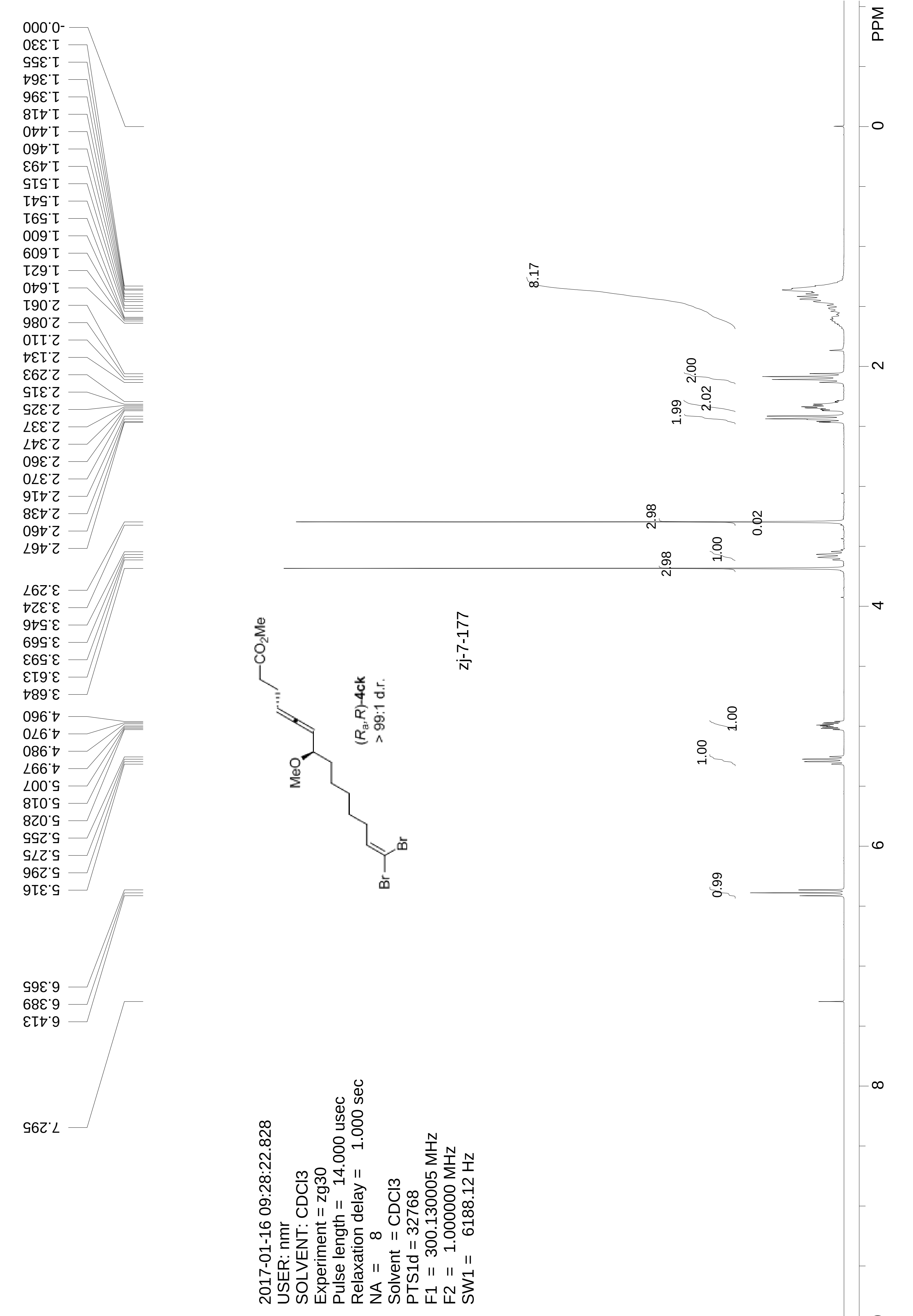

Supplementary Figure 220. **^{1}H NMR (300 MHz, $CDCl_3$) spectrum for (R_a,R)-4ck.**

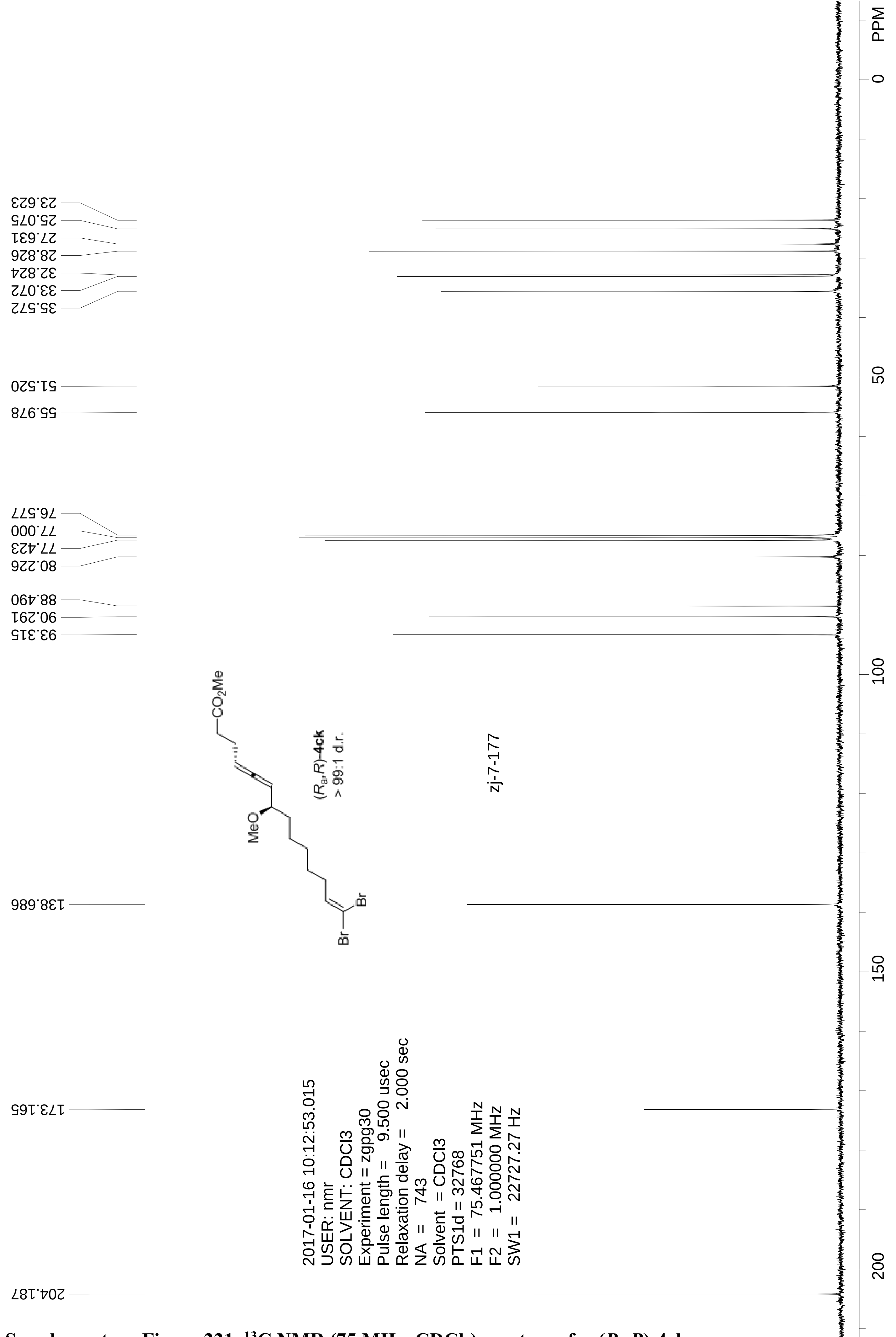

Supplementary Figure 221. ^{13}C NMR (75 MHz, $CDCl_3$) spectrum for (*R*$_a$,*R*)-4ck.

zj-7-177-if-3-100-1-1-214

实验时间：2017-01-17,10:38:27　　报告时间：2017-01-17,12:43:29
谱图文件:F:\zhuguangjiong\zj\20170117\zj-7-177-if-3-100-1-1-214.org

实验内容简介：

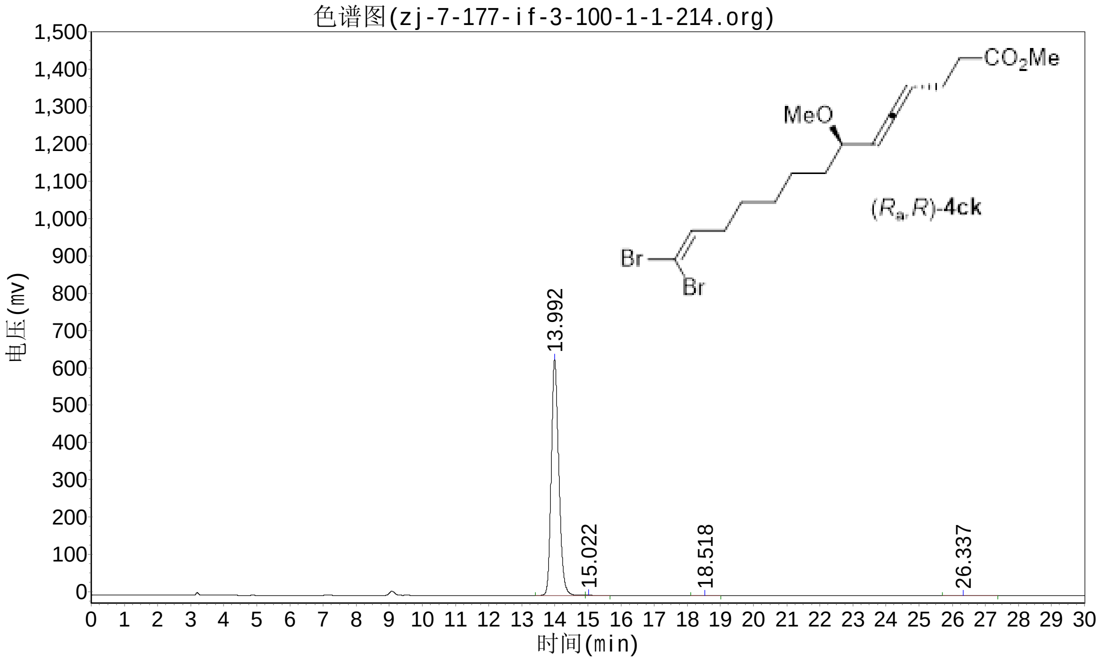

分析结果表

峰号	峰名	保留时间	峰高	峰面积	含量
1		13.992	632933.125	10014403.000	99.5776
2		15.022	595.728	11957.195	0.1189
3		18.518	245.622	4872.408	0.0484
4		26.337	688.221	25650.504	0.2551
总计			634462.696	10056883.107	100.0000

Supplementary Figure 222. HPLC spectrum for (R_a,R)-4ck.

zj-7-114-if-3-100-1-1-214

实验时间：2017-01-17,11:12:52　　　　报告时间：2017-01-17,12:44:44
谱图文件:F:\zhuguangjiong\zj\20170117\zj-7-114-if-3-100-1-1-214.org

实验内容简介：

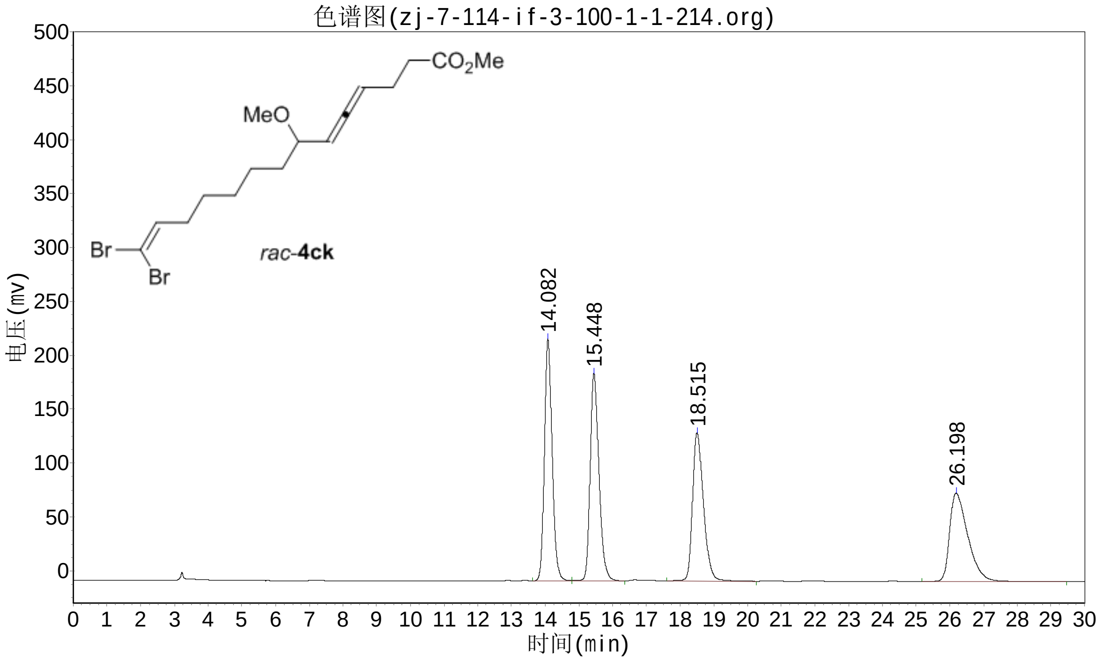

分析结果表

峰号	峰名	保留时间	峰高	峰面积	含量
1		14.082	224085.547	3595589.250	26.9346
2		15.448	193008.625	3533020.000	26.4659
3		18.515	137829.547	3136710.500	23.4971
4		26.198	81985.172	3084028.500	23.1025
总计			636908.891	13349348.250	100.0000

Supplementary Figure 223. HPLC spectrum for *rac*-4ck.

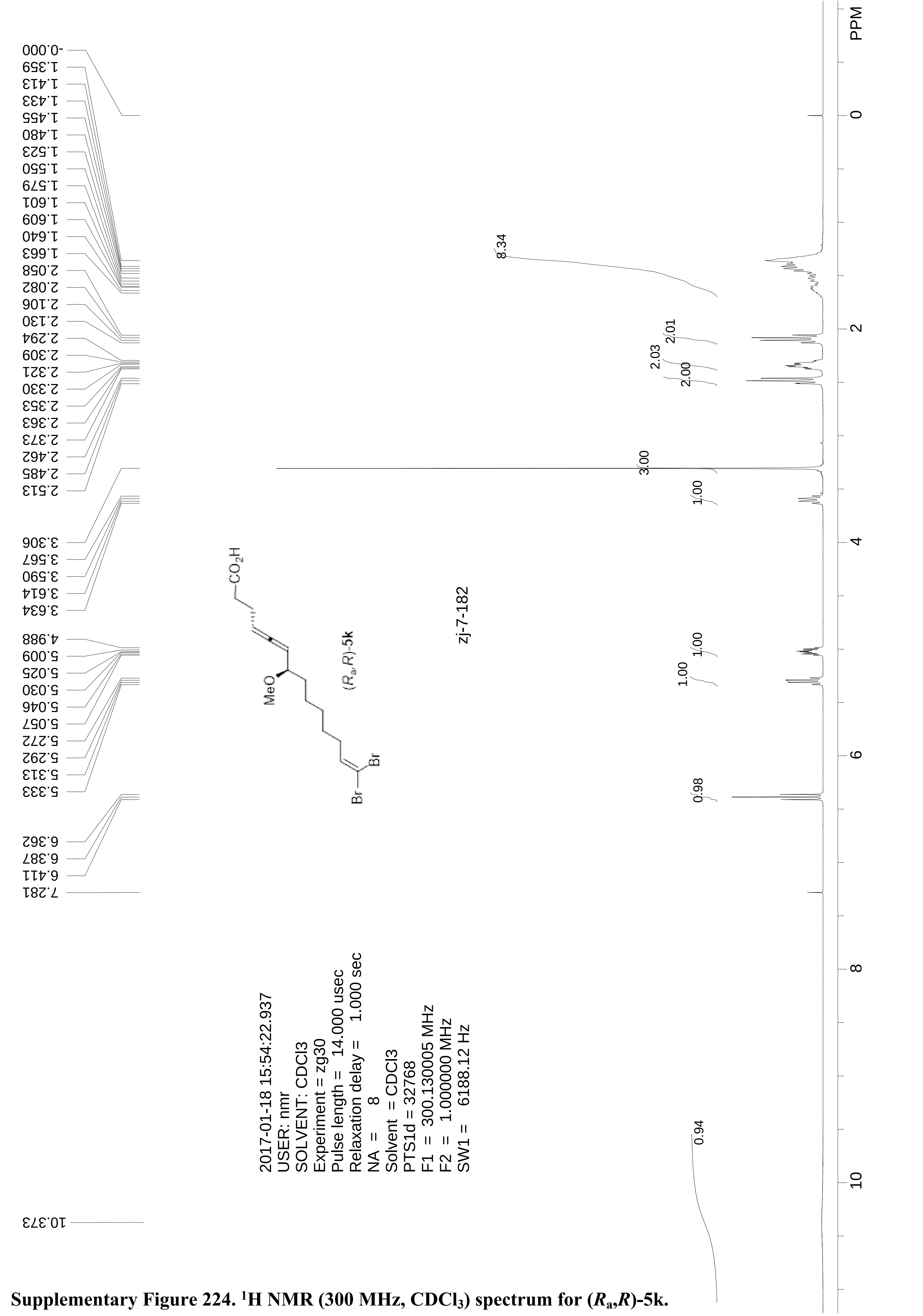

Supplementary Figure 224. **^{1}H NMR (300 MHz, $CDCl_3$) spectrum for (*R*$_a$,*R*)-5k.**

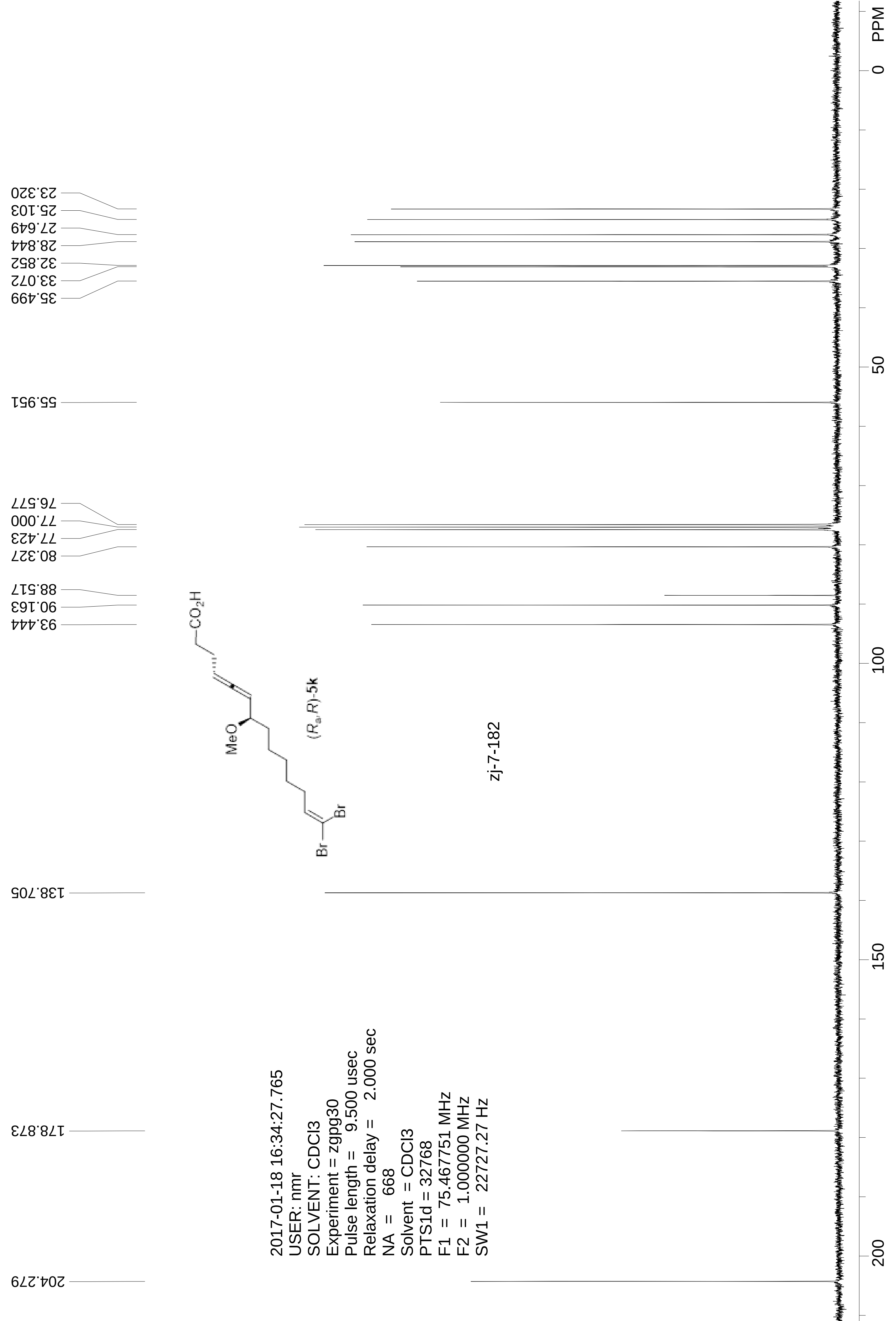

Supplementary Figure 225. ^{13}C NMR (75 MHz, $CDCl_3$) spectrum for (R_a,R)-5k.

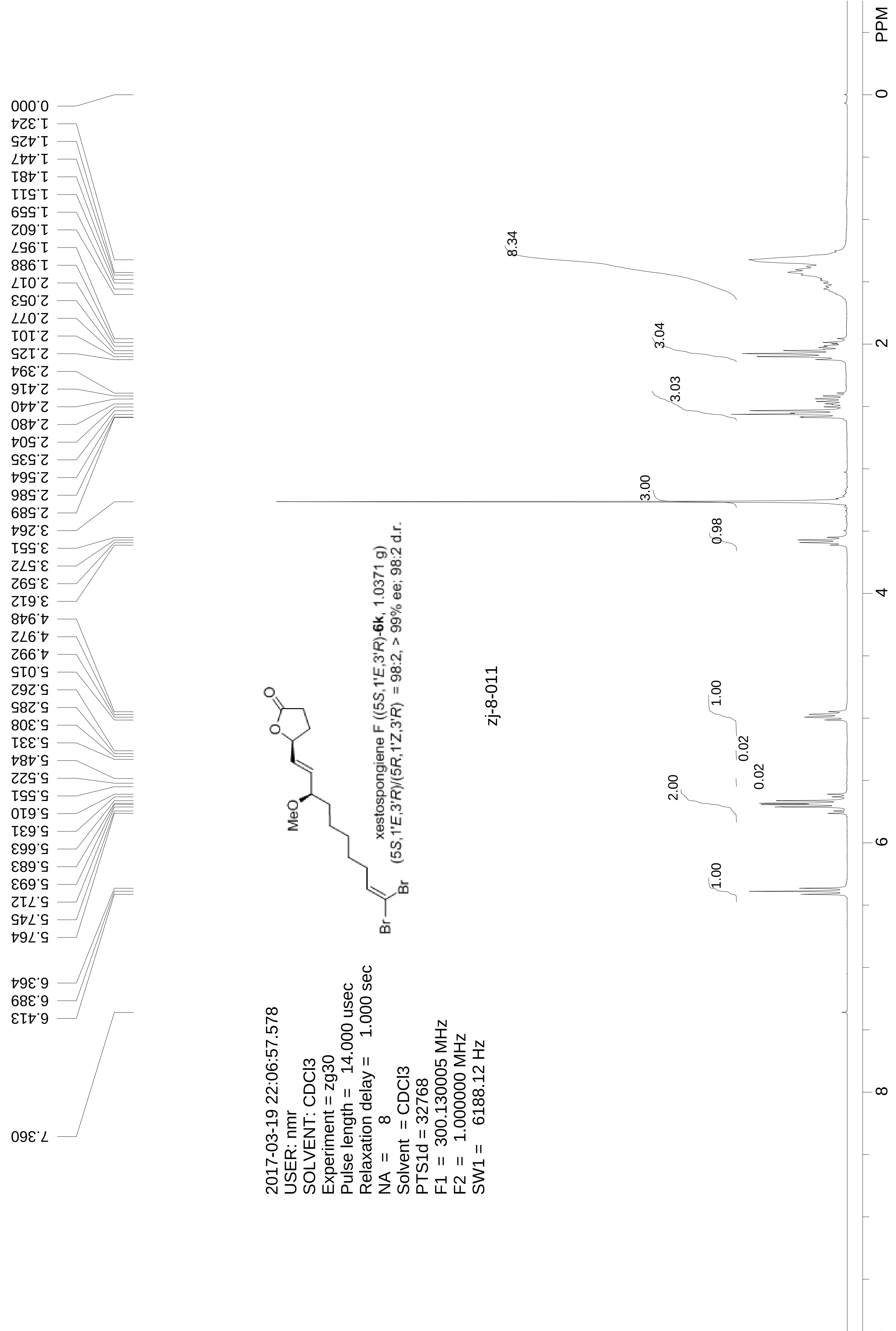

Supplementary Figure 226. **^{1}H NMR (300 MHz, $CDCl_3$) spectrum for xestospongiene F.**

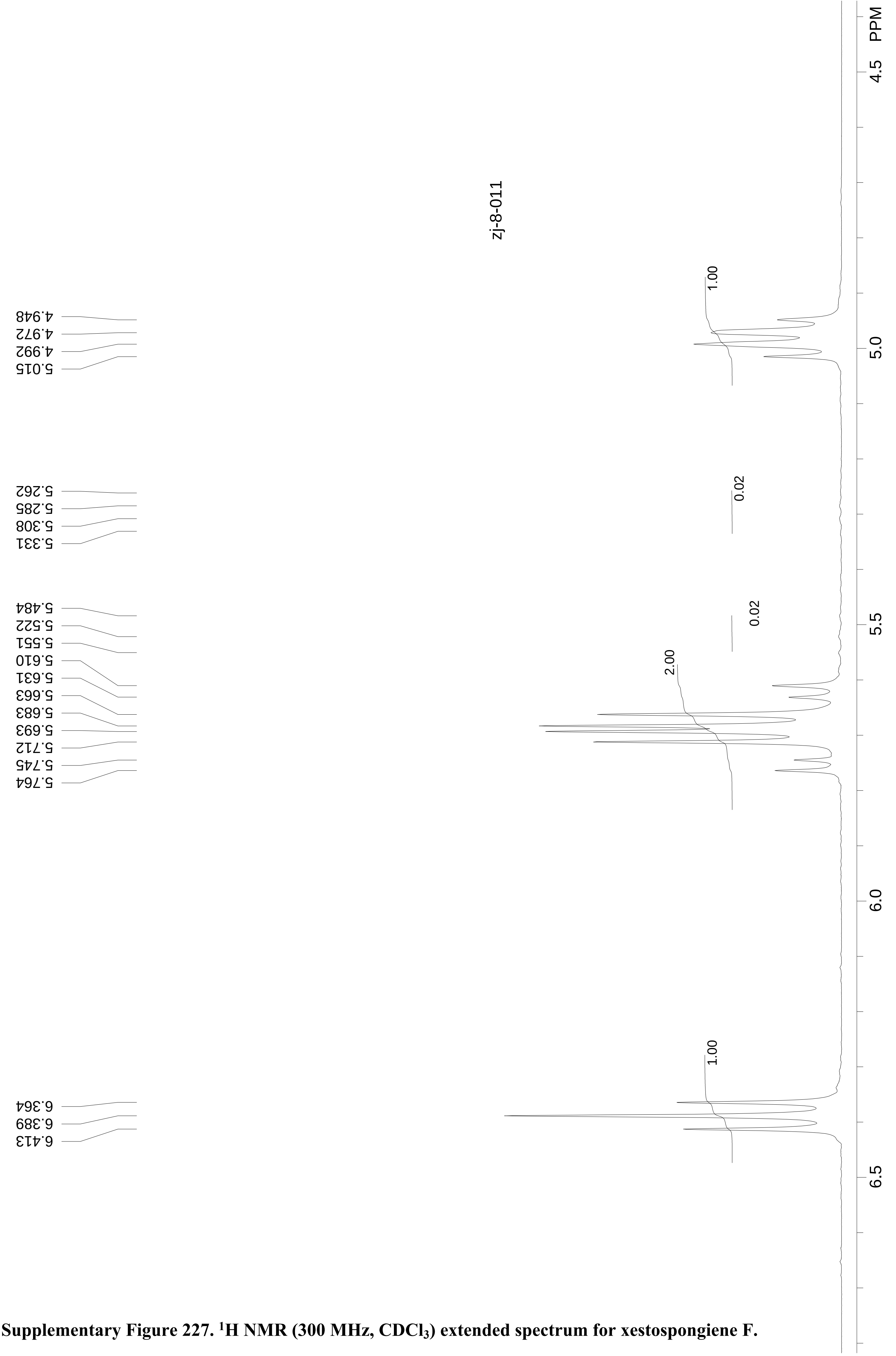

Supplementary Figure 227. ^{1}**H NMR (300 MHz, CDCl$_3$) extended spectrum for xestospongiene F.**

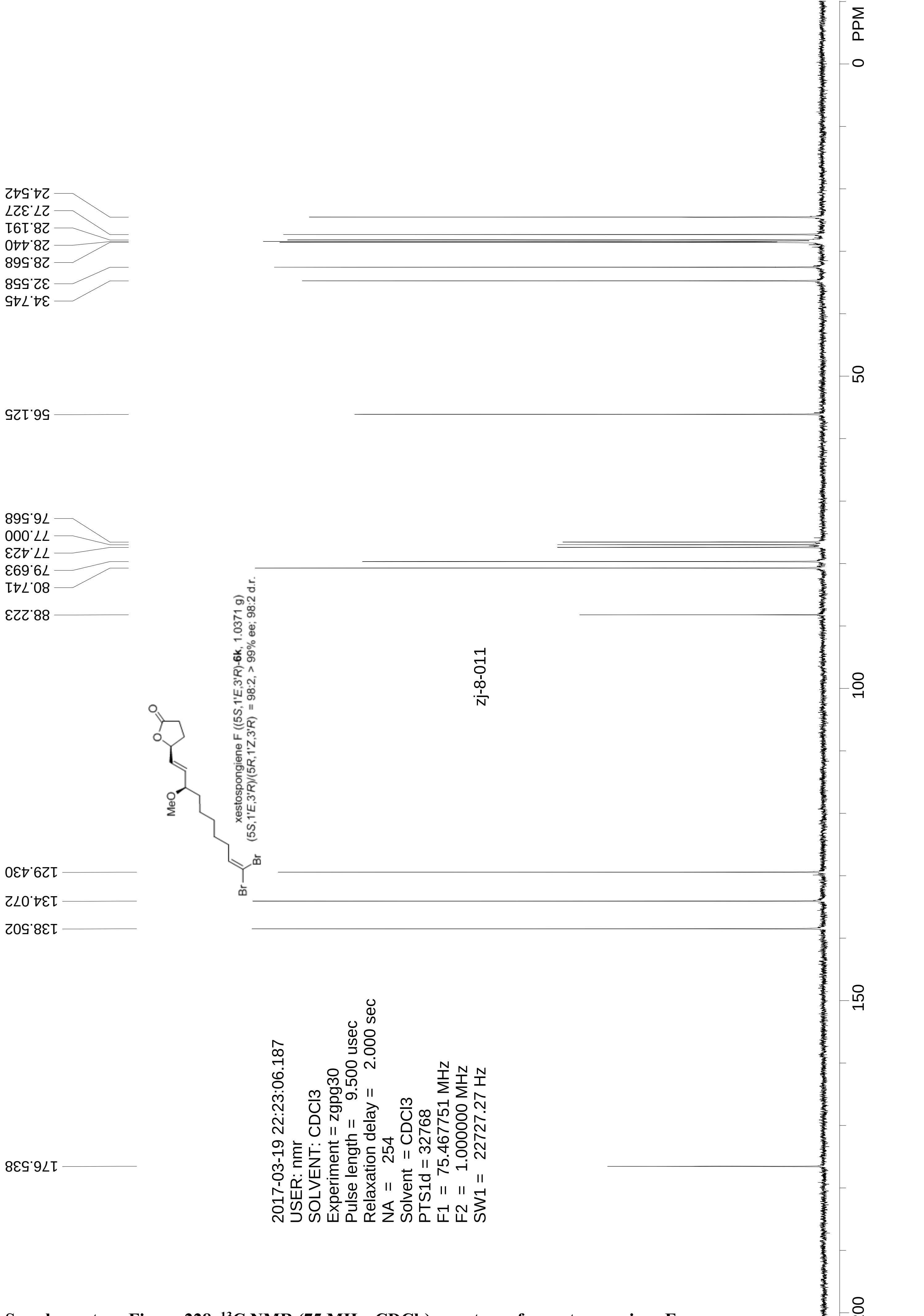

Supplementary Figure 228. ^{13}**C NMR (75 MHz,** $CDCl_3$**) spectrum for xestospongiene F.**

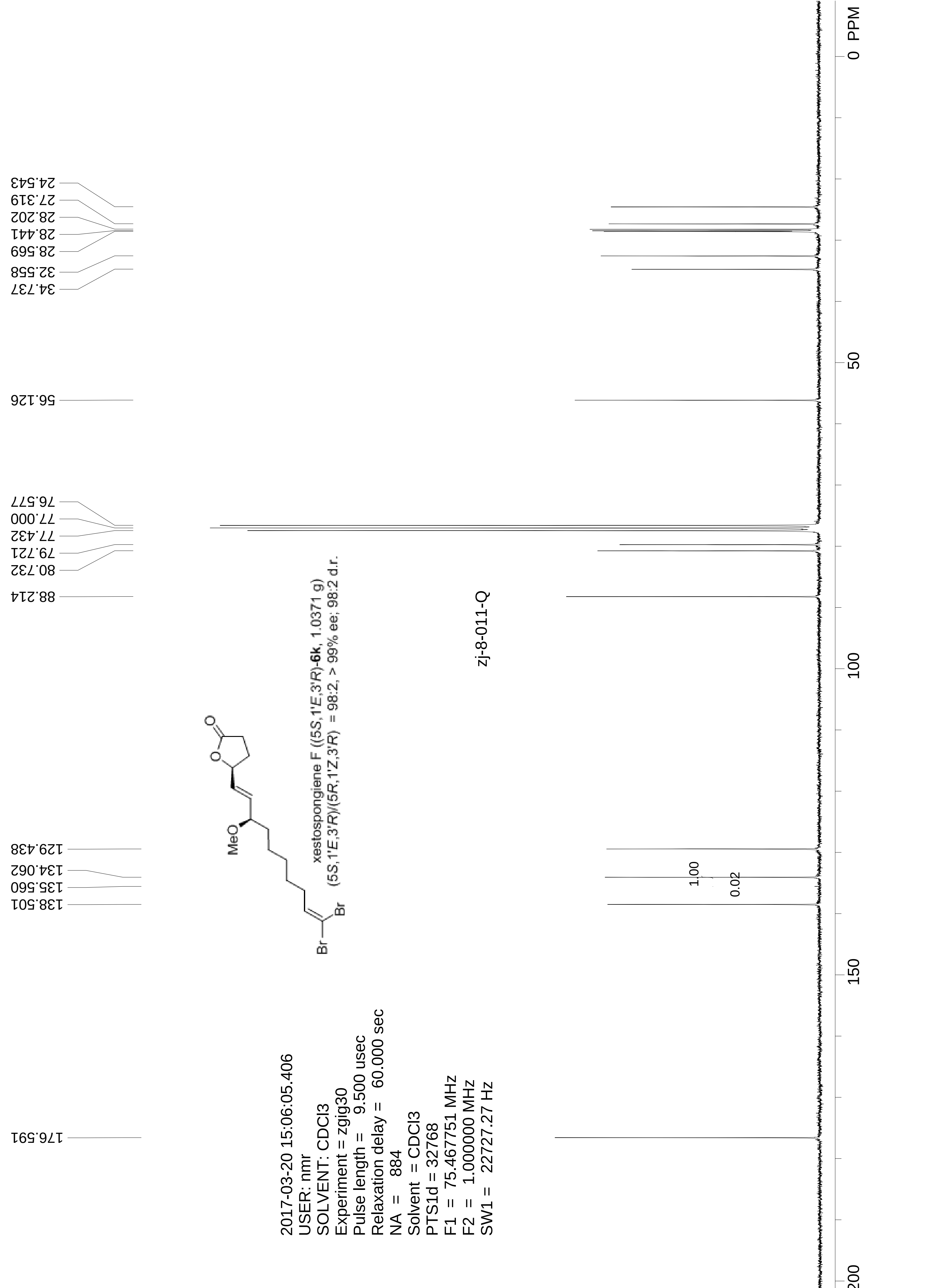

Supplementary Figure 229. Quantitative ^{13}C NMR (75 MHz, $CDCl_3$) spectrum for xestospongiene F.

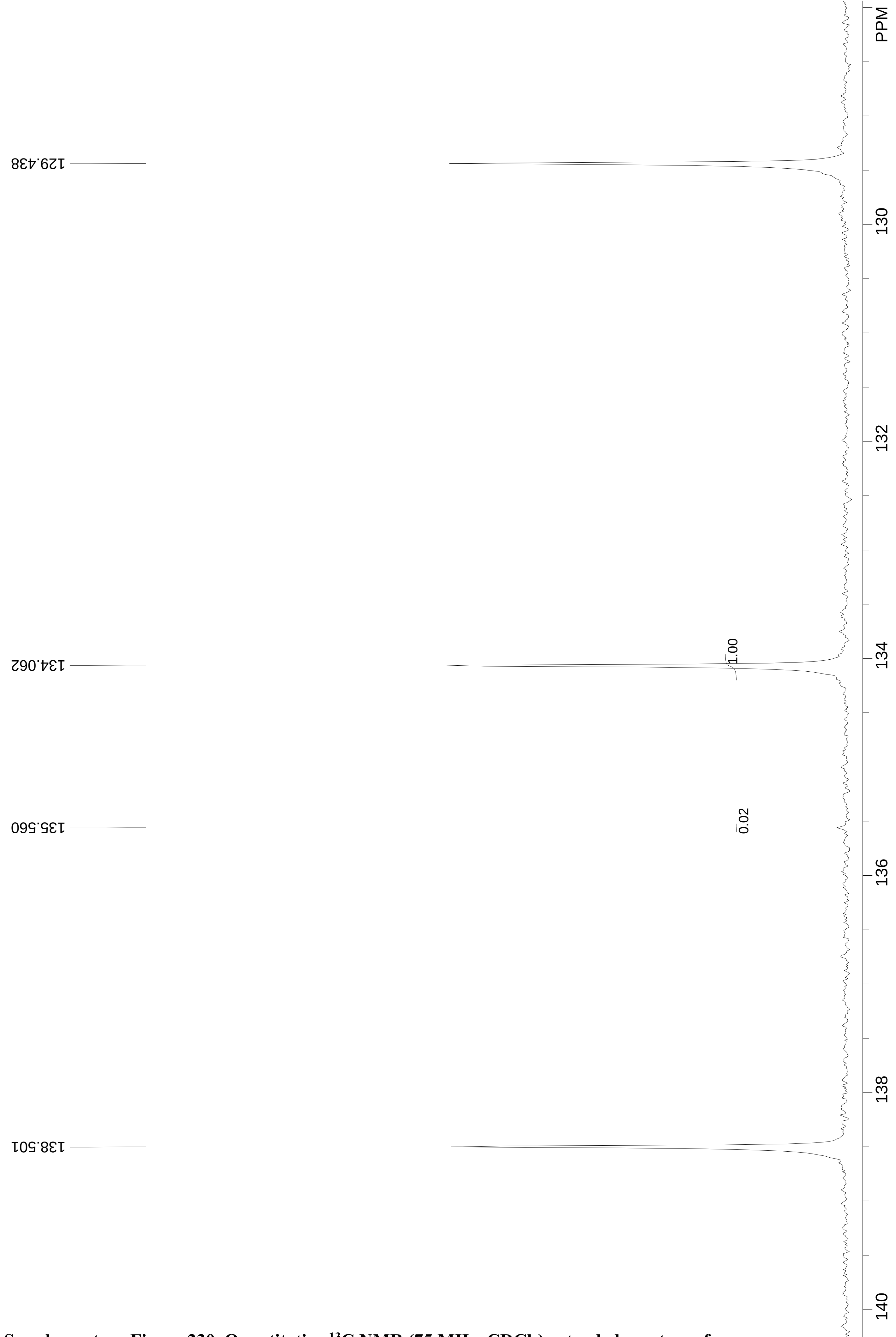

Supplementary Figure 230. Quantitative ^{13}C NMR (75 MHz, $CDCl_3$) extended spectrum for xestospongiene F.

Empower™ 3 SOFTWARE

SAMPLE INFORMATION

Sample Name:	ZJ8011 ADH912200040	Acquired By:	System
Sample Type:		Date Acquired:	2017/3/22 17:32:01 CST
Vial:	1:A,4	Acq. Method Set:	chiral_isocratic
Injection:	1	Date Processed:	2017/3/23 14:19:00 CST
Injection Volume:	3.00 ul	Processing Methoc	1
Run Time:	30.0 Minutes	Channel Name:	PDA Ch1 214 nm@1.2 nm
Sample Set Name	20170322	Proc. Chnl. Descr:	PDA Ch1 214 nm@1.2 nm

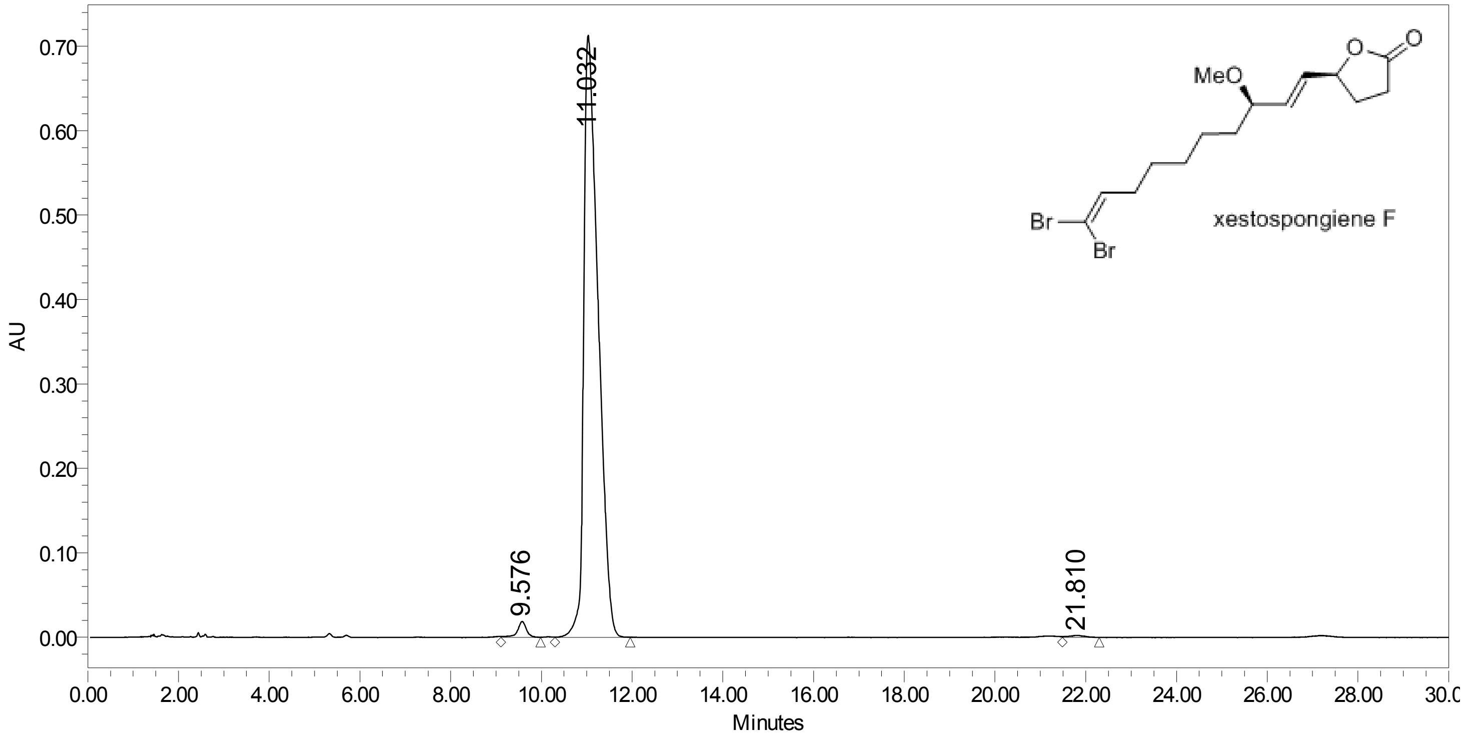

Peak Results

	RT	Area	Height	% Area
1	9.576	259455	18854	1.62
2	11.032	15714231	713261	98.03
3	21.810	56440	2248	0.35

Supplementary Figure 231. SFC spectrum for xestospongiene F.

SAMPLE INFORMATION

Sample Name:	ZJ7-158 adh912214200040	Acquired By:	System
Sample Type:		Date Acquired:	2017/3/22 14:45:20 CST
Vial:	1:A,1	Acq. Method Set:	chiral_isocratic
Injection:	1	Date Processed:	2017/3/23 14:07:00 CST
Injection Volume:	5.00 ul	Processing Methoc	1
Run Time:	30.0 Minutes	Channel Name:	PDA Ch1 214 nm@1.2 nm
Sample Set Name	20170213	Proc. Chnl. Descr:	PDA Ch1 214 nm@1.2 nm

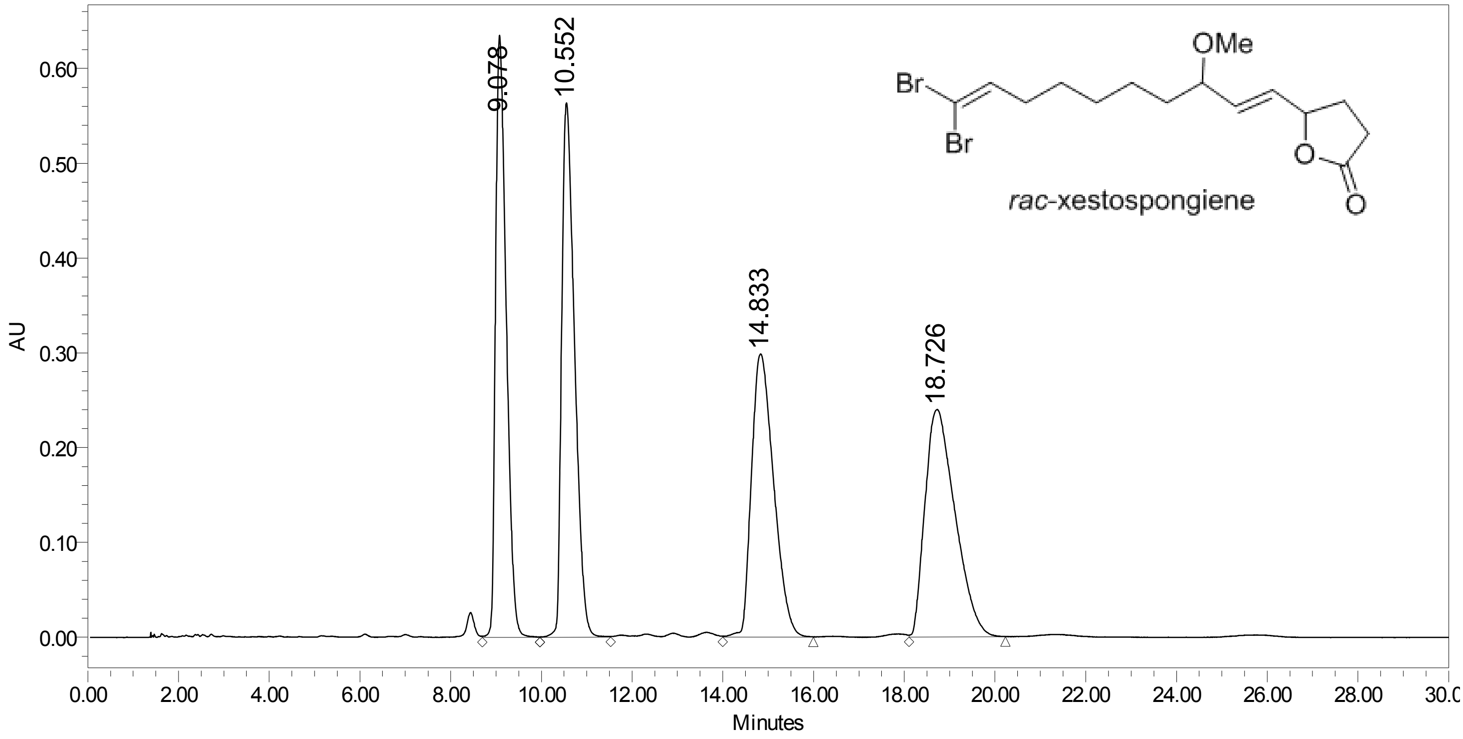

Peak Results

	RT	Area	Height	% Area
1	9.078	10369086	635306	24.26
2	10.552	1117745[illegible]	563713	26.15
3	14.833	10145220	298583	23.73
4	18.726	1105206[illegible]	239962	25.86

Supplementary Figure 232. SFC spectrum for *rac*-xestospongiene.

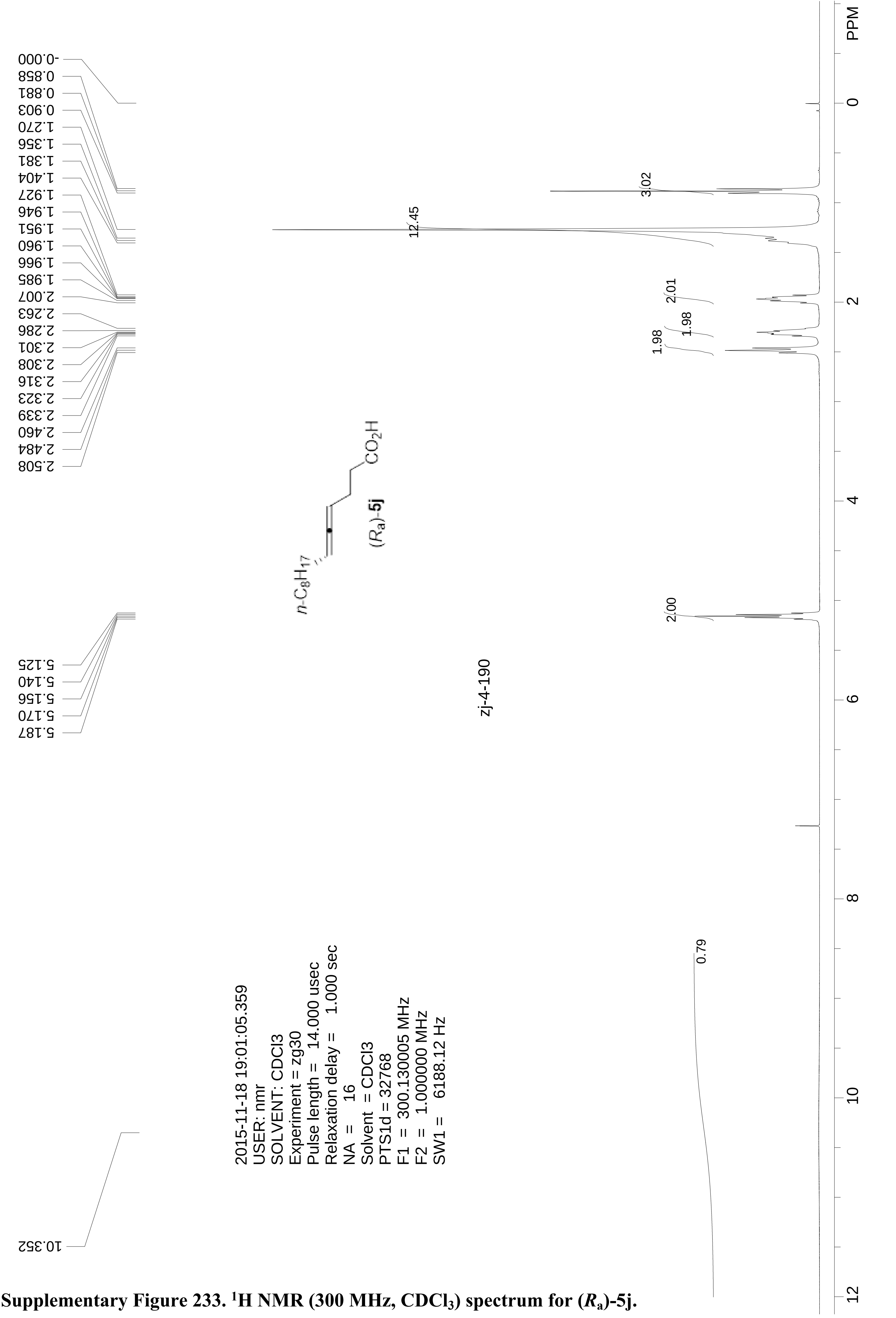

Supplementary Figure 233. ^{1}H NMR (300 MHz, $CDCl_3$) spectrum for (R_a)-5j.

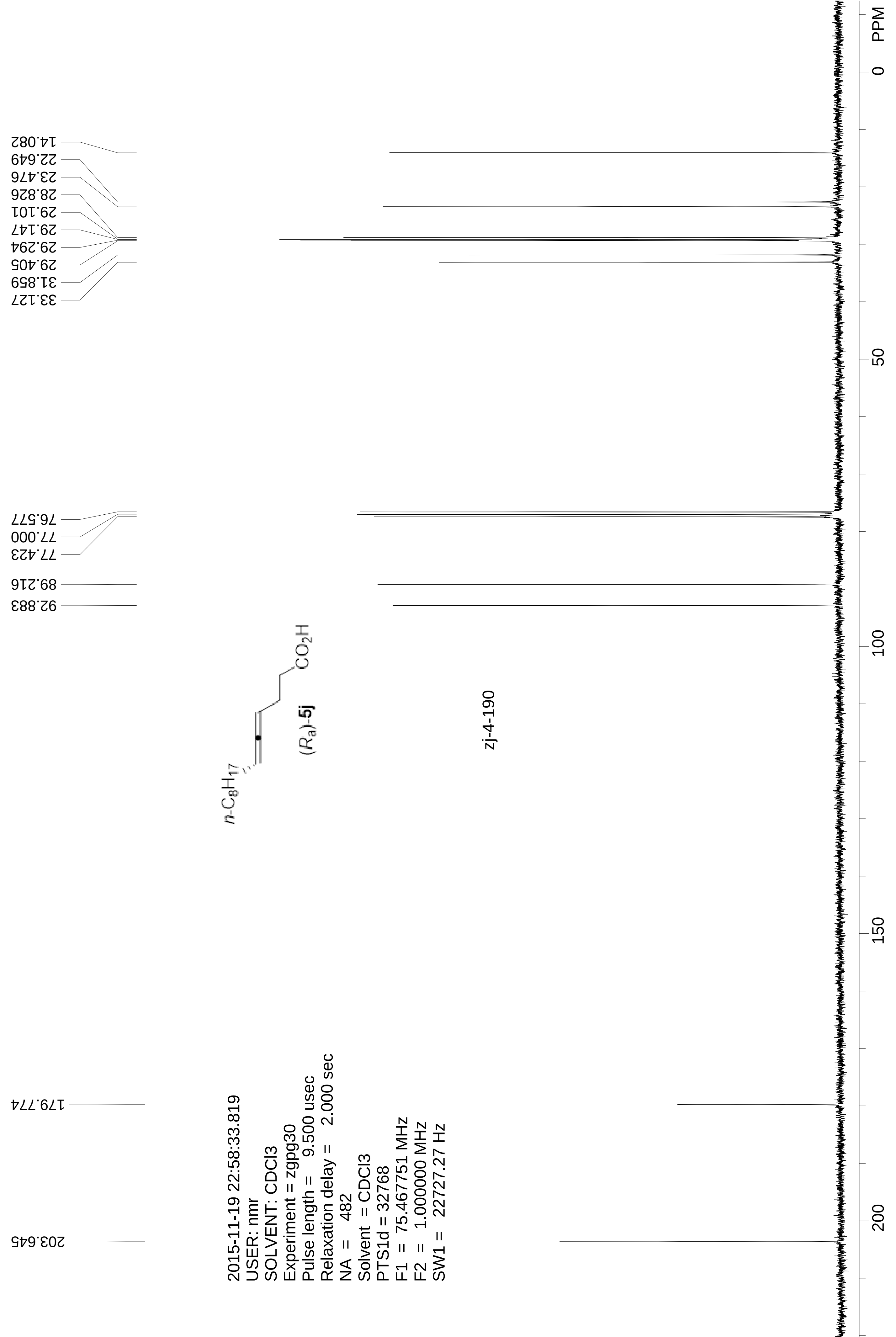

Supplementary Figure 234. ^{13}C NMR (75 MHz, $CDCl_3$) spectrum for (R_a)-5j.

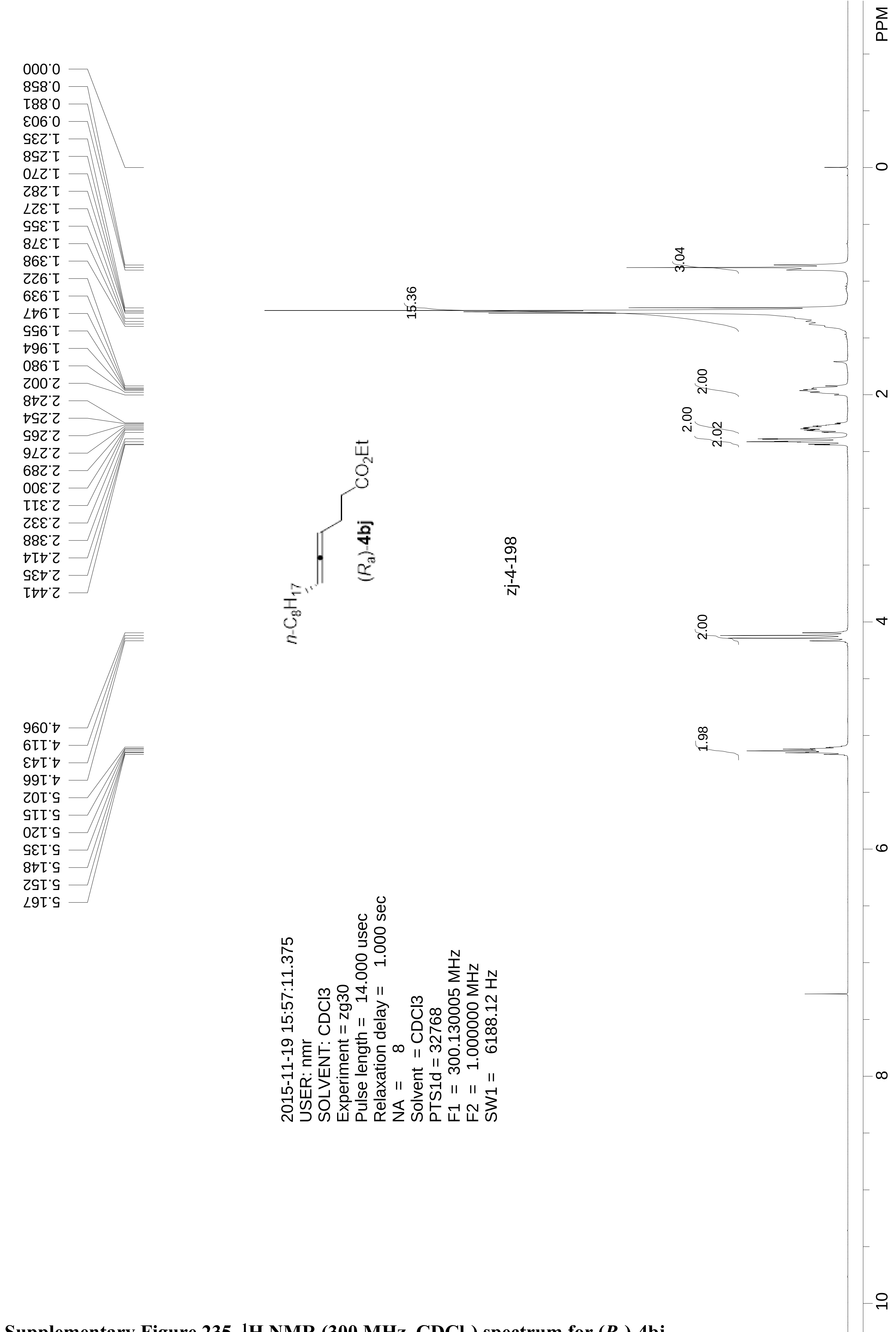

Supplementary Figure 235. ^{1}H NMR (300 MHz, CDCl$_3$) spectrum for (R_a)-4bj.

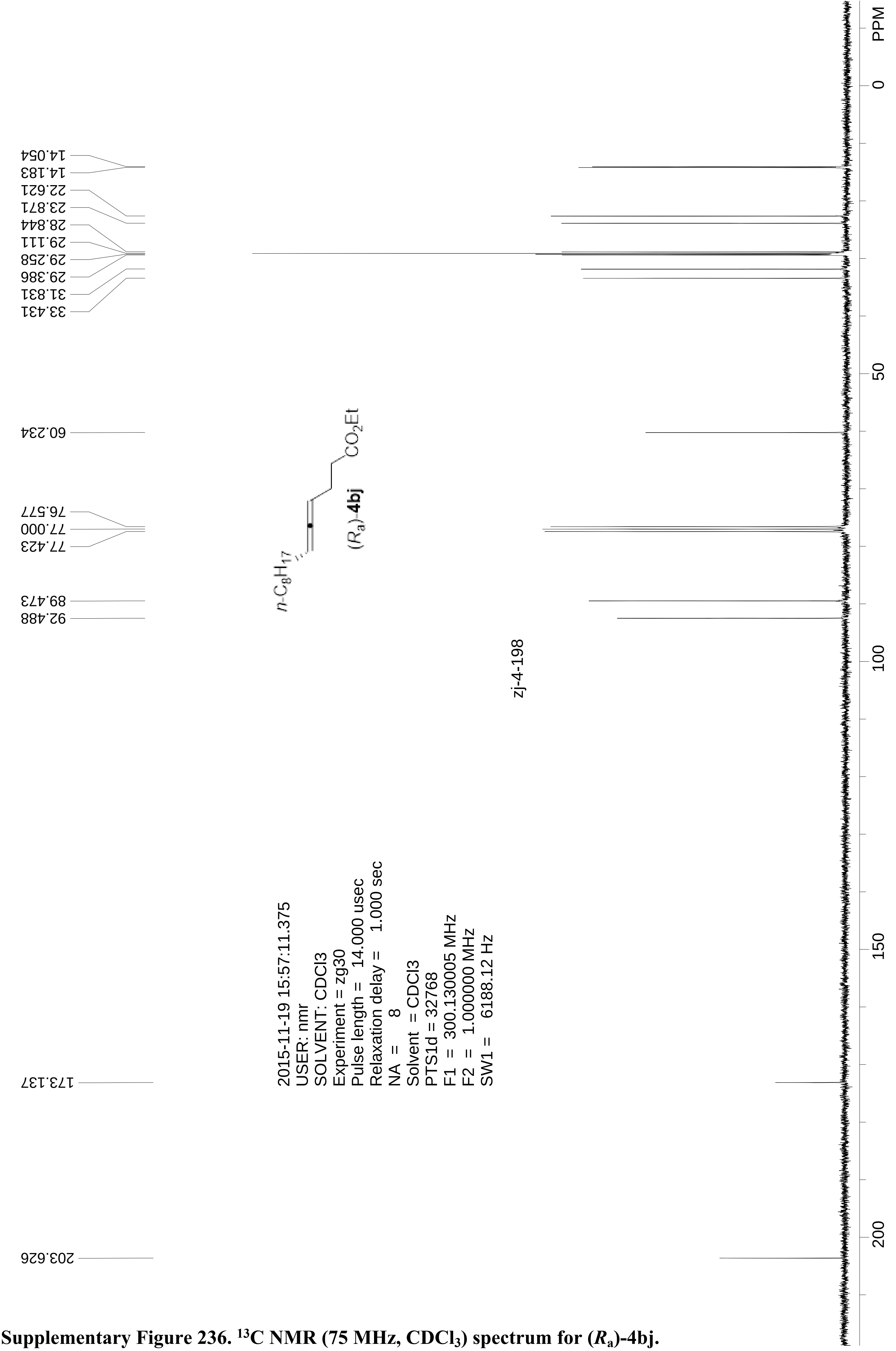

Supplementary Figure 236. ^{13}C NMR (75 MHz, $CDCl_3$) spectrum for (*R*$_a$)-4bj.

zj-4-198-oz-h-400-1-0.5-214

实验时间：2016-01-04,12:32:12　　　　　　　　　　报告时间：2016-01-05,17:42:24
谱图文件:D:\zhuguangjiong\zj\20160104\zj-4-198-oz-h-400-1-0.5-214..org

实验内容简介：

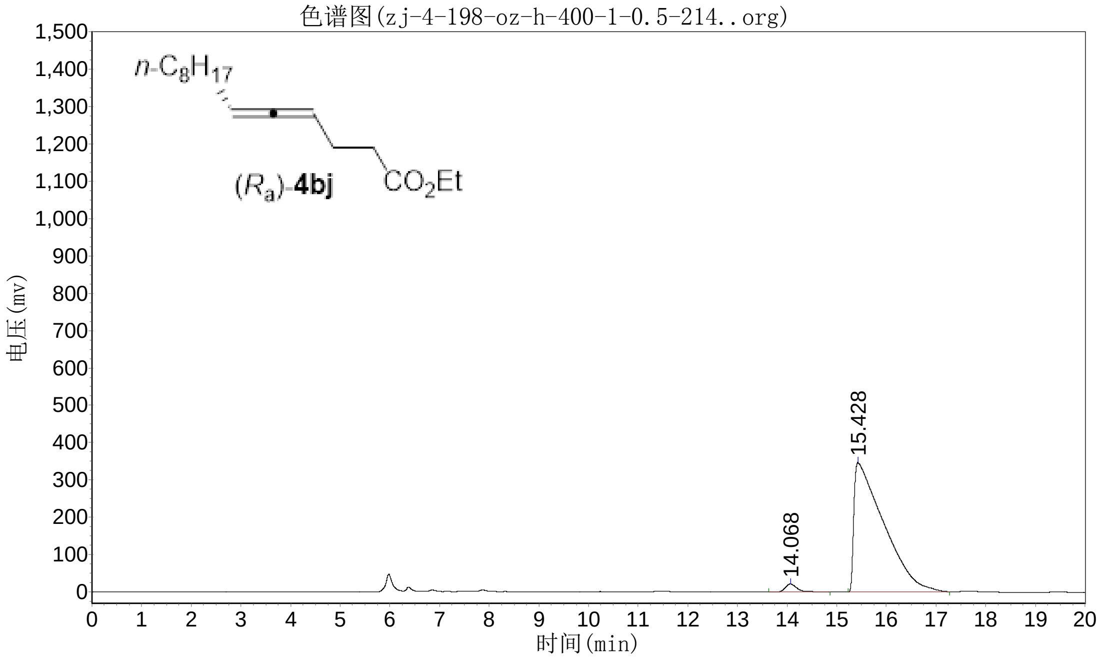

分析结果表

峰号	峰名	保留时间	峰高	峰面积	含量
1		14.068	21746.184	377848.406	2.5469
2		15.428	346879.906	14457886.000	97.4531
总计			368626.090	14835734.406	100.0000

Supplementary Figure 237. HPLC spectrum for (R_a)-4bj.

zj-4-197-oz-h-400-1-0.5-214

实验时间：2016-01-04,11:44:12　　　　　　　　　　　　报告时间：2016-01-05,17:40:22
谱图文件:d:\zhuguangjiong\zj\20160104\zj-4-197-oz-h-400-1-0.5-214.org

实验内容简介：

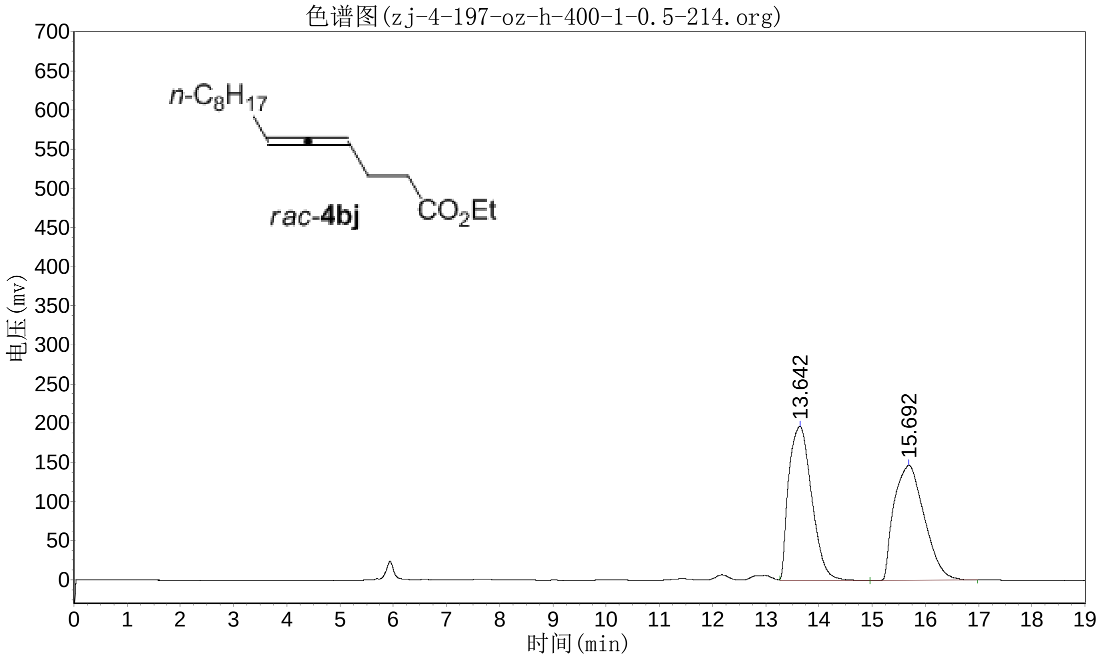

分析结果表

峰号	峰名	保留时间	峰高	峰面积	含量
1		13.642	197026.484	5910192.000	50.4442
2		15.692	147035.156	5806109.000	49.5558
总计			344061.641	11716301.000	100.0000

Supplementary Figure 238. HPLC spectrum for *rac*-4bj.

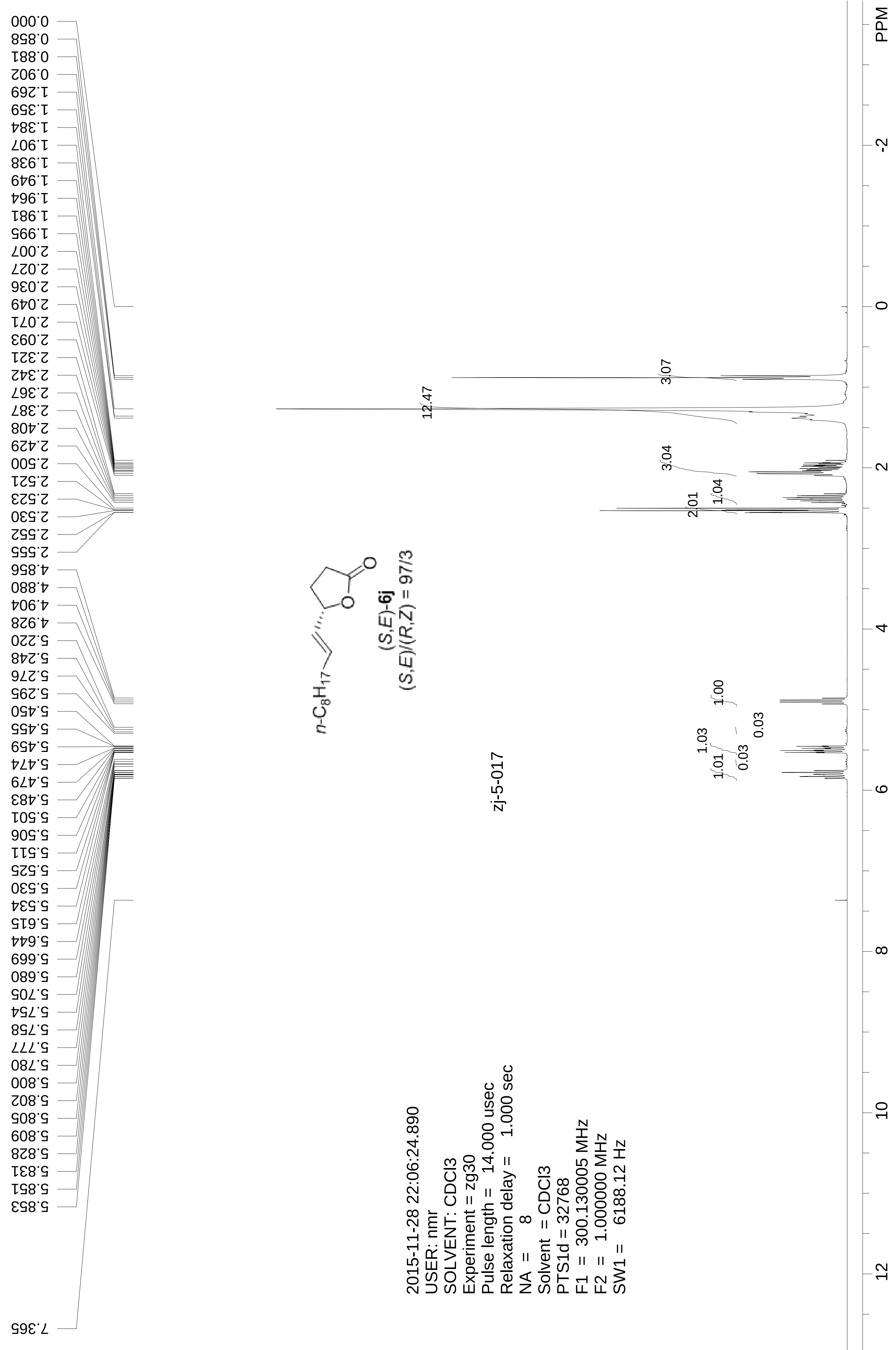

Supplementary Figure 239. ^{1}H NMR (300 MHz, $CDCl_3$) spectrum for (*S*,*E*)-6j.

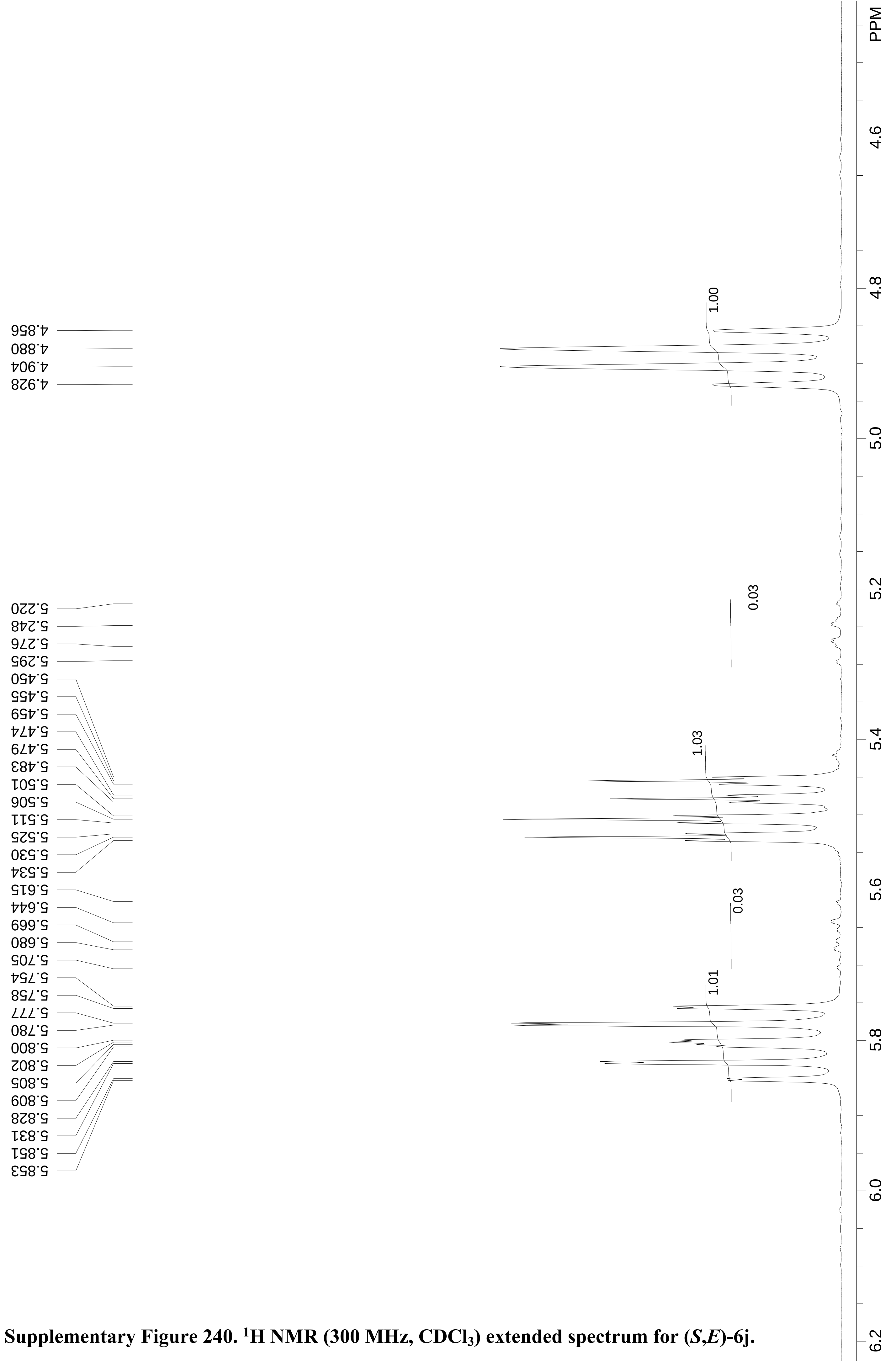

Supplementary Figure 240. ^{1}H NMR (300 MHz, $CDCl_3$) extended spectrum for (*S*,*E*)-6j.

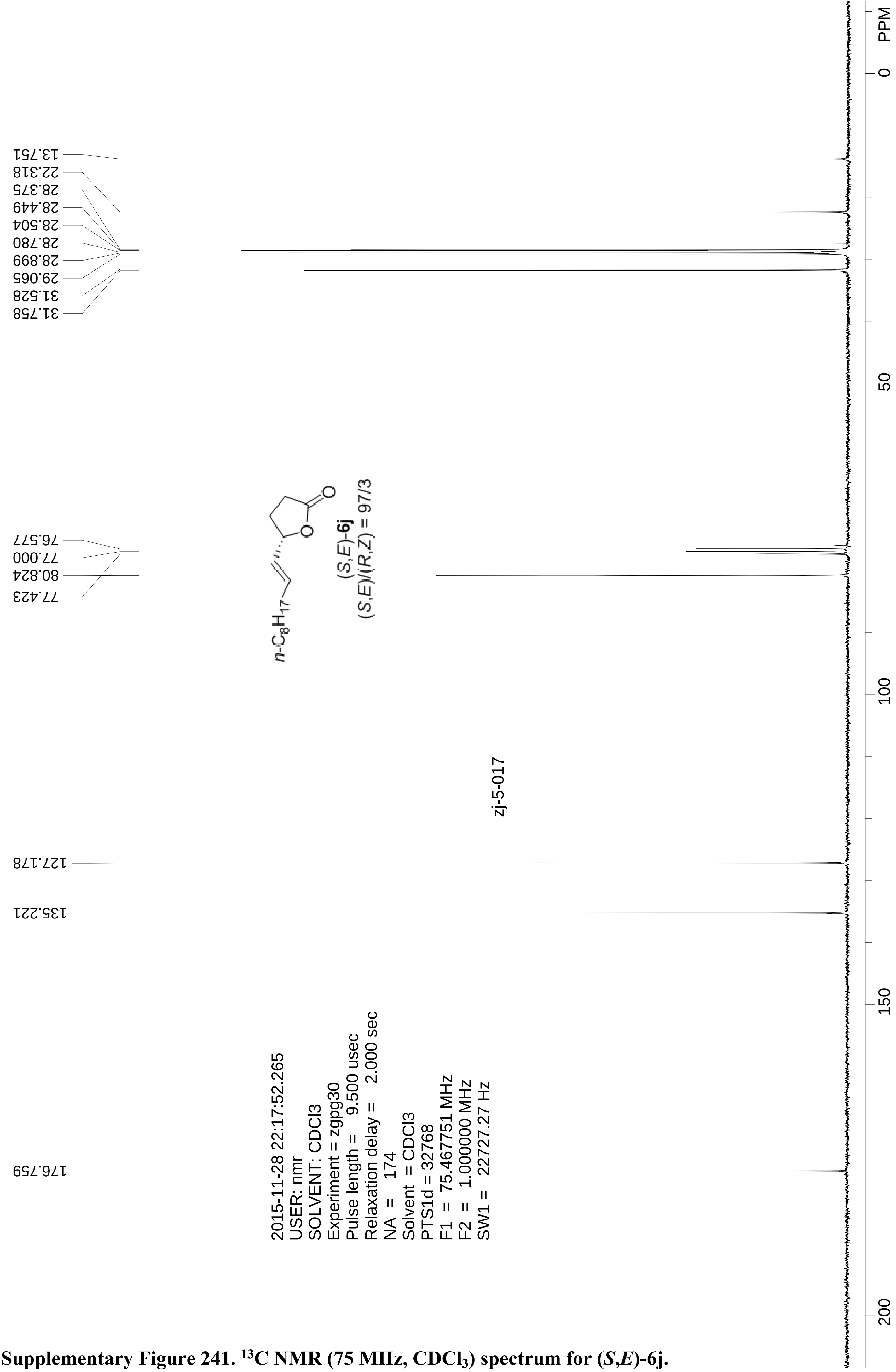

Supplementary Figure 241. ^{13}C NMR (75 MHz, CDCl$_3$) spectrum for (*S*,*E*)-6j.

zj-5-017-oj-h-100-1-1-214

实验时间：2016/1/7,15:51:30　　　　　　　　　　　　　报告时间：2016/1/7,17:05:14
谱图文件:d:\zhuguangjiong\zj\20160106\zj-5-017-oj-100-1-1-214..org

实验内容简介：

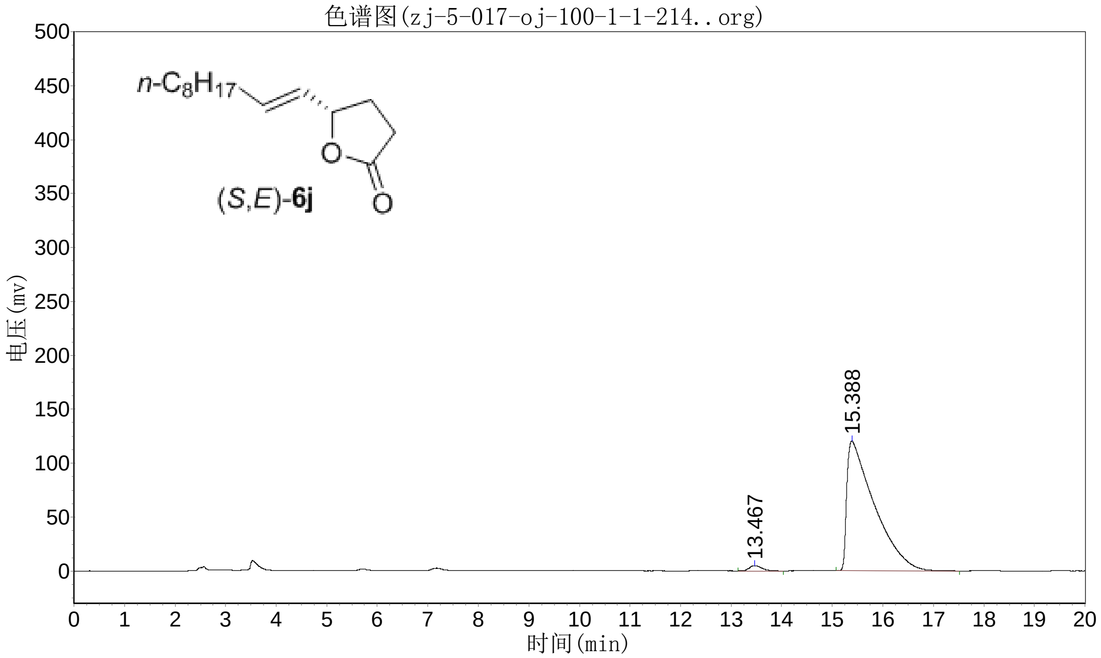

分析结果表

峰号	峰名	保留时间	峰高	峰面积	含量
1		13.467	4963.917	83447.000	1.8000
2		15.388	120397.070	4552410.500	98.2000
总计			125360.987	4635857.500	100.0000

Supplementary Figure 242. HPLC spectrum for (*S*,*E*)-6j.

zj-5-013-oj-h-100-1-1-214

实验时间：2016/1/7,13:38:12　　　　　　　　　　　　报告时间：2016/1/7,17:01:04
谱图文件:d:\zhuguangjiong\zj\20160106\zj-5-013-oj-100-1-1-214.org

实验内容简介：

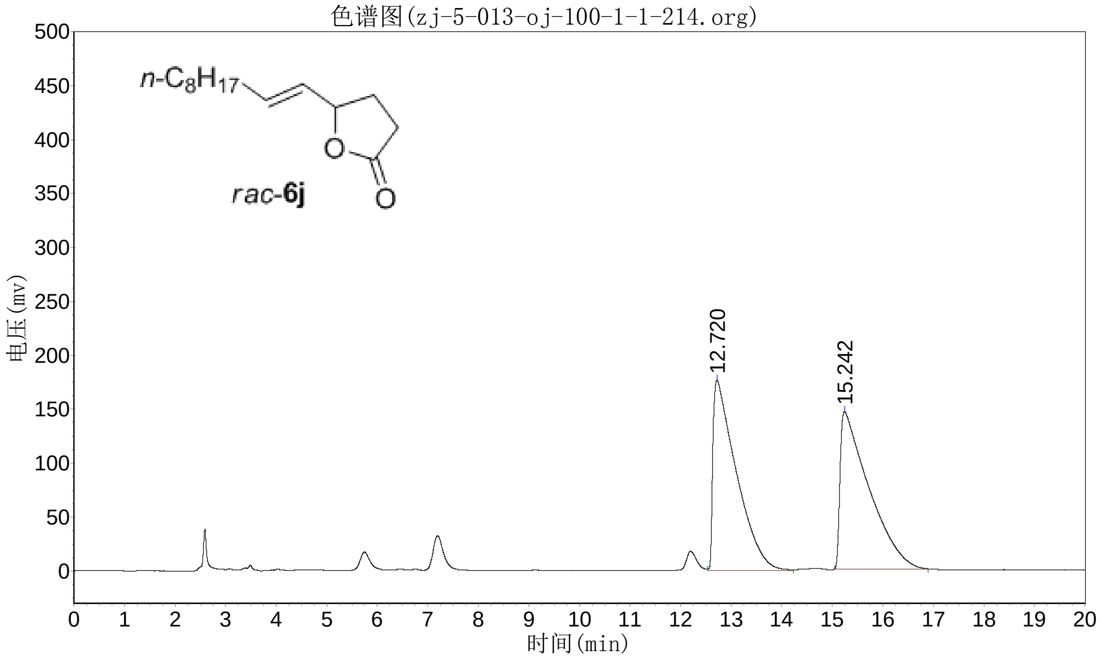

分析结果表

峰号	峰名	保留时间	峰高	峰面积	含量
1		12.720	176572.141	5660659.000	49.5398
2		15.242	146212.047	5765820.000	50.4602
总计			322784.188	11426479.000	100.0000

Supplementary Figure 243. HPLC spectrum for *rac*-6j.

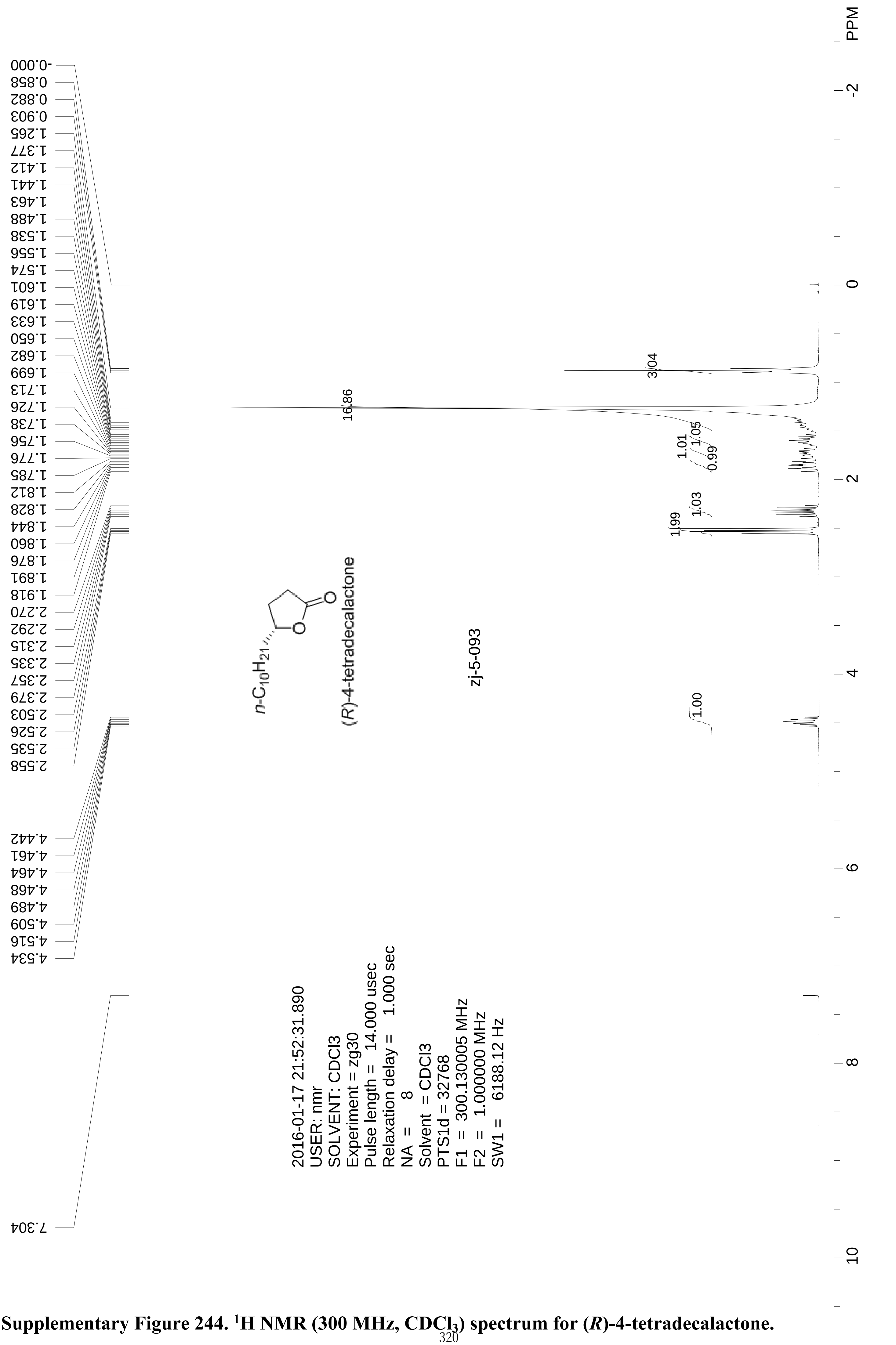

Supplementary Figure 244. ^{1}H NMR (300 MHz, $CDCl_3$) spectrum for (*R*)-4-tetradecalactone.

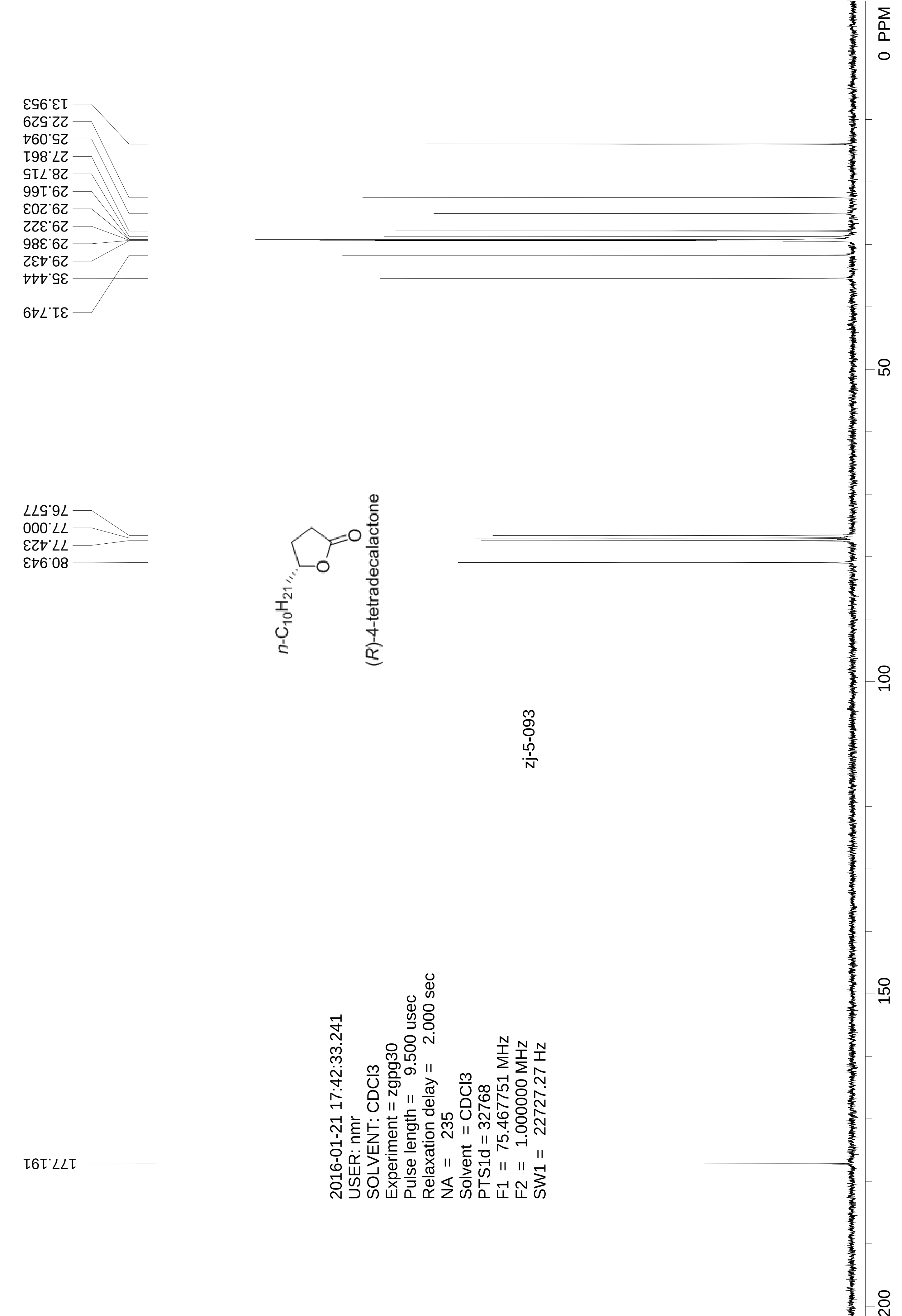

Supplementary Figure 245. ^{13}C **NMR (75 MHz,** $CDCl_3$**) spectrum for (*R*)-4-tetradecalactone.**

数据文件: C:\CHEM32\1\DATA\1\SIG1003988.D
样品名称: zj-5-093

==

操作者 : lxl
仪器 : 仪器 1 位置 : 样品瓶 1
进样日期 : 2016-5-26 15:21:37
进样量 : 手动
采集方法 : C:\CHEM32\1\METHODS\TEST2-1.M
最后修改 : 2016-5-26 15:20:24 : lxl
(调用后修改)
分析方法 : C:\CHEM32\1\METHODS\TEST2-1.M
最后修改 : 2016-5-26 17:33:32 : lxl
(调用后修改)
样品信息 :

附加信息: 峰已手动积分

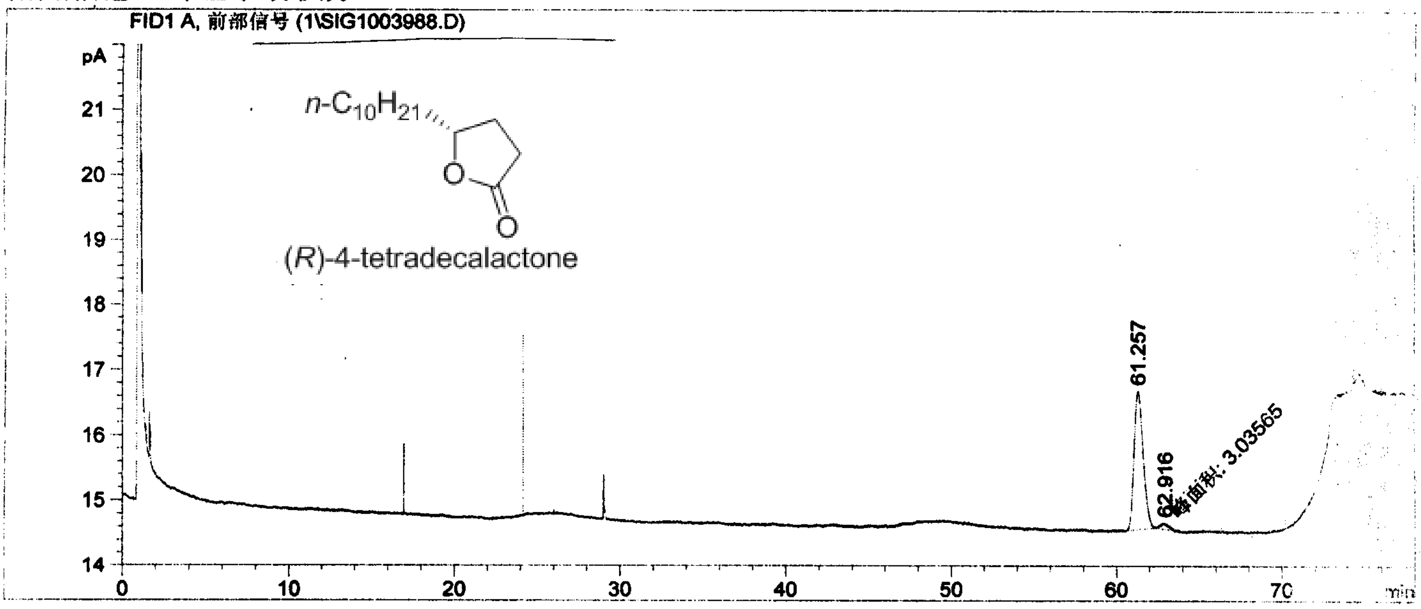

==
面积百分比报告
==

排序 : 信号
乘积因子: : 1.0000
稀释因子: : 1.0000
内标使用乘积因子和稀释因子

信号 1: FID1 A, 前部信号

峰 #	保留时间 [min]	类型	峰宽 [min]	峰面积 [pA*s]	峰高 [pA]	峰面积 %
1	61.257	BB	0.4434	80.26221	2.12646	96.35567
2	62.916	MM	0.5485	3.03565	9.22428e-2	3.64433

总量 : 83.29786 2.21871

==
*** 报告结束 ***

Supplementary Figure 246. GC spectrum for (*R*)-4-tetradecalactone.

仪器 1 2016-5-26 17:33:58 lxl 页 1/1

数据文件: C:\CHEM32\1\DATA\1\SIG1003986.D
样品名称: zj-5-089

===

操作者 : lxl
仪器 : 仪器 1 位置 : 样品瓶 1
进样日期 : 2016-5-26 13:41:28
进样量 : 手动
采集方法 : C:\CHEM32\1\METHODS\TEST2-1.M
最后修改 : 2016-5-26 13:34:17 : lxl
(调用后修改)
分析方法 : C:\CHEM32\1\METHODS\TEST2-1.M
最后修改 : 2016-5-26 15:05:08 : lxl
(调用后修改)
样品信息 :

附加信息: 峰已手动积分

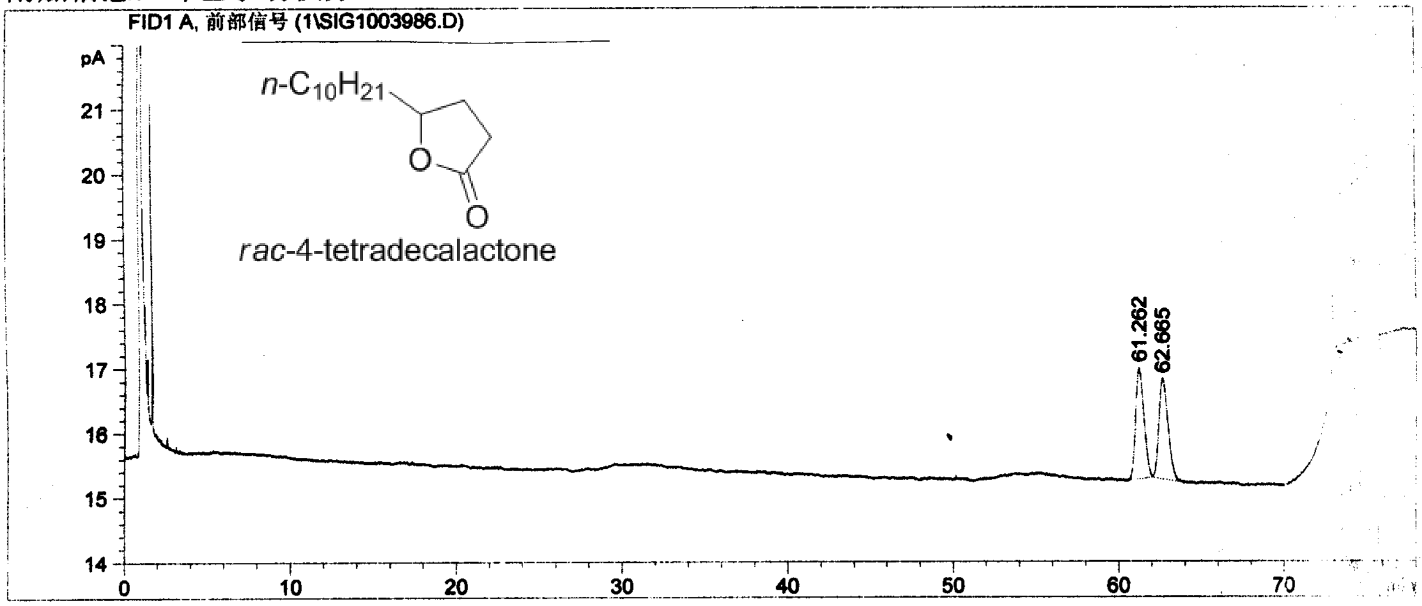

===

面积百分比报告

===

排序 : 信号
乘积因子: : 1.0000
稀释因子: : 1.0000
内标使用乘积因子和稀释因子

信号 1: FID1 A, 前部信号

峰 #	保留时间 [min]	类型	峰宽 [min]	峰面积 [pA*s]	峰高 [pA]	峰面积 %
1	61.262	BB	0.4154	60.14267	1.71822	50.11954
2	62.665	BB	0.4538	59.85578	1.54913	49.88046

总量 : 119.99844 3.26736

===

*** 报告结束 ***

Supplementary Figure 247. GC spectrum for *rac*-4-tetradecalactone.

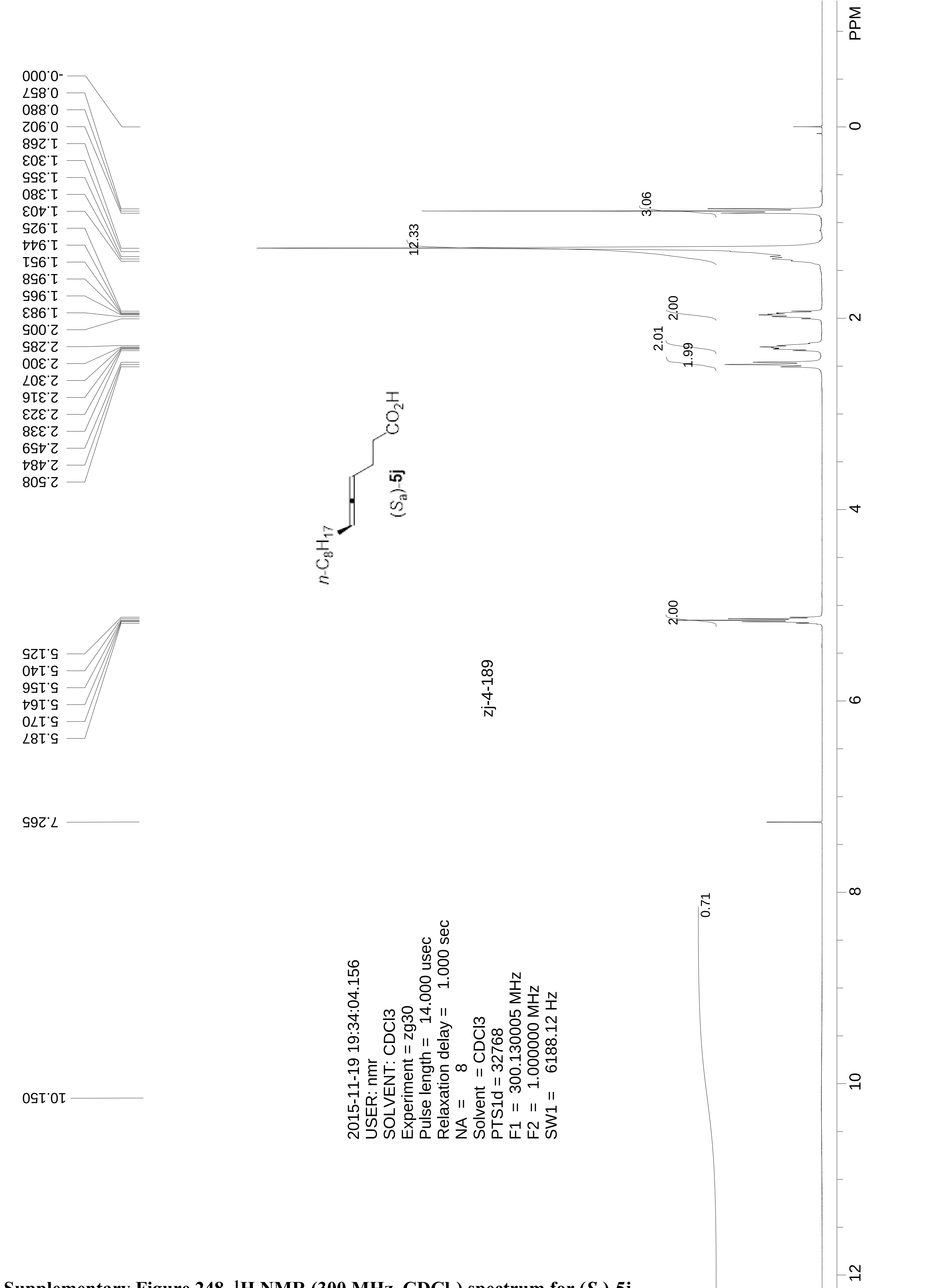

Supplementary Figure 248. ^{1}H NMR (300 MHz, $CDCl_3$) spectrum for (*S*$_a$)-5j.

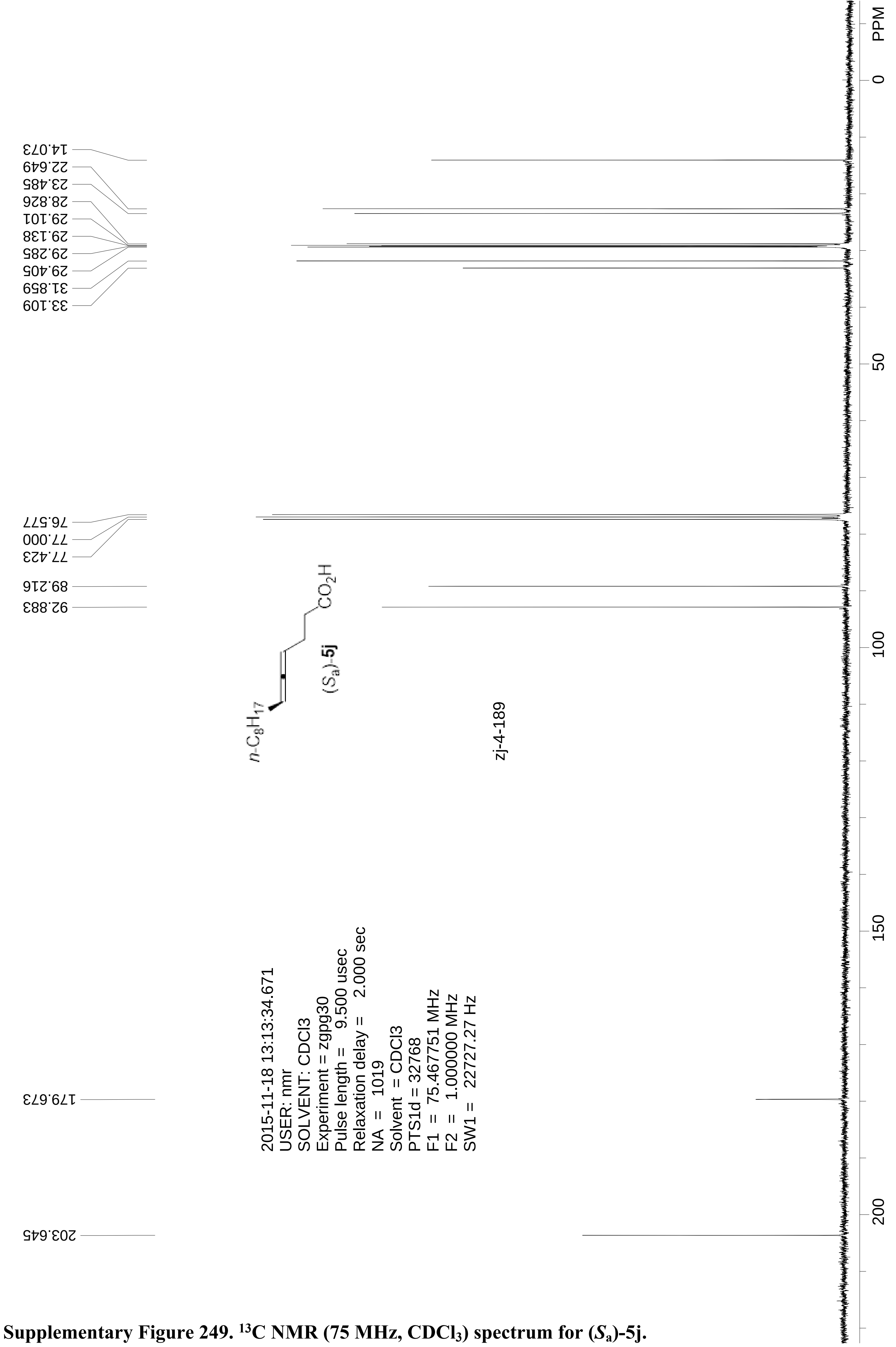

Supplementary Figure 249. ^{13}**C NMR (75 MHz, CDCl$_3$) spectrum for (*S*$_a$)-5j.**

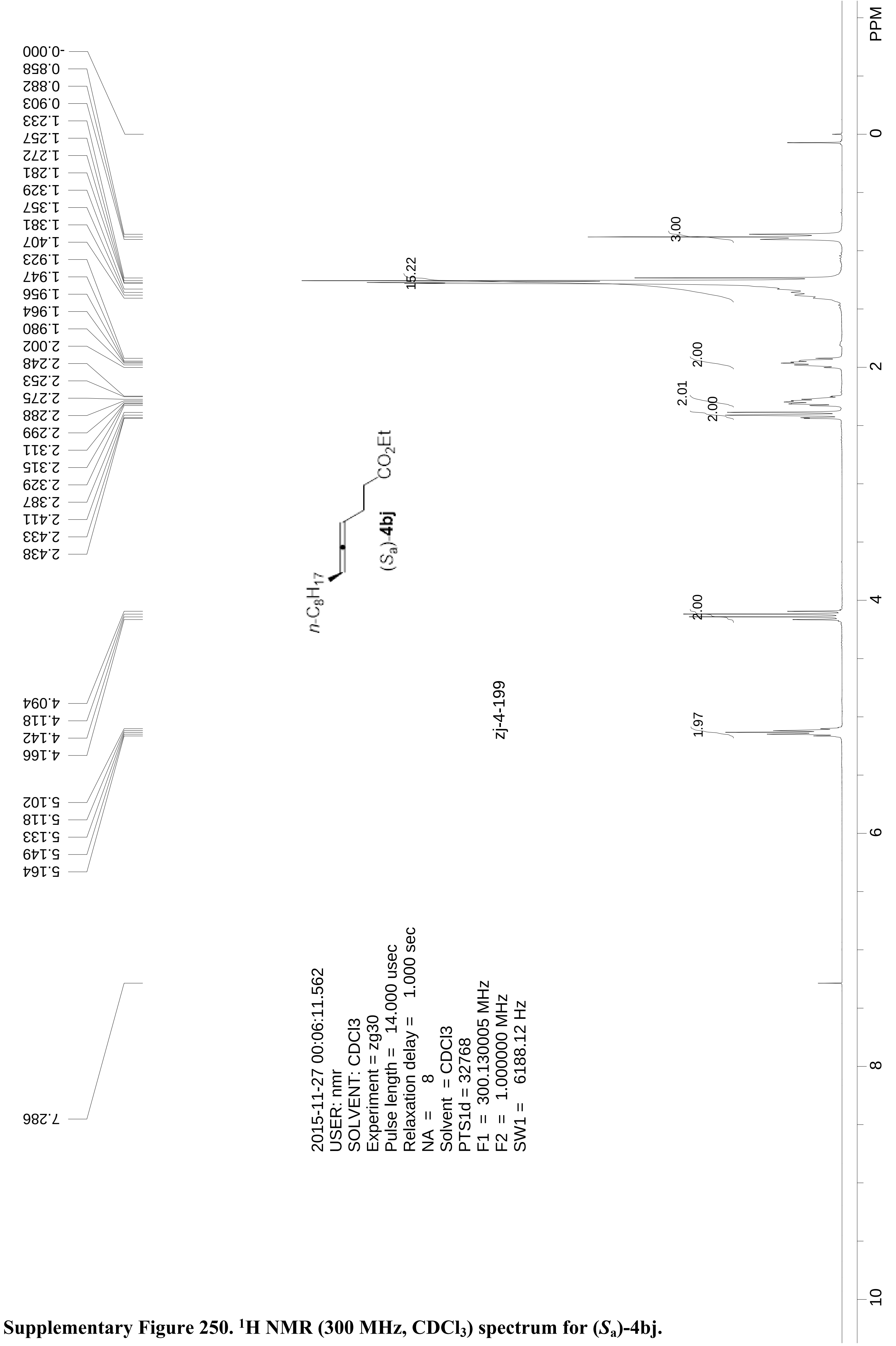

Supplementary Figure 250. ^{1}H NMR (300 MHz, $CDCl_3$) spectrum for (S_a)-4bj.

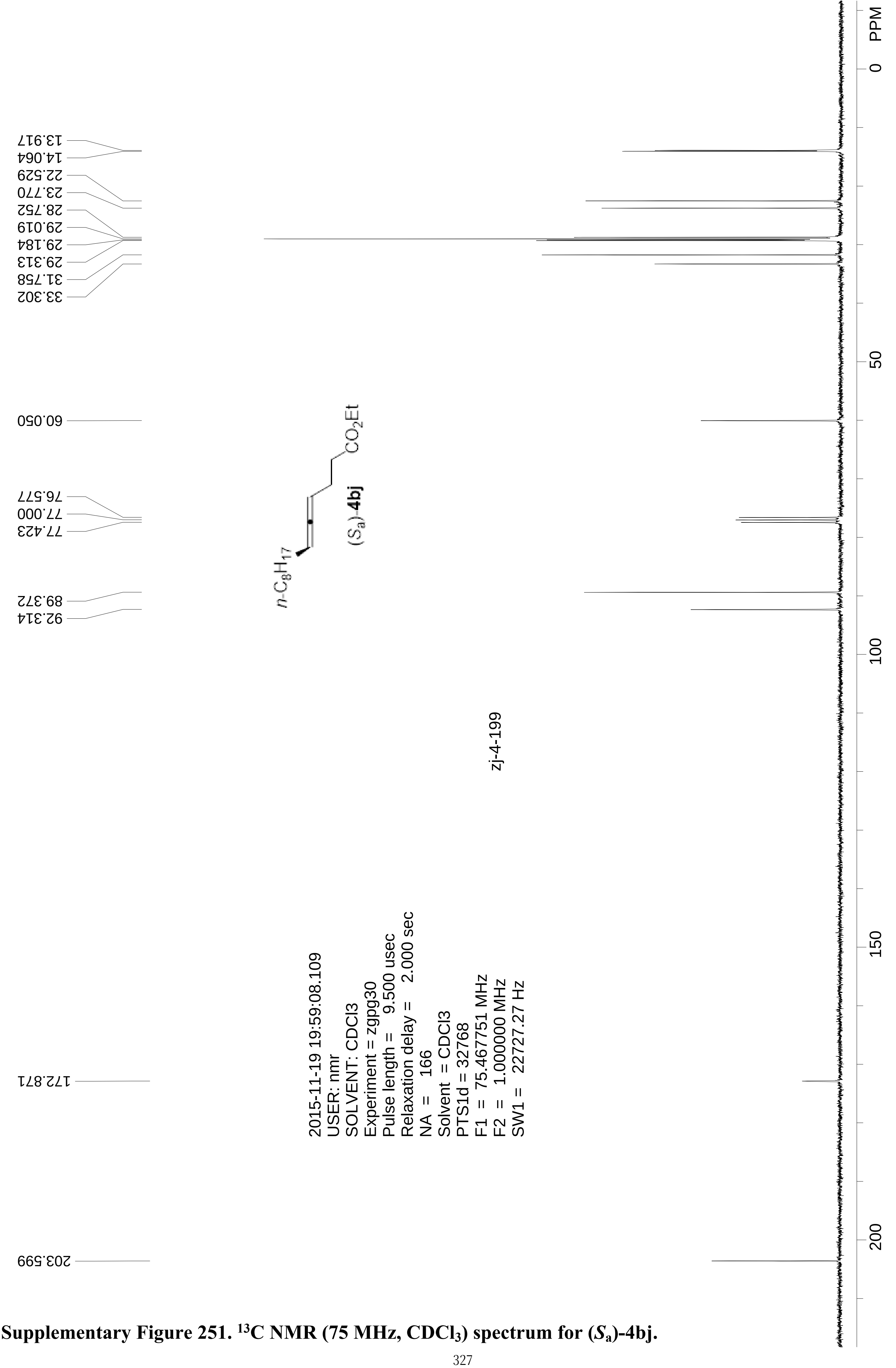

Supplementary Figure 251. ^{13}C NMR (75 MHz, $CDCl_3$) spectrum for (S_a)-4bj.

zj-4-199-oz-h-400-1-0.5-214

实验时间：2015/11/25,9:50:52　　　　　　　　　　报告时间：2015/11/26,18:04:03
谱图文件:D:\zhuguangjiong\zj\20151125\zj-4-199-oz-h-400-1-0.5-214..org

实验内容简介：

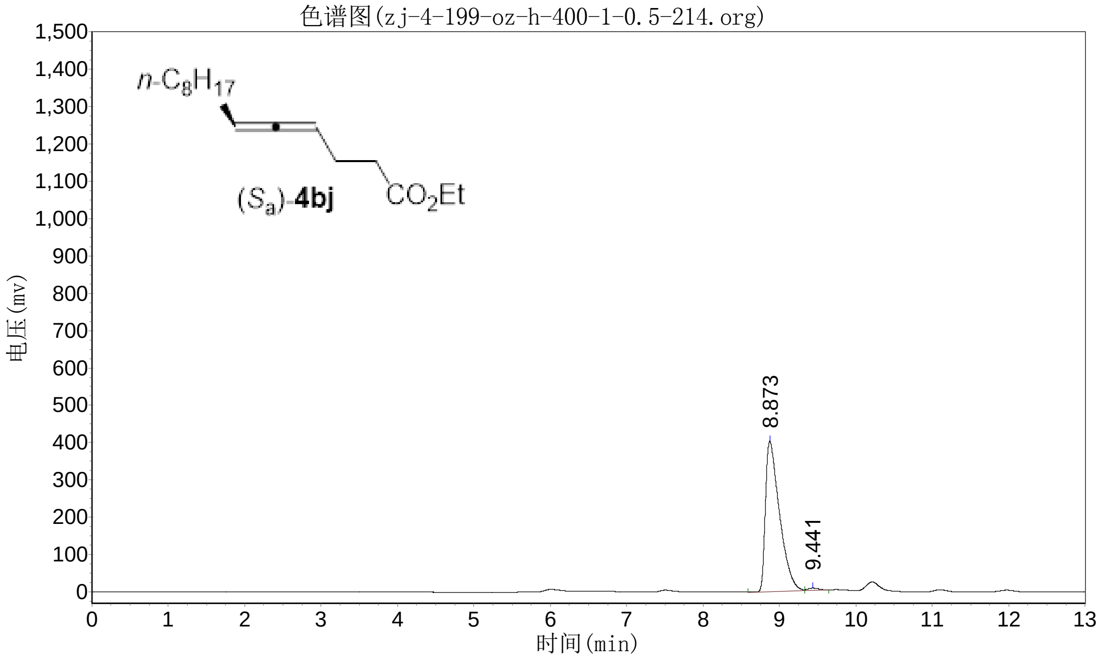

分析结果表

峰号	峰名	保留时间	峰高	峰面积	含量
1		8.873	403421.063	5154801.000	98.7158
2		9.441	6165.251	67059.127	1.2842
总计			409586.303	5219753.844	100.0000

Supplementary Figure 252. HPLC spectrum for (S_a)-4bj.

zj-4-197-oz-h-400-1-0.5-214

实验时间：2015/11/25,9:21:13 报告时间：2015/11/26,17:53:25
谱图文件:d:\zhuguangjiong\zj\20151125\zj-4-197-oz-h-400-1-0.5-214.org

实验内容简介：

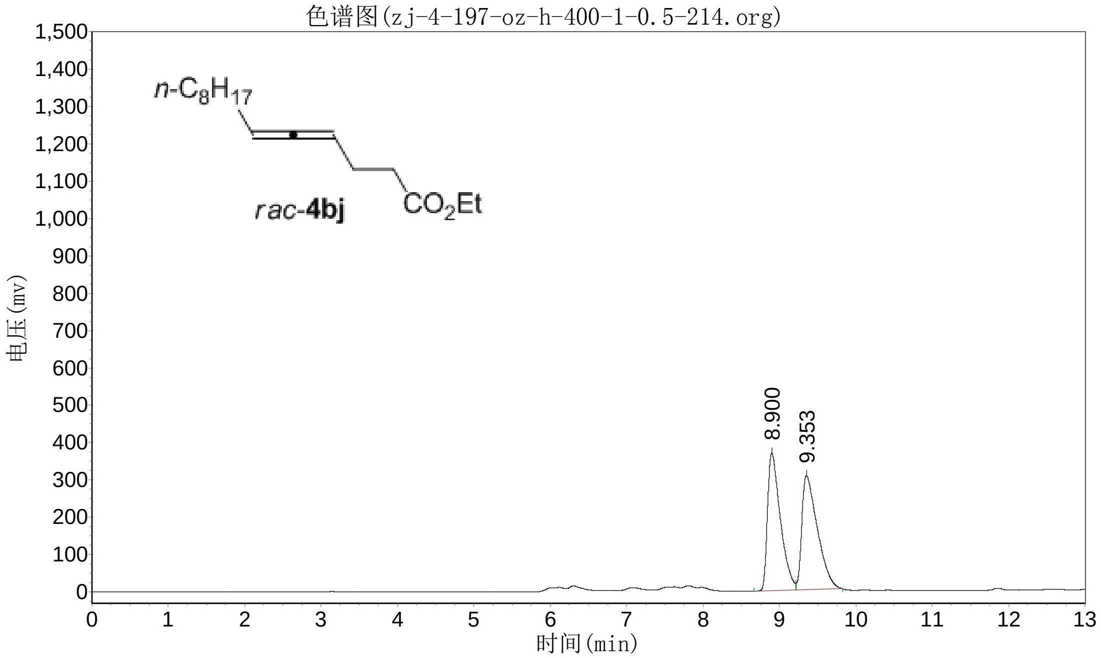

分析结果表

峰号	峰名	保留时间	峰高	峰面积	含量
1		8.900	367626.813	4300627.000	49.8297
2		9.353	305268.219	4330028.000	50.1703
总计			672895.031	8630655.000	100.0000

Supplementary Figure 253. HPLC spectrum for *rac*-4bj.

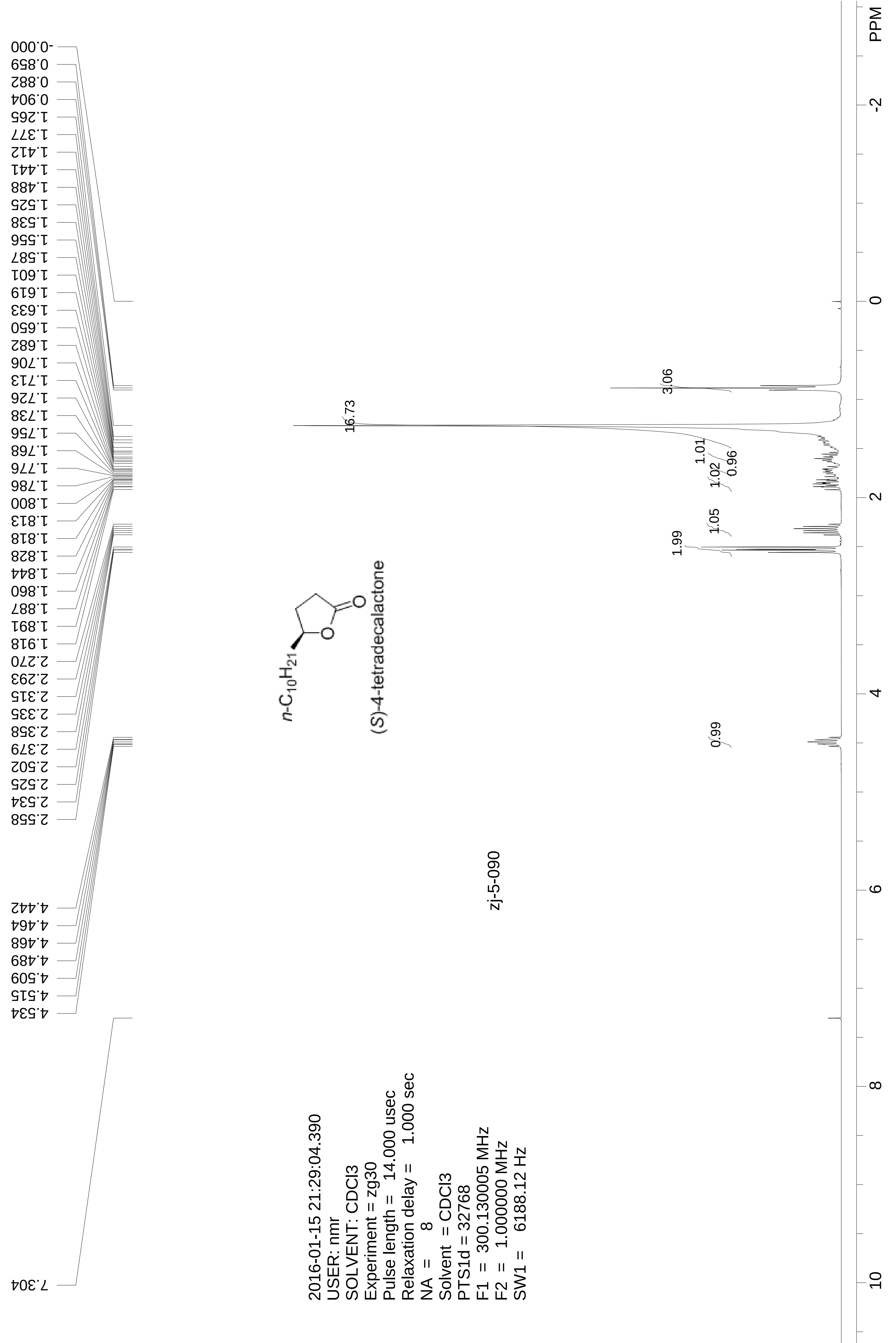

Supplementary Figure 254. **^{1}H NMR (300 MHz, $CDCl_3$) spectrum for (*S*)-4-tetradecalactone.**

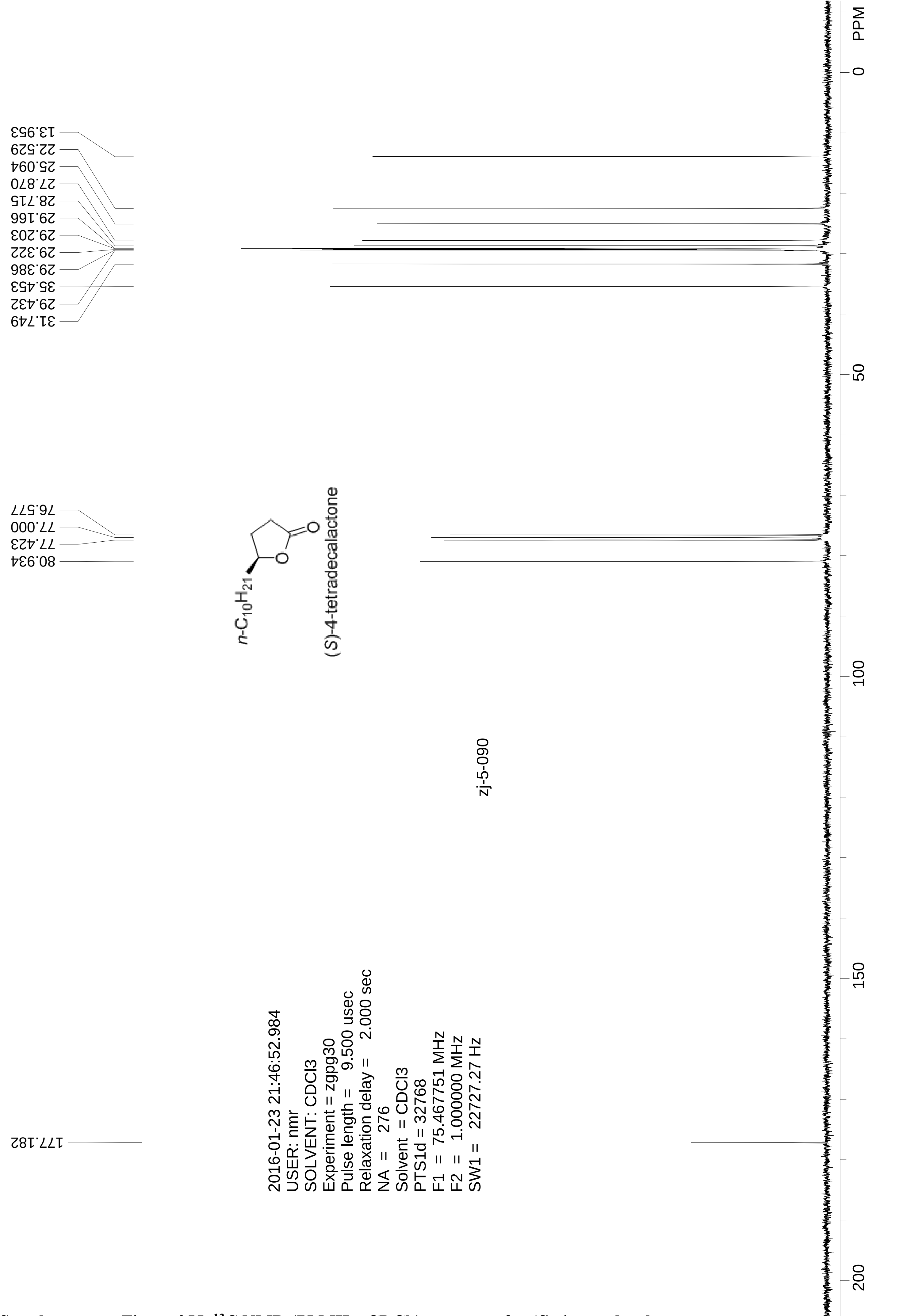

Supplementary Figure 255. ^{13}C **NMR (75 MHz,** $CDCl_3$**) spectrum for (*S*)-4-tetradecalactone.**

数据文件：C:\CHEM32\1\DATA\1\SIG1003990.D
样品名称：zj-5-090

===

操作者 : lxl
仪器 : 仪器 1 位置 : 样品瓶 1
进样日期 : 2016-5-26 17:56:45
进样量 : 手动
采集方法 : C:\CHEM32\1\METHODS\TEST2-1.M
最后修改 : 2016-5-26 18:00:16 : lxl
(调用后修改)
分析方法 : C:\CHEM32\1\METHODS\TEST2-1.M
最后修改 : 2016-5-26 17:33:32 : lxl
(调用后修改)
样品信息 :

附加信息：峰已手动积分

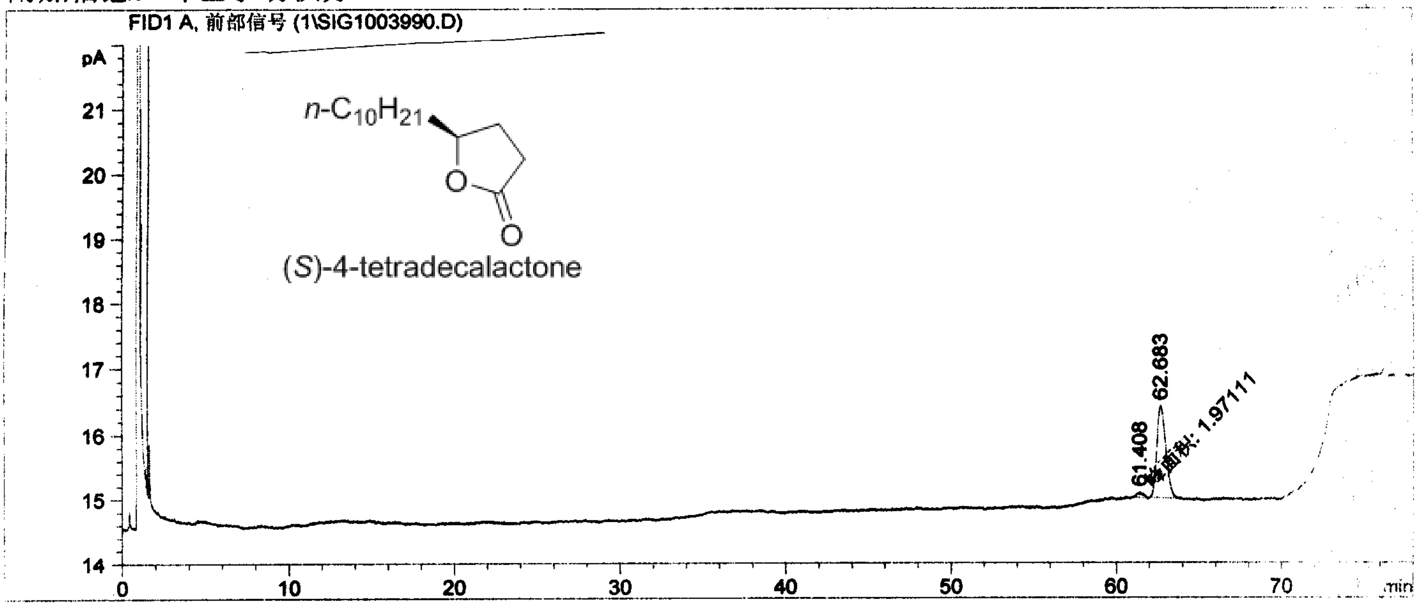

===

面积百分比报告

===

排序 : 信号
乘积因子: : 1.0000
稀释因子: : 1.0000
内标使用乘积因子和稀释因子

信号 1: FID1 A, 前部信号

峰 #	保留时间 [min]	类型	峰宽 [min]	峰面积 [pA*s]	峰高 [pA]	峰面积 %
1	61.408	MM	0.3836	1.97111	8.56391e-2	3.56208
2	62.683	BB	0.4427	53.36497	1.41268	96.43792

总量 : 55.33608 1.49832

===

*** 报告结束 ***

Supplementary Figure 256. GC spectrum for (*S*)-4-tetradecalactone.

数据文件：C:\CHEM32\1\DATA\1\SIG1003986.D
样品名称：zj-5-089

==

操作者	: lxl		
仪器	: 仪器 1	位置	: 样品瓶 1
进样日期	: 2016-5-26 13:41:28		
		进样量	: 手动
采集方法	: C:\CHEM32\1\METHODS\TEST2-1.M		
最后修改	: 2016-5-26 13:34:17 : lxl (调用后修改)		
分析方法	: C:\CHEM32\1\METHODS\TEST2-1.M		
最后修改	: 2016-5-26 15:05:08 : lxl (调用后修改)		
样品信息	:		

附加信息：峰已手动积分

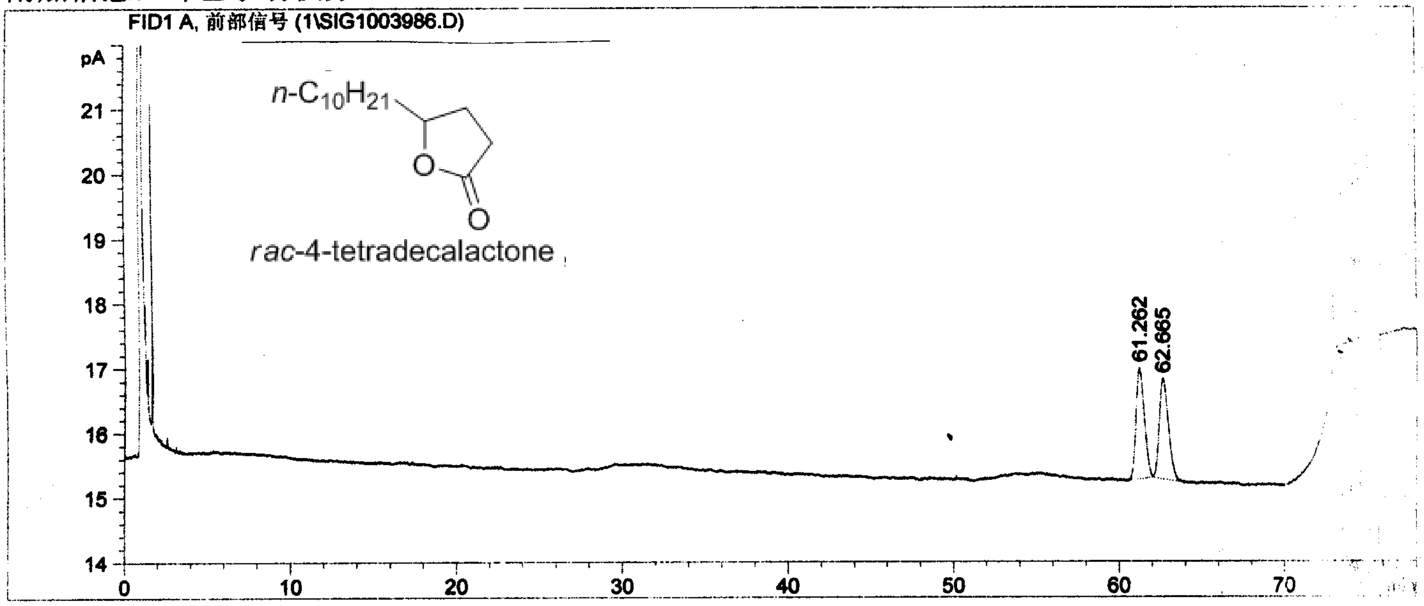

==

面积百分比报告

==

排序 : 信号
乘积因子: : 1.0000
稀释因子: : 1.0000
内标使用乘积因子和稀释因子

信号 1: FID1 A, 前部信号

峰 #	保留时间 [min]	类型	峰宽 [min]	峰面积 [pA*s]	峰高 [pA]	峰面积 %
1	61.262	BB	0.4154	60.14267	1.71822	50.11954
2	62.665	BB	0.4538	59.85578	1.54913	49.88046

总量 : 119.99844 3.26736

==

*** 报告结束 ***

Supplementary Figure 257. GC spectrum for *rac*-4-tetradecalactone.

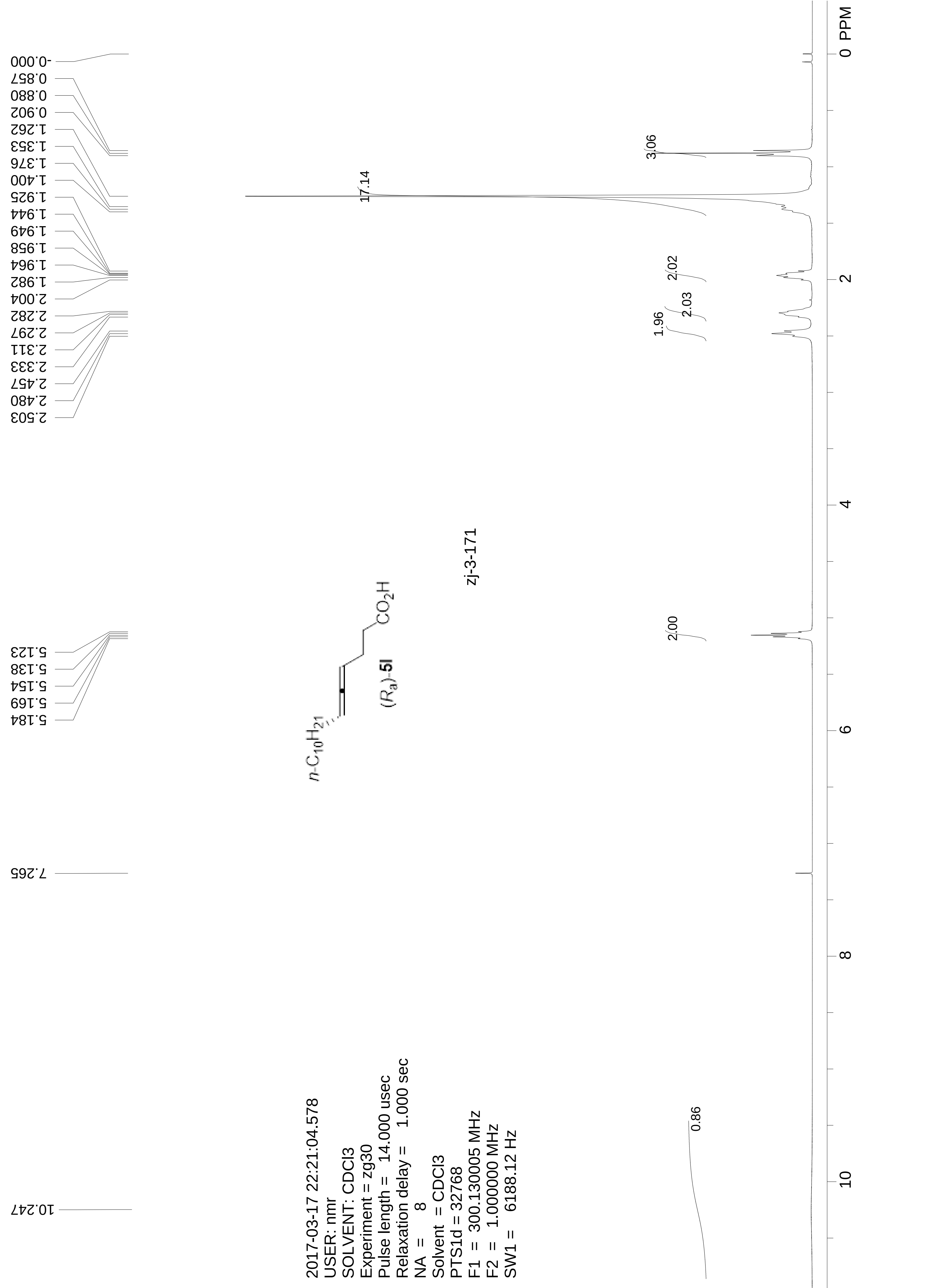

Supplementary Figure 258. ^{1}H NMR (300 MHz, $CDCl_3$) spectrum for (R_a)-5l.

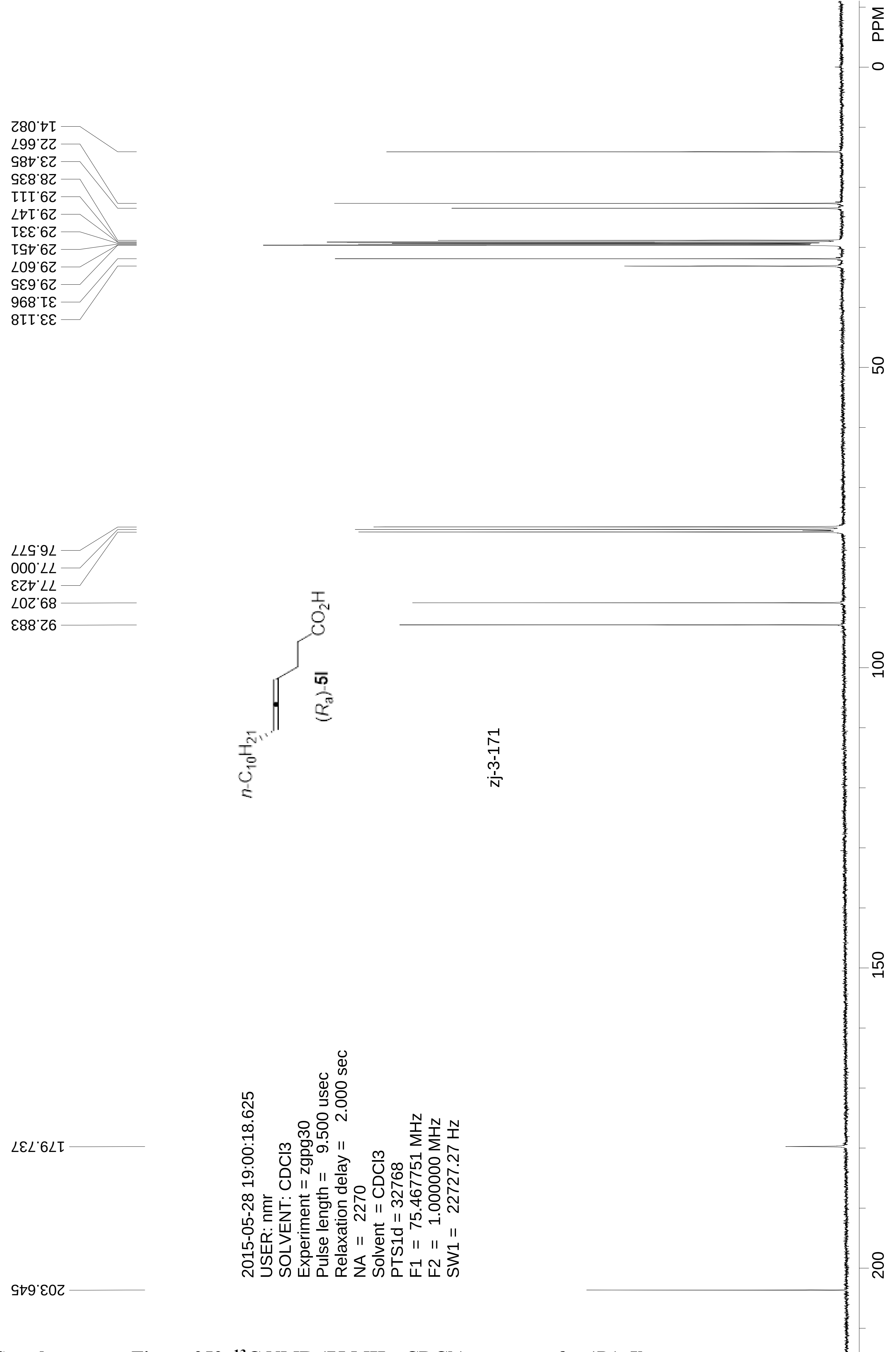

Supplementary Figure 259. ^{13}**C NMR (75 MHz, CDCl$_3$) spectrum for (*R*$_a$)-5l.**

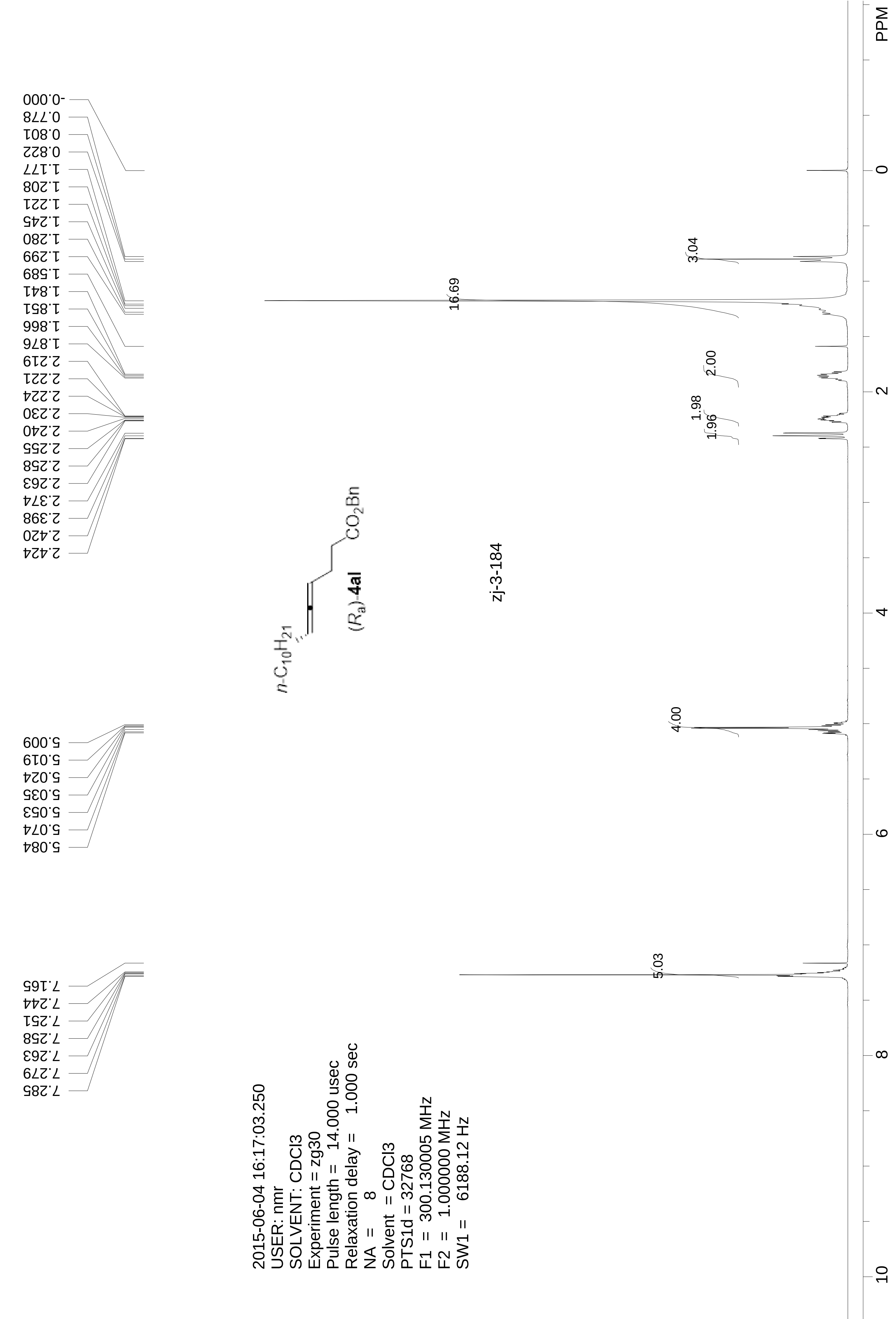

Supplementary Figure 260. ^{1}H NMR (300 MHz, $CDCl_3$) spectrum for (R_a)-4al.

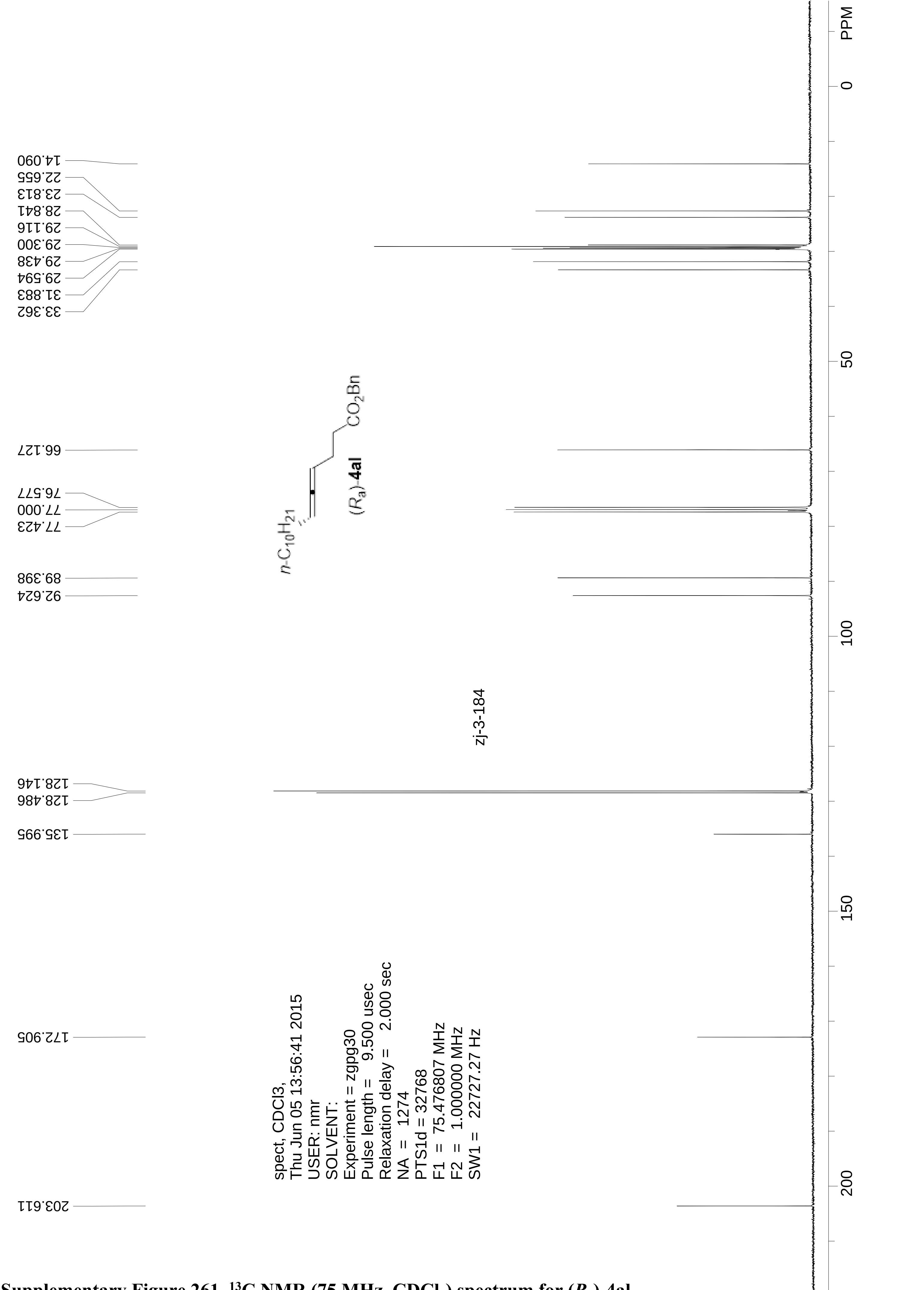

Supplementary Figure 261. ^{13}C NMR (75 MHz, CDCl$_3$) spectrum for (R_a)-4al.

zj-3-184-ia-100-0-0.8-214

实验时间：2015/6/16,11:25:47　　　　报告时间：2015/6/18,17:15:45
谱图文件:D:\zhuguangjiong\zj\20150612\zj-3-184-ia-100-0-0.8-214.org

实验内容简介：

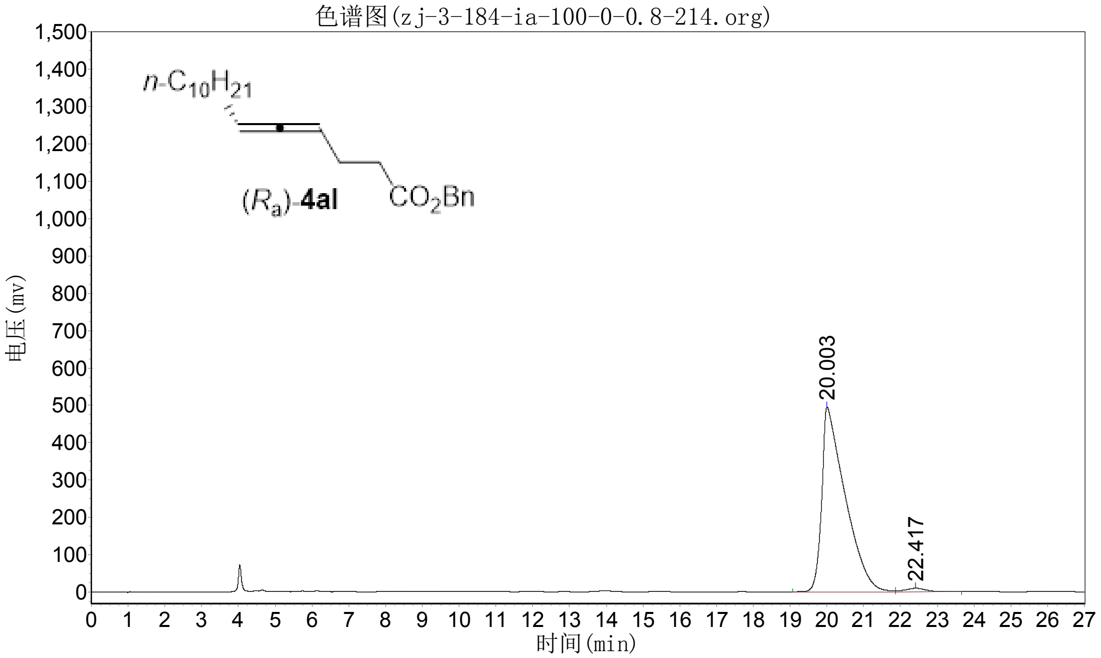

分析结果表

峰号	峰名	保留时间	峰高	峰面积	含量
1		20.003	494230.219	21830514.000	98.3777
2		22.417	9328.662	359993.031	1.6223
总计			503558.881	22190507.031	100.0000

Supplementary Figure 262. HPLC spectrum for (R_a)-4al.

zj-3-183-ia-100-0-0.8-214

实验时间：2015/6/16,10:34:25　　　　报告时间：2015/6/18,17:14:18
谱图文件:D:\zhuguangjiong\zj\20150612\zj-3-183-ia-100-0-0.8-214.org

实验内容简介：

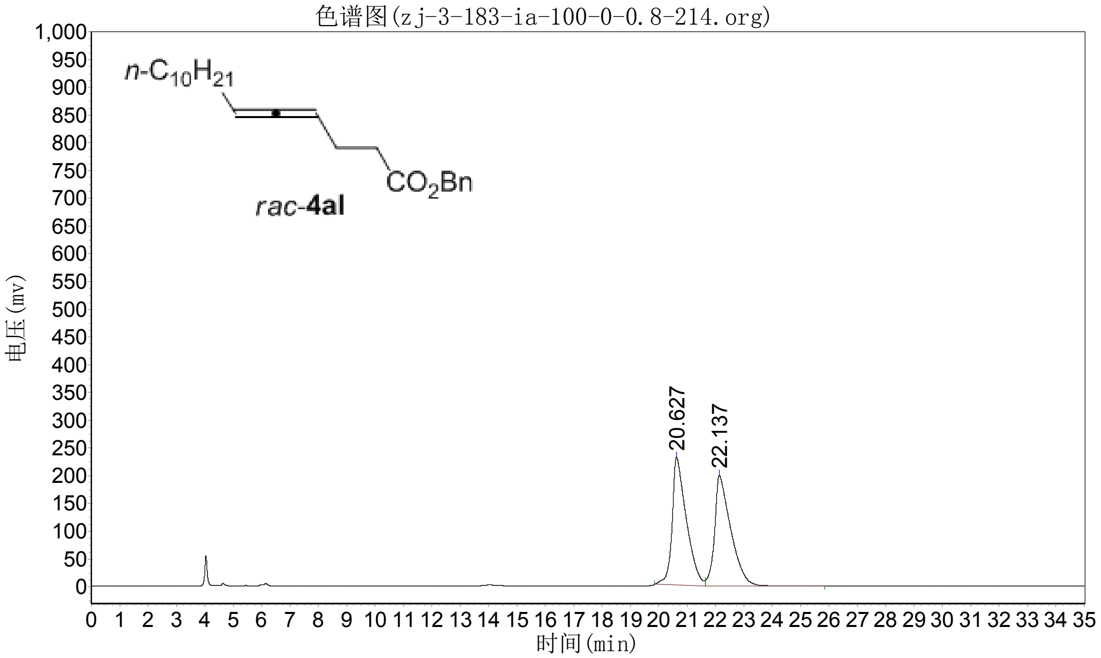

分析结果表

峰号	峰名	保留时间	峰高	峰面积	含量
1		20.627	230837.906	8070378.500	50.4257
2		22.137	199542.828	7934117.000	49.5743
总计			430380.734	16004495.500	100.0000

Supplementary Figure 263. HPLC spectrum for *rac*-4al.

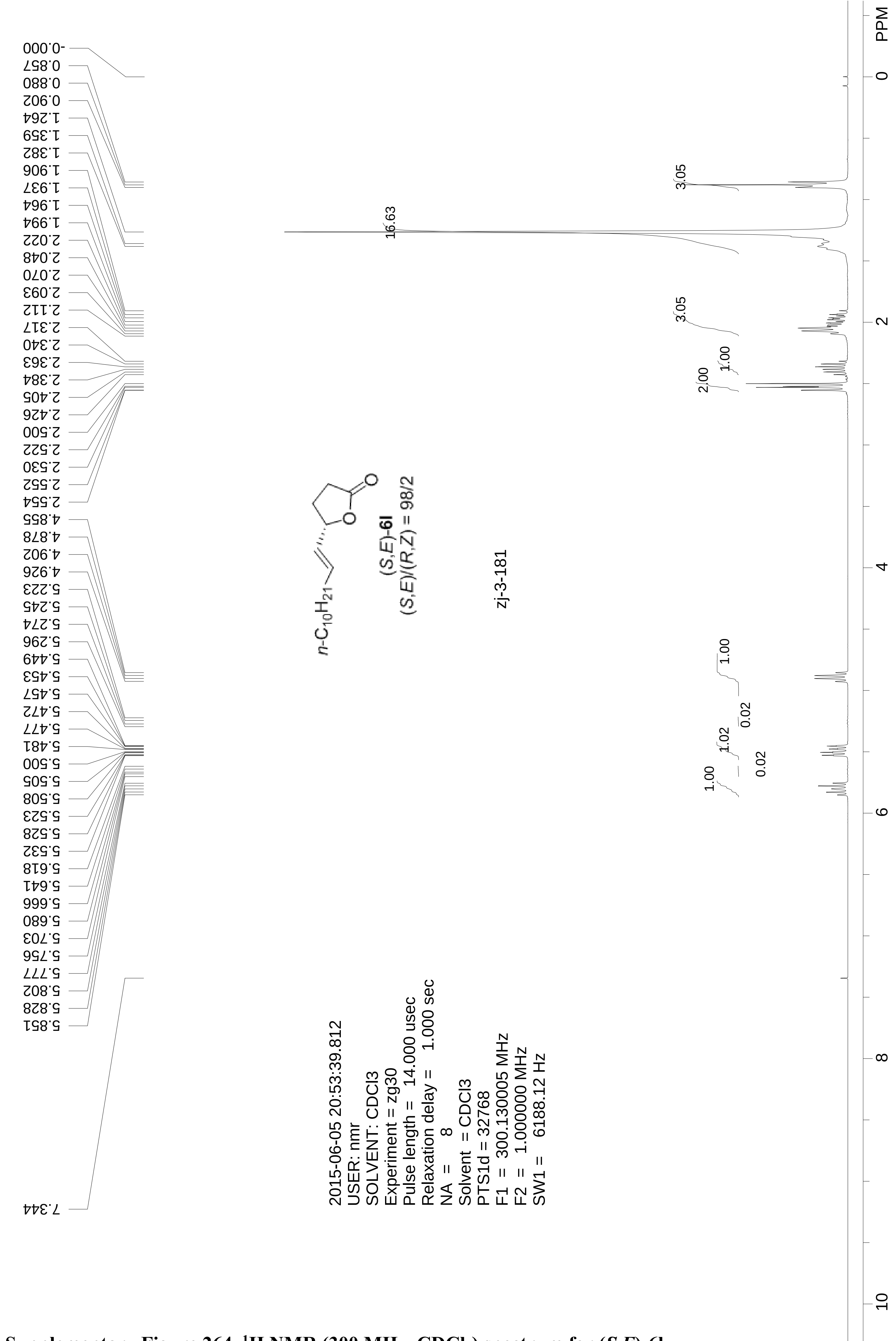

Supplementary Figure 264. ^{1}H NMR (300 MHz, $CDCl_3$) spectrum for (*S*,*E*)-6l.

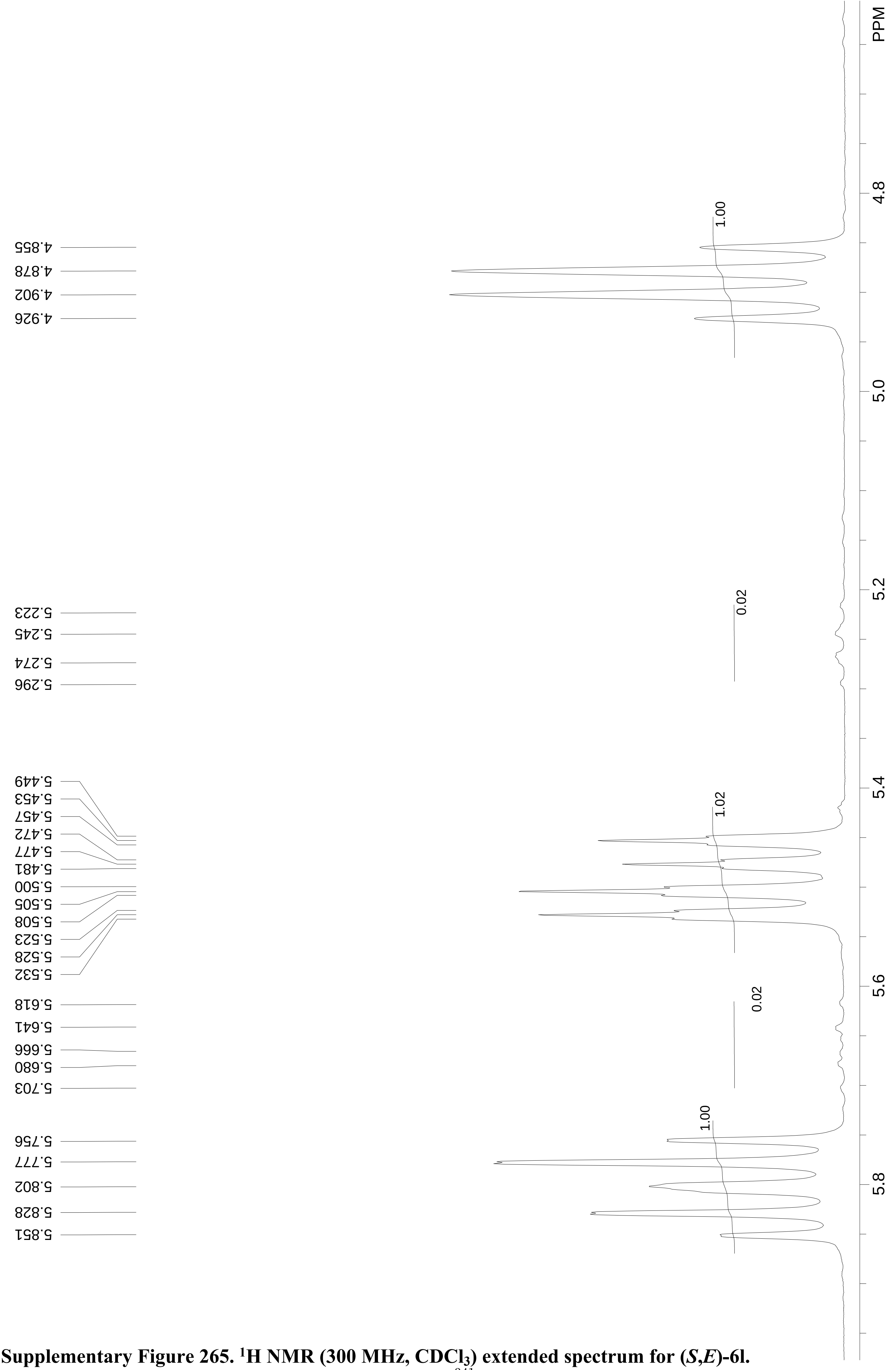

Supplementary Figure 265. ^{1}H NMR (300 MHz, $CDCl_3$) extended spectrum for (*S*,*E*)-6l.

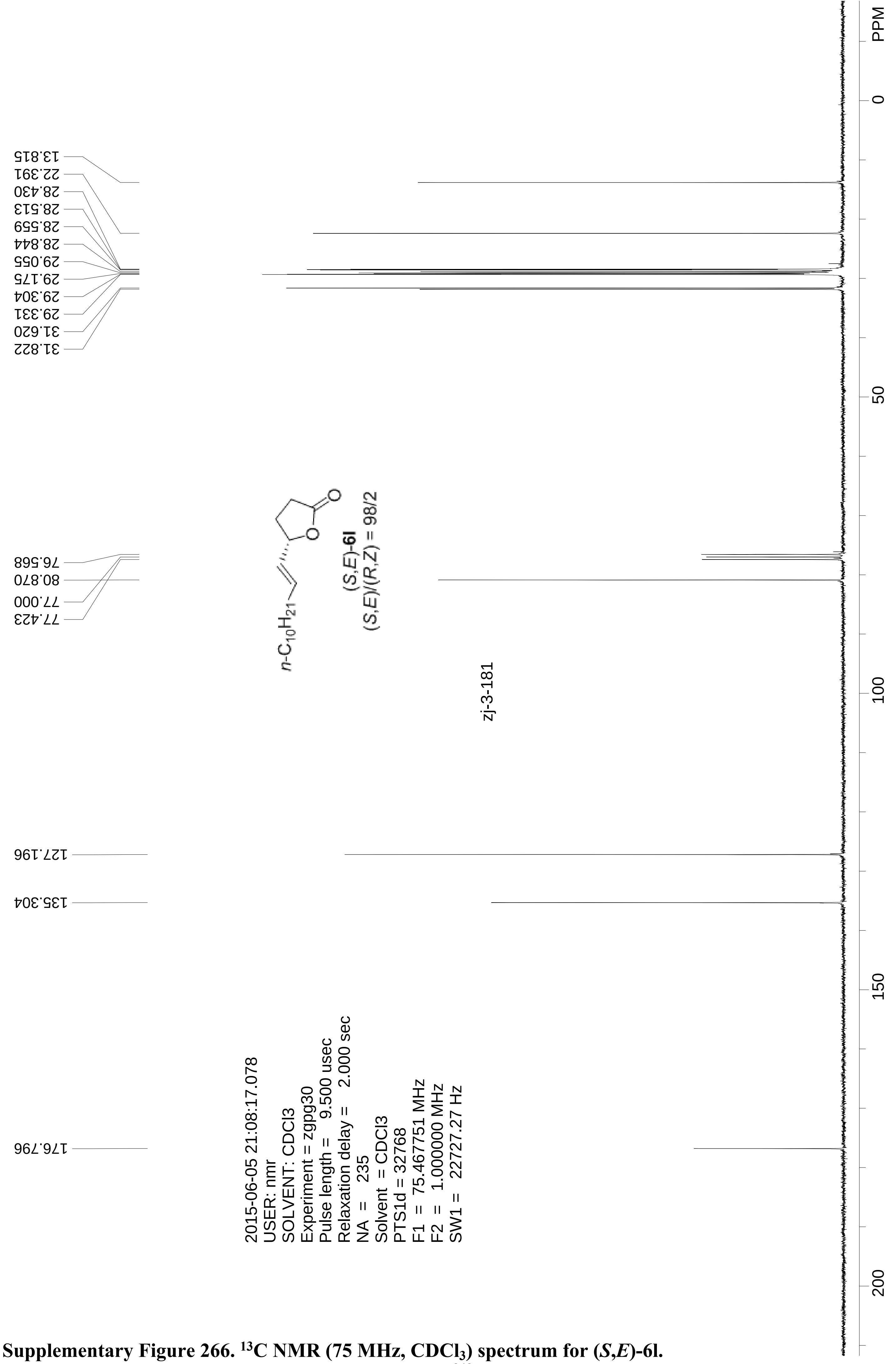

Supplementary Figure 266. ^{13}**C NMR (75 MHz,** $CDCl_3$**) spectrum for (*S*,*E*)-6l.**

zj-3-181-as-h-95-5-1-214

实验时间：2015-07-21,17:15:31　　　　报告时间：2015-07-21,17:39:04
谱图文件:D:\zhuguangjiong\zj\20150720\zj-3-181-as-h-95-5-1-214..org

实验内容简介：

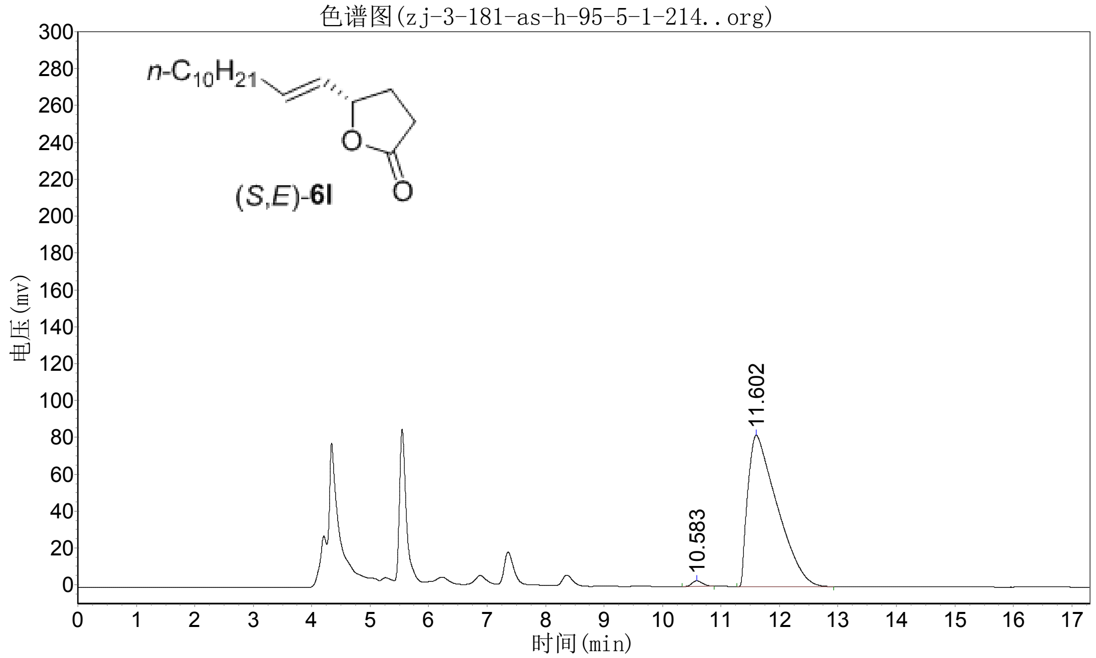

分析结果表

峰号	峰名	保留时间	峰高	峰面积	含量
1		10.583	3032.685	37218.348	1.2799
2		11.602	82252.625	2870599.500	98.7201
总计			85285.310	2907817.848	100.0000

Supplementary Figure 267. HPLC spectrum for (*S*,*E*)-6l.

zj-4-048-as-h-95-5-1-214

实验时间：2015-07-21,15:45:11　　　　　　　　　　报告时间：2015-07-21,17:37:21
谱图文件:d:\zhuguangjiong\zj\20150720\zj-4-048-as-h-95-5-1-214.org

实验内容简介：

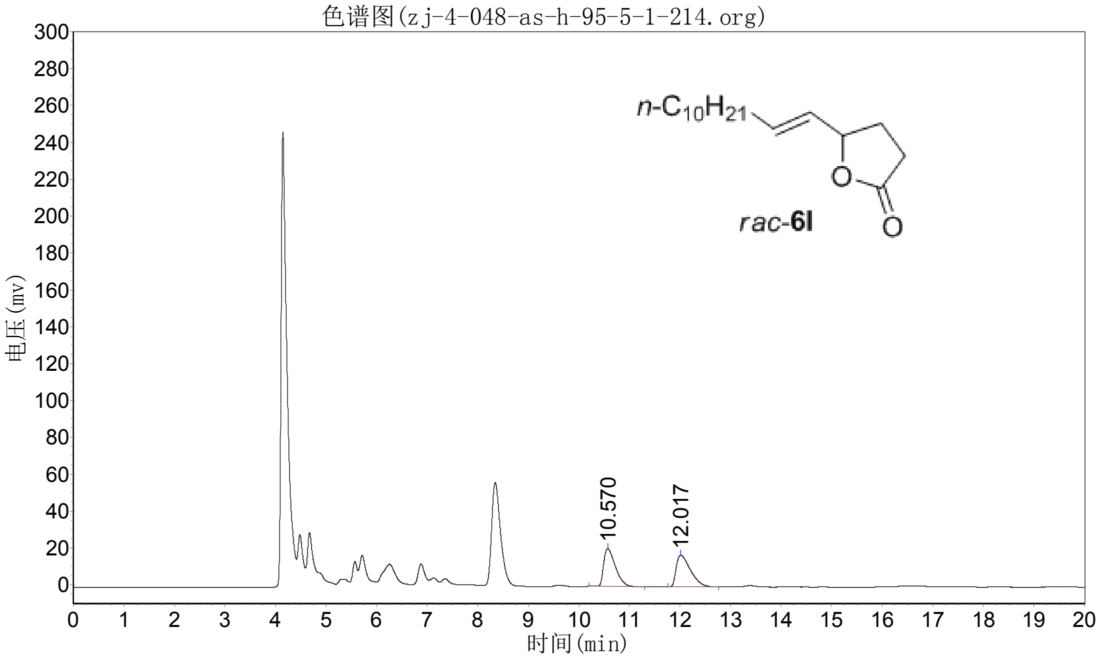

分析结果表

峰号	峰名	保留时间	峰高	峰面积	含量
1		10.570	20498.037	330317.313	49.5101
2		12.017	17177.754	336853.625	50.4899
总计			37675.791	667170.938	100.0000

Supplementary Figure 268. HPLC spectrum for *rac*-6l.

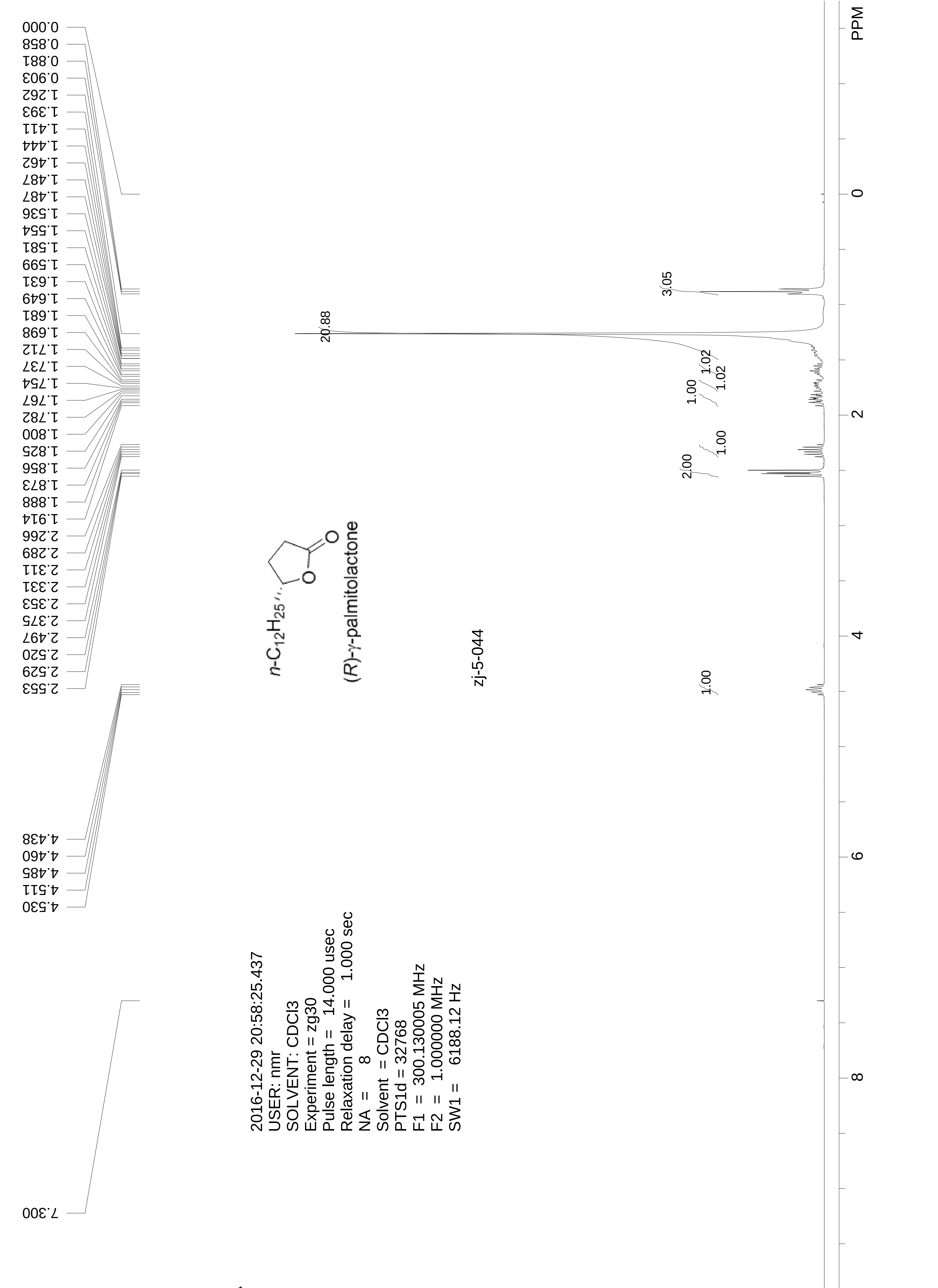

Supplementary Figure 269. ^{1}H NMR (300 MHz, $CDCl_3$) spectrum for (*R*)-γ-palmitolactone.

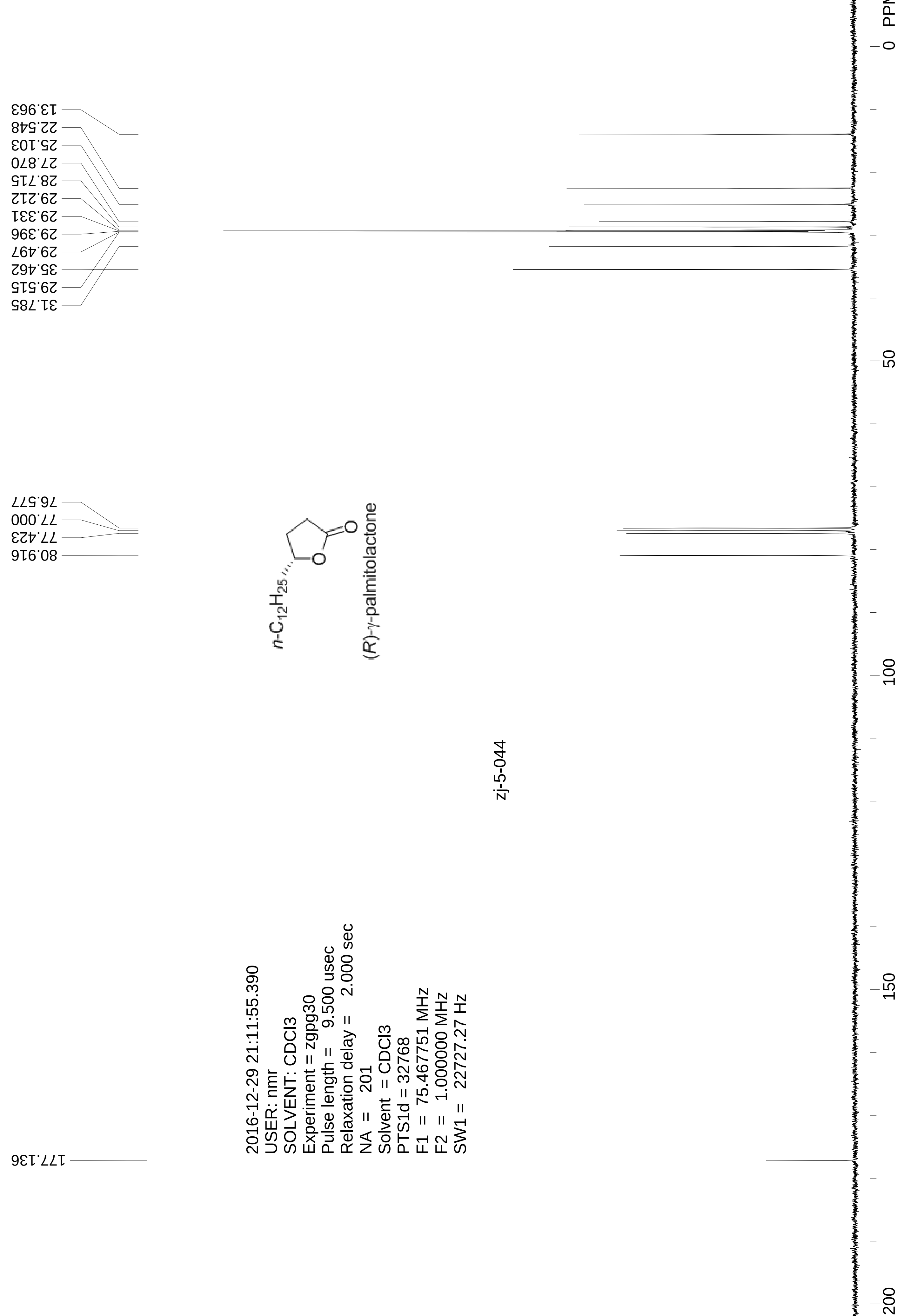

Supplementary Figure 270. ^{13}C NMR (75 MHz, $CDCl_3$) spectrum for (*R*)-γ-palmitolactone.

数据文件: C:\CHEM32\1\DATA\1\SIG1003994.D
样品名称: zj-5-044

===

操作者 : lxl
仪器 : 仪器 1 位置 : 样品瓶 1
进样日期 : 2016-5-27 14:48:11
进样量 : 手动
采集方法 : C:\CHEM32\1\METHODS\TEST2-1.M
最后修改 : 2016-5-27 14:46:45 : lxl
(调用后修改)
分析方法 : C:\CHEM32\1\METHODS\TEST2-1.M
最后修改 : 2016-5-27 17:12:22 : lxl
(调用后修改)
样品信息 :

附加信息: 峰已手动积分

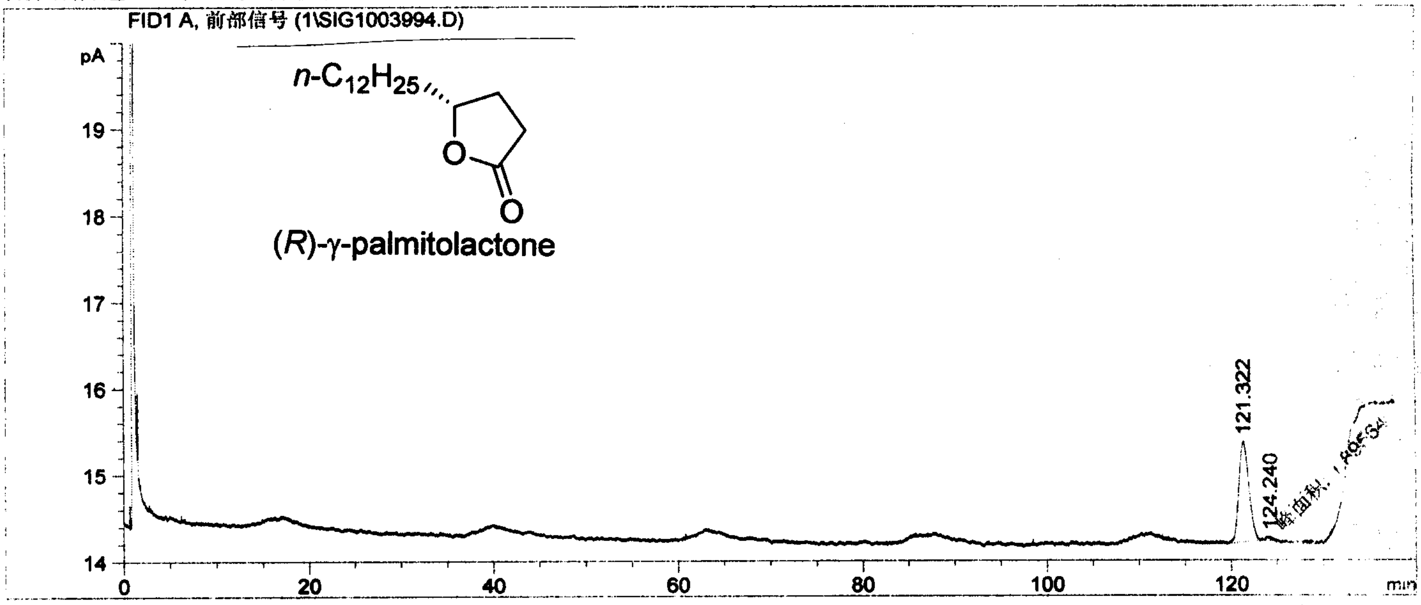

===

面积百分比报告

===

排序 : 信号
乘积因子: : 1.0000
稀释因子: : 1.0000
内标使用乘积因子和稀释因子

信号 1: FID1 A, 前部信号

峰 #	保留时间 [min]	类型	峰宽 [min]	峰面积 [pA*s]	峰高 [pA]	峰面积 %
1	121.322	BB	0.9040	87.18728	1.15586	97.87206
2	124.240	MM	0.6184	1.89564	5.10902e-2	2.12794

总量 : 89.08291 1.20695

===

*** 报告结束 ***

Supplementary Figure 271. GC spectrum for (*R*)-γ-palmitolactone.

数据文件：C:\CHEM32\1\DATA\1\SIG1003992.D
样品名称：zj-5-043

==

操作者	: lxl		
仪器	: 仪器 1	位置	: 样品瓶 1
进样日期	: 2016-5-27 11:19:37		
		进样量	: 手动
采集方法	: C:\CHEM32\1\METHODS\TEST2-1.M		
最后修改	: 2016-5-27 11:18:07 : lxl<br>(调用后修改)		
分析方法	: C:\CHEM32\1\METHODS\TEST2-1.M		
最后修改	: 2016-5-27 14:21:13 : lxl<br>(调用后修改)		
样品信息	:		

附加信息：峰已手动积分

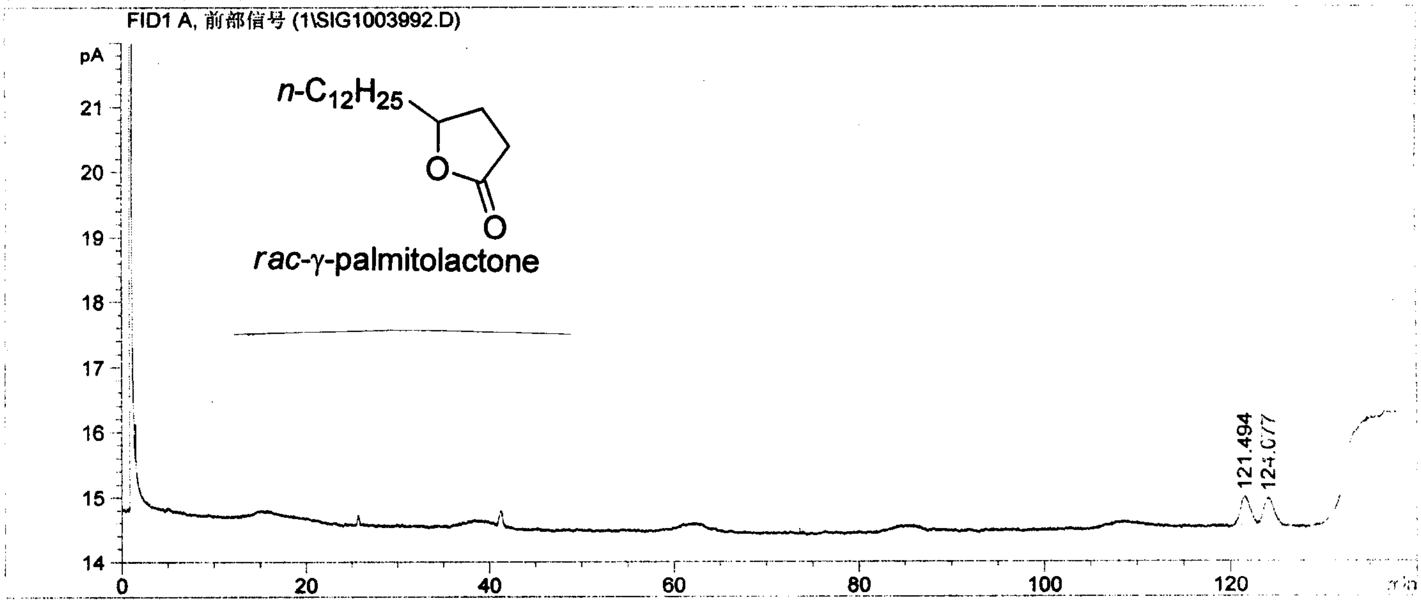

==

面积百分比报告

==

排序 : 信号
乘积因子: : 1.0000
稀释因子: : 1.0000
内标使用乘积因子和稀释因子

信号 1: FID1 A, 前部信号

峰 #	保留时间 [min]	类型	峰宽 [min]	峰面积 [pA*s]	峰高 [pA]	峰面积 %
1	121.494	BB	0.8626	31.67530	4.44895e-1	49.84436
2	124.077	BB	0.9188	31.87311	4.13741e-1	50.15564

总量 : 63.54841 8.58636e-1

==

*** 报告结束 ***

Supplementary Figure 272. GC spectrum for *rac*-γ-palmitolactone.

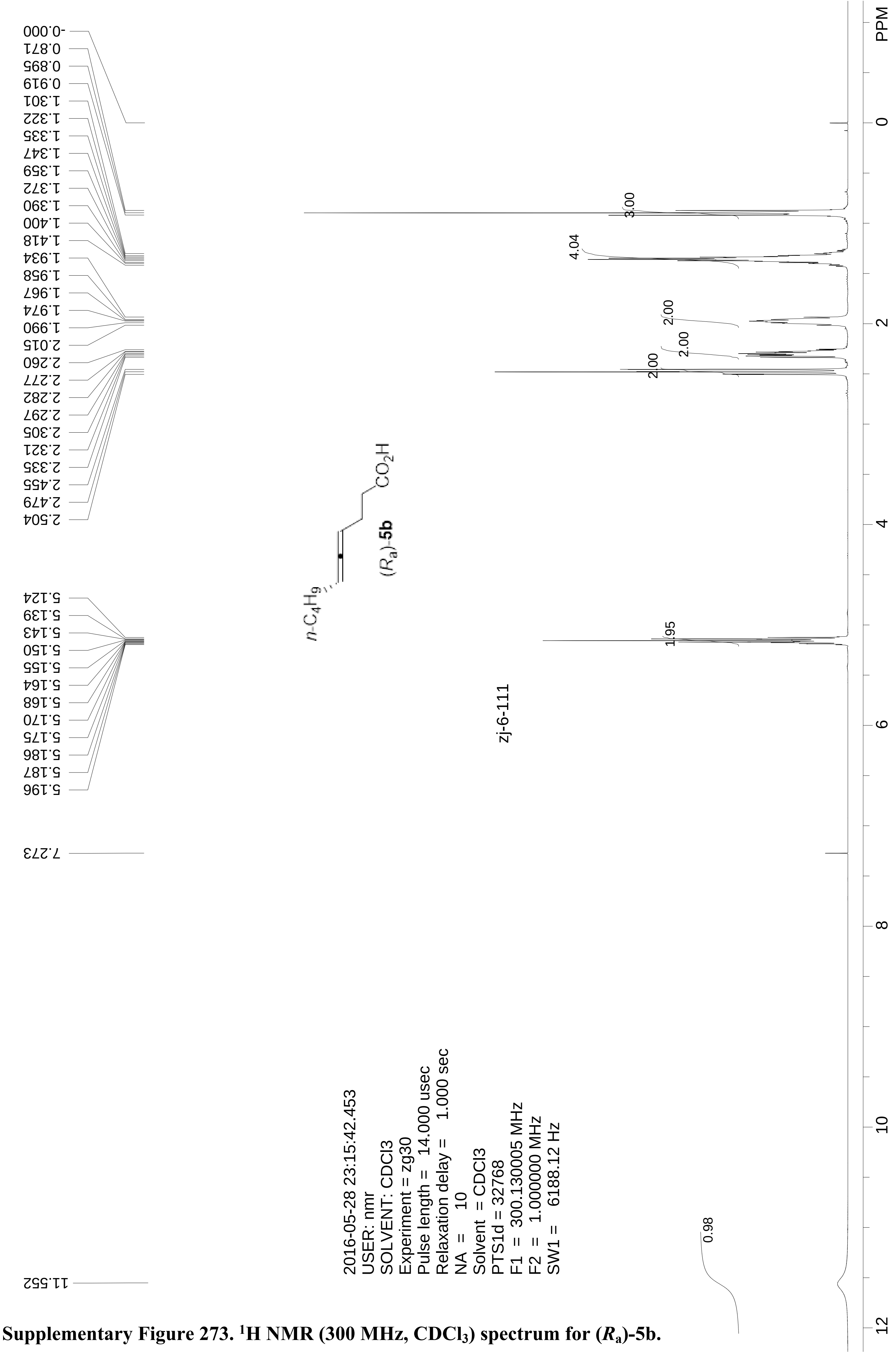

Supplementary Figure 273. ^{1}H NMR (300 MHz, $CDCl_3$) spectrum for (R_a)-5b.

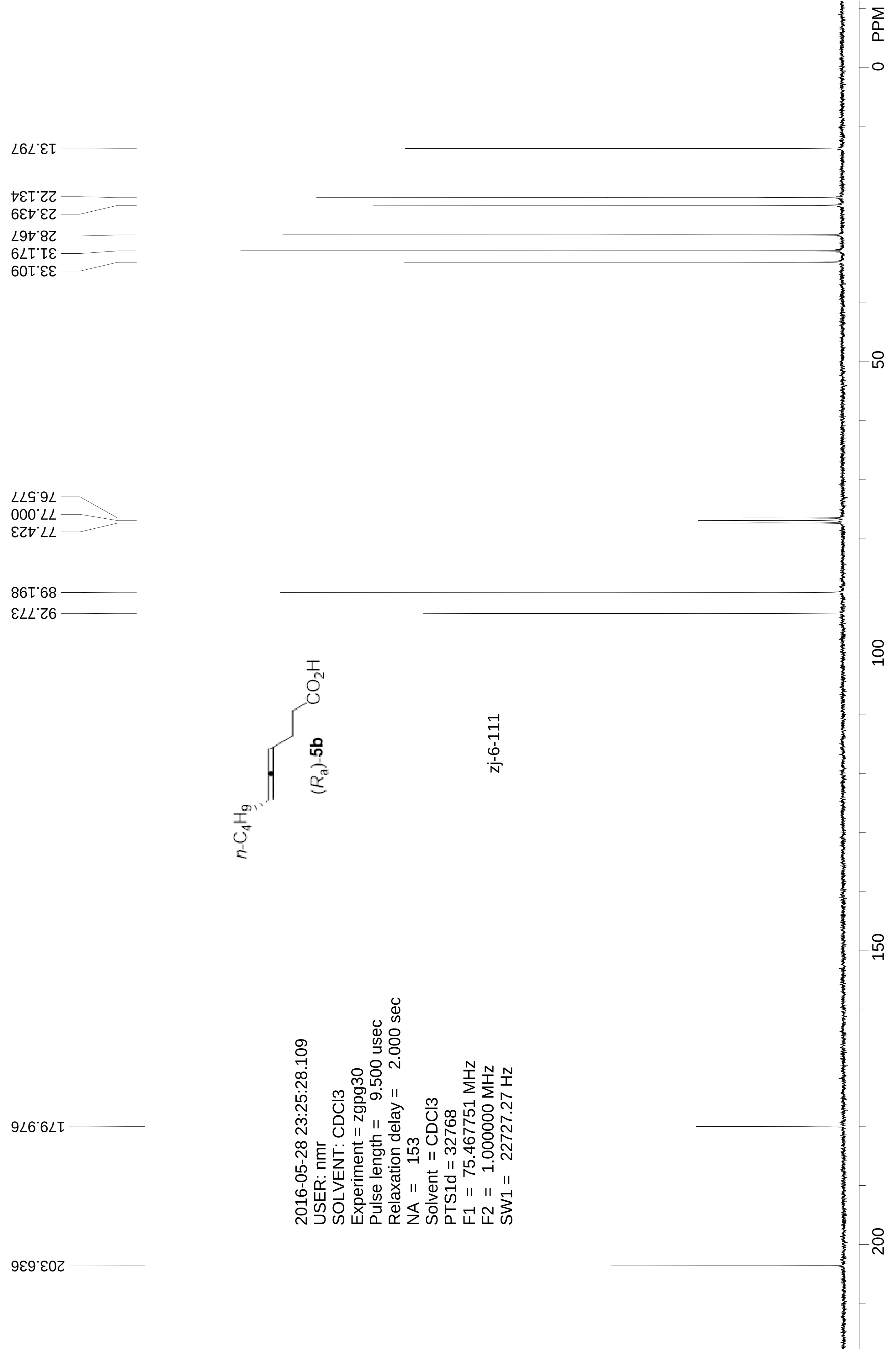

Supplementary Figure 274. **^{13}C NMR (75 MHz, $CDCl_3$) spectrum for ($\textit{R}_a$)-5b.**

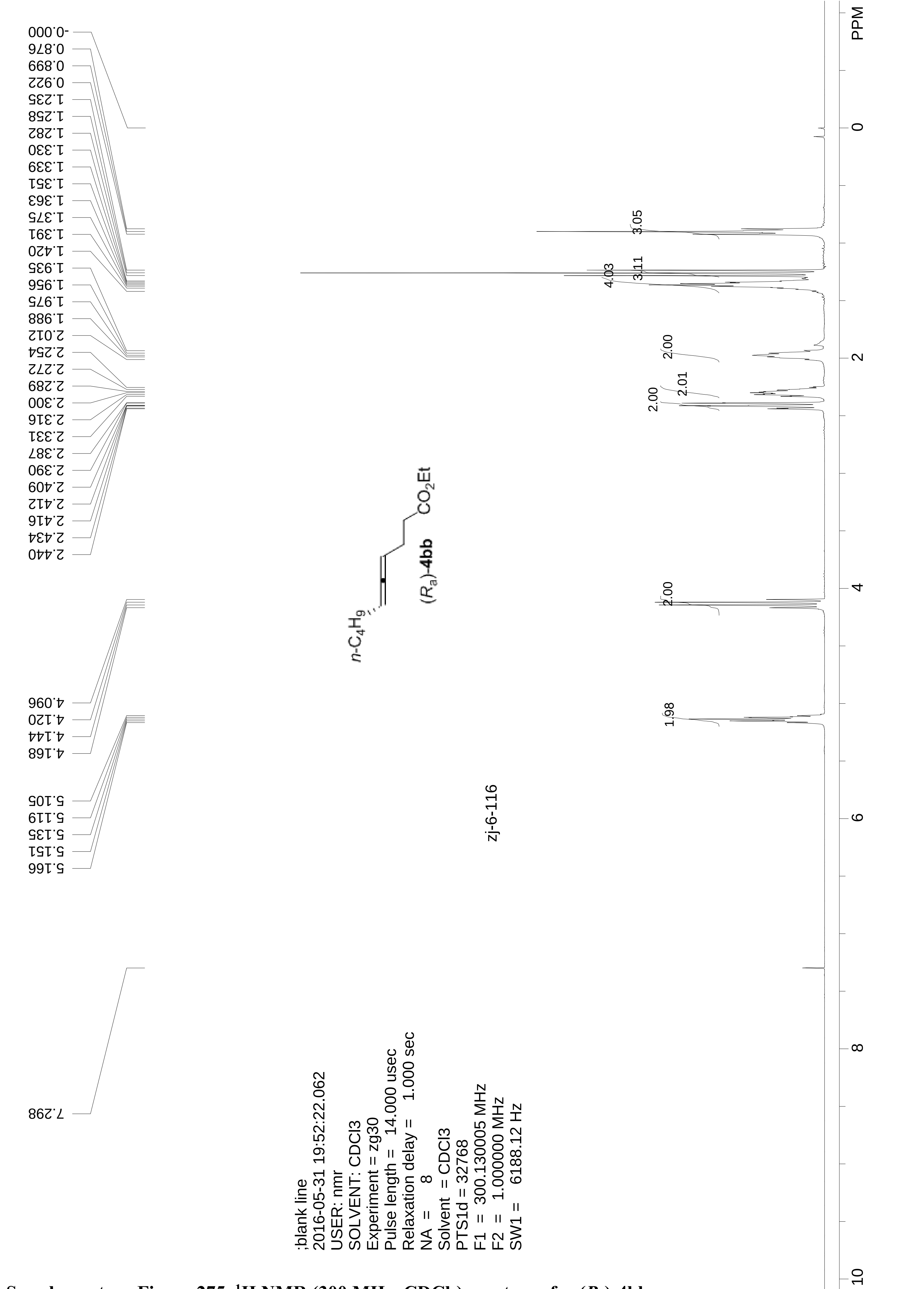

Supplementary Figure 275. ^{1}H NMR (300 MHz, $CDCl_3$) spectrum for (R_a)-4bb.

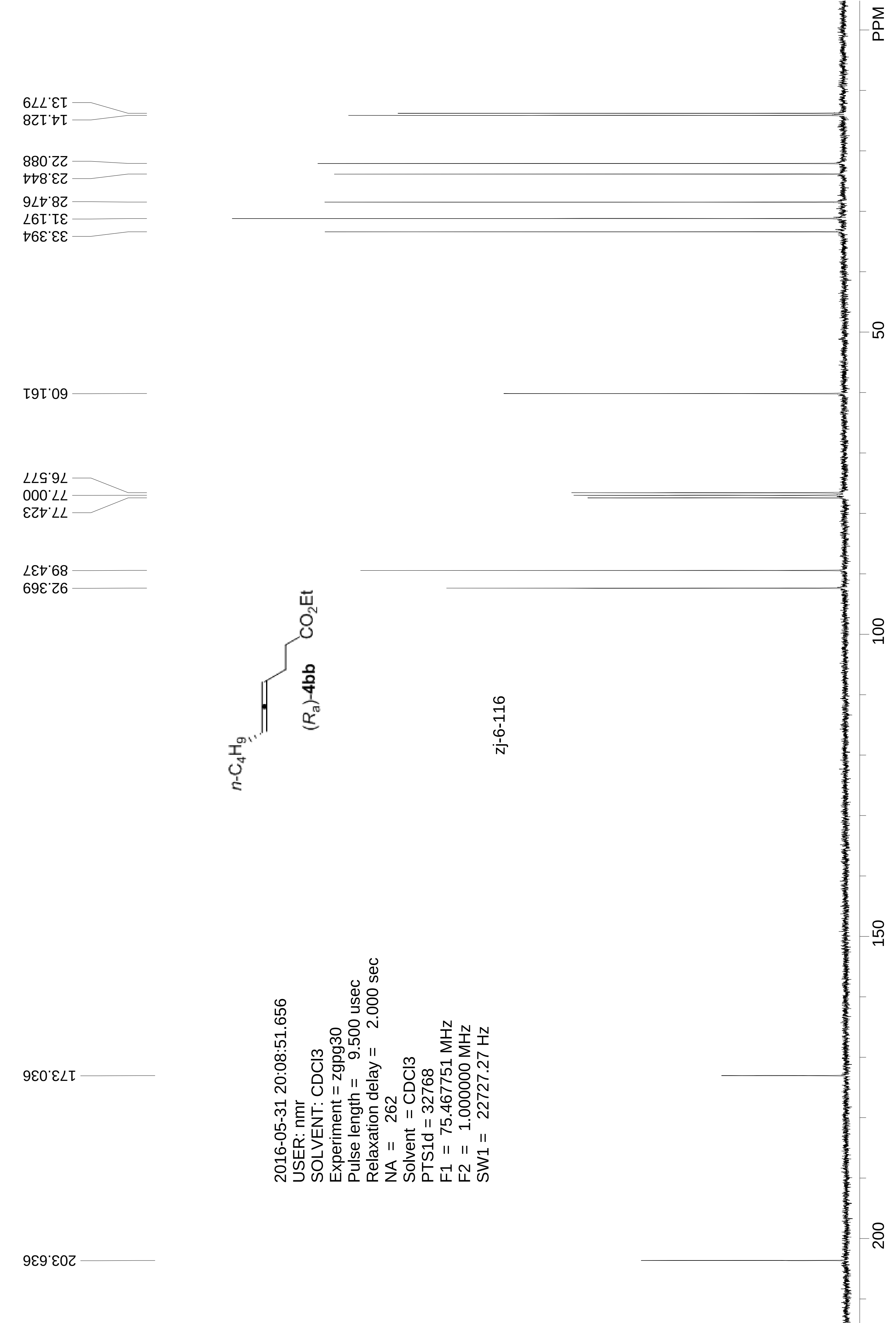

Supplementary Figure 276. ^{13}C NMR (75 MHz, $CDCl_3$) spectrum for (*R*$_a$)-4bb.

zj-6-116-oz-h-100-0-1-214

实验时间：2016-06-07,14:17:17　　　　报告时间：2016-06-07,15:56:32
谱图文件:D:\zhuguangjiong\zj\20160607\zj-6-116-oz-h-100-0-1-214.org

实验内容简介：

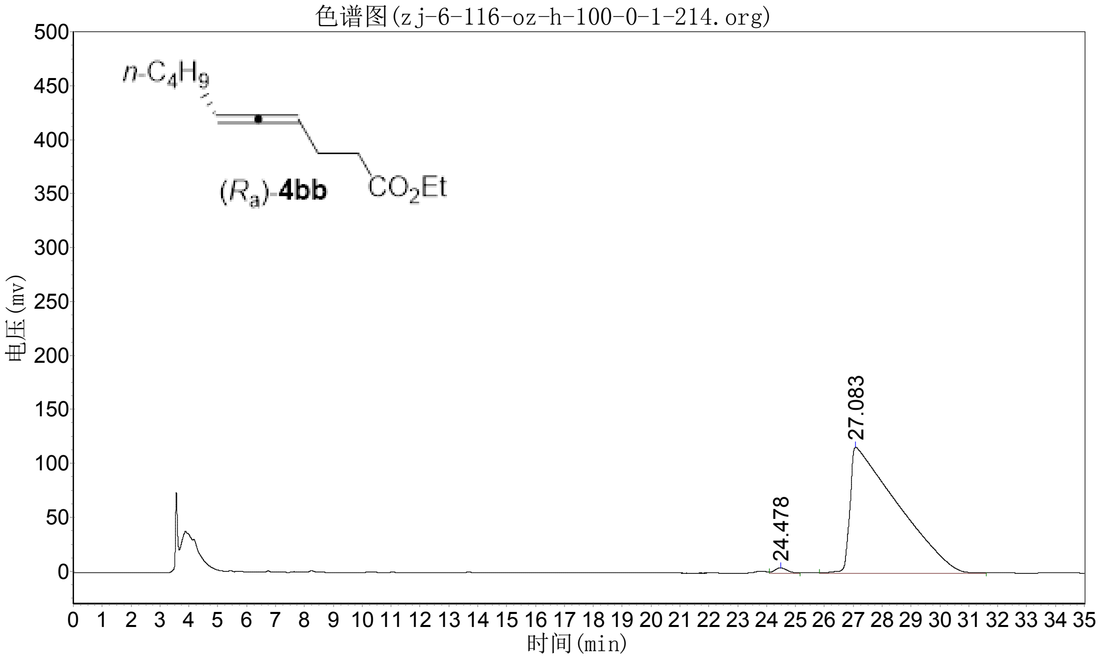

分析结果表

峰号	峰名	保留时间	峰高	峰面积	含量
1		24.478	4888.275	144705.719	1.0675
2		27.083	116701.664	13411313.000	98.9325
总计			121589.939	13556018.719	100.0000

Supplementary Figure 277. HPLC spectrum for (R_a)-4bb.

zj-6-054-oz-h-100-0-1-214

实验时间：2016-06-07,15:06:15　　　　　　　　　　　　　　报告时间：2016-06-07,15:55:26
谱图文件:D:\zhuguangjiong\zj\20160607\zj-6-054-oz-h-100-0-1-214..org

实验内容简介：

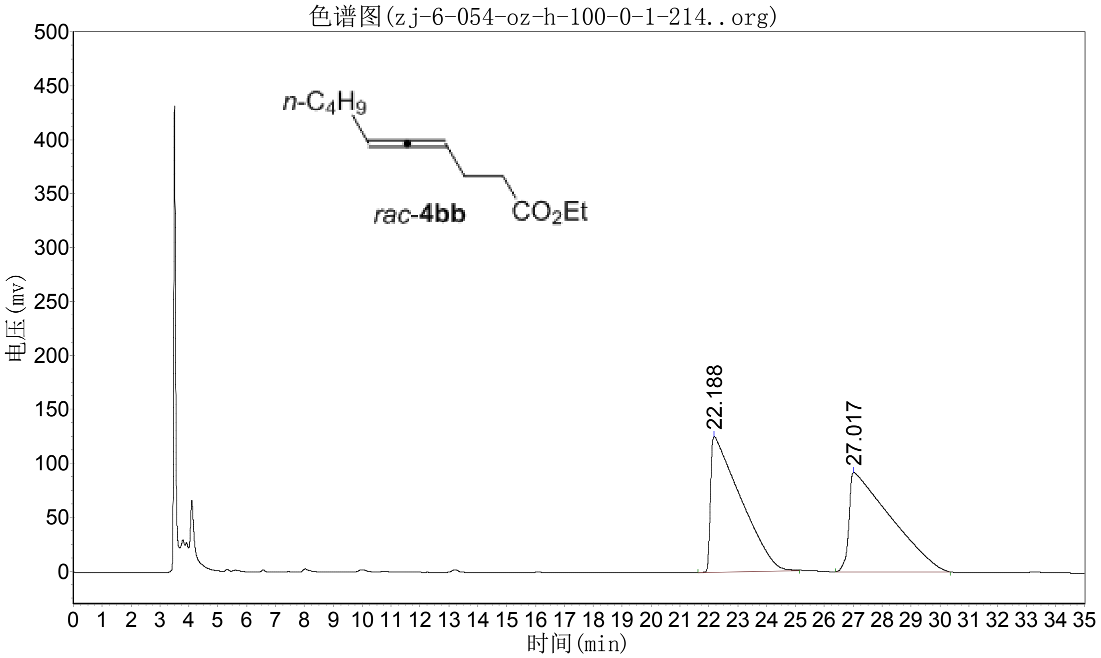

分析结果表

峰号	峰名	保留时间	峰高	峰面积	含量
1		22.188	125608.375	9041449.000	49.5156
2		27.017	91985.625	9218339.000	50.4844
总计			217594.000	18259788.000	100.0000

Supplementary Figure 278. HPLC spectrum for *rac*-4bb.

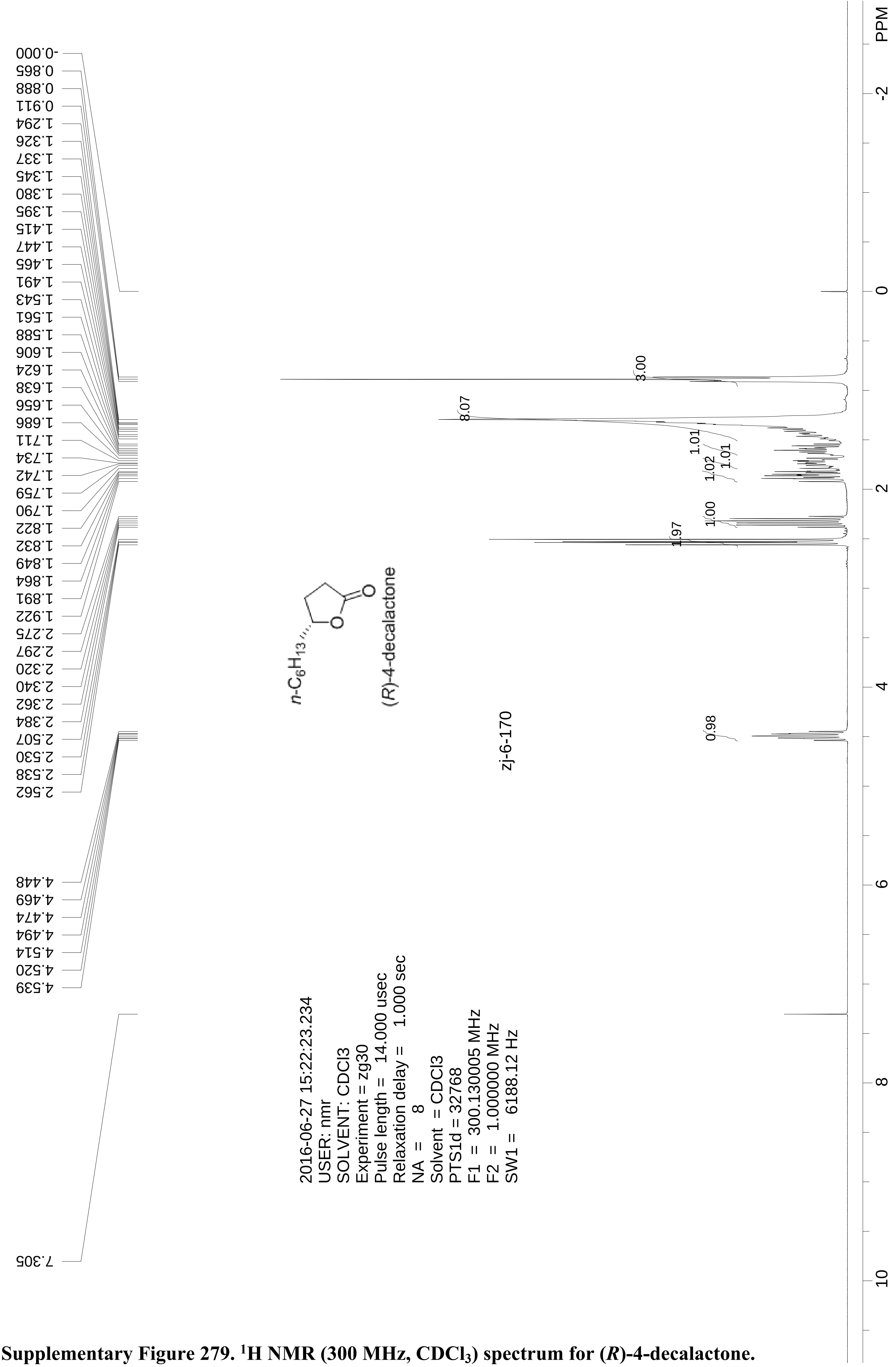

Supplementary Figure 279. **^{1}H NMR (300 MHz, $CDCl_3$) spectrum for (*R*)-4-decalactone.**

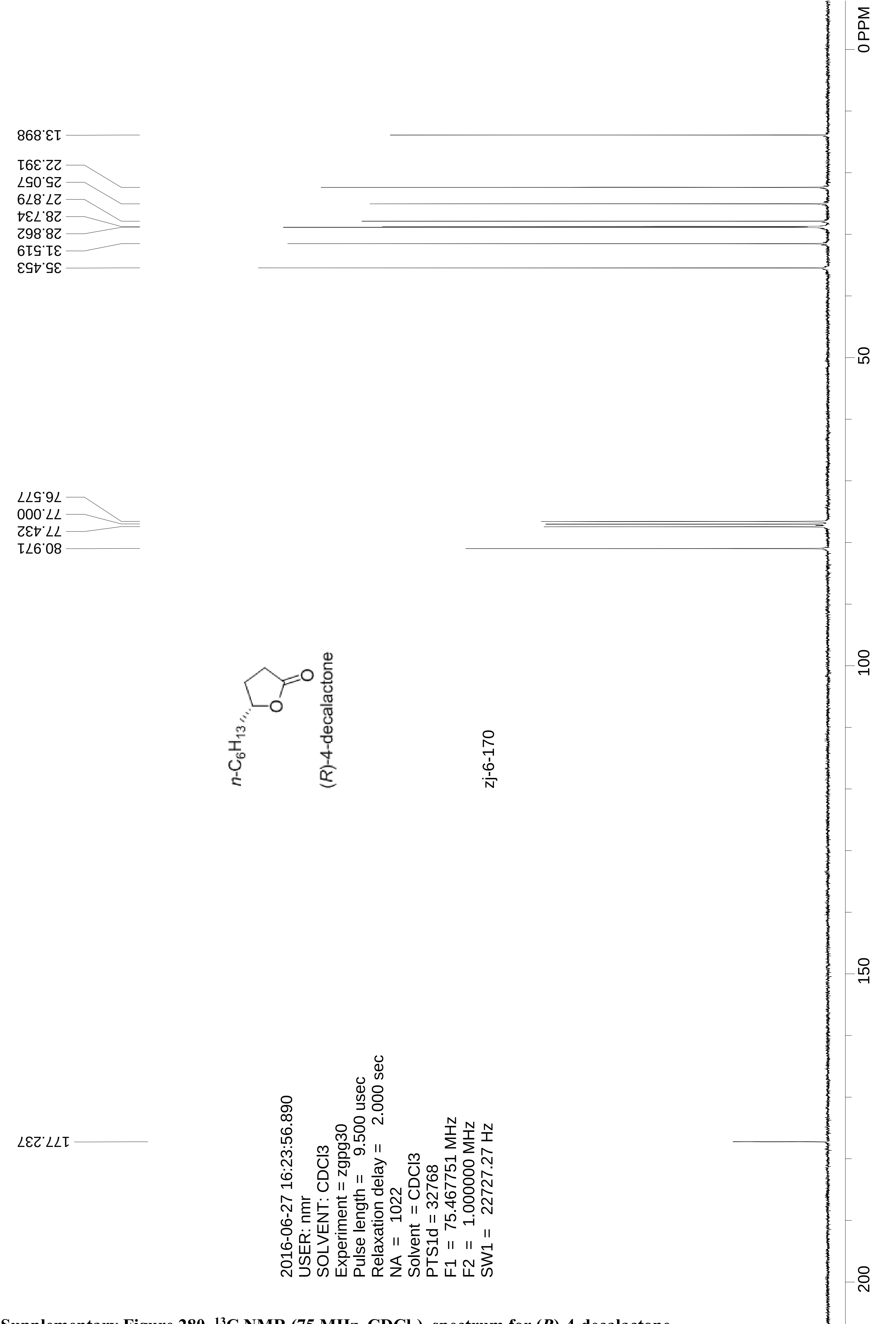

Supplementary Figure 280. ^{13}**C NMR (75 MHz, CDCl$_3$) spectrum for (*R*)-4-decalactone.**

Data File C:\CHEM32\1\DATA\SIG10089.D
Sample Name: zj-6-170

===
Acq. Operator   :
Acq. Instrument : Instrument 1   Location : Vial 1
Injection Date : 7/6/2016 12:49:40 PM
                                                Inj Volume : Manually
Acq. Method   : C:\CHEM32\1\METHODS\DEF_GC-2.M
Last changed   : 7/6/2016 12:49:39 PM
                  (modified after loading)
Analysis Method : C:\CHEM32\1\METHODS\DEF_GC-OFF.M
Last changed   : 7/7/2016 3:26:54 PM
                  (modified after loading)
Sample Info   : dex cb

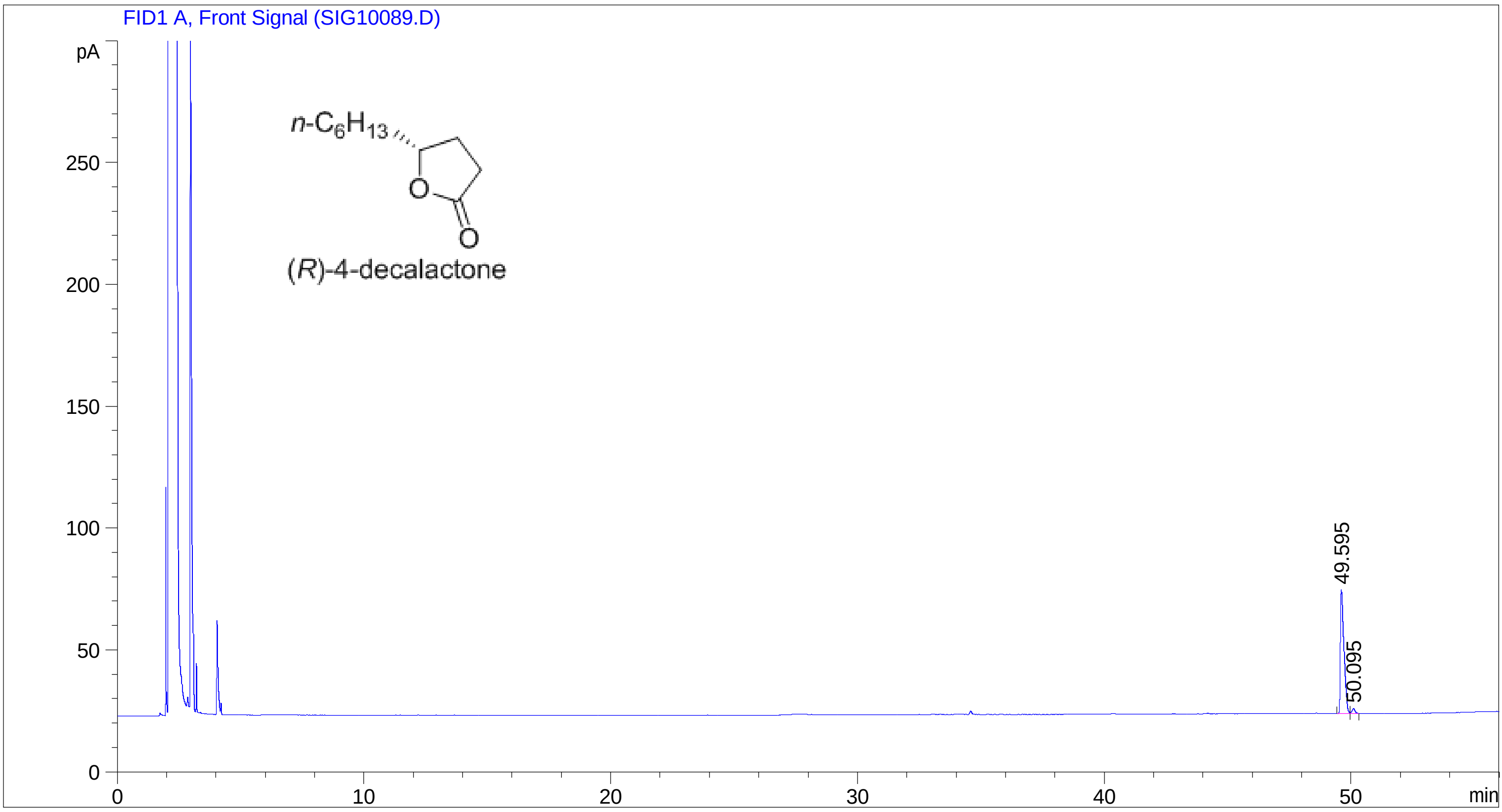

===
Area Percent Report
===

Sorted By   :   Signal
Multiplier: :   1.0000
Dilution: :   1.0000
Use Multiplier & Dilution Factor with ISTDs

Signal 1: FID1 A, Front Signal

Peak #	RetTime [min]	Type	Width [min]	Area [pA*s]	Height [pA]	Area %
1	49.595	MF R	0.1630	509.24765	50.51576	97.03940
2	50.095	FM R	0.1153	15.53679	1.96476	2.96060

Totals :   524.78444   52.48052

Supplementary Figure 281. GC spectrum for (*R*)-4-decalactone.

Data File C:\CHEM32\1\DATA\SIG10088.D
Sample Name: zj-6-161-rac

===

Acq. Operator :
Acq. Instrument : Instrument 1 Location : Vial 1
Injection Date : 7/6/2016 11:22:48 AM
Inj Volume : Manually
Acq. Method : C:\CHEM32\1\METHODS\DEF_GC-2.M
Last changed : 7/6/2016 11:22:46 AM
(modified after loading)
Analysis Method : C:\CHEM32\1\METHODS\DEF_GC-OFF.M
Last changed : 7/7/2016 3:25:28 PM
(modified after loading)
Sample Info : dex cb

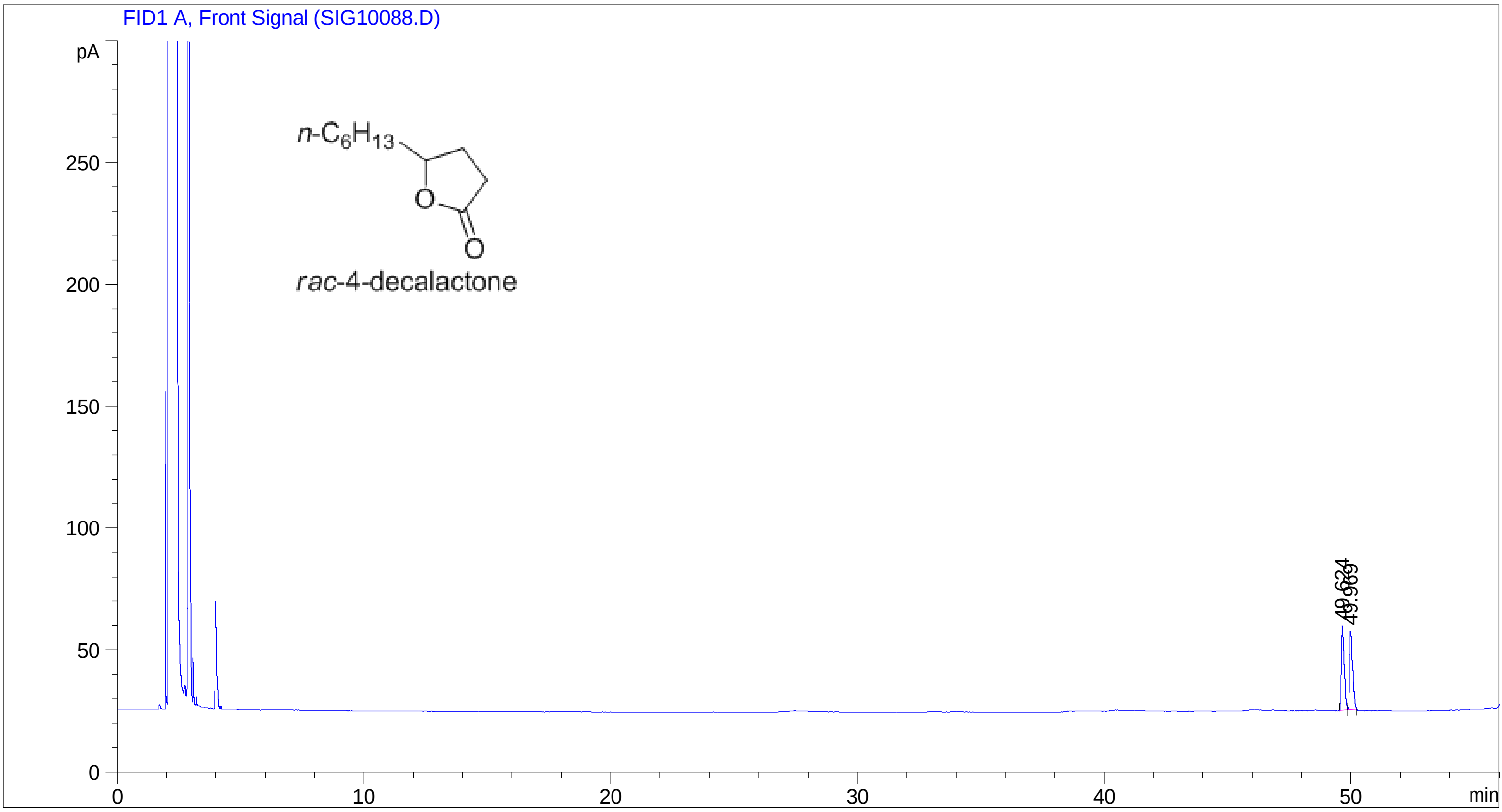

===

Area Percent Report

===

Sorted By : Signal
Multiplier: : 1.0000
Dilution: : 1.0000
Use Multiplier & Dilution Factor with ISTDs

Signal 1: FID1 A, Front Signal

Peak #	RetTime [min]	Type	Width [min]	Area [pA*s]	Height [pA]	Area %
1	49.624	BB	0.1094	282.17673	34.66543	50.30196
2	49.969	BB	0.1158	278.78891	32.32484	49.69804

Totals : 560.96564 66.99027

Supplementary Figure 282. GC spectrum for *rac*-4-decalactone.

Supplementary References:

1. Huang, X; Cao, T.; Han, Y.; Jiang, X.; Lin, W.; Zhang, J.; Ma, S. General $CuBr_2$-catalyzed highly enantioselective approach for optically active allenols from terminal alkynols. *Chem. Commun.* **51**, 6956-6959 (2015).

2. Xu, D.; Li, Z; Ma, S. Novozym-435-catalyzed enzymatic separation of racemic propargylic alcohols. A facile route to optically active terminal aryl propargylic alcohols. *Tetrahedron Lett*. **44**, 6343-6346 (2003).

3. Tellitu, I.; Serna, S.; Herrero, M. T.; Moreno, I.; Dom ńguez, E.; SanMartin, R. Intramolecular PIFA-mediated alkyne amidation and carboxylation reaction. *J. Org. Chem.* **72**, 1526-1529(2007).

4. Zhang, X.; Fu, C.; Yu, Y.; Ma, S. Stereoselective iodolactonization of 4-allenoic acids with efficient chirality transfer: development of a new electrophilic iodination reagent. *Chem. Eur. J.*, **18**, 13501-13509 (2012).

5. Barrot, M.; Fabrihs, G.; Camps, F. Synthesis of [l6,16,16-$^2H_3$] 11-hexadecynoic acid and [15,15,16,16,16-$^2H_5$] (*Z, Z*)-11,13-hexadecadienoic acid and their use as tracers in a key step of the sex pheromone Biosynthesis of the Processionary Moth. *Tetrahedron* **50**, 9789-9796 (1994).

6. Harris, B. D.; Bhat, K. I.; JoulliB, M. M. Synthetic studies of Detoxin complex II: syntheses of Detoxin B1 and B3. *Heterocycles* **1986**, *24*, 1045.

7. Windsor, K.; Genaro-Mattos, T. C.; Kim, H.-Y. H.; Liu, W.; Tallman, K. A.; Miyamoto, S.; Korade, Z.; Porter, N. A. Probing lipid-protein adduction with alkynyl surrogates: application to Smith-Lemli-Opitz syndrome. *J. Lipid Res.* **54**, 2842-2850 (2013).

8. Tungen, J. E.; Aursnes, M.; Hansen, T. V. Stereoselective synthesis of Maresin 1. *Tetrahedron Lett.* **56**, 1843-1846 (2015).

9. Eggleston, D. S.; Chodosh, D. F. Synthesis and X-ray Crystal Structure Determination of Two Pseudopolymorphic Forms of μ-[1,2-bis(diphenylphosphino)ethane] bis [chlorogold(I)]: a Digold(I) DNA Binder. *Inorg. Chim. Acta*, **108**, 221-226 (1985).

10. National Center for Biotechnology Information. PubChem Compound Database;

CID= 92978644, https://pubchem.ncbi.nlm.nih.gov/compound/92978644.

11. Doolittle R. E.; Tumlinson, J. H.; Proveaux, A. T.; Heath, R. R. Synthesis of the sex pheromone of the Japanese beetle. *J. Chem. Ecol.* **6**, 473-485 (1980).

12. Solladie, G.; Stone, G. B.; Hamdouchi, C. Application of the low-valent Titanium reductive elimination of 1,6-dibenzoate-2,4-dienes to the total synthesis of 6(*E*)-5(*S*)-12(*R*)-Leukotriene B_4. *Tetrahedron Lett.* **34**, 1807-1810 (1993).

13. Hulme, A. N.; HowellsStudies, G. E. towards the synthesis of the marine metabolite, Octalaetin A. *Tetrahedron Lett.* **38**, 8245-8248 (1997).

14. Rüttinger, R.; Leutzow, J.; Wilsdorf, M. Wilckens, K.; Czekelius, C. Reversal of selectivity in Gold-catalyzed cyclizations of 3,3-disubstituted 1,4-diynes. *Org. Lett.* **13**, 224-227 (2011).

15. Ma, S.; Liu, J.; Li, S.; Chen, B.; Cheng, J.; Kuang, J.; Liu, Y.; Wan, B.; Wang, Y.; Ye, J.; Yu, Q., Yuan, W.; Yu, S. Development of a general and practical iron nitrate/TEMPO-catalyzed aerobic oxidation of alcohols to aldehydes/ketones: catalysis with table salt. *Adv. Synth. Catal.* **353**, 1005-1017 (2011).

16. Desai, N. B.; Mckelvie, N.; Ramirez, F. A new synthesis of 1,1-dibromoölefins *via* phosphine-dibromomethylenes. The reaction of triphenylphosphine with carbon tetrabromide. *J. Am. Chem. Soc.* **84**, 1745-1747 (1962).

17. Gannett, P. M.; Nagel, D. L.; Reilly, P. J.; Lawson, T.; Sharpe, J.; Toth, B. The capsaicinoids: their separation, synthesis, and mutagenicity. *J. Org. Chem.* **53**, 1064-1071 (1988).

18. Ardolino, M. J.; Eno, M. S.; Morken, J. P. Stereocontrol in Palladium-catalyzed propargylic substitutions: kinetic resolution to give enantioenriched 1,5-enynes and propargyl acetates. *Adv. Synth. Catal.* **355**, 3413-3419 (2013).

19. Nagamitsu, T.; Takano, D.; Marumoto, K.; Fukuda, T.; Furuya, K.; Otoguro, K.; Takeda, K.; Kuwajima, I.; Harigaya, Y.; Omura, S. Total synthesis of Borrelidin. *J. Org. Chem.* **72**, 2744-2756 (2007).

20. Jiang, W.; Liu, D.; Deng, Z.; Voogd, N. J.; Proksch, P.; Lin, W. Brominated polyunsaturated lipids and their stereochemistry from the Chinese marine sponge Xestospongia testudinaria. *Tetrahedron* **67**, 58-68 (2011).

21. Pheromone synthesis. Part 249: Syntheses of methyl (*R,E*)-2,4,5-tetradecatrienoate and methyl (2*E*,4*Z*)-2,4-decadienoate, the pheromone components of the male dried bean beetle, Acanthoscelides obtectus (Say). *Tetrahedron* **68**, 1936-1946 (2012).

22. Krief, A.; Ronvaux, A.; Tuch, A. Catalytic enantioselective synthesis of 2-alkylcyclobutanones and 4-alkyl-γ-butyrolactones from alkylidenecyclopropanes and the sharpless AD-mix reagent. *Bull. Soc. Chim. Belg.* **106**, 699-702 (1997).

23. Leal, W. S.; Kuwahara, S.; Ono, M.; Kubota, S. (*R,Z*)-7,15-Hexadecadien-4-olide, sex pheromone of the yellowish elongate chafer, *heptophylla picea*. *Bioorg. Med. Chem.* **4**, 315-321 (1996).

24. Bravo, P.; Resnati, G.; Viani, F.; Arnone, A. Synthesis of the (4*S*,5*R*)-5-Hydroxy-decan-4-olide (L-Pactor) and of the (*R*)-decan-4-olide from a chiral sulphoxide. *Tetrahedron* **43**, 4635-4647 (1987).
